# Strain-level diversity of giant viruses infecting chlorarachniophyte algae in the subtropical North Pacific

**DOI:** 10.1093/ismejo/wrag093

**Published:** 2026-04-16

**Authors:** Max Emil Schön, Christopher R Schvarcz, Silja V Malkewitz, Fanny C Hinner, Anna Koslová, Ulrike Mersdorf, Fiona Schimm, Sebastian Rickert, Nadiia Pozhydaieva, Kelsey McBeain, Thomas Hackl, Alina Cosima Schneider, Karina Barenhoff, Katharina Höfer, Kyle F Edwards, Grieg F Steward, Matthias G Fischer

**Affiliations:** Department of Biomolecular Mechanisms, Max Planck Institute for Medical Research, 69120 Heidelberg, Germany; Department of Oceanography, University of Hawaiʻi at Mānoa, Honolulu, HI 96822, United States; Department of Biomolecular Mechanisms, Max Planck Institute for Medical Research, 69120 Heidelberg, Germany; Department of Biomolecular Mechanisms, Max Planck Institute for Medical Research, 69120 Heidelberg, Germany; Institute of Molecular Genetics, Czech Academy of Sciences, 14200 Prague, Czech Republic; Department of Biomolecular Mechanisms, Max Planck Institute for Medical Research, 69120 Heidelberg, Germany; Department of Biomolecular Mechanisms, Max Planck Institute for Medical Research, 69120 Heidelberg, Germany; Department of Biomolecular Mechanisms, Max Planck Institute for Medical Research, 69120 Heidelberg, Germany; Max Planck Institute for Terrestrial Microbiology, 35043 Marburg, Germany; Department of Oceanography, University of Hawaiʻi at Mānoa, Honolulu, HI 96822, United States; Groningen Institute for Evolutionary Life Sciences, University of Groningen, 9700 CC Groningen, The Netherlands; Department of Biomolecular Mechanisms, Max Planck Institute for Medical Research, 69120 Heidelberg, Germany; Department of Biomolecular Mechanisms, Max Planck Institute for Medical Research, 69120 Heidelberg, Germany; Max Planck Institute for Terrestrial Microbiology, 35043 Marburg, Germany; Department of Pharmacy, Institute of Pharmaceutical Biology and Biotechnology, Philipps Universität Marburg, 35032 Marburg, Germany; Center for Synthetic Microbiology (SYNMIKRO), Philipps Universität Marburg, 35043 Marburg, Germany; Department of Oceanography, University of Hawaiʻi at Mānoa, Honolulu, HI 96822, United States; Department of Oceanography, University of Hawaiʻi at Mānoa, Honolulu, HI 96822, United States; Department of Biomolecular Mechanisms, Max Planck Institute for Medical Research, 69120 Heidelberg, Germany

**Keywords:** giant virus, algal virus, strain diversity, long read sequencing, virus evolution, DNA methylation, viral infection, protist, virus ecology

## Abstract

Giant DNA viruses are ubiquitous among unicellular eukaryotes and occur in marine, freshwater, and terrestrial environments. Despite intense metagenomic data mining, their strain-level diversity remains largely unexplored. Here we introduce a model system comprising four isolates of a giant virus called ChlorV, which infects marine microalgae of the class Chlorarachniophyceae (Rhizaria) from station ALOHA, Hawai’i. The ChlorV genomes are 469 kbp to 493 kbp long and encode approximately 400 proteins, at least 106 of which are present in purified virions. Although the four viral genomes are highly syntenic, they differ by several insertions and deletions that often encode methyltransferases. We found that some of these methyltransferase genes correlated with specific DNA methylation patterns in the same ChlorV strain. Our study describes the first giant viruses infecting the eukaryotic supergroup Rhizaria and demonstrates how viral strain-level variation in gene content and epigenetic features may affect eco-evolutionary processes in marine microalgae.

## Introduction

Double-stranded DNA viruses of the phylum *Nucleocytoviricota* are the giants of the viral world, with particle sizes of up to 1.5 μm and genome lengths of several megabases [1–5]. When the first such virus, *Acanthamoeba polyphaga* mimivirus, was isolated and analyzed, it showed a remarkable genetic make-up previously unknown from any other virus [6]. Since then, a large number of additional giant virus isolates and thousands of metagenome-assembled genomes have been published. The phylum *Nucleocytoviricota* currently comprises five orders and 15 families [7]; among them, the family *Mimiviridae* (order *Imitervirales*) is the best-studied. The *Mimiviridae* comprises three subfamilies: *Megamimivirinae*, *Klosneuvirinae*, and *Aliimimivirinae*. The *Megamimivirinae* include the original mimivirus and relatives such as tupanvirus, moumouvirus, and megavirus, all of which have been isolated on Amoebozoa hosts such as *Acanthamoeba* or *Vermamoeba* [1, 8–11]. The *Klosneuvirinae* subfamily contains the isolates Bodo saltans virus, Fadolivirus, and Yasminevirus [12–15]. The third subfamily, *Aliimimivirinae*, contains the well-characterized Cafeteria roenbergensis virus (CroV) that infects the marine heterotrophic stramenopile *Cafeteria burkhardae*, and Chlorella virus XW01 that was isolated recently on the freshwater green alga *Chlorella* sp. [16, 17]*.* Nevertheless, the abundance of mimivirid-like sequence fragments found in eukaryotic genomes as well as the inferred co-evolutionary history of giant viruses and protists suggests that most microbial eukaryotes are potential hosts for giant viruses [18, 19].

The marine alga *Bigelowiella natans* belongs to the chlorarachniophytes, a mixotrophic lineage within the otherwise heterotrophic Rhizaria whose members acquired their plastid from a green alga by secondary endosymbiosis [20, 21]. In addition to the nuclear and plastid genomes, they retain a remnant of the green algal nucleus, called the nucleomorph [22, 23]. *Bigelowiella natans* has been studied in the context of plastid evolution and was the first sequenced genome of any rhizarian species [24]. Previous research [25] identified several endogenous viral elements in the *B. natans* genome assembly, suggesting that this alga frequently interacts with both giant viruses and virophages, the latter being smaller dsDNA viruses that depend on giant viruses for their own replication [26]. However, no virus infecting a chlorarachniophyte has been described to date, limiting insight into virus–host interactions in this clade.

Here, we characterize four isolated strains of chlorarachniophyte virus (ChlorV), a new species of virulent giant virus isolated from Pacific waters near Hawai’i. Phylogenetically, ChlorVs cluster with members of the subfamily *Aliimimivirinae*, thus expanding the confirmed host range of giant viruses to include chlorarachniophytes (Rhizaria). We describe the dynamics of ChlorV infection in its algal host and present a comparative genome analysis of the four ChlorV strains including methylation patterns and associated methyltransferase genes.

## Materials and methods

### Isolation of hosts and viruses

Strains of diverse eukaryotic phytoplankton were isolated from seawater samples collected from an oligotrophic open-ocean site (Station ALOHA, 22°45′ N, 158°00′ W) [27], located approximately 100 km north of O‘ahu. For collection dates, see Table S1. Seawater samples were typically enriched with artificial seawater media, f/2 medium, or Keller (K) medium [28, 29] and allowed to incubate in bottles or multiwell plates until cells of interest were identified. These cells were then isolated either by micropipette or by serial dilution. All phytoplankton cultures were maintained at 24–26°C on a 12:12 light:dark cycle with ~30–100 μmol photons m^−2^ s^−1^ of photosynthetically active radiation (PAR).

Small subunit ribosomal RNA (18S rRNA) gene sequencing was used initially to identify algal species. For this purpose, cells were pelleted (4000 RCF for 10 min) and DNA extracted and purified (MasterPure Complete DNA and RNA Purification Kit; LGC Biosearch Technologies) or ZymoBIOMICS DNA Mini Kit; Zymo Research). The near-full length gene was amplified by Polymerase Chain Reaction (PCR, Expand High Fidelity PCR System; Roche) with forward (5′- ACC TGG TTG ATC CTG CC AG -3′) and reverse (5′- TGA TCC TTC YGC AGG TTC AC -3′) primers targeting the 5′ and 3′ ends of the gene [30]. PCR products were cloned prior to sequencing (TOPO TA Cloning Kit for Sequencing; Invitrogen), and the insert was sequenced using four separate primers (M13f, M13r, 502f, and 1174r) [31] using a fluorescence-based Sanger technique (BigDye Terminator; Applied Biosystems) and capillary electrophoresis (3730XL DNA Analyzer; Applied Biosystems) at the University of Hawai‘i at Mānoa’s facility for Advanced Studies in Genomics, Proteomics, and Bioinformatics.

Viruses were isolated from seawater samples collected from the open-ocean Station ALOHA sampling site. Large volumes of seawater were pre-filtered (using either 0.8 μm or 0.45 μm pore size filters) to partially remove cells, and the virus community was concentrated by tangential flow filtration (Millipore Pellicon 2 Mini System) using 30 kDa nominal molecular weight limit (NMWL) filters. The viral concentrates were amended with media (f/2 or K) and added to healthy phytoplankton cultures. The virus-challenged cultures were observed for 1–2 weeks for signs of cell lysis, and lysates that continued to produce a lytic effect after multiple transfers to healthy cells (putatively virus-containing) were stored at 4°C and propagated at least once per month by challenging new cells (1–10% v/v of lysate added per challenge).

### Infection experiments

Chlorarachniophyte cultures were grown in K medium in sterile polycarbonate flasks, either in 250 ml Erlenmeyer flasks, or in 3 L Fernbach flasks. Cultures were grown in an incubator at 24°C, 50 rpm shaking, and in 12 h light (at 1300 to 1500 lux)–12 h dark conditions. Immediately prior to inoculation, host cultures were diluted with K medium to a density of 1.0E+06 cells per ml and aliquoted in 50 ml portions in 250 ml PC Erlenmeyer flasks. Triplicate cultures were inoculated at the onset of the 12 h light cycle with a 1.2 μm-filtered ChlorV suspension that was less than 2 weeks old and had been stored at 4°C. For the detailed ChlorV-1 infection experiment in host strain AL-FL05, the virus–host ratio (VHR) was two (inoculum of 0.5 ml). Three flasks with mock-infected host cultures were incubated as a negative control. The six flasks were sampled every hour for the first 36 h, and again at 3 dpi and 5 dpi. At each timepoint, the densities of cells and free virus particles were analyzed immediately by flow cytometry, and 25 μl of each suspension culture was frozen for later qPCR analysis. For the remaining virus–host strain combinations, we infected triplicate cultures at a VHR of 0.1 (inocula ranged from 25 μl to 1 ml) and analyzed cell and virus particle concentrations by flow cytometry at 0 hpi and 7 dpi.

### Flow cytometry

For cell counts, 200 μl of suspension culture was transferred to a 1.5 ml microfuge tube and analyzed on an Attune NxT Acoustic Focusing Flow Cytometer (ThermoFisher Scientific, Waltham, MA, USA) and proprietary software, with the following instrument settings: Red Laser (RL1-H, voltage 320, threshold 1000) for chlorophyll autofluorescence over side scatter (SSC-H, voltage 350, threshold 1000); blue laser filter slot 1: 488/10 + OD2; 50 μl acquisition volume, 25 μl/min flow rate, 25 μl total acquisition. For virus particle counts, 10 μl of suspension culture was pipetted in a 1.5 ml microfuge tube containing 2 μl of 25% EM-grade glutaraldehyde solution (Sigma Aldrich) and 88 μl of 0.1 μm filtered K medium. After incubation at 4°C for 10 min, 860 μl of 0.1 μm-filtered Tris-EDTA buffer and 40 μl of 100x SYBR Gold solution (Life Technologies Corporation, Eugene, OR, USA) were added. The mixture was incubated at 80°C for 10 min in the dark, allowed to cool to room temperature, and analyzed on an Attune NxT Flow Cytometer with the following settings: Blue Laser (BL1-H, voltage 490, threshold 5000) for SYBR fluorescence over side scatter (SSC-H, voltage 340, no threshold); blue laser filter slot 1: 488/10 (small particle filter); 50 μl acquisition volume, 25 μl min^−1^ flow rate, 25 μl total acquisition. Gates for cells and virus particles were set manually, and concentrations were derived directly from the Attune software.

### Quantitative PCR analysis

Sample lysis for qPCR use was performed by adding 50 μl of 2× lysis buffer (20 mM Tris–HCl, pH 8.0, 2 mM EDTA, 0.002% Triton X-100, 0.0002% SDS, 2 mg ml^−1^ proteinase K) and 25 μl of distilled H₂O to 25 μl of sample (stored at −20°C). The tubes were mixed by vortexing and briefly centrifuged, then incubated for 60 min at 58°C and for 10 min at 95°C. Each 20 μl qPCR measurement consisted of 10 μl PowerTrack SYBR Green Master Mix (ThermoFisher), 7 μl nuclease-free water, 0.5 μl of 100 μM forward primer, 0.5 μl of 100 μM reverse primer, and 2 μl of the lysed sample. For ChlorV-1 detection, primers MCP_ChlorV-1_F (5′- CAG CAA TAC TCC ATC CGA CGG -3′) and MCP_ChlorV-1_R (5′- ACT GTT AGC ACC GCC AAT GT -3′) were used, for detection of ChlorV-2, ChlorV-3, and ChlorV-4 we used primers MCP_ChlorV-2-4_F (5′ GTG CTG CAC CTA CTG TTC CT -3′) and MCP_ChlorV-2-4_R (5′ TAG CAG CTT CCC AAT CAC CG -3′). Samples were analyzed in technical duplicates per 96-well plate, using an Mx3005P Real-Time PCR System (Stratagene) with the following cycling conditions: 1 cycle of 7 min at 95°C, 40 cycles of 15 s at 95°C and 1 min at 60°C, 1 cycle of 1 min at 72°C, and a 55–95°C ramping for dissociation curve analysis.

### Sequencing of ChlorV genomes

ChlorV-1 samples for Illumina and PacBio sequencing were prepared using 0.45 μm filtration (Millipore Durapore 142 mm filter, overlaid with Whatman GF/C filter) followed by tangential flow filtration (30 kDa NMWL; Millipore Pellicon 2 Mini system). Viral particles were purified using centrifugation onto CsCl density cushions (6.5 ml 1.6 g ml^−1^ CsCl solution, overlaid with 2 ml 1.1 g ml^−1^ CsCl solution; 25 000 rpm for 50 min, using SW28 rotor) followed by centrifugation on a CsCl continuous gradient and 0.45 μm filtration (Millipore Sterivex) followed by another round on a CsCl continuous gradient. CsCl in the virus-containing fraction was exchanged with 1× Tris-EDTA (TE) buffer by three rounds of concentration and dilution in a centrifugal ultrafilter (30 kDa NMWL; Millipore Amicon Ultra-0.5) followed by DNA extraction and purification (MasterPure Complete DNA and RNA Purification Kit). Sequencing runs for ChlorV-1 were performed on a MiSeq System (Illumina, 250 bp paired-end) using the Nextera XT library preparation kit at the Georgia Genomics and Bioinformatics Core. PacBio sequencing was performed on a PacBio RS II with P6-C4 chemistry at the University of Washington PacBio Sequencing Services.

For Nanopore MinION sequencing, several liters of ChlorV suspension were centrifuged in Nalgene 1 L centrifuge bottles in a Fiberlite F9-6× 1000 LEX rotor (ThermoFisher) for 20 min at 6000× g, 18°C to remove remaining host cells and debris. The supernatant was concentrated by tangential flow filtration in a Vivaflow 200 0.2 μm PES unit (Sartorius) to 30–50 ml final volume. The concentrate was loaded on a 10–40% (w/v) linear Optiprep gradient (in 50 mM Tris–HCl, pH 8.0, 250 mM NaCl, 2 mM MgCl₂) and centrifuged in 14 ml Ultra-Clear SW40 tubes and an SW40 rotor (Beckman) for 2 h at 100000× g, 18°C. The visible virus band was extracted through the tube wall with a syringe and needle, diluted 3-fold with 50 mM Tris–HCl, pH 8.0, 2 mM MgCl₂ and the virus particles were pelleted by centrifugation (30 min at 20000× g, 18°C) in 1.5 ml microfuge tubes. Virus pellets were pooled, washed, and pelleted again. The final virus sample was resuspended in 200 μl of 50 mM Tris–HCl, pH 8.0, 2 mM MgCl₂ for DNA extraction with the QIAamp DNA Mini Kit (Qiagen) following the manufacturer’s instructions, but eluting in only 50 μl of nuclease-free water. The genomes of ChlorV-1, ChlorV-2, and ChlorV-3 were sequenced using the SQK-LSK114 kit and a MinION FLO-MIN114 flow cell; the genome of ChlorV-4 was sequenced with an SQK-LSK110 kit and a FLO-MIN106 flow cell (Oxford Nanopore Technologies). For sequencing details, see Table S2.

A ChlorV-4 DNA sample that was prepared identically as for Nanopore sequencing was sent to Eurofins Genomics Germany GmbH for sequencing on a NovaSeq 6000 S4 System (Illumina) using the TruSeq DNA PCR-Free library preparation kit.

### Virus genome assembly

Raw Nanopore signals were basecalled with Dorado v0.5.0 (ChlorV-1; duplex mode) using the highest-accuracy model dna_r10.4.1_e8.2_400bps_sup@v4.1.0 (ChlorV-1). For a full list of all basecalling models and versions of dorado see Table S2.

The Nanopore reads were assembled using MetaFlye v.2.9.1-b1780 [32] with options “--iterations 3 --nano-hq” after filtering reads with varying thresholds for length (ChlorV-1: 10000, ChlorV-2: 1000, ChlorV-3: 1000, ChlorV-4: 3000) and basecalling Phred quality score (ChlorV-1: 20, ChlorV-2: 5, ChlorV-3: 15, ChlorV-4: 15). Assemblies were further polished using racon v.1.5.0 with options “-m 8 -x -6 -g -8 -w 500” and medaka v.1.11.3 consensus. If available (i.e. ChlorV-1 and ChlorV-4), Illumina reads were used to polish assemblies using polypolish v.0.6.0 [33].

Average nucleotide identity (ANI) and the corresponding alignment coverage between assemblies and related genomes of isolated viruses was assessed with pyani v0.3.0-alpha [34] (average_nucleotide_identity.py -m ANIb --fragsize 500).

Open reading frames (ORFs) were identified using prodigal v.2.6.3 [35] and GeneMark v.4.32. Both sets of ORFs were then merged, picking longer ORFs in case of overlapping features and disregarding ORFs coding for less than 100 amino acids.

For all assemblies, Nanopore reads were mapped to the final polished viral genomes using minimap2 [36] v.2.24-r1122, and potential single nucleotide variants (SNVs) were identified and quality-filtered using bcftools v1.23 [37].

### Negative stain transmission electron microscopy

ChlorV particles from several liters of infected culture supernatant were concentrated and purified as described above for Nanopore genome sequencing. Iodixanol-pure virus pellets were resuspended in 50 mM Tris-Cl, pH 8.0, 250 mM NaCl, 2 mM MgCl_2_ and washed twice in the same buffer by centrifugation in 1.5 ml microfuge tubes for 15 min at 15 000× g, 10°C.

Six microliters of sample were prefixed with 4 μl of 1% paraformaldehyde (0.4% final concentration) applied and incubated for ~30 min onto a glow-discharged copper grid with carbon-coated Formvar. The paraformaldehyde solution was removed after ~30 min by gentle blotting from one side with filter paper. The grid was rinsed with three drops of water, blotted again, and treated for staining with 10 μl of 0.5% (w/v) uranyl acetate solution for ~15 s. After the staining solution was thoroughly removed by blotting with filter paper, the grid was air-dried. Electron micrographs were imaged on a FEI Tecnai G2 T20 twin transmission electron microscope operated at 200 kV and acquired with a FEI Eagle 4 k HS, 200 kV CCD camera.

### Proteomics sample preparation

A virus suspension with a concentration exceeding 10^10^ PFU/ml was used for the determination of structural proteomics. Virus particles were purified using a linear idioxanol (Optiprep) gradient according to the protocol above for Nanopore sequencing. The purified virions were resuspended in lysis buffer (50 mM Tris–HCl, pH 7.5, 1% SLS, 2 mM TCEP) and heated to 95°C for 10 min. A sonication step (10 s, 20% amplitude, 0.5 pulse) was performed to degrade nucleic acids in the samples. Following this, iodoacetamide was added to the final concentration of 4 mM, and the samples were incubated for 30 min under light protection.

A total of 20 μl each of Sera-Mag carboxylated magnetic beads (Cytivia) and Sera-Mag carboxylated magnetic beads with 0.05% Azide (Cytivia) were mixed, followed by two rounds of washing with 200 μl water and resuspension in 100 μl water. Subsequently, 4 μl of the bead mixture was used for protein purification. To this, a protein sample adjusted to a final acetonitrile (ACN) concentration of 70% (v/v) was added. The mixture was vortexed before the supernatant was discarded after the magnetic separation of the beads. The beads were washed twice with 300 μl of 70% Ethanol, followed by a wash with 200 μl of ACN, and were subsequently dried. For protein digestion, 100 μl of digestion buffer (50 mM ammonium bicarbonate with 1 μg of trypsin) was added to the beads. The mixture was incubated overnight at 30°C and 1200 rpm. The supernatant was collected, and the beads were further washed with 100 μl of water, which was then combined with the initially collected supernatant. To acidify the final sample, containing purified and digested proteins, 30 μl of 5% trifluoroacetic acid (TFA) was added. The resulting supernatant was desalted for mass spectrometric analysis using C18 solid-phase columns (Chromabond C18 spin columns, Macherey Nagel, Düren, Germany). After desalting, the solvent was removed by evaporation, and dried peptides were stored at −20°C until further use.

### Peptide analysis using liquid chromatography and mass spectrometry

Dried peptides were reconstituted in 0.1% TFA and then analyzed using liquid chromatography-mass spectrometry carried out on an Exploris 480 instrument connected to an Ultimate 3000 RSLC nano and a nanospray flex ion source (all Thermo Scientific). Peptide separation was performed on a reverse-phase HPLC column (75 μm × 42 cm) packed in-house with C18 resin (2.4 μm; Dr. Maisch). The following separating gradient was used: 98% solvent A (0.15% formic acid) and 5% solvent B (99.85% acetonitrile, 0.15% formic acid) to 30% solvent B over 45 min at a flow rate of 300 nl min^−1^.

The data acquisition mode was set to obtain one high-resolution MS scan at a resolution of 60 000 full width at half maximum (at m/z 200), followed by MS/MS scans of the most intense ions within 1 s (cycle 1 s). To increase the efficiency of MS/MS attempts, the charged state screening modus was enabled to exclude unassigned and singly charged ions. The dynamic exclusion duration was set to 14 s. The ion accumulation time was set to 50 ms (MS) and 50 ms at 17 500 resolution (MS/MS). The automatic gain control was set to 3 × 10^6^ for MS survey scan and 2 × 10^5^ for MS/MS scans.

For spectral-based assessment, MS raw files searches were carried out using MSFragger embedded within Scaffold 4 (Proteome Software) with 20 ppm peptide and fragment tolerance with carbamidomethylation (C) as fixed, and oxidation (M) as variable modification using the UniProt database.

### Host sequencing and 18S and 28S rRNA gene trees

We extracted genomic DNA from uninfected host cultures. Each sample was then sequenced on an ONT MinION flow cell and basecalled (for specific flow cell and model versions, see Table S3). Sequences were assembled as described below for viral genomes. The minimal read length was 1000 bp, minimal Phred quality score 5 except for AL-FL05 where we used a minimum of 10.

We extracted the full 18S and 28S rRNA gene sequences from the initial assemblies using barrnap [38]. Together with a selection of chlorarachniophytes and an outgroup from Rhizaria (Table S3), each gene was aligned separately using MAFFT-G-INS-i v.7.505 [39] and trimmed using trimAl -gt 0.1 v.1.4.rev15 [40]. Both trimmed alignments were then concatenated and used to reconstruct a phylogenetic tree using IQ-TREE v2.0.3 [41] (--ufboot 1000 --bnni -m MFP [42]), selecting TIM3+F+R3 as the best fitting model.

### Phylogenetic analyses

The seven marker genes A32 genome packaging ATPase, protein-primed DNA polymerase B, large subunit of the RNA polymerase, superfamily II helicase, transcription factor II B, topoisomerase II, and the poxvirus late transcription factor VLTF3 were used for phylogenetic reconstruction. For each marker, DIAMOND protein similarity searches (v.2.0.15.153 [43], BLASTp mode) between the predicted protein sequences and the reference sequences from the giant virus orthologous group (GVOG) database were computed. Alignments were then computed per marker (MAFFT-E-INS-i v.7.505, trimAl -gt 0.1 v.1.4.rev15 [40]) for a taxon sampling including all *Mimiviridae* members and other genomes of *Imitervirales* from the GVDB [44] as an outgroup. Single gene trees were reconstructed using IQ-TREE v2.0.3 [41] (--ufboot 1000 -m MFP [42] -mset LG) and inspected for duplicates, false positives, and other issues. Curated marker sets were re-aligned as above and concatenated. Final tree reconstruction was performed with IQ-TREE (-m MFP --mset LG,LG+C10,LG+C20,LG+C30,LG+C40,LG4X --ufboot 1000), selecting LG+C40+F+I+R10 as the best fitting model.

### Annotation and comparative genomics

Predicted protein sequences were functionally annotated using the eggnog-mapper v.2.1.12 [45] using the eggNOG database v.5.0.2 [46], the GVOG database (using HMMer v.3.3.2 [47]), and InterProScan v.5.62_94.0 [48]. tRNAs were annotated using tRNAscan-SE v.2.0.9 [49]. Taxonomic annotation of the proteins was performed with diamond v.2.0.15.153 [43] against the NR database. Transposable elements were identified using various tools as implemented in the reasonaTE pipeline v.1.0 [50].

The MEME tool v.5.5.0 [51] was used for predicting candidate promoter motifs. First, the upstream sequences of each CDS were partitioned based on the presence of the subsequence “TCTA.” The tool was then run on the sequences not containing “TCTA” and spanning 50 bases upstream and the sequences that do contain this subsequence and spanning 40 bases upstream of a CDS. This partitioning was necessary to improve motif signals, as no transcriptomic data for predicting “early” and “late” genes [16] was present. The two candidate motifs were then tested on all upstream sequences using the tools CENTRIMO and FIMO from the MEME suite v.5.5.0.

For each predicted protein sequence of ChlorV-1, we applied AlphaFold v.3.0.1 [52] to infer its 3D structure. Each predicted structure was then used as a query to search the PDB database using foldseek v.8.ef4e960 [53] “easy-search.” Alignments were visualized with Open-Source PyMOL v.3.1.0 [54]. For the four identified penton proteins and three double jelly roll (DJR) capsid proteins, we additionally performed pentameric or trimeric structural predictions using AlphaFold v.3.0.1, respectively.

All predicted proteins from all isolate genomes and MAGs that were classified in the *Aliimimivirinae* in GVDB [44] were subjected to ortholog identification using OrthoFinder v.2.5.4 [55]. For the different strains of ChlorVs, CroV, and *Chlorella* Virus XW01, all-vs-all protein searches were performed using MMseqs2 [56] v.14.7e284. Based on these searches, genes in ChlorV genomes with at least 70% sequence identity were manually grouped together.

All proteins predicted for the available *B. natans* genome [24] and some additional putative sequences predicted as integrated nucleocytovirus-like genes [25] were compared against the predicted ChlorV genes from all four strains using MMseqs2 [56] v.14.7e284 (easy-search -s 7 -e 1e-3).

### Base modifications

For ChlorV-2,3 and 4, basecalling of modified bases using dorado v.0.7.0 was performed using the context-free models dna_r10.4.1_e8.2_400bps_sup@v5.0.0_4mC_5mC@v3 for 4mC and 5mC methylations and dna_r10.4.1_e8.2_400bps_sup@v5.0.0_6mA@v3 for 6 mA methylations. For ChlorV-1, the rerio [57] models res_dna_r10.4.1_e8.2_400bps_sup@v4.0.1_6mA@v2 and res_dna_r10.4.1_e8.2_400bps_sup@v4.3.0_4mC_5mC@v1 were used. The basecalled reads were mapped to the respective genome using minimap2 [36] v.2.24-r1122 and summarized per-base using modkit [58] pileup v.0.4.2. Modkit motif was used to find short sequence motifs that were enriched in methylations.

For each of the four DNA methyltransferases putatively linked to a methylation motif, we performed additionally AlphaFold 3 structural predictions including DNA Sequences either including the motif or not, and including the protein both as a monomer or as a monodimer.

The genomes were additionally scanned for restriction-modification enzymes and systems using DefenseFinder v.2.0.0 [59] and diamond v.2.0.15.153 searches against the Restriction Enzyme Database [60].

## Results and discussion

In search of new algal viruses from tropical marine environments, we collected samples from surface waters at Station ALOHA, a long-term oceanographic monitoring site located 100 km north of the Hawaiian Island of Oʻahu (Fig. S1). Several strains of chlorarachniophyte algae (Fig. S2) and viruses infecting them were isolated between 2010 and 2012 (Table S1). Because these viruses replicate in members of the Chlorarachniophyceae, we refer to them collectively as “ChlorVs” (for chlorarachniophyte viruses).

All viruses replicated in chlorarachniophyte host strains, albeit with notable differences in strain specificity and virion yield (Table S4). Whereas host strains AL-TEMP06, AL-TEMP07, and AL-DI01 were permissive to all four ChlorV strains, host strain AL-FL05 supported only ChlorV-1 replication and was resistant to the remaining three ChlorV strains. In contrast, host strain AL-FL10 could be infected only by ChlorV-2, but not by the other virus strains. Specific virus–host strain combinations also differed in viral yield by more than one order of magnitude (Table S4), with ChlorV-1 in AL-FL05 producing the highest virion concentrations. Because algal strains AL-FL05 and AL-TEMP06 grew most favorably in our culture conditions, we used them as production strains for ChlorV-1 and ChlorV-2 to ChlorV-4, respectively. All chlorarachniophyte strains used in this study belong to the species *B. natans*, except for strain AL-FL05, which appears to represent the first species of a distinct chlorarachniophyte genus (Fig. S2).

**Figure 1:**
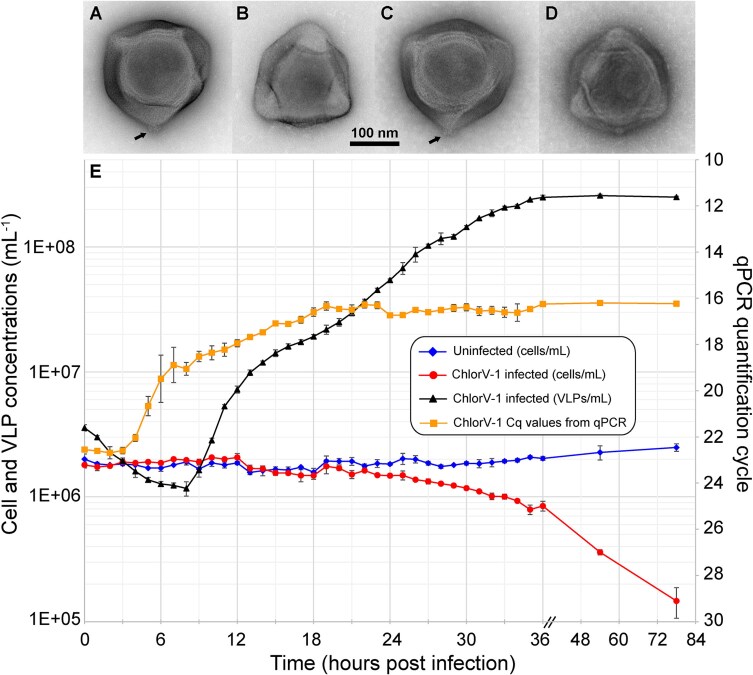
ChlorV particle morphology and infection dynamics. (A–D) Negative stain transmission electron micrographs of ChlorV particles. (A) ChlorV-1. (B) ChlorV-2. (C) ChlorV-3. (D) ChlorV-4. Arrows indicate potential unique capsid structures such as vertex portals. (E) ChlorV-1 infection dynamics in host strain AL-FL05. Densities of host cells and extracellular VLPs of uninfected and ChlorV-1-infected AL-FL05 cultures were measured by flow cytometry. Quantitative PCR was used to monitor viral DNA replication. VLP concentrations and Cq values of uninfected cultures are not plotted, as these were without exception below the limit of detection. Error bars show standard deviations between the three biological replicates and may be smaller than the data symbols.

Extracellular ChlorV particles were readily detectable by flow cytometry after DNA staining with SYBR Gold (Fig. S3). By infecting several liters of algal culture, we collected and concentrated approximately 10^11^ virus particles for each of the four ChlorV strains. Imaging virus particles by negative staining transmission electron microscopy revealed pentagonal or hexagonal outlines typical of icosahedral capsids (Fig. 1A–D). Similar to the two other isolated viruses of the subfamily *Aliimimivirinae* (CroV and Chlorella virus XW01), their capsids appear to be lacking external fibrils. The capsid diameter varied from 200 nm to 230 nm across the four virus strains. Occasionally, small protrusions (13 nm × 5 nm) at one of the capsid corners were visible, which suggests a modified vertex that could function in host recognition or genome release (Fig. 1A and C) [61, 62].

### Infection dynamics

We analyzed the infection dynamics of ChlorV-1 in host strain AL-FL05 because this combination resulted consistently in the highest virus titers when compared to any other ChlorV–host strain combinations. We exposed the algal cultures to a 12 h light–12 h dark cycle and infected AL-FL05 with ChlorV-1 at the onset of the light cycle. Triplicate cultures of AL-FL05 were infected with freshly prepared ChlorV-1 at a virus-to-host ratio of two (virus particles measured by flow cytometry) and monitored hourly for the first 36 h by flow cytometry and quantitative PCR using ChlorV-specific primers (Fig. 1E). The qPCR signal remained constant from the time of infection until 4 h post infection (hpi); thereafter, the quantification cycle (Cq) decreased from 22.5 to 16.0 by 19 hpi, indicating the onset of viral DNA replication around 3–4 hpi and a peak in viral DNA by ~19 hpi. Using flow cytometry, we observed a continuous decrease in extracellular virus-like particles (VLPs) from 0 h post infection (hpi) to 8 hpi, most likely due to the uptake of ChlorV-1 particles by host cells. Following this latent period, VLP concentrations in the culture medium increased steadily from 9 hpi to 36 hpi. The slope of VLP increase was steeper toward the end of the first light cycle (8–12 hpi) than during the following dark and light cycles (13–36 hpi). Furthermore, we observed a delayed decline of host cells in the infected cultures, which occurred only after extracellular VLP concentrations had reached their maximum at 36 hpi. These data are not compatible with a one-step growth curve and instead suggest a constant release of virus particles. In summary, our infection experiments show that ChlorV-1 is a lytic virus; however, the release of progeny virus particles is not immediately linked to host lysis, suggesting a continuous and, at least initially, non-destructive exit strategy, such as budding from host membranes or exocytosis [63].

### Genome features

We generated high-quality genome assemblies for all viral strains using ultra long MinION sequencing reads at 31× to 700× coverage (Table 1). For ChlorV-1 and ChlorV-4, we also obtained short-read Illumina sequencing data to further polish the assemblies. The genomes, which were each assembled as a single contiguous sequence, appear to have a linear topology, as they contained short (1.5 kbp to 2.5 kbp) terminal inverted repeats at both ends, and ranged in size from 469 kbp (ChlorV-3) to 493 kbp (ChlorV-4). Whereas the genomes of ChlorV-2 and ChlorV-4 were virtually identical with an ANI exceeding 99.8%, ANIs between other pairs of genomes ranged between 93.2% (ChlorV-1 vs. ChlorV-4) and 97.3% (ChlorV-3 vs. ChlorV-4; Fig. S4). The differences (insertions/deletions) between genomes were distributed over their complete lengths, and none of the observed differences were longer than 10 000 bp. In comparison, the ANI between any of the four ChlorV genomes and their most closely related giant virus isolates, CroV and Chlorella virus XW01, was ~70%. However, the alignments between Chlorella virus XW01 and ChlorVs covered ~10%–12% of their respective genomes, whereas those between CroV and the ChlorVs covered only ~4%–6% (within ChlorVs, the coverage ranged from 75% to 100%, Fig. S4).

**Table 1:** Genome statistics of ChlorV strains 1–4. ONT reads were used to create the initial assemblies, which were then polished using Illumina data if available. PacBio data for ChlorV-1 were only used to validate the ONT-based assembly.

Strain	Genome length (bp)	GC (%)	ORFs	Coverage (ONT)	Data source	Accession
ChlorV-1	487 585	19.05	402	263	ONT + PacBio + Illumina	GCA_974392635
ChlorV-2	490 841	18.54	409	639	ONT	GCA_975145565
ChlorV-3	468 809	18.82	391	700	ONT	GCA_976281805
ChlorV-4	493 487	18.59	394	31	ONT + Illumina	GCA_975141545

In each of the four ChlorV genomes, we identified ≈400 ORFs encoding proteins ≥100 amino acids, with 56 to 72 sequences per genome not matching any known cellular or viral genes (ORFans). Accounting for ORFs that were split in some assemblies, 396 non-redundant genes were identified across all virus strains. A core genome of 307 genes was shared across all strains, whereas 381 were shared between ChlorV-2 and ChlorV-4, 336 between ChlorV-2, ChlorV-3, and ChlorV-4, and 316 between ChlorV-1 and ChlorV-3. None of the viruses showed significant genetic heterogeneity in terms of SNVs, with the average number of SNVs ranging from 0.006 to 0.116 SNVs/kbp (3 to 57 SNVs in total per strain).

### ChlorV phylogenetic position

To establish the phylogenetic position of these viruses, we identified putative homologs of established marker genes specific for viruses of the phylum *Nucleocytoviricota* [44]. Each ChlorV genome contained a full complement of these conserved genes, further validating the quality of the assemblies. For each marker gene, we reconstructed a phylogenetic tree with broad taxonomic sampling across the *Nucleocytoviricota*. In each of these trees, the four ChlorV genomes formed a tight cluster in the *Mimiviridae* family, with CroV and Chlorella virus XW01 being the closest related isolates (Supplementary File 1).

**Figure 2:**
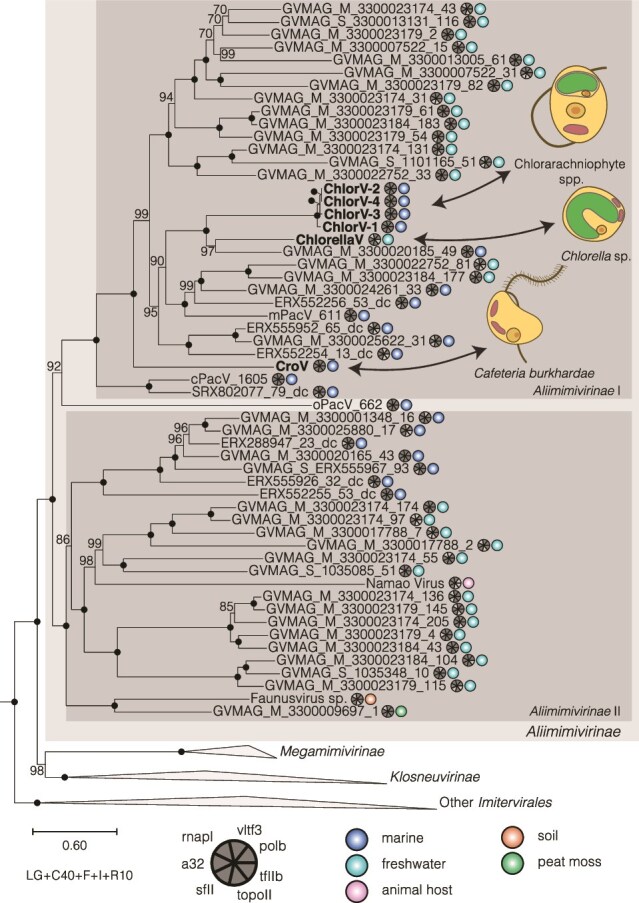
Phylogenetic position of ChlorV isolates. ChlorV strains 1–4 form a well-supported and closely related group within the *Mimiviridae* subfamily *Aliimimivirinae*. Their most closely related isolates are Chlorella virus XW01 and CroV of the same subfamily. The tree is based on the concatenated alignment of seven marker genes and was inferred under the best fitting evolutionary model LG+C40+F+I+R10, whereas support was estimated using 1000 ultrafast bootstrap replicates. Besides the phylogenetic position, the distribution of marker genes per genome is drawn as a Coulson plot and the source environment as a colored circle.

We reconstructed a phylogenetic tree based on a concatenated alignment of seven marker proteins, focusing on genomes of *Mimiviridae* members (isolates and environmental genomes) from the giant virus database (GVDB). Selected representatives of other *Imitervirales* lineages were chosen as an outgroup. The ChlorVs formed a well-supported monophyletic group with Chlorella virus XW01, CroV, and several environmental genomes in the subfamily *Aliimimivirinae* (Fig. 2).

The addition of ChlorVs makes the *Aliimimivirinae* one of the most diverse groups of *Nucleocytoviricota* with respect to host range. Three different eukaryotic supergroups (Rhizaria: Chlorarachniophytes; Stramenopiles: *Cafeteria*; Archaeplastida: *Chlorella*) are infected by members of this subfamily. In fact, the ChlorVs, CroV, and Chlorella virus XW01 cluster in one group within the *Aliimimivirinae* (*Aliimimivirinae* I), whereas the lake sturgeon-associated Namao virus and various metagenome-assembled genomes form a second group (*Aliimimivirinae* II), indicating two well-supported clades within *Aliimimivirinae* (here referred to as clades I and II; Fig. 2, Fig. S5).

### Genome annotation and comparative genomics

We annotated the predicted protein sequences by combining several tools and databases, including EggNOG, InterPro (incorporating PFAM), and the GVOGs [44–46, 48]. Besides ORFans with no matches to any known sequences, 128–146 ORFs per genome only matched poorly characterized reference sequences, often predicted from other giant viruses. Of the 173–190 sequences with a robust annotation, most were categorized as “hydrolases” (e.g. peptidases or nucleases), “replication, transcription, and DNA repair” (e.g. helicases, replication factors, polymerases), or “transferases” (e.g. methyl- and glycosyltransferases) (Table S5). The ChlorV genomes encode a full complement of DNA replication genes, including a family B DNA polymerase (ChlorV-1..065), a DNA clamp (ChlorV-1..016), and clamp loader (replication factor C, e.g. ChlorV-1..164). They further encode a DNA ligase (ChlorV-1..107) and a ribonuclease H (ChlorV-1..329) as well as multiple helicases including a D5-like helicase-primase (ChlorV-1..361) and two subunits of a ribonucleotide reductase (ChlorV-1..059 and ChlorV-1..370). Transcription-related enzymes are also encoded in the genomes, including several subunits of the RNA polymerase (Rpb1,2,3/11,5,6,7, ChlorV-1..202039325253359251), an mRNA capping enzyme (ChlorV-1..311), a poly(A) polymerase (ChlorV-1..312), and several transcription factors including homologs of the Poxvirus VLTF2 (ChlorV-1..152) and VLTF3 (ChlorV-1..384). Several translation initiation (e.g. eIF4E, ChlorV-1..017) and elongation (EF-Tu, ChlorV-1..328) factors are also present. A homolog of the DNA polymerase beta (ChlorV-1..031) that is involved in base excision repair (BER), a putative DNA mismatch repair protein MutS (ChlorV-1..354), and two DNA photolyases (ChlorV-1..224 and ChlorV-1..336) that are potentially involved in DNA repair outside of the host are encoded as well. Additional proteins include several peptidases (e.g. ChlorV-1..086) and ankyrin repeat proteins (e.g. ChlorV-1..071). A putative YqaJ-like viral recombinase (ChlorV-1..348) has homologs that cleave genomic concatemers in some bacteriophages [64]. A putative tubulin–tyrosine ligase (ChlorV-1..043) could be involved in the manipulation of host cells [65]. Several glycosyltransferases (e.g. ChlorV-1..212) and glycosylases (e.g. ChlorV-1..397) are encoded in the genomes of all strains and could be involved in capsid modifications and host interactions [66]. At least one acetyltransferase (ChlorV-1..005) and one aminotransferase (ChlorV-1..145) were also present.

**Figure 3:**
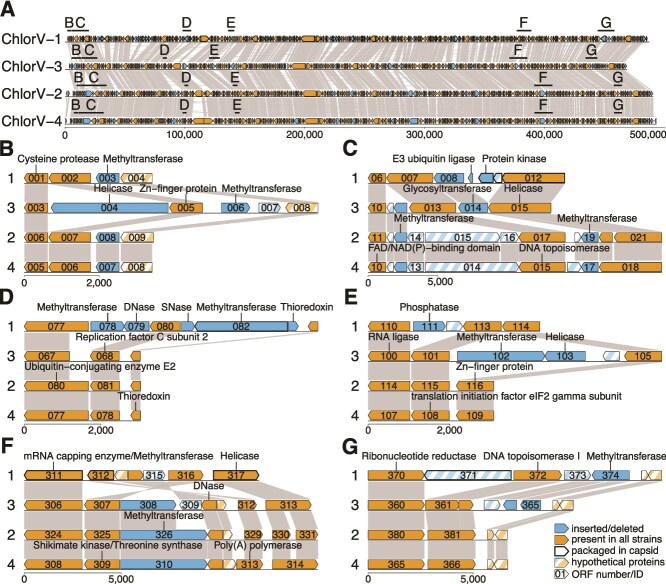
ChlorV strain level genomics. (A) Overview of the complete genomes from four strains of Chlorarachniophyte viruses (ChlorVs 1-4), with strain-specific regions highlighted by letters B to G. (B–G) Details of genomic loci where putative insertions/deletions and an inversion (F) were observed. Genes with significant sequence matches are connected by ribbons. DNase: deoxyribonuclease, SNase: staphylococcal nuclease (DNA and RNA endo-exonuclease).

Each ChlorV genome encoded multiple predicted methyltransferase genes (12 in ChlorV-3, 11 in the other strains), putatively modifying diverse target molecules such as DNA (ChlorV-1..078), RNA (e.g. the FtsJ-like methyltransferase ChlorV-1..022), proteins (ChlorV-3..133), and others (e.g. the FkbM family methyltransferase ChlorV-4..007). These genes were often located in strain-specific insertion/deletion loci (Fig. 3A–G), in contrast to other genes that were usually conserved across all strains. Several types of nucleases (both RNases and DNases) were also common throughout the genomes, being either shared among strains or strain specific (e.g. the DNA endonuclease ChlorV-1..079, Fig. 3D and F).

We predicted the tertiary structure of all ChlorV-1 ORFs and additional ORFs encoded by other strains using AlphaFold 3, and then compared them to experimentally derived protein structures from public databases to improve the genome annotation (Supplementary File 2, Table S6) [52, 53]. For most ORFs, the sequence-based and structure-based annotations (where available) agreed well. Occasionally, the structural comparison provided annotations for ORFs with no sequence-based annotation, e.g. for the endonuclease ChlorV-1..079 or the putative penton protein ChlorV-1..095 (Table S6). In other cases, sequence-based annotations were not supported by the structural comparison, e.g. the predicted homing endonuclease ChlorV-1..154. In addition to protein-coding genes, ChlorV strains encoded up to seven tRNA genes, compared to 48 tRNA genes in CroV, and none reported for Chlorella virus XW01.

We investigated putative promoter sequences of ChlorV genes and compared them to previously described motifs from CroV and other giant viruses [16, 67, 68]. We identified the AT-rich early motif “AAAAATTGA” that is highly conserved in mimiviridids, as well as the late motif “TCTA” that is shared in sequence and position only with its distant neighbor CroV (Fig. S6).

We compared the predicted ChlorV gene sequences with putative nucleocytovirus-like genes and cellular genes in the genome of *B. natans* [24, 25]. There are several homologous genes shared between ChlorVs and the host (Table S7), although most of these are likely cellular genes (e.g. ChlorV-2..233, identified as a eukaryote-like gene in Table S5). Previous research identified 36 putative nucleocytovirus-like genes in the *B. natans* genome based on amino acid sequence homology searches, including a transcriptionally silent region of 165 kbp that contained nine nucleocytovirus-like genes [25]. We identified eight distinct genes in each strain (nine in ChlorV-1) with similarity to any of these 36 putative integrated nucleocytovirus-like genes. These matches all displayed low amino acid sequence identities (below 35%), and mostly corresponded to nucleocytovirus core genes encoding for capsid proteins, genome packaging ATPases of DNA polymerases, where homology to distantly related nucleocytoviruses is expected (Table S7, column “NCLDV”).

### Genome modifications

**Figure 4:**
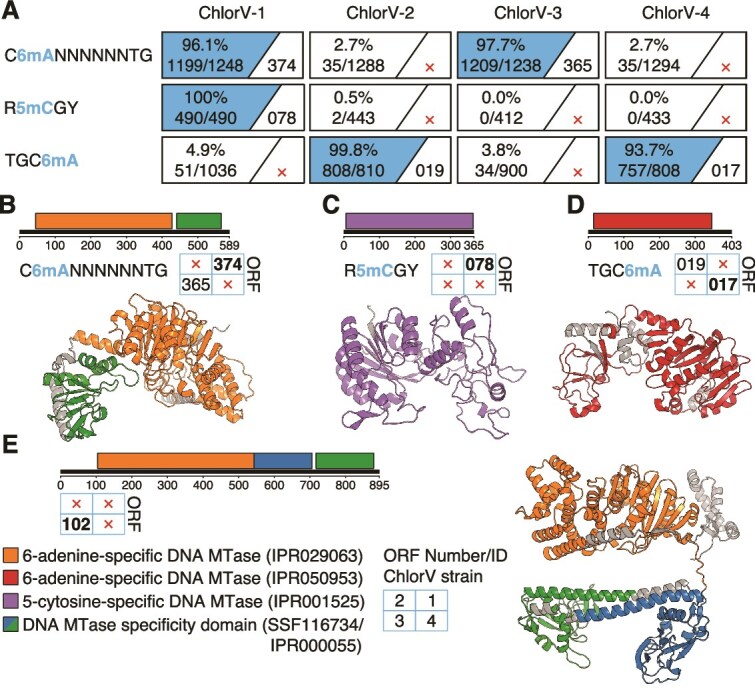
DNA modifications in ChlorV genomes. (A) Sequence motifs with strong (>99%) modification signals in at least one ChlorV strain are shown on the left. Numbers in boxes indicate how many occurrences of the motif were methylated (left) and gene identifiers of any putative associated methyltransferases (right). For ChlorV-3, ORF ChlorV-3..102 is an additional candidate to ORF ChlorV-3..365 for methylation of the CANNNNNNTG motif, although no homolog of ChlorV-3..102 could be identified in ChlorV-1. (B–E) Domain-level annotations and structural models of the four predicted ChlorV methyltransferases: three specific for 6-adenine and one for 5-cytosine. Numbers in boxes refer to gene identifiers in ChlorV-1 (first quadrant) to ChlorV-4 (fourth quadrant), with IDs in bold indicating which domain/structure model is shown. pTM scores for the predicted structures are 0.89 for ChlorV-1..374, 0.94 for ChlorV-1..078, 0.89 for ChlorV-4..017, and 0.61 for ChlorV-3..102.

We used Nanopore sequencing data to analyze DNA base modification patterns in the ChlorV genomes. The dominant modification in each assembly was N6-methyladenine (6 mA), followed by C5-methylcytosine (5mC) and N4-methylcytosine (4mC). The latter two were rare, except for ChlorV-1 where 5mC was comparatively frequent (Fig. S7). This pattern confirms previous observations that giant viruses display prokaryote-like DNA modifications with 6 mA being the dominant type [69]. We identified three sequence motifs that were highly enriched (modified in >90% of occurrences) in methylated bases across the four genomes, including two motifs that were shared by two genomes each (Fig. 4A). All motifs were palindromic; accordingly, we did not detect strand-specific differences. ChlorV-1 and ChlorV-3 displayed a 10-base-pair motif (CANNNNNNTG) that resembled a bipartite recognition sequence, consistent with Type I or certain Type II systems [60, 70]. We identified one gene that was present in ChlorV-1 and ChlorV-3, but not in other strains, as a candidate methyltransferase targeting this sequence (Fig. 3G). It possessed a 6-adenine-specific methyltransferase domain as well as a single target specificity domain similar to other type I modification genes (Fig. 4B). However, this enzyme could also function as a homooligomer, recognizing and modifying the same DNA sequence on opposite strands, like the type IIB system HaeIV [71]. This is supported by the high confidence scores (ipTM = 0.8, pTM = 0.85) of a modeled protein-DNA complex containing two copies of this protein together with a DNA sequence containing the motif CANNNNNNTG, but lower confidences (ipTM <0.8) if using only a single copy or another DNA sequence (Table S8, Fig. S8). ChlorV-3 (but not ChlorV-1) encoded another candidate methyltransferase with a similar DNA methyltransferase domain and two specificity domains, more similar to typical type I modification genes [72] (Fig. 4E). We also identified a motif for 5mC methylation (RCGY) in ChlorV-1. This motif could be the target of a putative 5-cytosine-specific methyltransferase (ChlorV-1..078) encoded only by ChlorV-1, which was adjacent to an endonuclease (ChlorV-1..079), suggesting the presence of functioning restriction-modification system (Figs 3D and 4C). Finally, we identified a different 6 mA motif (TGCA) in ChlorV-2 and ChlorV-4. This motif could be putatively linked to a 6-adenine-specific methyltransferase gene that resembled methyltransferases of type IV restriction-modification systems and was encoded only in these two strains (Figs 3C, 4A and D).

### Virion proteomics

We analyzed the protein composition of purified ChlorV-1 virions by mass spectrometry and identified 106 viral proteins, each represented by at least two peptides (see Table S9 for abundance estimates). Virion proteins were encoded by genes that are spread fairly evenly across the viral genome (Fig. S9). However, nine of the ten most abundant virion proteins were encoded by genes that are located within a 90 kbp central region of the genome, indicating a non-random distribution of important structural genes. The most abundant protein was the DJR major capsid protein ChlorV-1..153, followed by the major core protein ChlorV-1..167 that is homologous to the core proteins of poxvirus (P4B) and CroV (Crov332). The third-most abundant virion protein was ChlorV-1..175, a 709 amino acid long hypothetical protein that lacked homologs in other ChlorV strains (both by sequence- and structure-based searches; Table S6). Peptides for all three additional DJR capsid paralogs, as well as for two of the four single jelly roll (SJR) penton protein candidates, were also found. We generated structural predictions for three of the four DJR proteins as trimers and for the four SJR proteins as pentamers (Figs S10 and S11). The structures were generally predicted with high confidence, except for some surface protrusions that might interact with other capsid proteins. The peculiarly structured SJR protein ChlorV-1..087 had a lower confidence value, although the core penton-like structure was still resolved with good support (Fig. S11). We did not predict hetero-multimers for pseudo-hexameric capsomers involving more than one DJR protein, although it is quite possible that such capsomers exist in ChlorVs, similar to *Paramecium bursaria* chlorella virus 1 (PBCV-1) [73].

Among virion proteins with predicted enzymatic functions, we identified the two predicted DNA photolyases ChlorV-1..224 and ChlorV-1..336. As is typical for mimivirids, ChlorV-1 packages its own transcription system that is represented here by five RNA polymerase subunits, mRNA capping enzyme, poly(A) polymerase, and two transcription factors. These transcription proteins are also encoded and packaged by CroV [16, 74].

## Conclusions

The present work expands the known host range for members of the *Mimirividae* family to Rhizarian microalgae and confirms chlorarachniophytes as hosts for nucleocytoviruses of the subfamily *Aliimimivirinae*. An association of *B. natans* with viruses of the *Nucleocytoviricota* phylum had been predicted previously based on nucleocytovirus-like genes in the host nuclear genome [25]. Here, we establish a multi-isolate virus–host system for studying strain-level interactions and the effects of viral infections in *Bigelowiella* and a related genus.

The four ChlorV genomes share prominent features with CroV, including the early and late promoter motifs [16, 68] as well as many homologous proteins that are packaged into the capsid [74]. ChlorVs are predicted to encode four DJR and four SJR proteins, and their capsids may feature a unique vertex structure; however, more work is needed to compare the diverse minor capsid proteins with other high-resolution virion structures [73].

ChlorVs and CroV are lytic viruses that ultimately cause host cell death, although ChlorV-1 particle release in host strain AL-FL05 did not follow the typical burst dynamics observed for CroV in *Cafeteria burkhardae* [75]. Instead, free virions accumulated continuously from approximately 9 hpi to 36 hpi, whereas most host cells lysed later than 36 hpi, as observed by flow cytometry. Budding of viral particles from intact cells is thus a likely mechanism of ChlorV-1 release, although we cannot exclude other mechanisms.

Whereas we describe the infection cycle for ChlorV-1 in a single chlorarachniophyte strain (AL-FL05), more work is needed to investigate the full host range of different ChlorV strains and understand additional influencing factors such as diel cycles [76], nutrient availability, or temperature. The phylogenetic distance of ChlorV-1 compared to other ChlorV strains likely correlates with its ability to infect the distantly related chlorarachniophyte strain AL-FL05, but we cannot infer whether this is an adaptation of ChlorV to expand its host range or a specialization to *B. natans* in the other strains.

The function of ChlorV strain-specific methyltransferases remains to be elucidated. They could be involved in virulence, protecting viral DNA during infection while degrading the host genome by associated nucleases. A similar system has been described for chloroviruses of the order *Algavirales* [77, 78]. Given that closely related ChlorV strains differ in their repertoire of methyltransferases, it is also possible that these putative restriction-modification systems play a role in interviral competition, as proposed for a group of viruses infecting the microalga *Micromonas polaris* [79]. Alternatively, as chlorarachniophyte genomes contain endogenous virophages, the restriction-modification systems could suppress coinfecting virophages, which might otherwise have a negative impact on ChlorV replication [25, 80]. Modifying or disrupting viral methyltransferase genes in the chlorarachniophyte-ChlorV system could help to test these hypotheses for a better understanding of strain-specific host–virus interactions in a widespread marine microalga.

## Supplementary Material

Supplementary_Material_wrag093

## Data Availability

All sequence data, including raw Nanopore, PacBio, and Illumina reads, assemblies, and host rRNA genes (OZ264022–OZ264049) have been deposited in the European Nucleotide Archive under project PRJEB90372. Further data such as multiple sequence alignments, phylogenetic trees, AlphaFold 3 models, and other relevant data have been deposited in Edmond (10.17617/3.CC4JL7). Custom code and scripts are available at https://codeberg.org/maxemil/chlorv-genomes and 10.5281/zenodo.18596002.
