# Supplementary material for: Strain-level diversity of giant viruses infecting chlorarachniophyte algae in the subtropical North Pacific
Source: ISME J. 2026 Apr 16;20(1):wrag093. doi: 10.1093/ismejo/wrag093 (PMC13196603; doi:10.1093/ismejo/wrag093)
Supplement: Supplementary_Material_wrag093 [file supplementary_material_wrag093.zip › ChlorV ISMEJ SI.pdf]

Supplementary Information for: Strain-level diversity of giant viruses infecting chlorarachniophyte algae in the subtropical North Pacific

Authors:
[1]*Max Emil Schön, [2]Christopher R. Schvarcz, [1]Silja V. Malkewitz, [1]Fanny C. Hinner, [3]Anna Koslová, [1]Ulrike Mersdorf, [1]Fiona Schimm, [1]Sebastian Rickert, [4]Nadiia Pozhydaieva, [2]Kelsey McBeain, [5]Thomas Hackl, [1]Alina Cosima Schneider, [1]Karina Barenhoff, [4,6,7]Katharina Höfer, [2]Kyle F. Edwards, [2]Grieg F. Steward, [1‡]*Matthias G. Fischer

[1]Max Planck Institute for Medical Research, Heidelberg, Germany
[2]University of Hawai‘i at Mānoa, Honolulu, HI, USA
[3]Institute of Molecular Genetics, Czech Academy of Sciences, Prague, Czech Republic
[4]Max Planck Institute for Terrestrial Microbiology, Marburg, Germany
[5]Groningen University, Groningen, The Netherlands
[6]Department of Pharmacy, Institute of Pharmaceutical Biology and Biotechnology, Philipps Universität Marburg, Marburg, Germany
[7]Center for Synthetic Microbiology (SYNMIKRO), Philipps Universität Marburg, Marburg, Germany
[‡]Current affiliation: Max Planck Institute for Marine Microbiology, Bremen, Germany

*Corresponding authors: mschoen@mr.mpg.de; mfischer@mpi-bremen.de

Supplementary Figures:

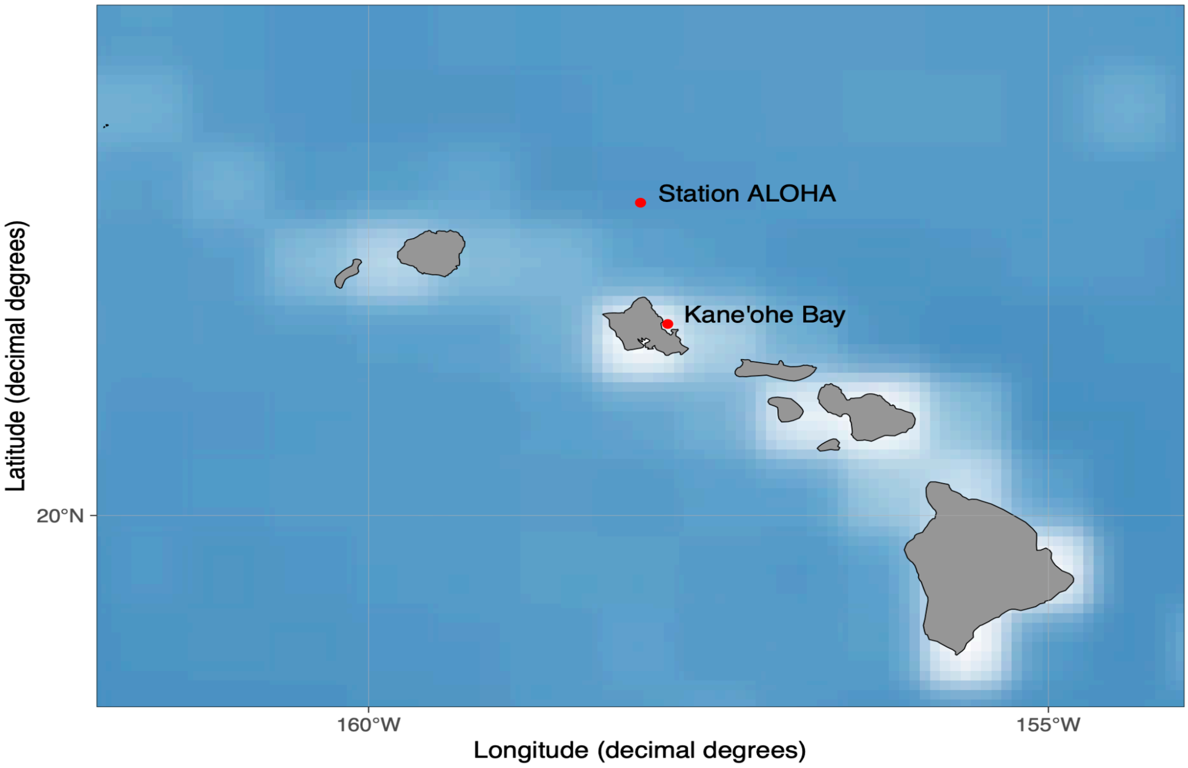

Figure S1: Location of the open-ocean collection point Station ALOHA approximately 100 km north of O‘ahu.

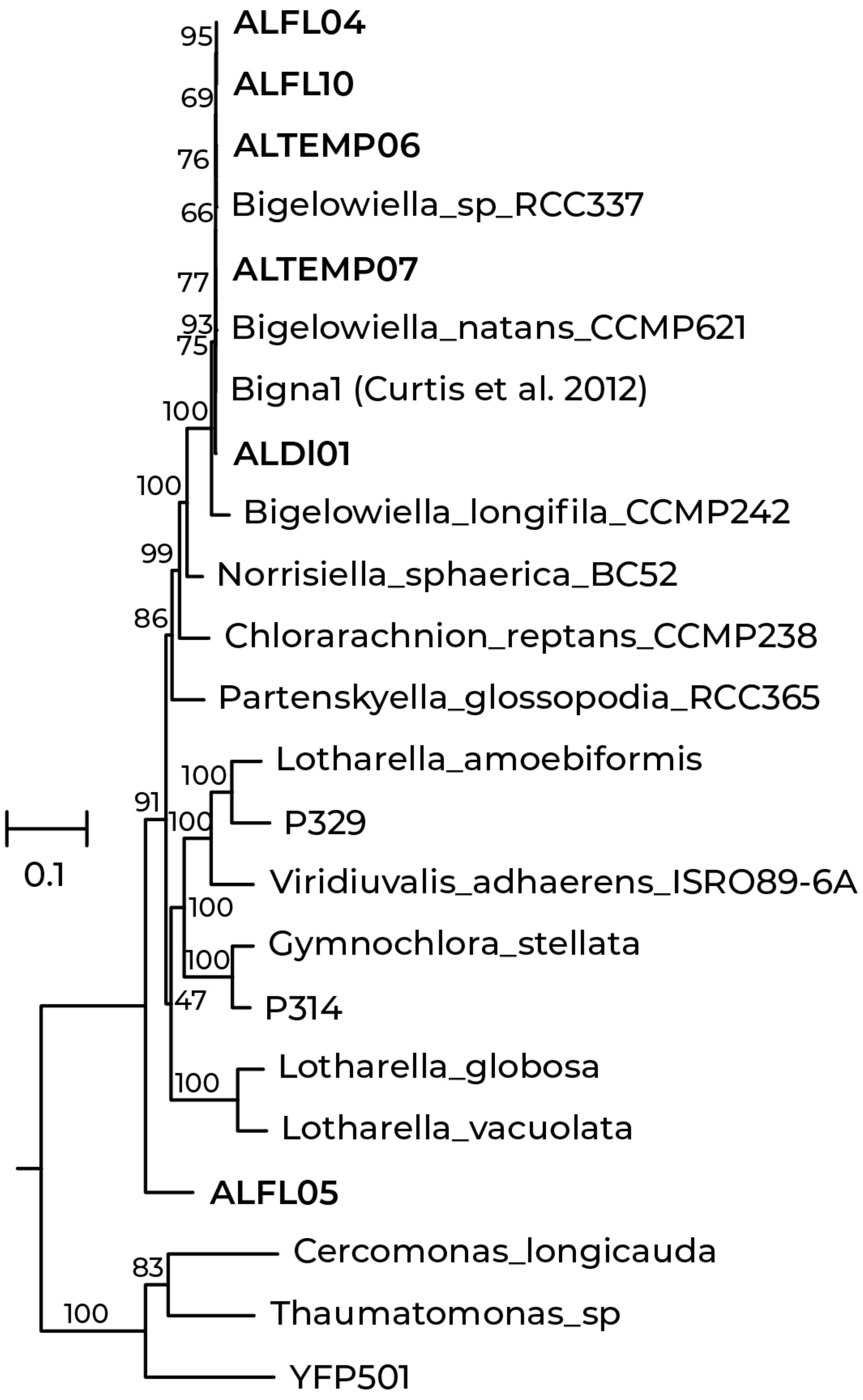

Figure S2: Phylogenetic tree of chlorarachniophyte host strains (bold) and reference species of chlorarachniophytes and an outgroup of non-photosynthetic rhizarians. 18S and 28S rRNA gene sequence alignments were concatenated and used to reconstruct a phylogenetic

tree under the TIM3+F+R3 model (selected by automatic model selection) in IQ-TREE with 1000 ultrafast bootstraps.

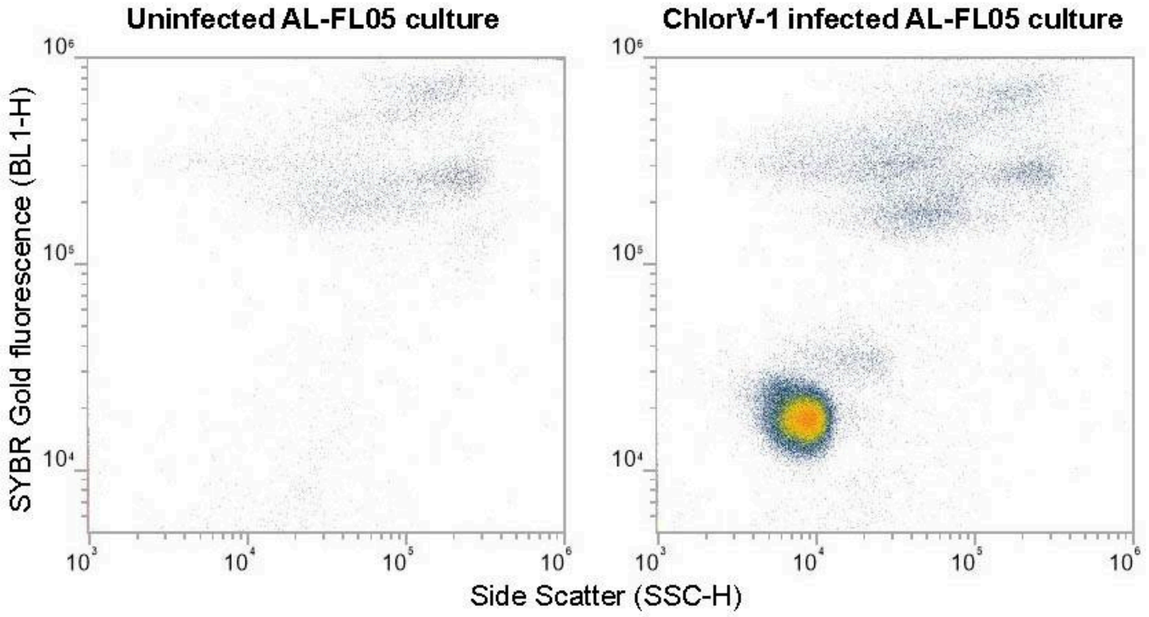

Figure S3: Flow cytometric detection of ChlorV-1 particles. Viral particles are clearly detectable by their fluorescence & side scatter signature after glutaraldehyde fixation and staining with SYBR Gold on an Attune NxT™ flow cytometer equipped with a small particle SSC filter (488/10). The viral population is the circular cloud of dots with the yellow/red center; diffuse populations of dots at the top represent bacteria that are present in the cultures.

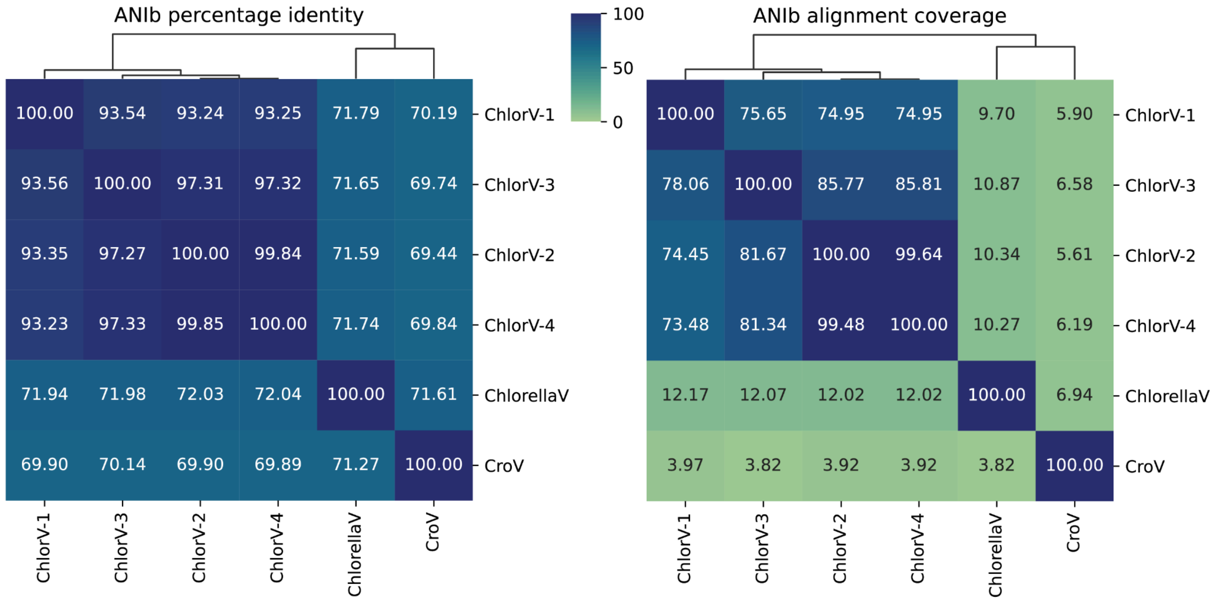

Figure S4: Average nucleotide identity (ANI, left) and pairwise alignment coverage (right) of ChlorVs and isolated virus genomes from the subfamily *Aliimimivirinae*.

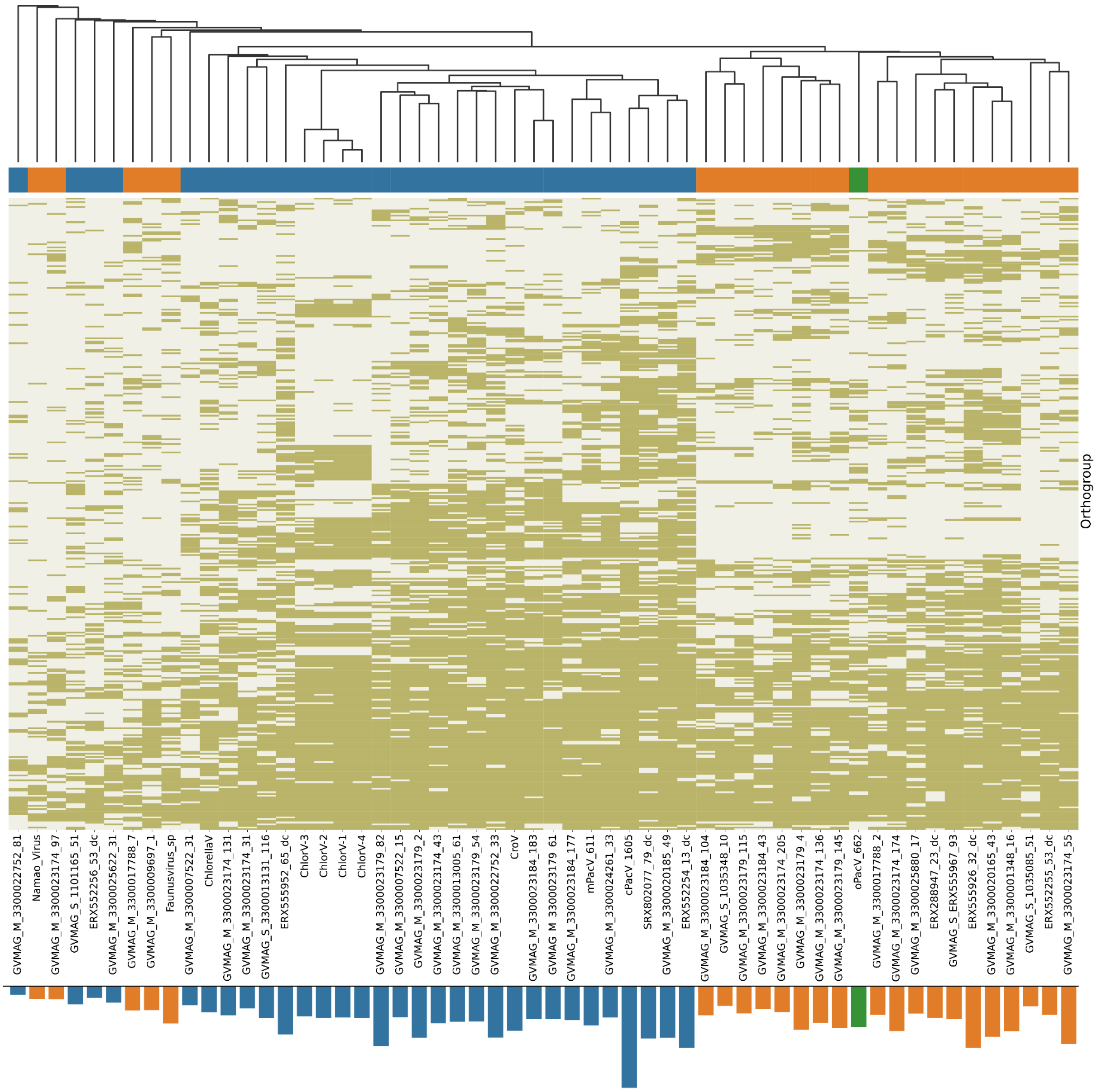

Figure S5: Gene sharing network and genome sizes of aliimimivirins. All predicted proteins of isolate and cultivation-independently acquired genomes of viruses from the subfamily *Aliimimivirinae* were subjected to orthogroup prediction. The orthogroups were then used to cluster the genomes. Genomes in the cluster *Aliimimivirinae* I are labelled blue, whereas members of *Aliimimivirinae* II are labelled orange. The genome oPacV_662 was not confidently placed in either of these groups and thus labelled green.

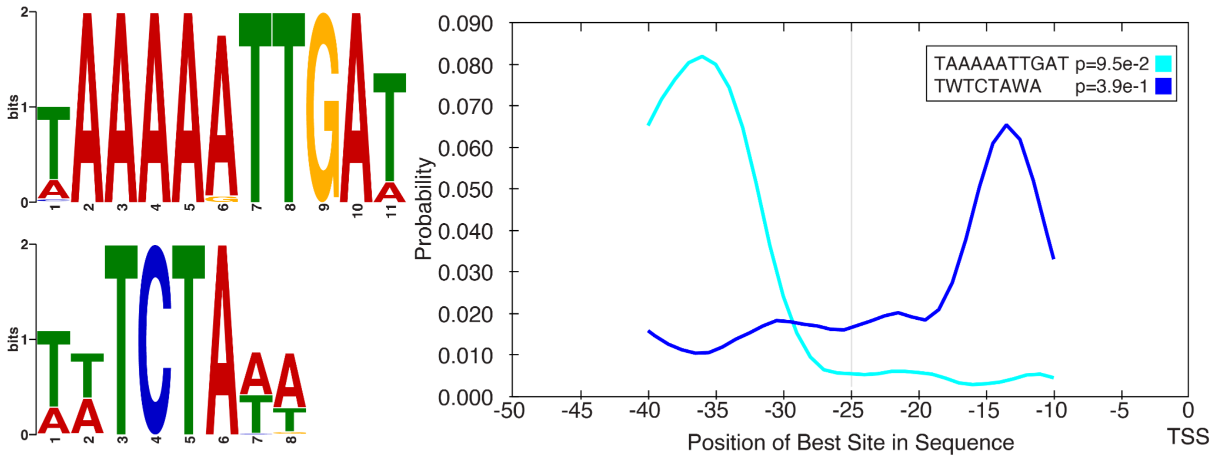

Figure S6: Predicted promoter motifs in ChlorV-1. Two significant motifs were found, likely representing an early (AAAAATTGA) and a late (TCTA) promoter consensus sequence, similar to the related Cafeteria roenbergensis virus. The position relative to the transcription start site (TSS) differed between these two motifs, with AAAAATTGA showing a peak around -34 and TCTA around -14.

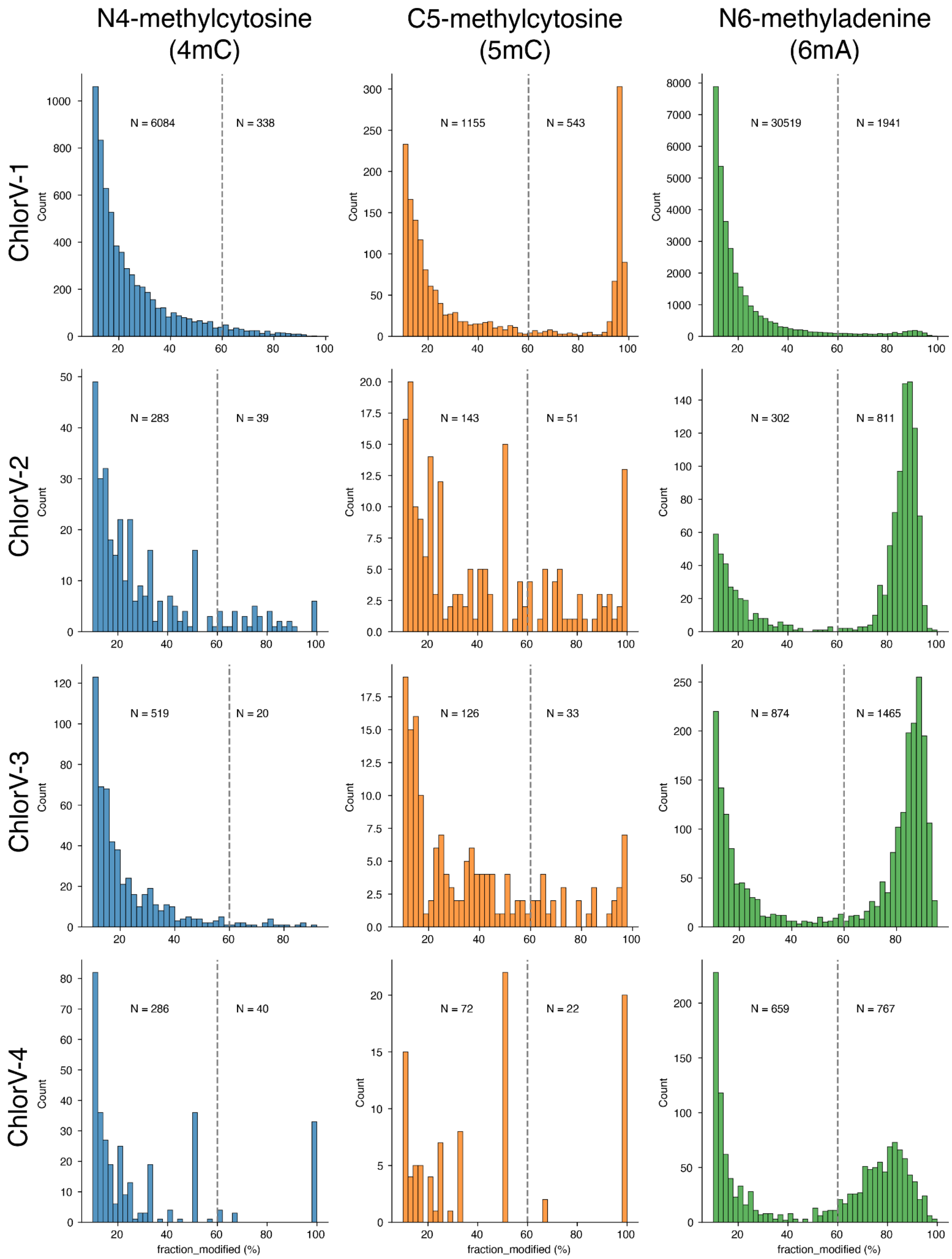

Figure S7: Modification frequency in ChlorV genomes for N4-methylcytosine (4mC), C5-methylcytosine (5mC) and N6-methyladenine (6mA). The “fraction modified” is the ratio of reads corroborating a specific modification per site and the total coverage. For example, at a site with a total of 1000 reads mapped, of which only 500 were predicted to be methylated at that site, the ”fraction modified” for the site would be 50%. The histograms represent all sites with at least 10% methylation frequency for the respective type of

modification. For ChlorV strains 2, 3 and 4, slightly different models (dna_r10.4.1_e8.2_400bps_sup@v5.0.0_4mC_5mC@v3 for 4mC and 5mC methylations and dna_r10.4.1_e8.2_400bps_sup@v5.0.0_6mA@v3 for 6mA) were used to basecall the data than for ChlorV-1 (rerio models res_dna_r10.4.1_e8.2_400bps_sup@v4.0.1_6mA@v2 and res_dna_r10.4.1_e8.2_400bps_sup@v4.3.0_4mC_5mC@v1) due to delays between sequencing runs. Depth of coverage for the ChlorV-1 assembly was 2328, whereas ChlorV-2 had an average depth of 434, ChlorV-3 983 and ChlorV-4 81.

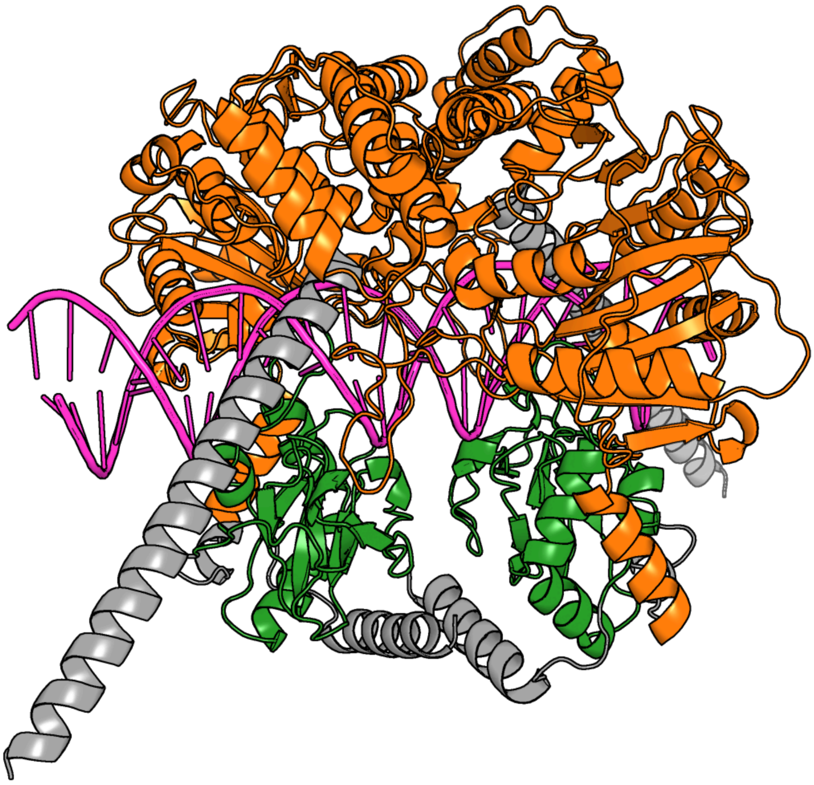

Figure S8: AlphaFold 3 protein structure prediction of a dimer of ChlorV-1..374 in complex with the DNA sequence ATTAACAT**CAATTGTATG**AAAATT. Protein domains are colored as in Figure 4. pTM=0.85, ipTM=0.8.

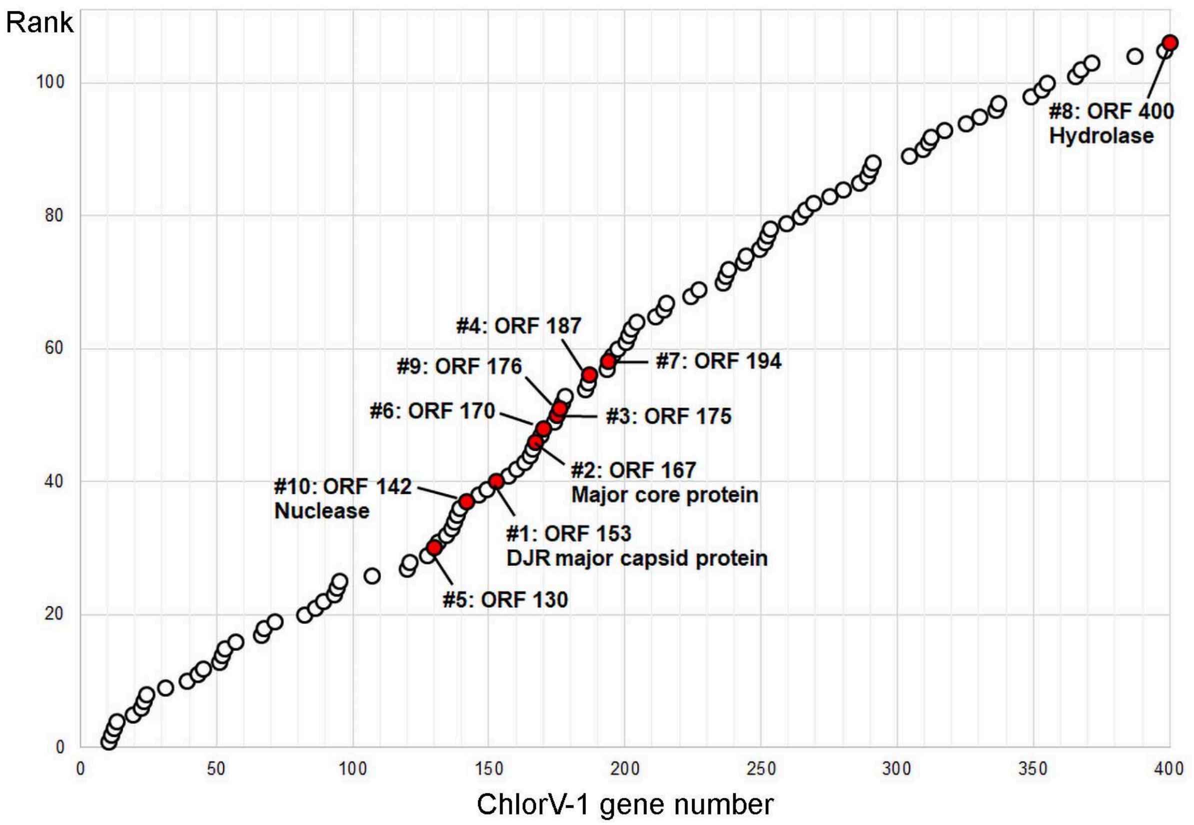

Figure S9: Genome position plot of ChlorV-1 virion proteins. Genes encoding proteins that were detected in purified virions by mass spectrometry are shown as circles in ascending order of their respective gene number (“‘Rank”). The 10 most abundant virion proteins are marked in red and labeled with their gene number.

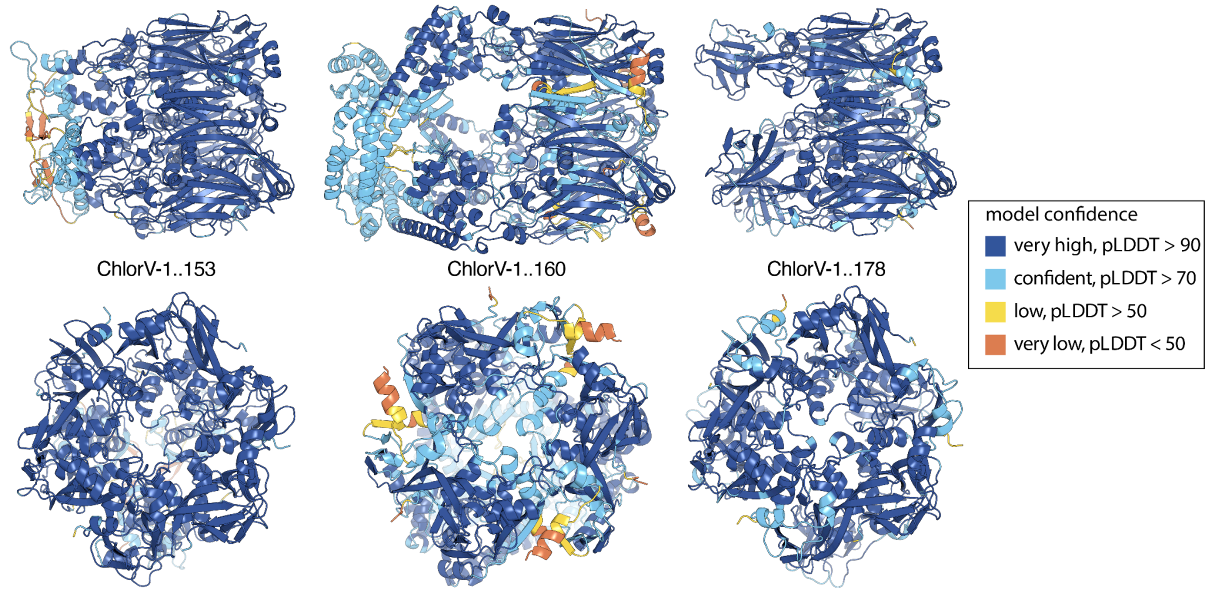

Figure S10: Trimeric structure predictions of the putative ChlorV-1 DJR capsid proteins 153, 160, and 178 with AlphaFold 3. All proteins are experimentally verified virion components Upper row: side view; lower row: top view.

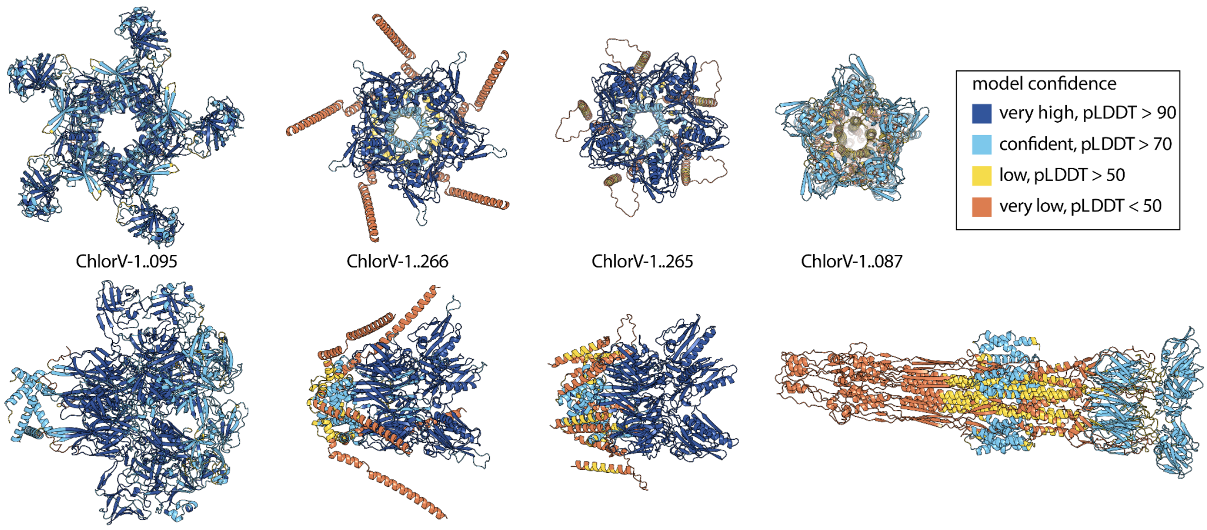

Figure S11: Pentameric structure predictions of the putative ChlorV-1 penton proteins 087, 095, 265 and 266 with AlphaFold 3. ChlorV-1..087 and ChlorV-1..266 are experimentally verified virion proteins. Upper row: top view; lower row: side view.

Supplementary Tables:

Table S1: Isolation times and location of hosts and viruses. This table summarizes collection metadata for chlorarachniophyte host strains and their associated viruses. The upper section lists host strains with strain ID, collection date and location, water collection depth (m), and collection number (UHM codes). The lower section lists virus strains (ChlorV-1–4) with collection date and location, water collection depth (m), and the algal host used for isolation.

Table S2: Sequencing read accessions and basecalling models. This table lists sequencing runs across Oxford Nanopore (ONT), PacBio, and Illumina platforms. The ENA/NCBI accession is given when available, target virus (ChlorV-1–4), run purpose (primary/modifications/auxiliary), run date, dorado (or bonito) version, flow cell type (e.g., FLO-MIN106/114), and the exact basecalling model. For non-ONT entries, the sequencing instrument is given in the “model” column.

Table S3: Chlorarachniophyte hosts 18S and 28S rRNA gene accessions. This table compiles 18S (SSU) and 28S (LSU) rRNA gene accession numbers for cultured chlorarachniophyte hosts and selected cercozoan relatives, with sequencing metadata where applicable. Accessions point to INSDC repositories (e.g., NCBI GenBank). Rows without flow cell/kit/basecaller/model indicate accessions from previous studies. Sequences generated here were sequenced using Oxford Nanopore, with the flow cell (FLO-MIN106/-112/-114), library kit (SQK-LSK110/-112/-114), basecaller (bonito or dorado, with version), and the specific basecalling model reported.

Table S4: ChlorV reproduction in different chlorarachniophyte strains. This table summarizes the virus-host strain combinations that led to detectable concentrations of newly produced extracellular virions. Chlorarachniophyte strain names are listed in the top row, virus strain names are listed in the leftmost column. Combinations that had led to productive infections are shaded in blue, combinations where neither virus production nor host lysis was detected are shaded in red. Numbers represent typical concentrations of ChlorV-like particles as detected by flow cytometry in particles per millilitre. Bold numbers indicate the strain

combination that led to the initial isolation of the respective virus strain. The *B. natans* strain AL-FL04, on which ChlorV-2 was isolated, is not part of this study.

Table S5: Sequence-based annotation for all predicted proteins from all four ChlorV strains. This table compiles sequence-based annotations for all predicted ORFs across ChlorV-1 to ChlorV-4. Each row is a hit from one database/tool for a given ORF (seqid). Reported databases include GVOG/NCVOG (giant virus orthologous groups), NCBI NR/Taxonomy (lineage/taxid), EggNOG (orthologous groups) and domain/structure databases (Interproscan). Columns include accession and description: identifiers and brief functional or taxonomic notes from the source database. tag: gene names from EggNOG, if available. start/stop: amino-acid coordinates of the matched region on the query protein. evalue: significance as reported by the tool; "-" indicates methods without E-values (e.g., Coils).

Table S6: ChlorV gene clusters and sequence/structure-based selected annotations. This table links putative orthologous genes across the four ChlorV genomes and summarizes their functional inference. Columns are ChlorV-1 to ChlorV-4: locus IDs for each genome; blanks indicate no detectable ortholog; multiple IDs in a cell denote paralogs or split/tandem genes. annot_sequence: function inferred from sequence homology/domain searches (Table S5). annot_structure: function inferred from structural similarity (Supplementary File 2). annot_consensus: reconciled call between sequence- and structure-based evidence. category: broad functional class (e.g., transferase, replication, translation, hypothetical). Where evidence is insufficient, "hypothetical protein" is given as the functional annotation.

Table S7: Homologous gene sequences between ChlorVs and the genome of *B natans*. Protein-level homology matches based on MMseqs2 between *Bigelowiella natans* gene models (query) and predicted ChlorV proteins (target). Each row is a query–target alignment with quality metrics and optional functional notes. identity [%], fident: percent identity and fractional identity (0–1). alnlen, mismatch, gapopen: alignment length, mismatch and gap counts. qstart/qend, tstart/tend: alignment coordinates on query and target. e-value, bitscore: statistical significance and alignment score. annotation: putative function of the ChlorV ORF, when available. NCLDV: *B. natans* gene models identified as NCLDV-like by Blanc et al. 2015.

Table S8: Summary of ipTM and pTM values for all structural predictions of dimeric or monomeric methyltransferases and different DNA sequences. This table reports confidence metrics for structural models of ChlorV methyltransferase candidates evaluated with multiple DNA sequences in two states (monomer vs homodimer). For each protein–DNA pair, predicted inter-chain interface confidence (ipTM) and overall fold confidence (pTM) are listed on a 0–1 scale. protein: ChlorV locus ID of the methyltransferase candidate. DNA: 24-nt test sequence (sense strand shown) used in the model. monomer ipTM/pTM: confidence for a single protein subunit with DNA; ipTM near zero reflects the absence of a protein–protein interface. dimer ipTM/pTM: confidence for a homodimer with DNA; higher ipTM indicates a more confident dimer interface, while pTM reflects overall model quality.

Table S9: Experimentally detected proteins using mass spectrometry. This table lists ChlorV-1 proteins identified by LC–MS/MS. Each row represents a protein group with its quantitative support and functional note. Protein.Group / Protein.Ids: ChlorV locus ID of the leading protein and all proteins in the group (identical here). Global.PG.Q.Value:

protein-group q-value (FDR-adjusted confidence). NrOfPrecursorsMeasured: number of peptide precursors measured for this protein group. Quantity: label-free quantification intensity. annotation: functional assignment (e.g., major capsid protein, P4B core protein, kinases, nuclease, lipase).

Supplementary Files:
Supplementary File 1: Single gene trees for giant virus phylogenetic markers. Order of genes is A32 genome packaging ATPase, protein-primed DNA polymerase B, RNA polymerase large subunit, superfamily II helicase, transcription factor IIB, topoisomerase II, and poxvirus late transcription factor VLTF3. Predicted proteins were matched to GVOG references with DIAMOND (BLASTp), aligned per marker with MAFFT E-INS-i, and trimmed with trimAl (-gt 0.1). Taxon sampling includes all Mimiviridae with other Imitervirales from GVDB as outgroup. Trees were inferred with IQ-TREE 2 (UFBoot 1000; -m MFP; -mset LG). ChlorV sequences are highlighted with blue stars. *Aliimimivirinae* and *Mimiviridae* clusters are shaded in teal and grey, respectively.

Supplementary File 2: Protein structure predictions using AlphaFold 3 and structure-based annotation. Each structural model was queried against PDB using Foldseek (easy-search) and alignments were visualized in PyMOL side-by-side. AlphaFold 3 models are colored according to pLDDT values (>90: very high confidence–dark blue; 70–90: confident–light blue; 50–70: low confidence–yellow;<50: very low confidence–orange). For each comparison that yielded a significant match, the top three PDB matches are given.

Datashare (https://doi.org/10.17617/3.CC4JL7): Trees and alignments, AlphaFold 3 models, tabular methylation calls, defense finder result files.

Code: https://codeberg.org/maxemil/chlorv-genomes and https://doi.org/10.5281/zenodo.18596002
