# Supplementary material for: Strain-level diversity of giant viruses infecting chlorarachniophyte algae in the subtropical North Pacific
Source: ISME J. 2026 Apr 16;20(1):wrag093. doi: 10.1093/ismejo/wrag093 (PMC13196603; doi:10.1093/ismejo/wrag093)

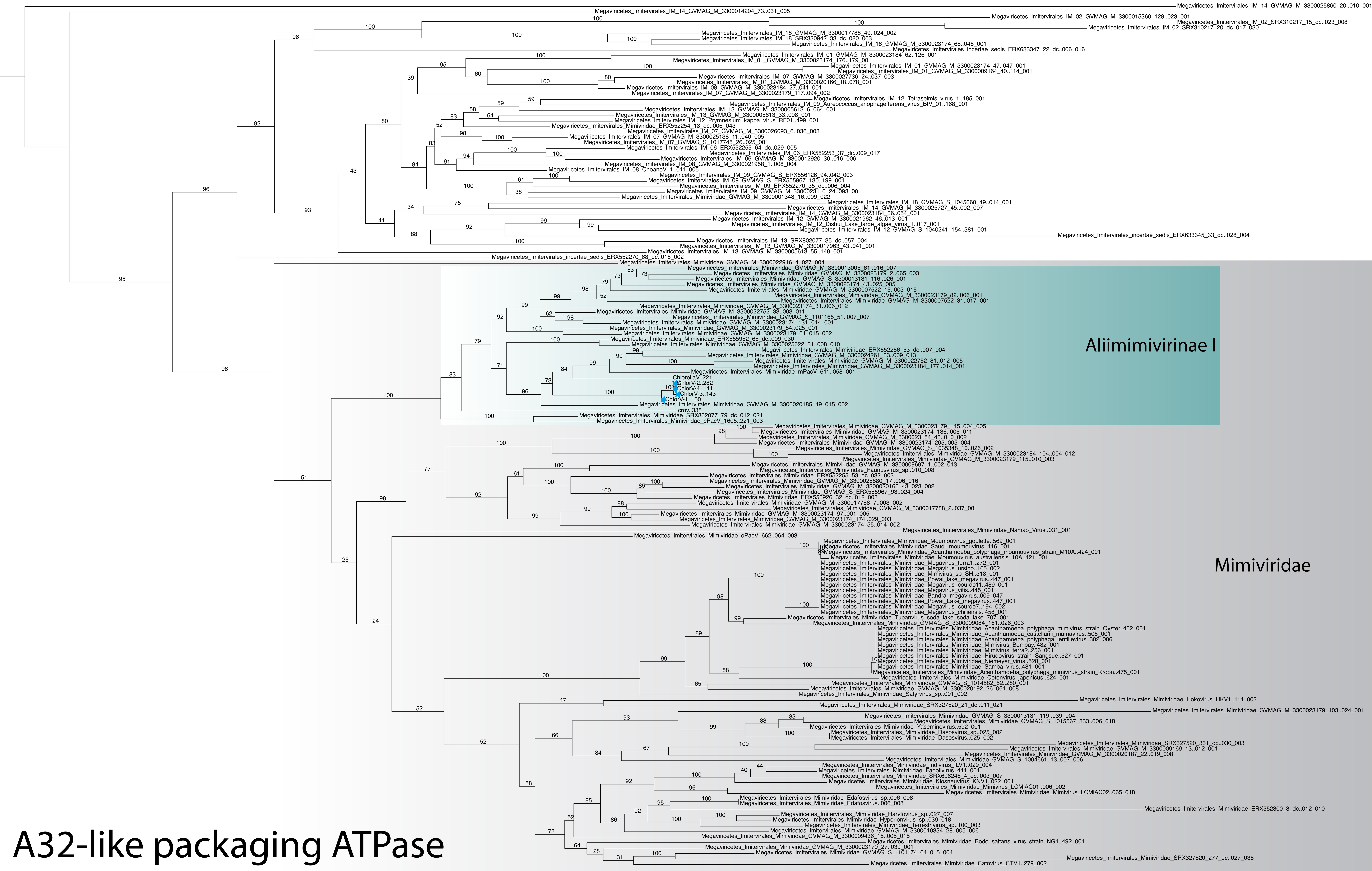
Aliimimivirinae I
Mimiviridae
A32-like packaging ATPase

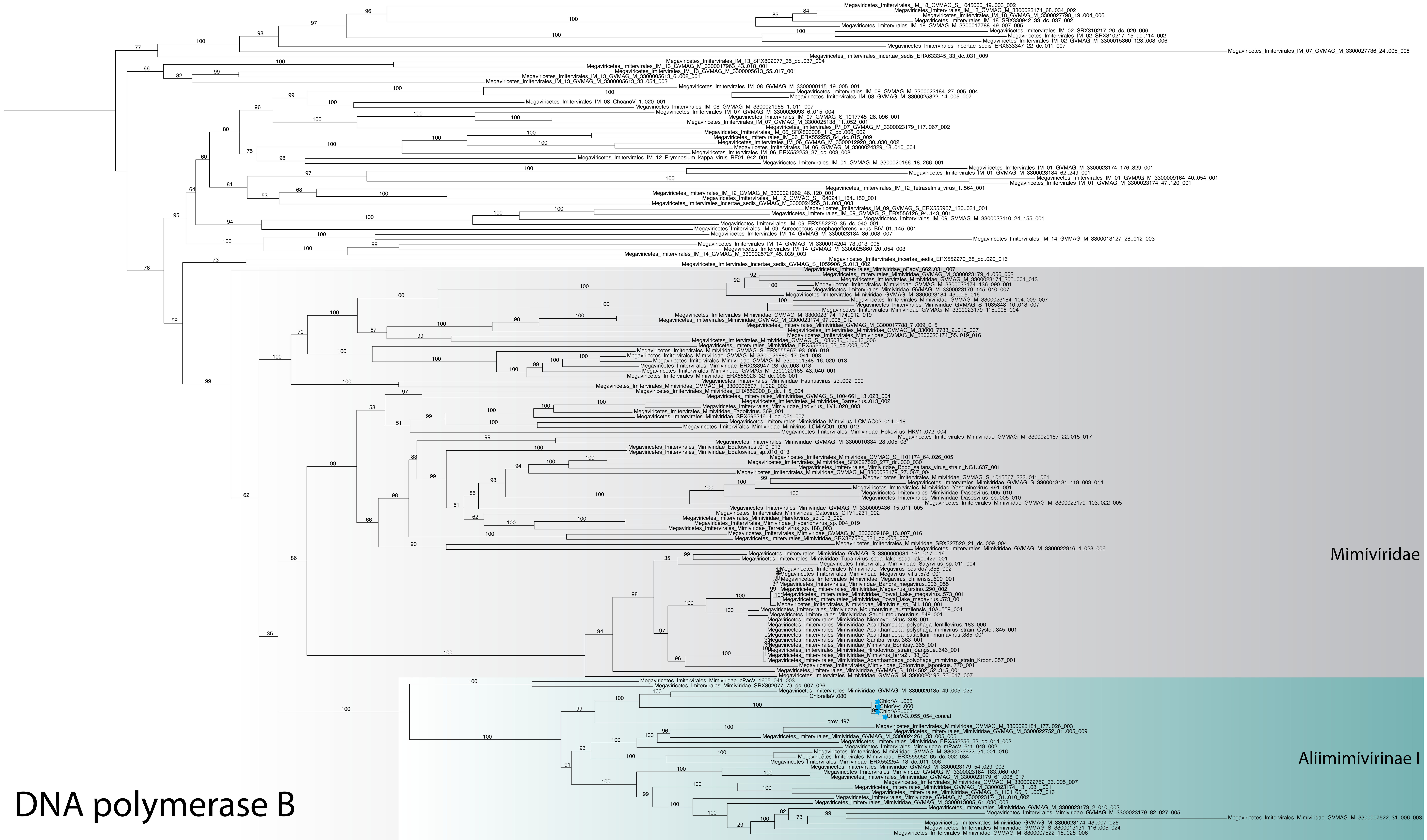
Mimiviridae
Aliimimivirinae I
DNA polymerase B

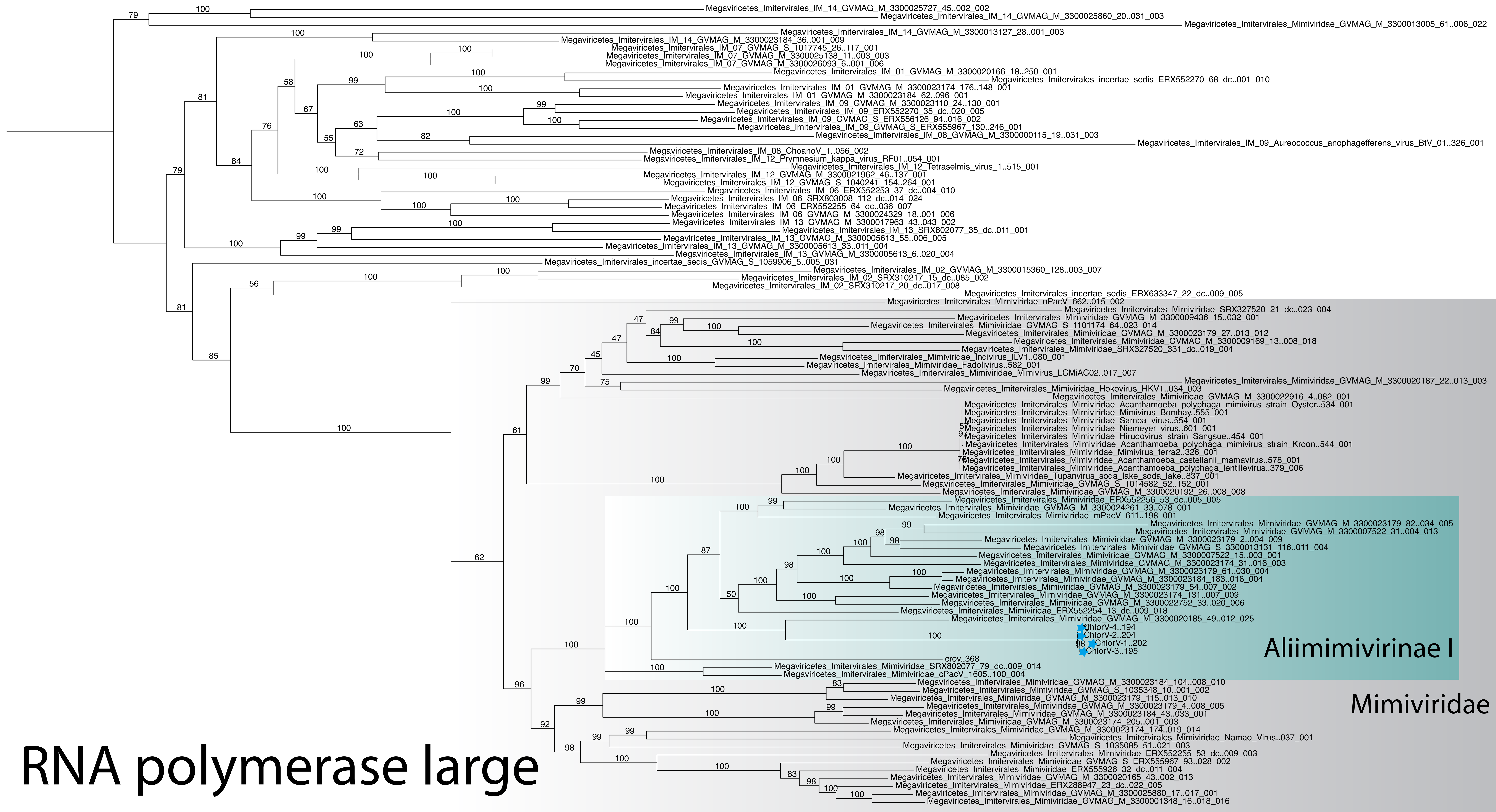

Megaviricetes_Imitervirales_IM_14_GVMAG_M_3300025727_45..002_002
Megaviricetes_Imitervirales_IM_14_GVMAG_M_3300025860_20..031_003
Megaviricetes_Imitervirales_Mimiviridae_GVMAG_M_3300013005_61..006_022
Megaviricetes_Imitervirales_IM_14_GVMAG_M_3300013127_28..001_003
Megaviricetes_Imitervirales_IM_14_GVMAG_M_3300023184_36..001_009
Megaviricetes_Imitervirales_IM_07_GVMAG_S_1017745_26..117_001
Megaviricetes_Imitervirales_IM_07_GVMAG_M_3300025138_11..003_003
Megaviricetes_Imitervirales_IM_07_GVMAG_M_3300026093_6..001_006
Megaviricetes_Imitervirales_IM_01_GVMAG_M_3300020166_18..250_001
Megaviricetes_Imitervirales_incertae_sedis_ERX552270_68_dc..001_010
Megaviricetes_Imitervirales_IM_01_GVMAG_M_3300023174_176..148_001
Megaviricetes_Imitervirales_IM_01_GVMAG_M_3300023184_62..096_001
Megaviricetes_Imitervirales_IM_09_GVMAG_M_3300023110_24..130_001
Megaviricetes_Imitervirales_IM_09_ERX552270_35_dc..020_005
Megaviricetes_Imitervirales_IM_09_GVMAG_S_ERX556126_94..016_002
Megaviricetes_Imitervirales_IM_09_GVMAG_S_ERX555967_130..246_001
Megaviricetes_Imitervirales_IM_08_GVMAG_M_3300000115_19..031_003
Megaviricetes_Imitervirales_IM_09_Aureococcus_anophagefferens_virus_BtV_01..326_001
Megaviricetes_Imitervirales_IM_08_ChoanoV_1..056_002
Megaviricetes_Imitervirales_IM_12_Prymnesium_kappa_virus_RF01..054_001
Megaviricetes_Imitervirales_IM_12_Tetraselmis_virus_1..515_001
Megaviricetes_Imitervirales_IM_12_GVMAG_M_3300021962_46..137_001
Megaviricetes_Imitervirales_IM_12_GVMAG_S_1040241_154..264_001
Megaviricetes_Imitervirales_IM_06_ERX552253_37_dc..004_010
Megaviricetes_Imitervirales_IM_06_SRX803008_112_dc..014_024
Megaviricetes_Imitervirales_IM_06_ERX552255_64_dc..036_007
Megaviricetes_Imitervirales_IM_06_GVMAG_M_3300024329_18..001_006
Megaviricetes_Imitervirales_IM_13_GVMAG_M_3300017963_43..043_002
Megaviricetes_Imitervirales_IM_13_SRX802077_35_dc..011_001
Megaviricetes_Imitervirales_IM_13_GVMAG_M_3300005613_55..006_005
Megaviricetes_Imitervirales_IM_13_GVMAG_M_3300005613_33..011_004
Megaviricetes_Imitervirales_IM_13_GVMAG_M_3300005613_6..020_004
Megaviricetes_Imitervirales_incertae_sedis_GVMAG_S_1059906_5..005_031
Megaviricetes_Imitervirales_IM_02_GVMAG_M_3300015360_128..003_007
Megaviricetes_Imitervirales_IM_02_SRX310217_15_dc..085_002
Megaviricetes_Imitervirales_IM_02_SRX310217_20_dc..017_008
Megaviricetes_Imitervirales_incertae_sedis_ERX633347_22_dc..009_005
Megaviricetes_Imitervirales_Mimiviridae_oPacV_662..015_002
Megaviricetes_Imitervirales_Mimiviridae_SRX327520_21_dc..023_004
Megaviricetes_Imitervirales_Mimiviridae_GVMAG_M_3300009436_15..032_001
Megaviricetes_Imitervirales_Mimiviridae_GVMAG_S_1101174_64..023_014
Megaviricetes_Imitervirales_Mimiviridae_GVMAG_M_3300023179_27..013_012
Megaviricetes_Imitervirales_Mimiviridae_GVMAG_M_3300009169_13..008_018
Megaviricetes_Imitervirales_Mimiviridae_SRX327520_331_dc..019_004
Megaviricetes_Imitervirales_Mimiviridae_Indivirus_ILV1..080_001
Megaviricetes_Imitervirales_Mimiviridae_Fadolivirus..582_001
Megaviricetes_Imitervirales_Mimiviridae_Mimivirus_LCMiAC02..017_007
Megaviricetes_Imitervirales_Mimiviridae_GVMAG_M_3300020187_22..013_003
Megaviricetes_Imitervirales_Mimiviridae_Hokovirus_HKV1..034_003
Megaviricetes_Imitervirales_Mimiviridae_GVMAG_M_3300022916_4..082_001
Megaviricetes_Imitervirales_Mimiviridae_Acanthamoeba_polyphaga_mimivirus_strain_Oyster..534_001
Megaviricetes_Imitervirales_Mimiviridae_Mimivirus_Bombay..555_001
Megaviricetes_Imitervirales_Mimiviridae_Samba_virus..554_001
Megaviricetes_Imitervirales_Mimiviridae_Niemeyer_virus..601_001
Megaviricetes_Imitervirales_Mimiviridae_Hirudovirus_strain_Sangsue..454_001
Megaviricetes_Imitervirales_Mimiviridae_Acanthamoeba_polyphaga_mimivirus_strain_Kroon..544_001
Megaviricetes_Imitervirales_Mimiviridae_Mimivirus_terra2..326_001
Megaviricetes_Imitervirales_Mimiviridae_Acanthamoeba_castellanii_mamavirus..578_001
Megaviricetes_Imitervirales_Mimiviridae_Acanthamoeba_polyphaga_lentillevirus..379_006
Megaviricetes_Imitervirales_Mimiviridae_Tupanvirus_soda_lake_soda_lake..837_001
Megaviricetes_Imitervirales_Mimiviridae_GVMAG_S_1014582_52..152_001
Megaviricetes_Imitervirales_Mimiviridae_GVMAG_M_3300020192_26..008_008
Megaviricetes_Imitervirales_Mimiviridae_ERX552256_53_dc..005_005
Megaviricetes_Imitervirales_Mimiviridae_GVMAG_M_3300024261_33..078_001
Megaviricetes_Imitervirales_Mimiviridae_mPacV_611..198_001
Megaviricetes_Imitervirales_Mimiviridae_GVMAG_M_3300023179_82..034_005
Megaviricetes_Imitervirales_Mimiviridae_GVMAG_M_3300007522_31..004_013
Megaviricetes_Imitervirales_Mimiviridae_GVMAG_M_3300023179_2..004_009
Megaviricetes_Imitervirales_Mimiviridae_GVMAG_S_3300013131_116..011_004
Megaviricetes_Imitervirales_Mimiviridae_GVMAG_M_3300007522_15..003_001
Megaviricetes_Imitervirales_Mimiviridae_GVMAG_M_3300023174_31..016_003
Megaviricetes_Imitervirales_Mimiviridae_GVMAG_M_3300023179_61..030_004
Megaviricetes_Imitervirales_Mimiviridae_GVMAG_M_3300023184_183..016_004
Megaviricetes_Imitervirales_Mimiviridae_GVMAG_M_3300023179_54..007_002
Megaviricetes_Imitervirales_Mimiviridae_GVMAG_M_3300023174_131..007_009
Megaviricetes_Imitervirales_Mimiviridae_GVMAG_M_3300022752_33..020_006
Megaviricetes_Imitervirales_Mimiviridae_ERX552254_13_dc..009_018
Megaviricetes_Imitervirales_Mimiviridae_GVMAG_M_3300020185_49..012_025
ChlorV-4..194
ChlorV-2..204
ChlorV-1..202
ChlorV-3..195
crov..368
Megaviricetes_Imitervirales_Mimiviridae_SRX802077_79_dc..009_014
Megaviricetes_Imitervirales_Mimiviridae_cPacV_1605..100_004
Aliimimivirinae I
Megaviricetes_Imitervirales_Mimiviridae_GVMAG_M_3300023184_104..008_010
Megaviricetes_Imitervirales_Mimiviridae_GVMAG_S_1035348_10..001_002
Megaviricetes_Imitervirales_Mimiviridae_GVMAG_M_3300023179_115..013_010
Megaviricetes_Imitervirales_Mimiviridae_GVMAG_M_3300023179_4..008_005
Megaviricetes_Imitervirales_Mimiviridae_GVMAG_M_3300023184_43..033_001
Megaviricetes_Imitervirales_Mimiviridae_GVMAG_M_3300023174_205..001_003
Megaviricetes_Imitervirales_Mimiviridae_GVMAG_M_3300023174_174..019_014
Megaviricetes_Imitervirales_Mimiviridae_Namao_Virus..037_001
Megaviricetes_Imitervirales_Mimiviridae_GVMAG_S_1035085_51..021_003
Megaviricetes_Imitervirales_Mimiviridae_ERX552255_53_dc..009_003
Megaviricetes_Imitervirales_Mimiviridae_GVMAG_S_ERX555967_93..028_002
Megaviricetes_Imitervirales_Mimiviridae_ERX555926_32_dc..011_004
Megaviricetes_Imitervirales_Mimiviridae_GVMAG_M_3300020165_43..002_013
Megaviricetes_Imitervirales_Mimiviridae_ERX288947_23_dc..022_005
Megaviricetes_Imitervirales_Mimiviridae_GVMAG_M_3300025880_17..017_001
Megaviricetes_Imitervirales_Mimiviridae_GVMAG_M_3300001348_16..018_016
Mimiviridae
RNA polymerase large

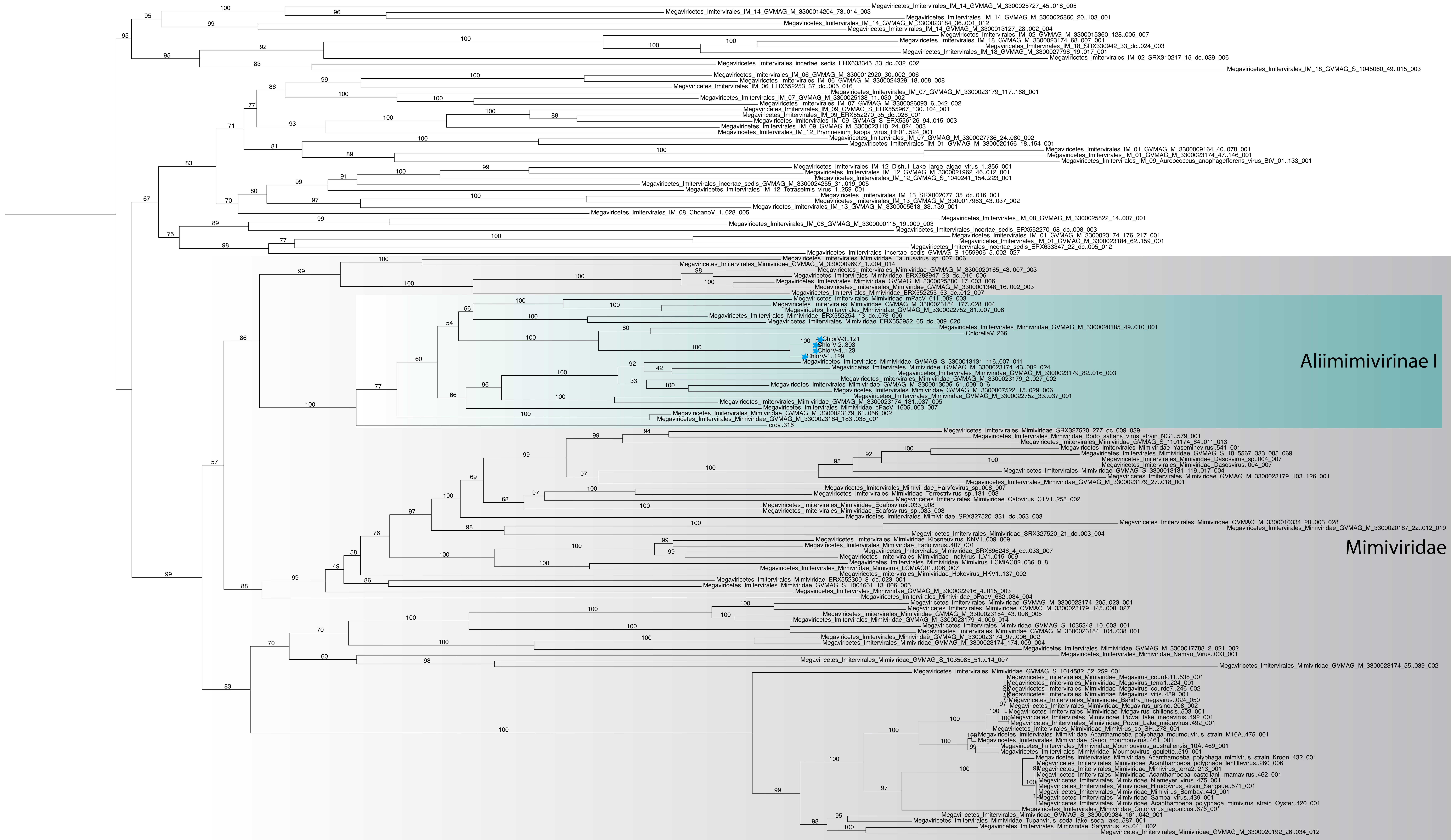
Aliimimivirinae I
Mimiviridae

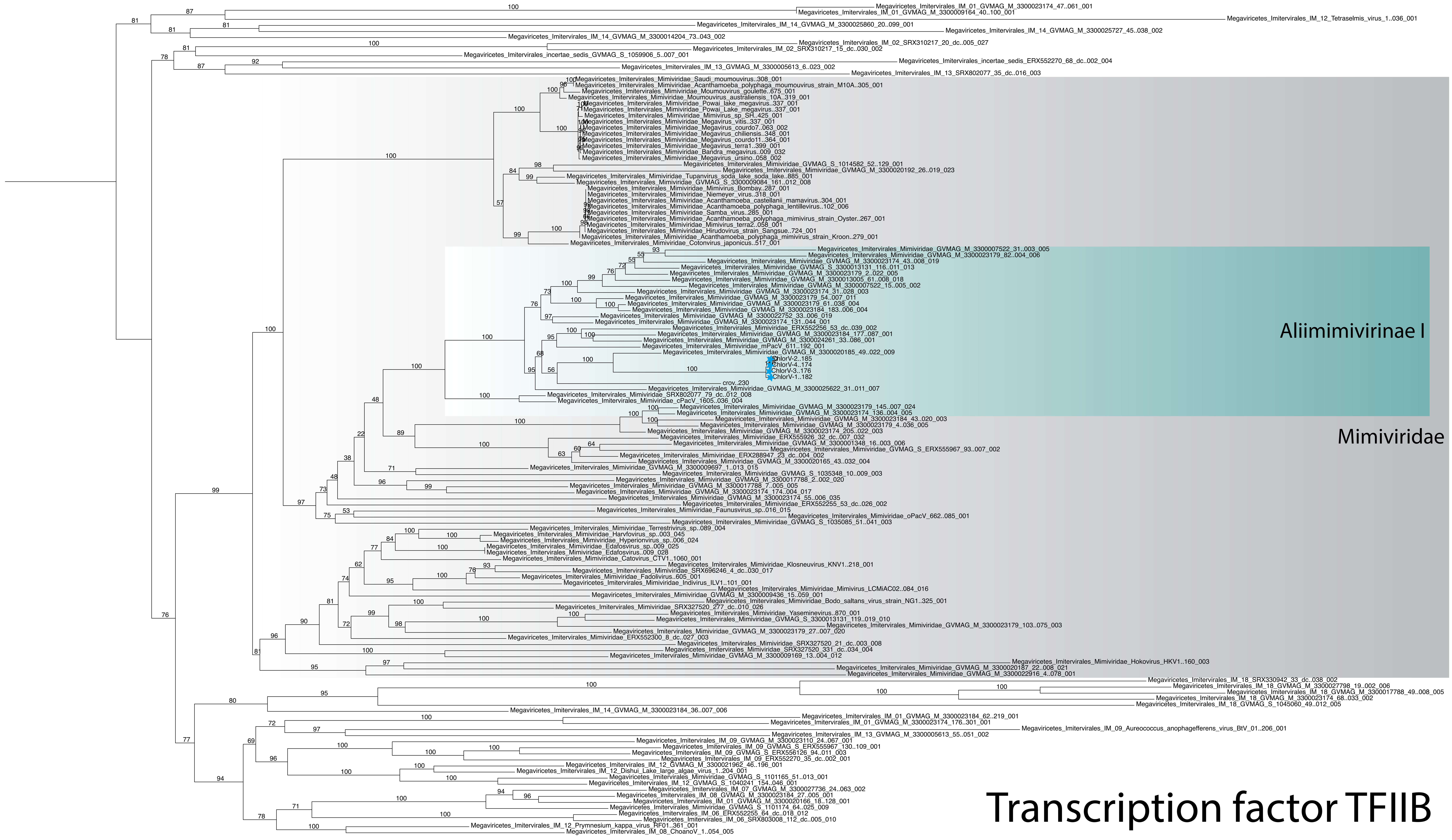
Aliimimivirinae I
Mimiviridae
Transcription factor TFIIB

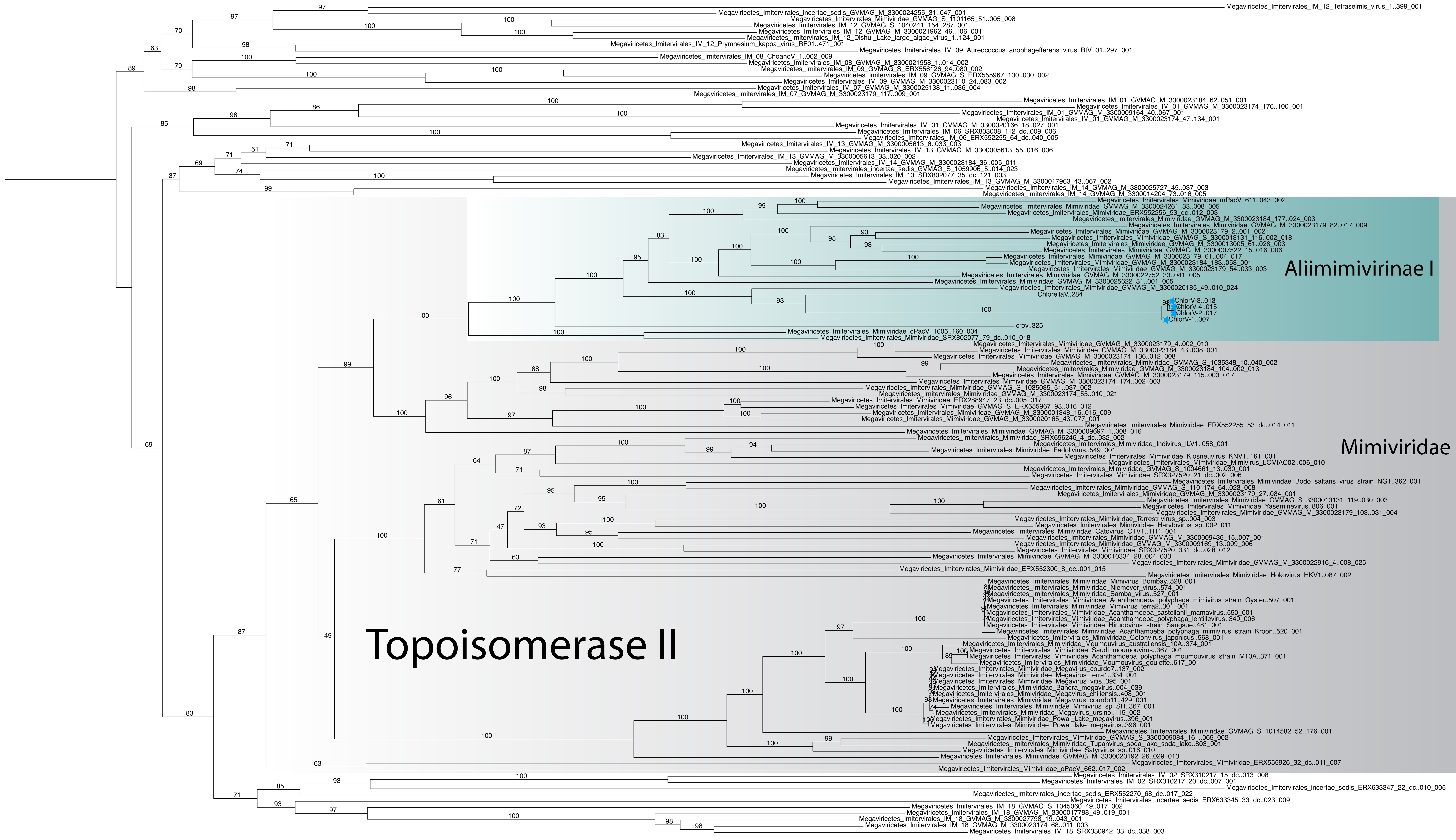
Megaviricetes_Imitervirales_IM_12_Tetraselmis_virus_1..399_001
Megaviricetes_Imitervirales_incertae_sedis_GVMAG_M_3300024255_31..047_001
Megaviricetes_Imitervirales_Mimiviridae_GVMAG_S_1101165_51..005_008
Megaviricetes_Imitervirales_IM_12_GVMAG_S_1040241_154..287_001
Megaviricetes_Imitervirales_IM_12_GVMAG_M_3300021962_46..106_001
Megaviricetes_Imitervirales_IM_12_Dishui_Lake_large_algae_virus_1..124_001
Megaviricetes_Imitervirales_IM_12_Prymnesium_kappa_virus_RF01..471_001
Megaviricetes_Imitervirales_IM_09_Aureococcus_anophagefferens_virus_BtV_01..297_001
Megaviricetes_Imitervirales_IM_08_ChoanoV_1..002_009
Megaviricetes_Imitervirales_IM_08_GVMAG_M_3300021958_1..014_002
Megaviricetes_Imitervirales_IM_09_GVMAG_S_ERX556126_94..080_002
Megaviricetes_Imitervirales_IM_09_GVMAG_S_ERX555967_130..030_002
Megaviricetes_Imitervirales_IM_09_GVMAG_M_3300023110_24..083_002
Megaviricetes_Imitervirales_IM_07_GVMAG_M_3300025138_11..036_004
Megaviricetes_Imitervirales_IM_07_GVMAG_M_3300023179_117..009_001
Megaviricetes_Imitervirales_IM_01_GVMAG_M_3300023184_62..051_001
Megaviricetes_Imitervirales_IM_01_GVMAG_M_3300023174_176..100_001
Megaviricetes_Imitervirales_IM_01_GVMAG_M_3300009164_40..067_001
Megaviricetes_Imitervirales_IM_01_GVMAG_M_3300023174_47..134_001
Megaviricetes_Imitervirales_IM_01_GVMAG_M_3300020166_18..027_001
Megaviricetes_Imitervirales_IM_06_SRX803008_112_dc..009_006
Megaviricetes_Imitervirales_IM_06_ERX552255_64_dc..040_005
Megaviricetes_Imitervirales_IM_13_GVMAG_M_3300005613_6..033_003
Megaviricetes_Imitervirales_IM_13_GVMAG_M_3300005613_55..016_006
Megaviricetes_Imitervirales_IM_13_GVMAG_M_3300005613_33..020_002
Megaviricetes_Imitervirales_IM_14_GVMAG_M_3300023184_36..005_011
Megaviricetes_Imitervirales_incertae_sedis_GVMAG_S_1059906_5..014_023
Megaviricetes_Imitervirales_IM_13_SRX802077_35_dc..121_003
Megaviricetes_Imitervirales_IM_13_GVMAG_M_3300017963_43..067_002
Megaviricetes_Imitervirales_IM_14_GVMAG_M_3300025727_45..037_003
Megaviricetes_Imitervirales_IM_14_GVMAG_M_3300014204_73..016_005
Megaviricetes_Imitervirales_Mimiviridae_mPacV_611..043_002
Megaviricetes_Imitervirales_Mimiviridae_GVMAG_M_3300024261_33..008_005
Megaviricetes_Imitervirales_Mimiviridae_ERX552256_53_dc..012_003
Megaviricetes_Imitervirales_Mimiviridae_GVMAG_M_3300023184_177..024_003
Megaviricetes_Imitervirales_Mimiviridae_GVMAG_M_3300023179_82..017_009
Megaviricetes_Imitervirales_Mimiviridae_GVMAG_M_3300023179_2..001_002
Megaviricetes_Imitervirales_Mimiviridae_GVMAG_S_3300013131_116..002_018
Megaviricetes_Imitervirales_Mimiviridae_GVMAG_M_3300013005_61..028_003
Megaviricetes_Imitervirales_Mimiviridae_GVMAG_M_3300007522_15..016_006
Megaviricetes_Imitervirales_Mimiviridae_GVMAG_M_3300023179_61..004_017
Megaviricetes_Imitervirales_Mimiviridae_GVMAG_M_3300023184_183..058_001
Megaviricetes_Imitervirales_Mimiviridae_GVMAG_M_3300023179_54..033_003
Megaviricetes_Imitervirales_Mimiviridae_GVMAG_M_3300022752_33..041_005
Megaviricetes_Imitervirales_Mimiviridae_GVMAG_M_3300025622_31..001_005
Megaviricetes_Imitervirales_Mimiviridae_GVMAG_M_3300020185_49..010_024
ChlorellaV..284
ChlorV-3..013
ChlorV-4..015
ChlorV-2..017
ChlorV-1..007
crov..325
Megaviricetes_Imitervirales_Mimiviridae_cPacV_1605..160_004
Megaviricetes_Imitervirales_Mimiviridae_SRX802077_79_dc..010_018
Aliimimivirinae I
Megaviricetes_Imitervirales_Mimiviridae_GVMAG_M_3300023179_4..002_010
Megaviricetes_Imitervirales_Mimiviridae_GVMAG_M_3300023184_43..008_001
Megaviricetes_Imitervirales_Mimiviridae_GVMAG_M_3300023174_136..012_008
Megaviricetes_Imitervirales_Mimiviridae_GVMAG_S_1035348_10..040_002
Megaviricetes_Imitervirales_Mimiviridae_GVMAG_M_3300023184_104..002_013
Megaviricetes_Imitervirales_Mimiviridae_GVMAG_M_3300023179_115..003_017
Megaviricetes_Imitervirales_Mimiviridae_GVMAG_M_3300023174_174..002_003
Megaviricetes_Imitervirales_Mimiviridae_GVMAG_S_1035085_51..037_002
Megaviricetes_Imitervirales_Mimiviridae_GVMAG_M_3300023174_55..010_021
Megaviricetes_Imitervirales_Mimiviridae_ERX288947_23_dc..005_017
Megaviricetes_Imitervirales_Mimiviridae_GVMAG_S_ERX555967_93..016_012
Megaviricetes_Imitervirales_Mimiviridae_GVMAG_M_3300001348_16..016_009
Megaviricetes_Imitervirales_Mimiviridae_GVMAG_M_3300020165_43..077_001
Megaviricetes_Imitervirales_Mimiviridae_ERX552255_53_dc..014_011
Megaviricetes_Imitervirales_Mimiviridae_GVMAG_M_3300009697_1..008_016
Megaviricetes_Imitervirales_Mimiviridae_SRX696246_4_dc..032_002
Megaviricetes_Imitervirales_Mimiviridae_Indivirus_ILV1..058_001
Megaviricetes_Imitervirales_Mimiviridae_Fadolivirus..549_001
Megaviricetes_Imitervirales_Mimiviridae_Klosneuvirus_KNV1..161_001
Megaviricetes_Imitervirales_Mimiviridae_Mimivirus_LCMiAC02..006_010
Megaviricetes_Imitervirales_Mimiviridae_GVMAG_S_1004661_13..030_001
Megaviricetes_Imitervirales_Mimiviridae_SRX327520_21_dc..002_006
Megaviricetes_Imitervirales_Mimiviridae_Bodo_saltans_virus_strain_NG1..362_001
Megaviricetes_Imitervirales_Mimiviridae_GVMAG_S_1101174_64..023_008
Megaviricetes_Imitervirales_Mimiviridae_GVMAG_M_3300023179_27..084_001
Megaviricetes_Imitervirales_Mimiviridae_GVMAG_S_3300013131_119..030_003
Megaviricetes_Imitervirales_Mimiviridae_Yaseminevirus..806_001
Megaviricetes_Imitervirales_Mimiviridae_GVMAG_M_3300023179_103..031_004
Megaviricetes_Imitervirales_Mimiviridae_Terrestrivirus_sp..004_003
Megaviricetes_Imitervirales_Mimiviridae_Harvfovirus_sp..002_011
Megaviricetes_Imitervirales_Mimiviridae_Catovirus_CTV1..1111_001
Megaviricetes_Imitervirales_Mimiviridae_GVMAG_M_3300009436_15..007_001
Megaviricetes_Imitervirales_Mimiviridae_GVMAG_M_3300009169_13..009_006
Megaviricetes_Imitervirales_Mimiviridae_SRX327520_331_dc..028_012
Megaviricetes_Imitervirales_Mimiviridae_GVMAG_M_3300010334_28..004_033
Megaviricetes_Imitervirales_Mimiviridae_GVMAG_M_3300022916_4..008_025
Megaviricetes_Imitervirales_Mimiviridae_ERX552300_8_dc..001_015
Megaviricetes_Imitervirales_Mimiviridae_Hokovirus_HKV1..087_002
Megaviricetes_Imitervirales_Mimiviridae_Mimivirus_Bombay..528_001
Megaviricetes_Imitervirales_Mimiviridae_Niemeyer_virus..574_001
Megaviricetes_Imitervirales_Mimiviridae_Samba_virus..527_001
Megaviricetes_Imitervirales_Mimiviridae_Acanthamoeba_polyphaga_mimivirus_strain_Oyster..507_001
Megaviricetes_Imitervirales_Mimiviridae_Mimivirus_terra2..301_001
Megaviricetes_Imitervirales_Mimiviridae_Acanthamoeba_castellanii_mamavirus..550_001
Megaviricetes_Imitervirales_Mimiviridae_Acanthamoeba_polyphaga_lentillevirus..349_006
Megaviricetes_Imitervirales_Mimiviridae_Hirudovirus_strain_Sangsue..481_001
Megaviricetes_Imitervirales_Mimiviridae_Acanthamoeba_polyphaga_mimivirus_strain_Kroon..520_001
Megaviricetes_Imitervirales_Mimiviridae_Cotonvirus_japonicus..568_001
Megaviricetes_Imitervirales_Mimiviridae_Moumouvirus_australiensis_10A..374_001
Megaviricetes_Imitervirales_Mimiviridae_Saudi_moumouvirus..367_001
Megaviricetes_Imitervirales_Mimiviridae_Acanthamoeba_polyphaga_moumouvirus_strain_M10A..371_001
Megaviricetes_Imitervirales_Mimiviridae_Moumouvirus_goulette..617_001
Megaviricetes_Imitervirales_Mimiviridae_Megavirus_courdo7..137_002
Megaviricetes_Imitervirales_Mimiviridae_Megavirus_terra1..334_001
Megaviricetes_Imitervirales_Mimiviridae_Megavirus_vitis..395_001
Megaviricetes_Imitervirales_Mimiviridae_Bandra_megavirus..004_039
Megaviricetes_Imitervirales_Mimiviridae_Megavirus_chiliensis..408_001
Megaviricetes_Imitervirales_Mimiviridae_Megavirus_courdo11..429_001
Megaviricetes_Imitervirales_Mimiviridae_Mimivirus_sp_SH..367_001
Megaviricetes_Imitervirales_Mimiviridae_Megavirus_ursino..115_002
Megaviricetes_Imitervirales_Mimiviridae_Powai_Lake_megavirus..396_001
Megaviricetes_Imitervirales_Mimiviridae_Powai_lake_megavirus..396_001
Megaviricetes_Imitervirales_Mimiviridae_GVMAG_S_1014582_52..176_001
Megaviricetes_Imitervirales_Mimiviridae_GVMAG_S_3300009084_161..065_002
Megaviricetes_Imitervirales_Mimiviridae_Tupanvirus_soda_lake_soda_lake..803_001
Megaviricetes_Imitervirales_Mimiviridae_Satyrvirus_sp..016_010
Megaviricetes_Imitervirales_Mimiviridae_GVMAG_M_3300020192_26..029_013
Megaviricetes_Imitervirales_Mimiviridae_ERX555926_32_dc..011_007
Megaviricetes_Imitervirales_Mimiviridae_oPacV_662..017_002
Mimiviridae
Topoisomerase II
Megaviricetes_Imitervirales_IM_02_SRX310217_15_dc..013_008
Megaviricetes_Imitervirales_IM_02_SRX310217_20_dc..007_001
Megaviricetes_Imitervirales_incertae_sedis_ERX633347_22_dc..010_005
Megaviricetes_Imitervirales_incertae_sedis_ERX552270_68_dc..017_022
Megaviricetes_Imitervirales_incertae_sedis_ERX633345_33_dc..023_009
Megaviricetes_Imitervirales_IM_18_GVMAG_S_1045060_49..017_002
Megaviricetes_Imitervirales_IM_18_GVMAG_M_3300017788_49..019_001
Megaviricetes_Imitervirales_IM_18_GVMAG_M_3300027798_19..043_001
Megaviricetes_Imitervirales_IM_18_GVMAG_M_3300023174_68..011_003
Megaviricetes_Imitervirales_IM_18_SRX330942_33_dc..038_003

Virus late transcription factor 3

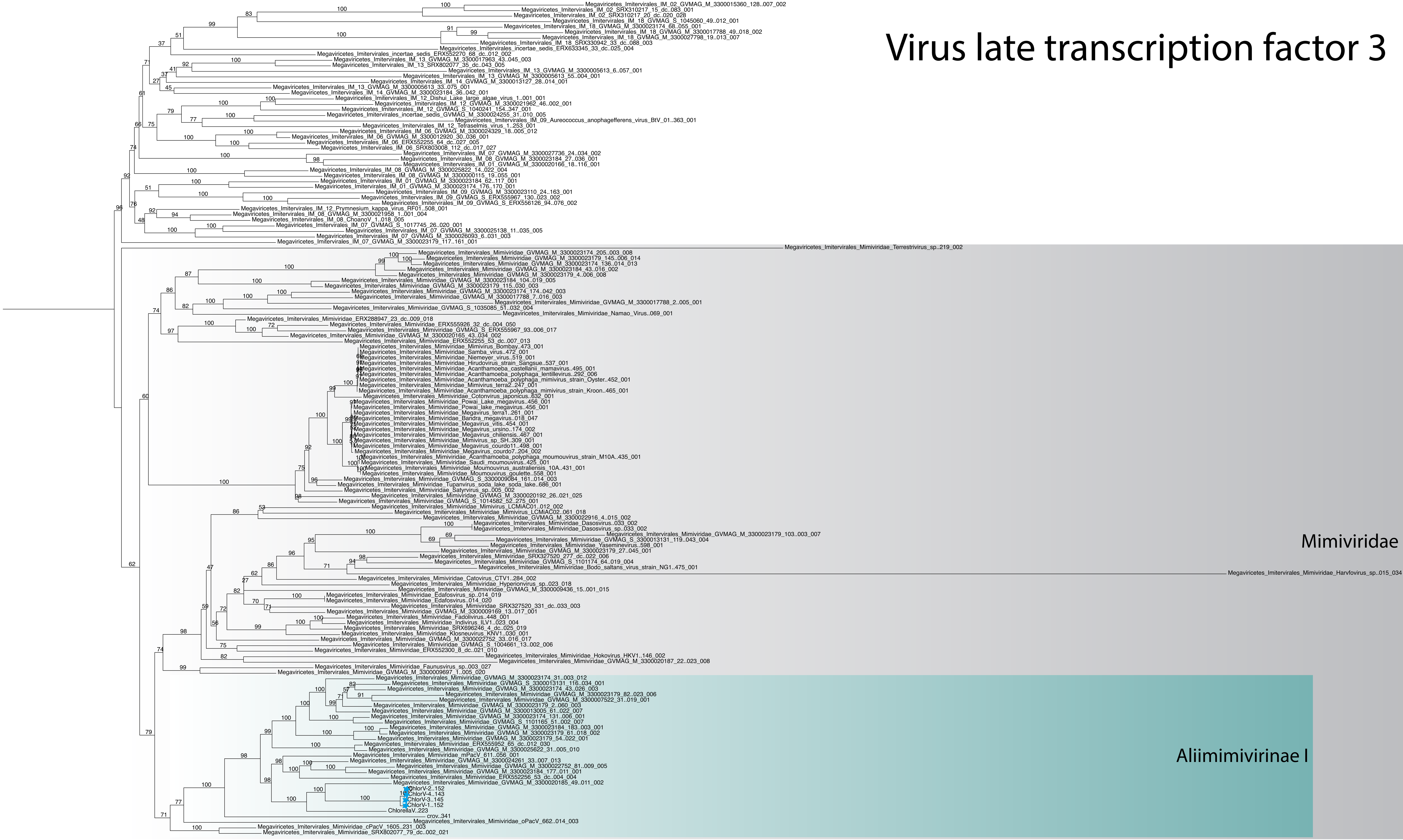

Supplement: Supplementary_Material_wrag093 [file supplementary_material_wrag093.zip › Supplementary_File1_single_gene_trees.pdf]
