# Supplementary material for: Strain-level diversity of giant viruses infecting chlorarachniophyte algae in the subtropical North Pacific
Source: ISME J. 2026 Apr 16;20(1):wrag093. doi: 10.1093/ismejo/wrag093 (PMC13196603; doi:10.1093/ismejo/wrag093)
Supplement: Supplementary_Material_wrag093 [file supplementary_material_wrag093.zip › Supplementary_File2_ChlorV_structural_alignments.pdf]

chlorv-1..001

- Sequence-based annotation for chlorv-1..001 is putative cysteine protease
- Best hit was 2jjq chain A: UNCHARACTERIZED RNA METHYLTRANSFERASE PYRAB10780

target	prob	fident	alnlen	evalue	theader
2jjq-assembly1.cif.gz_A	1	0.158	221	6.58e-10	The crystal structure of Pyrococcus abyssi tRNA (uracil-54, C5)- methyltransferase in complex with S-adenosyl-L-homocysteine
5zq8-assembly1.cif.gz_B	1	0.162	216	8.932e-10	Crystal structure of spRlmCD with U747 stemloop RNA
1uwv-assembly1.cif.gz_A	1	0.134	208	1.009e-09	Crystal Structure of RumA, the iron-sulfur cluster containing E. coli 23S Ribosomal RNA 5-Methyluridine Methyltransferase

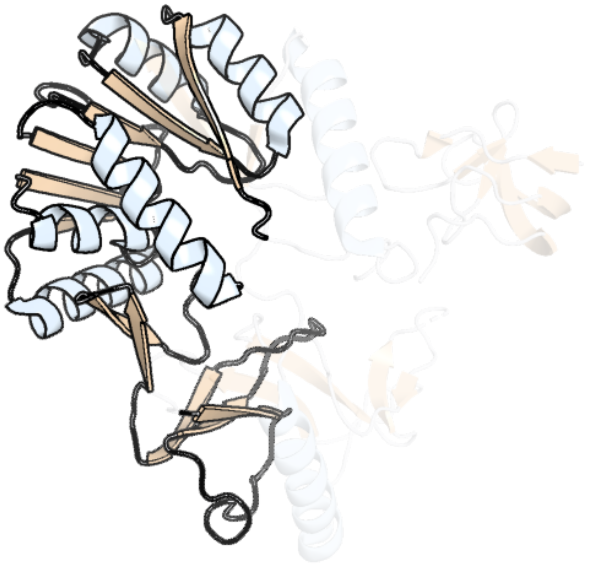

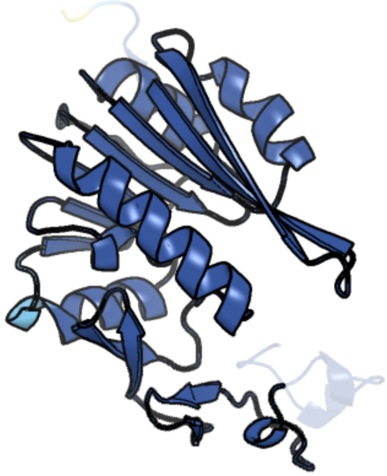

Figure 1: left: reference structure of 2jjq chain A. right: predicted structure of chlorv-1..001, unaligned sequences are shown as transparent

chlorv-1..002

- Sequence-based annotation for chlorv-1..002 is putative Zinc finger
- No significant structural hit found

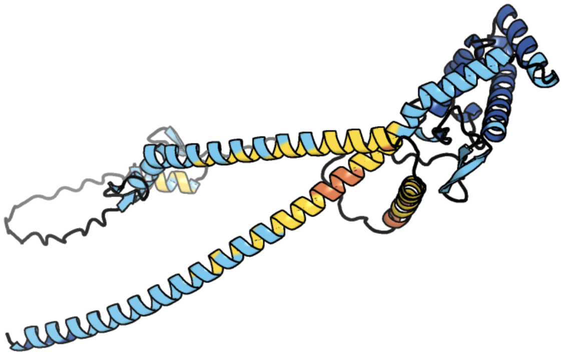

Figure 2: predicted structure of chlorv-1..002

chlorv-1..003

- Sequence-based annotation for chlorv-1..003 is putative Methyltransferase
- Best hit was 2py6 chain A: Methyltransferase FkbM

target	prob	fident	alnlen	evalue	theader
2py6-assembly1.cif.gz_A	1	0.122	221	4.785e-06	Crystal structure of Methyltransferase FkbM (YP_546752.1) from Methylobacillus flagellatus KT at 2.20 A resolution
6rxx-assembly1.cif.gz_CB	1	0.091	218	0.0001177	Cryo-EM structure of the 90S pre-ribosome (Kre33-Noc4) from Chaetomium thermophilum, state C, Poly-Ala
3e05-assembly2.cif.gz_C	1	0.109	219	0.0001177	CRYSTAL STRUCTURE OF Precorrin-6y C5,15-methyltransferase FROM Geobacter metallireducens GS-15

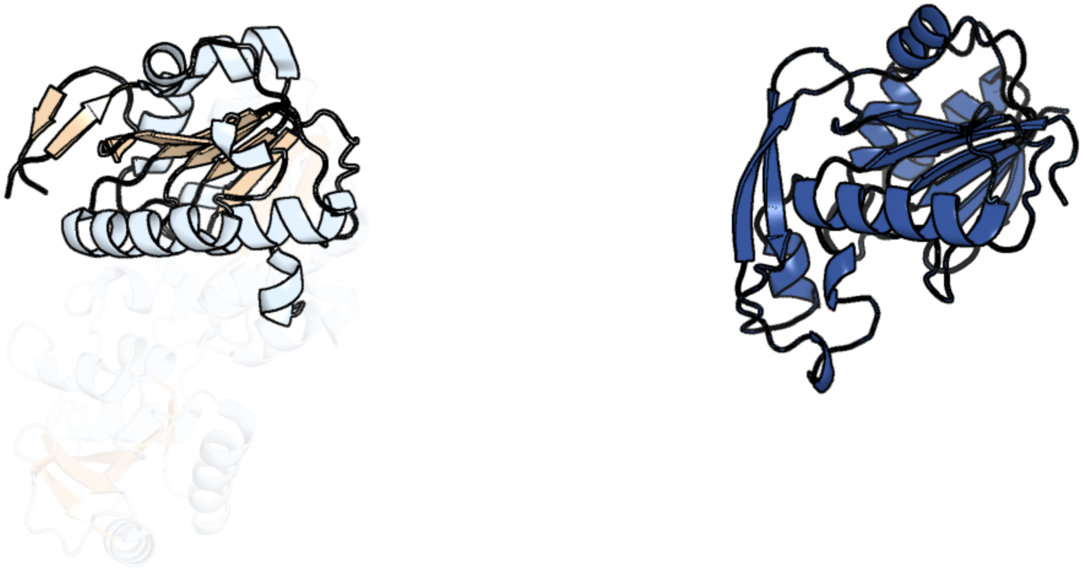

Figure 3: left: reference structure of 2py6 chain A. right: predicted structure of chlorv-1..003, unaligned sequences are shown as transparent

chlorv-1..004

- Sequence-based annotation for chlorv-1..004 is hypothetical protein
- No significant structural hit found

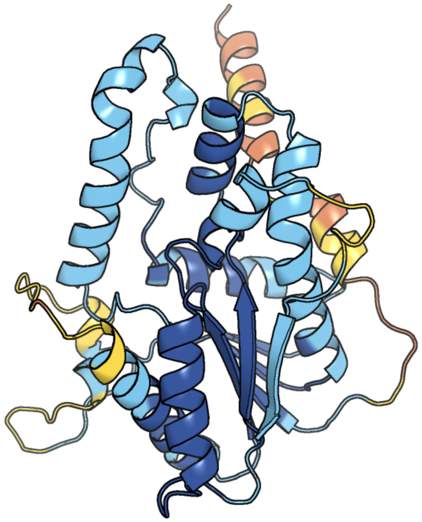

Figure 4: predicted structure of chlorv-1..004

chlorv-1..005

- Sequence-based annotation for chlorv-1..005 is putative Acetyltransferase
- Best hit was 1yvk chain D: hypothetical protein BSU33890

target	prob	fident	alnlen	evalue	theader
1yvk-assembly1.cif.gz_D	1	0.114	149	3.843e-06	Crystal Structure of the Bacillis subtilis Acetyltransferase in complex with CoA, Northeast Structural Genomics Target SR237.
3owc-assembly2.cif.gz_B	1	0.181	110	3.843e-06	Crystal structure of GNAT superfamily protein PA2578 from Pseudomonas aeruginosa
5ix3-assembly1.cif.gz_A	1	0.238	113	6.661e-06	Crystal structure of N-acetyltransferase from Staphylococcus aureus.

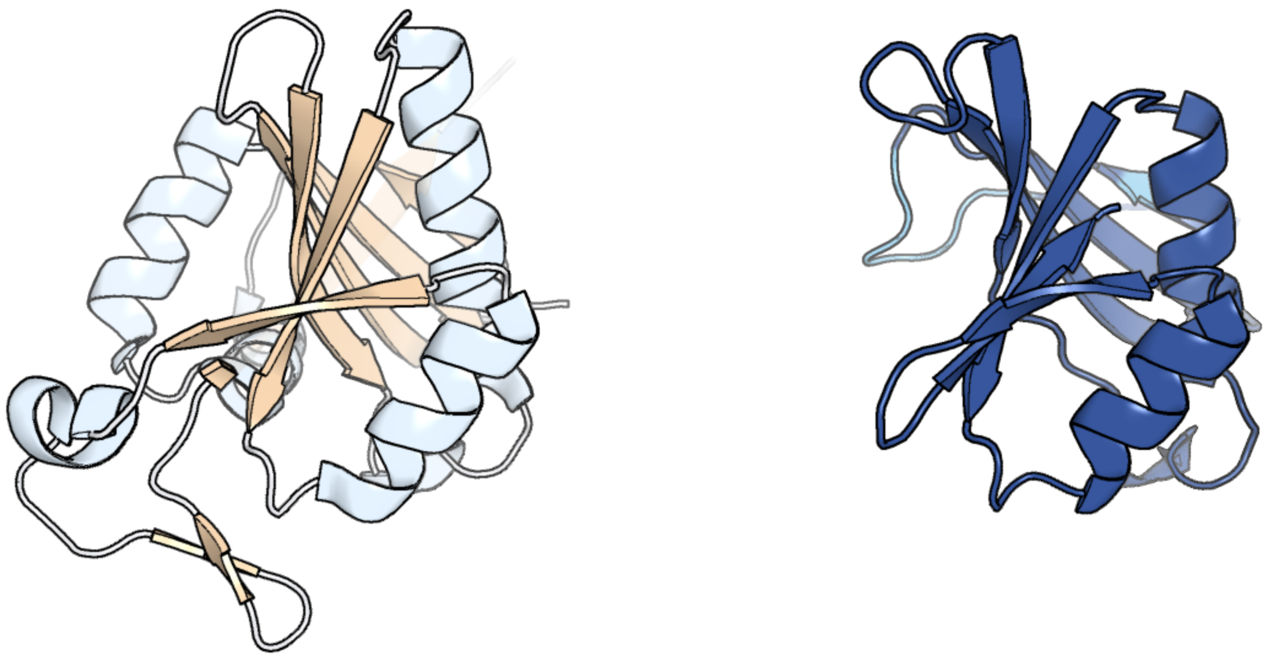

Figure 5: left: reference structure of 1yvk chain D. right: predicted structure of chlorv-1..005, unaligned sequences are shown as transparent

chlorv-1..006

- Sequence-based annotation for chlorv-1..006 is putative FAD/NAD(P)-binding domain
- Best hit was 6f7l chain B: Amine oxidase LkcE

target	prob	fident	alnlen	evalue	theader
6f7l-assembly1.cif.gz_B	1	0.118	412	1.371e-17	Crystal structure of LkcE R326Q mutant in complex with its substrate
6f7l-assembly1.cif.gz_A	1	0.115	416	1.449e-17	Crystal structure of LkcE R326Q mutant in complex with its substrate
6f32-assembly1.cif.gz_A	1	0.113	423	2.806e-17	Crystal structure of a dual function amine oxidase/cyclase in complex with substrate analogues

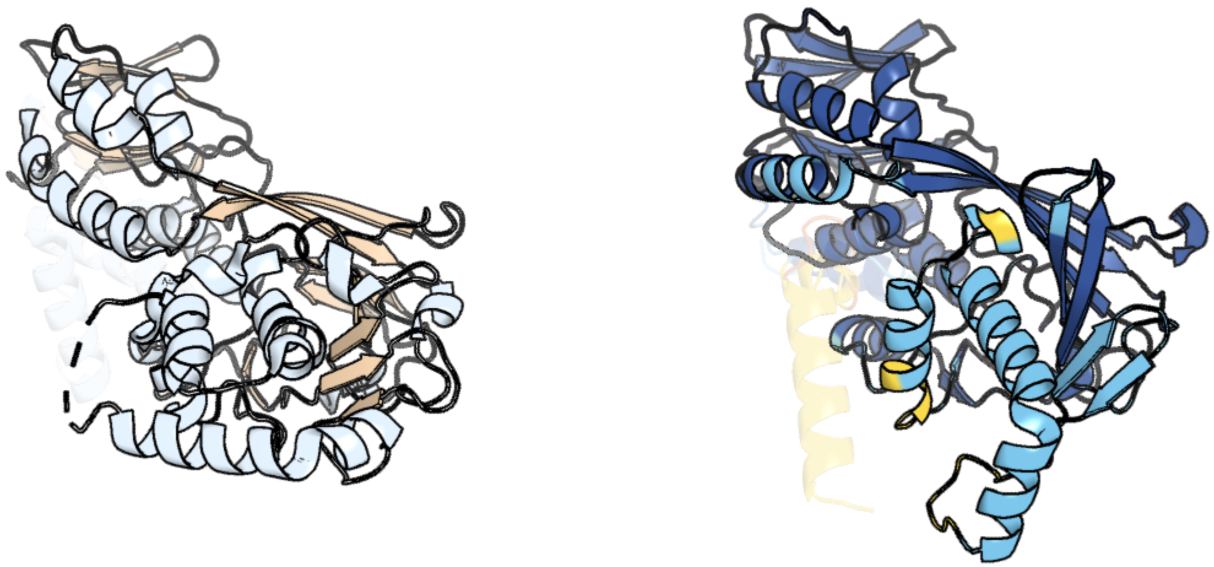

Figure 6: left: reference structure of 6f7l chain B. right: predicted structure of chlorv-1..006, unaligned sequences are shown as transparent

chlorv-1..007

- Sequence-based annotation for chlorv-1..007 is putative DNA topoisomerase/gyrase
- Best hit was 4gfh chain F: DNA topoisomerase 2

target	prob	fident	alnlen	evalue	theader
4gfh-assembly1.cif.gz_F	1	0.421	1123	0	Topoisomerase II-DNA-AMPPNP complex
6zy8-assembly1.cif.gz_A	1	0.422	1142	0	Cryo-EM structure of the entire Human topoisomerase II alpha in State 2
6zy7-assembly1.cif.gz_B	1	0.42	1139	0	Cryo-EM structure of the entire Human topoisomerase II alpha in State 1

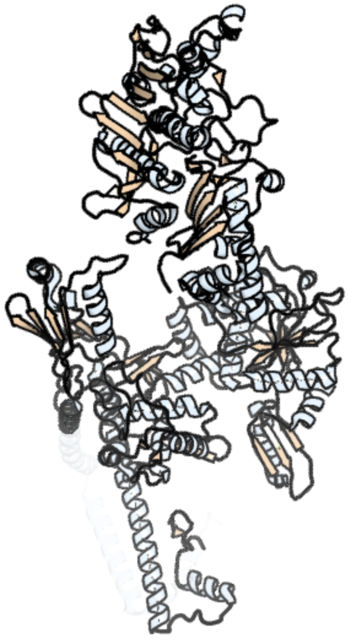

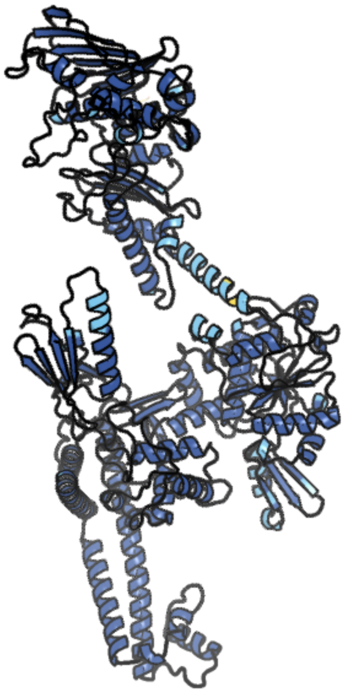

Figure 7: left: reference structure of 4gfh chain F. right: predicted structure of chlorv-1..007, unaligned sequences are shown as transparent

chlorv-1..008

- Sequence-based annotation for chlorv-1..008 is putative Glycosyltransferase
- Best hit was 2z87 chain A: Chondroitin synthase

target	prob	fident	alnlen	evalue	theader
2z87-assembly2.cif.gz_A	1	0.102	596	2.551e-12	Crystal structure of chondroitin polymerase from Escherichia coli strain K4 (K4CP) complexed with UDP-GalNAc and UDP
2z86-assembly1.cif.gz_A	1	0.1	585	2.692e-12	Crystal structure of chondroitin polymerase from Escherichia coli strain K4 (K4CP) complexed with UDP-GlcUA and UDP
2z86-assembly1.cif.gz_B	1	0.096	611	5.423e-12	Crystal structure of chondroitin polymerase from Escherichia coli strain K4 (K4CP) complexed with UDP-GlcUA and UDP

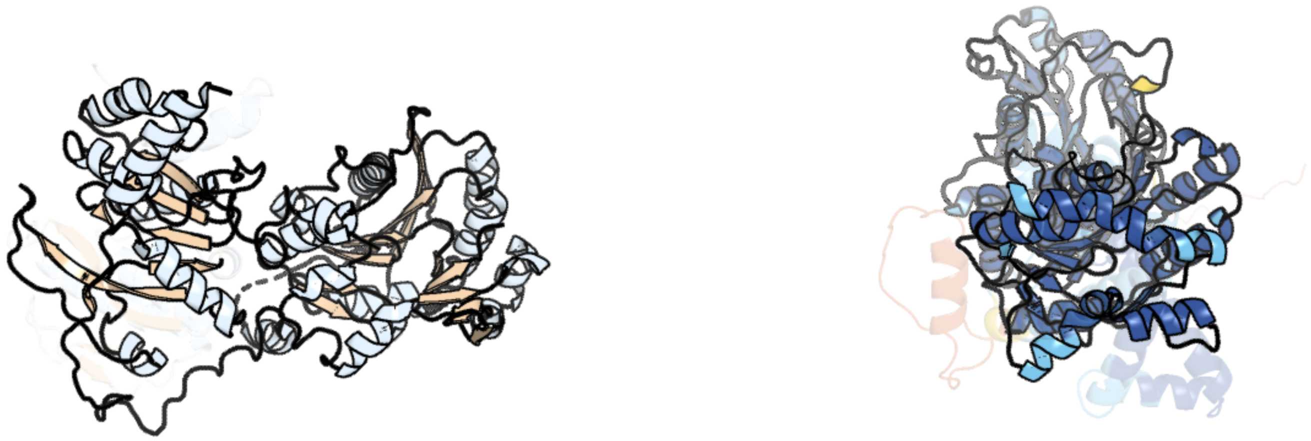

Figure 8: left: reference structure of 2z87 chain A. right: predicted structure of chlorv-1..008, unaligned sequences are shown as transparent

chlorv-1..009

- Sequence-based annotation for chlorv-1..009 is putative RING-finger-containing E3 ubiquitin ligase
- No significant structural hit found

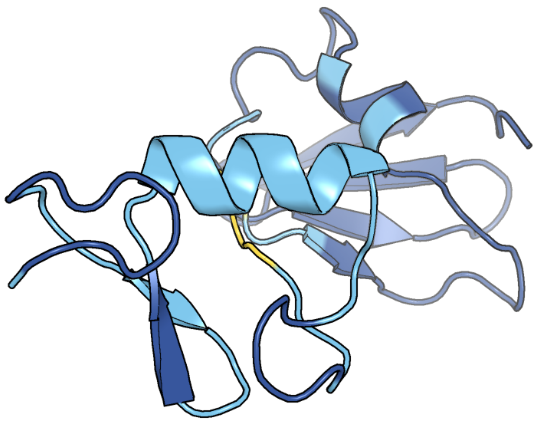

Figure 9: predicted structure of chlorv-1..009

chlorv-1..010

- Sequence-based annotation for chlorv-1..010 is putative Protein kinase
- Best hit was 5jzn chain A: Serine/threonine-protein kinase DCLK1

target	prob	fident	alnlen	evalue	theader
5jzn-assembly1.cif.gz_A	1	0.166	265	1.214e-11	Crystal structure of DCLK1-KD in complex with NVP-TAE684
5jzj-assembly1.cif.gz_A	1	0.16	287	1.214e-11	Crystal structure of DCLK1-KD in complex with AMPPN
7kx6-assembly1.cif.gz_A	1	0.171	274	1.284e-11	Crystal structure of DCLK1-KD in complex with XMD8-85

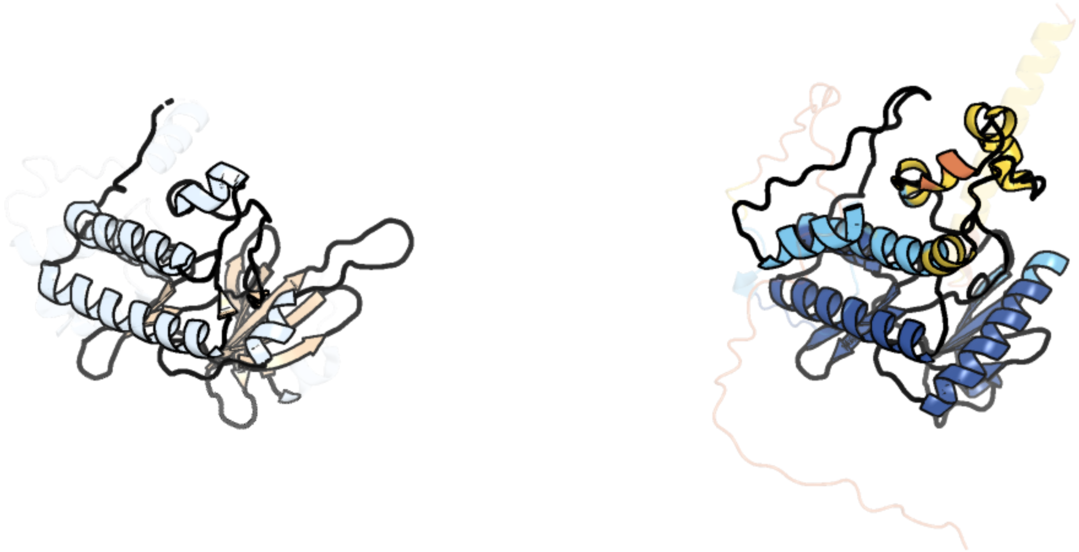

Figure 10: left: reference structure of 5jzn chain A. right: predicted structure of chlorv-1..010, unaligned sequences are shown as transparent

chlorv-1..011

- Sequence-based annotation for chlorv-1..011 is hypothetical protein
- No significant structural hit found

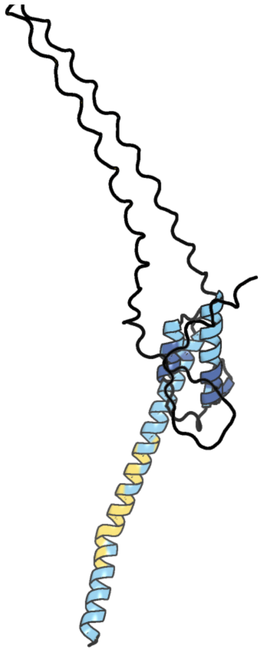

Figure 11: predicted structure of chlorv-1..011

chlorv-1..012

- Sequence-based annotation for chlorv-1..012 is putative Helicase
- Best hit was 5lta chain A: Pre-mRNA-splicing factor ATP-dependent RNA helicase PRP43

target	prob	fident	alnlen	evalue	theader
5lta-assembly1.cif.gz_A	1	0.202	549	2.423e-19	Crystal structure of the Prp43-ADP-BeF3-U7-RNA complex
6zww-assembly2.cif.gz_C	1	0.2	534	5.952e-19	Crystal structure of E. coli RNA helicase HrpA in complex with RNA
5vhd-assembly1.cif.gz_D	1	0.169	620	1.337e-18	DHX36 with an N-terminal truncation bound to ADP-AlF4

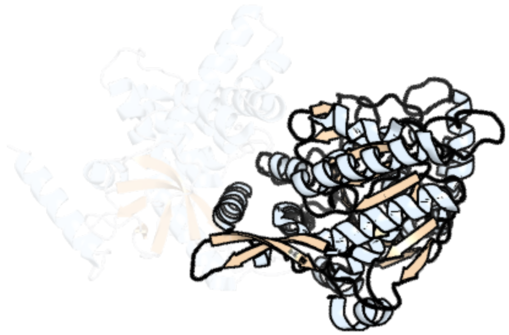

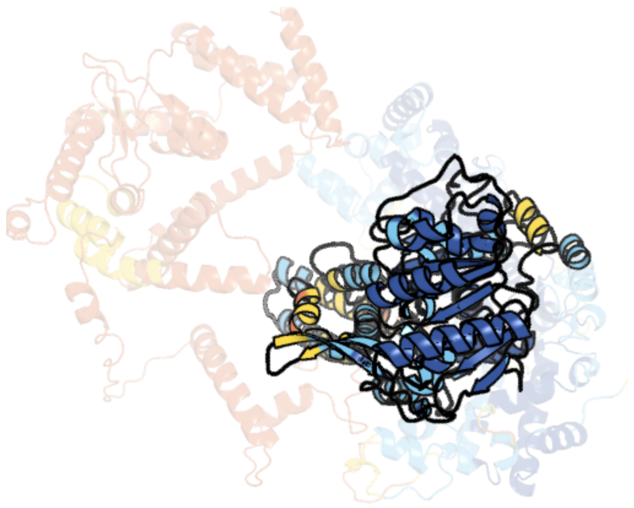

Figure 12: left: reference structure of 5lta chain A. right: predicted structure of chlorv-1..012, unaligned sequences are shown as transparent

chlorv-1..013

- Sequence-based annotation for chlorv-1..013 is putative Phosphoglycerate mutase
- Best hit was 2yn2 chain A: UNCHARACTERIZED PROTEIN YNL108C

target	prob	fident	alnlen	evalue	theader
2yn2-assembly1.cif.gz_A	1	0.216	226	2.573e-11	Huf protein - paralogue of the tau55 histidine phosphatase domain
2yn0-assembly1.cif.gz_A	1	0.205	243	3.114e-11	tau55 histidine phosphatase domain
4ij6-assembly1.cif.gz_B	1	0.163	208	3.114e-11	Crystal Structure of a Novel-type Phosphoserine Phosphatase Mutant (H9A) from Hydrogenobacter thermophilus TK-6 in Complex with L-phosphoserine

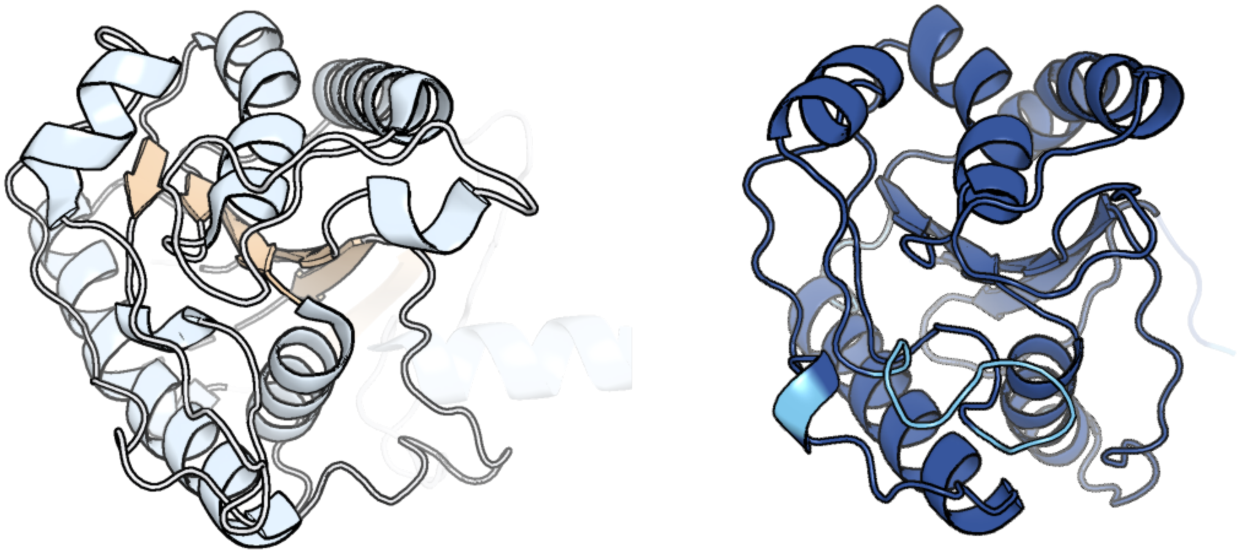

Figure 13: left: reference structure of 2yn2 chain A. right: predicted structure of chlorv-1..013, unaligned sequences are shown as transparent

chlorv-1..014

- Sequence-based annotation for chlorv-1..014 is hypothetical protein
- No significant structural hit found

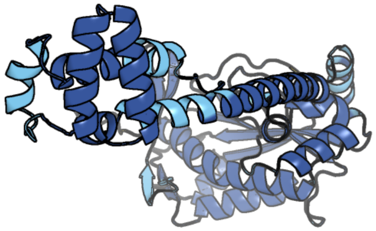

Figure 14: predicted structure of chlorv-1..014

chlorv-1..015

- Sequence-based annotation for chlorv-1..015 is putative DnaJ domain
- Best hit was 7zhs chain A: Ubiquitin-like protein SMT3,DnaJ homolog subfamily A member 2

target	prob	fident	alnlen	evalue	theader
7zhs-assembly1.cif.gz_A	1	0.283	243	1.888e-24	3D reconstruction of the cylindrical assembly of DnaJA2 delta G/F by imposing D5 symmetry
1nlt-assembly1.cif.gz_A	1	0.246	227	4.557e-19	The crystal structure of Hsp40 Ydj1
6jzb-assembly1.cif.gz_A	1	0.175	245	2.865e-18	Structural characterization of DnaJ from Streptococcus pneumonia presents a new tetramer of Hsp40 family

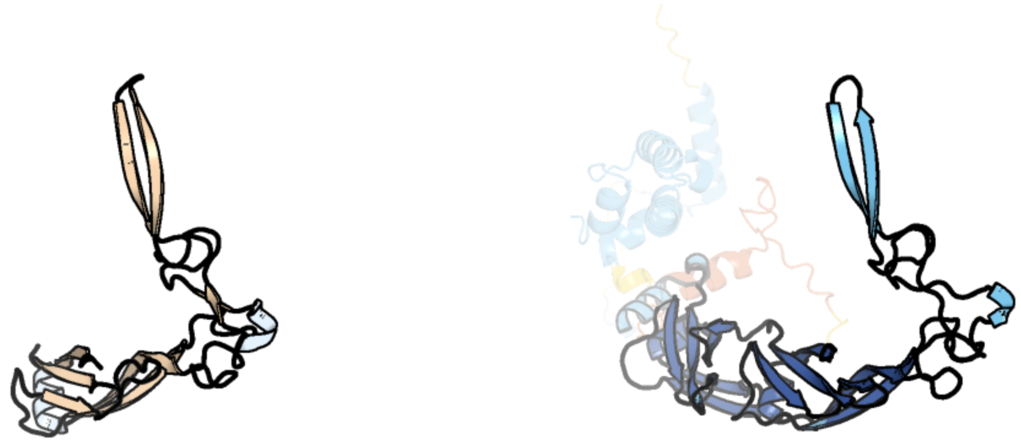

Figure 15: left: reference structure of 7zhs chain A. right: predicted structure of chlorv-1..015, unaligned sequences are shown as transparent

chlorv-1..016

- Sequence-based annotation for chlorv-1..016 is putative DNA clamp
- Best hit was 5yd8 chain X: Proliferating cell nuclear antigen

target	prob	fident	alnlen	evalue	theader
5yd8-assembly2.cif.gz_X	1	0.261	264	5.365e-26	Crystal structure of human PCNA in complex with APIM of human ZRANB3
3tbl-assembly1.cif.gz_A	1	0.26	265	9.252e-26	Structure of Mono-ubiquitinated PCNA: Implications for DNA Polymerase Switching and Okazaki Fragment Maturation
6gis-assembly1.cif.gz_B	1	0.261	264	1.596e-25	Structural basis of human clamp sliding on DNA

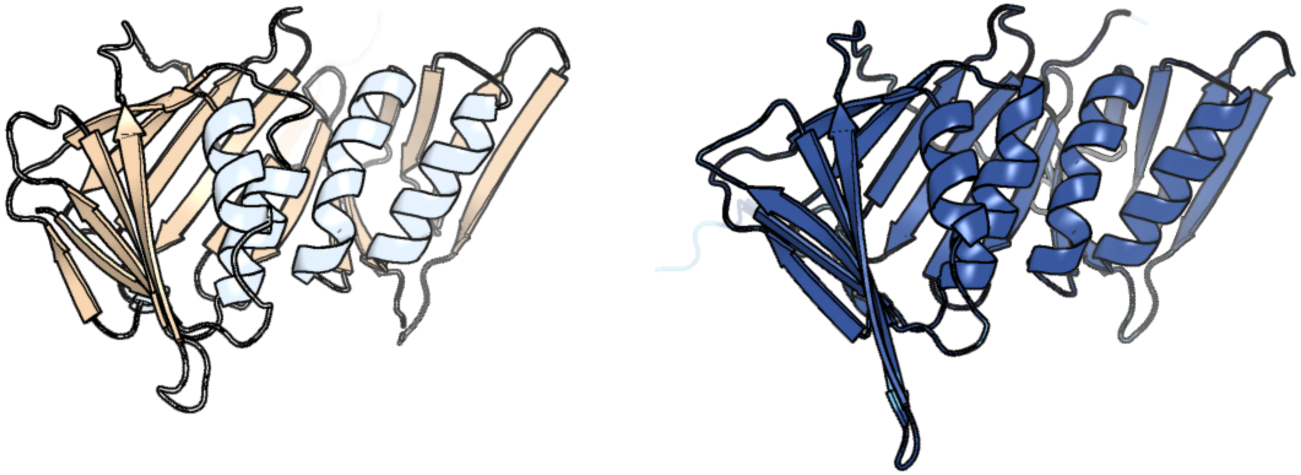

Figure 16: left: reference structure of 5yd8 chain X. right: predicted structure of chlorv-1..016, unaligned sequences are shown as transparent

chlorv-1..017

- Sequence-based annotation for chlorv-1..017 is putative eukaryotic translation initiation factor 4E
- Best hit was 4axg chain A: EUKARYOTIC TRANSLATION INITIATION FACTOR 4E

target	prob	fident	alnlen	evalue	theader
4axg-assembly1.cif.gz_A	1	0.246	162	7.028e-12	Structure of eIF4E-Cup complex
4ueb-assembly1.cif.gz_C	1	0.236	169	2.121e-11	Complex of D. melanogaster eIF4E with a designed 4E-binding protein (Form II)
5abv-assembly4.cif.gz_G	1	0.217	170	2.884e-11	Complex of D. melanogaster eIF4E with the 4E-binding protein Mextli

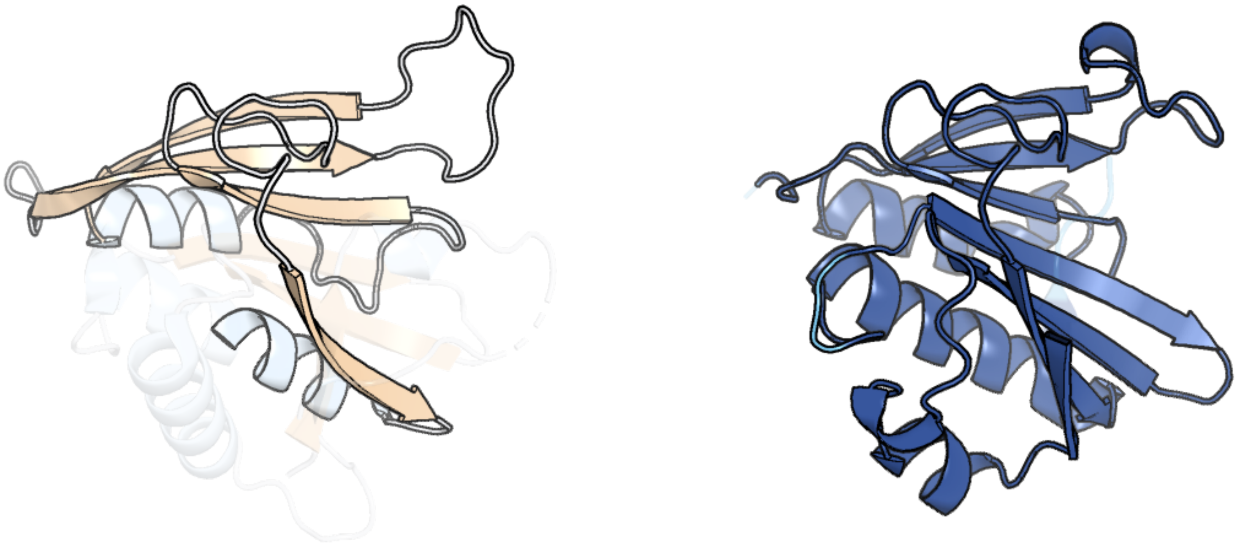

Figure 17: left: reference structure of 4axg chain A. right: predicted structure of chlorv-1..017, unaligned sequences are shown as transparent

chlorv-1..018

- Sequence-based annotation for chlorv-1..018 is hypothetical protein
- No significant structural hit found

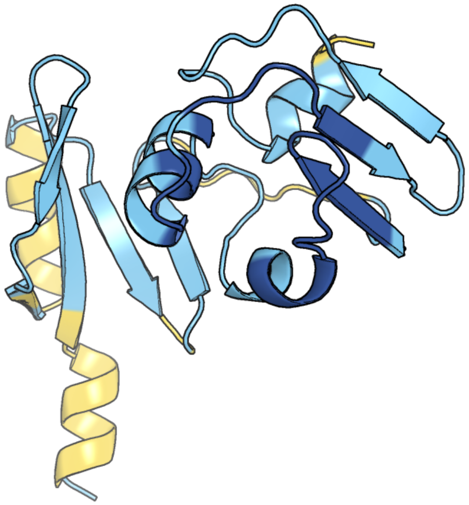

Figure 18: predicted structure of chlorv-1..018

chlorv-1..019

- Sequence-based annotation for chlorv-1..019 is hypothetical protein
- No significant structural hit found

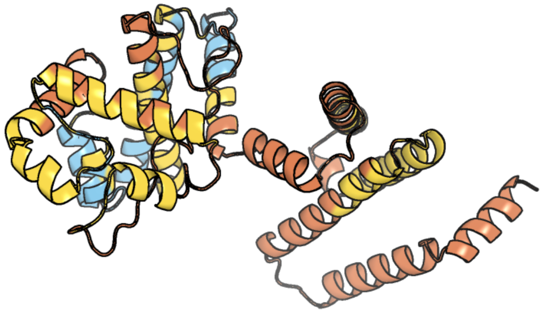

Figure 19: predicted structure of chlorv-1..019

chlorv-1..020

- Sequence-based annotation for chlorv-1..020 is hypothetical protein
- No significant structural hit found

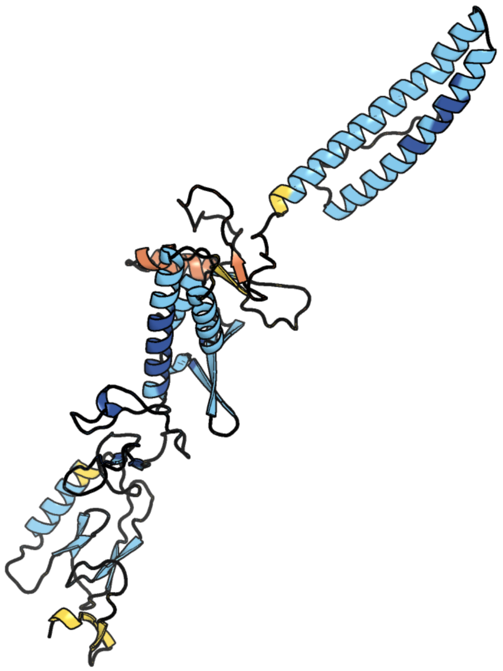

Figure 20: predicted structure of chlorv-1..020

chlorv-1..021

- Sequence-based annotation for chlorv-1..021 is hypothetical protein
- No significant structural hit found

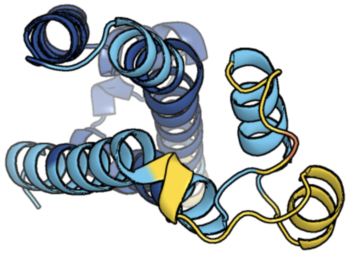

Figure 21: predicted structure of chlorv-1..021

chlorv-1..022

- Sequence-based annotation for chlorv-1..022 is putative Methyltransferase
- Best hit was 3gdh chain C: Trimethylguanosine synthase homolog

target	prob	fident	alnlen	evalue	theader
3gdh-assembly3.cif.gz_C	1	0.216	203	2.148e-09	Methyltransferase domain of human Trimethylguanosine Synthase 1 (TGS1) bound to m7GTP and adenosyl-homocysteine (active form)
3gdh-assembly2.cif.gz_B	1	0.221	203	3.956e-09	Methyltransferase domain of human Trimethylguanosine Synthase 1 (TGS1) bound to m7GTP and adenosyl-homocysteine (active form)
3egi-assembly1.cif.gz_A	1	0.216	189	1.235e-07	Methyltransferase domain of human trimethylguanosine synthase TGS1 bound to m7GpppA (inactive form)

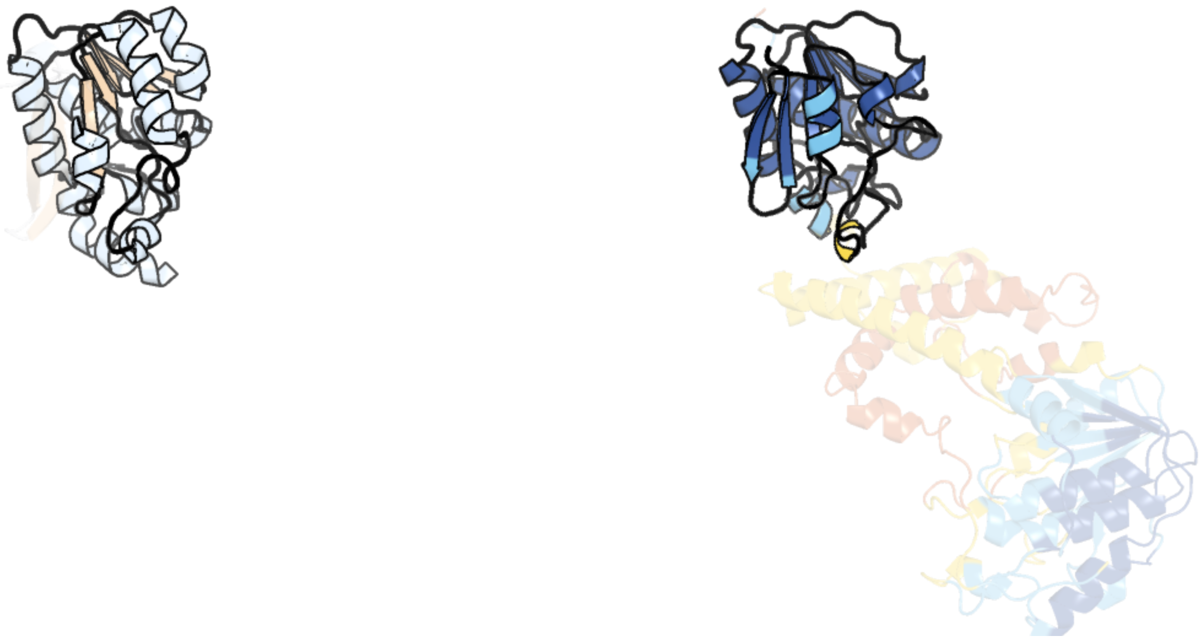

Figure 22: left: reference structure of 3gdh chain C. right: predicted structure of chlorv-1..022, unaligned sequences are shown as transparent

chlorv-1..023

- Sequence-based annotation for chlorv-1..023 is hypothetical protein
- No significant structural hit found

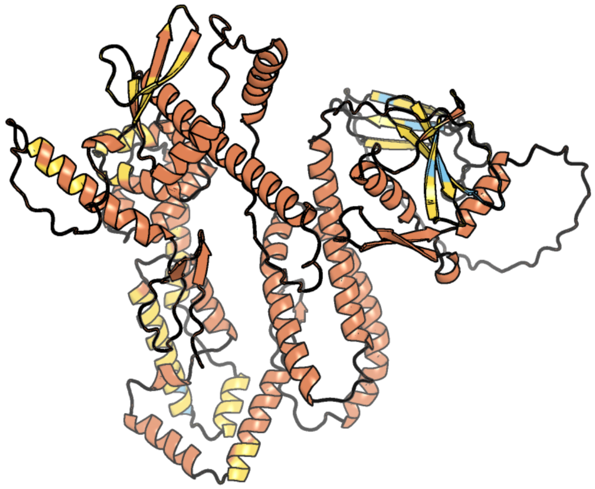

Figure 23: predicted structure of chlorv-1..023

chlorv-1..024

- Sequence-based annotation for chlorv-1..024 is putative Helicase
- Best hit was 7amv chain W: ATP-dependent helicase VETFS

target	prob	fident	alnlen	evalue	theader
7amv-assembly1.cif.gz_W	1	0.165	858	2.792e-22	Atomic structure of the poxvirus transcription pre-initiation complex in the initially melted state
6rfl-assembly1.cif.gz_Y	1	0.163	702	3.652e-15	Structure of the complete Vaccinia DNA-dependent RNA polymerase complex
7aoh-assembly1.cif.gz_Y	1	0.157	724	9.751e-15	Atomic structure of the poxvirus late initially transcribing complex

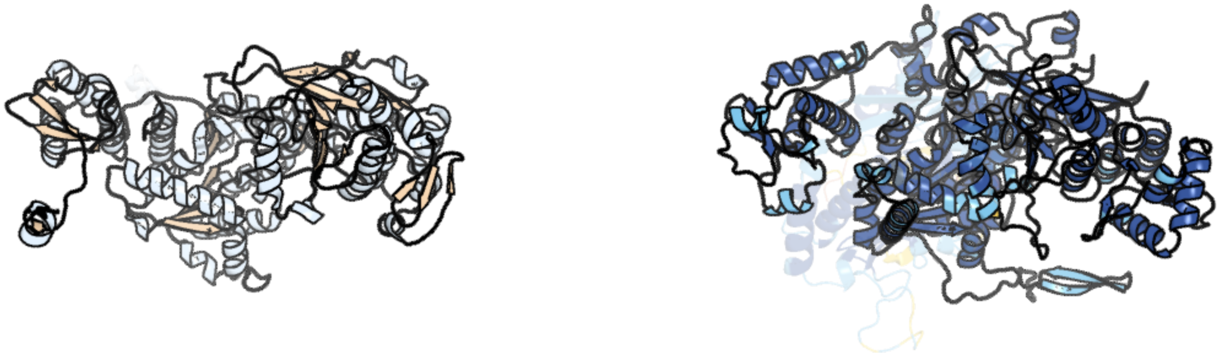

Figure 24: left: reference structure of 7amv chain W. right: predicted structure of chlorv-1..024, unaligned sequences are shown as transparent

chlorv-1..025

- Sequence-based annotation for chlorv-1..025 is hypothetical protein
- No significant structural hit found

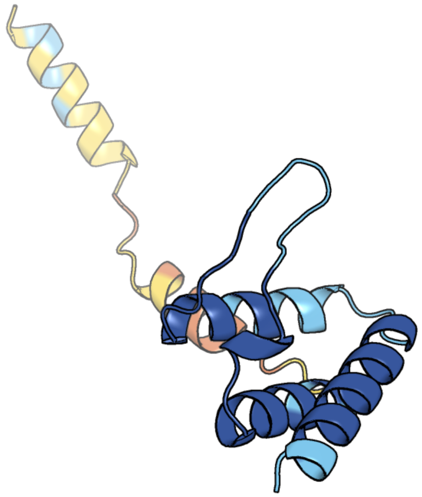

Figure 25: predicted structure of chlorv-1..025

chlorv-1..026

- Sequence-based annotation for chlorv-1..026 is hypothetical protein
- No significant structural hit found

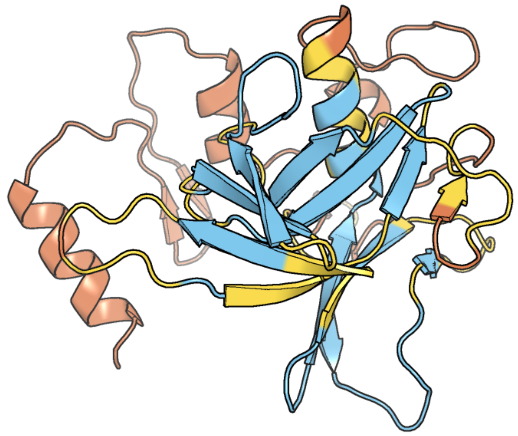

Figure 26: predicted structure of chlorv-1..026

chlorv-1..027

- Sequence-based annotation for chlorv-1..027 is putative POZ (Pox virus and Zinc finger) domain
- Best hit was 8h37 chain A: Kelch repeat and BTB domain-containing protein 2

target	prob	fident	alnlen	evalue	theader
8h37-assembly1.cif.gz_A	1	0.145	213	8.206e-07	Cryo-EM Structure of the KBTBD2-CUL3-Rbx1-p85a tetrameric complex
8h37-assembly1.cif.gz_P	1	0.147	217	1.543e-06	Cryo-EM Structure of the KBTBD2-CUL3-Rbx1-p85a tetrameric complex
6i2m-assembly1.cif.gz_A-2	1	0.155	200	2.178e-06	Crystal structure of vaccinia virus protein A55 BTB-Back domain in complex with human Cullin-3 N-terminus

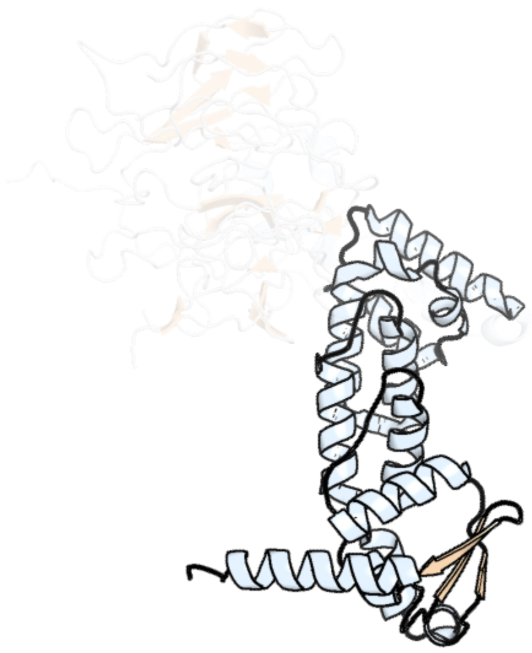

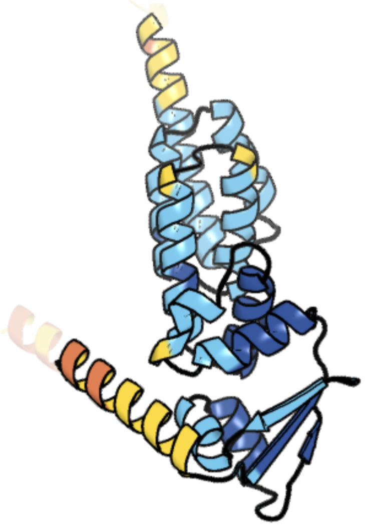

Figure 27: left: reference structure of 8h37 chain A. right: predicted structure of chlorv-1..027, unaligned sequences are shown as transparent

chlorv-1..028

- Sequence-based annotation for chlorv-1..028 is hypothetical protein
- No significant structural hit found

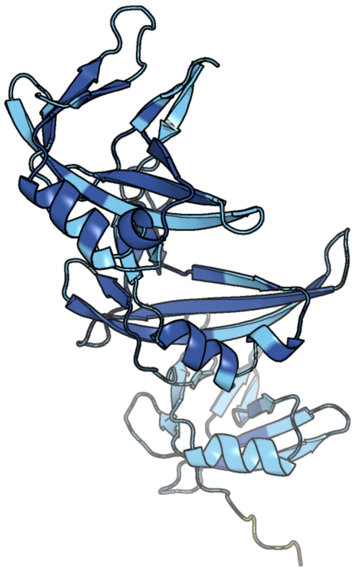

Figure 28: predicted structure of chlorv-1..028

chlorv-1..029

- Sequence-based annotation for chlorv-1..029 is hypothetical protein
- No significant structural hit found

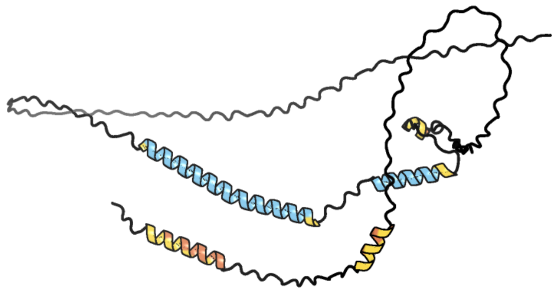

Figure 29: predicted structure of chlorv-1..029

chlorv-1..030

- Sequence-based annotation for chlorv-1..030 is hypothetical protein
- No significant structural hit found

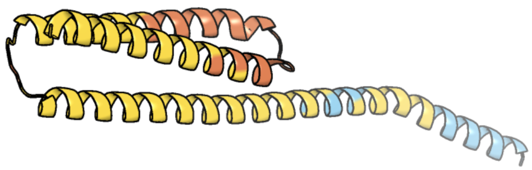

Figure 30: predicted structure of chlorv-1..030

chlorv-1..031

- Sequence-based annotation for chlorv-1..031 is putative DNA polymerase beta
- Best hit was 4m9h chain A: DNA polymerase beta

target	prob	fident	alnlen	evalue	theader
4m9h-assembly1.cif.gz_A	1	0.258	356	3.476e-22	DNA Polymerase Beta E295K Soaked with dTTP
6uok-assembly2.cif.gz_A	1	0.264	351	3.891e-22	Y271G DNA polymerase beta substrate complex with templating cytosine and incoming r8-oxo-GTP
4mfc-assembly1.cif.gz_A	1	0.262	358	5.769e-22	Structure of human DNA polymerase beta complexed with O6MG in the template base paired with incoming non-hydrolyzable CTP

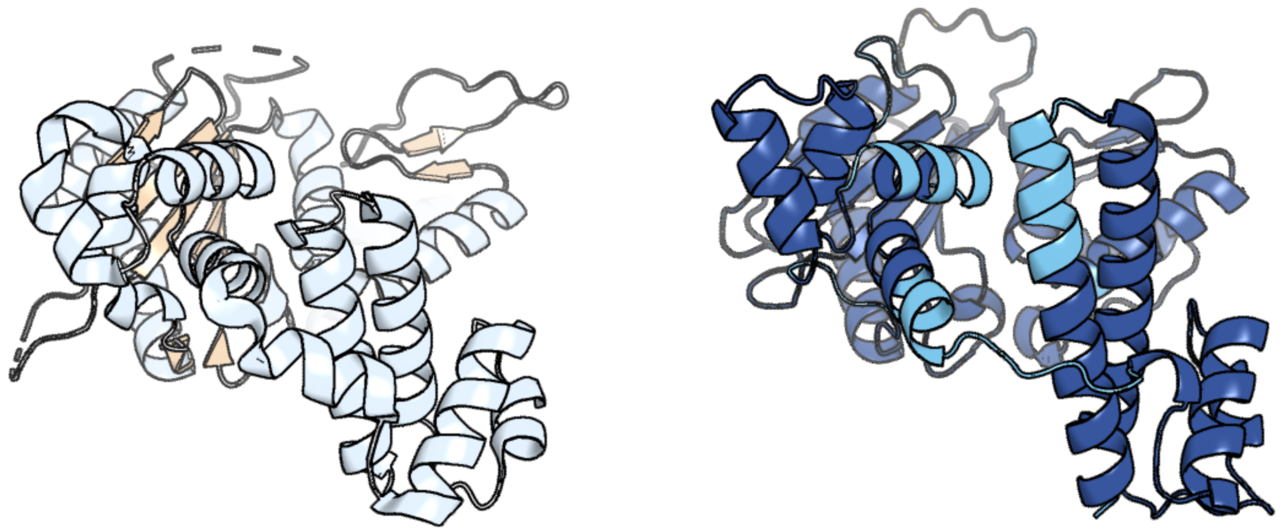

Figure 31: left: reference structure of 4m9h chain A. right: predicted structure of chlorv-1..031, unaligned sequences are shown as transparent

chlorv-1..032

- Sequence-based annotation for chlorv-1..032 is putative Ubiquitin Conjugating Enzyme
- Best hit was 3ptf chain A: Ubiquitin-conjugating enzyme E2 D1

target	prob	fident	alnlen	evalue	theader
3ptf-assembly1.cif.gz_A	1	0.458	144	6.684e-19	X-ray structure of the non-covalent complex between UbcH5A and Ubiquitin
2oxq-assembly1.cif.gz_B	1	0.479	144	7.104e-19	Structure of the UbcH5 :CHIP U-box complex
4wz3-assembly1.cif.gz_A	1	0.465	144	7.104e-19	Crystal structure of the complex between LubX/LegU2/Lpp2887 U-box 1 and Homo sapiens UBE2D2

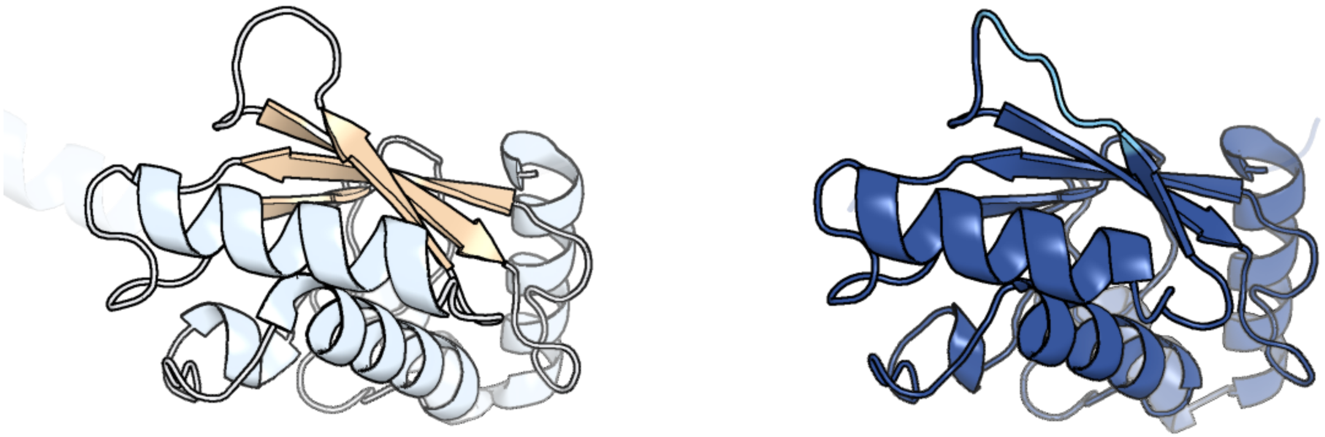

Figure 32: left: reference structure of 3ptf chain A. right: predicted structure of chlorv-1..032, unaligned sequences are shown as transparent

chlorv-1..033

- Sequence-based annotation for chlorv-1..033 is hypothetical protein
- No significant structural hit found

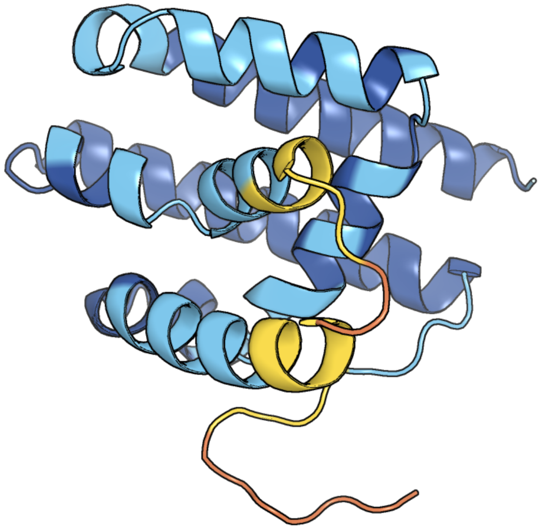

Figure 33: predicted structure of chlorv-1..033

chlorv-1..034

- Sequence-based annotation for chlorv-1..034 is hypothetical protein
- No significant structural hit found

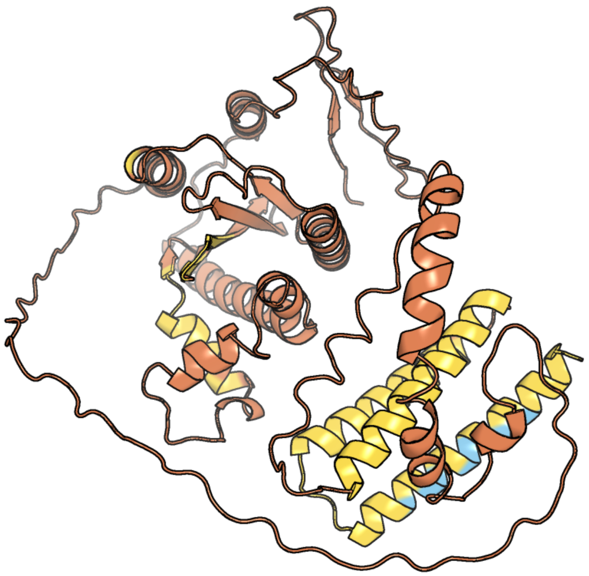

Figure 34: predicted structure of chlorv-1..034

chlorv-1..035

- Sequence-based annotation for chlorv-1..035 is hypothetical protein
- No significant structural hit found

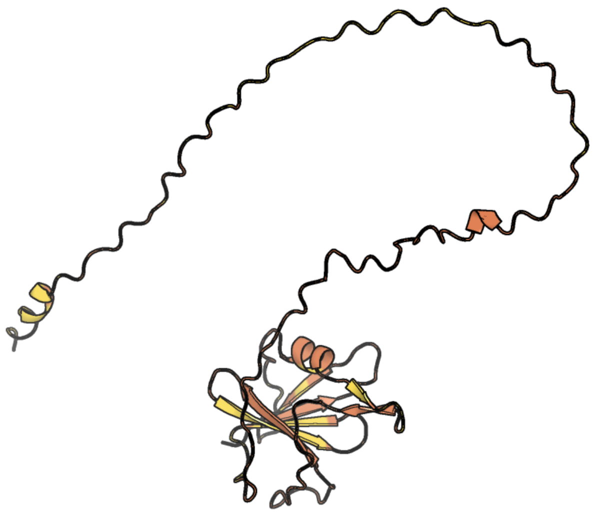

Figure 35: predicted structure of chlorv-1..035

chlorv-1..036

- Sequence-based annotation for chlorv-1..036 is putative Peptidase
- Best hit was 1cb5 chain A: BLEOMYCIN HYDROLASE

target	prob	fident	alnlen	evalue	theader
1cb5-assembly1.cif.gz_A	1	0.336	443	4.469e-45	HUMAN BLEOMYCIN HYDROLASE.
2cb5-assembly1.cif.gz_A	1	0.338	437	1.537e-44	HUMAN BLEOMYCIN HYDROLASE, C73S/DELE455 MUTANT
7v5l-assembly1.cif.gz_A	1	0.338	443	3.373e-44	Crystal structure of human bleomycin hydrolase

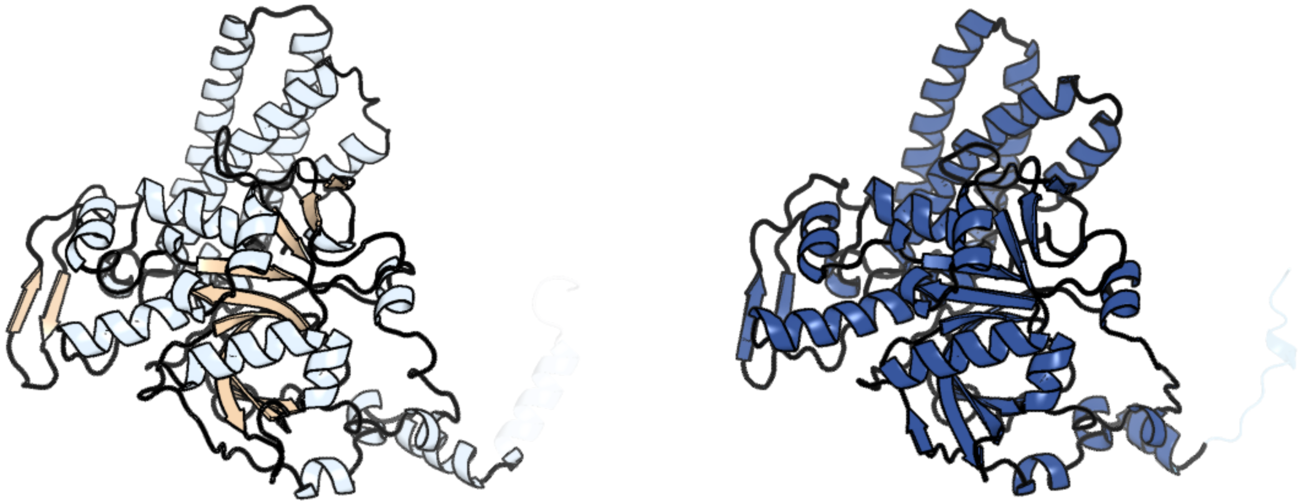

Figure 36: left: reference structure of 1cb5 chain A. right: predicted structure of chlorv-1..036, unaligned sequences are shown as transparent

chlorv-1..037

- Sequence-based annotation for chlorv-1..037 is putative Methyltransferase
- Best hit was 3gdh chain C: Trimethylguanosine synthase homolog

target	prob	fident	alnlen	evalue	theader
3gdh-assembly3.cif.gz_C	1	0.212	207	6.271e-13	Methyltransferase domain of human Trimethylguanosine Synthase 1 (TGS1) bound to m7GTP and adenosyl-homocysteine (active form)
3gdh-assembly2.cif.gz_B	1	0.216	203	3.528e-12	Methyltransferase domain of human Trimethylguanosine Synthase 1 (TGS1) bound to m7GTP and adenosyl-homocysteine (active form)
3egi-assembly1.cif.gz_A	1	0.197	197	6.284e-10	Methyltransferase domain of human trimethylguanosine synthase TGS1 bound to m7GpppA (inactive form)

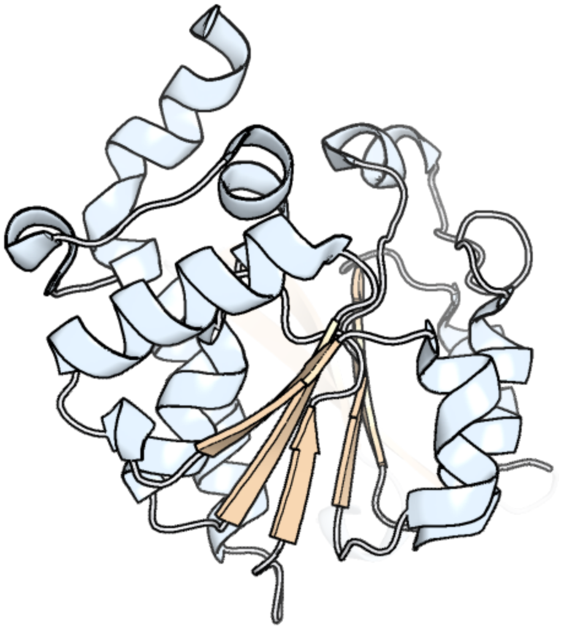

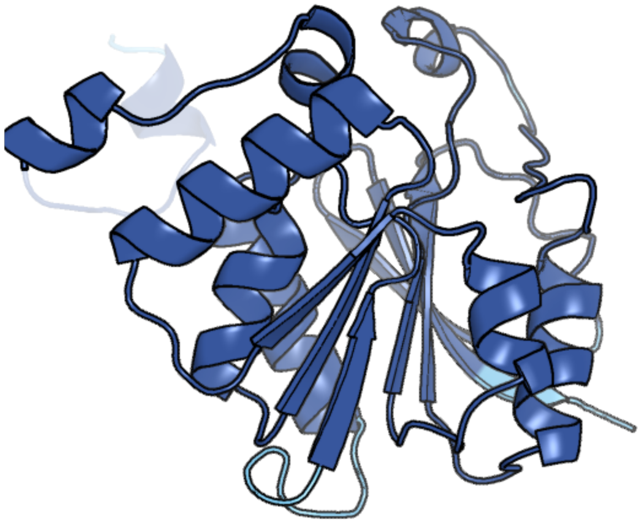

Figure 37: left: reference structure of 3gdh chain C. right: predicted structure of chlorv-1..037, unaligned sequences are shown as transparent

chlorv-1..038

- Sequence-based annotation for chlorv-1..038 is putative Peptidase
- Best hit was 5xu8 chain A: Ubiquitin carboxyl-terminal hydrolase 2

target	prob	fident	alnlen	evalue	theader
5xu8-assembly1.cif.gz_A	1	0.182	434	8.761e-25	Crystal structure of human USP2 in complex with ubiquitin and 6-thioguanine
2ibi-assembly1.cif.gz_A	1	0.168	439	1.039e-24	Covalent Ubiquitin-USP2 Complex
3v6e-assembly1.cif.gz_A	1	0.18	438	1.461e-24	Crystal Structure of USP2 and a mutant form of Ubiquitin

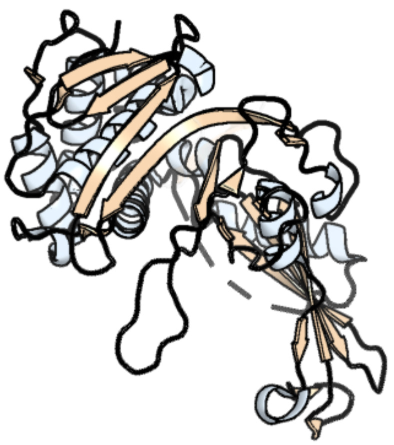

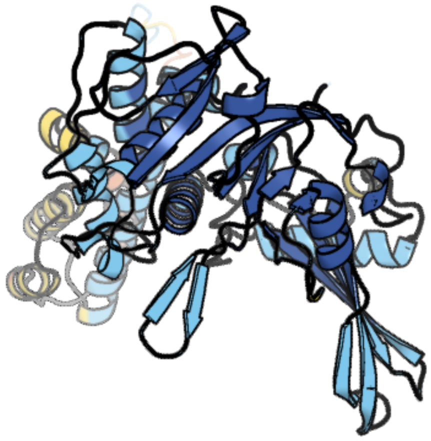

Figure 38: left: reference structure of 5xu8 chain A. right: predicted structure of chlorv-1..038, unaligned sequences are shown as transparent

chlorv-1..039

- Sequence-based annotation for chlorv-1..039 is putative RNA polymerase beta subunit
- Best hit was 8oev chain B: DNA-directed RNA polymerase subunit beta

target	prob	fident	alnlen	evalue	theader
8oev-assembly1.cif.gz_B	1	0.356	1205	2.803e-102	Structure of the mammalian Pol II-SPT6-Elongin complex, lacking ELOA latch (composite structure, structure 3)
8was-assembly1.cif.gz_p	1	0.355	1220	2.81e-101	Structure of transcribing complex 9 (TC9), the initially transcribing complex with Pol II positioned 9nt downstream of TSS.
7pks-assembly1.cif.gz_B	1	0.36	1197	9.796e-101	Structural basis of Integrator-mediated transcription regulation

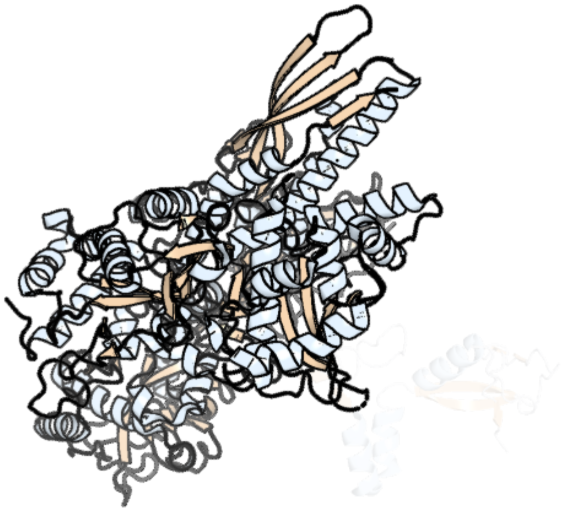

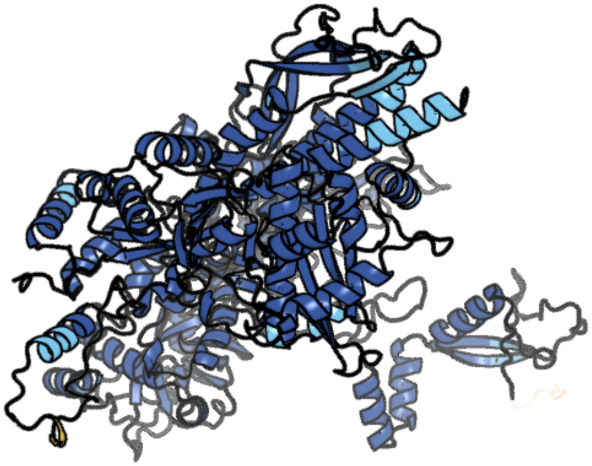

Figure 39: left: reference structure of 8oev chain B. right: predicted structure of chlorv-1..039, unaligned sequences are shown as transparent

chlorv-1..040

- Sequence-based annotation for chlorv-1..040 is putative Translation initiation factor
- No significant structural hit found

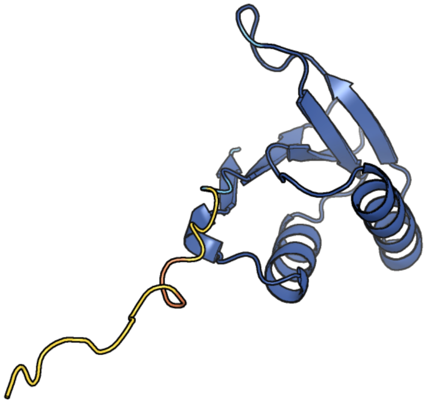

Figure 40: predicted structure of chlorv-1..040

chlorv-1..041

- Sequence-based annotation for chlorv-1..041 is hypothetical protein
- No significant structural hit found

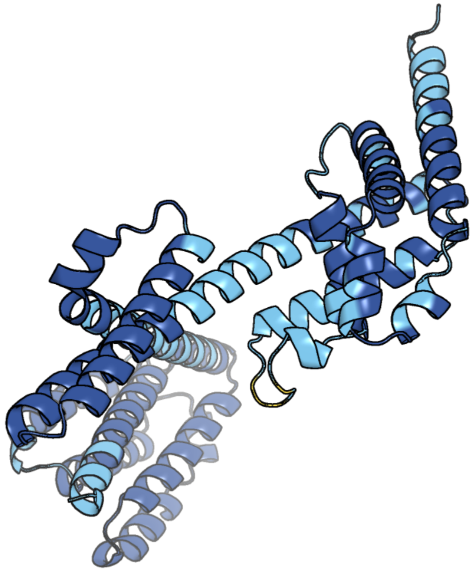

Figure 41: predicted structure of chlorv-1..041

chlorv-1..042

- Sequence-based annotation for chlorv-1..042 is hypothetical protein
- Best hit was 3wr7 chain A: Spermidine N1-acetyltransferase

target	prob	fident	alnlen	evalue	theader
3wr7-assembly1.cif.gz_A-3	1	0.161	161	6.269e-07	Crystal Structure of Spermidine Acetyltransferase from Escherichia coli
8fv0-assembly1.cif.gz_C	1	0.198	161	7.418e-07	SpeG spermidine N-acetyltransferase from Staphylococcus aureus in complex with spermine
6vfn-assembly1.cif.gz_D	1	0.16	162	1.098e-06	Crystal structure of SpeG allosteric polyamine acetyltransferase from Bacillus thuringiensis in complex with spermine

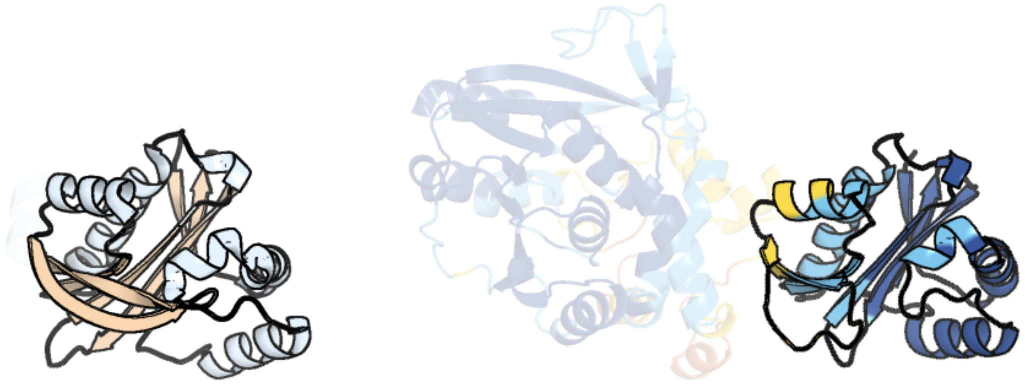

Figure 42: left: reference structure of 3wr7 chain A. right: predicted structure of chlorv-1..042, unaligned sequences are shown as transparent

chlorv-1..043

- Sequence-based annotation for chlorv-1..043 is hypothetical protein
- Best hit was 8asn chain G: Tubulin–tyrosine ligase

target	prob	fident	alnlen	evalue	theader
8asn-assembly1.cif.gz_G	1	0.154	356	2.528e-16	Crystal structure of the apo human TTL in complex with tubulin-stathmin
8asn-assembly1.cif.gz_I	1	0.152	360	3.6e-16	Crystal structure of the apo human TTL in complex with tubulin-stathmin
8asn-assembly1.cif.gz_H	1	0.147	359	8.214e-16	Crystal structure of the apo human TTL in complex with tubulin-stathmin

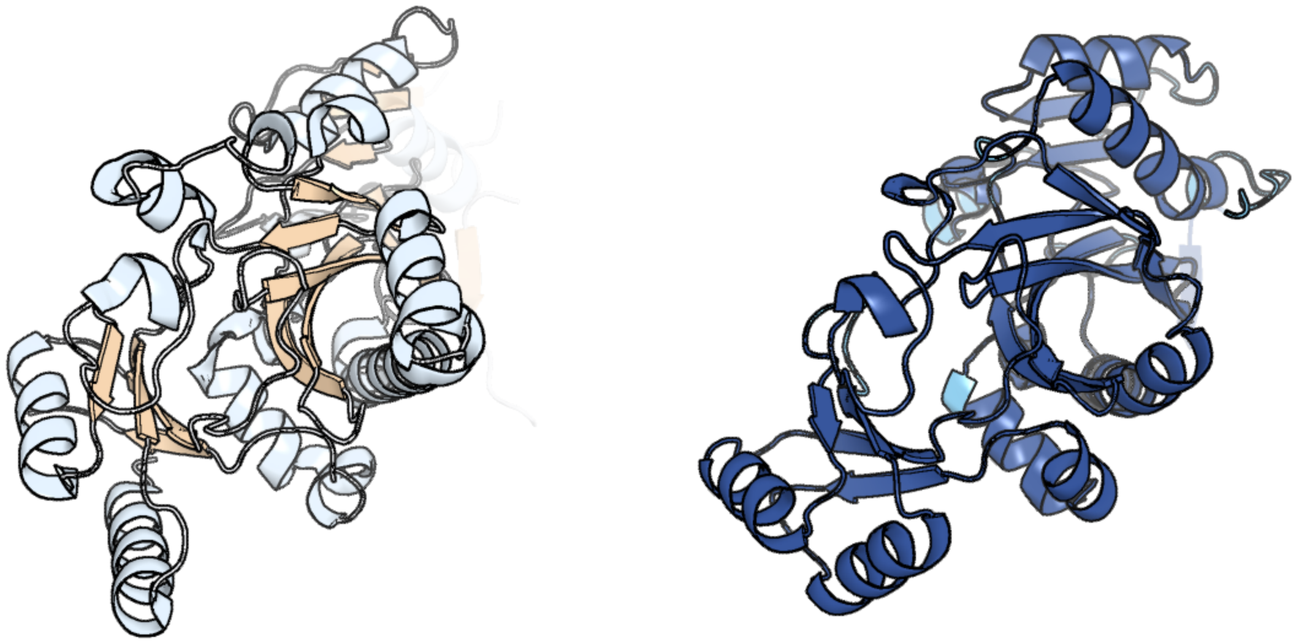

Figure 43: left: reference structure of 8asn chain G. right: predicted structure of chlorv-1..043, unaligned sequences are shown as transparent

chlorv-1..044

- Sequence-based annotation for chlorv-1..044 is hypothetical protein
- Best hit was 4n4a chain A: Cap-specific mRNA (nucleoside-2'-O-)-methyltransferase 1

target	prob	fident	alnlen	evalue	theader
4n4a-assembly1.cif.gz_A	1	0.118	370	1.193e-08	Cystal structure of Cap-specific mRNA (nucleoside-2'-O-)-methyltransferase 1
4n49-assembly1.cif.gz_A	1	0.114	374	2.444e-07	Cap-specific mRNA (nucleoside-2'-O-)-methyltransferase 1 Protein in complex with m7GpppG and SAM
4n48-assembly1.cif.gz_B	1	0.12	375	3.918e-07	Cap-specific mRNA (nucleoside-2'-O-)-methyltransferase 1 Protein in complex with capped RNA fragment

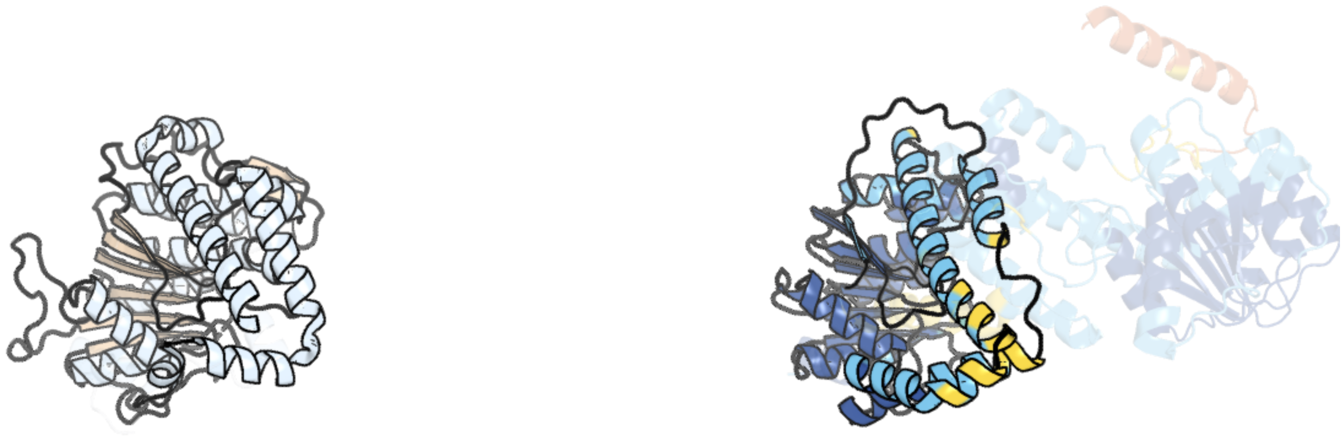

Figure 44: left: reference structure of 4n4a chain A. right: predicted structure of chlorv-1..044, unaligned sequences are shown as transparent

chlorv-1..045

- Sequence-based annotation for chlorv-1..045 is hypothetical protein
- No significant structural hit found

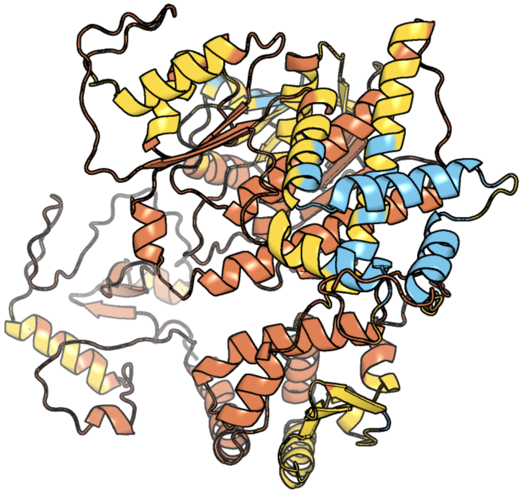

Figure 45: predicted structure of chlorv-1..045

chlorv-1..046

- Sequence-based annotation for chlorv-1..046 is putative Kinase
- Best hit was 1del chain A: DEOXYNUCLEOSIDE MONOPHOSPHATE KINASE

target	prob	fident	alnlen	evalue	theader
1del-assembly1.cif.gz_A	1	0.197	243	1.807e-07	DEOXYNUCLEOSIDE MONOPHOSPHATE KINASE COMPLEXED WITH DEOXY-GMP AND AMP
2grj-assembly2.cif.gz_B	1	0.177	197	5.028e-06	Crystal structure of Dephospho-CoA kinase (EC 2.7.1.24) (Dephosphocoenzyme A kinase) (tm1387) from THERMOTOGA MARITIMA at 2.60 A resolution
1del-assembly1.cif.gz_B	1	0.16	256	2.737e-05	DEOXYNUCLEOSIDE MONOPHOSPHATE KINASE COMPLEXED WITH DEOXY-GMP AND AMP

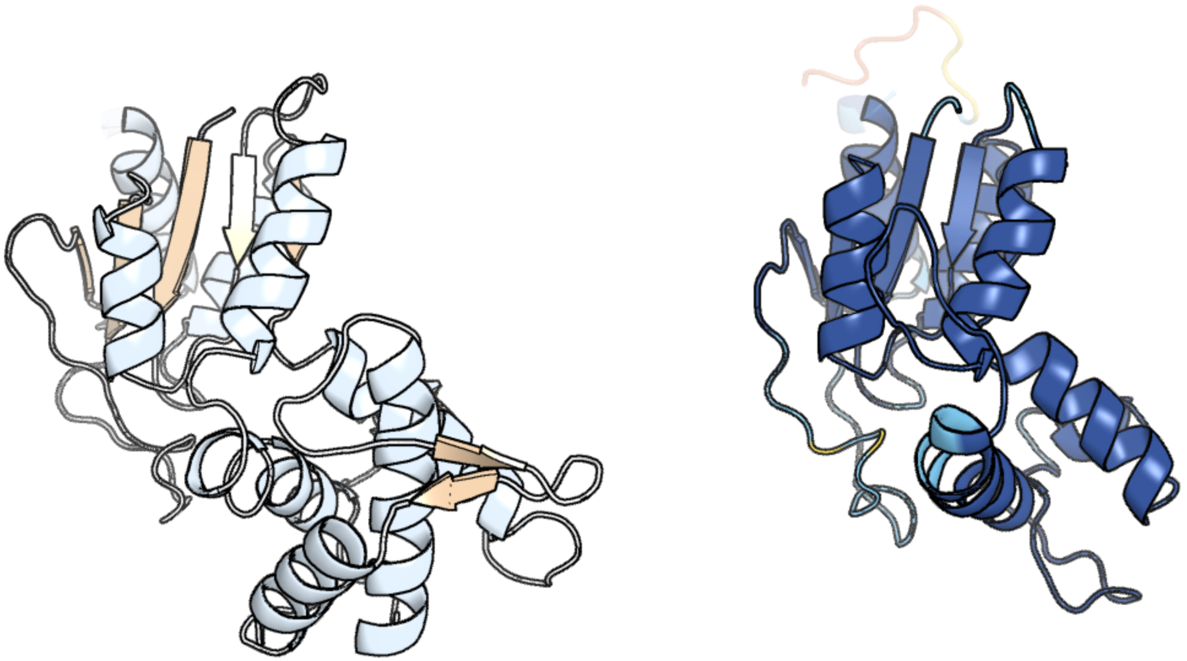

Figure 46: left: reference structure of 1del chain A. right: predicted structure of chlorv-1..046, unaligned sequences are shown as transparent

chlorv-1..047

- Sequence-based annotation for chlorv-1..047 is putative Protein kinase
- Best hit was 5ezv chain A: 5'-AMP-activated protein kinase catalytic subunit alpha-2/alpha-1 RIM SWAP chimera

target	prob	fident	alnlen	evalue	theader
5ezv-assembly1.cif.gz_A	1	0.216	332	6.039e-16	X-ray crystal structure of AMP-activated protein kinase alpha-2/alpha-1 RIM chimaera (alpha-2(1-347)/alpha-1(349-401)/alpha-2(397-end) beta-1 gamma-1) co-crystallized with C2 (5-(5-hydroxyl-isoxazol-3-yl)-furan-2-phosphonic acid)
3iec-assembly4.cif.gz_D	1	0.227	321	6.349e-16	Helicobacter pylori CagA Inhibits PAR1/MARK Family Kinases by Mimicking Host Substrates
6c9d-assembly1.cif.gz_A	1	0.236	322	1.218e-15	Crystal structure of KA1-autoinhibited MARK1 kinase

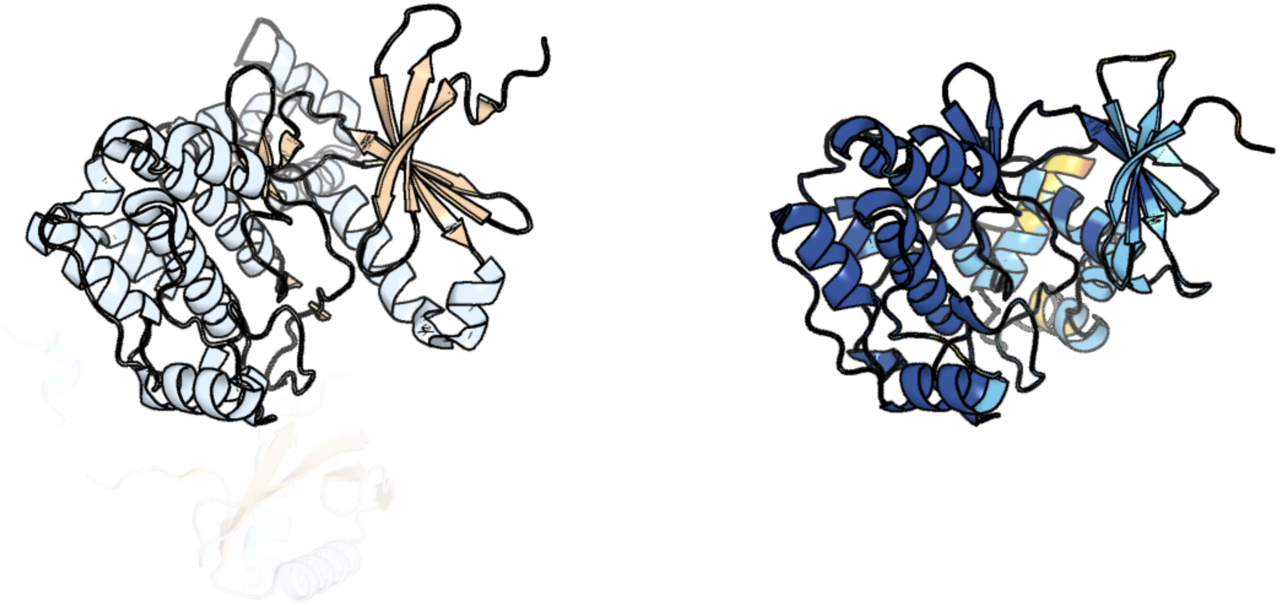

Figure 47: left: reference structure of 5ezv chain A. right: predicted structure of chlorv-1..047, unaligned sequences are shown as transparent

chlorv-1..048

- Sequence-based annotation for chlorv-1..048 is hypothetical protein
- No significant structural hit found

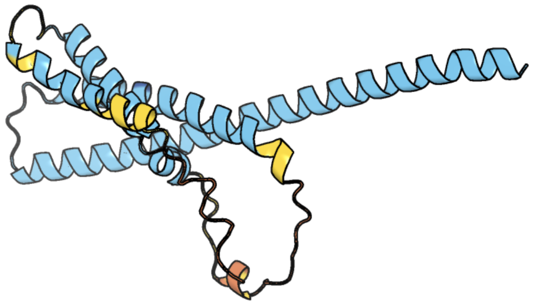

Figure 48: predicted structure of chlorv-1..048

chlorv-1..049

- Sequence-based annotation for chlorv-1..049 is putative dUTPase
- Best hit was 3t64 chain A: Deoxyuridine 5'-triphosphate nucleotidohydrolase, putative

target	prob	fident	alnlen	evalue	theader
3t64-assembly2.cif.gz_A	1	0.311	183	1.075e-12	5'-Diphenyl Nucleoside Inhibitors of Plasmodium falciparum dUTPase
1vyq-assembly1.cif.gz_A	1	0.307	166	1.724e-12	Novel inhibitors of Plasmodium Falciparum dUTPase provide a platform for anti-malarial drug design
3t64-assembly2.cif.gz_C	1	0.331	178	3.3e-12	5'-Diphenyl Nucleoside Inhibitors of Plasmodium falciparum dUTPase

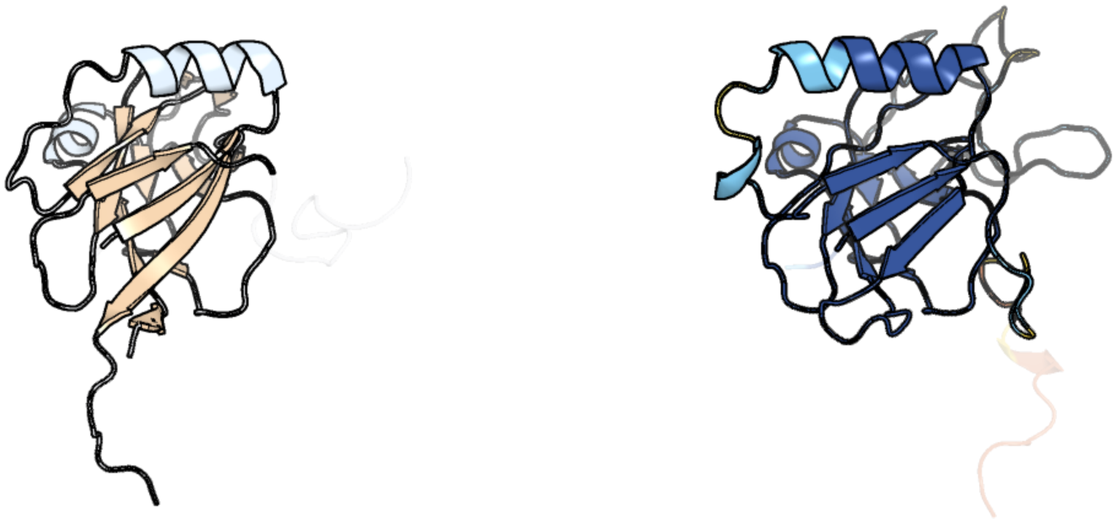

Figure 49: left: reference structure of 3t64 chain A. right: predicted structure of chlorv-1..049, unaligned sequences are shown as transparent

chlorv-1..050

- Sequence-based annotation for chlorv-1..050 is hypothetical protein
- No significant structural hit found

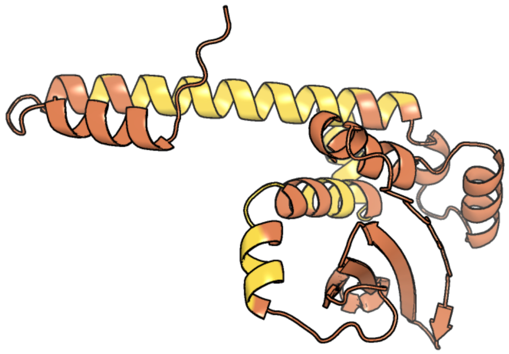

Figure 50: predicted structure of chlorv-1..050

chlorv-1..051

- Sequence-based annotation for chlorv-1..051 is hypothetical protein
- No significant structural hit found

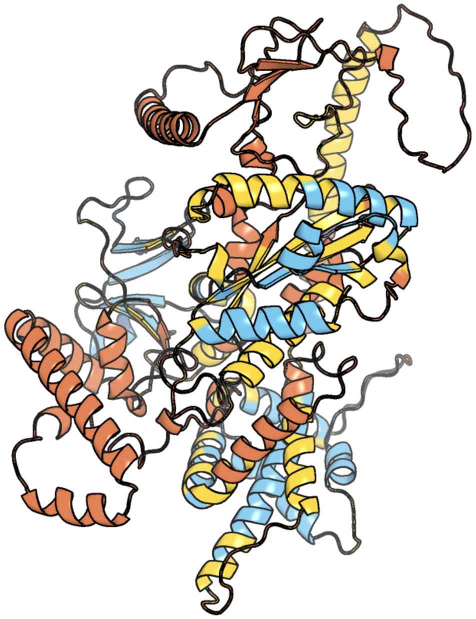

Figure 51: predicted structure of chlorv-1..051

chlorv-1..052

- Sequence-based annotation for chlorv-1..052 is putative Methyltransferase
- Best hit was 4n48 chain B: Cap-specific mRNA (nucleoside-2'-O-)-methyltransferase 1

target	prob	fident	alnlen	evalue	theader
4n48-assembly1.cif.gz_B	1	0.145	418	1.196e-10	Cap-specific mRNA (nucleoside-2'-O-)-methyltransferase 1 Protein in complex with capped RNA fragment
4n48-assembly2.cif.gz_A	1	0.146	409	1.314e-10	Cap-specific mRNA (nucleoside-2'-O-)-methyltransferase 1 Protein in complex with capped RNA fragment
4n49-assembly1.cif.gz_A	1	0.154	415	1.662e-10	Cap-specific mRNA (nucleoside-2'-O-)-methyltransferase 1 Protein in complex with m7GpppG and SAM

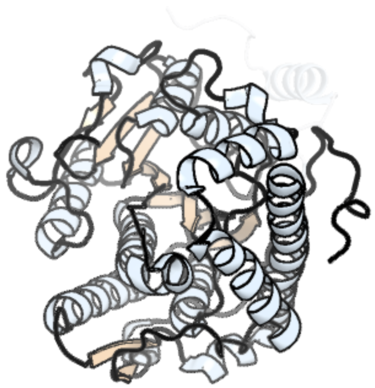

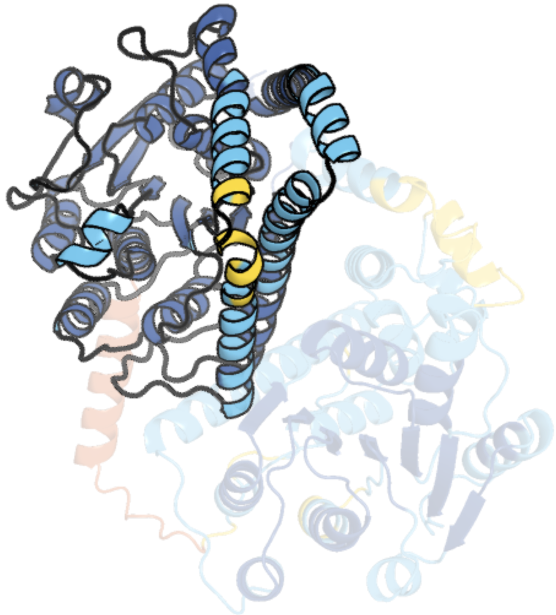

Figure 52: left: reference structure of 4n48 chain B. right: predicted structure of chlorv-1..052, unaligned sequences are shown as transparent

chlorv-1..053

- Sequence-based annotation for chlorv-1..053 is putative Methyltransferase
- Best hit was 4n48 chain A: Cap-specific mRNA (nucleoside-2'-O-)-methyltransferase 1

target	prob	fident	alnlen	evalue	theader
4n48-assembly2.cif.gz_A	1	0.137	416	1.075e-09	Cap-specific mRNA (nucleoside-2'-O-)-methyltransferase 1 Protein in complex with capped RNA fragment
4n48-assembly1.cif.gz_B	1	0.14	413	1.075e-09	Cap-specific mRNA (nucleoside-2'-O-)-methyltransferase 1 Protein in complex with capped RNA fragment
4n4a-assembly1.cif.gz_A	1	0.139	401	1.348e-09	Cystal structure of Cap-specific mRNA (nucleoside-2'-O-)-methyltransferase 1

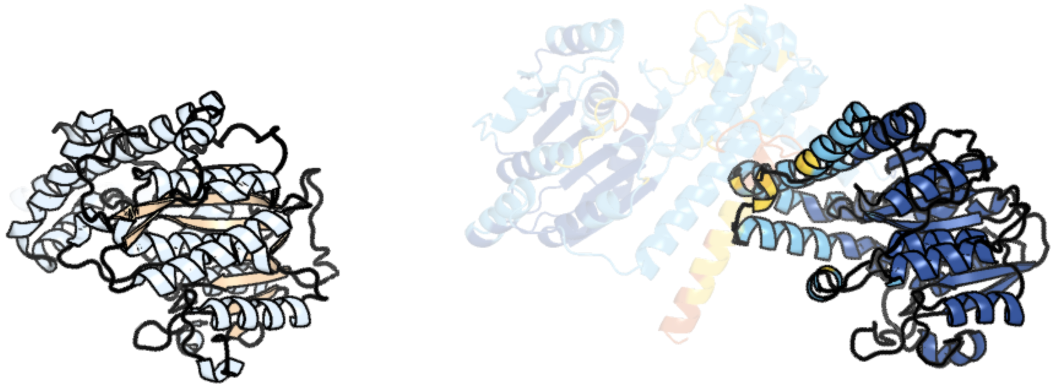

Figure 53: left: reference structure of 4n48 chain A. right: predicted structure of chlorv-1..053, unaligned sequences are shown as transparent

chlorv-1..054

- Sequence-based annotation for chlorv-1..054 is putative Protein kinase
- Best hit was 3ckx chain A: Serine/threonine-protein kinase 24

target	prob	fident	alnlen	evalue	theader
3ckx-assembly1.cif.gz_A	1	0.269	252	3.415e-20	Crystal structure of sterile 20-like kinase 3 (MST3, STK24) in complex with staurosporine
4zy4-assembly1.cif.gz_A	1	0.274	237	3.622e-20	Crystal structure of P21 activated kinase 1 in complex with an inhibitor compound 4
5dew-assembly1.cif.gz_A	1	0.256	242	4.858e-20	Crystal structure of PAK1 in complex with an inhibitor compound 5

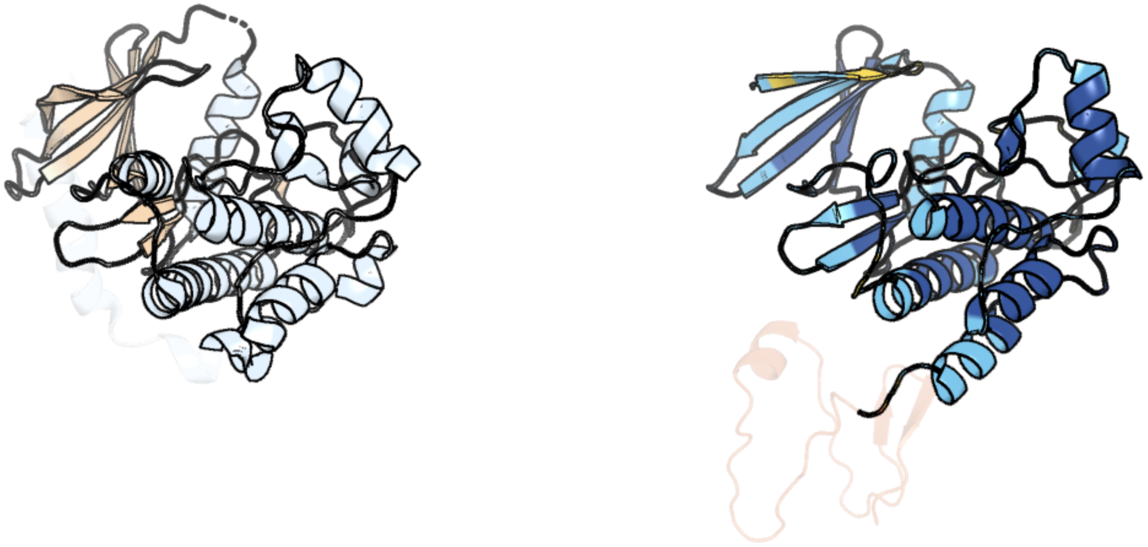

Figure 54: left: reference structure of 3ckx chain A. right: predicted structure of chlorv-1..054, unaligned sequences are shown as transparent

chlorv-1..055

- Sequence-based annotation for chlorv-1..055 is hypothetical protein
- No significant structural hit found

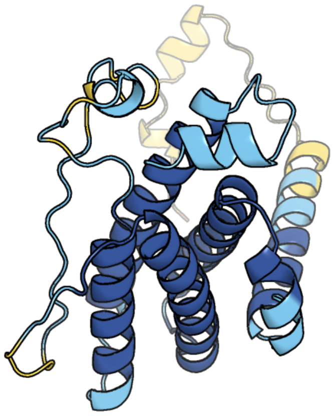

Figure 55: predicted structure of chlorv-1..055

chlorv-1..056

- Sequence-based annotation for chlorv-1..056 is hypothetical protein
- No significant structural hit found

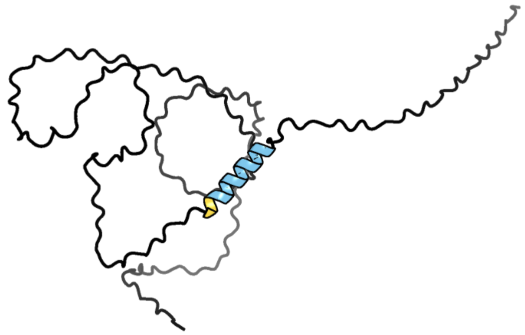

Figure 56: predicted structure of chlorv-1..056

chlorv-1..057

- Sequence-based annotation for chlorv-1..057 is hypothetical protein
- No significant structural hit found

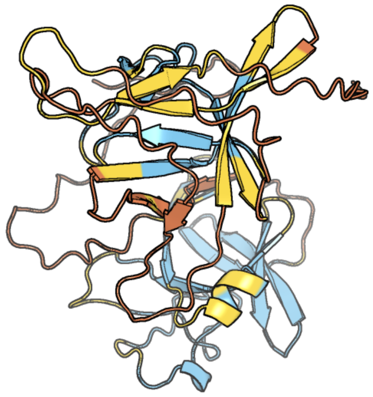

Figure 57: predicted structure of chlorv-1..057

chlorv-1..058

- Sequence-based annotation for chlorv-1..058 is hypothetical protein
- No significant structural hit found

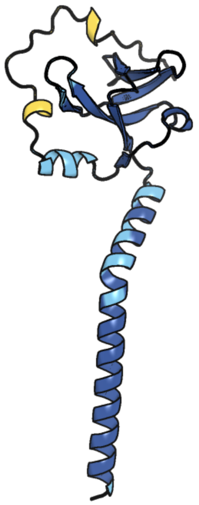

Figure 58: predicted structure of chlorv-1..058

chlorv-1..059

- Sequence-based annotation for chlorv-1..059 is putative Ribonucleotide reductase small subunit
- Best hit was 3vpm chain B: Ribonucleoside-diphosphate reductase subunit M2

target	prob	fident	alnlen	evalue	theader
3vpm-assembly1.cif.gz_B	1	0.516	281	5.282e-23	Crystal structure of human ribonucleotide reductase subunit M2 (hRRM2) mutant
3vpo-assembly1.cif.gz_B	1	0.516	281	7.401e-23	Crystal structure of human ribonucleotide reductase subunit M2 (hRRM2) mutant
3olj-assembly1.cif.gz_D	1	0.519	281	1.28e-22	Crystal structure of human ribonucleotide reductase subunit M2 (hRRM2)

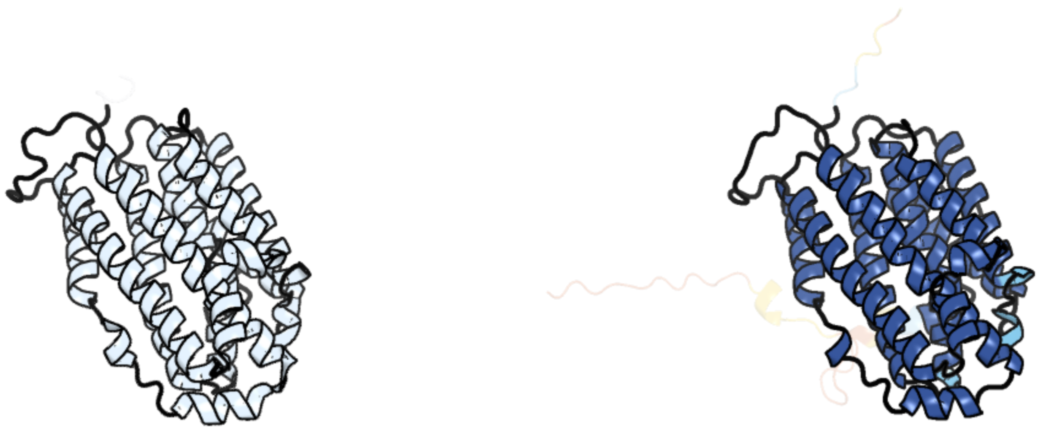

Figure 59: left: reference structure of 3vpm chain B. right: predicted structure of chlorv-1..059, unaligned sequences are shown as transparent

chlorv-1..060

- Sequence-based annotation for chlorv-1..060 is hypothetical protein
- No significant structural hit found

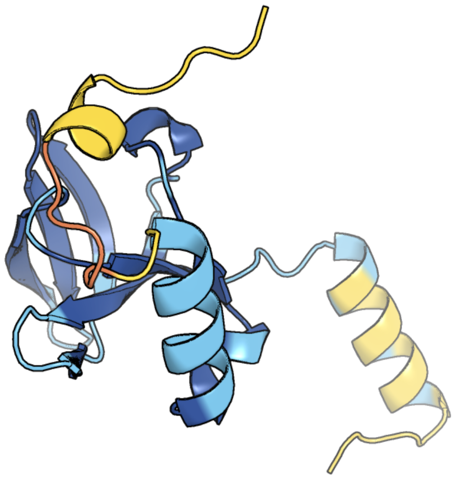

Figure 60: predicted structure of chlorv-1..060

chlorv-1..061

- Sequence-based annotation for chlorv-1..061 is hypothetical protein
- Best hit was 6u05 chain A: tRNA ligase

target	prob	fident	alnlen	evalue	theader
6u05-assembly1.cif.gz_A	1	0.126	449	1.103e-08	Crystal Structure of Fungal RNA Kinase
4jt2-assembly1.cif.gz_A	1	0.257	140	9.69e-07	Structure of Clostridium thermocellum polynucleotide kinase bound to CTP
4gp6-assembly1.cif.gz_B-2	1	0.25	140	1.154e-06	Polynucleotide kinase

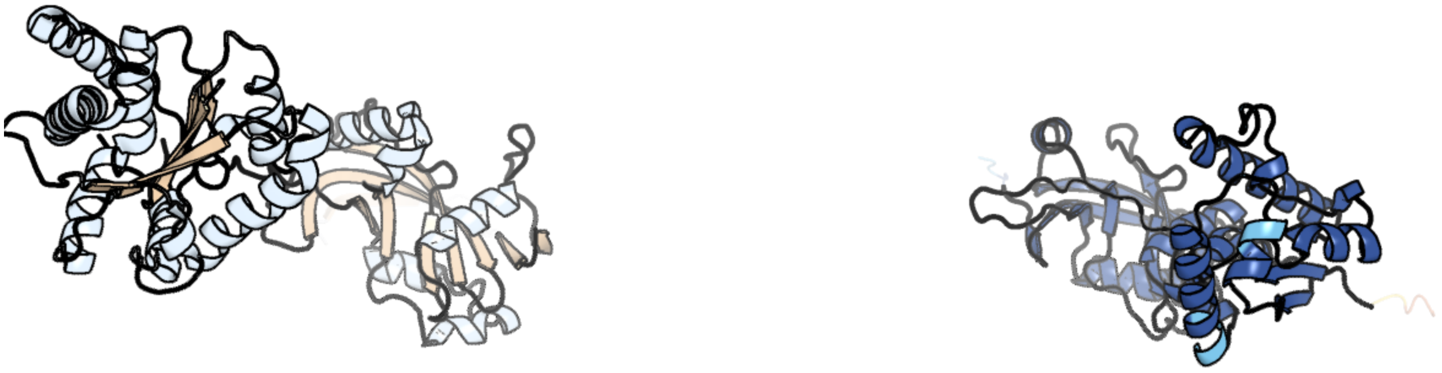

Figure 61: left: reference structure of 6u05 chain A. right: predicted structure of chlorv-1..061, unaligned sequences are shown as transparent

chlorv-1..062

- Sequence-based annotation for chlorv-1..062 is hypothetical protein
- No significant structural hit found

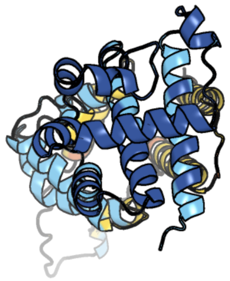

Figure 62: predicted structure of chlorv-1..062

chlorv-1..063

- Sequence-based annotation for chlorv-1..063 is hypothetical protein
- No significant structural hit found

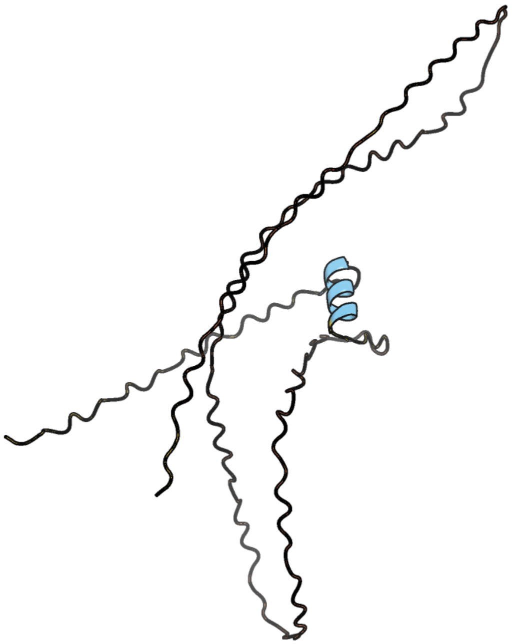

Figure 63: predicted structure of chlorv-1..063

chlorv-1..064

- Sequence-based annotation for chlorv-1..064 is putative eukaryotic translation initiation factor 5
- Best hit was 6fyx chain m: Eukaryotic translation initiation factor 5

target	prob	fident	alnlen	evalue	theader
6fyx-assembly1.cif.gz_m	1	0.307	140	5.861e-10	Structure of a partial yeast 48S preinitiation complex with eIF5 N-terminal domain (model C1)
8cas-assembly1.cif.gz_m	1	0.285	140	1.178e-09	Cryo-EM structure of native Otu2-bound ubiquitinated 48S initiation complex (partial)
2e9h-assembly1.cif.gz_A	1	0.274	142	1.249e-09	Solution structure of the eIF-5_eIF-2B domain from human Eukaryotic translation initiation factor 5

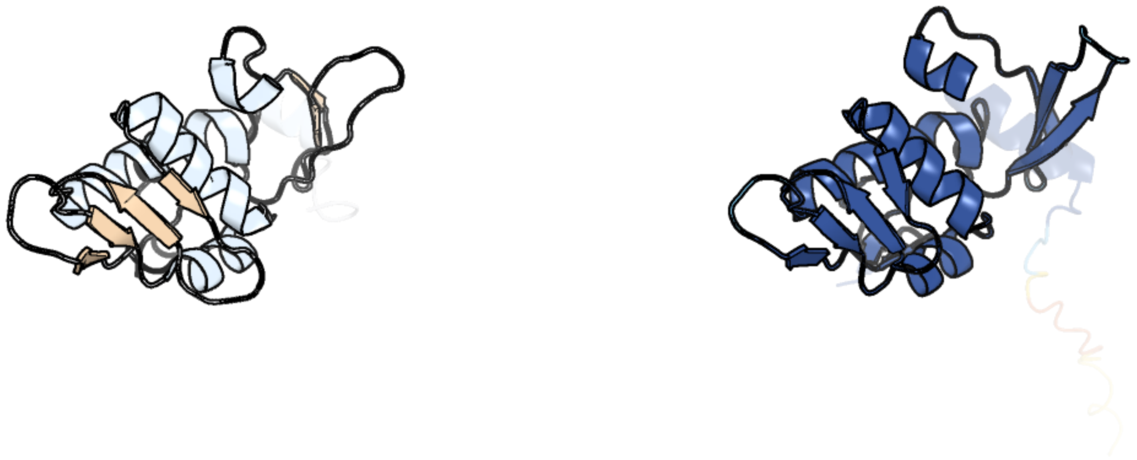

Figure 64: left: reference structure of 6fyx chain m. right: predicted structure of chlorv-1..064, unaligned sequences are shown as transparent

chlorv-1..065

- Sequence-based annotation for chlorv-1..065 is putative DNA polymerase family B
- Best hit was 6p1h chain A: DNA polymerase delta catalytic subunit

target	prob	fident	alnlen	evalue	theader
6p1h-assembly1.cif.gz_A	1	0.197	1330	4.259e-56	Cryo-EM Structure of DNA Polymerase Delta Holoenzyme
3iay-assembly1.cif.gz_A	1	0.205	1311	4.399e-55	Ternary complex of DNA polymerase delta
7kc0-assembly1.cif.gz_A	1	0.201	1313	1.189e-51	Structure of the Saccharomyces cerevisiae replicative polymerase delta in complex with a primer/template and the PCNA clamp

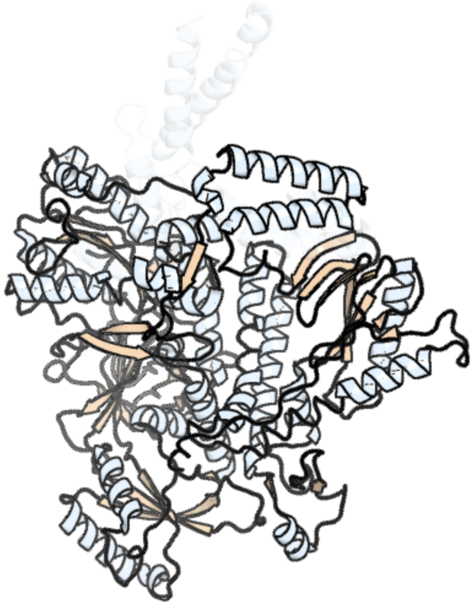

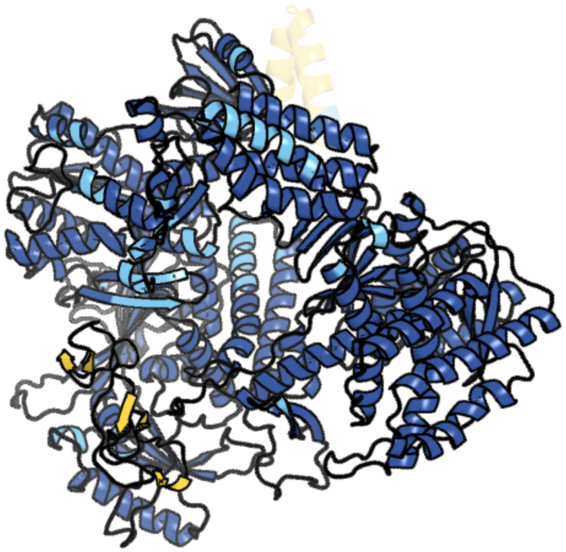

Figure 65: left: reference structure of 6p1h chain A. right: predicted structure of chlorv-1..065, unaligned sequences are shown as transparent

chlorv-1..066

- Sequence-based annotation for chlorv-1..066 is hypothetical protein
- No significant structural hit found

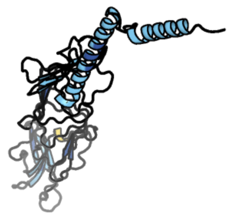

Figure 66: predicted structure of chlorv-1..066

chlorv-1..067

- Sequence-based annotation for chlorv-1..067 is putative Protein kinase
- No significant structural hit found

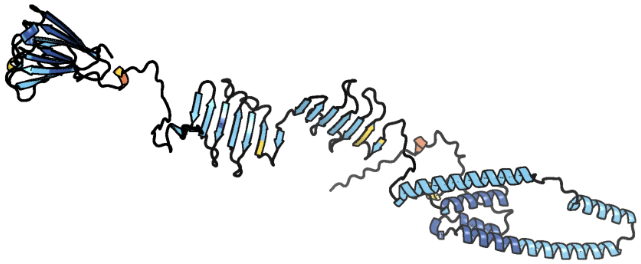

Figure 67: predicted structure of chlorv-1..067

chlorv-1..068

- Sequence-based annotation for chlorv-1..068 is putative Arginase
- Best hit was 4q3r chain D: Arginase

target	prob	fident	alnlen	evalue	theader
4q3r-assembly4.cif.gz_D	1	0.284	299	3.526e-22	Crystal structure of Schistosoma mansoni arginase in complex with inhibitor ABHDP
4q3v-assembly3.cif.gz_C	1	0.277	303	3.734e-22	Crystal structure of Schistosoma mansoni arginase in complex with inhibitor BEC
4q3v-assembly1.cif.gz_A	1	0.284	302	4.698e-22	Crystal structure of Schistosoma mansoni arginase in complex with inhibitor BEC

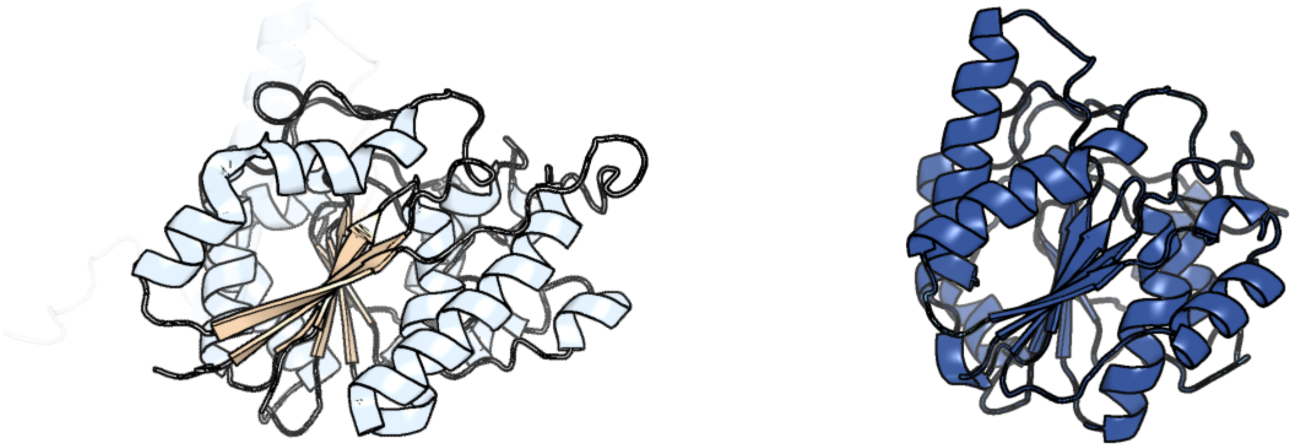

Figure 68: left: reference structure of 4q3r chain D. right: predicted structure of chlorv-1..068, unaligned sequences are shown as transparent

chlorv-1..069

- Sequence-based annotation for chlorv-1..069 is putative Ankyrin repeat protein
- Best hit was 5op1 chain A: DARPin A4

target	prob	fident	alnlen	evalue	theader
5op1-assembly1.cif.gz_A	1	0.205	170	2.926e-07	Designed Ankyrin Repeat Protein (DARPin) A4 in complex with Lysozyme
6h46-assembly1.cif.gz_B	1	0.195	174	3.791e-07	Human KRAS in complex with darpin K13
4hna-assembly1.cif.gz_D	1	0.184	179	6.042e-07	Kinesin motor domain in the ADP-MG-ALFX state in complex with tubulin and a DARPIN

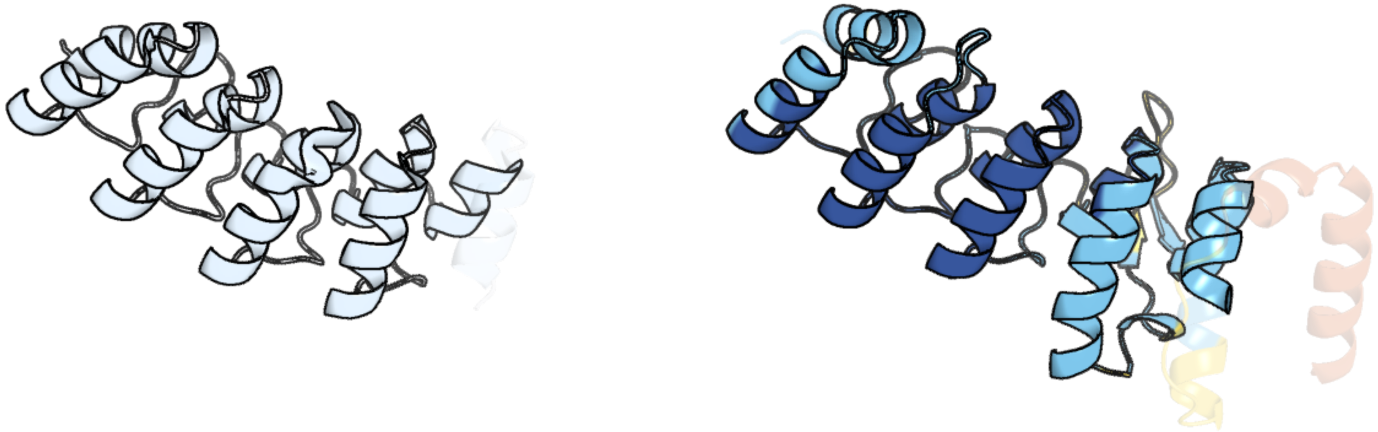

Figure 69: left: reference structure of 5op1 chain A. right: predicted structure of chlorv-1..069, unaligned sequences are shown as transparent

chlorv-1..070

- Sequence-based annotation for chlorv-1..070 is hypothetical protein
- No significant structural hit found

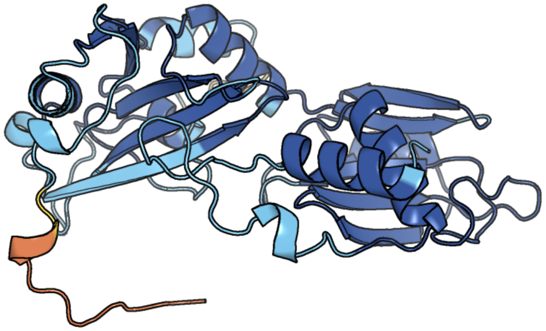

Figure 70: predicted structure of chlorv-1..070

chlorv-1..071

- Sequence-based annotation for chlorv-1..071 is putative Ankyrin repeat protein
- Best hit was 1n11 chain A: Ankyrin

target	prob	fident	alnlen	evalue	theader
8cs9-assembly1.cif.gz_A	1	0.126	459	1.177e-06	Composite reconstruction of Class 1 of the erythrocyte ankyrin-1 complex
7v0x-assembly1.cif.gz_J	1	0.115	397	2.308e-06	Local refinement of ankyrin-1 (C-terminal half), class 1 of erythrocyte ankyrin-1 complex
1n11-assembly1.cif.gz_A	1	0.106	402	4.11e-06	D34 REGION OF HUMAN ANKYRIN-R AND LINKER

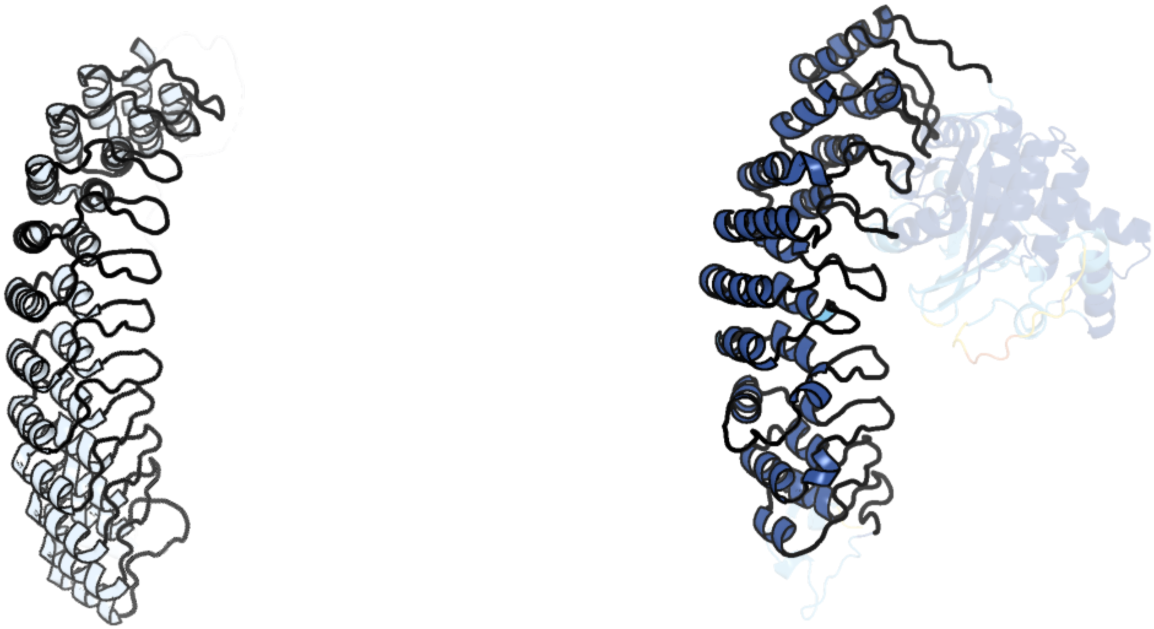

Figure 71: left: reference structure of 1n11 chain A. right: predicted structure of chlorv-1..071, unaligned sequences are shown as transparent

chlorv-1..072

- Sequence-based annotation for chlorv-1..072 is hypothetical protein
- Best hit was 8ppl chain Iq: Eukaryotic translation initiation factor 1A, X-chromosomal

target	prob	fident	alnlen	evalue	theader
8ppl-assembly1.cif.gz_Iq	1	0.232	112	3.688e-06	MERS-CoV Nsp1 bound to the human 43S pre-initiation complex
6fyy-assembly1.cif.gz_i	1	0.243	111	6.684e-06	Structure of a partial yeast 48S preinitiation complex with eIF5 N-terminal domain (model C2)
2oqk-assembly1.cif.gz_A	1	0.26	100	1.364e-05	Crystal structure of putative Cryptosporidium parvum translation initiation factor eIF-1A

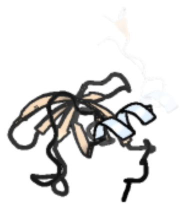

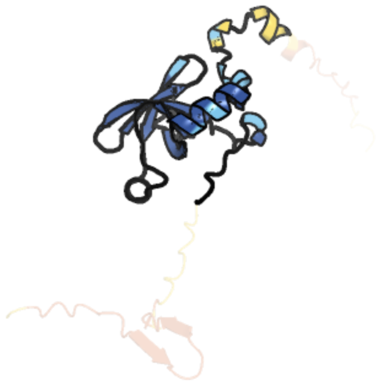

Figure 72: left: reference structure of 8ppl chain Iq. right: predicted structure of chlorv-1..072, unaligned sequences are shown as transparent

chlorv-1..073

- Sequence-based annotation for chlorv-1..073 is hypothetical protein
- No significant structural hit found

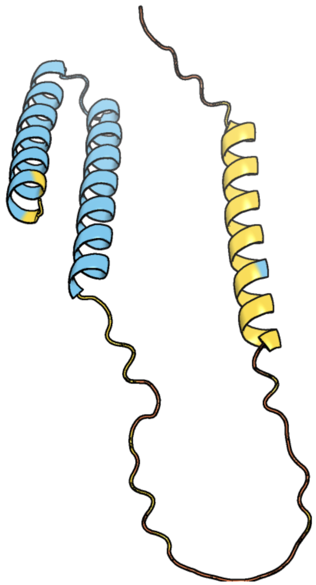

Figure 73: predicted structure of chlorv-1..073

chlorv-1..074

- Sequence-based annotation for chlorv-1..074 is hypothetical protein
- No significant structural hit found

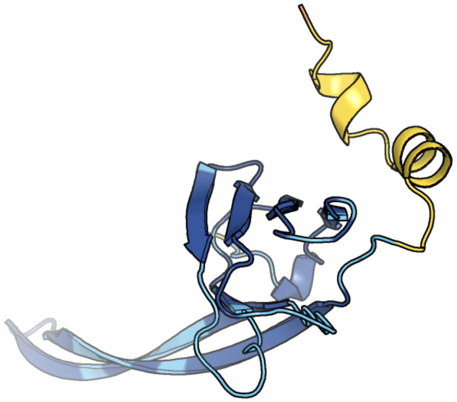

Figure 74: predicted structure of chlorv-1..074

chlorv-1..075

- Sequence-based annotation for chlorv-1..075 is putative Peptidase
- Best hit was 4rgh chain A: Protein DDI1 homolog 2

target	prob	fident	alnlen	evalue	theader
4rgh-assembly1.cif.gz_A	1	0.303	122	4.185e-13	Human DNA Damage-Inducible Protein: From Protein Chemistry and 3D Structure to Deciphering its Cellular Role
4z2z-assembly1.cif.gz_B	1	0.336	122	5.881e-13	New crystal structure of yeast Ddi1 aspartyl protease reveals substrate engagement mode
5yq8-assembly2.cif.gz_D	1	0.333	120	8.938e-12	Crystal structure of retroviral protease-like domain of Ddi1 from Leishmania major

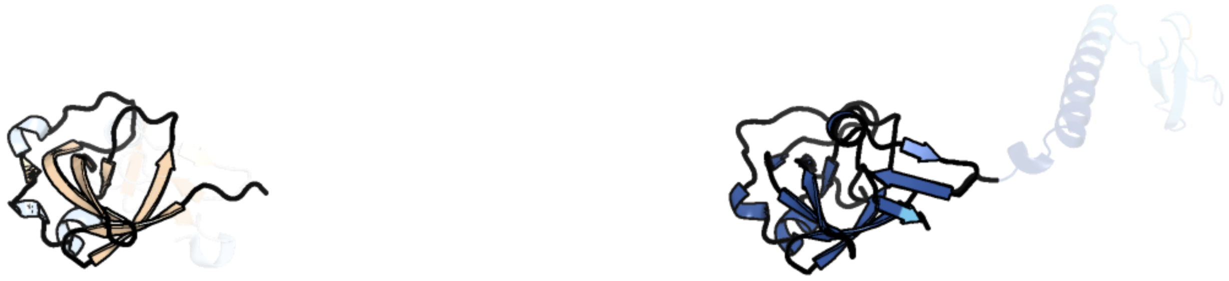

Figure 75: left: reference structure of 4rgh chain A. right: predicted structure of chlorv-1..075, unaligned sequences are shown as transparent

chlorv-1..076

- Sequence-based annotation for chlorv-1..076 is hypothetical protein
- No significant structural hit found

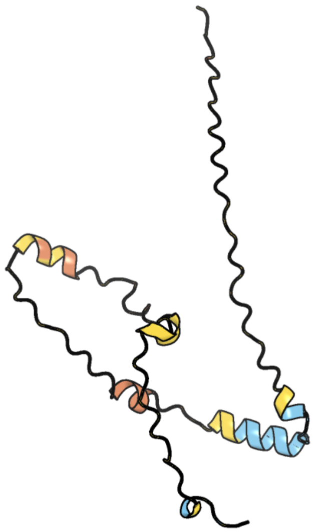

Figure 76: predicted structure of chlorv-1..076

chlorv-1..077

- Sequence-based annotation for chlorv-1..077 is putative Ubiquitin Conjugating Enzyme
- Best hit was 3ceg chain A: Baculoviral IAP repeat-containing protein 6

target	prob	fident	alnlen	evalue	theader
3ceg-assembly1.cif.gz_A	1	0.385	285	1.257e-23	Crystal structure of the UBC domain of baculoviral IAP repeat-containing protein 6
3ceg-assembly2.cif.gz_B	1	0.383	284	6.597e-23	Crystal structure of the UBC domain of baculoviral IAP repeat-containing protein 6
8gxr-assembly1.cif.gz_A	1	0.266	274	1.517e-15	crystal structure of UBC domain of UBE2O

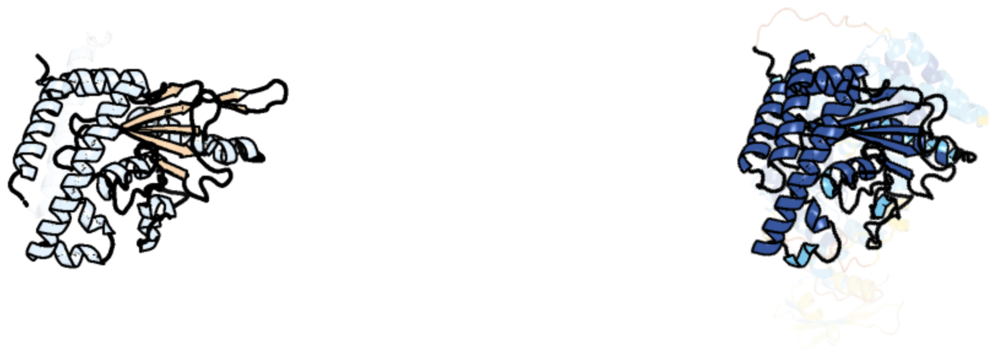

Figure 77: left: reference structure of 3ceg chain A. right: predicted structure of chlorv-1..077, unaligned sequences are shown as transparent

chlorv-1..078

- Sequence-based annotation for chlorv-1..078 is putative Methyltransferase
- Best hit was 3ubt chain B: Modification methylase HaeIII

target	prob	fident	alnlen	evalue	theader
3ubt-assembly3.cif.gz_B	1	0.215	385	6.425e-20	Crystal Structure of C71S Mutant of DNA Cytosine-5 Methyltransferase M.HaeIII Bound to DNA
3g7u-assembly1.cif.gz_A	1	0.185	398	2.36e-19	Crystal structure of putative DNA modification methyltransferase encoded within prophage Cp-933R (E.coli)
1dct-assembly2.cif.gz_B	1	0.223	381	2.671e-19	DNA (CYTOSINE-5) METHYLASE FROM HAEIII COVALENTLY BOUND TO DNA

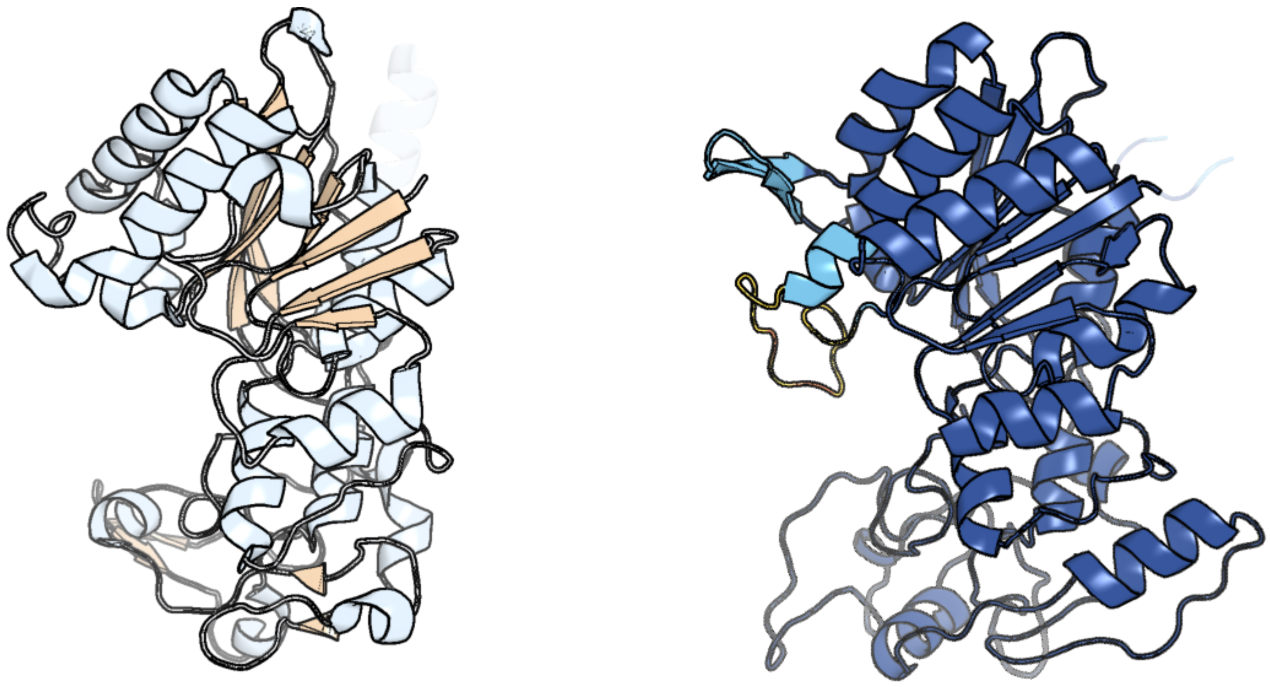

Figure 78: left: reference structure of 3ubt chain B. right: predicted structure of chlorv-1..078, unaligned sequences are shown as transparent

chlorv-1..079

- Sequence-based annotation for chlorv-1..079 is hypothetical protein
- Best hit was 2fkc chain B: R.HinP1I restriction endonuclease

target	prob	fident	alnlen	evalue	theader
2fkc-assembly2.cif.gz_B	1	0.141	283	2.765e-08	Crystal Form I of Pre-Reactive Complex of Restriction Endonuclease HinP1I with Cognate DNA and Calcium Ion
1yfi-assembly2.cif.gz_B	1	0.11	235	5.088e-05	Crystal Structure of restriction endonuclease MspI in complex with its cognate DNA in P212121 space group
4x28-assembly1.cif.gz_B	0.029	0.094	85	0.6603	Crystal structure of the ChsE4-ChsE5 complex from Mycobacterium tuberculosis

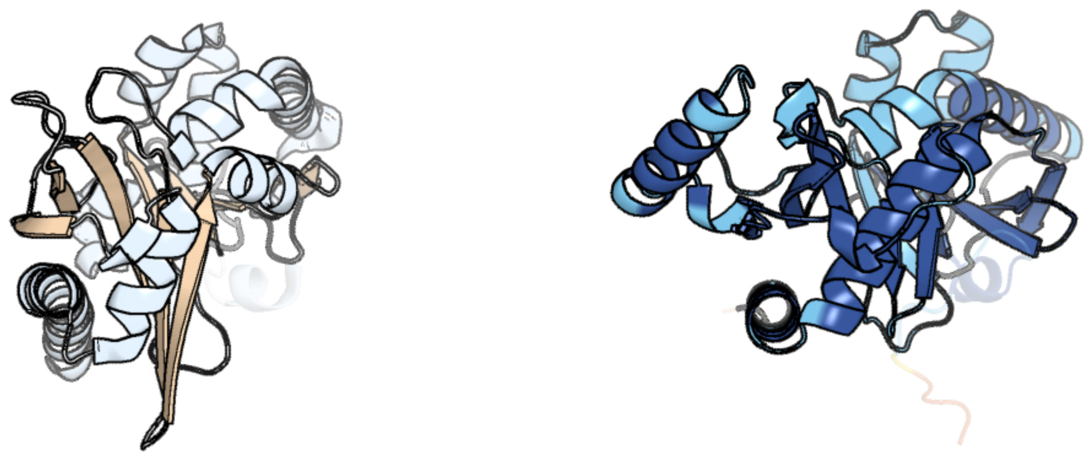

Figure 79: left: reference structure of 2fkc chain B. right: predicted structure of chlorv-1..079, unaligned sequences are shown as transparent

chlorv-1..080

- Sequence-based annotation for chlorv-1..080 is putative ATPase family protein/replication factor C small subunit 2
- Best hit was 8dqx chain C: Replication factor C subunit 3

target	prob	fident	alnlen	evalue	theader
8dqx-assembly1.cif.gz_C	1	0.329	325	2.859e-22	Open state of RFC:PCNA bound to a 3' ss/dsDNA junction
6vvo-assembly1.cif.gz_C	1	0.33	312	1.449e-21	Structure of the human clamp loader (Replication Factor C, RFC) bound to the sliding clamp (Proliferating Cell Nuclear Antigen, PCNA)
1sxj-assembly1.cif.gz_C	1	0.33	321	5.015e-21	Crystal Structure of the Eukaryotic Clamp Loader (Replication Factor C, RFC) Bound to the DNA Sliding Clamp (Proliferating Cell Nuclear Antigen, PCNA)

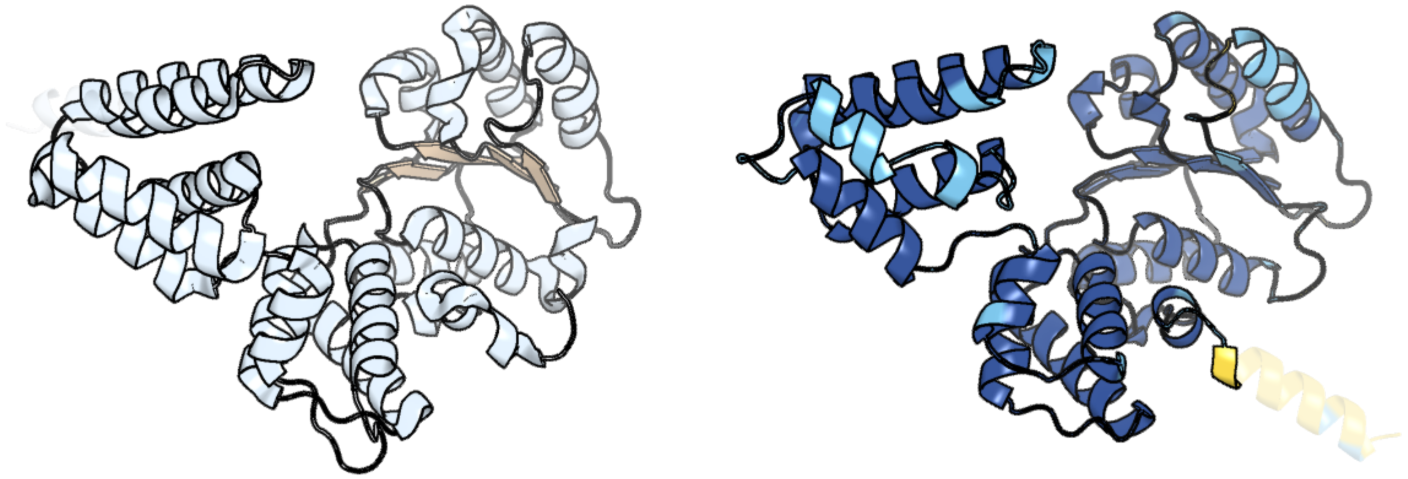

Figure 80: left: reference structure of 8dqx chain C. right: predicted structure of chlorv-1..080, unaligned sequences are shown as transparent

chlorv-1..081

- Sequence-based annotation for chlorv-1..081 is putative Nuclease
- Best hit was 4qmg chain C: Staphylococcal nuclease domain-containing protein 1

target	prob	fident	alnlen	evalue	theader
4qmg-assembly3.cif.gz_C	1	0.191	115	3.611e-06	The Structure of MTDH-SND1 Complex Reveals Novel Cancer-Promoting Interactions
2f0w-assembly1.cif.gz_A	1	0.26	123	5.059e-06	Crystal structure of Staphylococcal nuclease mutant V23I/L25I/V66L/I72L
4qmg-assembly5.cif.gz_E	1	0.189	116	6.701e-06	The Structure of MTDH-SND1 Complex Reveals Novel Cancer-Promoting Interactions

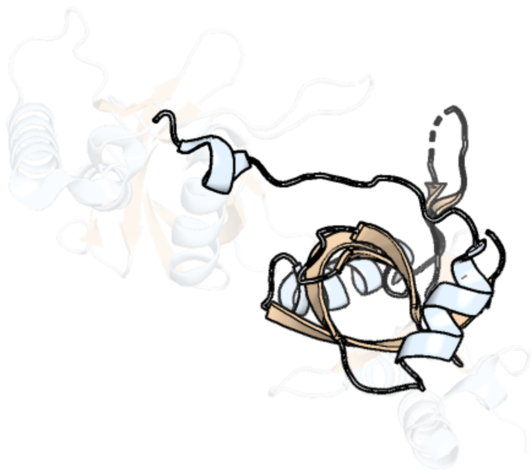

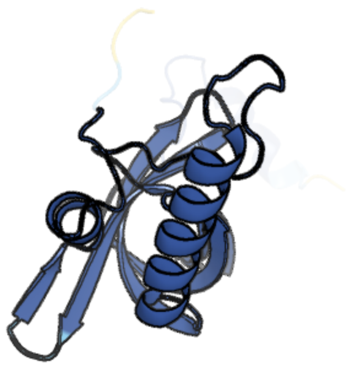

Figure 81: left: reference structure of 4qmg chain C. right: predicted structure of chlorv-1..081, unaligned sequences are shown as transparent

chlorv-1..082

- Sequence-based annotation for chlorv-1..082 is putative Methyltransferases
- Best hit was 1j0a chain A: 1-aminocyclopropane-1-carboxylate deaminase

target	prob	fident	alnlen	evalue	theader
1j0a-assembly1.cif.gz_A	1	0.139	302	5.058e-08	Crystal Structure Analysis of the ACC deaminase homologue
3iau-assembly1.cif.gz_A	1	0.132	279	9.878e-08	The structure of the processed form of threonine deaminase isoform 2 from Solanum lycopersicum
2egu-assembly1.cif.gz_A	1	0.144	276	1.825e-07	Crystal structure of O-acetylserine sulfhydrase from Geobacillus kaustophilus HTA426

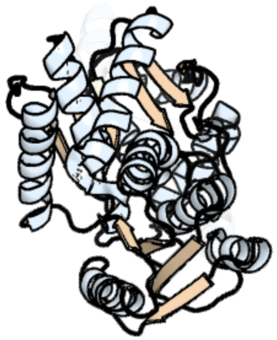

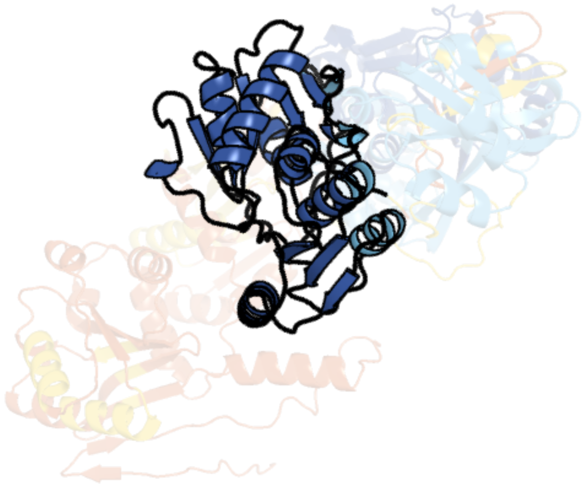

Figure 82: left: reference structure of 1j0a chain A. right: predicted structure of chlorv-1..082, unaligned sequences are shown as transparent

chlorv-1..083

- Sequence-based annotation for chlorv-1..083 is putative Thioredoxin
- No significant structural hit found

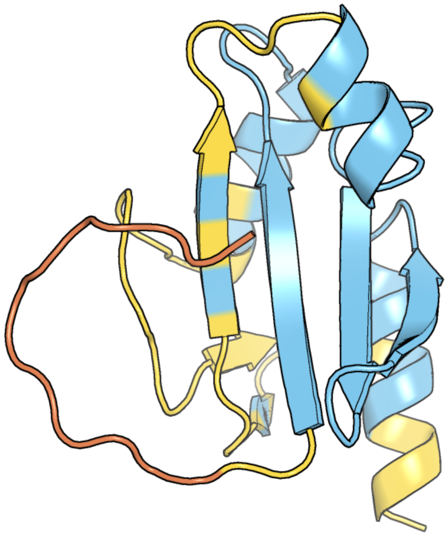

Figure 83: predicted structure of chlorv-1..083

chlorv-1..084

- Sequence-based annotation for chlorv-1..084 is putative Thioredoxin
- Best hit was 2vim chain A: THIOREDOXIN

target	prob	fident	alnlen	evalue	theader
2vim-assembly1.cif.gz_A	1	0.152	105	1.792e-06	X-ray structure of Fasciola hepatica thioredoxin
2f51-assembly2.cif.gz_B	1	0.205	107	3.683e-06	Structure of Trichomonas vaginalis thioredoxin
6z7o-assembly1.cif.gz_A-2	1	0.23	113	4.198e-06	Crystal structure of Thioredoxin T from Drosophila melanogaster

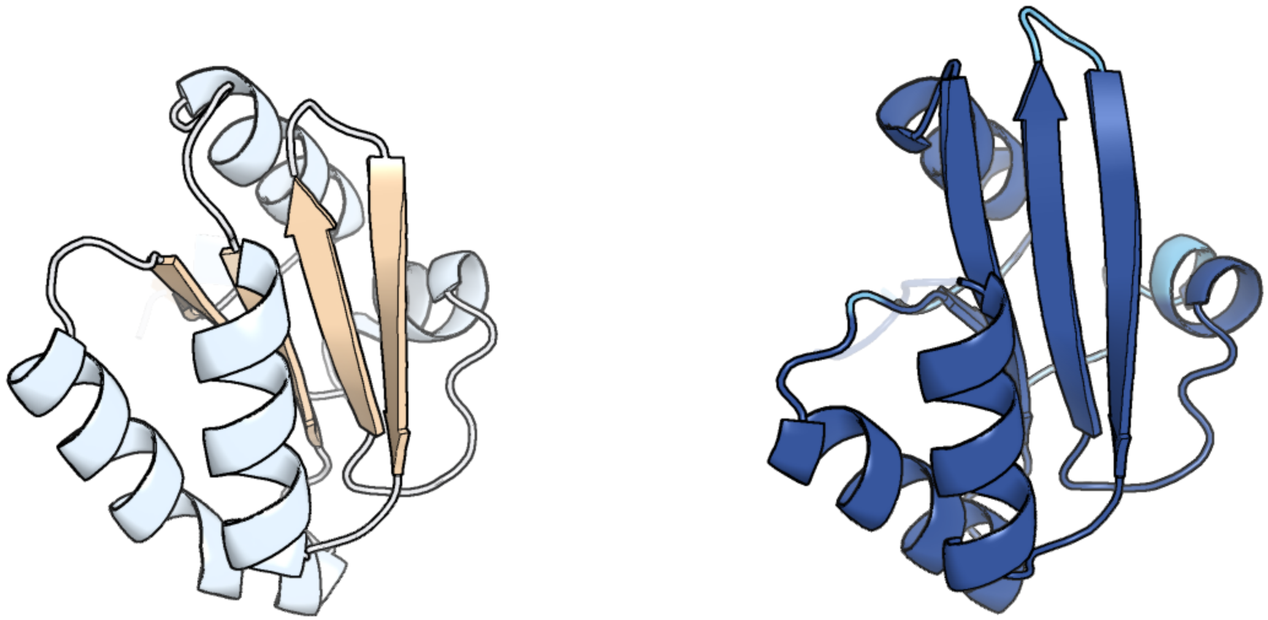

Figure 84: left: reference structure of 2vim chain A. right: predicted structure of chlorv-1..084, unaligned sequences are shown as transparent

chlorv-1..085

- Sequence-based annotation for chlorv-1..085 is hypothetical protein
- No significant structural hit found

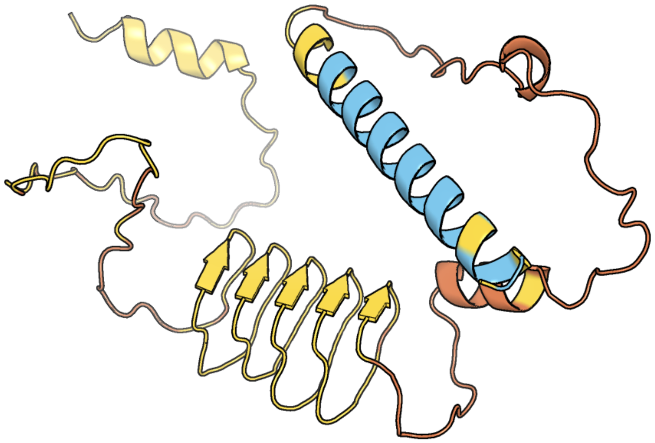

Figure 85: predicted structure of chlorv-1..085

chlorv-1..086

- Sequence-based annotation for chlorv-1..086 is putative Peptidase
- Best hit was 6lj9 chain A: Cysteine protease S273R

target	prob	fident	alnlen	evalue	theader
6lj9-assembly1.cif.gz_A	1	0.24	250	6.465e-14	Crystal Structure of Se-Met ASFV pS273R protease
6ljb-assembly1.cif.gz_A	1	0.226	256	1.219e-13	Crystal Structure of ASFV pS273R protease
6lj9-assembly1.cif.gz_B	1	0.222	252	8.748e-11	Crystal Structure of Se-Met ASFV pS273R protease

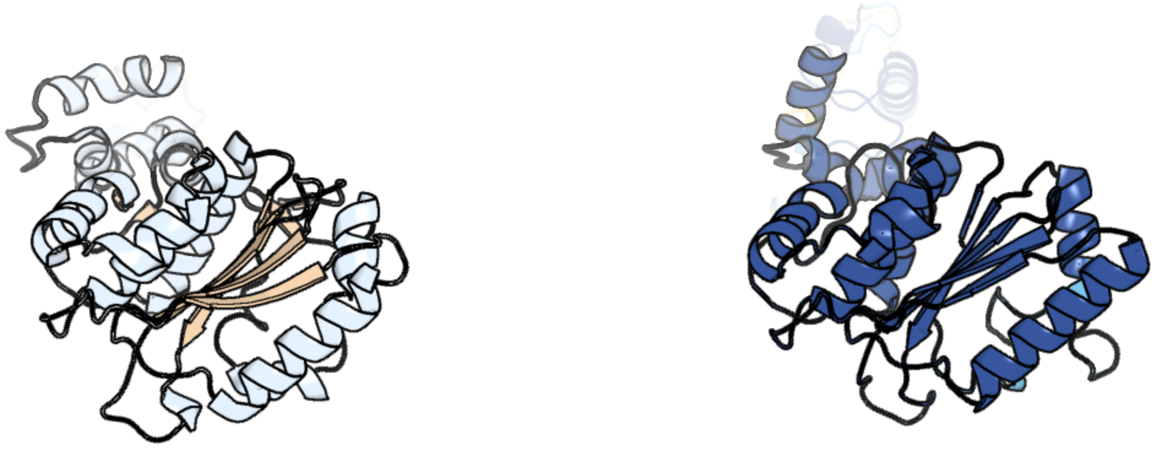

Figure 86: left: reference structure of 6lj9 chain A. right: predicted structure of chlorv-1..086, unaligned sequences are shown as transparent

chlorv-1..087

- Sequence-based annotation for chlorv-1..087 is hypothetical protein
- Best hit was 8h2i chain bG: P1v1

target	prob	fident	alnlen	evalue	theader
8h2i-assembly1.cif.gz_bG	1	0.089	460	7.516e-06	Near-atomic structure of five-fold averaged PBCV-1 capsid
8rbs-assembly1.cif.gz_K	0.993	0.111	268	0.0002393	Emiliania huxleyi virus 201 (EhV-201) asymmetrical unit of capsid proteins predicted by AlphaFold2 fitted into the cryo-EM density of EhV-201 virion composite map.
6yba-assembly1.cif.gz_M	0.992	0.075	398	0.001474	HAdV-F41 Capsid

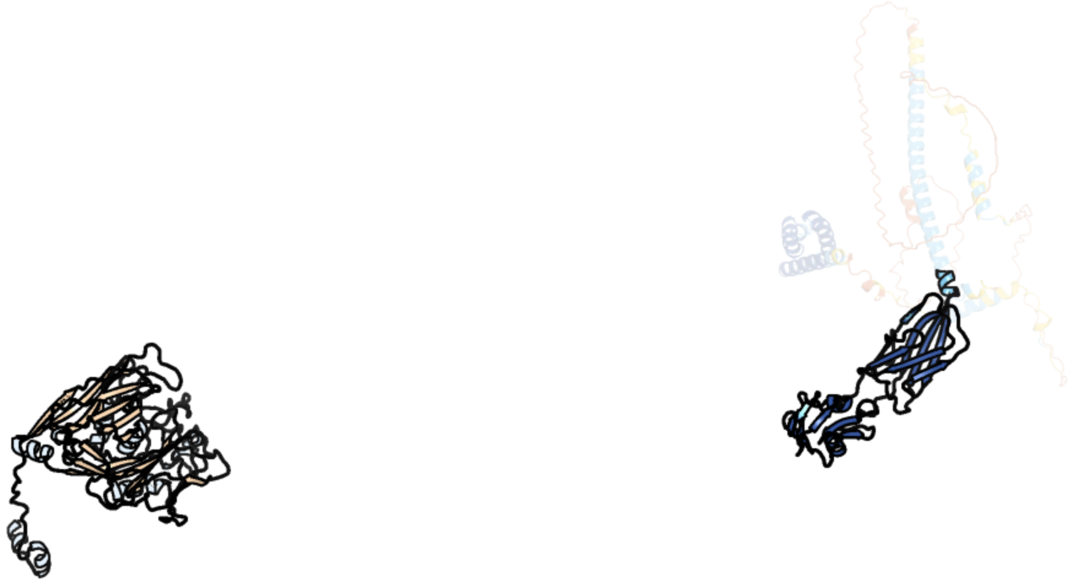

Figure 87: left: reference structure of 8h2i chain bG. right: predicted structure of chlorv-1..087, unaligned sequences are shown as transparent

chlorv-1..088

- Sequence-based annotation for chlorv-1..088 is hypothetical protein
- No significant structural hit found

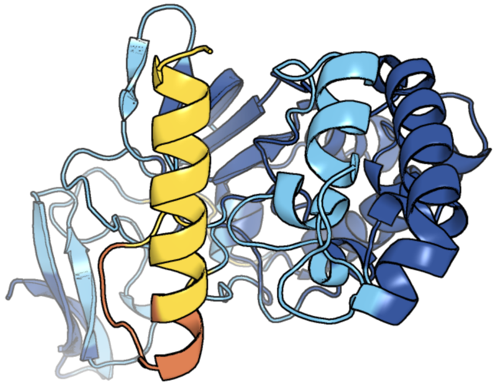

Figure 88: predicted structure of chlorv-1..088

chlorv-1..089

- Sequence-based annotation for chlorv-1..089 is hypothetical protein
- No significant structural hit found

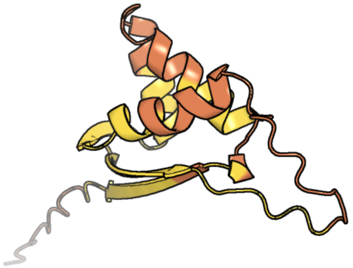

Figure 89: predicted structure of chlorv-1..089

chlorv-1..090

- Sequence-based annotation for chlorv-1..090 is putative GTP binding protein
- Best hit was 5wfs chain z: Elongation factor Tu 2

target	prob	fident	alnlen	evalue	theader
5wfs-assembly1.cif.gz_z	1	0.11	398	1.693e-15	70S ribosome-EF-Tu H84A complex with GTP and near-cognate tRNA (Complex C4)
5we4-assembly1.cif.gz_z	1	0.105	399	1.897e-15	70S ribosome-EF-Tu wt complex with GppNHp
4cxg-assembly1.cif.gz_A	1	0.107	428	6.285e-15	Regulation of the mammalian elongation cycle by 40S subunit rolling: a eukaryotic-specific ribosome rearrangement

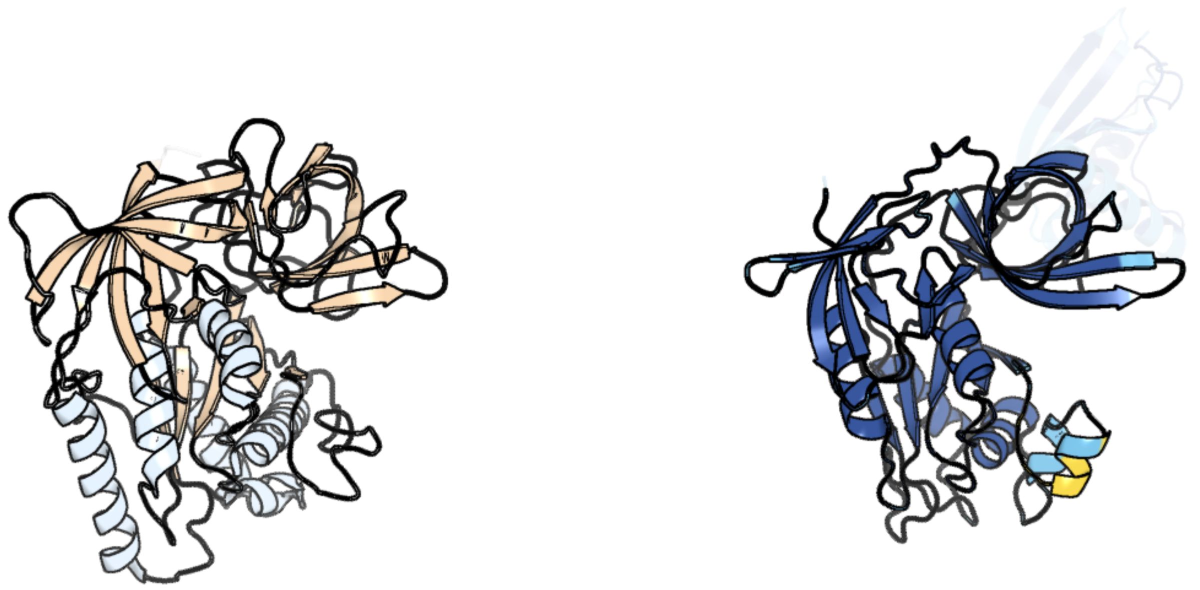

Figure 90: left: reference structure of 5wfs chain z. right: predicted structure of chlorv-1..090, unaligned sequences are shown as transparent

chlorv-1..091

- Sequence-based annotation for chlorv-1..091 is hypothetical protein
- No significant structural hit found

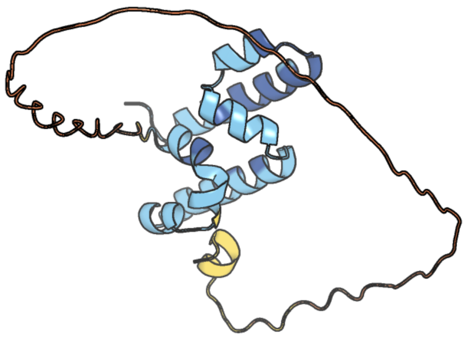

Figure 91: predicted structure of chlorv-1..091

chlorv-1..092

- Sequence-based annotation for chlorv-1..092 is hypothetical protein
- No significant structural hit found

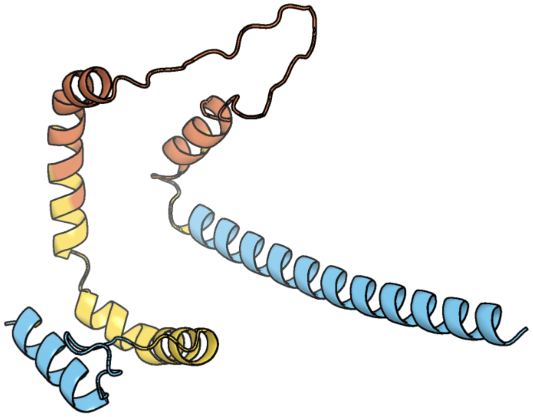

Figure 92: predicted structure of chlorv-1..092

chlorv-1..093

- Sequence-based annotation for chlorv-1..093 is hypothetical protein
- No significant structural hit found

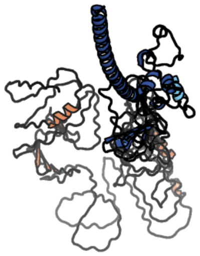

Figure 93: predicted structure of chlorv-1..093

chlorv-1..094

- Sequence-based annotation for chlorv-1..094 is putative Ankyrin repeat protein
- Best hit was 4hqd chain B: Engineered Protein OR265

target	prob	fident	alnlen	evalue	theader
4hqd-assembly2.cif.gz_B	1	0.269	115	9.919e-06	Crystal Structure of Engineered Protein. Northeast Structural Genomics Consortium Target OR265.
7ujj-assembly1.cif.gz_G	1	0.238	113	9.919e-06	Stx2a and DARPin complex
2xzt-assembly2.cif.gz_G	1	0.23	117	1.277e-05	Caspase-3 in Complex with DARPin-3.4_I78S

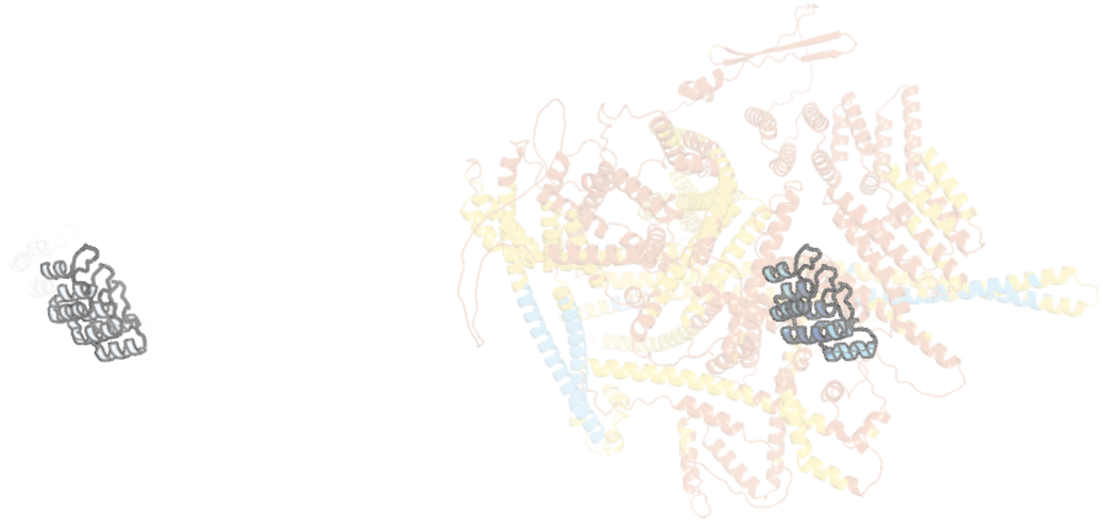

Figure 94: left: reference structure of 4hqd chain B. right: predicted structure of chlorv-1..094, unaligned sequences are shown as transparent

chlorv-1..095

- Sequence-based annotation for chlorv-1..095 is hypothetical protein
- Best hit was 8rbs chain K: Penton protein

target	prob	fident	alnlen	evalue	theader
8rbs-assembly1.cif.gz_K	1	0.103	799	1.186e-10	Emiliania huxleyi virus 201 (EhV-201) asymmetrical unit of capsid proteins predicted by AlphaFold2 fitted into the cryo-EM density of EhV-201 virion composite map.
8h2i-assembly1.cif.gz_bG	1	0.135	503	3.282e-08	Near-atomic structure of five-fold averaged PBCV-1 capsid
6g41-assembly1.cif.gz_B	1	0.103	396	2.489e-05	Crystal structure of SeMet-labeled mavirus penton protein

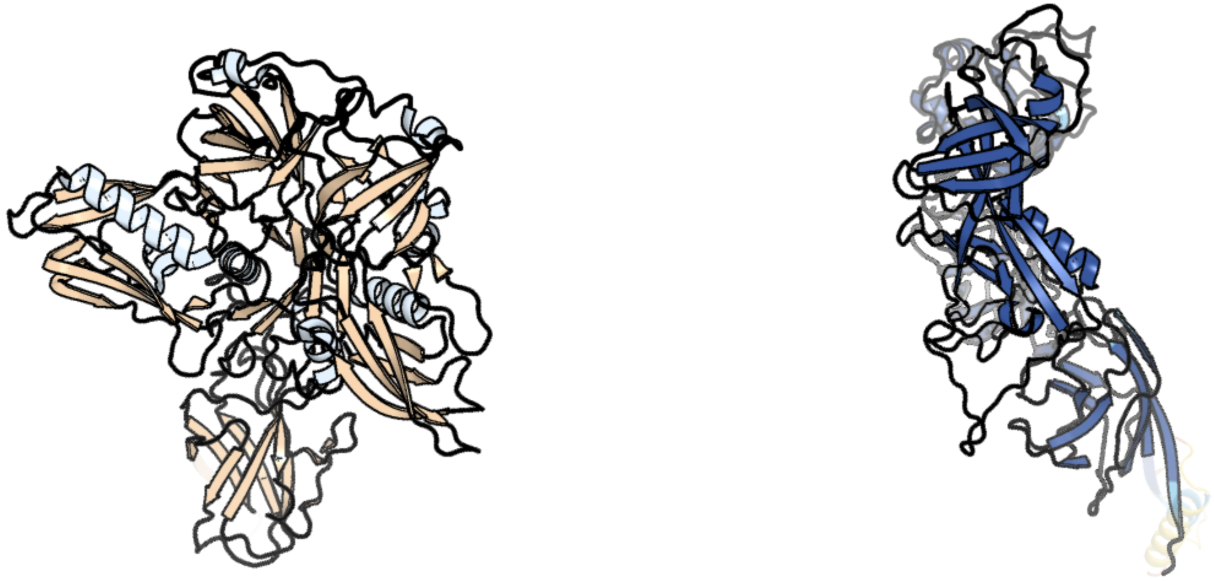

Figure 95: left: reference structure of 8rbs chain K. right: predicted structure of chlorv-1..095, unaligned sequences are shown as transparent

chlorv-1..096

- Sequence-based annotation for chlorv-1..096 is hypothetical protein
- No significant structural hit found

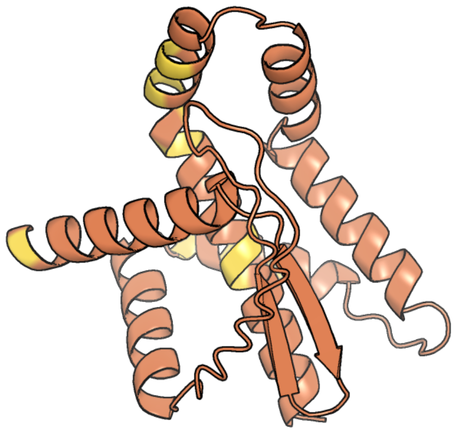

Figure 96: predicted structure of chlorv-1..096

chlorv-1..097

- Sequence-based annotation for chlorv-1..097 is hypothetical protein
- No significant structural hit found

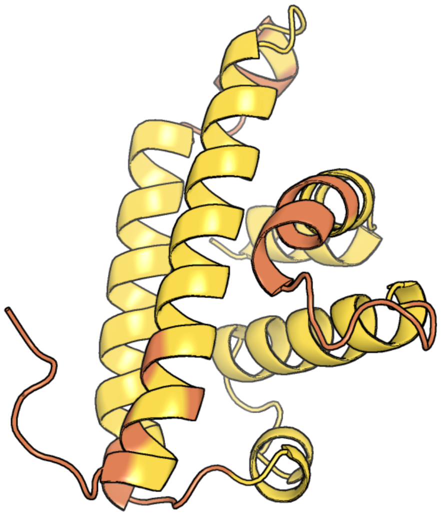

Figure 97: predicted structure of chlorv-1..097

chlorv-1..098

- Sequence-based annotation for chlorv-1..098 is hypothetical protein
- No significant structural hit found

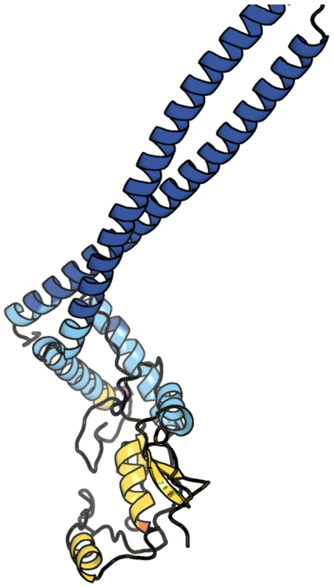

Figure 98: predicted structure of chlorv-1..098

chlorv-1..099

- Sequence-based annotation for chlorv-1..099 is putative Ceramidase
- Best hit was 6yxh chain A: Alkaline ceramidase 3

target	prob	fident	alnlen	evalue	theader
6yxh-assembly1.cif.gz_A	1	0.2	230	3.071e-07	Cryogenic human alkaline ceramidase 3 (ACER3) at 2.6 A resolution determined by Serial Crystallography (SSX) using CrystalDirect
7y69-assembly1.cif.gz_B	1	0.134	291	0.02441	ApoSIDT2-pH5.5
7y68-assembly1.cif.gz_B	0.988	0.119	293	0.06607	SIDT2-pH5.5 plus miRNA

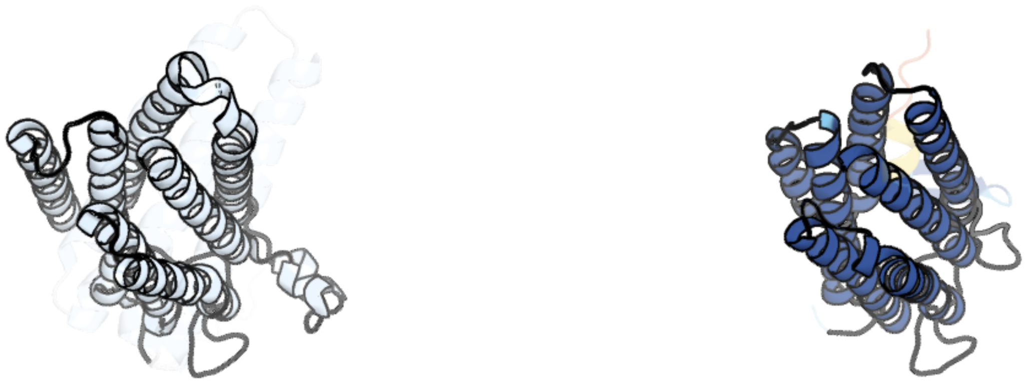

Figure 99: left: reference structure of 6yxh chain A. right: predicted structure of chlorv-1..099, unaligned sequences are shown as transparent

chlorv-1..100

- Sequence-based annotation for chlorv-1..100 is hypothetical protein
- No significant structural hit found

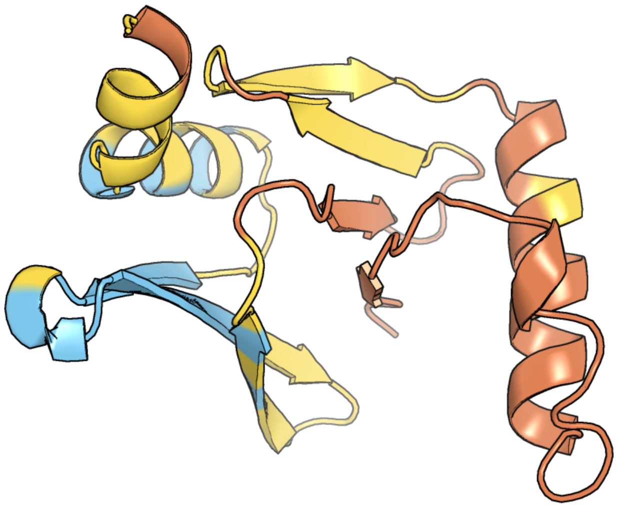

Figure 100: predicted structure of chlorv-1..100

chlorv-1..101

- Sequence-based annotation for chlorv-1..101 is hypothetical protein
- No significant structural hit found

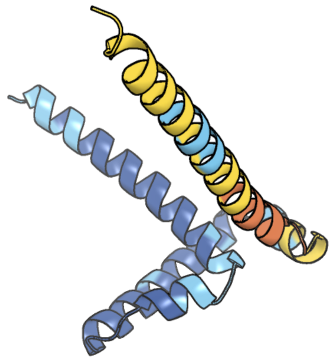

Figure 101: predicted structure of chlorv-1..101

chlorv-1..102

- Sequence-based annotation for chlorv-1..102 is hypothetical protein
- No significant structural hit found

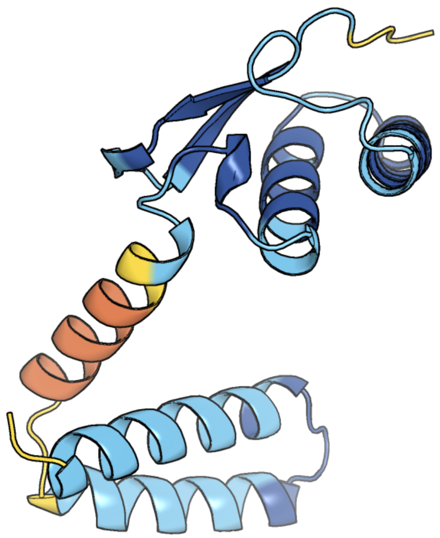

Figure 102: predicted structure of chlorv-1..102

chlorv-1..103

- Sequence-based annotation for chlorv-1..103 is putative Ribonuclease III
- Best hit was 1o0w chain A: Ribonuclease III

target	prob	fident	alnlen	evalue	theader
1o0w-assembly1.cif.gz_A	1	0.239	271	4.342e-13	Crystal structure of Ribonuclease III (TM1102) from Thermotoga maritima at 2.0 A resolution
7r97-assembly1.cif.gz_B	1	0.213	262	2.069e-12	Crystal structure of postcleavge complex of Escherichia coli RNase III
1rc7-assembly1.cif.gz_A	1	0.217	258	1.163e-10	Crystal structure of RNase III Mutant E110K from Aquifex Aeolicus complexed with ds-RNA at 2.15 Angstrom Resolution

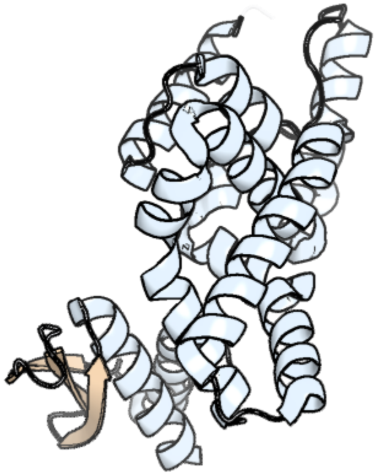

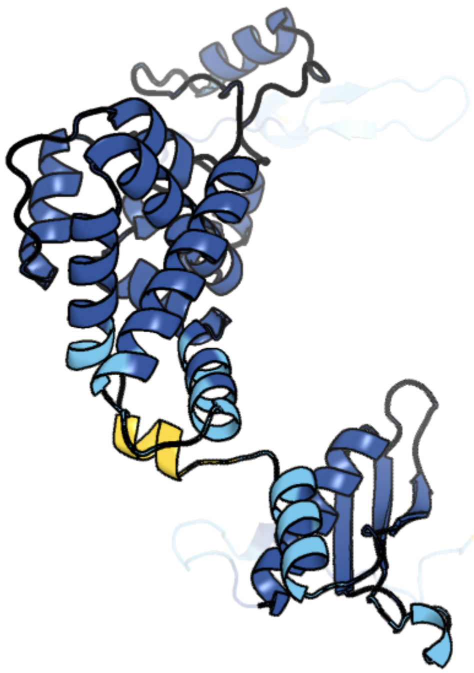

Figure 103: left: reference structure of 1o0w chain A. right: predicted structure of chlorv-1..103, unaligned sequences are shown as transparent

chlorv-1..104

- Sequence-based annotation for chlorv-1..104 is putative Nuclease
- Best hit was 3q8l chain A: Flap endonuclease 1

target	prob	fident	alnlen	evalue	theader
3q8l-assembly1.cif.gz_A	1	0.302	340	4.379e-25	Crystal Structure of Human Flap Endonuclease FEN1 (WT) in complex with substrate 5'-flap DNA, SM3+, and K+
5um9-assembly1.cif.gz_A	1	0.294	340	7.788e-25	Flap endonuclease 1 (FEN1) D86N with 5'-flap substrate DNA and Sm3+
5k97-assembly1.cif.gz_A	1	0.292	339	9.602e-25	Flap endonuclease 1 (FEN1) D233N with cleaved product fragment and Sm3+

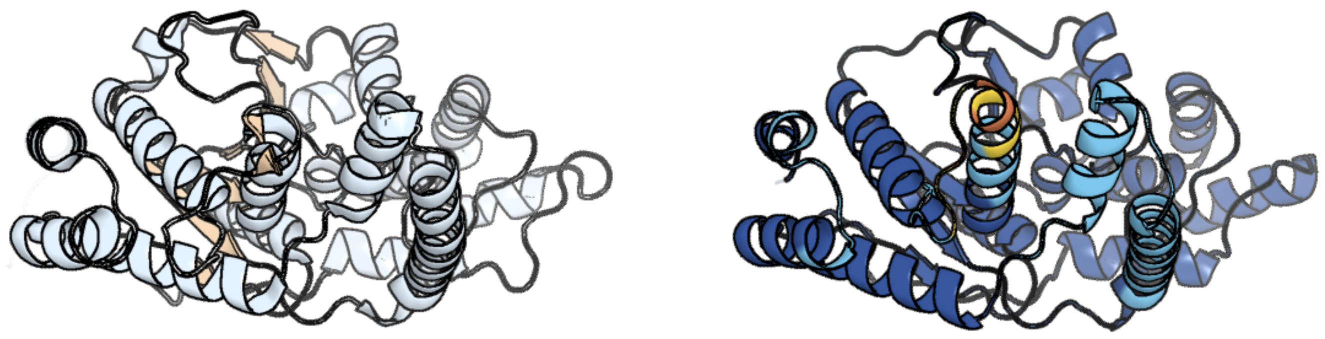

Figure 104: left: reference structure of 3q8l chain A. right: predicted structure of chlorv-1..104, unaligned sequences are shown as transparent

chlorv-1..105

- Sequence-based annotation for chlorv-1..105 is putative Nuclease
- Best hit was 3pif chain B: 5'->3' EXORIBONUCLEASE (xrn1)

target	prob	fident	alnlen	evalue	theader
3pif-assembly2.cif.gz_B	1	0.187	681	6.256e-14	Crystal structure of the 5'->3' exoribonuclease Xrn1, E178Q mutant in Complex with Manganese
3fqd-assembly1.cif.gz_A	1	0.151	711	6.54e-14	Crystal Structure of the S. pombe Rat1-Rai1 Complex
7opk-assembly1.cif.gz_A	1	0.161	669	2.17e-13	Crystal structure of C. thermophilum Xrn2

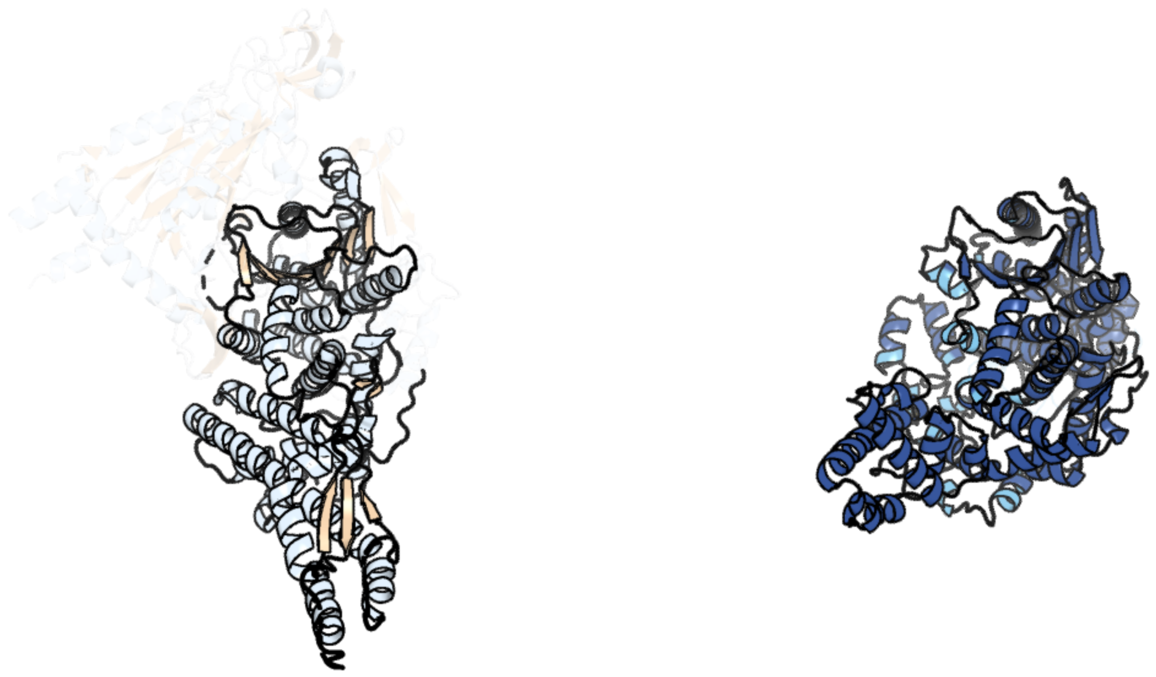

Figure 105: left: reference structure of 3pif chain B. right: predicted structure of chlorv-1..105, unaligned sequences are shown as transparent

chlorv-1..106

- Sequence-based annotation for chlorv-1..106 is putative Peptidase
- Best hit was 6on2 chain C: ATP-dependent protease La

target	prob	fident	alnlen	evalue	theader
6on2-assembly1.cif.gz_C	1	0.288	540	1.273e-38	Lon Protease from Yersinia pestis with Y2853 substrate
7p0m-assembly1.cif.gz_B	1	0.27	554	1.805e-38	Human mitochondrial Lon protease with substrate in the ATPase and protease domains
7krz-assembly1.cif.gz_D	1	0.271	557	1.994e-38	Human mitochondrial LONP1 in complex with Bortezomib

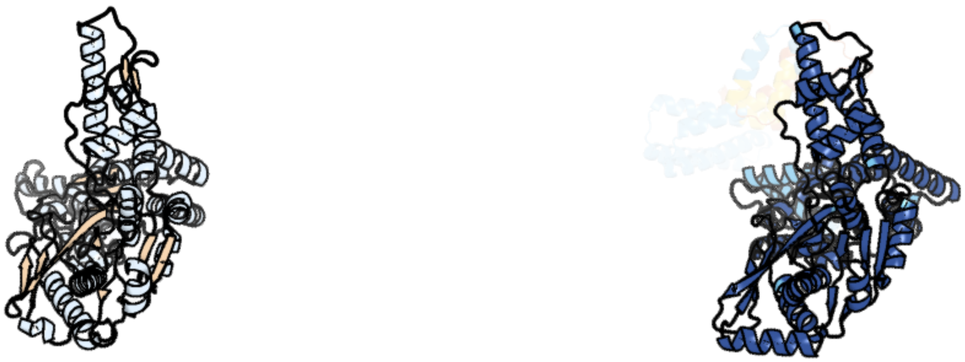

Figure 106: left: reference structure of 6on2 chain C. right: predicted structure of chlorv-1..106, unaligned sequences are shown as transparent

chlorv-1..107

- Sequence-based annotation for chlorv-1..107 is putative DNA ligase
- Best hit was 5tt5 chain A: DNA ligase

target	prob	fident	alnlen	evalue	theader
5tt5-assembly1.cif.gz_A	1	0.176	605	1.772e-30	Escherichia coli LigA (K115M) in complex with NAD+
2owo-assembly1.cif.gz_A	1	0.178	609	5.476e-30	Last Stop on the Road to Repair: Structure of E.coli DNA Ligase Bound to Nicked DNA-Adenylate
4glx-assembly1.cif.gz_A	1	0.177	597	8.881e-30	DNA ligase A in complex with inhibitor

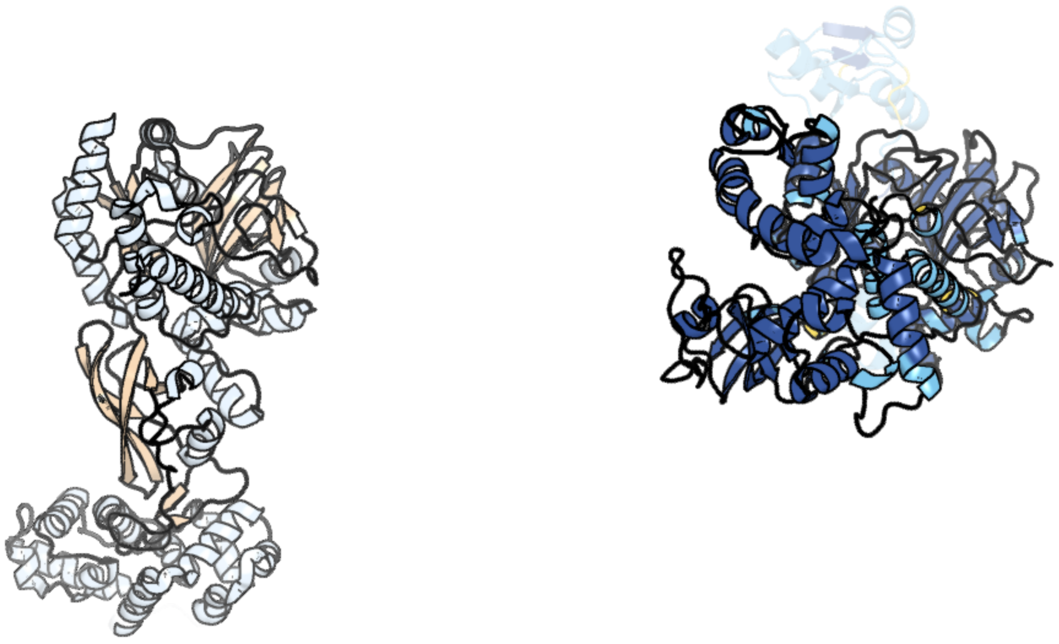

Figure 107: left: reference structure of 5tt5 chain A. right: predicted structure of chlorv-1..107, unaligned sequences are shown as transparent

chlorv-1..108

- Sequence-based annotation for chlorv-1..108 is putative Cytidine deaminase
- Best hit was 7zob chain E: Metagenomic cytidine deaminase Cdd

target	prob	fident	alnlen	evalue	theader
7zob-assembly2.cif.gz_E	1	0.409	127	2.885e-15	Metagenomic cytidine deaminase Cdd
1r5t-assembly1.cif.gz_D	1	0.333	132	2.791e-14	The Crystal Structure of Cytidine Deaminase CDD1, an Orphan C to U editase from Yeast
3dmo-assembly1.cif.gz_C	1	0.33	127	4.394e-14	1.6 A crystal structure of cytidine deaminase from Burkholderia pseudomallei

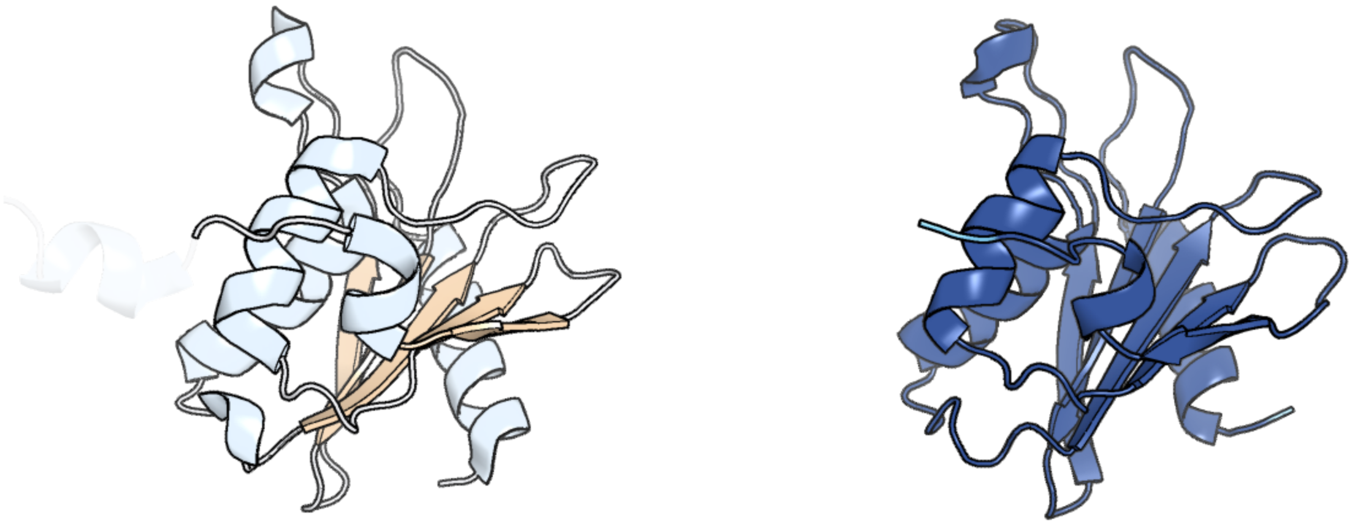

Figure 108: left: reference structure of 7zob chain E. right: predicted structure of chlorv-1..108, unaligned sequences are shown as transparent

chlorv-1..109

- Sequence-based annotation for chlorv-1..109 is hypothetical protein
- No significant structural hit found

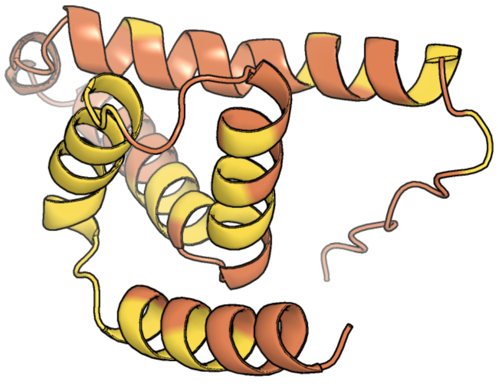

Figure 109: predicted structure of chlorv-1..109

chlorv-1..110

- Sequence-based annotation for chlorv-1..110 is putative RNA ligase
- Best hit was 2c5u chain B: RNA LIGASE

target	prob	fident	alnlen	evalue	theader
2c5u-assembly2.cif.gz_B	1	0.148	405	2.005e-10	T4 RNA Ligase (Rnl1) Crystal Structure
5tt6-assembly1.cif.gz_A	1	0.154	448	3.89e-09	T4 RNA Ligase 1 (K99M)
6n67-assembly1.cif.gz_A	1	0.109	422	1.184e-06	Crystal structure of the ligase domain of fungal tRNA ligase Trl1

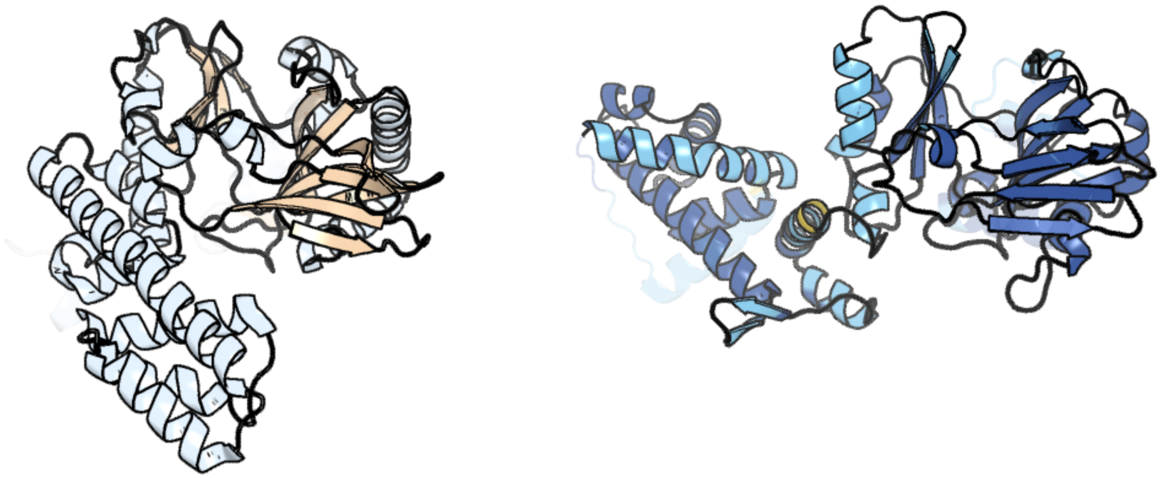

Figure 110: left: reference structure of 2c5u chain B. right: predicted structure of chlorv-1..110, unaligned sequences are shown as transparent

chlorv-1..111

- Sequence-based annotation for chlorv-1..111 is putative Phosphatase
- Best hit was 2iq1 chain A: Protein phosphatase 2C kappa, PPM1K

target	prob	fident	alnlen	evalue	theader
2iq1-assembly1.cif.gz_A	1	0.191	324	3.039e-19	Crystal structure of human PPM1K
6ak7-assembly1.cif.gz_A	1	0.19	320	5.325e-18	Crystal structure of PPM1K-N94K
4da1-assembly1.cif.gz_A	1	0.19	326	2.511e-17	Crystal structure of branched-chain alpha-ketoacid dehydrogenase phosphatase with Mg (II) ions at the active site

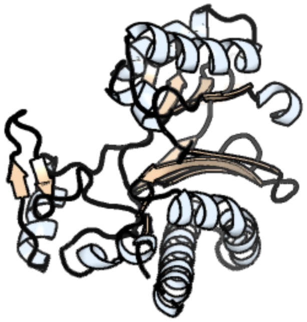

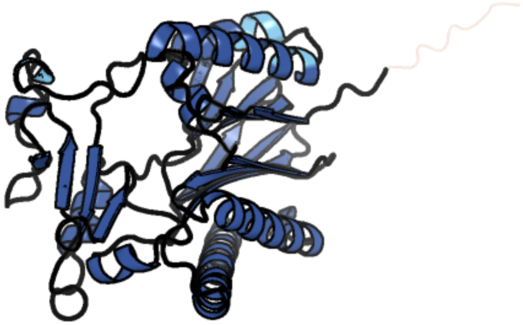

Figure 111: left: reference structure of 2iq1 chain A. right: predicted structure of chlorv-1..111, unaligned sequences are shown as transparent

chlorv-1..112

- Sequence-based annotation for chlorv-1..112 is hypothetical protein
- No significant structural hit found

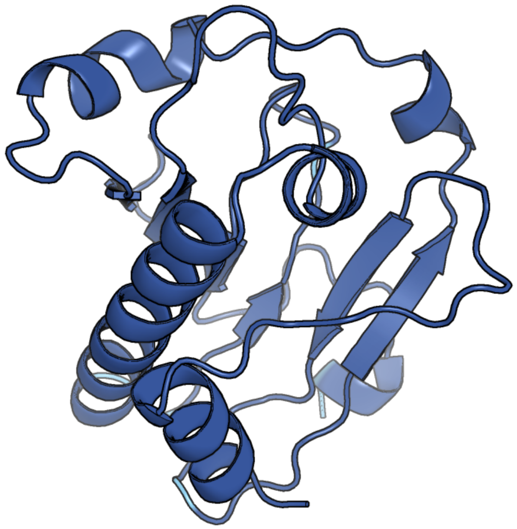

Figure 112: predicted structure of chlorv-1..112

chlorv-1..113

- Sequence-based annotation for chlorv-1..113 is putative translation initiation factor eIF2 gamma subunit
- Best hit was 6r8s chain A: Translation initiation factor 2 subunit gamma

target	prob	fident	alnlen	evalue	theader
6r8s-assembly1.cif.gz_A	1	0.33	411	4.892e-46	Crystal structure of aIF2gamma subunit I181K from archaeon Sulfolobus solfataricus complexed with GDPCP
4rd0-assembly1.cif.gz_A	1	0.327	409	1.474e-45	Structure of aIF2-gamma D19A variant from Sulfolobus solfataricus bound to GDP
4qfm-assembly1.cif.gz_A	1	0.33	411	1.971e-45	The structure of aIF2gamma subunit D152A from archaeon Sulfolobus solfataricus complexed with GDPCP

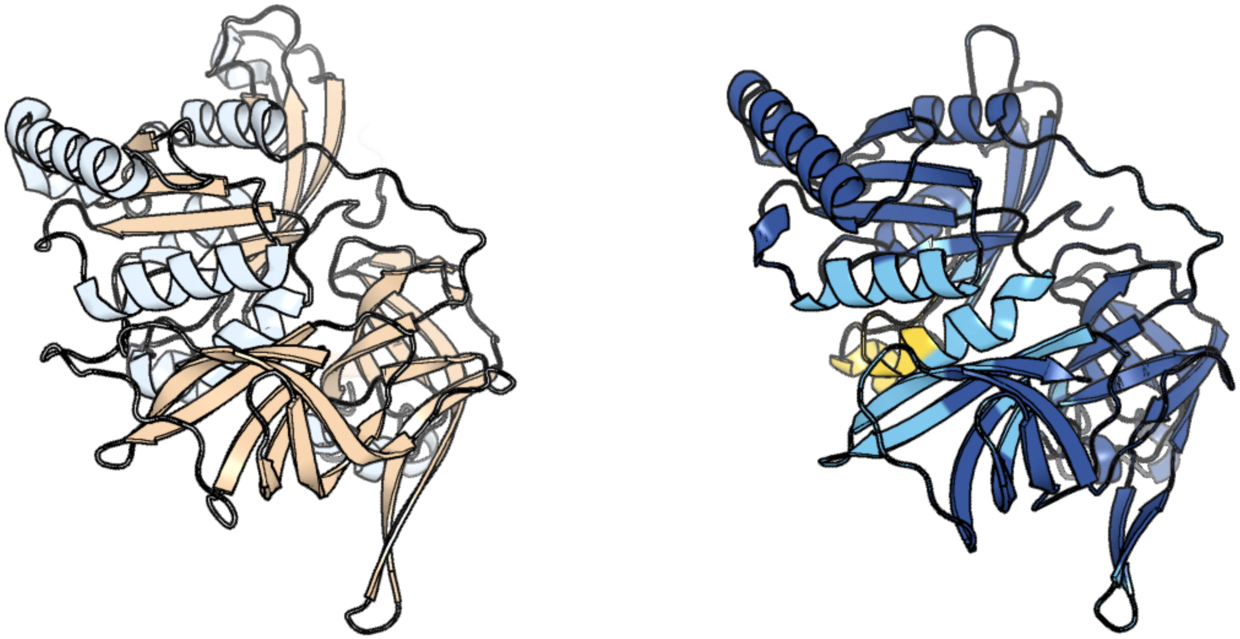

Figure 113: left: reference structure of 6r8s chain A. right: predicted structure of chlorv-1..113, unaligned sequences are shown as transparent

chlorv-1..114

- Sequence-based annotation for chlorv-1..114 is hypothetical protein
- No significant structural hit found

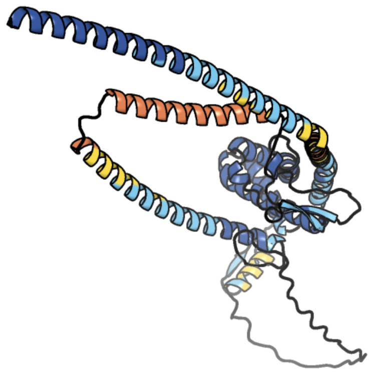

Figure 114: predicted structure of chlorv-1..114

chlorv-1..115

- Sequence-based annotation for chlorv-1..115 is putative DUF285 domain-containing protein
- No significant structural hit found

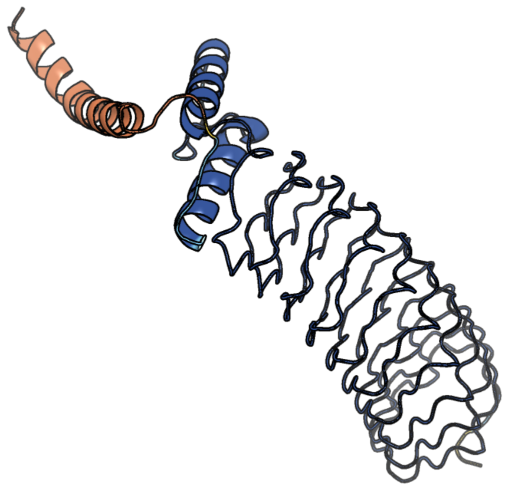

Figure 115: predicted structure of chlorv-1..115

chlorv-1..116

- Sequence-based annotation for chlorv-1..116 is putative DUF285 domain-containing protein
- No significant structural hit found

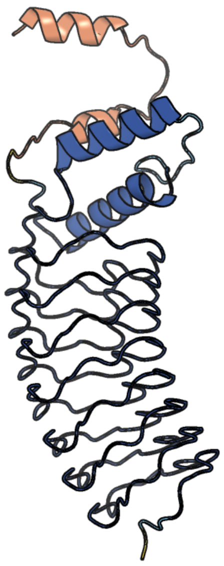

Figure 116: predicted structure of chlorv-1..116

chlorv-1..117

- Sequence-based annotation for chlorv-1..117 is hypothetical protein
- No significant structural hit found

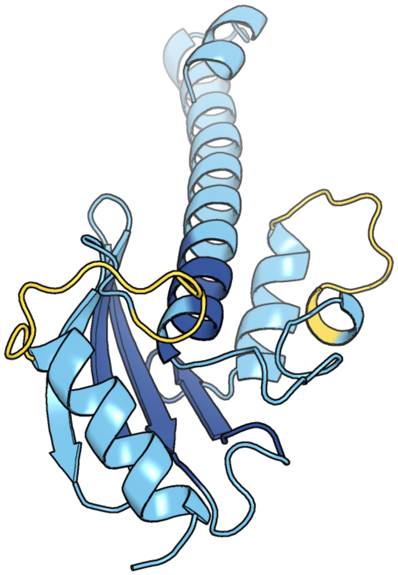

Figure 117: predicted structure of chlorv-1..117

chlorv-1..118

- Sequence-based annotation for chlorv-1..118 is putative IMP dehydrogenase / GMP reductase
- Best hit was 6jig chain A: GMP reductase

target	prob	fident	alnlen	evalue	theader
6jig-assembly1.cif.gz_A	1	0.398	487	2.217e-63	Crystal structure of GMP reductase C318A from Trypanosoma brucei in complex with guanosine 5'-monophosphate
3tsb-assembly1.cif.gz_A	1	0.354	483	4.499e-57	Crystal Structure of Inosine-5'-monophosphate Dehydrogenase from Bacillus anthracis str. Ames
6jl8-assembly1.cif.gz_A	1	0.386	481	1.008e-56	Crystal structure of GMP reductase C318A from Trypanosoma brucei

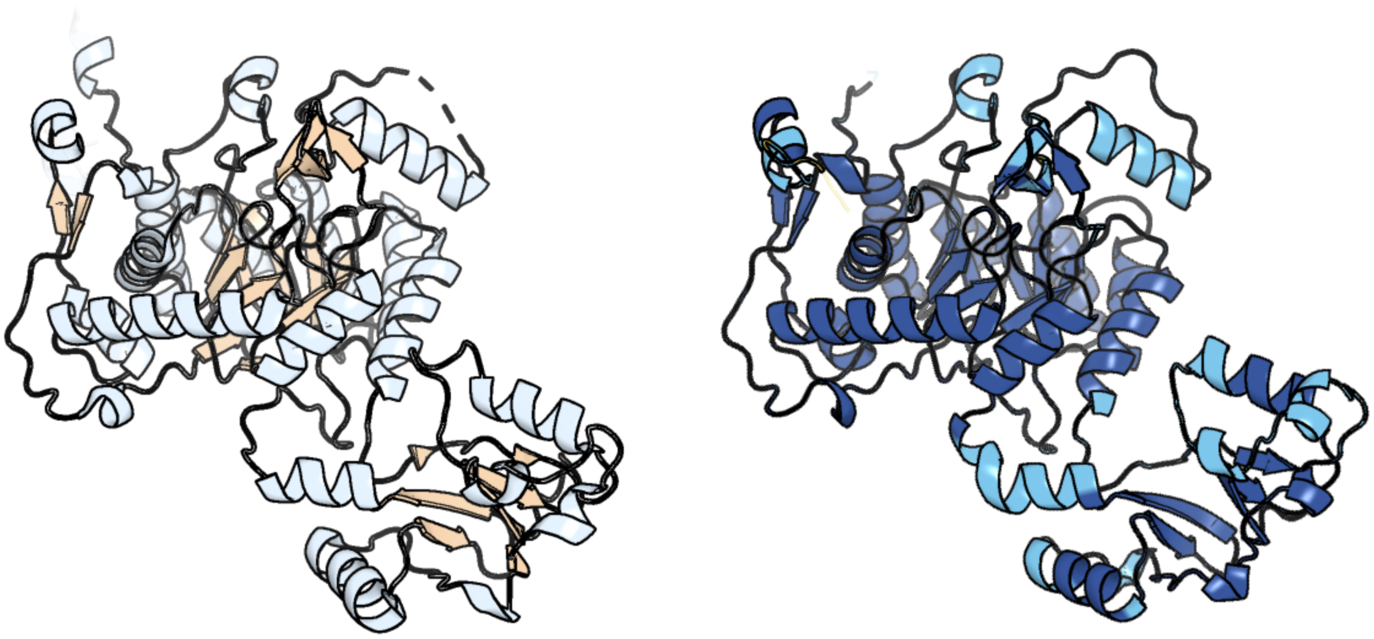

Figure 118: left: reference structure of 6jig chain A. right: predicted structure of chlorv-1..118, unaligned sequences are shown as transparent

chlorv-1..119

- Sequence-based annotation for chlorv-1..119 is putative Peptidase
- Best hit was 6on2 chain C: ATP-dependent protease La

target	prob	fident	alnlen	evalue	theader
7p0m-assembly1.cif.gz_B	1	0.263	619	9.47e-37	Human mitochondrial Lon protease with substrate in the ATPase and protease domains
6on2-assembly1.cif.gz_C	1	0.276	597	1.518e-36	Lon Protease from Yersinia pestis with Y2853 substrate
7p0m-assembly1.cif.gz_A	1	0.271	594	1.834e-36	Human mitochondrial Lon protease with substrate in the ATPase and protease domains

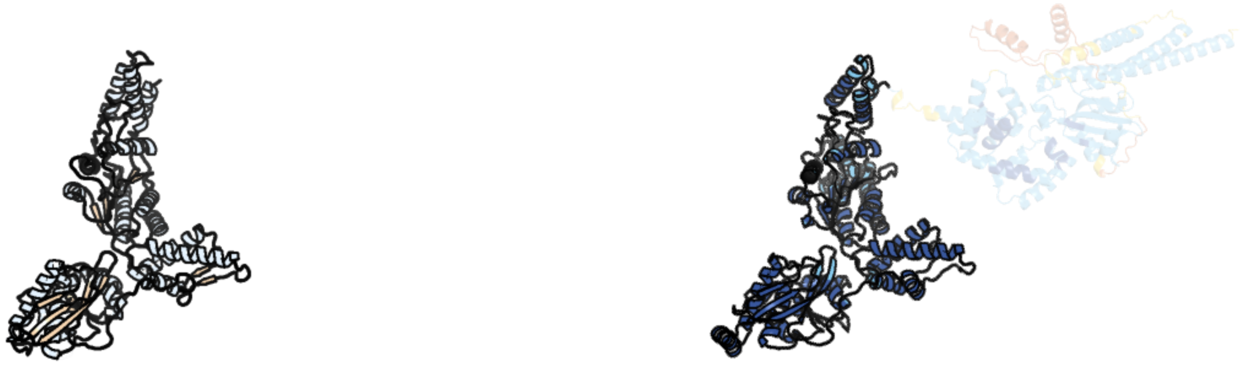

Figure 119: left: reference structure of 6on2 chain C. right: predicted structure of chlorv-1..119, unaligned sequences are shown as transparent

chlorv-1..120

- Sequence-based annotation for chlorv-1..120 is putative Rhodanese-like domain
- Best hit was 3tp9 chain B: BETA-LACTAMASE and RHODANESE DOMAIN PROTEIN

target	prob	fident	alnlen	evalue	theader
3tp9-assembly1.cif.gz_B	1	0.231	95	3.534e-05	Crystal structure of Alicyclobacillus acidocaldarius protein with beta-lactamase and rhodanese domains
6mxv-assembly1.cif.gz_A	1	0.277	108	4.299e-05	The crystal structure of a rhodanese-like family protein from Francisella tularensis subsp. tularensis SCHU S4
3o3w-assembly2.cif.gz_D	1	0.238	105	6.361e-05	Crystal Structure of BH2092 protein (residues 14-131) from Bacillus halodurans, Northeast Structural Genomics Consortium Target BhR228A

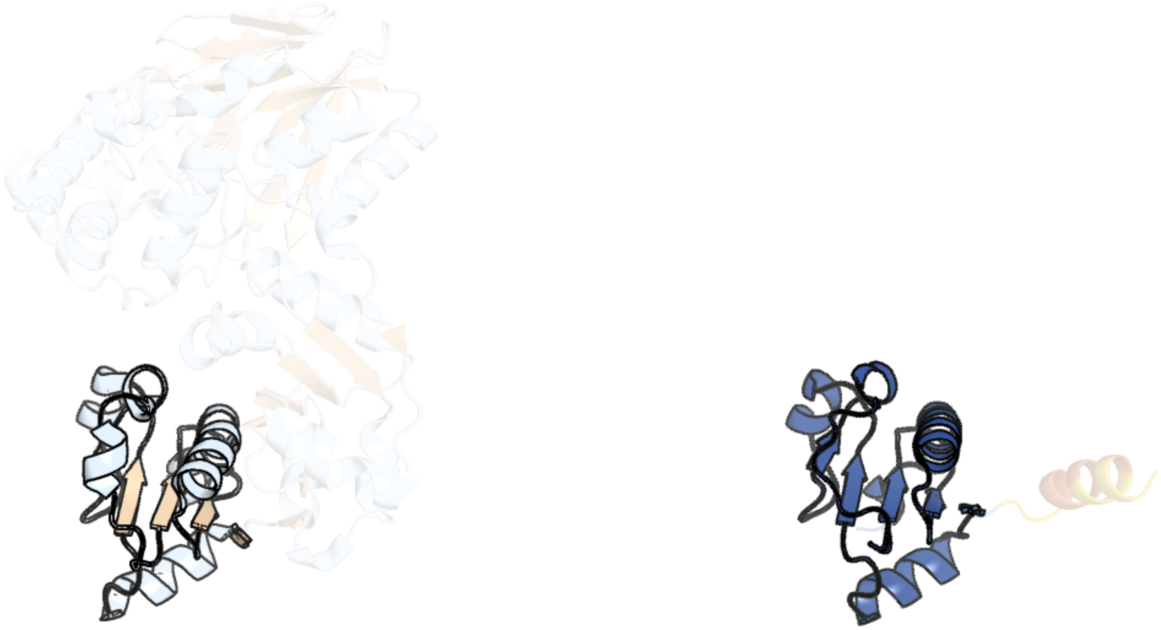

Figure 120: left: reference structure of 3tp9 chain B. right: predicted structure of chlorv-1..120, unaligned sequences are shown as transparent

chlorv-1..121

- Sequence-based annotation for chlorv-1..121 is hypothetical protein
- Best hit was 4gf1 chain B: Putative ADP-ribosyltransferase Certhrax

target	prob	fident	alnlen	evalue	theader
4gf1-assembly2.cif.gz_B	1	0.164	419	1.677e-08	Crystal Structure of Certhrax
4fxq-assembly2.cif.gz_B	1	0.167	419	1.86e-08	Full-length Certhrax toxin from Bacillus cereus in complex with Inhibitor P6
4gf1-assembly1.cif.gz_A	1	0.181	396	4.499e-08	Crystal Structure of Certhrax

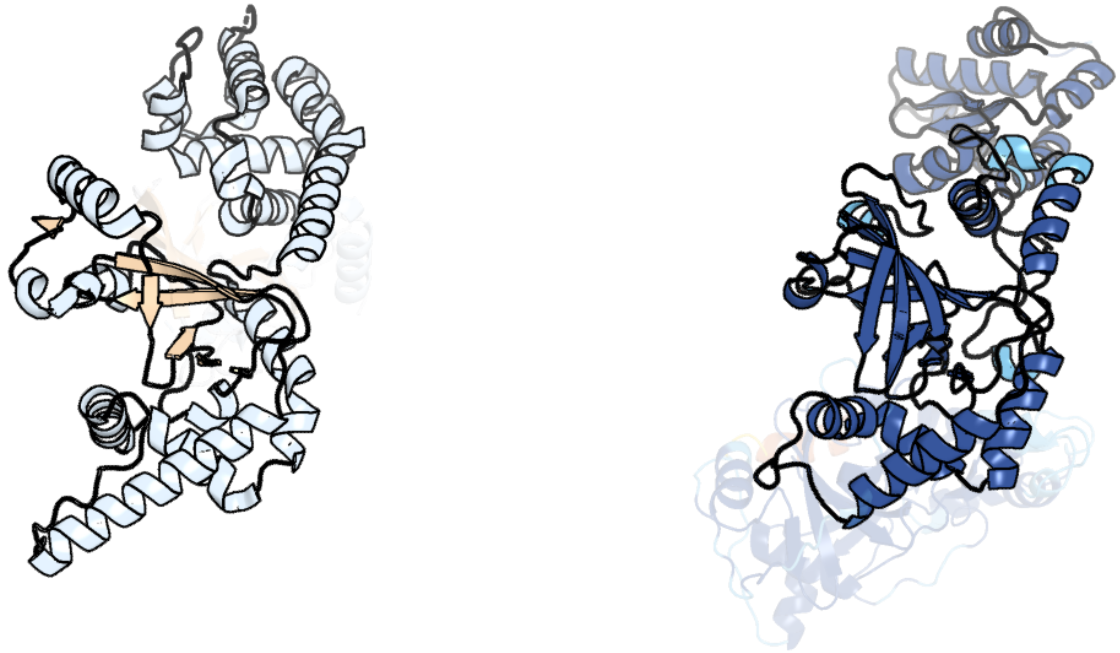

Figure 121: left: reference structure of 4gf1 chain B. right: predicted structure of chlorv-1..121, unaligned sequences are shown as transparent

chlorv-1..122

- Sequence-based annotation for chlorv-1..122 is hypothetical protein
- Best hit was 5gm6 chain e: Protein HSH49

target	prob	fident	alnlen	evalue	theader
5gm6-assembly1.cif.gz_e	1	0.137	160	6.54e-06	Cryo-EM structure of the activated spliceosome (Bact complex) at 3.5 angstrom resolution
7abi-assembly1.cif.gz_B	1	0.15	180	7.819e-06	Human pre-Bact-2 spliceosome
7uk1-assembly1.cif.gz_B	1	0.125	175	1.186e-05	Complex Structure of Human Polypyrimidine Splicing Factor (PSF/SFPQ) with Murine Virus-like 30S Transcript-1 (VS30-1) Reveals Cooperative Binding of RNA

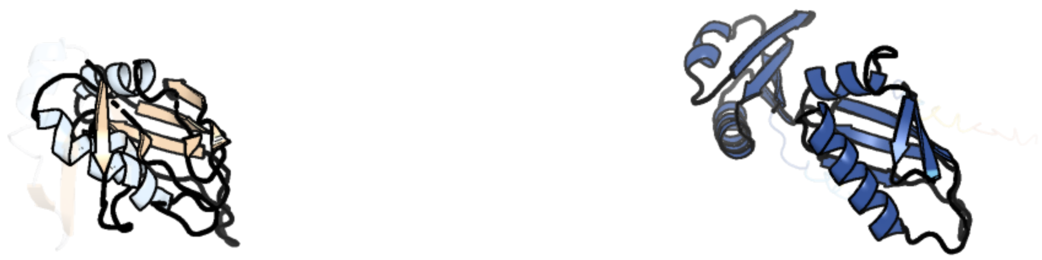

Figure 122: left: reference structure of 5gm6 chain e. right: predicted structure of chlorv-1..122, unaligned sequences are shown as transparent

chlorv-1..123

- Sequence-based annotation for chlorv-1..123 is putative Ribonuclease
- Best hit was 7uwe chain C: Ribonuclease HII

target	prob	fident	alnlen	evalue	theader
7uwe-assembly1.cif.gz_C	1	0.364	192	6.732e-19	CryoEM Structure of E. coli Transcription-Coupled Ribonucleotide Excision Repair (TC-RER) complex
3o3g-assembly1.cif.gz_A	1	0.384	190	1.817e-18	T. maritima RNase H2 in complex with nucleic acid substrate and calcium ions
5y9p-assembly1.cif.gz_A	1	0.354	189	2.636e-18	Staphylococcus aureus RNase HII

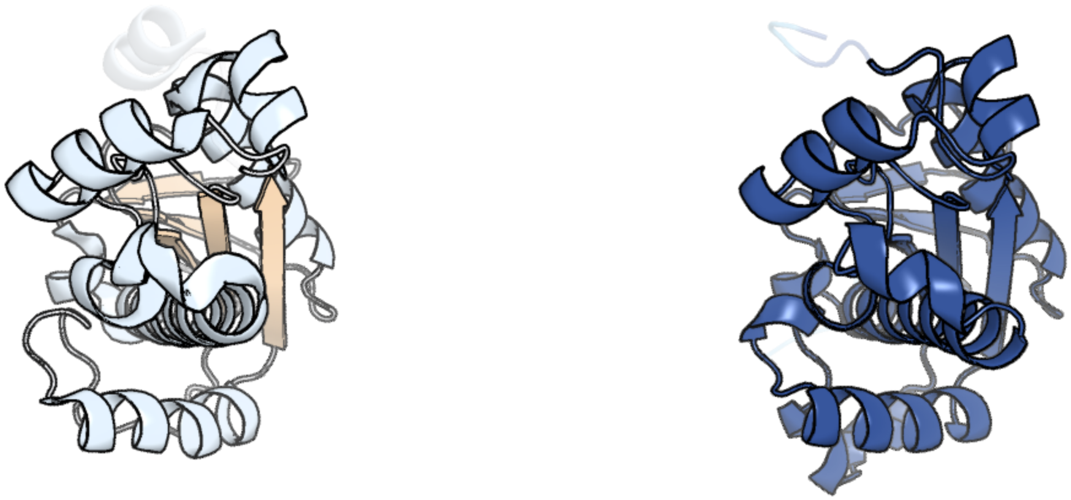

Figure 123: left: reference structure of 7uwe chain C. right: predicted structure of chlorv-1..123, unaligned sequences are shown as transparent

chlorv-1..124

- Sequence-based annotation for chlorv-1..124 is putative Exonuclease
- Best hit was 7opk chain A: 5'-3' exoribonuclease

target	prob	fident	alnlen	evalue	theader
7opk-assembly1.cif.gz_A	1	0.301	574	8.139e-35	Crystal structure of C. thermophilum Xrn2
3fqd-assembly1.cif.gz_A	1	0.298	600	2.205e-34	Crystal Structure of the S. pombe Rat1-Rai1 Complex
5fir-assembly4.cif.gz_G	1	0.261	566	9.262e-33	Crystal structure of C. elegans XRN2 in complex with the XRN2-binding domain of PAXT-1

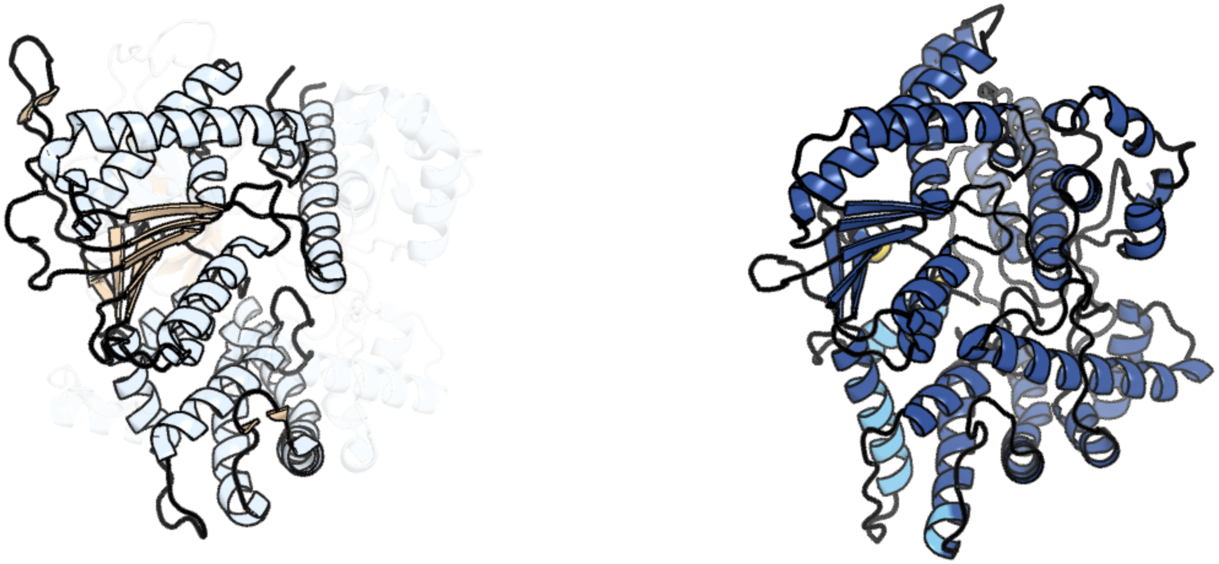

Figure 124: left: reference structure of 7opk chain A. right: predicted structure of chlorv-1..124, unaligned sequences are shown as transparent

chlorv-1..125

- Sequence-based annotation for chlorv-1..125 is putative Protein kinase
- Best hit was 2wb8 chain A: SERINE/THREONINE-PROTEIN KINASE HASPIN

target	prob	fident	alnlen	evalue	theader
2wb8-assembly1.cif.gz_A	1	0.162	370	2.937e-11	Crystal structure of Haspin kinase
6g3a-assembly1.cif.gz_A	1	0.163	367	1.079e-10	Crystal structure of haspin F605T mutant in complex with 5-iodotubercidin
6d3k-assembly1.cif.gz_A	1	0.146	347	2.656e-07	Crystal structure of unphosphorylated human PKR kinase domain in complex with ADP

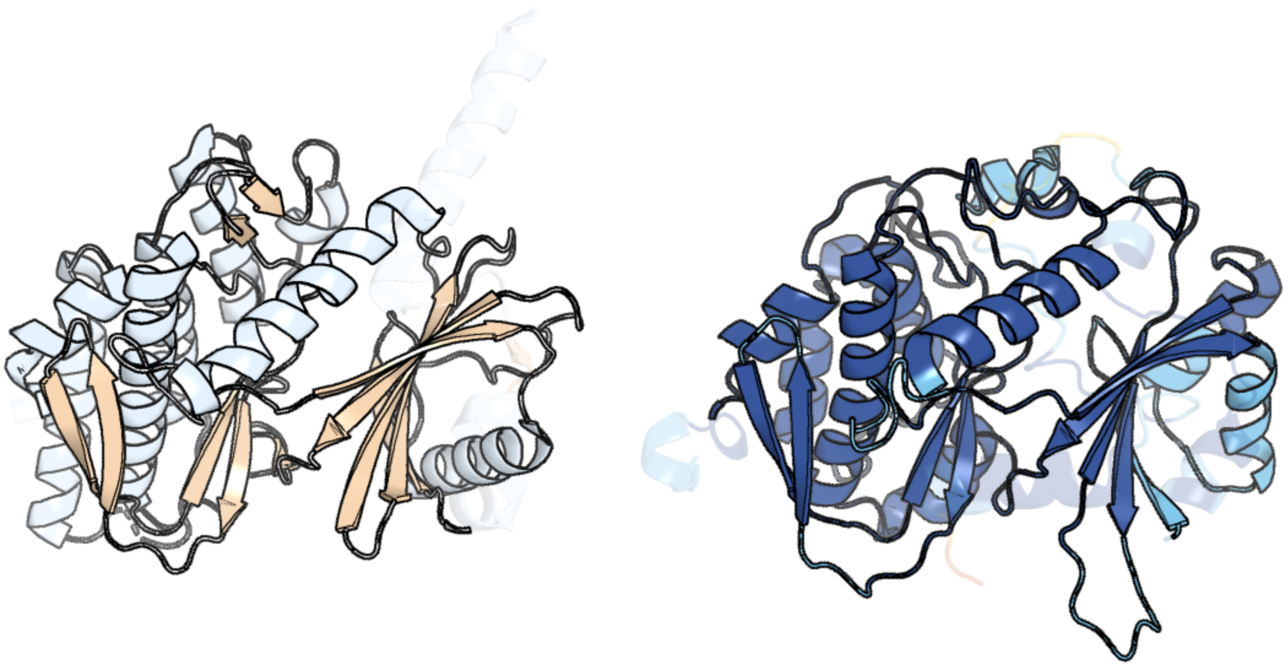

Figure 125: left: reference structure of 2wb8 chain A. right: predicted structure of chlorv-1..125, unaligned sequences are shown as transparent

chlorv-1..126

- Sequence-based annotation for chlorv-1..126 is hypothetical protein
- No significant structural hit found

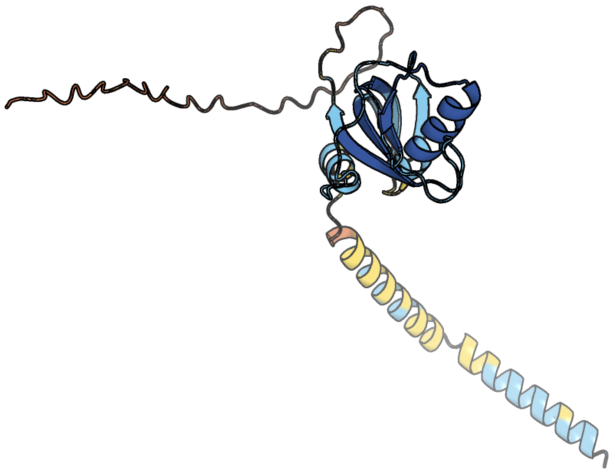

Figure 126: predicted structure of chlorv-1..126

chlorv-1..127

- Sequence-based annotation for chlorv-1..127 is hypothetical protein
- No significant structural hit found

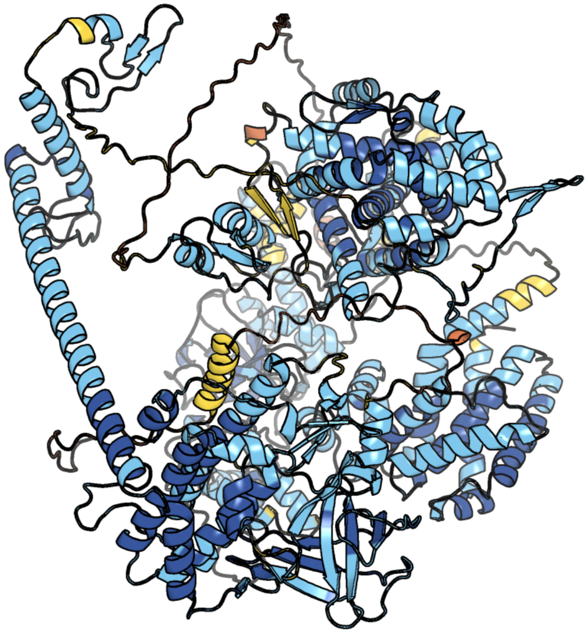

Figure 127: predicted structure of chlorv-1..127

chlorv-1..128

- Sequence-based annotation for chlorv-1..128 is hypothetical protein
- No significant structural hit found

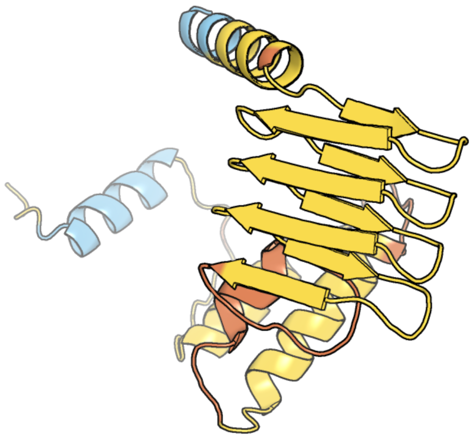

Figure 128: predicted structure of chlorv-1..128

chlorv-1..129

- Sequence-based annotation for chlorv-1..129 is putative Helicase
- Best hit was 7zsb chain 7: General transcription and DNA repair factor IIH helicase subunit XPB

target	prob	fident	alnlen	evalue	theader
7zsb-assembly1.cif.gz_7	1	0.155	503	7.849e-19	Yeast RNA polymerase II transcription pre-initiation complex with the +1 nucleosome and NTP, complex C
8bvw-assembly1.cif.gz_0	1	0.167	506	1.852e-18	RNA polymerase II pre-initiation complex with the distal +1 nucleosome (PIC-Nuc18W)
6p4w-assembly2.cif.gz_C	1	0.222	440	2.061e-18	XPB helicase in a complex with truncated Bax1 from Sulfurisphaera tokodaii at 2.96 Angstrom resolution

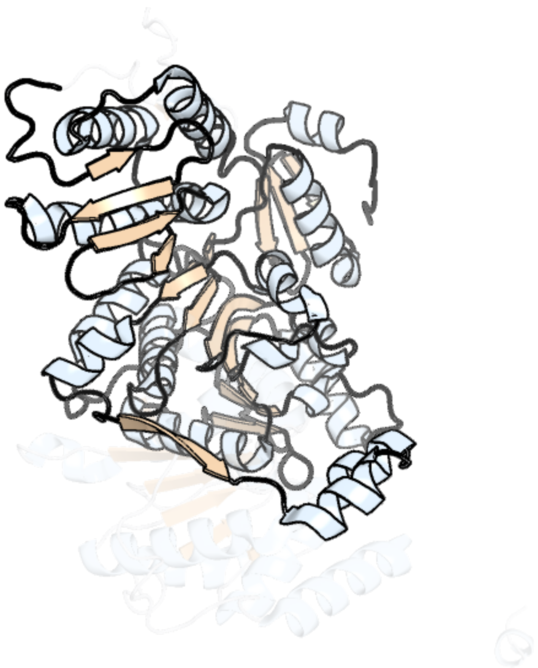

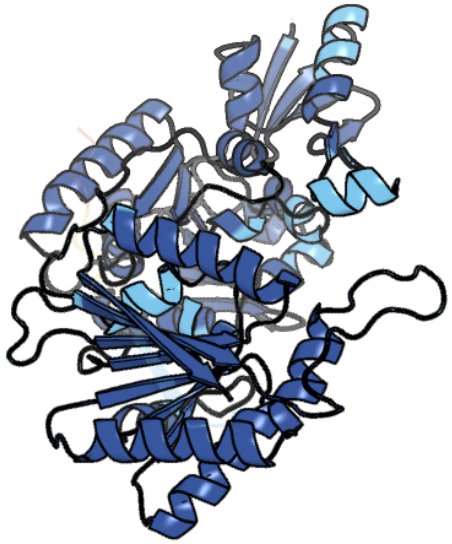

Figure 129: left: reference structure of 7zsb chain 7. right: predicted structure of chlorv-1..129, unaligned sequences are shown as transparent

chlorv-1..130

- Sequence-based annotation for chlorv-1..130 is hypothetical protein
- No significant structural hit found

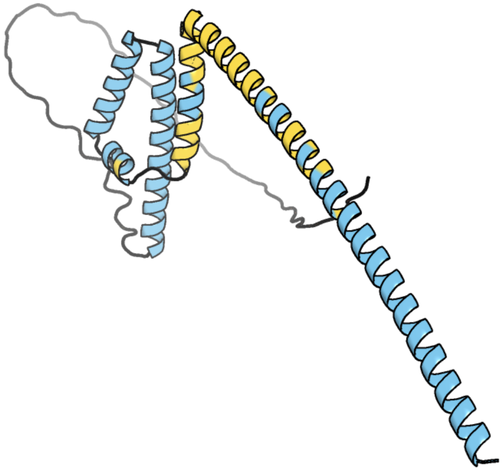

Figure 130: predicted structure of chlorv-1..130

chlorv-1..131

- Sequence-based annotation for chlorv-1..131 is putative Calcineurin-like phosphoesterase
- Best hit was 1v73 chain A: psychrophilic phosphatase I

target	prob	fident	alnlen	evalue	theader
1v73-assembly1.cif.gz_A	1	0.217	373	9.711e-17	Crystal Structure of Cold-Active Protein-Tyrosine Phosphatase of a Psychrophile Shewanella SP.
2zbm-assembly1.cif.gz_A	1	0.217	372	1.837e-16	Crystal Structure of I115M Mutant Cold-Active Protein Tyrosine Phosphatase
2z72-assembly1.cif.gz_A	1	0.214	359	3.281e-16	New Structure Of Cold-Active Protein Tyrosine Phosphatase At 1.1 Angstrom

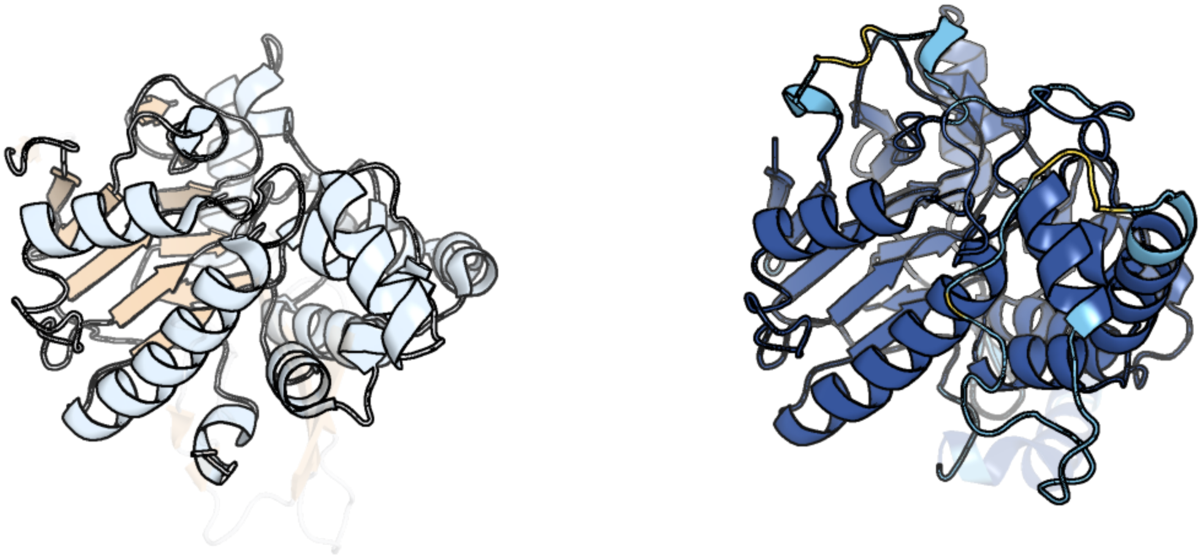

Figure 131: left: reference structure of 1v73 chain A. right: predicted structure of chlorv-1..131, unaligned sequences are shown as transparent

chlorv-1..132

- Sequence-based annotation for chlorv-1..132 is putative AAA family ATPase
- Best hit was 6sh3 chain A: Mitochondrial chaperone BCS1

target	prob	fident	alnlen	evalue	theader
6sh3-assembly1.cif.gz_A	1	0.269	375	8.27e-25	Structure of the ADP state of the heptameric Bcs1 AAA-ATPase
6uks-assembly1.cif.gz_A	1	0.251	389	8.774e-25	ATPgammaS bound mBcs1
6uko-assembly1.cif.gz_C	1	0.241	381	2.4e-24	Structure analysis of full-length mouse bcs1 complex

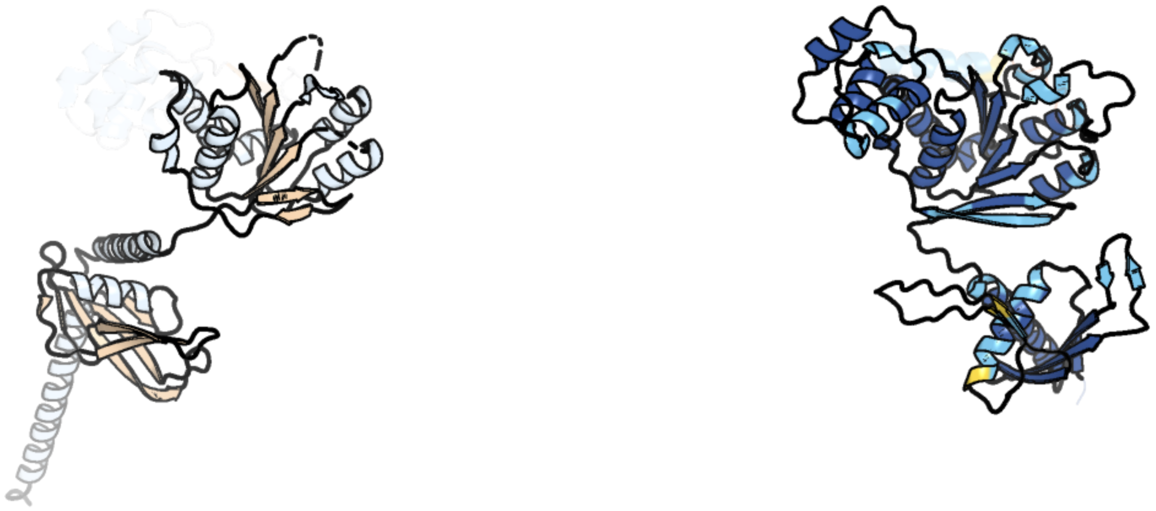

Figure 132: left: reference structure of 6sh3 chain A. right: predicted structure of chlorv-1..132, unaligned sequences are shown as transparent

chlorv-1..133

- Sequence-based annotation for chlorv-1..133 is putative eukaryotic translation initiation factor 5
- Best hit was 6sw9 chain 8: Translation initiation factor 2 subunit beta

target	prob	fident	alnlen	evalue	theader
8ppl-assembly1.cif.gz_Is	1	0.188	143	1.615e-07	MERS-CoV Nsp1 bound to the human 43S pre-initiation complex
6sw9-assembly1.cif.gz_8	1	0.196	127	4.562e-07	IC2A model of cryo-EM structure of a full archaeal ribosomal translation initiation complex devoid of aIF1 in P. abyssi
6fyy-assembly1.cif.gz_l	1	0.209	129	6.583e-07	Structure of a partial yeast 48S preinitiation complex with eIF5 N-terminal domain (model C2)

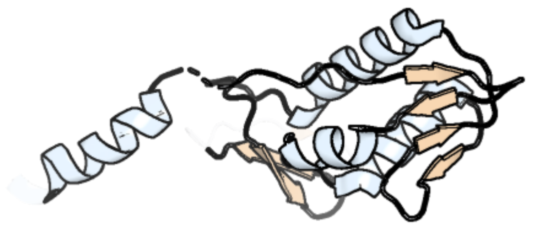

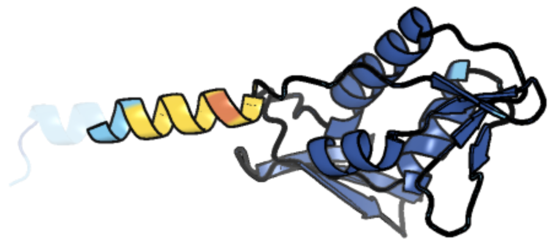

Figure 133: left: reference structure of 6sw9 chain 8. right: predicted structure of chlorv-1..133, unaligned sequences are shown as transparent

chlorv-1..134

- Sequence-based annotation for chlorv-1..134 is hypothetical protein
- No significant structural hit found

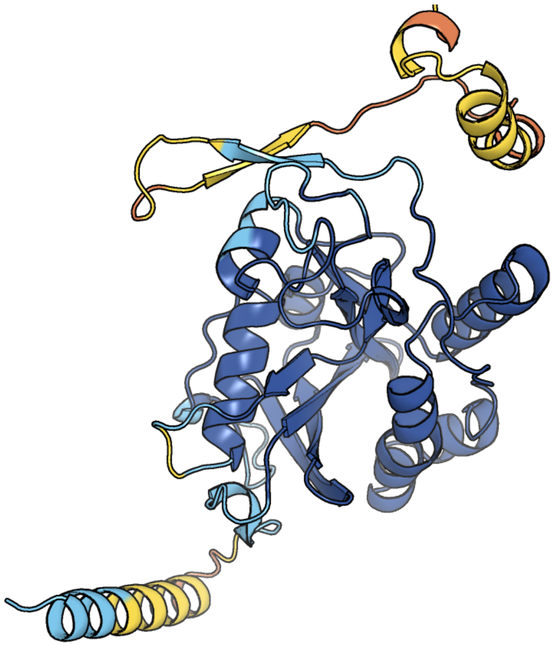

Figure 134: predicted structure of chlorv-1..134

chlorv-1..135

- Sequence-based annotation for chlorv-1..135 is hypothetical protein
- Best hit was 4d0q chain A: HYALURONATE LYASE

target	prob	fident	alnlen	evalue	theader
4d0q-assembly1.cif.gz_A	1	0.161	118	3.842e-05	Hyaluronan Binding Module of the Streptococcal Pneumoniae Hyaluronate Lyase
6xwv-assembly1.cif.gz_D	1	0.115	113	7.897e-05	Crystal structure of drosophila melanogaster CENP-C bound to CAL1
2xon-assembly1.cif.gz_A	0.998	0.175	131	8.347e-05	Structure of TmCBM61 in complex with beta-1,4-galactotriose at 1.4 A resolution

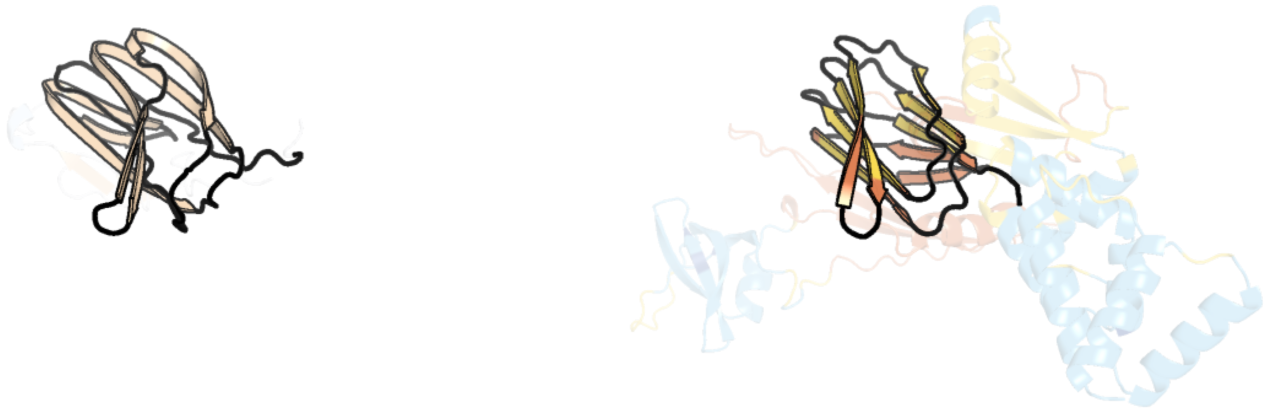

Figure 135: left: reference structure of 4d0q chain A. right: predicted structure of chlorv-1..135, unaligned sequences are shown as transparent

chlorv-1..136

- Sequence-based annotation for chlorv-1..136 is hypothetical protein
- No significant structural hit found

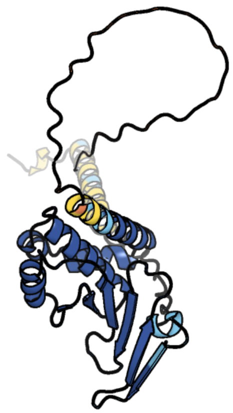

Figure 136: predicted structure of chlorv-1..136

chlorv-1..137

- Sequence-based annotation for chlorv-1..137 is hypothetical protein
- Best hit was 6tbn chain B: Probable cytosolic iron-sulfur protein assembly protein Ciao1

target	prob	fident	alnlen	evalue	theader
6tbn-assembly1.cif.gz_B	1	0.102	460	1.97e-13	Crystal structure of CIAO1-CIAO2B CIA core complex
5yzv-assembly1.cif.gz_B	1	0.13	436	2.857e-13	Biophysical and structural characterization of the thermostable WD40 domain of a prokaryotic protein, Thermomonospora curvata PkwA
7mqa-assembly1.cif.gz_LQ	1	0.128	443	3.726e-13	Cryo-EM structure of the human SSU processome, state post-A1

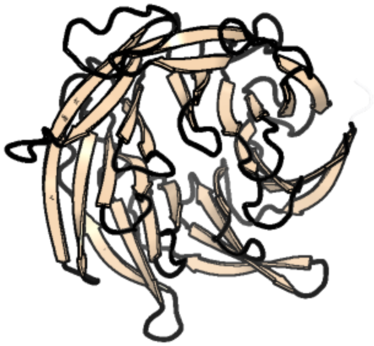

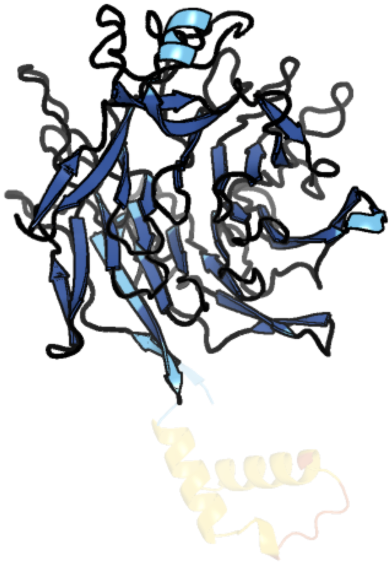

Figure 137: left: reference structure of 6tbn chain B. right: predicted structure of chlorv-1..137, unaligned sequences are shown as transparent

chlorv-1..138

- Sequence-based annotation for chlorv-1..138 is hypothetical protein
- Best hit was 7qi3 chain C: Arylamine N-acetyltransferase

target	prob	fident	alnlen	evalue	theader
7qi3-assembly1.cif.gz_C	1	0.099	291	7.615e-08	Structure of Fusarium verticillioides NAT1 (FDB2) N-malonyltransferase
4dmo-assembly2.cif.gz_B	1	0.142	273	1.185e-07	Crystal structure of the (BACCR)NAT3 arylamine N-acetyltransferase from Bacillus cereus reveals a unique Cys-His-Glu catalytic triad
4b55-assembly1.cif.gz_A	1	0.093	289	4.726e-07	Crystal Structure of the Covalent Adduct Formed between Mycobacterium marinum Aryalamine N-acetyltransferase and Phenyl vinyl ketone a derivative of Piperidinols

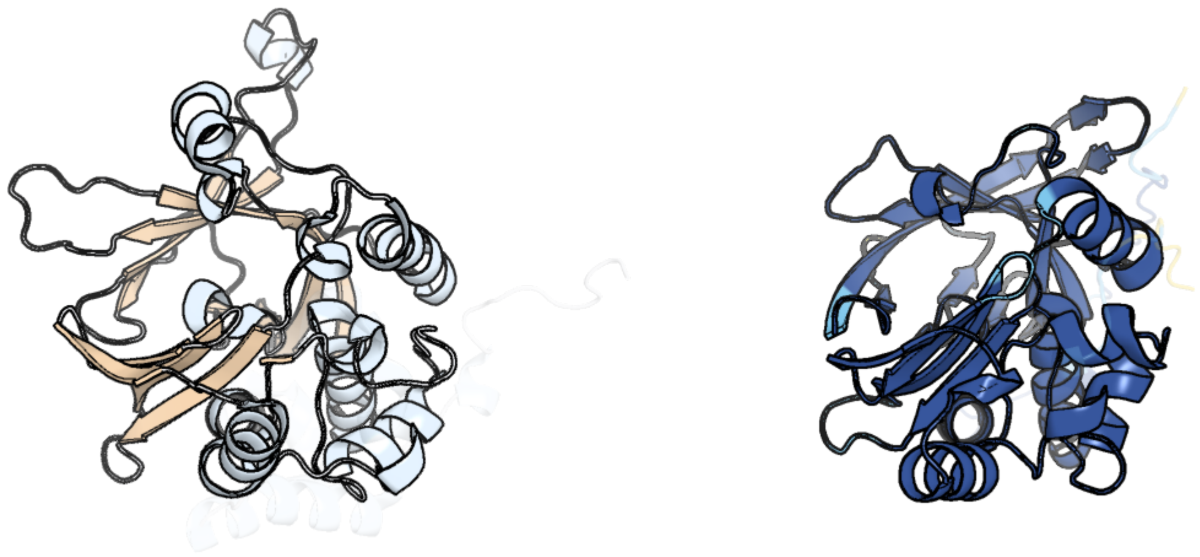

Figure 138: left: reference structure of 7qi3 chain C. right: predicted structure of chlorv-1..138, unaligned sequences are shown as transparent

chlorv-1..139

- Sequence-based annotation for chlorv-1..139 is putative Peptidase
- Best hit was 5ilb chain A: Protease Do-like 2, chloroplastic,Protease Do-like 9

target	prob	fident	alnlen	evalue	theader
5ilb-assembly1.cif.gz_A	1	0.207	473	3.657e-32	Crystal structure of protease domain of Deg2 linked with the PDZ domain of Deg9
5il9-assembly1.cif.gz_A	1	0.208	466	9.676e-31	Crystal structure of Deg9
5il9-assembly1.cif.gz_B	1	0.204	459	1.343e-30	Crystal structure of Deg9

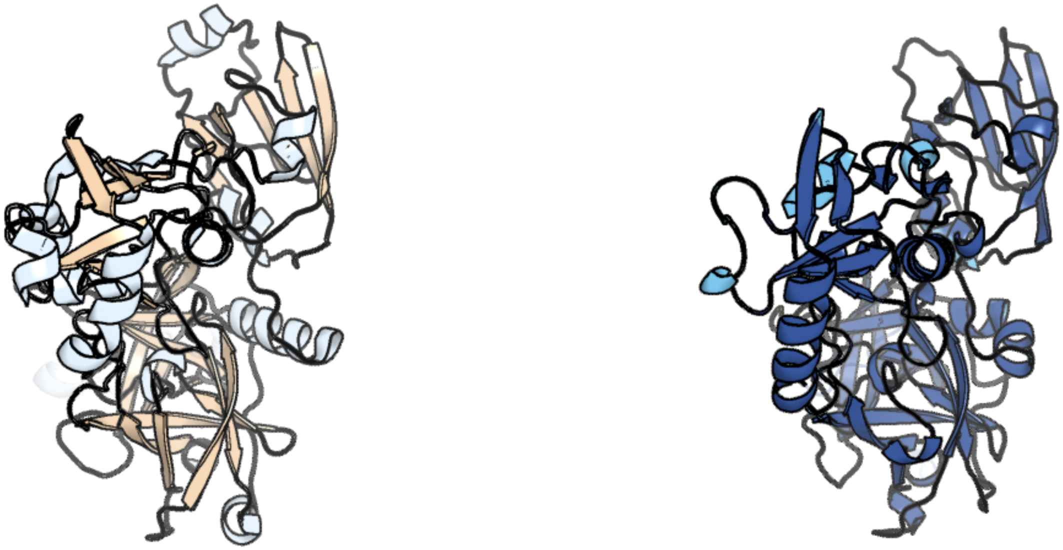

Figure 139: left: reference structure of 5ilb chain A. right: predicted structure of chlorv-1..139, unaligned sequences are shown as transparent

chlorv-1..140

- Sequence-based annotation for chlorv-1..140 is putative Methyltransferases
- Best hit was 4ckc chain D: MRNA-CAPPING ENZYME CATALYTIC SUBUNIT

target	prob	fident	alnlen	evalue	theader
4ckc-assembly2.cif.gz_D	1	0.132	696	2.412e-12	Vaccinia virus capping enzyme complexed with SAH (monoclinic form)
4ckc-assembly1.cif.gz_A	1	0.134	656	3.261e-12	Vaccinia virus capping enzyme complexed with SAH (monoclinic form)
4ckb-assembly2.cif.gz_A	1	0.132	679	9.858e-12	Vaccinia virus capping enzyme complexed with GTP and SAH

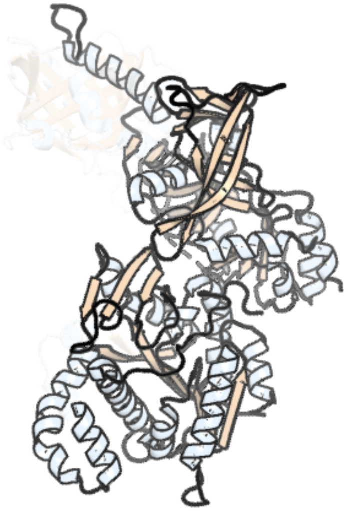

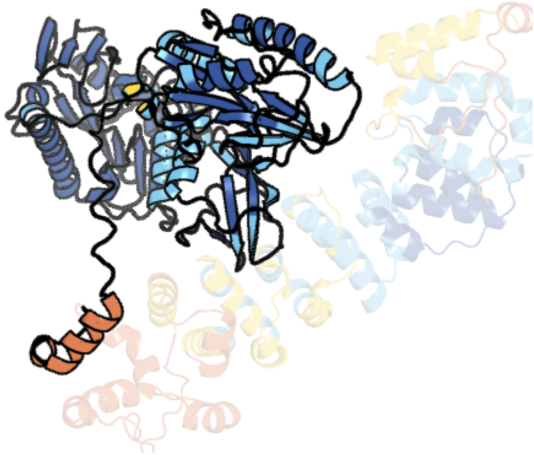

Figure 140: left: reference structure of 4ckc chain D. right: predicted structure of chlorv-1..140, unaligned sequences are shown as transparent

chlorv-1..141

- Sequence-based annotation for chlorv-1..141 is hypothetical protein
- No significant structural hit found

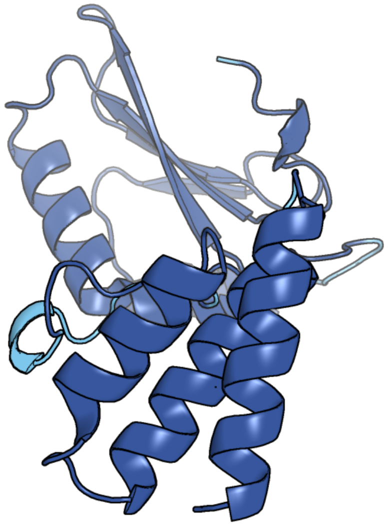

Figure 141: predicted structure of chlorv-1..141

chlorv-1..142

- Sequence-based annotation for chlorv-1..142 is putative Nuclease
- Best hit was 6bt1 chain A: Nocturnin

target	prob	fident	alnlen	evalue	theader
6bt1-assembly1.cif.gz_A	1	0.156	345	4.806e-13	Structure of the human Nocturnin catalytic domain
6bt2-assembly2.cif.gz_B	1	0.134	342	2.259e-12	Structure of the human Nocturnin catalytic domain with bound sulfate anion
6mal-assembly1.cif.gz_A	1	0.154	337	6.214e-12	Structure of human Nocturnin C-terminal domain

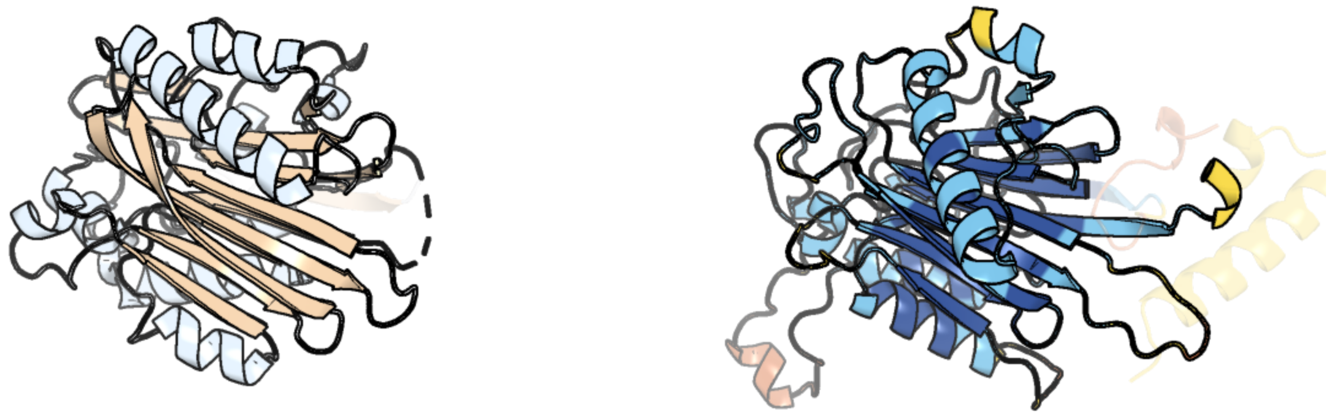

Figure 142: left: reference structure of 6bt1 chain A. right: predicted structure of chlorv-1..142, unaligned sequences are shown as transparent

chlorv-1..143

- Sequence-based annotation for chlorv-1..143 is hypothetical protein
- No significant structural hit found

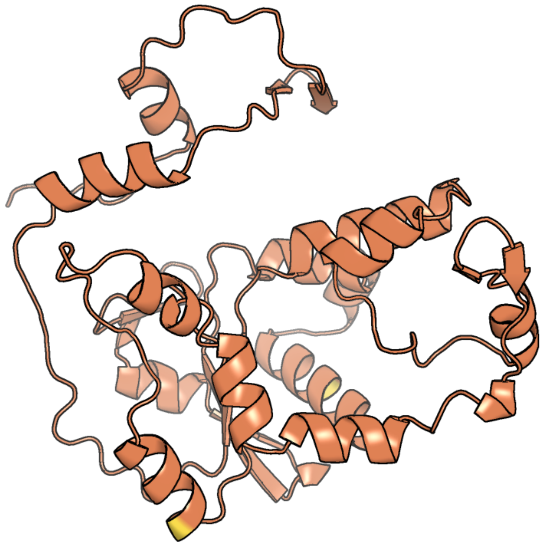

Figure 143: predicted structure of chlorv-1..143

chlorv-1..144

- Sequence-based annotation for chlorv-1..144 is hypothetical protein
- No significant structural hit found

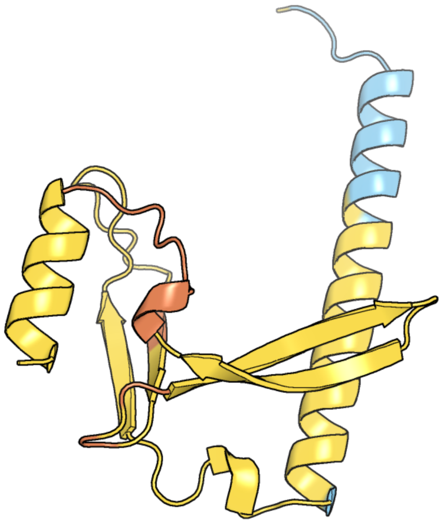

Figure 144: predicted structure of chlorv-1..144

chlorv-1..145

- Sequence-based annotation for chlorv-1..145 is putative Aminotransferase
- Best hit was 7lk1 chain A: Ornithine aminotransferase, mitochondrial

target	prob	fident	alnlen	evalue	theader
7lk1-assembly1.cif.gz_A	1	0.517	402	2.951e-55	Ornithine Aminotransferase (OAT) with its potent inhibitor - (S)-3-amino-4,4-difluorocyclopent-1-enecarboxylic acid (SS-1-148) - 1 Hour Soaking
2byj-assembly2.cif.gz_C-2	1	0.504	406	4.632e-55	Ornithine aminotransferase mutant Y85I
6hx7-assembly3.cif.gz_C-3	1	0.512	400	6.14e-55	Crystal structure of human R180T variant of ORNITHINE AMINOTRANSFERASE at 1.8 Angstrom

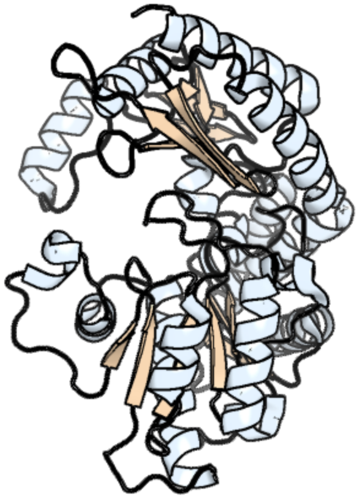

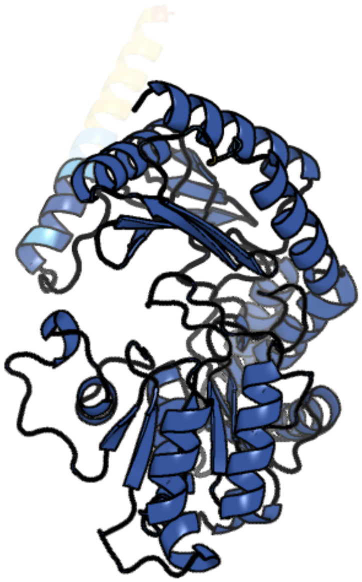

Figure 145: left: reference structure of 7lk1 chain A. right: predicted structure of chlorv-1..145, unaligned sequences are shown as transparent

chlorv-1..146

- Sequence-based annotation for chlorv-1..146 is hypothetical protein
- No significant structural hit found

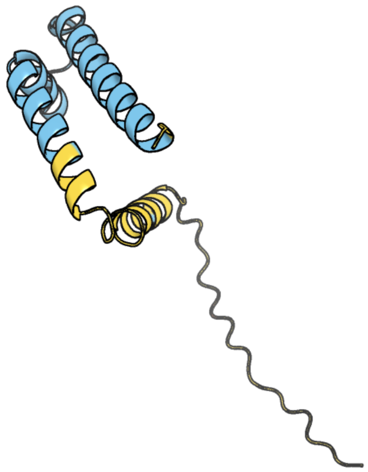

Figure 146: predicted structure of chlorv-1..146

chlorv-1..147

- Sequence-based annotation for chlorv-1..147 is putative DUF5760 domain-containing protein
- No significant structural hit found

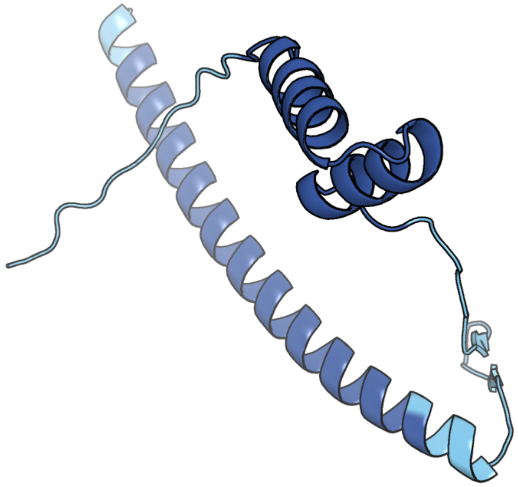

Figure 147: predicted structure of chlorv-1..147

chlorv-1..148

- Sequence-based annotation for chlorv-1..148 is hypothetical protein
- Best hit was 7eyr chain B: 2-oxoglutarate/Fe(II)-dependent dioxygenase SptF

target	prob	fident	alnlen	evalue	theader
7eyr-assembly2.cif.gz_B-3	1	0.124	258	3.615e-07	Fe(II)/(alpha)ketoglutarate-dependent dioxygenase SptF apo
7eyw-assembly1.cif.gz_B	1	0.121	205	4.091e-07	Fe(II)/(alpha)ketoglutarate-dependent dioxygenase SptF with terretonin C
7eyr-assembly1.cif.gz_D-2	1	0.125	240	4.629e-07	Fe(II)/(alpha)ketoglutarate-dependent dioxygenase SptF apo

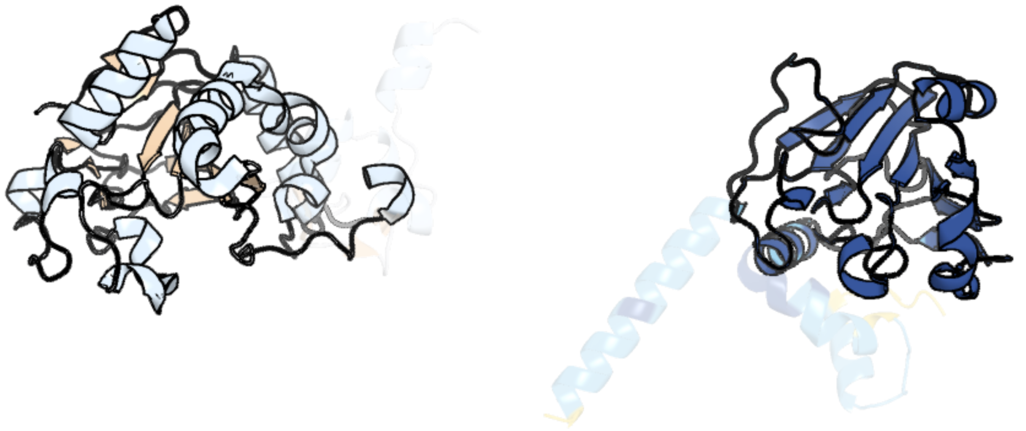

Figure 148: left: reference structure of 7eyr chain B. right: predicted structure of chlorv-1..148, unaligned sequences are shown as transparent

chlorv-1..149

- Sequence-based annotation for chlorv-1..149 is hypothetical protein
- No significant structural hit found

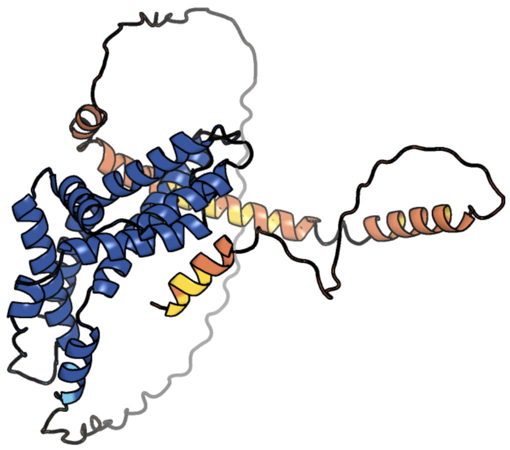

Figure 149: predicted structure of chlorv-1..149

chlorv-1..150

- Sequence-based annotation for chlorv-1..150 is putative packaging ATPase/Poxvirus A32 protein
- Best hit was 4kfs chain B: Genome packaging NTPase B204

target	prob	fident	alnlen	evalue	theader
4kfs-assembly2.cif.gz_B	1	0.168	219	2.915e-09	Structure of the genome packaging NTPase B204 from Sulfolobus turreted icosahedral virus 2 in complex with AMP
4kfs-assembly1.cif.gz_A	1	0.147	237	4.497e-09	Structure of the genome packaging NTPase B204 from Sulfolobus turreted icosahedral virus 2 in complex with AMP
4kfr-assembly3.cif.gz_C	1	0.152	236	6.129e-09	Structure of the genome packaging NTPase B204 from Sulfolobus turreted icosahedral virus 2 in complex with sulfate

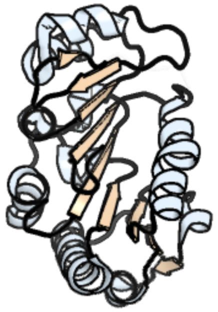

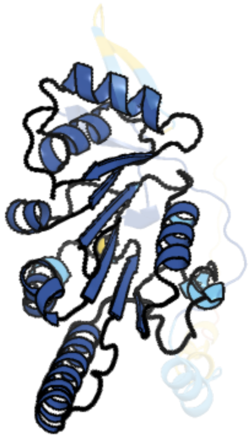

Figure 150: left: reference structure of 4kfs chain B. right: predicted structure of chlorv-1..150, unaligned sequences are shown as transparent

chlorv-1..151

- Sequence-based annotation for chlorv-1..151 is hypothetical protein
- No significant structural hit found

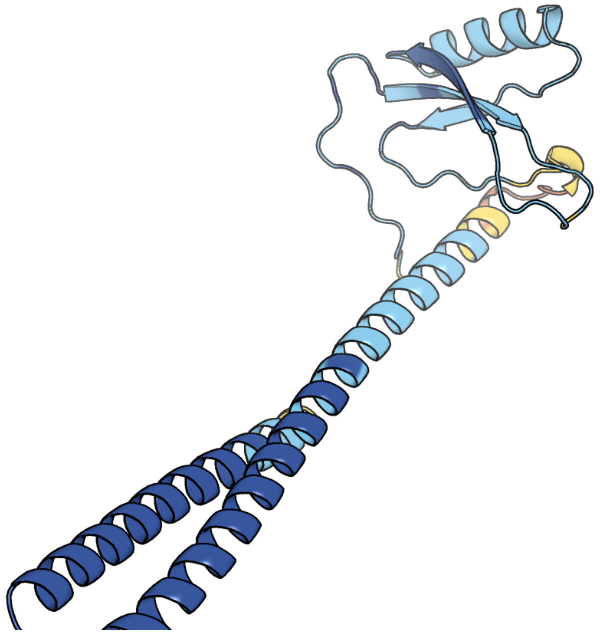

Figure 151: predicted structure of chlorv-1..151

chlorv-1..152

- Sequence-based annotation for chlorv-1..152 is putative Poxvirus Late Transcription Factor VLTF3
- No significant structural hit found

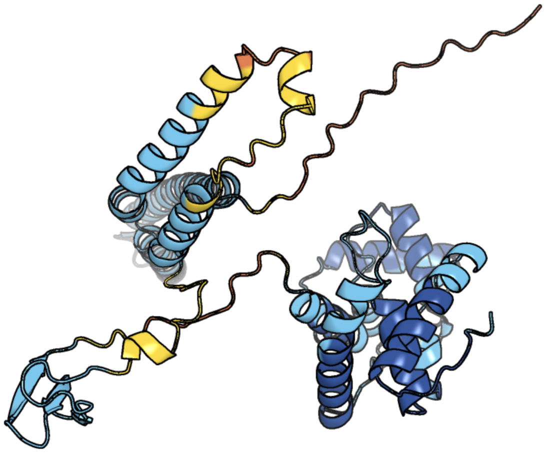

Figure 152: predicted structure of chlorv-1..152

chlorv-1..153

- Sequence-based annotation for chlorv-1..153 is putative Major capsid protein
- Best hit was 8h2i chain ab: Major capsid protein (MCP)

target	prob	fident	alnlen	evalue	theader
8h2i-assembly1.cif.gz_ab-5	1	0.35	550	4.038e-46	Near-atomic structure of five-fold averaged PBCV-1 capsid
6ncl-assembly1.cif.gz_d0	1	0.352	550	1.603e-45	Near-atomic structure of icosahedrally averaged PBCV-1 capsid
3kk5-assembly1.cif.gz_A	1	0.328	532	3.621e-42	Crystal structure of PBCV-1 VP54 fitted into a cryo-EM reconstruction of the virophage Sputnik

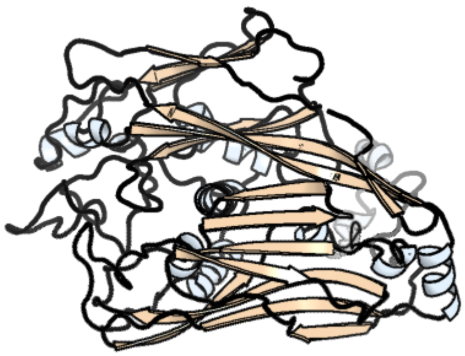

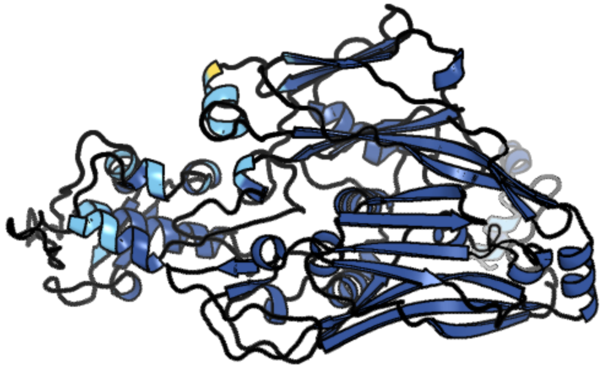

Figure 153: left: reference structure of 8h2i chain ab. right: predicted structure of chlorv-1..153, unaligned sequences are shown as transparent

chlorv-1..154

- Sequence-based annotation for chlorv-1..154 is putative HNH endonuclease
- No significant structural hit found

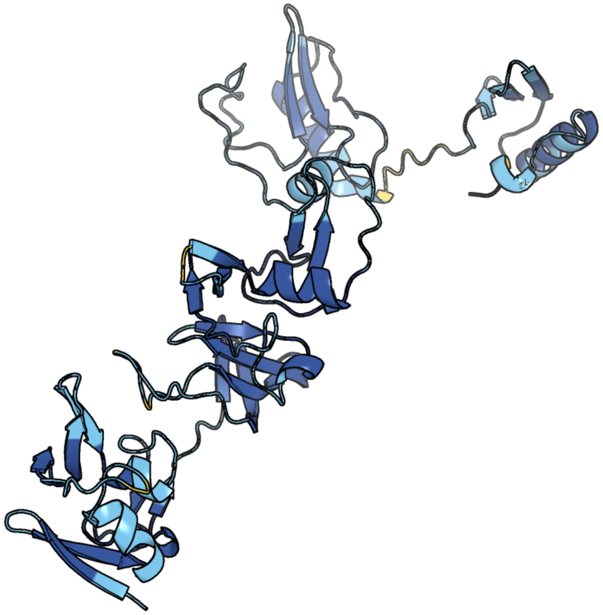

Figure 154: predicted structure of chlorv-1..154

chlorv-1..155

- Sequence-based annotation for chlorv-1..155 is hypothetical protein
- Best hit was 1qma chain A: NUCLEAR TRANSPORT FACTOR 2

target	prob	fident	alnlen	evalue	theader
1qma-assembly1.cif.gz_A	1	0.136	117	2.886e-05	Nuclear Transport Factor 2 (NTF2) W7A mutant
2r4i-assembly1.cif.gz_B	1	0.139	122	3.882e-05	CRYSTAL STRUCTURE OF A NTF2-LIKE PROTEIN (CHU_1428) FROM CYTOPHAGA HUTCHINSONII ATCC 33406 AT 1.60 A RESOLUTION
3nv0-assembly1.cif.gz_B	1	0.108	138	4.371e-05	Crystal structure and mutational analysis of the NXF2/NXT1 heterodimeric complex from caenorhabditis elegans at 1.84 A resolution

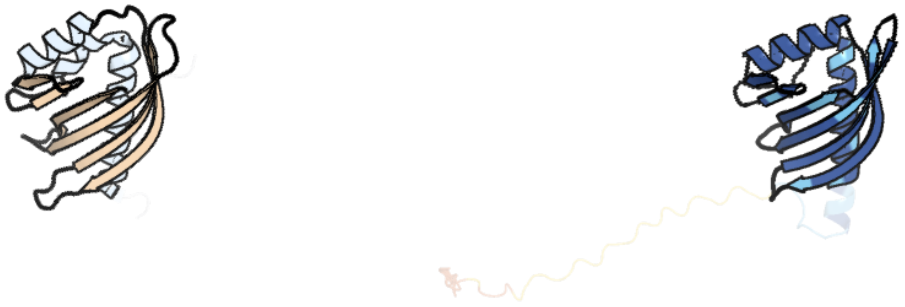

Figure 155: left: reference structure of 1qma chain A. right: predicted structure of chlorv-1..155, unaligned sequences are shown as transparent

chlorv-1..156

- Sequence-based annotation for chlorv-1..156 is putative Dioxygenase
- Best hit was 4z82 chain A: Cysteine dioxygenase type 1

target	prob	fident	alnlen	evalue	theader
4z82-assembly1.cif.gz_A	1	0.271	114	2.365e-08	Cysteine bound rat cysteine dioxygenase C164S variant at pH 8.1
5i0u-assembly1.cif.gz_A	1	0.274	113	8.477e-08	Incompletely interpreted D-cysteine soak of Cysteine Dioxygenase at pH 7.0
6u4l-assembly1.cif.gz_A	1	0.25	116	9.944e-08	cysteine dioxygenase variant - C93E

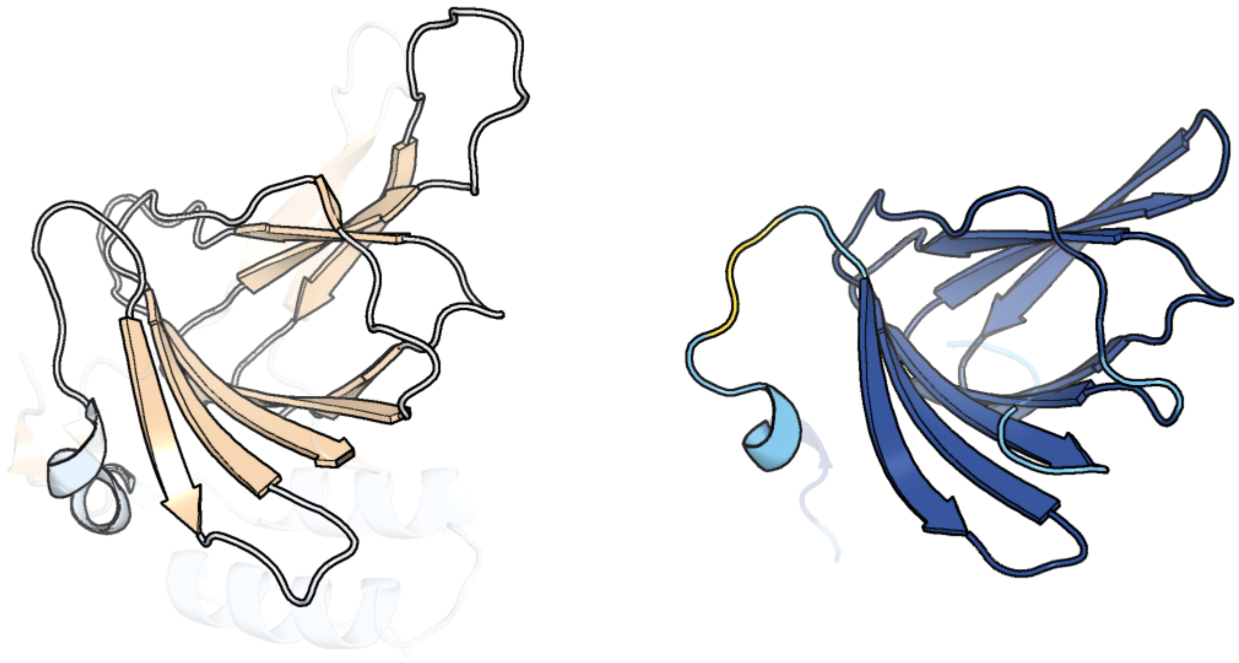

Figure 156: left: reference structure of 4z82 chain A. right: predicted structure of chlorv-1..156, unaligned sequences are shown as transparent

chlorv-1..157

- Sequence-based annotation for chlorv-1..157 is putative DUF2177 domain-containing membrane protein
- No significant structural hit found

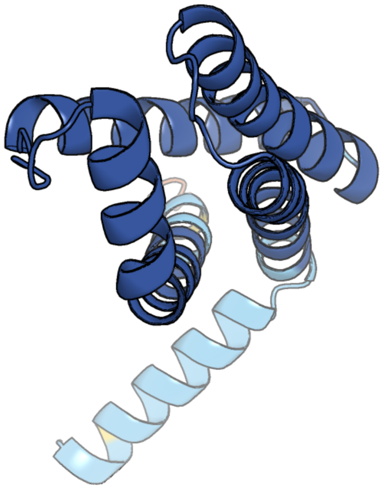

Figure 157: predicted structure of chlorv-1..157

chlorv-1..158

- Sequence-based annotation for chlorv-1..158 is hypothetical protein
- No significant structural hit found

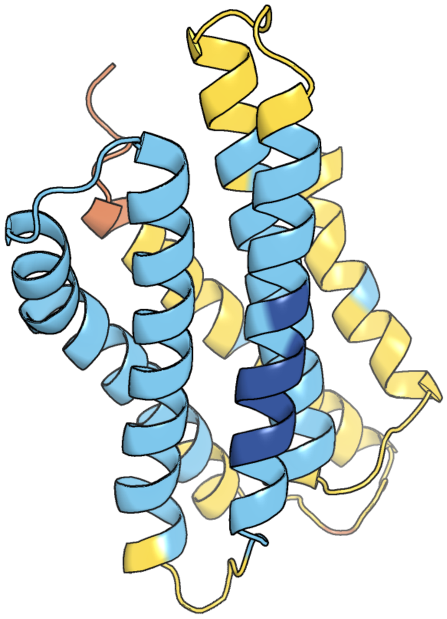

Figure 158: predicted structure of chlorv-1..158

chlorv-1..159

- Sequence-based annotation for chlorv-1..159 is hypothetical protein
- No significant structural hit found

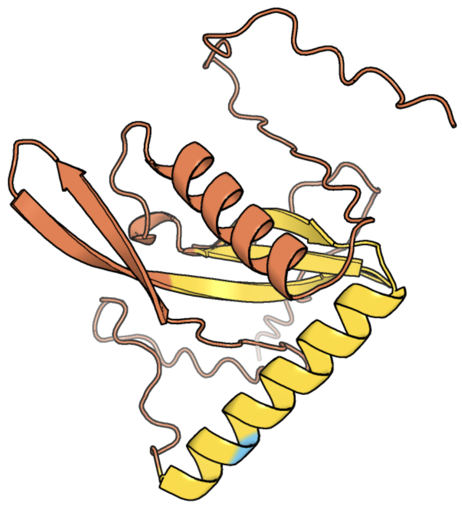

Figure 159: predicted structure of chlorv-1..159

chlorv-1..160

- Sequence-based annotation for chlorv-1..160 is putative Major capsid protein
- Best hit was 8rbs chain A1: Major capsid protein

target	prob	fident	alnlen	evalue	theader
8rbs-assembly1.cif.gz__A1	1	0.145	681	3.083e-29	Emiliania huxleyi virus 201 (EhV-201) asymmetrical unit of capsid proteins predicted by AlphaFold2 fitted into the cryo-EM density of EhV-201 virion composite map.
8h2i-assembly1.cif.gz__ab-5	1	0.147	704	6.205e-26	Near-atomic structure of five-fold averaged PBCV-1 capsid
6ncl-assembly1.cif.gz__d0	1	0.14	705	2.533e-25	Near-atomic structure of icosahedrally averaged PBCV-1 capsid

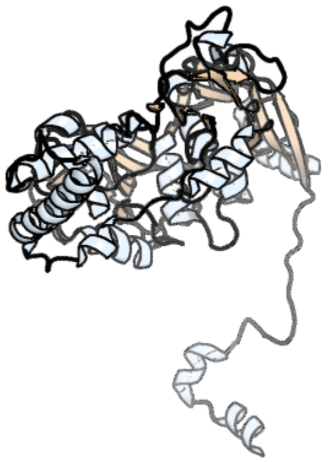

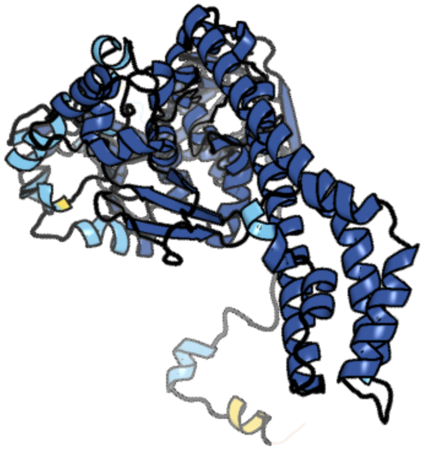

Figure 160: left: reference structure of 8rbs chain A1. right: predicted structure of chlorv-1..160, unaligned sequences are shown as transparent

chlorv-1..161

- Sequence-based annotation for chlorv-1..161 is hypothetical protein
- No significant structural hit found

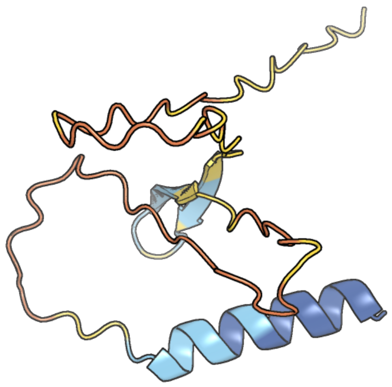

Figure 161: predicted structure of chlorv-1..161

chlorv-1..162

- Sequence-based annotation for chlorv-1..162 is hypothetical protein
- No significant structural hit found

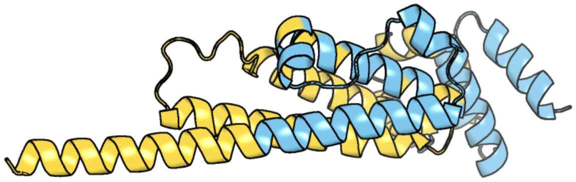

Figure 162: predicted structure of chlorv-1..162

chlorv-1..163

- Sequence-based annotation for chlorv-1..163 is putative Capsid protein
- Best hit was 1m4x chain A: PBCV-1 virus capsid

target	prob	fident	alnlen	evalue	theader
3kk5-assembly1.cif.gz_A	1	0.18	433	2.876e-18	Crystal structure of PBCV-1 VP54 fitted into a cryo-EM reconstruction of the virophage Sputnik
1m4x-assembly1.cif.gz_A	1	0.182	427	3.619e-18	PBCV-1 virus capsid, quasi-atomic model
8h2i-assembly1.cif.gz_ab-5	1	0.188	429	4.554e-18	Near-atomic structure of five-fold averaged PBCV-1 capsid

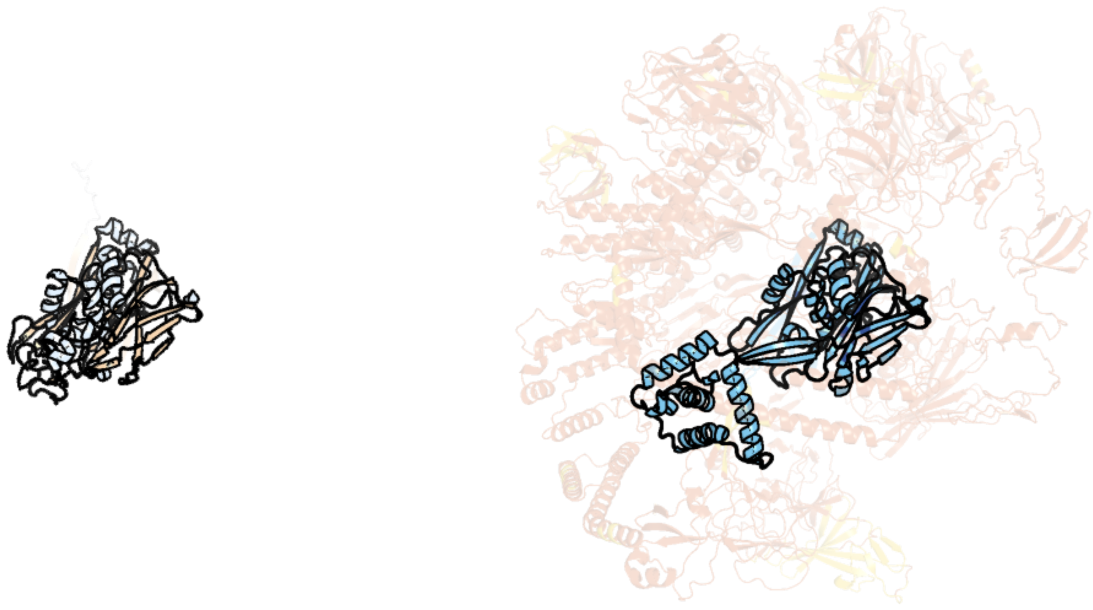

Figure 163: left: reference structure of 1m4x chain A. right: predicted structure of chlorv-1..163, unaligned sequences are shown as transparent

chlorv-1..164

- Sequence-based annotation for chlorv-1..164 is putative replication factor C small subunit 2
- Best hit was 7tku chain E: Replication factor C subunit 5

target	prob	fident	alnlen	evalue	theader
7tku-assembly1.cif.gz_E	1	0.233	343	4.754e-20	Structure of the yeast clamp loader (Replication Factor C RFC) bound to the open sliding clamp (Proliferating Cell Nuclear Antigen PCNA)
8dr5-assembly1.cif.gz_E	1	0.261	329	1.268e-19	Open state of RFC:PCNA bound to a 3' ss/dsDNA junction (DNA2) with NTD
7thv-assembly1.cif.gz_E	1	0.258	325	3.748e-19	Structure of the yeast clamp loader (Replication Factor C RFC) bound to the sliding clamp (Proliferating Cell Nuclear Antigen PCNA) in an autoinhibited conformation

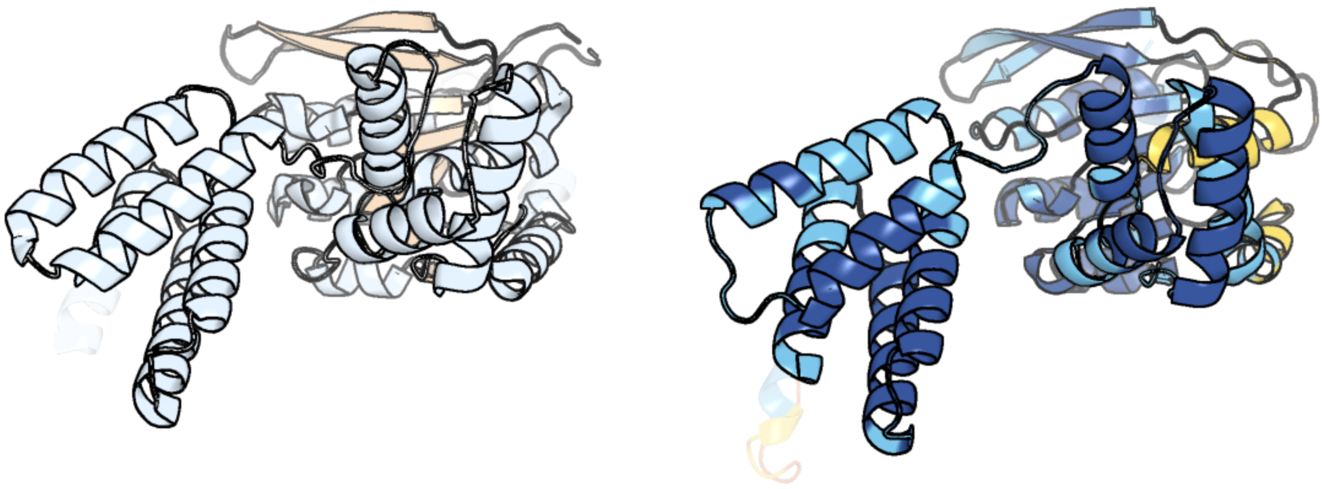

Figure 164: left: reference structure of 7tku chain E. right: predicted structure of chlorv-1..164, unaligned sequences are shown as transparent

chlorv-1..165

- Sequence-based annotation for chlorv-1..165 is putative Protein kinase
- No significant structural hit found

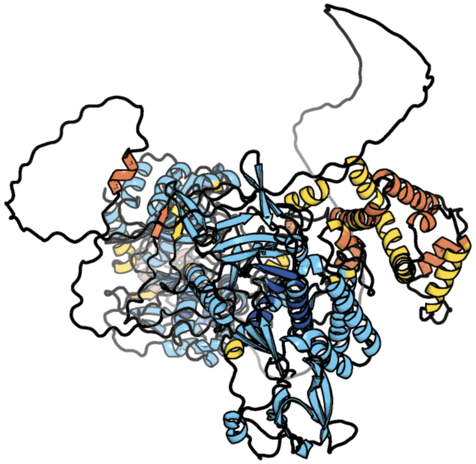

Figure 165: predicted structure of chlorv-1..165

chlorv-1..166

- Sequence-based annotation for chlorv-1..166 is putative DUF5761 domain-containing protein
- Best hit was 6ncl chain c9: P8

target	prob	fident	alnlen	evalue	theader
6ncl-assembly1.cif.gz_c9	1	0.25	136	5.349e-05	Near-atomic structure of icosahedrally averaged PBCV-1 capsid
5ku9-assembly2.cif.gz_B	0.196	0.139	136	0.701	Crystal structure of MCL1 with compound 1
5f3q-assembly1.cif.gz_B	0.063	0.115	147	1.201	Crystal structure of a noncanonical Dicer protein from Entamoeba histolytica

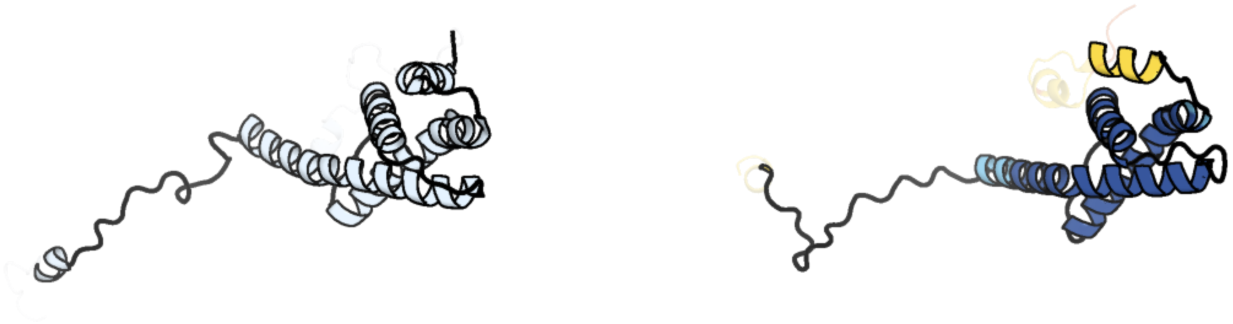

Figure 166: left: reference structure of 6ncl chain c9. right: predicted structure of chlorv-1..166, unaligned sequences are shown as transparent

chlorv-1..167

- Sequence-based annotation for chlorv-1..167 is putative Poxvirus P4B major core protein
- Best hit was 5chv chain B: Ubl carboxyl-terminal hydrolase 18

target	prob	fident	alnlen	evalue	theader
5chv-assembly2.cif.gz_B	1	0.085	373	3.998e-08	Crystal structure of USP18-ISG15 complex
2y6e-assembly1.cif.gz_C	1	0.128	404	4.881e-08	Structure of the D1D2 domain of USP4, the conserved catalytic domain
5l8h-assembly1.cif.gz_A	1	0.084	380	5.131e-08	Structure of USP46-UbVME

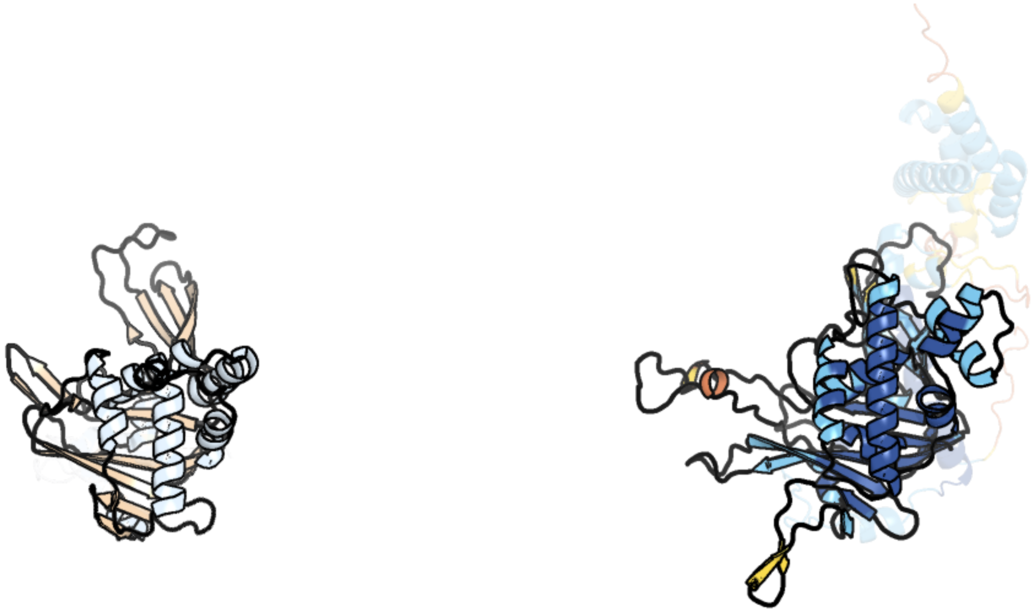

Figure 167: left: reference structure of 5chv chain B. right: predicted structure of chlorv-1..167, unaligned sequences are shown as transparent

chlorv-1..168

- Sequence-based annotation for chlorv-1..168 is hypothetical protein
- Best hit was 8dr5 chain A: Replication factor C subunit 1

target	prob	fident	alnlen	evalue	theader
8dr5-assembly1.cif.gz_A	1	0.197	491	4.046e-17	Open state of RFC:PCNA bound to a 3' ss/dsDNA junction (DNA2) with NTD
8dr0-assembly1.cif.gz_A	1	0.189	491	5.47e-17	Closed state of RFC:PCNA bound to a 3' ss/dsDNA junction
8dr3-assembly1.cif.gz_A	1	0.203	482	9.508e-17	Closed state of RFC:PCNA bound to a 3' ss/dsDNA junction (DNA2) with NTD

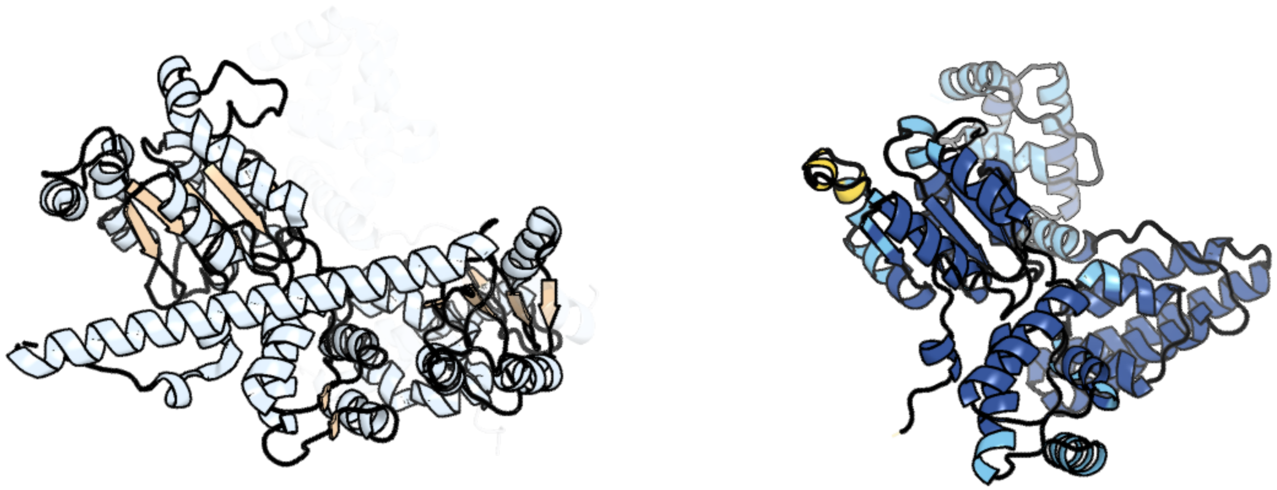

Figure 168: left: reference structure of 8dr5 chain A. right: predicted structure of chlorv-1..168, unaligned sequences are shown as transparent

chlorv-1..169

- Sequence-based annotation for chlorv-1..169 is hypothetical protein
- No significant structural hit found

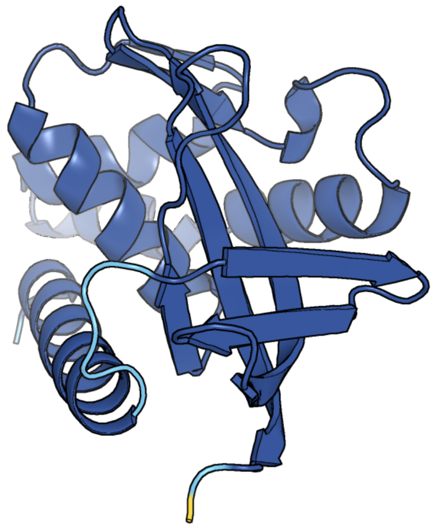

Figure 169: predicted structure of chlorv-1..169

chlorv-1..170

- Sequence-based annotation for chlorv-1..170 is hypothetical protein
- No significant structural hit found

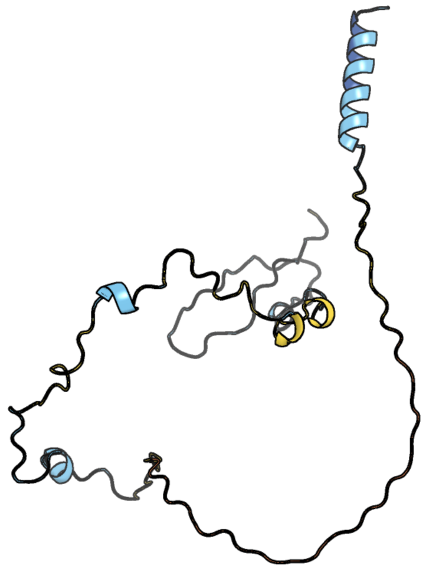

Figure 170: predicted structure of chlorv-1..170

chlorv-1..171

- Sequence-based annotation for chlorv-1..171 is putative ELP3-like acetyltransferase
- Best hit was 6iad chain A: Histone acetyltransferase, ELP3 family

target	prob	fident	alnlen	evalue	theader
6iad-assembly1.cif.gz_A	1	0.36	452	4.67e-37	Apo crystal structure of archaeal Methanocaldococcus infernus Elp3 (del1-54)
8asw-assembly1.cif.gz_C	1	0.32	453	2.651e-36	Cryo-EM structure of yeast Elp123 in complex with alanine tRNA
8asv-assembly1.cif.gz_C	1	0.272	587	5.807e-36	Cryo-EM structure of yeast Elongator complex

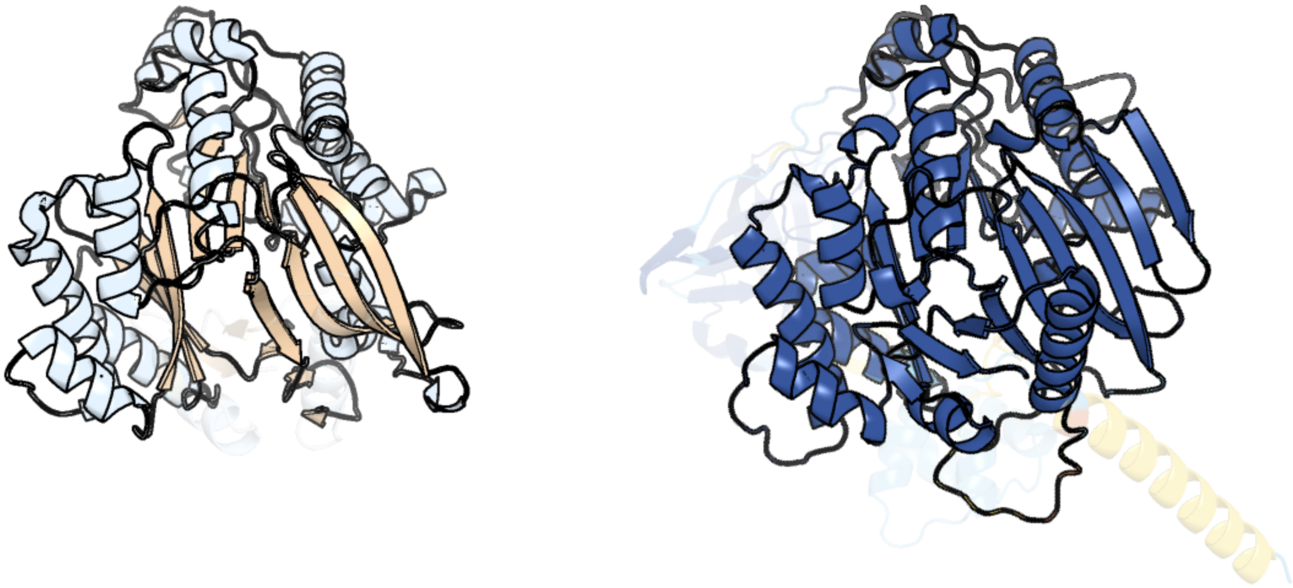

Figure 171: left: reference structure of 6iad chain A. right: predicted structure of chlorv-1..171, unaligned sequences are shown as transparent

chlorv-1..172

- Sequence-based annotation for chlorv-1..172 is putative Patatin-like phospholipase
- Best hit was 5fya chain A: PATATIN-LIKE PROTEIN, PLPD

target	prob	fident	alnlen	evalue	theader
5fya-assembly1.cif.gz_A	1	0.187	283	3.529e-14	Cubic crystal of the native PlpD
5fqu-assembly1.cif.gz_A	1	0.184	276	1.252e-13	Orthorhombic crystal structure of of PlpD (selenomethionine derivative)
5fya-assembly1.cif.gz_B	1	0.187	283	1.669e-13	Cubic crystal of the native PlpD

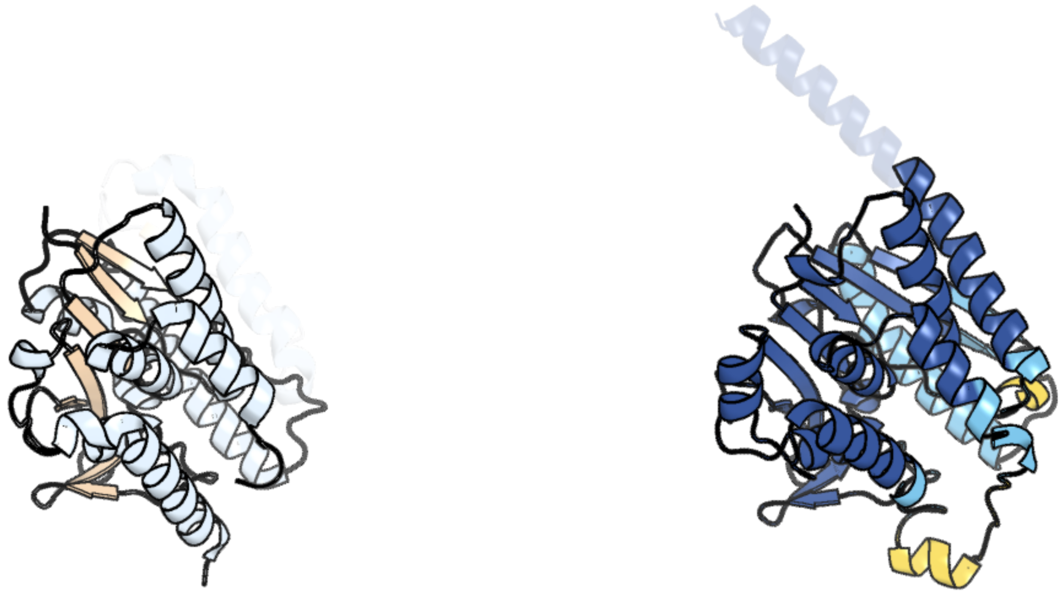

Figure 172: left: reference structure of 5fya chain A. right: predicted structure of chlorv-1..172, unaligned sequences are shown as transparent

chlorv-1..173

- Sequence-based annotation for chlorv-1..173 is putative DnaJ domain
- No significant structural hit found

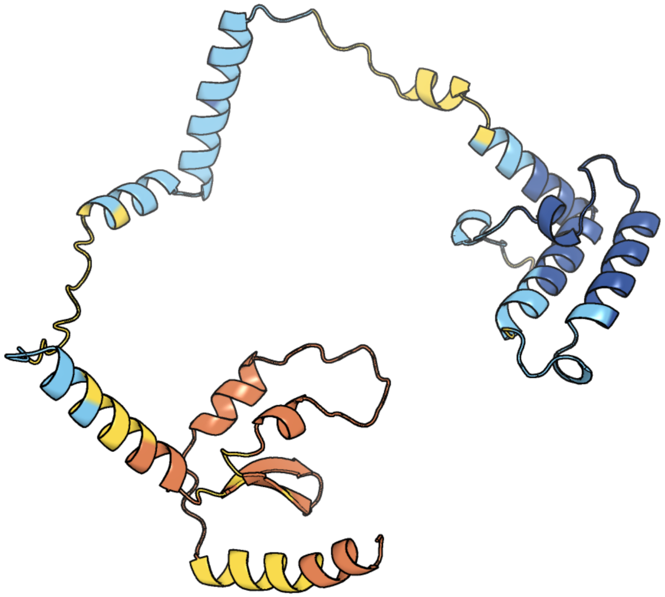

Figure 173: predicted structure of chlorv-1..173

chlorv-1..174

- Sequence-based annotation for chlorv-1..174 is putative ADP-ribosylglycohydrolase
- Best hit was 3hfw chain A: Protein ADP-ribosylarginine hydrolase

target	prob	fident	alnlen	evalue	theader
3hfw-assembly1.cif.gz_A	1	0.231	371	2.68e-14	Crystal Structure of human ADP-ribosylhydrolase 1 (hARH1)
7aks-assembly2.cif.gz_CCC	1	0.167	376	7.6e-11	Human ADP-ribosylserine hydrolase ARH3 mutant E41A in complex with H2B-S7-mar peptide
6g1p-assembly1.cif.gz_A	1	0.169	377	3.123e-10	Apo form of ADP-ribosylserine hydrolase ARH3 of Latimeria chalumnae

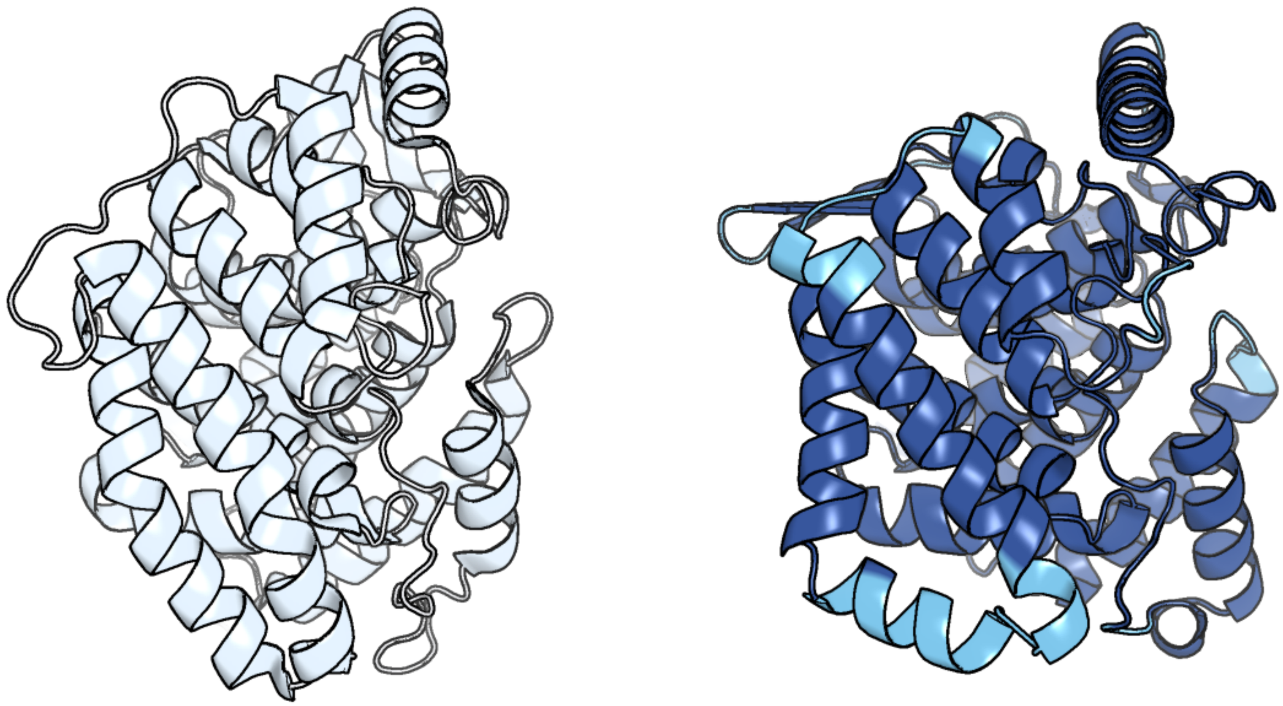

Figure 174: left: reference structure of 3hfw chain A. right: predicted structure of chlorv-1..174, unaligned sequences are shown as transparent

chlorv-1..175

- Sequence-based annotation for chlorv-1..175 is hypothetical protein
- No significant structural hit found

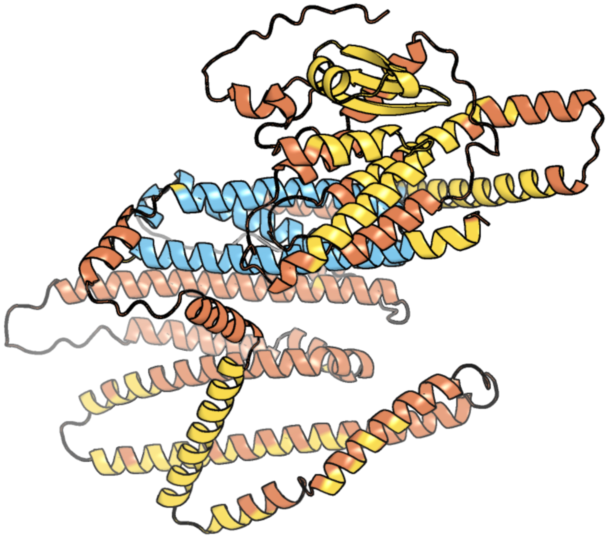

Figure 175: predicted structure of chlorv-1..175

chlorv-1..176

- Sequence-based annotation for chlorv-1..176 is hypothetical protein
- No significant structural hit found

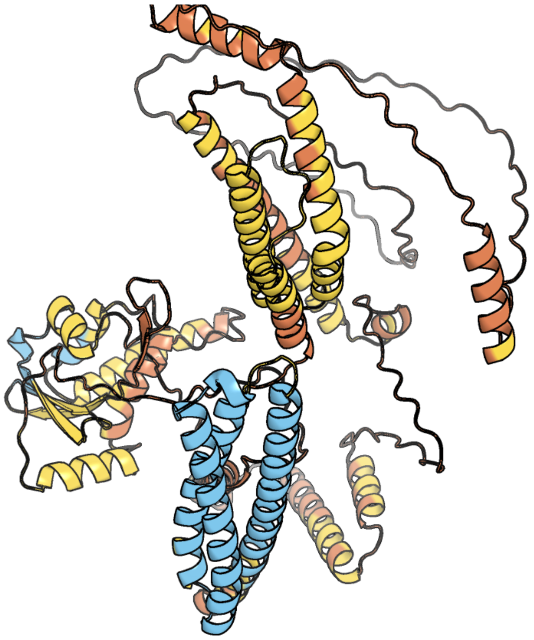

Figure 176: predicted structure of chlorv-1..176

chlorv-1..177

- Sequence-based annotation for chlorv-1..177 is hypothetical protein
- No significant structural hit found

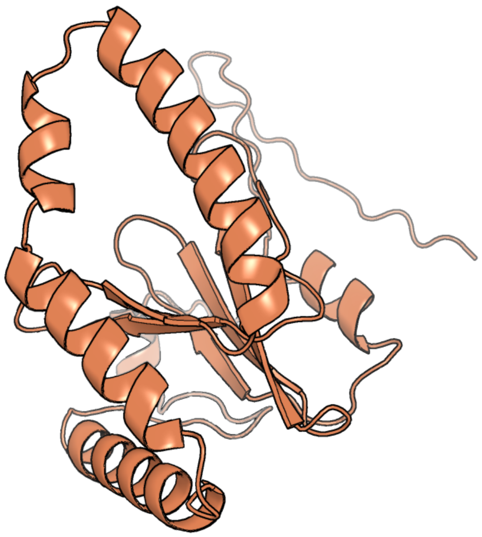

Figure 177: predicted structure of chlorv-1..177

chlorv-1..178

- Sequence-based annotation for chlorv-1..178 is putative Major capsid protein
- Best hit was 8rbs chain A1: Major capsid protein

target	prob	fident	alnlen	evalue	theader
8rbs-assembly1.cif.gz__A1	1	0.206	561	7.748e-36	Emiliania huxleyi virus 201 (EhV-201) asymmetrical unit of capsid proteins predicted by AlphaFold2 fitted into the cryo-EM density of EhV-201 virion composite map.
8h2i-assembly1.cif.gz__ab-5	1	0.235	530	1.655e-35	Near-atomic structure of five-fold averaged PBCV-1 capsid
8h2i-assembly1.cif.gz__bu	1	0.229	593	2.554e-35	Near-atomic structure of five-fold averaged PBCV-1 capsid

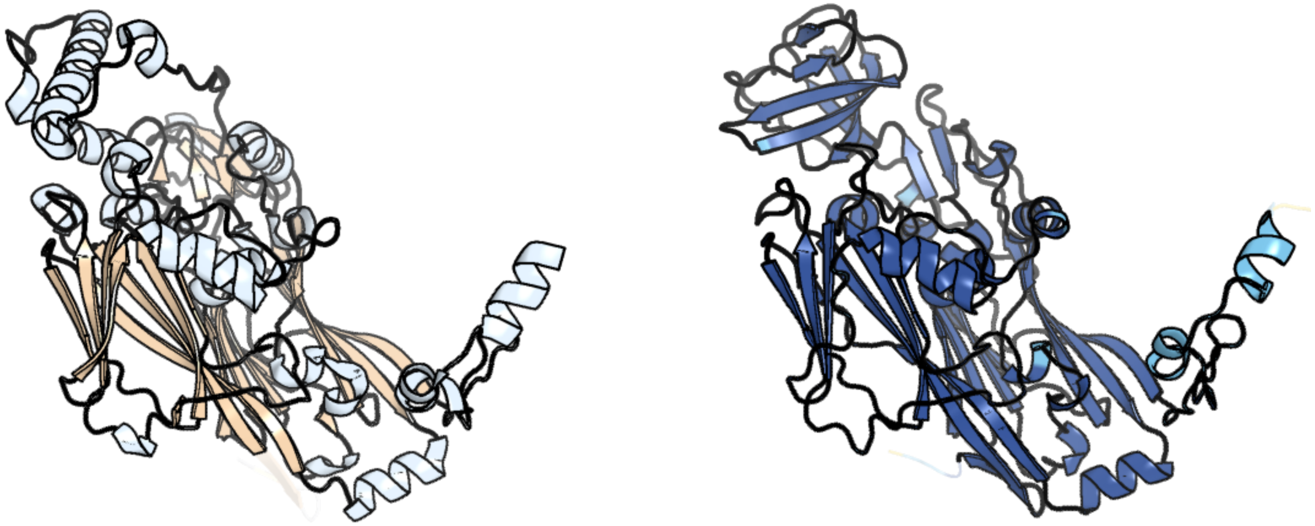

Figure 178: left: reference structure of 8rbs chain A1. right: predicted structure of chlorv-1..178, unaligned sequences are shown as transparent

chlorv-1..179

- Sequence-based annotation for chlorv-1..179 is putative Oxygenase
- Best hit was 3btz chain A: Alpha-ketoglutarate-dependent dioxygenase alkB homolog 2

target	prob	fident	alnlen	evalue	theader
3btz-assembly1.cif.gz_A	1	0.211	199	4.043e-09	Crystal structure of human ABH2 cross-linked to dsDNA
3rzl-assembly1.cif.gz_A	1	0.208	182	1.04e-08	Duplex Interrogation by a Direct DNA Repair Protein in the Search of Damage
3buc-assembly1.cif.gz_A	1	0.194	190	1.162e-08	X-ray structure of human ABH2 bound to dsDNA with Mn(II) and 2KG

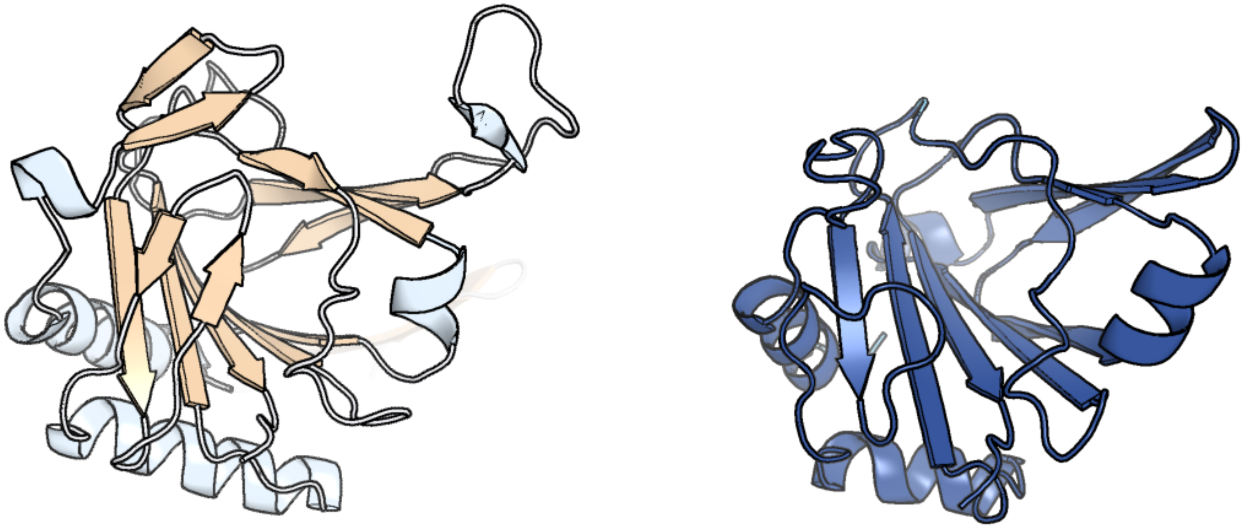

Figure 179: left: reference structure of 3btz chain A. right: predicted structure of chlorv-1..179, unaligned sequences are shown as transparent

chlorv-1..180

- Sequence-based annotation for chlorv-1..180 is putative Dihydrofolate reductase
- Best hit was 6n1s chain A: Bifunctional dihydrofolate reductase-thymidylate synthase

target	prob	fident	alnlen	evalue	theader
6n1s-assembly1.cif.gz_A	1	0.335	515	2.806e-51	Toxoplasma gondii TS-DHFR in complex with selective inhibitor 29
6kp2-assembly1.cif.gz_A	1	0.326	515	3.355e-51	Quadruple mutant plasmodium falciparum dihydrofolate reductase complexed with B10042
6kot-assembly1.cif.gz_A	1	0.329	519	4.521e-51	Quadruple mutant (N51I+C59R+S108N+I164L) plasmodium falciparum dihydrofolate reductase-thymidylate synthase (PfDHFR-TS) complexed with B12128 and NADPH

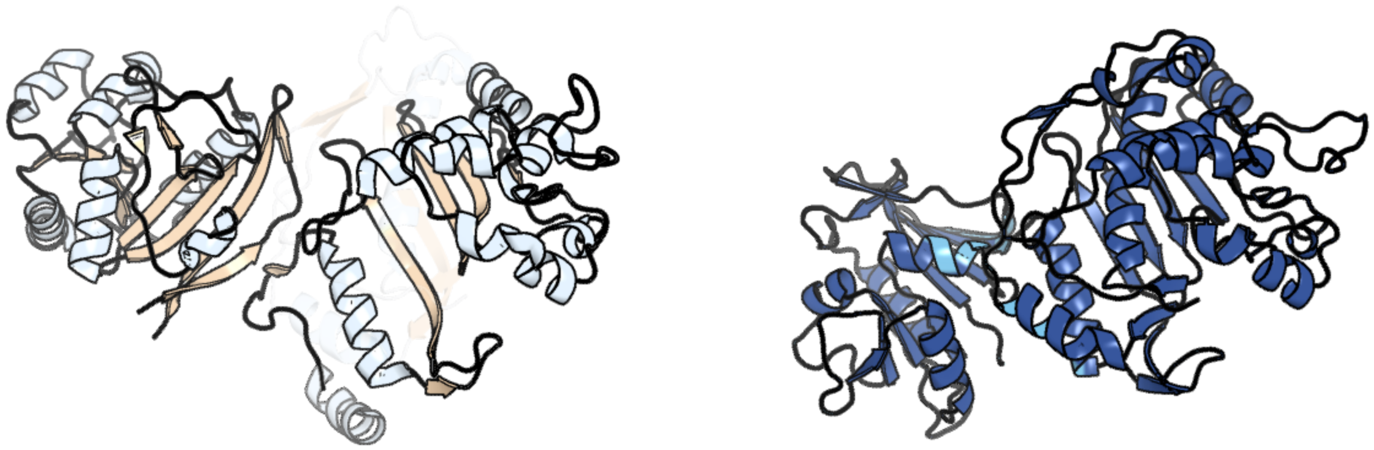

Figure 180: left: reference structure of 6n1s chain A. right: predicted structure of chlorv-1..180, unaligned sequences are shown as transparent

chlorv-1..181

- Sequence-based annotation for chlorv-1..181 is putative Cytidine deaminase
- No significant structural hit found

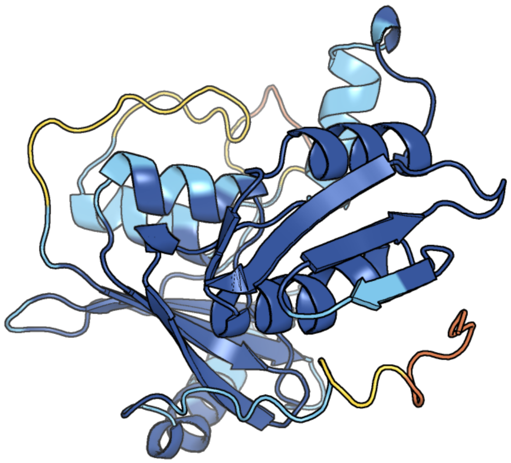

Figure 181: predicted structure of chlorv-1..181

chlorv-1..182

- Sequence-based annotation for chlorv-1..182 is putative Transcription initiation factor IIB
- Best hit was 7nvu chain M: Transcription initiation factor IIB

target	prob	fident	alnlen	evalue	theader
7nvu-assembly1.cif.gz_M	1	0.238	298	6.766e-12	RNA polymerase II core pre-initiation complex with open promoter DNA
8wak-assembly1.cif.gz_R	1	0.226	292	8.65e-11	Structure of transcribing complex 2 (TC2), the initially transcribing complex with Pol II positioned 2nt downstream of TSS.
8bvw-assembly1.cif.gz_M	1	0.208	288	1.54e-10	RNA polymerase II pre-initiation complex with the distal +1 nucleosome (PIC-Nuc18W)

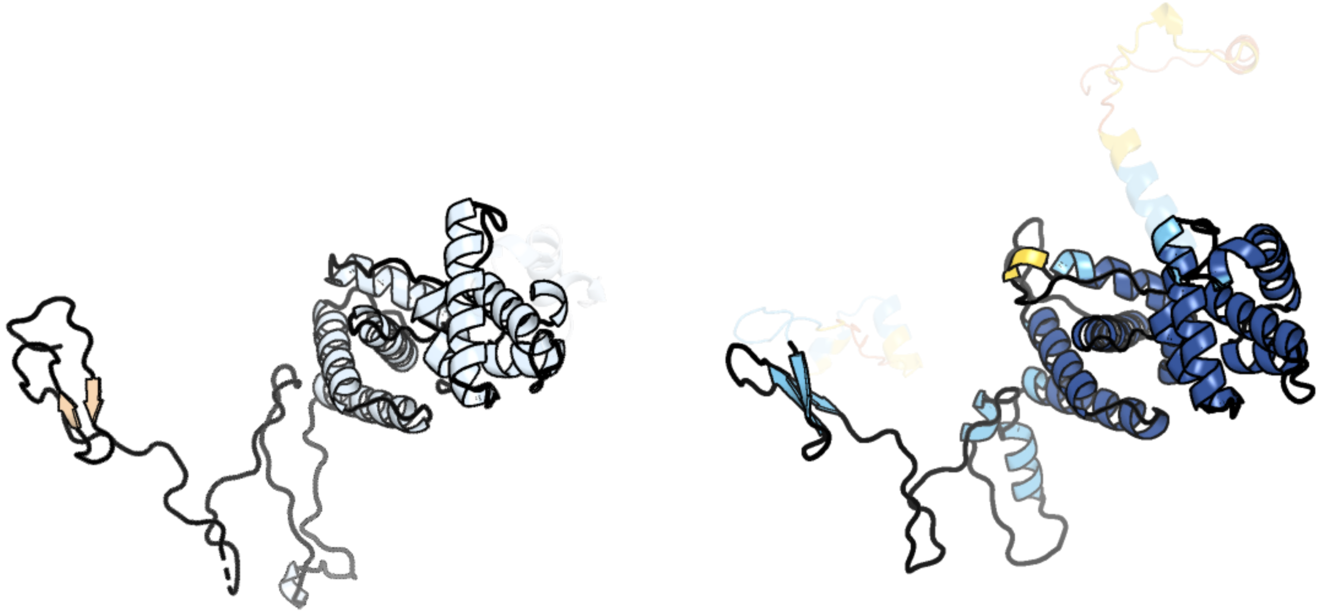

Figure 182: left: reference structure of 7nvu chain M. right: predicted structure of chlorv-1..182, unaligned sequences are shown as transparent

chlorv-1..183

- Sequence-based annotation for chlorv-1..183 is putative ATPase
- Best hit was 3syk chain A: Protein CbbX

target	prob	fident	alnlen	evalue	theader
3syk-assembly1.cif.gz_A	1	0.272	283	2.024e-17	Crystal structure of the AAA+ protein CbbX, selenomethionine structure
3syl-assembly1.cif.gz_B	1	0.267	284	1.165e-16	Crystal structure of the AAA+ protein CbbX, native structure
1iy0-assembly1.cif.gz_A	1	0.189	264	2.255e-10	Crystal structure of the FtsH ATPase domain with AMP-PNP from Thermus thermophilus

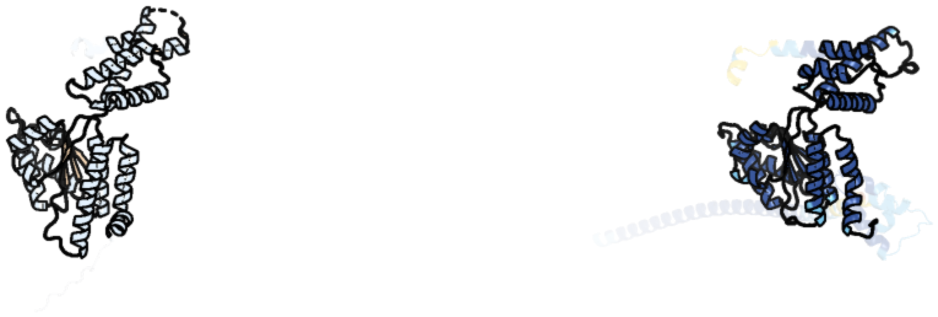

Figure 183: left: reference structure of 3syk chain A. right: predicted structure of chlorv-1..183, unaligned sequences are shown as transparent

chlorv-1..184

- Sequence-based annotation for chlorv-1..184 is putative Exonuclease
- Best hit was 6o3f chain B: Lysine–tRNA ligase

target	prob	fident	alnlen	evalue	theader
6o3f-assembly1.cif.gz_B	1	0.164	201	2.793e-06	Crystal Structure of Lysyl-tRNA Synthetase from Chlamydia trachomatis with complexed with L-lysine and a difluoro cyclohexyl chromone ligand
3a74-assembly1.cif.gz_A	1	0.134	171	5.04e-06	Lysyl-tRNA synthetase from Bacillus stearothermophilus complexed with Diadenosine Tetraphosphate (AP4A)
4ex5-assembly1.cif.gz_B	1	0.135	170	8.081e-06	Crystal structure of lysyl-tRNA synthetase LysRS from Burkholderia thailandensis bound to lysine

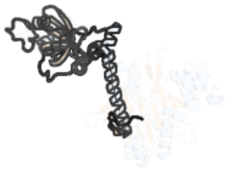

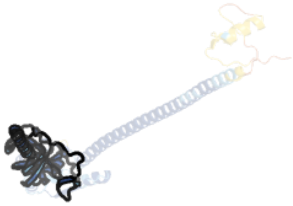

Figure 184: left: reference structure of 6o3f chain B. right: predicted structure of chlorv-1..184, unaligned sequences are shown as transparent

chlorv-1..185

- Sequence-based annotation for chlorv-1..185 is hypothetical protein
- No significant structural hit found

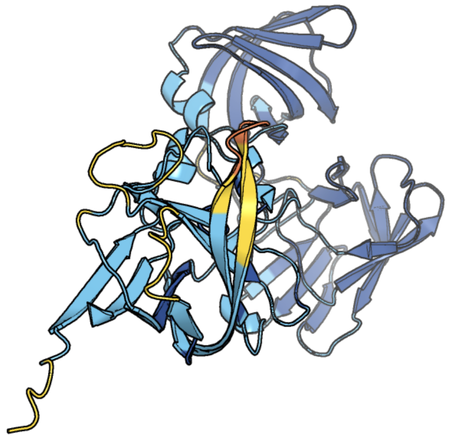

Figure 185: predicted structure of chlorv-1..185

chlorv-1..186

- Sequence-based annotation for chlorv-1..186 is hypothetical protein
- No significant structural hit found

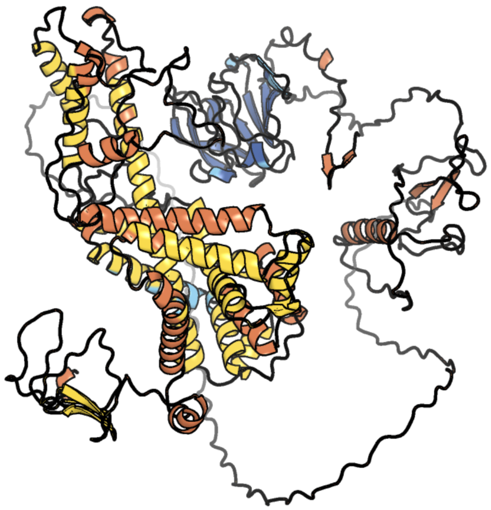

Figure 186: predicted structure of chlorv-1..186

chlorv-1..187

- Sequence-based annotation for chlorv-1..187 is hypothetical protein
- No significant structural hit found

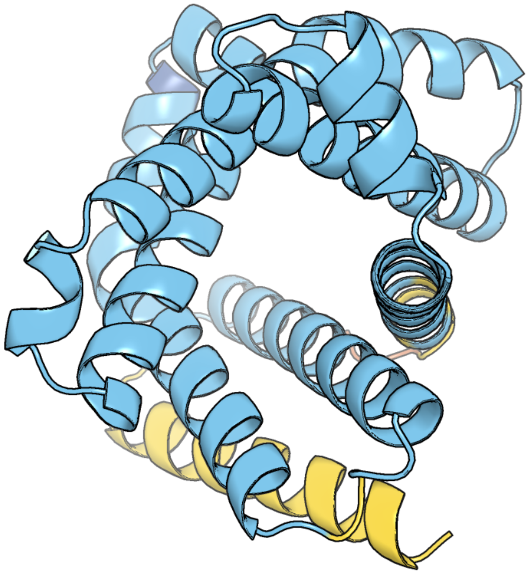

Figure 187: predicted structure of chlorv-1..187

chlorv-1..188

- Sequence-based annotation for chlorv-1..188 is hypothetical protein
- No significant structural hit found

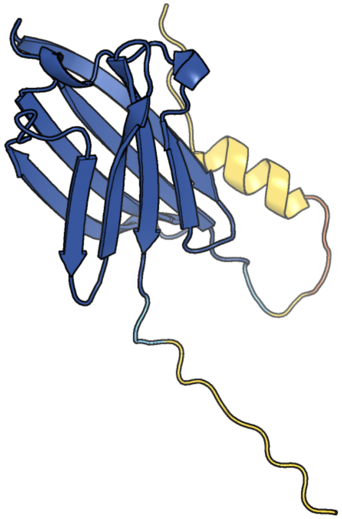

Figure 188: predicted structure of chlorv-1..188

chlorv-1..189

- Sequence-based annotation for chlorv-1..189 is hypothetical protein
- No significant structural hit found

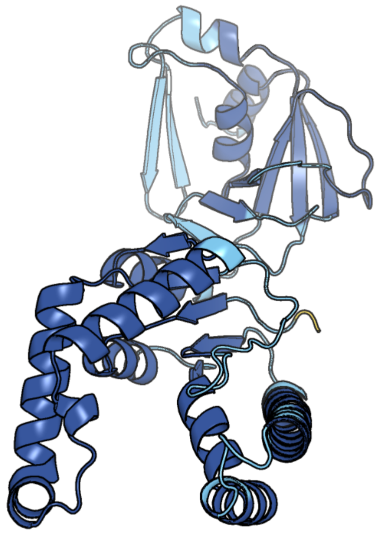

Figure 189: predicted structure of chlorv-1..189

chlorv-1..190

- Sequence-based annotation for chlorv-1..190 is putative Transcription factor S-II
- Best hit was 8a40 chain U: Transcription elongation factor A protein 1

target	prob	fident	alnlen	evalue	theader
8a40-assembly1.cif.gz_U	1	0.247	178	5.394e-07	Structure of mammalian Pol II-TFIIS elongation complex
7und-assembly1.cif.gz_O	1	0.224	178	1.022e-06	Pol II-DSIF-SPT6-PAF1c-TFIIS-nucleosome complex (stalled at +38)
6o9l-assembly1.cif.gz_U	1	0.241	178	1.449e-06	Human holo-PIC in the closed state

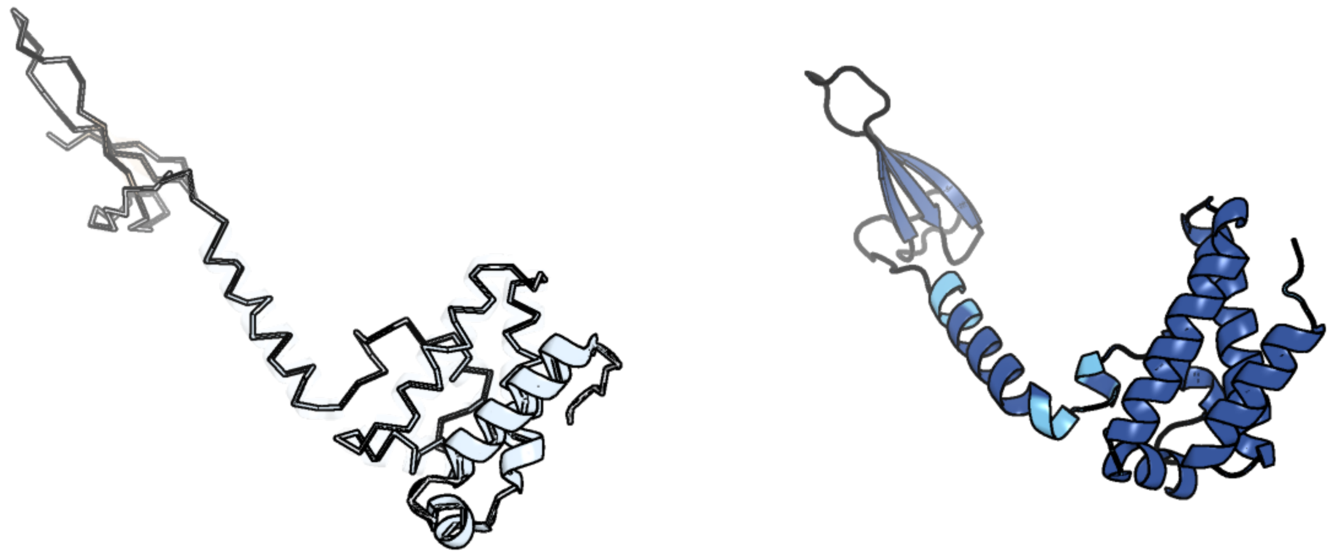

Figure 190: left: reference structure of 8a40 chain U. right: predicted structure of chlorv-1..190, unaligned sequences are shown as transparent

chlorv-1..191

- Sequence-based annotation for chlorv-1..191 is putative RING-finger-containing E3 ubiquitin ligase
- No significant structural hit found

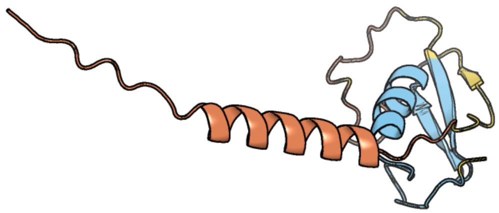

Figure 191: predicted structure of chlorv-1..191

chlorv-1..192

- Sequence-based annotation for chlorv-1..192 is putative Threonyl-tRNA synthetase
- Best hit was 4hwt chain A: Threonine–tRNA ligase, cytoplasmic

target	prob	fident	alnlen	evalue	theader
4hwt-assembly1.cif.gz_A	1	0.53	390	5.12e-61	Crystal structure of human Threonyl-tRNA synthetase bound to a novel inhibitor
4p3n-assembly1.cif.gz_B	1	0.533	390	1.504e-59	Structural Basis for Full-Spectrum Inhibition of Threonyl-tRNA Synthetase by Borrelidin 1
7l3o-assembly2.cif.gz_B	1	0.526	393	1.898e-58	Crystal Structure of the RNA binding domain of Threonyl-tRNA synthetase from Cryptosporidium parvum Iowa II

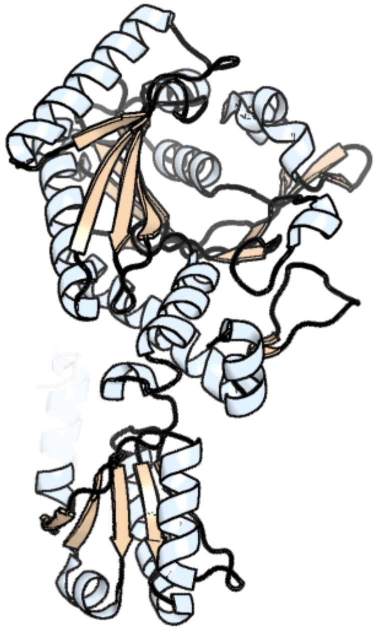

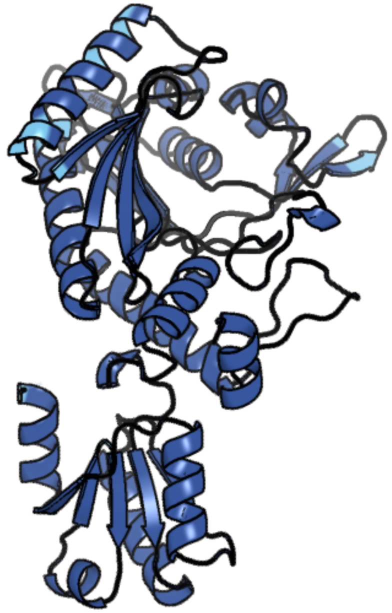

Figure 192: left: reference structure of 4hwt chain A. right: predicted structure of chlorv-1..192, unaligned sequences are shown as transparent

chlorv-1..193

- Sequence-based annotation for chlorv-1..193 is hypothetical protein
- No significant structural hit found

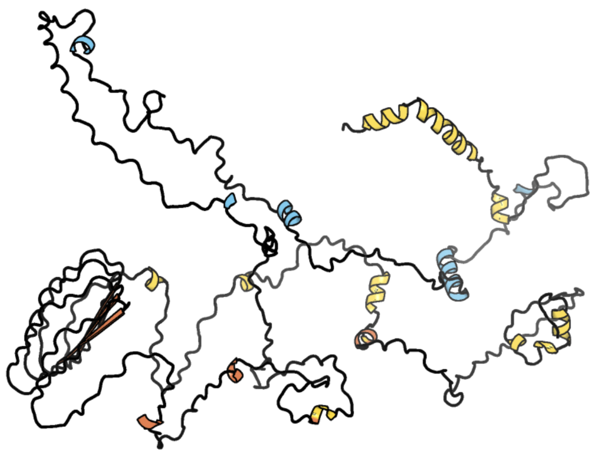

Figure 193: predicted structure of chlorv-1..193

chlorv-1..194

- Sequence-based annotation for chlorv-1..194 is hypothetical protein
- No significant structural hit found

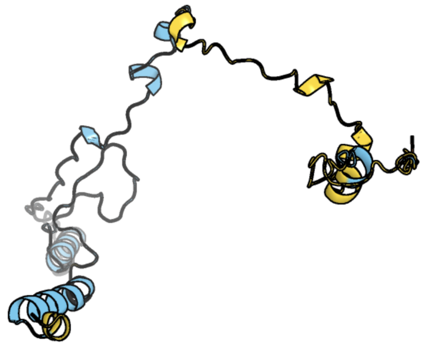

Figure 194: predicted structure of chlorv-1..194

chlorv-1..195

- Sequence-based annotation for chlorv-1..195 is hypothetical protein
- No significant structural hit found

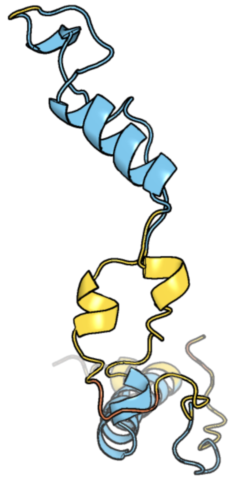

Figure 195: predicted structure of chlorv-1..195

chlorv-1..196

- Sequence-based annotation for chlorv-1..196 is hypothetical protein
- No significant structural hit found

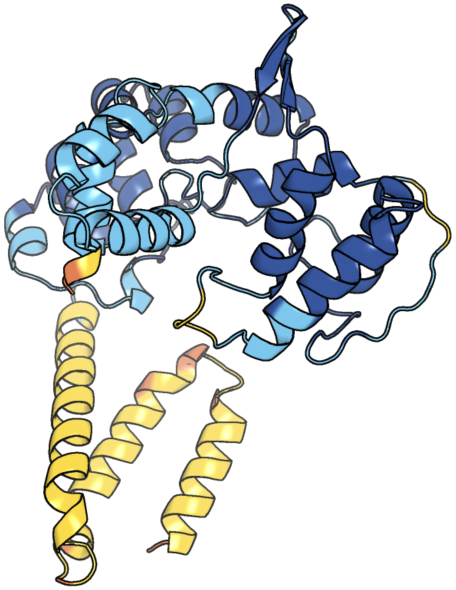

Figure 196: predicted structure of chlorv-1..196

chlorv-1..197

- Sequence-based annotation for chlorv-1..197 is hypothetical protein
- No significant structural hit found

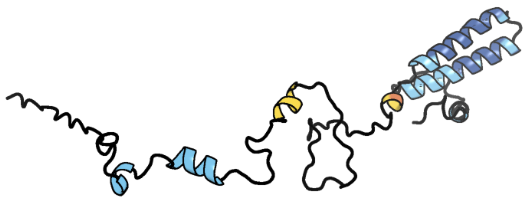

Figure 197: predicted structure of chlorv-1..197

chlorv-1..198

- Sequence-based annotation for chlorv-1..198 is putative Replication factor C subunit 2
- Best hit was 7z6h chain D: Replication factor C subunit 4

target	prob	fident	alnlen	evalue	theader
7z6h-assembly1.cif.gz_D	1	0.386	321	4.777e-26	Structure of DNA-bound human RAD17-RFC clamp loader and 9-1-1 checkpoint clamp
8dqx-assembly1.cif.gz_C	1	0.36	325	2.663e-24	Open state of RFC:PCNA bound to a 3' ss/dsDNA junction
1sxj-assembly1.cif.gz_C	1	0.354	327	3.786e-24	Crystal Structure of the Eukaryotic Clamp Loader (Replication Factor C, RFC) Bound to the DNA Sliding Clamp (Proliferating Cell Nuclear Antigen, PCNA)

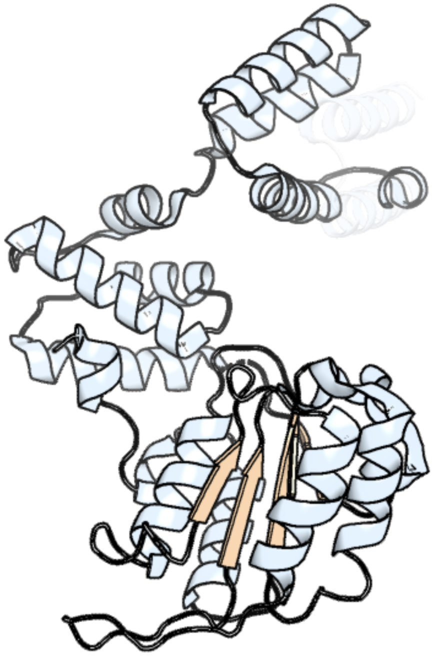

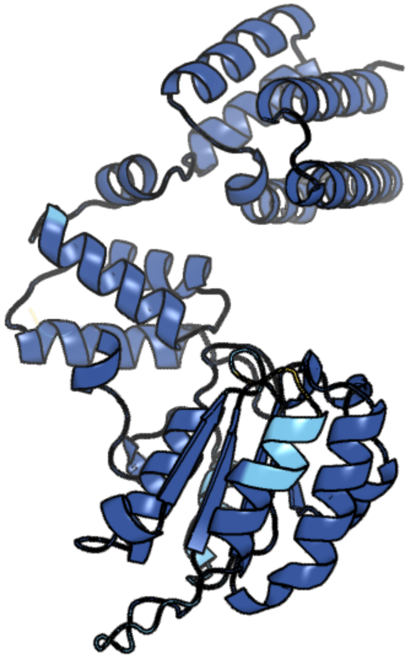

Figure 198: left: reference structure of 7z6h chain D. right: predicted structure of chlorv-1..198, unaligned sequences are shown as transparent

chlorv-1..199

- Sequence-based annotation for chlorv-1..199 is hypothetical protein
- No significant structural hit found

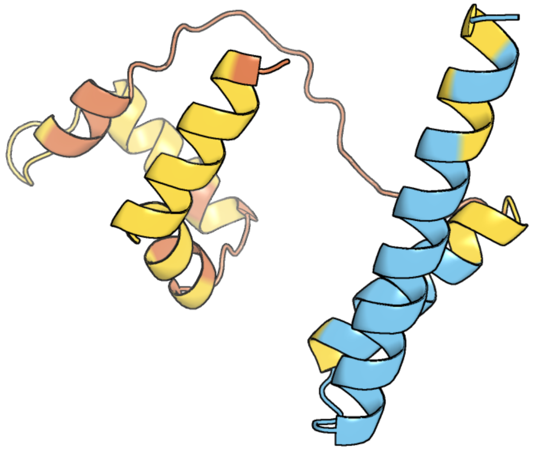

Figure 199: predicted structure of chlorv-1..199

chlorv-1..200

- Sequence-based annotation for chlorv-1..200 is putative DNA topoisomerase I
- Best hit was 1k4t chain A: DNA topoisomerase I

target	prob	fident	alnlen	evalue	theader
1k4t-assembly1.cif.gz_A	1	0.365	563	3.318e-50	HUMAN DNA TOPOISOMERASE I (70 KDA) IN COMPLEX WITH THE POISON TOPOTECAN AND COVALENT COMPLEX WITH A 22 BASE PAIR DNA DUPLEX
1seu-assembly1.cif.gz_A	1	0.367	563	2.532e-48	Human DNA Topoisomerase I (70 Kda) In Complex With The Indolocarbazole SA315F and Covalent Complex With A 22 Base Pair DNA Duplex
1lpq-assembly1.cif.gz_A	1	0.371	560	2.064e-45	Human DNA Topoisomerase I (70 Kda) In Non-Covalent Complex With A 22 Base Pair DNA Duplex Containing an 8-oxoG Lesion

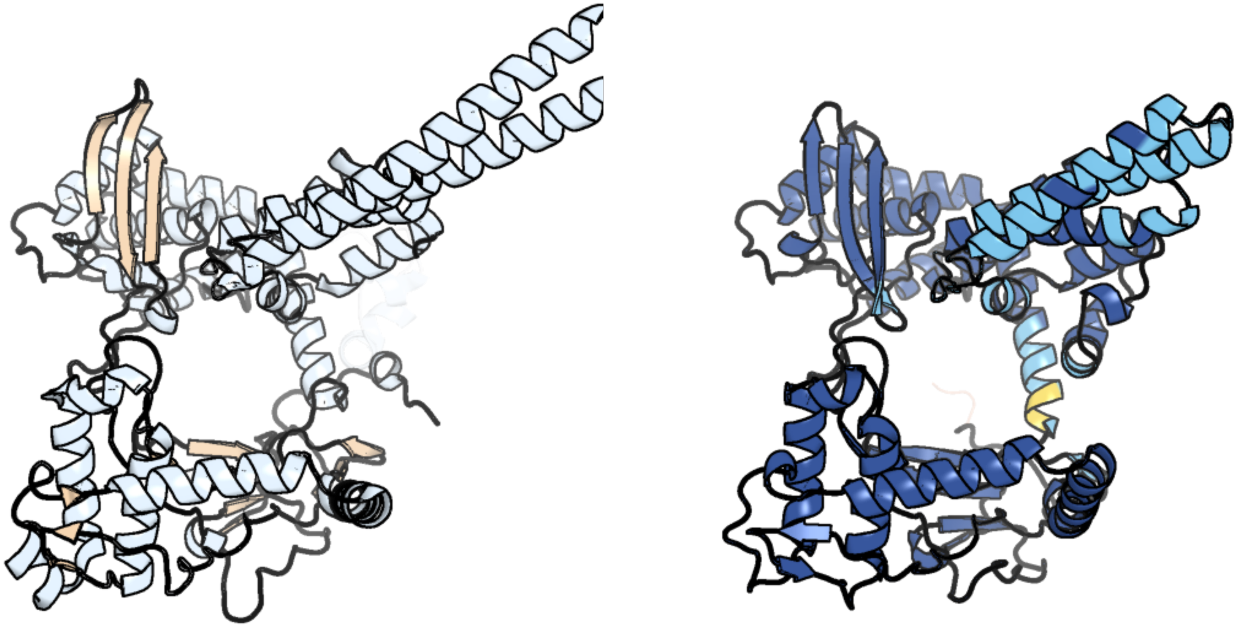

Figure 200: left: reference structure of 1k4t chain A. right: predicted structure of chlorv-1..200, unaligned sequences are shown as transparent

chlorv-1..201

- Sequence-based annotation for chlorv-1..201 is hypothetical protein
- No significant structural hit found

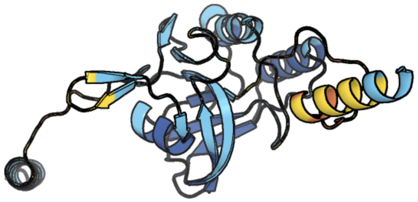

Figure 201: predicted structure of chlorv-1..201

chlorv-1..202

- Sequence-based annotation for chlorv-1..202 is putative RNA polymerase subunit alpha
- Best hit was 7okx chain A: DNA-directed RNA polymerase II subunit RPB1

target	prob	fident	alnlen	evalue	theader
8jh4-assembly1.cif.gz_A	1	0.289	1499	0	RNA polymerase II elongation complex containing 60 bp upstream DNA loop, stalled at SHL(-1) of the nucleosome
8h0v-assembly1.cif.gz_A	1	0.289	1511	0	RNA polymerase II transcribing a chromatosome (type I)
7okx-assembly1.cif.gz_A	1	0.295	1516	0	Structure of active transcription elongation complex Pol II-DSIF (SPT5-KOW5)-ELL2-EAF1 (composite structure)

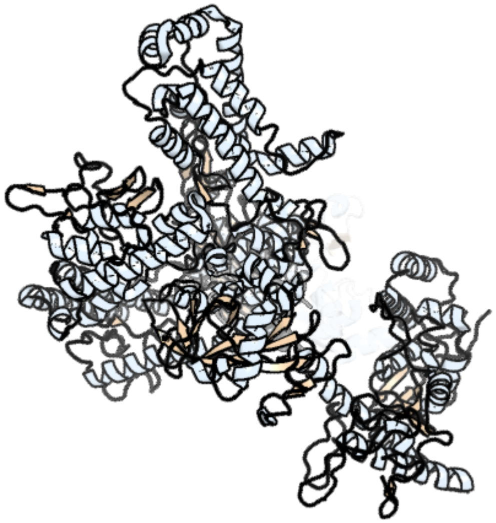

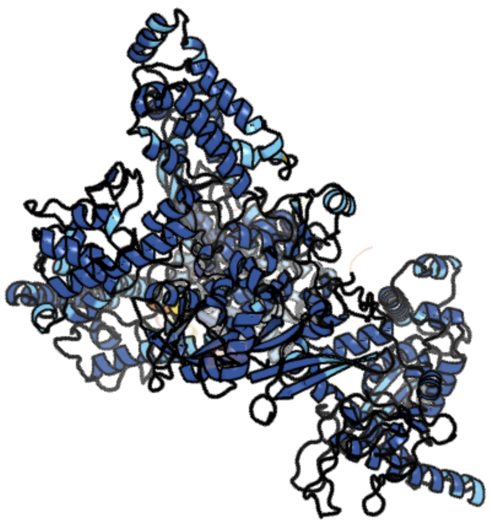

Figure 202: left: reference structure of 7okx chain A. right: predicted structure of chlorv-1..202, unaligned sequences are shown as transparent

chlorv-1..203

- Sequence-based annotation for chlorv-1..203 is hypothetical protein
- No significant structural hit found

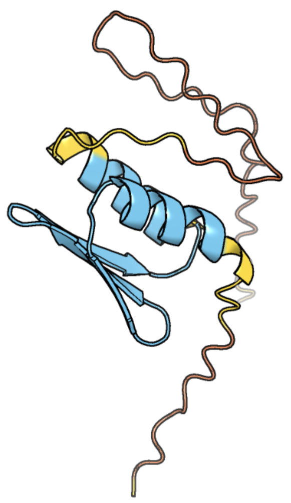

Figure 203: predicted structure of chlorv-1..203

chlorv-1..204

- Sequence-based annotation for chlorv-1..204 is putative Peptidase
- Best hit was 4pf9 chain A: Insulin-degrading enzyme

target	prob	fident	alnlen	evalue	theader
4pf9-assembly1.cif.gz_A	1	0.202	935	6.53e-52	Crystal structure of insulin degrading enzyme complexed with inhibitor
7k1f-assembly1.cif.gz_B	1	0.203	929	1.28e-51	Crystal structure of human insulin degrading enzyme (IDE) in complex with compound BDM_88558
4nxo-assembly1.cif.gz_A	1	0.2	932	1.88e-51	Crystal Structure of Insulin Degrading Enzyme in complex with BDM44768

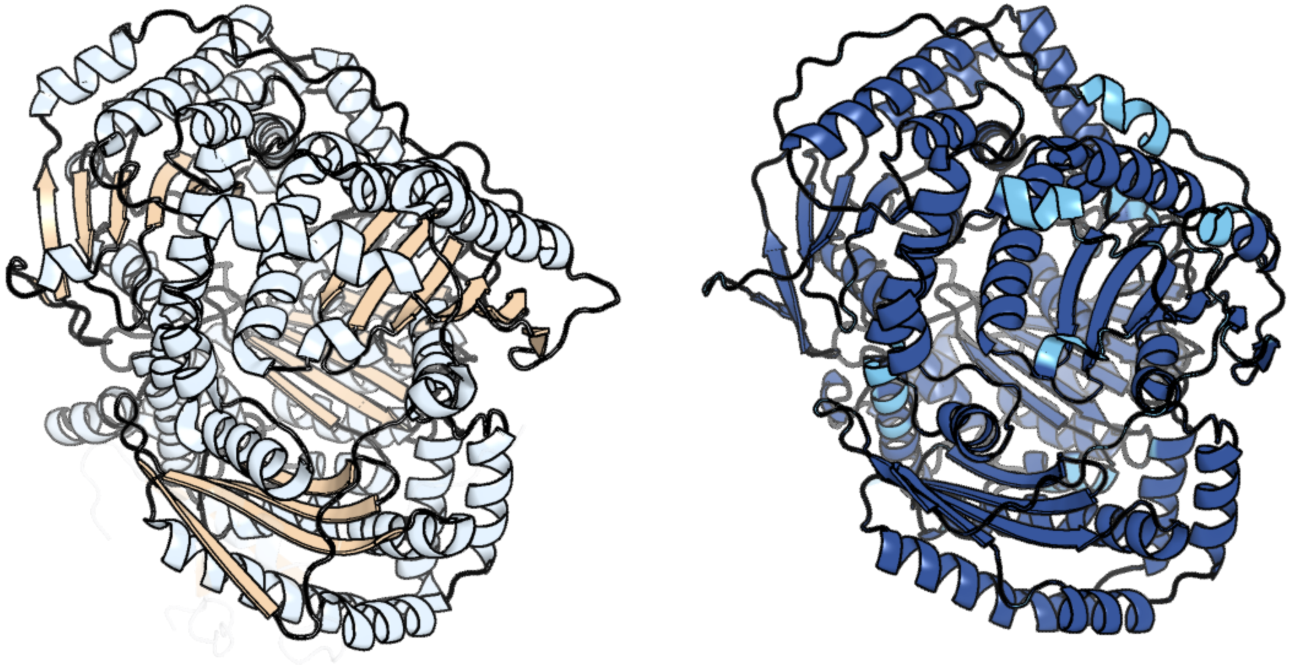

Figure 204: left: reference structure of 4pf9 chain A. right: predicted structure of chlorv-1..204, unaligned sequences are shown as transparent

chlorv-1..205

- Sequence-based annotation for chlorv-1..205 is hypothetical protein
- No significant structural hit found

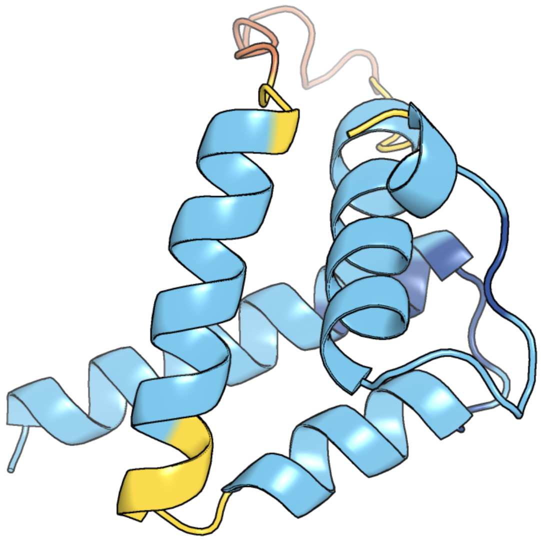

Figure 205: predicted structure of chlorv-1..205

chlorv-1..206

- Sequence-based annotation for chlorv-1..206 is putative Hsp70 protein
- Best hit was 7n1r chain A: Endoplasmic reticulum chaperone BiP

target	prob	fident	alnlen	evalue	theader
7n1r-assembly1.cif.gz_A	1	0.162	597	4.163e-25	A novel and unique ATP hydrolysis to AMP by a human Hsp70 BiP
4b9q-assembly2.cif.gz_B	1	0.17	651	2.349e-24	Open conformation of ATP-bound Hsp70 homolog DnaK
4b9q-assembly4.cif.gz_D	1	0.176	639	3.573e-24	Open conformation of ATP-bound Hsp70 homolog DnaK

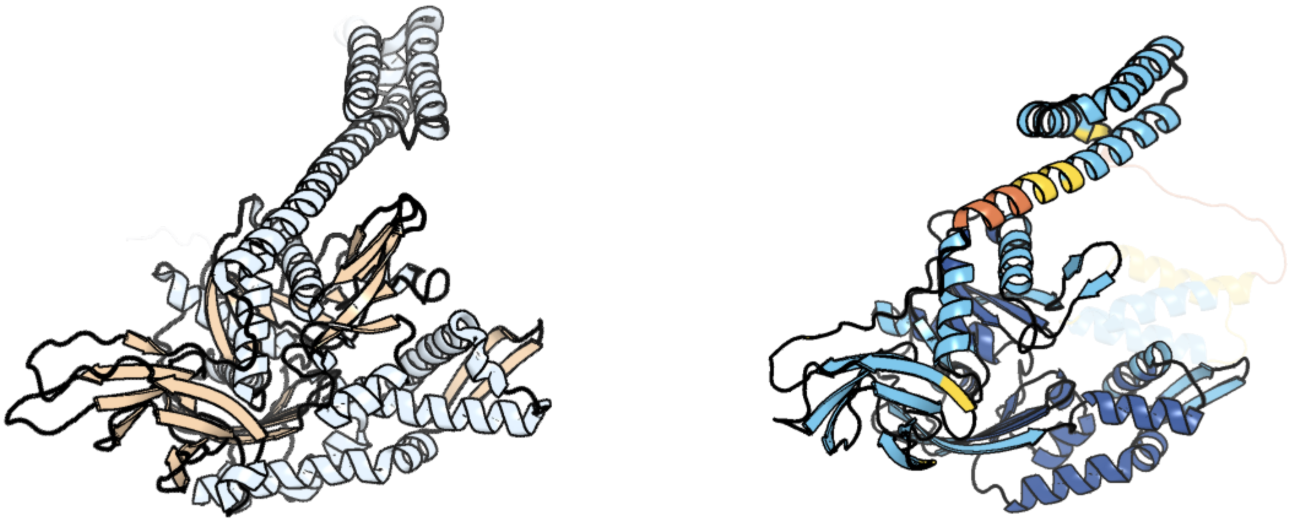

Figure 206: left: reference structure of 7n1r chain A. right: predicted structure of chlorv-1..206, unaligned sequences are shown as transparent

chlorv-1..207

- Sequence-based annotation for chlorv-1..207 is hypothetical protein
- No significant structural hit found

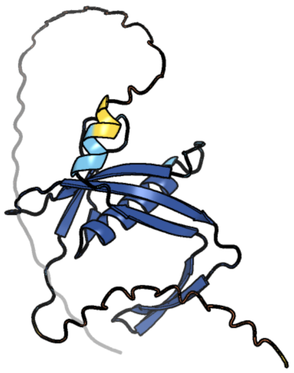

Figure 207: predicted structure of chlorv-1..207

chlorv-1..208

- Sequence-based annotation for chlorv-1..208 is putative Peptidase
- Best hit was 6on2 chain C: ATP-dependent protease La

target	prob	fident	alnlen	evalue	theader
6on2-assembly1.cif.gz_C	1	0.279	551	4.504e-39	Lon Protease from Yersinia pestis with Y2853 substrate
7p0m-assembly1.cif.gz_B	1	0.25	562	1.368e-38	Human mitochondrial Lon protease with substrate in the ATPase and protease domains
7p0m-assembly1.cif.gz_A	1	0.255	555	1.433e-38	Human mitochondrial Lon protease with substrate in the ATPase and protease domains

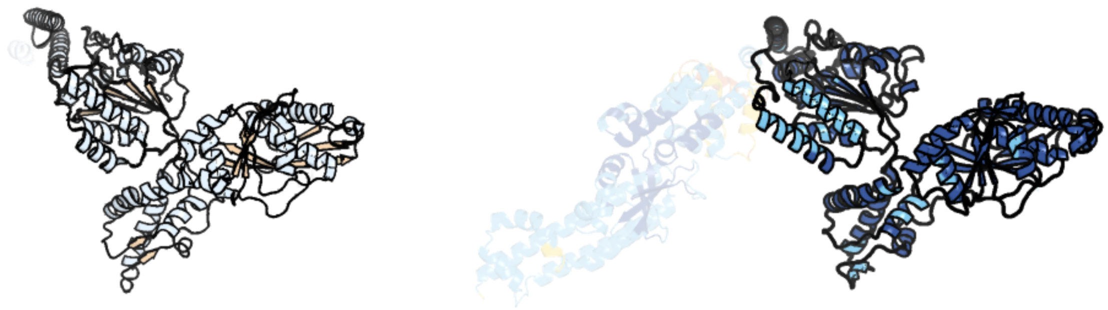

Figure 208: left: reference structure of 6on2 chain C. right: predicted structure of chlorv-1..208, unaligned sequences are shown as transparent

chlorv-1..209

- Sequence-based annotation for chlorv-1..209 is hypothetical protein
- No significant structural hit found

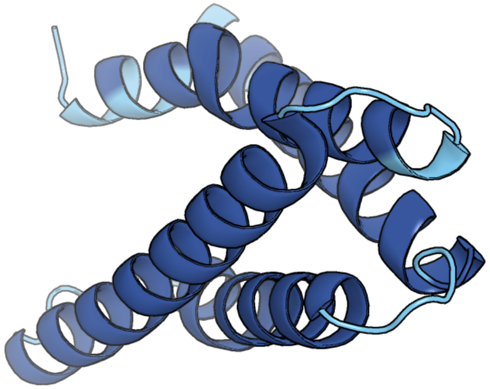

Figure 209: predicted structure of chlorv-1..209

chlorv-1..210

- Sequence-based annotation for chlorv-1..210 is hypothetical protein
- No significant structural hit found

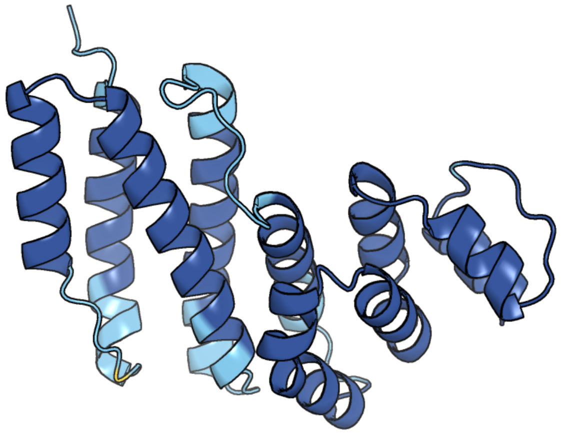

Figure 210: predicted structure of chlorv-1..210

chlorv-1..211

- Sequence-based annotation for chlorv-1..211 is hypothetical protein
- No significant structural hit found

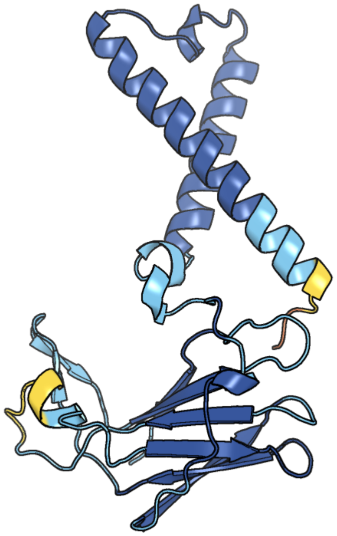

Figure 211: predicted structure of chlorv-1..211

chlorv-1..212

- Sequence-based annotation for chlorv-1..212 is putative Glycosyltransferase
- Best hit was 7mi0 chain A: Glycosyltransferase

target	prob	fident	alnlen	evalue	theader
7mi0-assembly1.cif.gz_A	1	0.132	453	3.668e-11	Crystal Structure of Glycosyltransferase from Rickettsia africae ESF-5
3l01-assembly2.cif.gz_B	1	0.094	496	6.756e-11	Crystal structure of monomeric glycogen synthase from Pyrococcus abyssi
3c4q-assembly1.cif.gz_A	1	0.125	448	7.549e-11	Structure of the retaining glycosyltransferase MshA : The first step in mycothiol biosynthesis. Organism : Corynebacterium glutamicum- Complex with UDP

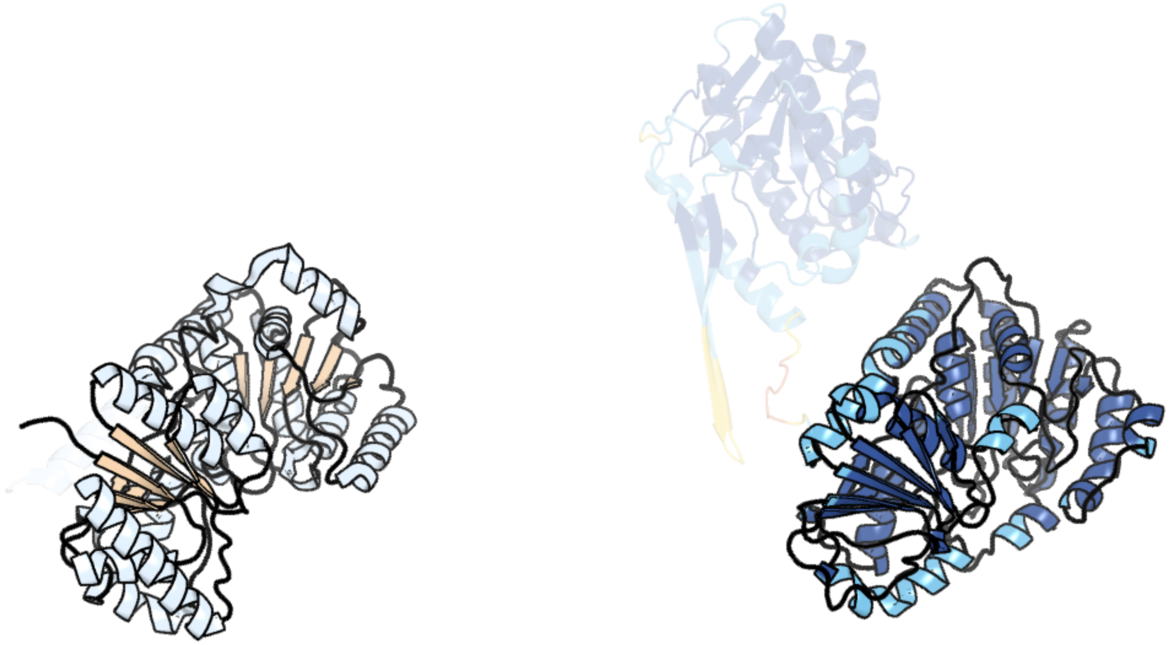

Figure 212: left: reference structure of 7mi0 chain A. right: predicted structure of chlorv-1..212, unaligned sequences are shown as transparent

chlorv-1..213

- Sequence-based annotation for chlorv-1..213 is hypothetical protein
- No significant structural hit found

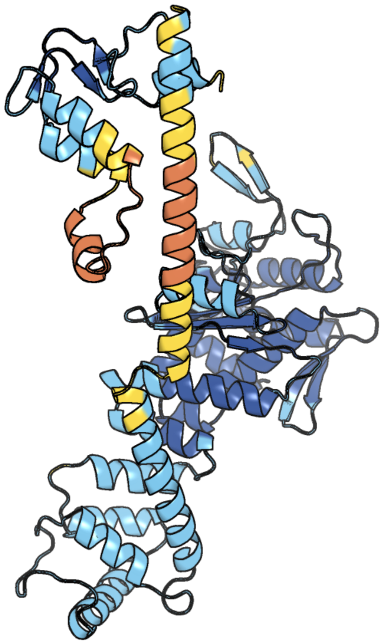

Figure 213: predicted structure of chlorv-1..213

chlorv-1..214

- Sequence-based annotation for chlorv-1..214 is hypothetical protein
- No significant structural hit found

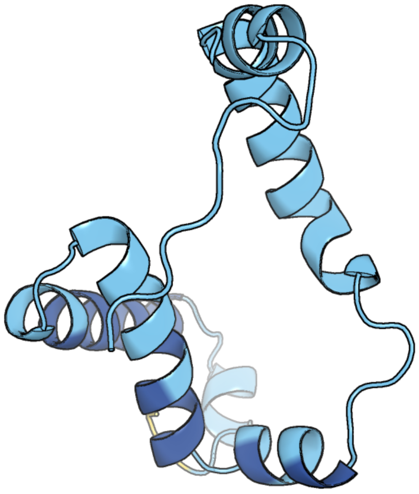

Figure 214: predicted structure of chlorv-1..214

chlorv-1..215

- Sequence-based annotation for chlorv-1..215 is putative Thioredoxin
- Best hit was 3vww chain A: Protein disulfide-isomerase A6

target	prob	fident	alnlen	evalue	theader
3vww-assembly1.cif.gz_A	1	0.3	90	2.137e-07	Crystal structure of a0-domain of P5 from H. sapiens
1x5d-assembly1.cif.gz_A	1	0.285	91	2.435e-07	The solution structure of the second thioredoxin-like domain of human Protein disulfide-isomerase A6
3wge-assembly1.cif.gz_A	1	0.275	87	6.062e-07	Crystal structure of ERp46 Trx2

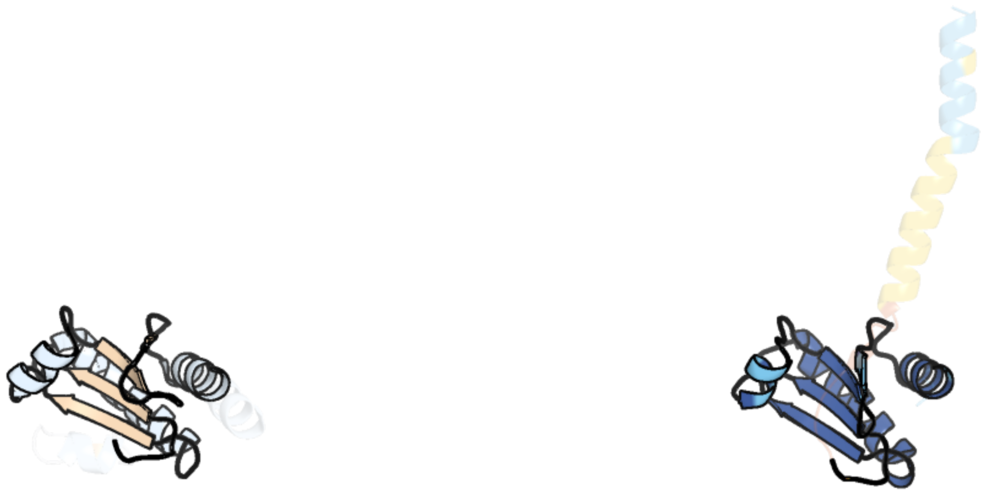

Figure 215: left: reference structure of 3vww chain A. right: predicted structure of chlorv-1..215, unaligned sequences are shown as transparent

chlorv-1..216

- Sequence-based annotation for chlorv-1..216 is hypothetical protein
- No significant structural hit found

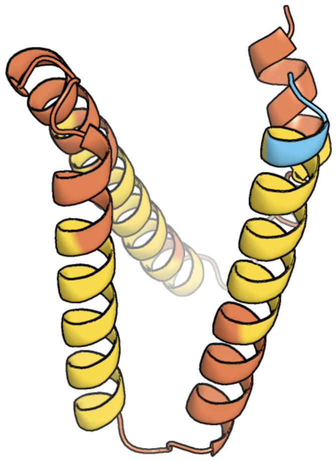

Figure 216: predicted structure of chlorv-1..216

chlorv-1..217

- Sequence-based annotation for chlorv-1..217 is putative Peptidase
- Best hit was 5cvm chain A: Ubiquitin carboxyl-terminal hydrolase 46

target	prob	fident	alnlen	evalue	theader
5cvm-assembly1.cif.gz_A	1	0.239	309	1.671e-24	USP46~ubiquitin BEA covalent complex
5cvo-assembly2.cif.gz_E	1	0.242	321	1.878e-24	WDR48:USP46~ubiquitin ternary complex
6dgf-assembly1.cif.gz_A	1	0.229	327	5.384e-24	Ubiquitin Variant bound to USP2

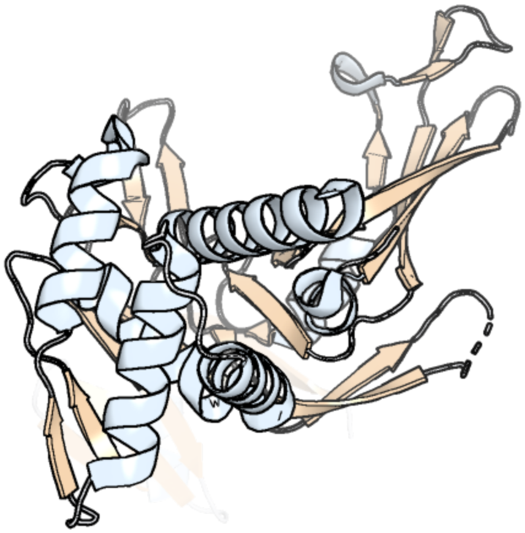

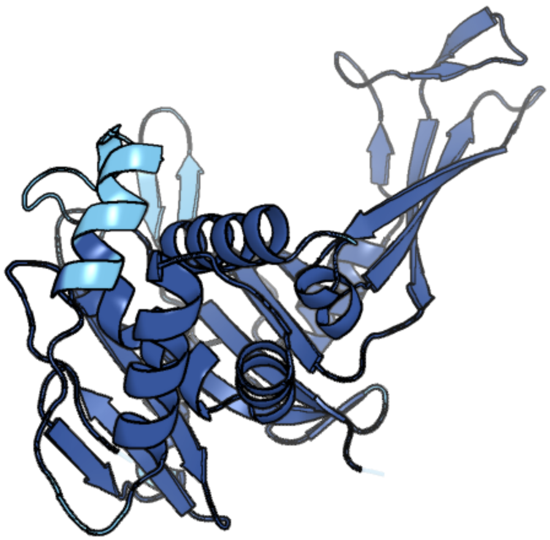

Figure 217: left: reference structure of 5cvm chain A. right: predicted structure of chlorv-1..217, unaligned sequences are shown as transparent

chlorv-1..218

- Sequence-based annotation for chlorv-1..218 is hypothetical protein
- No significant structural hit found

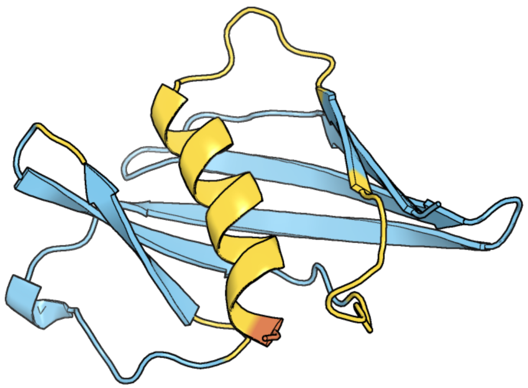

Figure 218: predicted structure of chlorv-1..218

chlorv-1..219

- Sequence-based annotation for chlorv-1..219 is putative Nuclease
- Best hit was 7dcy chain A: Ribonuclease R

target	prob	fident	alnlen	evalue	theader
7dcy-assembly1.cif.gz_A	1	0.18	660	3.424e-25	Apo form of Mycoplasma genitalium RNase R
7did-assembly1.cif.gz_A	1	0.175	665	1.639e-24	Mycoplasma genitalium RNase R in complex with ribose methylated single-stranded RNA
7tuv-assembly1.cif.gz_A	1	0.149	582	5.873e-23	Crystal structure of the exoribonucleolytic module of T. brucei RRP44

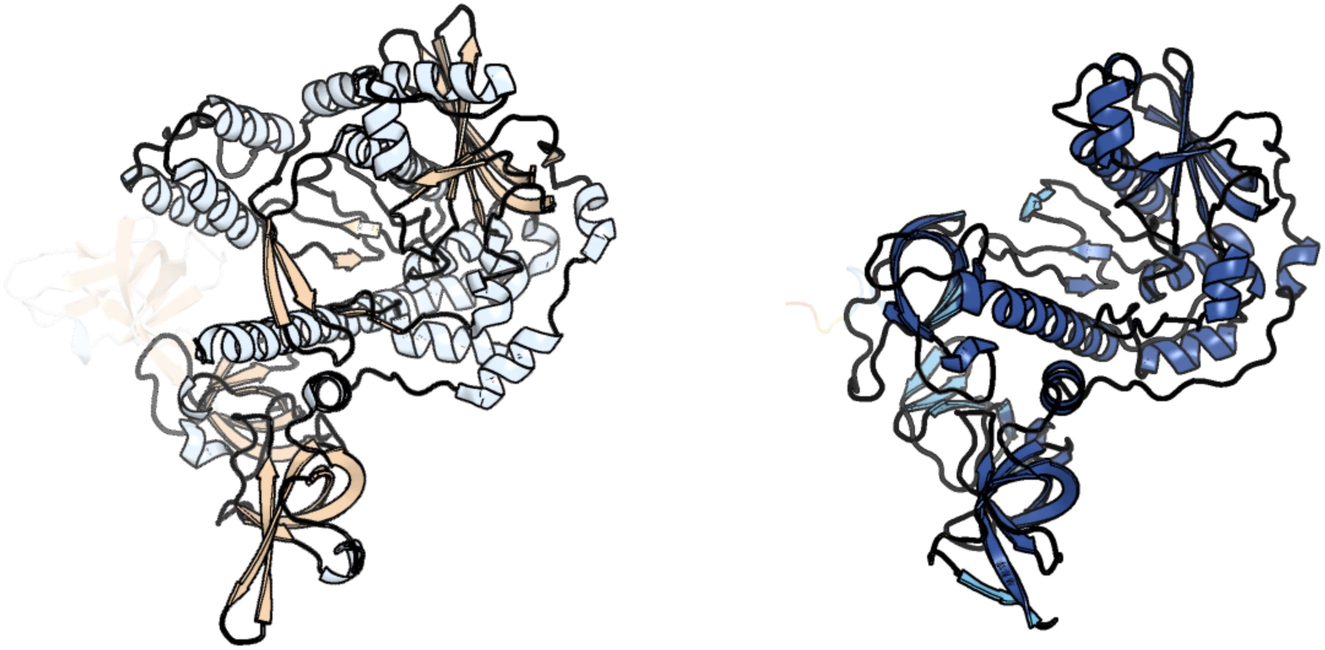

Figure 219: left: reference structure of 7dcy chain A. right: predicted structure of chlorv-1..219, unaligned sequences are shown as transparent

chlorv-1..220

- Sequence-based annotation for chlorv-1..220 is hypothetical protein
- No significant structural hit found

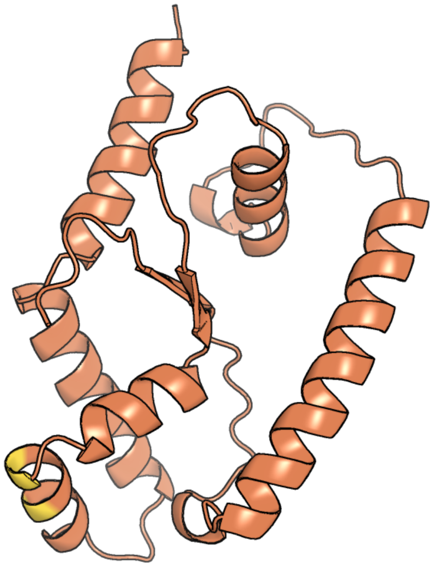

Figure 220: predicted structure of chlorv-1..220

chlorv-1..221

- Sequence-based annotation for chlorv-1..221 is hypothetical protein
- No significant structural hit found

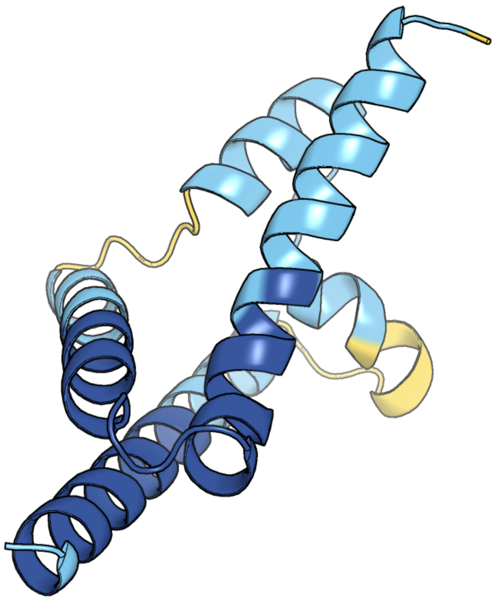

Figure 221: predicted structure of chlorv-1..221

chlorv-1..222

- Sequence-based annotation for chlorv-1..222 is hypothetical protein
- No significant structural hit found

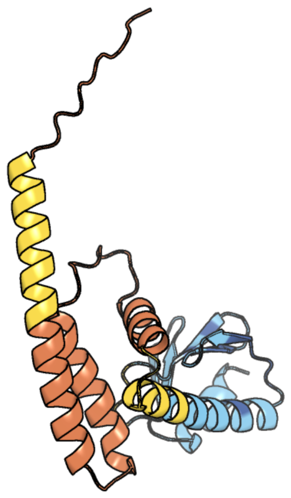

Figure 222: predicted structure of chlorv-1..222

chlorv-1..223

- Sequence-based annotation for chlorv-1..223 is hypothetical protein
- No significant structural hit found

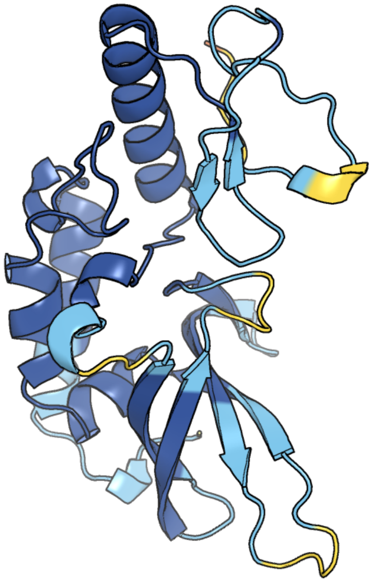

Figure 223: predicted structure of chlorv-1..223

chlorv-1..224

- Sequence-based annotation for chlorv-1..224 is putative Photolyase
- Best hit was 4dja chain A: Photolyase

target	prob	fident	alnlen	evalue	theader
4dja-assembly1.cif.gz_A	1	0.257	474	3.081e-27	Crystal structure of a prokaryotic (6-4) photolyase PhrB from Agrobacterium Tumefaciens with an Fe-S cluster and a 6,7-dimethyl-8-ribityllumazine antenna chromophore at 1.45A resolution
5kcm-assembly2.cif.gz_B	1	0.256	479	5.129e-27	Crystal structure of iron-sulfur cluster containing photolyase PhrB mutant I51W
5lfa-assembly1.cif.gz_A	1	0.253	469	6.618e-27	Crystal structure of iron-sulfur cluster containing bacterial (6-4) photolyase PhrB - Y424F mutant with impaired DNA repair activity

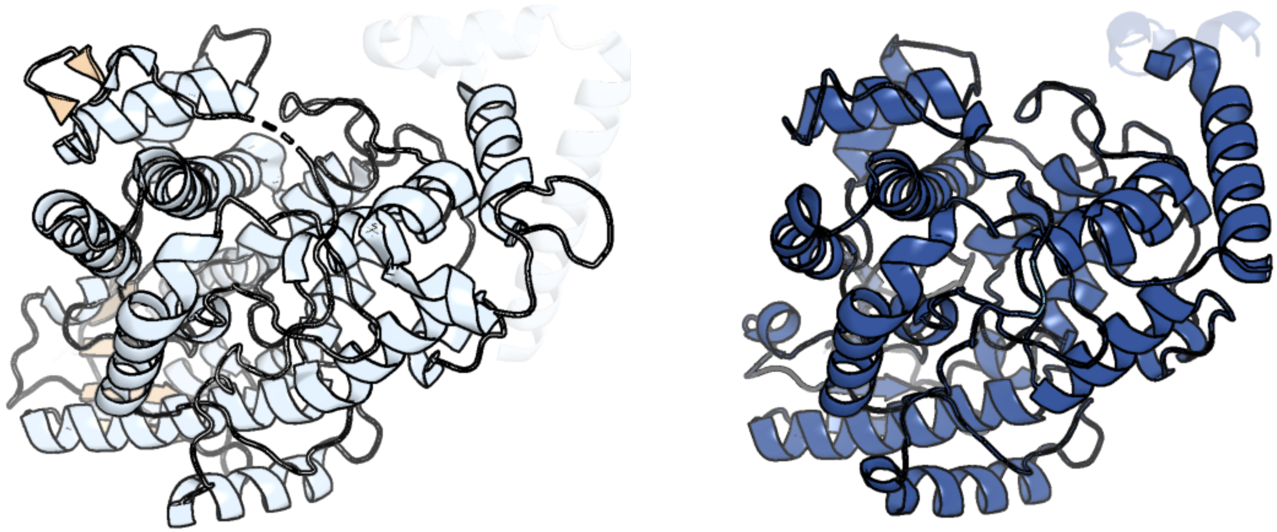

Figure 224: left: reference structure of 4dja chain A. right: predicted structure of chlorv-1..224, unaligned sequences are shown as transparent

chlorv-1..225

- Sequence-based annotation for chlorv-1..225 is putative ABC transporter
- Best hit was 5zxd chain B: ATP-binding cassette sub-family F member 1

target	prob	fident	alnlen	evalue	theader
5zxd-assembly2.cif.gz_B	1	0.309	539	8.39e-41	Crystal structure of ATP-bound human ABCF1
5zxd-assembly1.cif.gz_A	1	0.284	545	6.451e-38	Crystal structure of ATP-bound human ABCF1
4fin-assembly1.cif.gz_A	1	0.24	562	8.462e-36	Crystal Structure of EttA (formerly YjjK) - an E. coli ABC-type ATPase

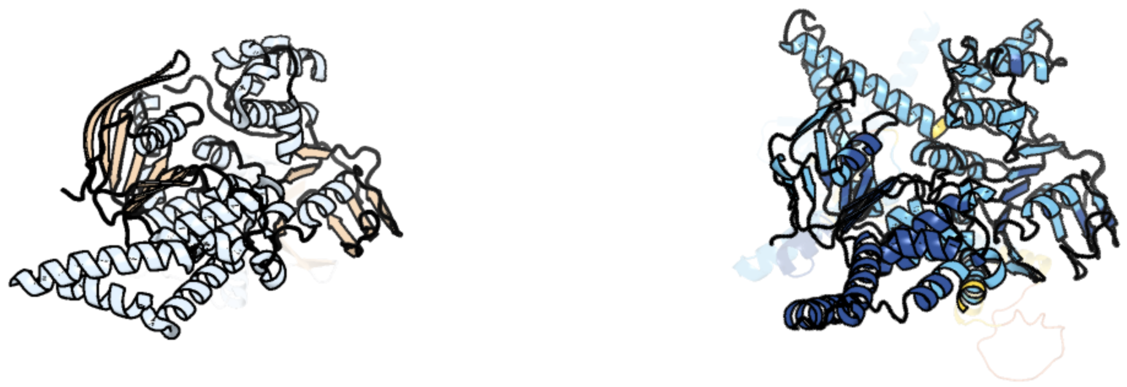

Figure 225: left: reference structure of 5zxd chain B. right: predicted structure of chlorv-1..225, unaligned sequences are shown as transparent

chlorv-1..226

- Sequence-based annotation for chlorv-1..226 is putative Helicase
- Best hit was 6ro1 chain A: Exosome RNA helicase MTR4

target	prob	fident	alnlen	evalue	theader
6ro1-assembly1.cif.gz_A	1	0.252	923	2.082e-49	X-ray crystal structure of the MTR4 NVL complex
2xgj-assembly2.cif.gz_B	1	0.279	806	2.512e-49	Structure of Mtr4, a DExH helicase involved in nuclear RNA processing and surveillance
4u4c-assembly1.cif.gz_A	1	0.251	939	3.485e-47	The molecular architecture of the TRAMP complex reveals the organization and interplay of its two catalytic activities

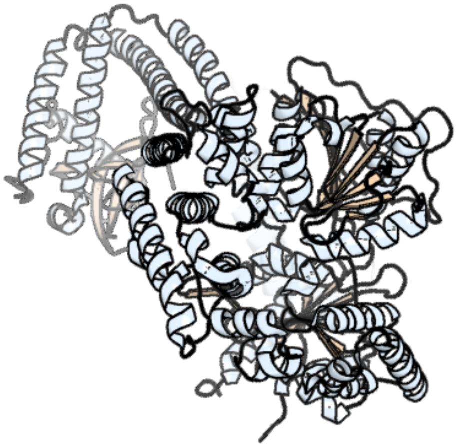

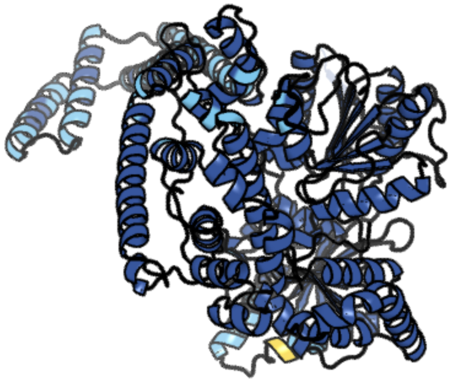

Figure 226: left: reference structure of 6ro1 chain A. right: predicted structure of chlorv-1..226, unaligned sequences are shown as transparent

chlorv-1..227

- Sequence-based annotation for chlorv-1..227 is hypothetical protein
- No significant structural hit found

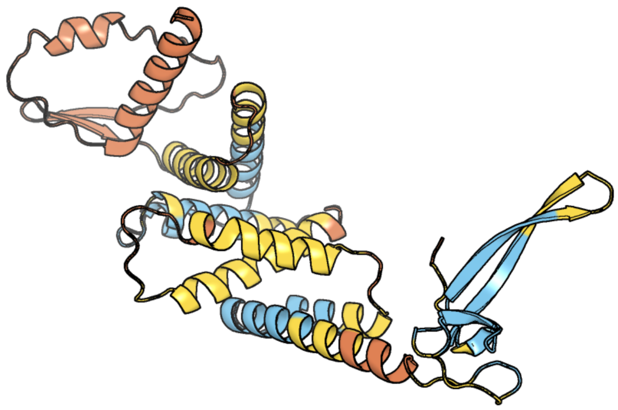

Figure 227: predicted structure of chlorv-1..227

chlorv-1..228

- Sequence-based annotation for chlorv-1..228 is putative Cysteine peptidase (DUF1796)
- No significant structural hit found

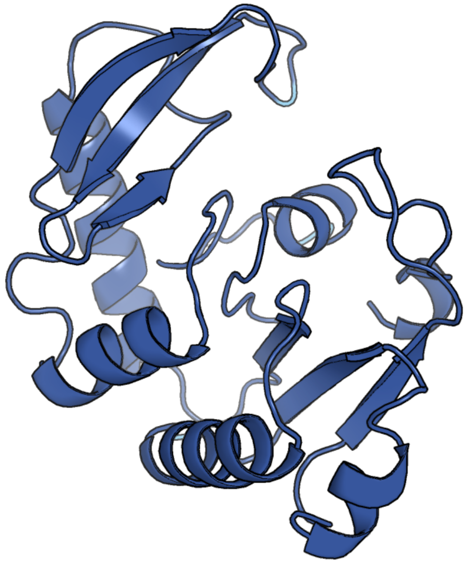

Figure 228: predicted structure of chlorv-1..228

chlorv-1..229

- Sequence-based annotation for chlorv-1..229 is hypothetical protein
- No significant structural hit found

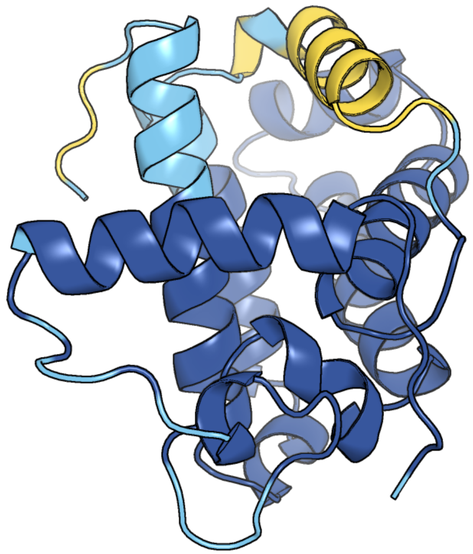

Figure 229: predicted structure of chlorv-1..229

chlorv-1..230

- Sequence-based annotation for chlorv-1..230 is hypothetical protein
- No significant structural hit found

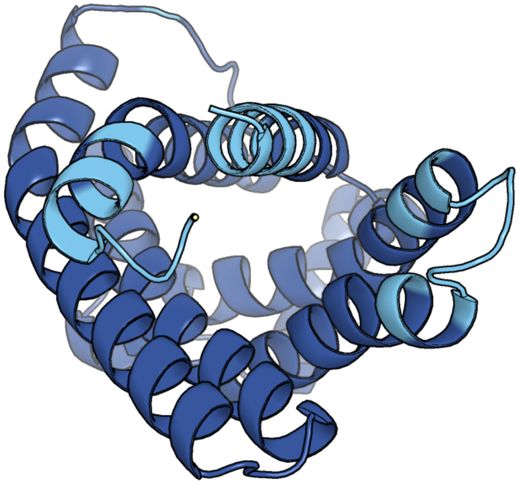

Figure 230: predicted structure of chlorv-1..230

chlorv-1..231

- Sequence-based annotation for chlorv-1..231 is hypothetical protein
- Best hit was 5dku chain B: Prex DNA polymerase

target	prob	fident	alnlen	evalue	theader
5dku-assembly2.cif.gz_B	1	0.189	327	3.536e-06	C-terminal His tagged apPOL exonuclease mutant
5dkt-assembly1.cif.gz_A	1	0.182	329	3.896e-06	N-terminal His tagged apPOL exonuclease mutant
5dku-assembly1.cif.gz_A	1	0.195	302	6.641e-06	C-terminal His tagged apPOL exonuclease mutant

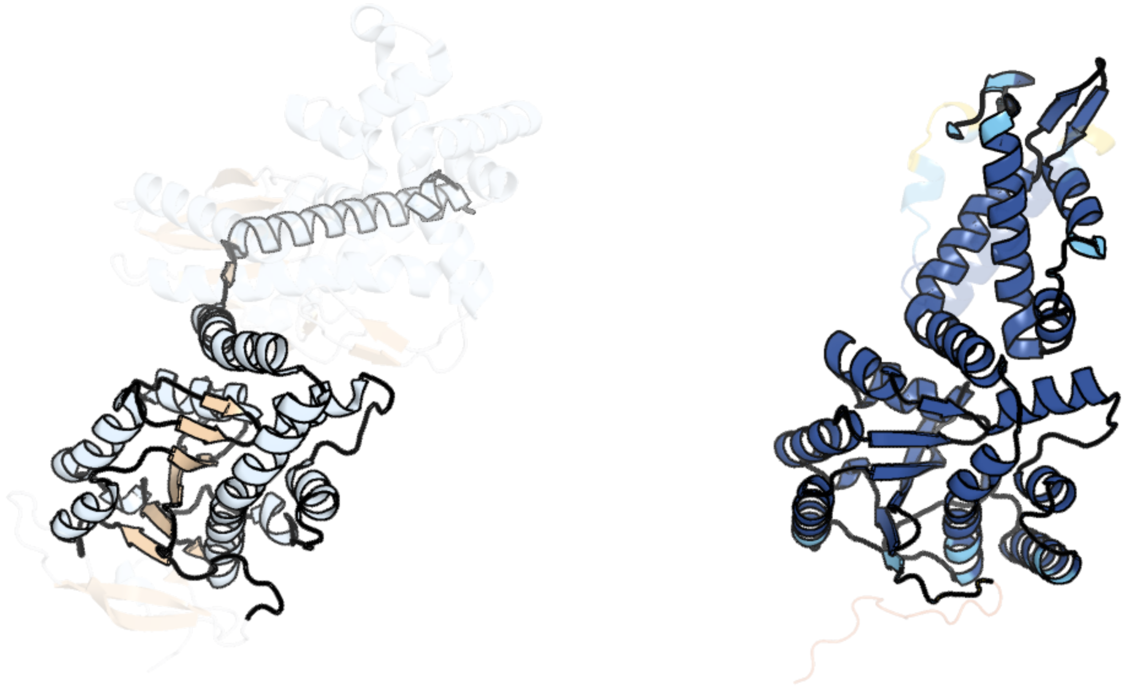

Figure 231: left: reference structure of 5dku chain B. right: predicted structure of chlorv-1..231, unaligned sequences are shown as transparent

chlorv-1..232

- Sequence-based annotation for chlorv-1..232 is hypothetical protein
- Best hit was 1yvp chain B: 60-kDa SS-A/Ro ribonucleoprotein

target	prob	fident	alnlen	evalue	theader
1yvp-assembly2.cif.gz_B	1	0.13	588	1.146e-08	Ro autoantigen complexed with RNAs
8e59-assembly1.cif.gz_D	1	0.129	217	0.004582	Human L-type voltage-gated calcium channel Cav1.3 in the presence of Amiodarone at 3.1 Angstrom resolution
7n1o-assembly1.cif.gz_A	0.999	0.086	277	0.006035	The von Willebrand factor A domain of human capillary morphogenesis gene II, flexibly fused to the 1TEL crystallization chaperone

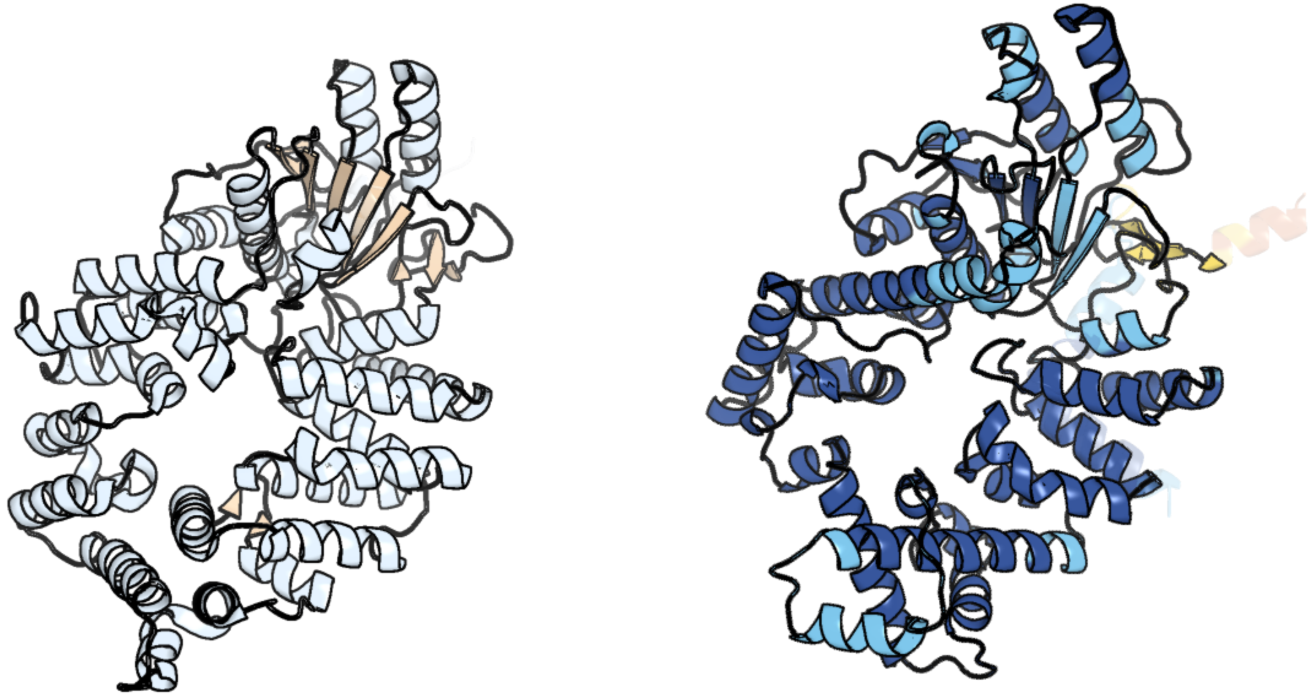

Figure 232: left: reference structure of 1yvp chain B. right: predicted structure of chlorv-1..232, unaligned sequences are shown as transparent

chlorv-1..233

- Sequence-based annotation for chlorv-1..233 is hypothetical protein
- Best hit was 6gci chain A: mitochondrial ADP/ATP carrier

target	prob	fident	alnlen	evalue	theader
6gci-assembly1.cif.gz_A	1	0.169	284	3.96e-06	Structure of the bongkrekic acid-inhibited mitochondrial ADP/ATP carrier
8g8w-assembly1.cif.gz_A	1	0.192	286	1.253e-05	Molecular mechanism of nucleotide inhibition of human uncoupling protein 1
8gym-assembly1.cif.gz_m2	1	0.128	288	0.0003796	Cryo-EM structure of Tetrahymena thermophila respiratory mega-complex MC IV2+(I+III2+II)2

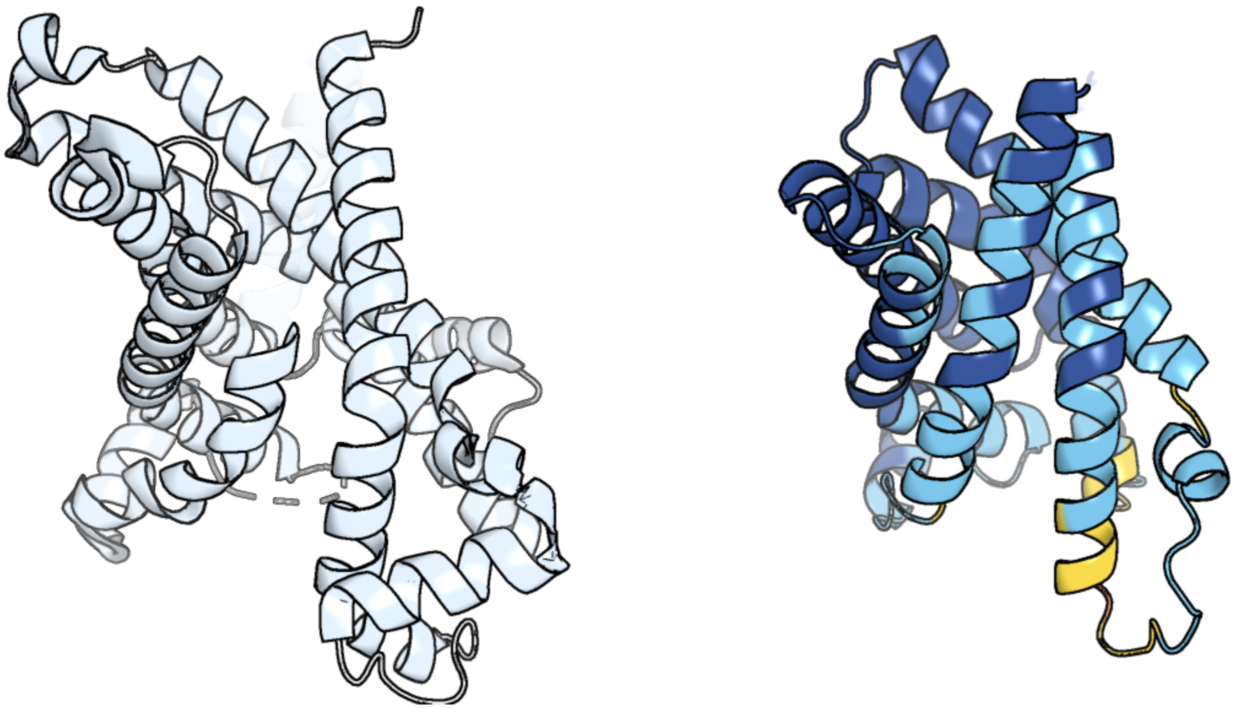

Figure 233: left: reference structure of 6gci chain A. right: predicted structure of chlorv-1..233, unaligned sequences are shown as transparent

chlorv-1..234

- Sequence-based annotation for chlorv-1..234 is hypothetical protein
- No significant structural hit found

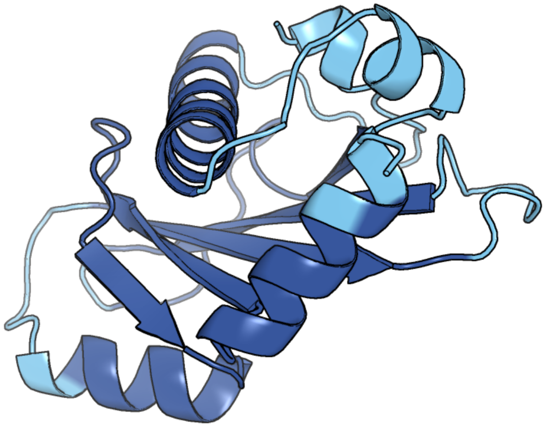

Figure 234: predicted structure of chlorv-1..234

chlorv-1..235

- Sequence-based annotation for chlorv-1..235 is hypothetical protein
- No significant structural hit found

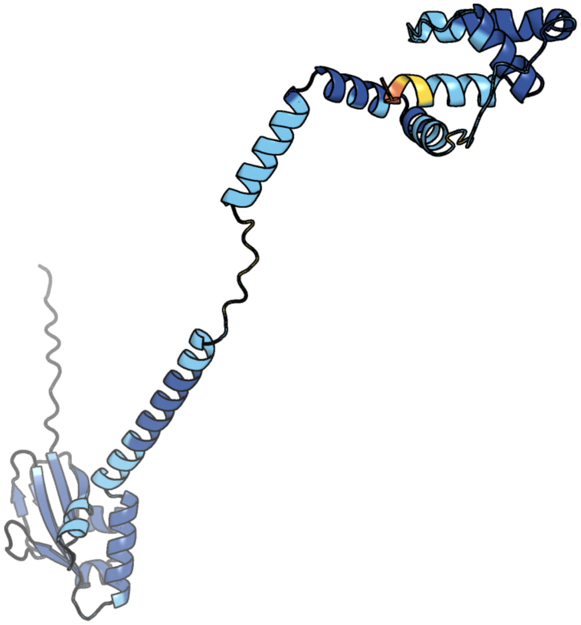

Figure 235: predicted structure of chlorv-1..235

chlorv-1..236

- Sequence-based annotation for chlorv-1..236 is hypothetical protein
- No significant structural hit found

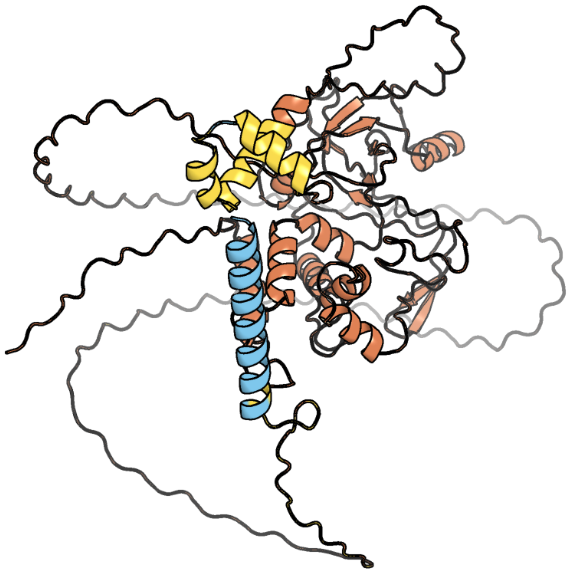

Figure 236: predicted structure of chlorv-1..236

chlorv-1..237

- Sequence-based annotation for chlorv-1..237 is hypothetical protein
- No significant structural hit found

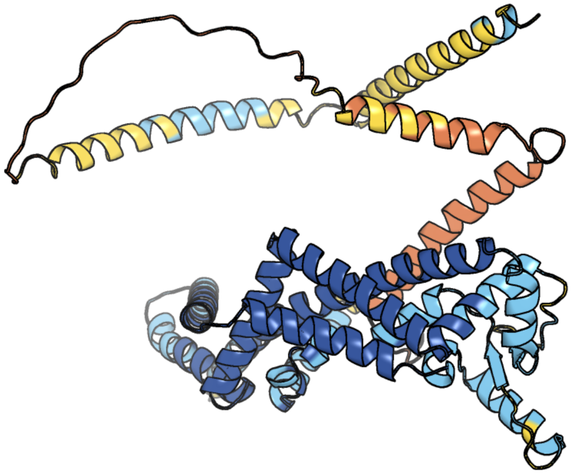

Figure 237: predicted structure of chlorv-1..237

chlorv-1..238

- Sequence-based annotation for chlorv-1..238 is hypothetical protein
- No significant structural hit found

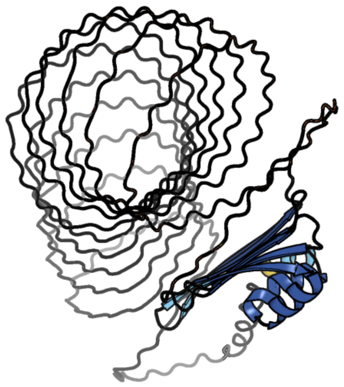

Figure 238: predicted structure of chlorv-1..238

chlorv-1..239

- Sequence-based annotation for chlorv-1..239 is hypothetical protein
- No significant structural hit found

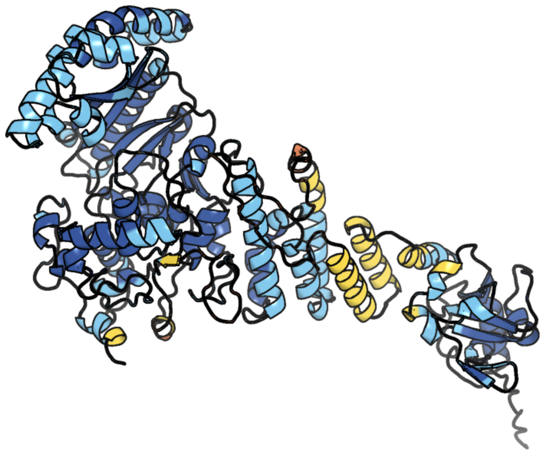

Figure 239: predicted structure of chlorv-1..239

chlorv-1..240

- Sequence-based annotation for chlorv-1..240 is hypothetical protein
- Best hit was 8cdj chain C: Cullin-1

target	prob	fident	alnlen	evalue	theader
8cdj-assembly1.cif.gz_C	1	0.103	761	4.624e-06	CAND1 b-hairpin++-SCF-SKP2 CAND1 rolling SCF engaged
6v9i-assembly1.cif.gz_C	1	0.117	783	5.717e-06	cryo-EM structure of Cullin5 bound to RING-box protein 2 (Cul5-Rbx2)
8or3-assembly1.cif.gz_A	1	0.1	750	7.069e-06	CAND1-CUL1-RBX1-SKP1-SKP2-DCNL1

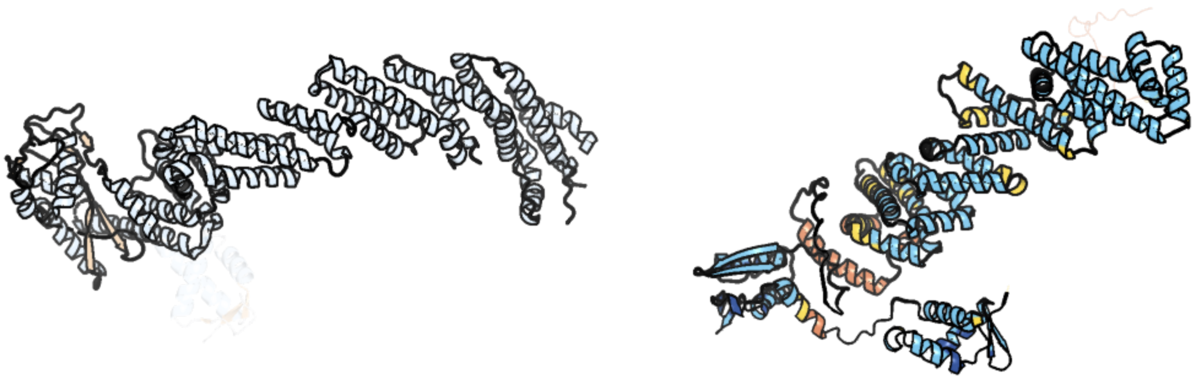

Figure 240: left: reference structure of 8cdj chain C. right: predicted structure of chlorv-1..240, unaligned sequences are shown as transparent

chlorv-1..241

- Sequence-based annotation for chlorv-1..241 is hypothetical protein
- No significant structural hit found

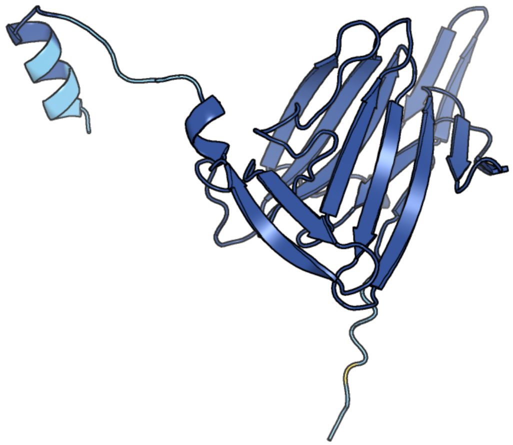

Figure 241: predicted structure of chlorv-1..241

chlorv-1..242

- Sequence-based annotation for chlorv-1..242 is hypothetical protein
- No significant structural hit found

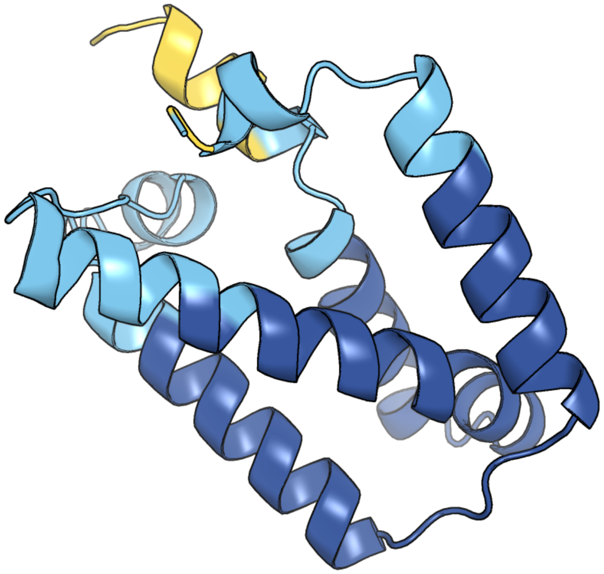

Figure 242: predicted structure of chlorv-1..242

chlorv-1..243

- Sequence-based annotation for chlorv-1..243 is putative DUF5755 domain-containing protein
- No significant structural hit found

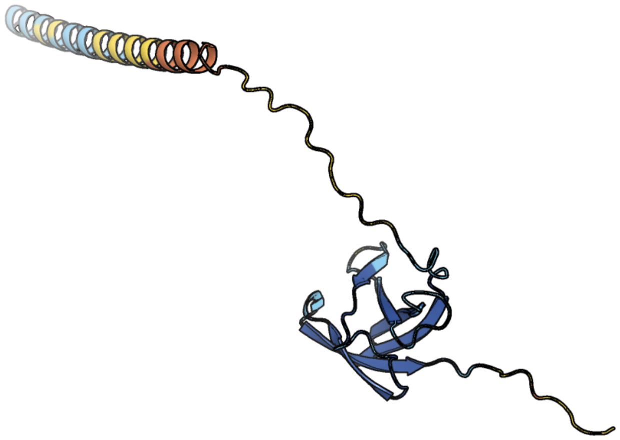

Figure 243: predicted structure of chlorv-1..243

chlorv-1..244

- Sequence-based annotation for chlorv-1..244 is hypothetical protein
- No significant structural hit found

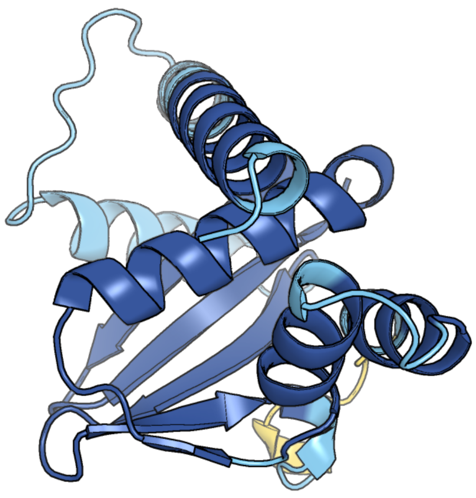

Figure 244: predicted structure of chlorv-1..244

chlorv-1..245

- Sequence-based annotation for chlorv-1..245 is putative mRNA decapping protein 2 NUDIX hydrolase domain
- Best hit was 7dnu chain A: mRNA-decapping protein g5R

target	prob	fident	alnlen	evalue	theader
7dnu-assembly1.cif.gz_A	1	0.217	280	1.487e-10	mRNA-decapping enzyme g5Rp with inhibitor insp6 complex
5j3t-assembly1.cif.gz_B	0.997	0.148	236	0.0002148	Crystal structure of S. pombe Dcp2:Dcp1:Edc1 mRNA decapping complex
4kg4-assembly2.cif.gz_B	1	0.243	119	0.0003015	Crystal structure of Saccharomyces cerevisiae Dcp2 Nudix domain (E198Q mutation)

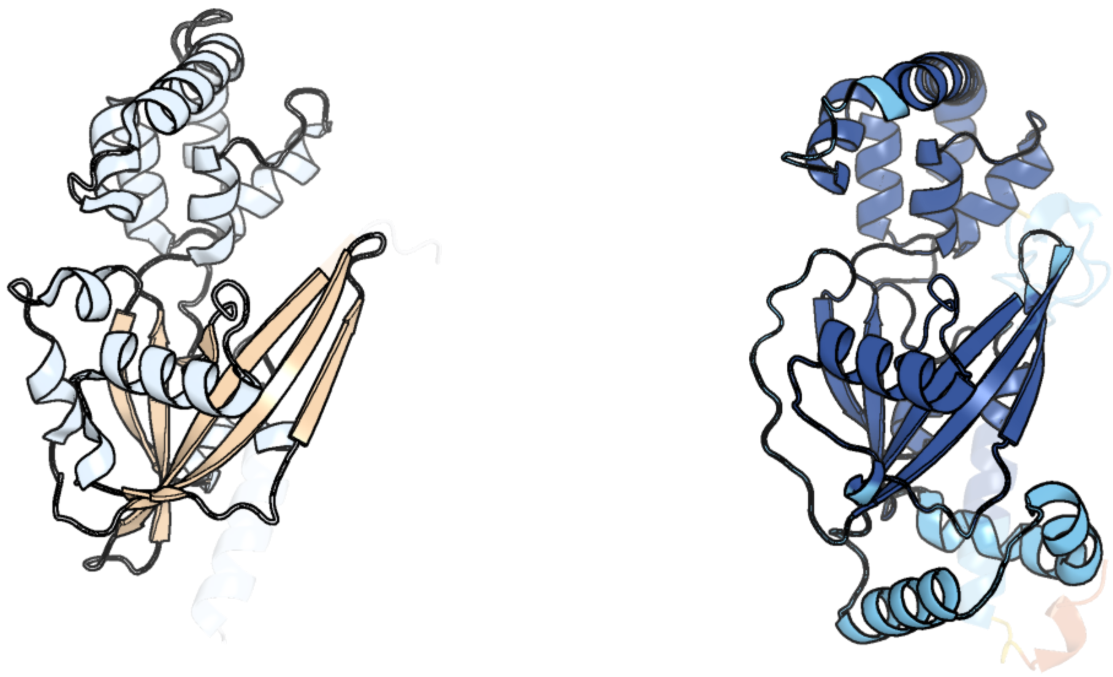

Figure 245: left: reference structure of 7dnu chain A. right: predicted structure of chlorv-1..245, unaligned sequences are shown as transparent

chlorv-1..246

- Sequence-based annotation for chlorv-1..246 is hypothetical protein
- No significant structural hit found

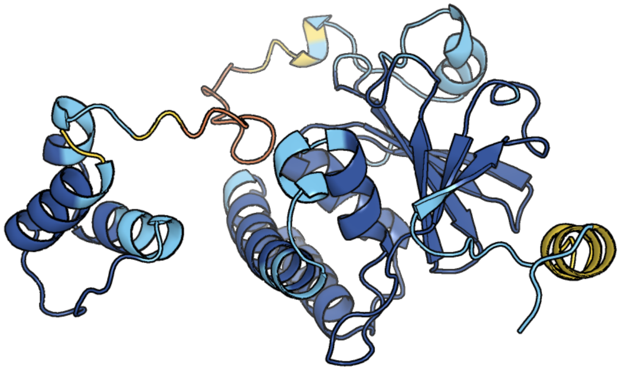

Figure 246: predicted structure of chlorv-1..246

chlorv-1..247

- Sequence-based annotation for chlorv-1..247 is putative Erv1/Alr family protein/Oxidoreductase
- Best hit was 3gwn chain B: Probable FAD-linked sulfhydryl oxidase R596

target	prob	fident	alnlen	evalue	theader
3gwn-assembly1.cif.gz_B	1	0.372	110	3.74e-07	Crystal structure of the FAD binding domain from mimivirus sulfhydryl oxidase R596
3td7-assembly1.cif.gz_A-2	1	0.371	113	5.545e-07	Crysal structure of the mimivirus sulfhydryl oxidase R596
2hj3-assembly1.cif.gz_A	1	0.265	98	3.972e-06	Structure of the Arabidopsis Thaliana Erv1 Thiol Oxidase

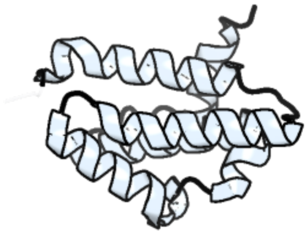

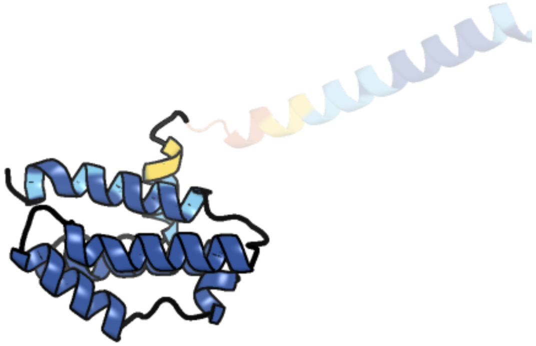

Figure 247: left: reference structure of 3gwn chain B. right: predicted structure of chlorv-1..247, unaligned sequences are shown as transparent

chlorv-1..248

- Sequence-based annotation for chlorv-1..248 is hypothetical protein
- No significant structural hit found

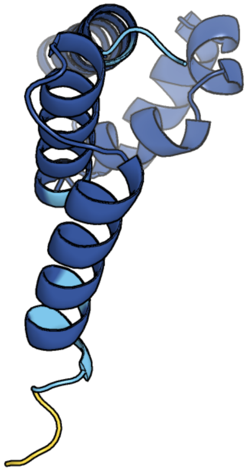

Figure 248: predicted structure of chlorv-1..248

chlorv-1..249

- Sequence-based annotation for chlorv-1..249 is putative Methyltransferase
- Best hit was 4n48 chain B: Cap-specific mRNA (nucleoside-2'-O-)-methyltransferase 1

target	prob	fident	alnlen	evalue	theader
4n48-assembly1.cif.gz_B	1	0.178	442	2.201e-15	Cap-specific mRNA (nucleoside-2'-O-)-methyltransferase 1 Protein in complex with capped RNA fragment
8p4e-assembly1.cif.gz_O	1	0.183	435	3.232e-15	Structural insights into human co-transcriptional capping - structure 5
4n48-assembly2.cif.gz_A	1	0.174	436	3.558e-15	Cap-specific mRNA (nucleoside-2'-O-)-methyltransferase 1 Protein in complex with capped RNA fragment

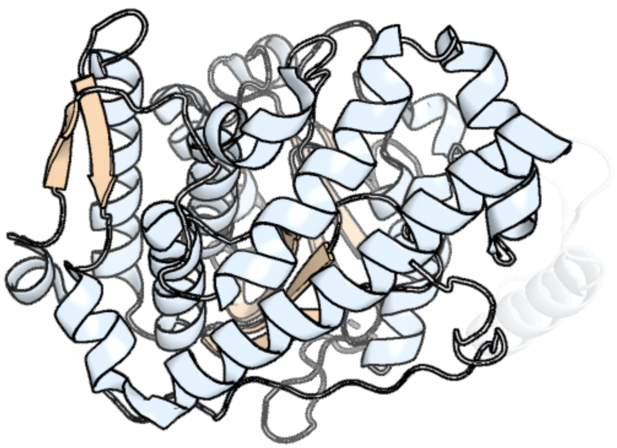

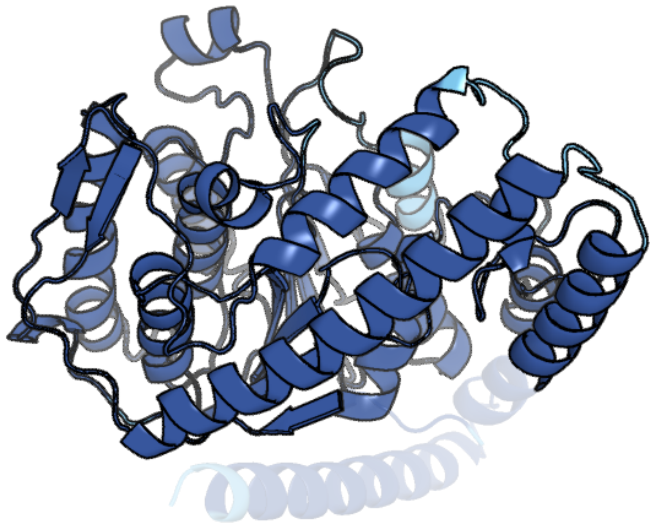

Figure 249: left: reference structure of 4n48 chain B. right: predicted structure of chlorv-1..249, unaligned sequences are shown as transparent

chlorv-1..250

- Sequence-based annotation for chlorv-1..250 is hypothetical protein
- No significant structural hit found

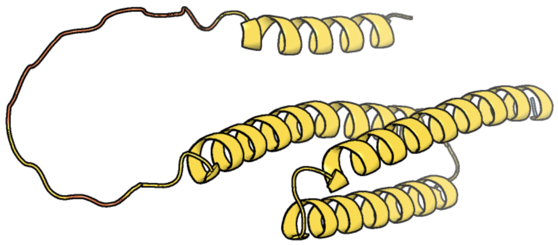

Figure 250: predicted structure of chlorv-1..250

chlorv-1..251

- Sequence-based annotation for chlorv-1..251 is hypothetical protein
- Best hit was 7nvu chain G: DNA-directed RNA polymerase II subunit RPB7

target	prob	fident	alnlen	evalue	theader
7nvu-assembly1.cif.gz_G	1	0.218	169	6.014e-11	RNA polymerase II core pre-initiation complex with open promoter DNA
1go3-assembly1.cif.gz_E	1	0.194	159	7.185e-10	Structure of an archeal homolog of the eukaryotic RNA polymerase II RPB4/RPB7 complex
3h0g-assembly1.cif.gz_G	1	0.182	164	8.478e-10	RNA Polymerase II from Schizosaccharomyces pombe

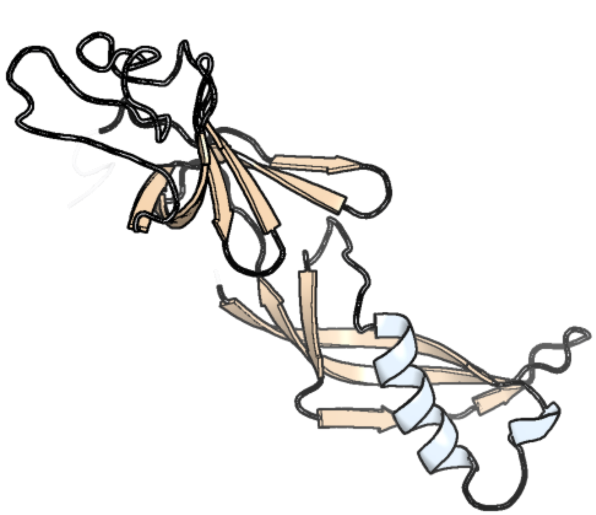

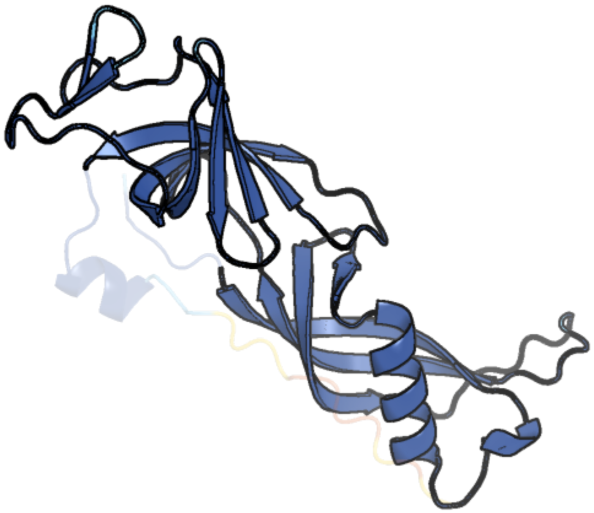

Figure 251: left: reference structure of 7nvu chain G. right: predicted structure of chlorv-1..251, unaligned sequences are shown as transparent

chlorv-1..252

- Sequence-based annotation for chlorv-1..252 is putative Ankyrin repeat protein
- Best hit was 5lee chain A: DDD_D12_12_D12_12_D12

target	prob	fident	alnlen	evalue	theader
5lee-assembly1.cif.gz_A	1	0.185	367	1.335e-08	Crystal structure of DARPin-DARPin rigid fusion, variant DDD_D12_12_D12_12_D12
5leb-assembly1.cif.gz_A	1	0.17	369	1.673e-08	Crystal structure of DARPin-DARPin rigid fusion, variant DDD_D12_06_D12_06_D12
5le8-assembly2.cif.gz_B	1	0.178	341	2.294e-08	Crystal structure of DARPin-DARPin rigid fusion, variant DD_D12_15_D12

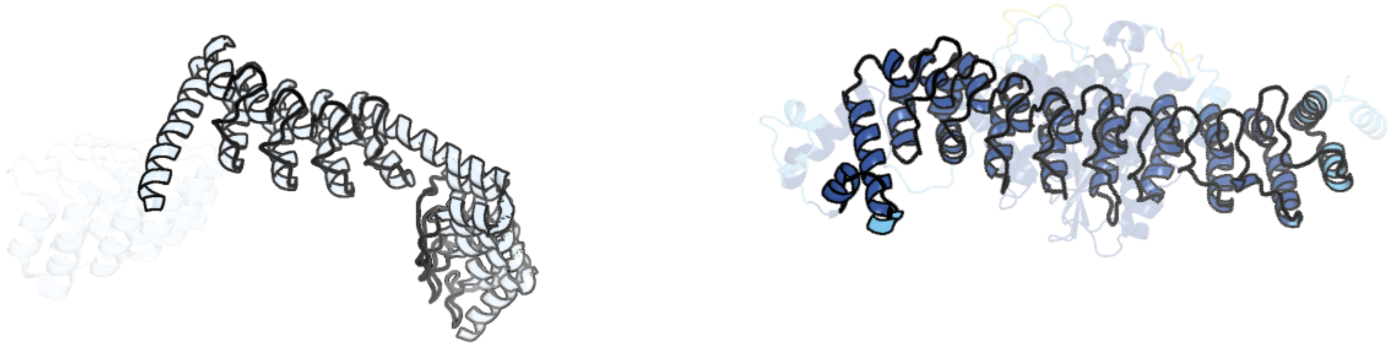

Figure 252: left: reference structure of 5lee chain A. right: predicted structure of chlorv-1..252, unaligned sequences are shown as transparent

chlorv-1..253

- Sequence-based annotation for chlorv-1..253 is putative RNA polymerase subunit 5
- Best hit was 7vba chain E: DNA-directed RNA polymerases I, II, and III subunit RPABC1

target	prob	fident	alnlen	evalue	theader
7vba-assembly1.cif.gz_E	1	0.231	207	4.32e-13	Structure of the pre state human RNA Polymerase I Elongation Complex
5iy8-assembly1.cif.gz_E	1	0.238	210	9.004e-13	Human holo-PIC in the initial transcribing state
8cen-assembly1.cif.gz_E	1	0.215	209	2.255e-12	Yeast RNA polymerase II transcription pre-initiation complex with core Mediator

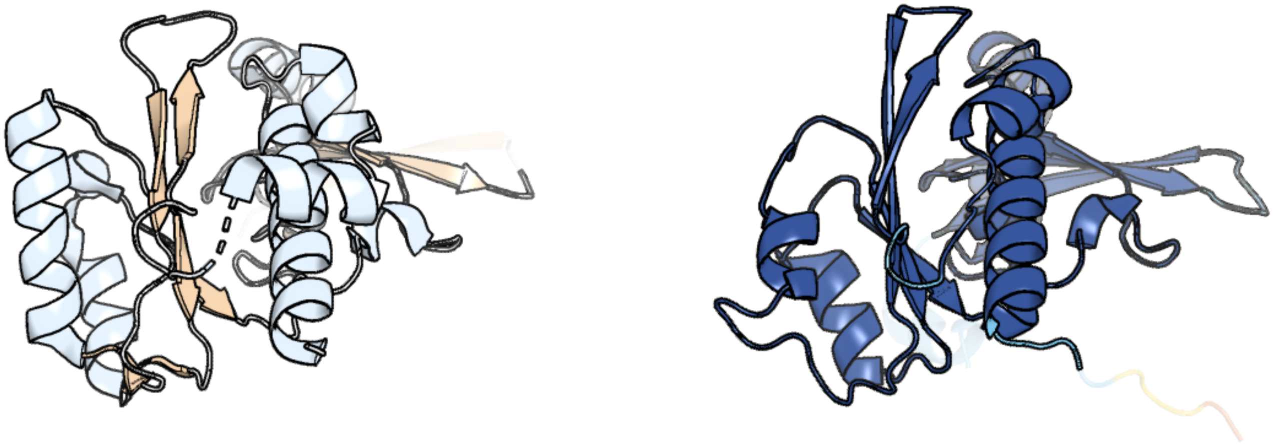

Figure 253: left: reference structure of 7vba chain E. right: predicted structure of chlorv-1..253, unaligned sequences are shown as transparent

chlorv-1..254

- Sequence-based annotation for chlorv-1..254 is hypothetical protein
- No significant structural hit found

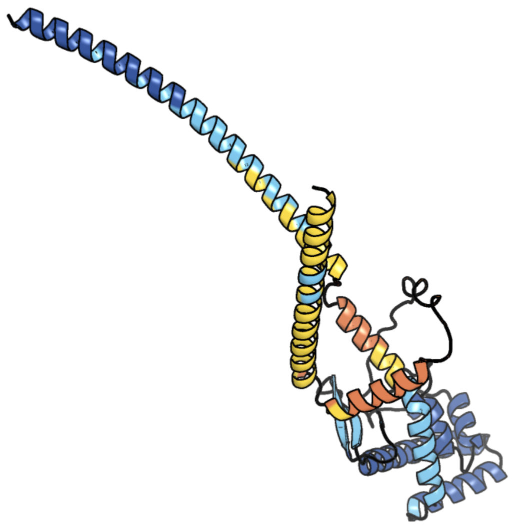

Figure 254: predicted structure of chlorv-1..254

chlorv-1..255

- Sequence-based annotation for chlorv-1..255 is putative Ubiquitin-activating enzyme
- Best hit was 6dc6 chain A: Ubiquitin-like modifier-activating enzyme 1

target	prob	fident	alnlen	evalue	theader
6dc6-assembly1.cif.gz_A	1	0.125	884	1.523e-32	Crystal structure of human ubiquitin activating enzyme E1 (Uba1) in complex with ubiquitin
6zhu-assembly4.cif.gz_G	1	0.138	885	4.203e-32	Yeast Uba1 in complex with Ubc3 and ATP
6zhs-assembly1.cif.gz_A	1	0.133	884	5.572e-32	Uba1 bound to two E2 (Ubc13) molecules

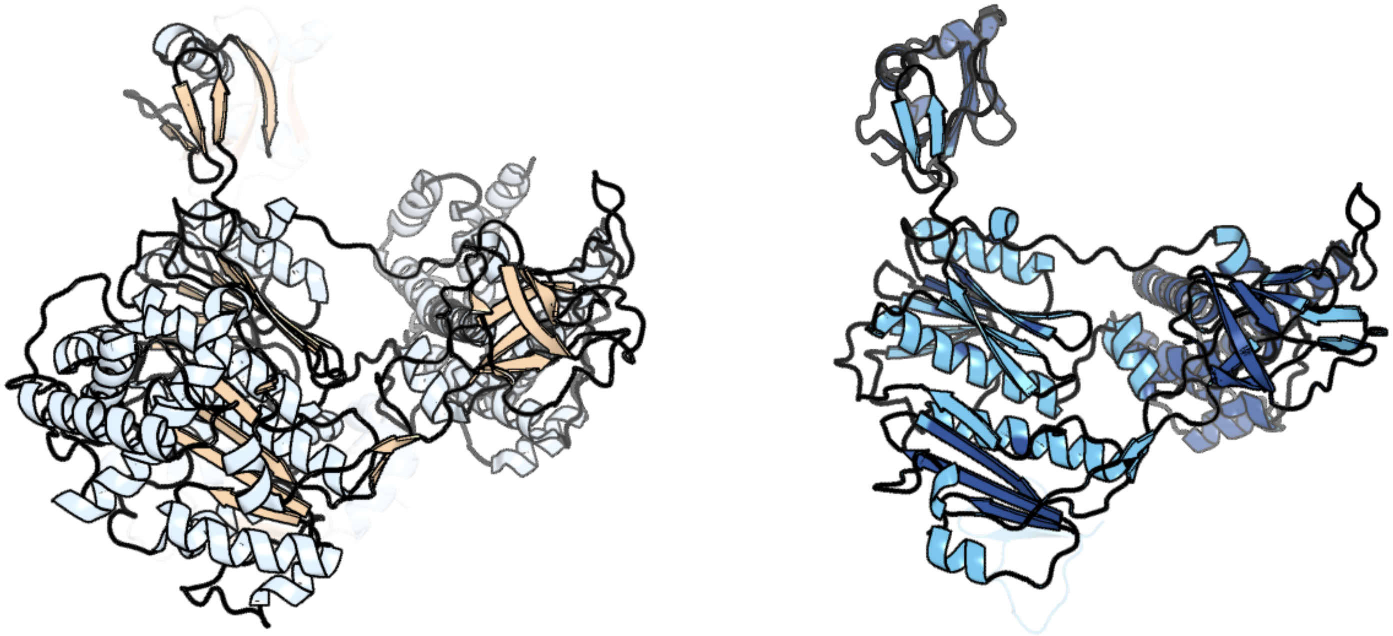

Figure 255: left: reference structure of 6dc6 chain A. right: predicted structure of chlorv-1..255, unaligned sequences are shown as transparent

chlorv-1..256

- Sequence-based annotation for chlorv-1..256 is putative Methionine aminopeptidase
- Best hit was 5lyx chain A: Methionine aminopeptidase 2

target	prob	fident	alnlen	evalue	theader
5lyx-assembly1.cif.gz_A	1	0.366	311	3.003e-32	CRYSTAL STRUCTURE OF HUMAN METHIONINE AMINOPEPTIDASE-2 IN COMPLEX; WITH AN INHIBITOR 5-((R)-1-[1,2,4]Triazolo[1,5-a]pyrimidin-7-yl-pyrrolidin-2-ylmethoxy)-isoquinoline
1kq9-assembly1.cif.gz_A	1	0.372	311	5.083e-32	Human methionine aminopeptidase type II in complex with L-methionine
1yw7-assembly1.cif.gz_A	1	0.372	311	1.295e-31	h-MetAP2 complexed with A444148

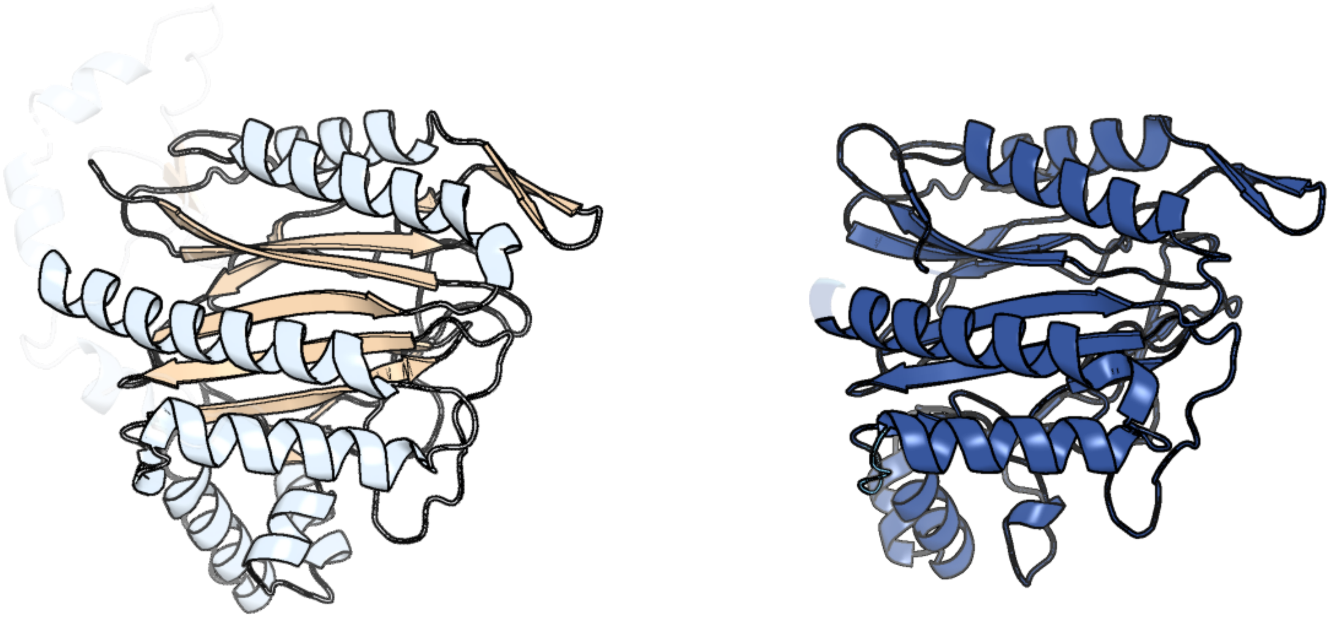

Figure 256: left: reference structure of 5lyx chain A. right: predicted structure of chlorv-1..256, unaligned sequences are shown as transparent

chlorv-1..257

- Sequence-based annotation for chlorv-1..257 is putative Ankyrin repeat protein
- Best hit was 5orm chain A: cPPR-Telo1

target	prob	fident	alnlen	evalue	theader
5orm-assembly1.cif.gz_A	1	0.117	374	9.735e-09	Crystal structure of designed cPPR-Telo1
5i9g-assembly1.cif.gz_C	1	0.09	355	9.735e-09	Crystal structure of designed pentatricopeptide repeat protein dPPR-U8C2 in complex with its target RNA U8C2
5i9f-assembly1.cif.gz_A	1	0.082	387	3.267e-08	Crystal structure of designed pentatricopeptide repeat protein dPPR-U10 in complex with its target RNA U10

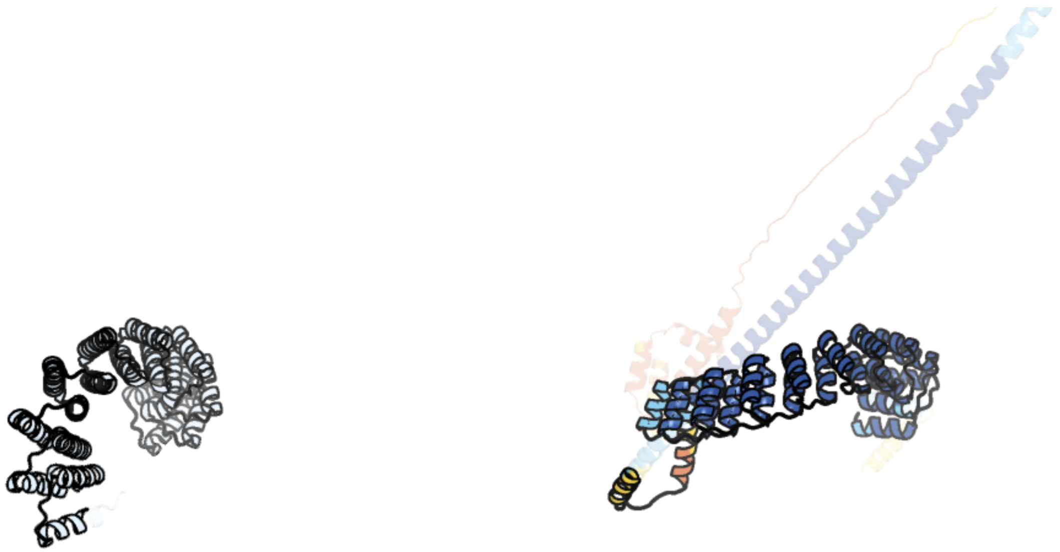

Figure 257: left: reference structure of 5orm chain A. right: predicted structure of chlorv-1..257, unaligned sequences are shown as transparent

chlorv-1..258

- Sequence-based annotation for chlorv-1..258 is hypothetical protein
- No significant structural hit found

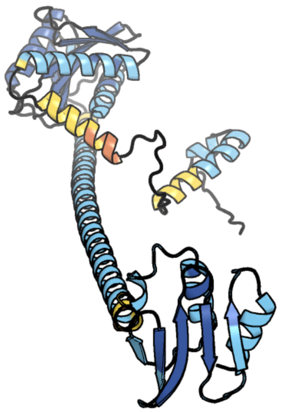

Figure 258: predicted structure of chlorv-1..258

chlorv-1..259

- Sequence-based annotation for chlorv-1..259 is hypothetical protein
- No significant structural hit found

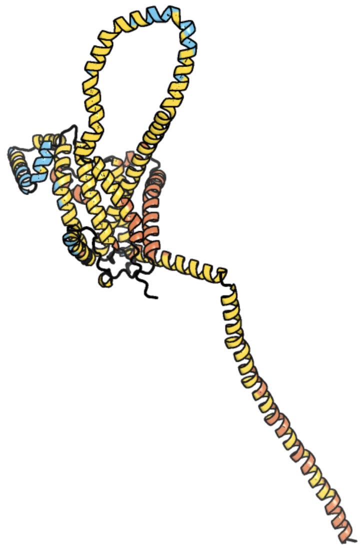

Figure 259: predicted structure of chlorv-1..259

chlorv-1..260

- Sequence-based annotation for chlorv-1..260 is hypothetical protein
- Best hit was 2jrs chain A: RNA-binding protein 39

target	prob	fident	alnlen	evalue	theader
2jrs-assembly1.cif.gz_A	1	0.163	98	8.287e-06	Solution NMR Structure of CAPER RRM2 Domain. Northeast Structural Genomics Target HR4730A
3hi9-assembly1.cif.gz_A	1	0.215	79	1.074e-05	The x-ray crystal structure of the first RNA recognition motif (RRM1) of the AU-rich element (ARE) binding protein HuR at 2.0 angstrom resolution
8fle-assembly1.cif.gz_SH	1	0.139	79	1.305e-05	Human nuclear pre-60S ribosomal subunit (State L2)

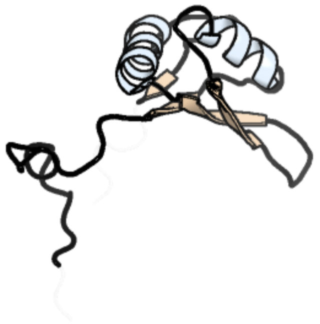

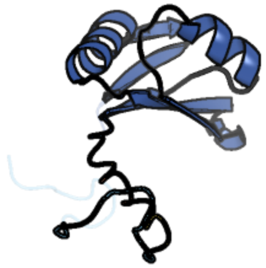

Figure 260: left: reference structure of 2jrs chain A. right: predicted structure of chlorv-1..260, unaligned sequences are shown as transparent

chlorv-1..261

- Sequence-based annotation for chlorv-1..261 is hypothetical protein
- No significant structural hit found

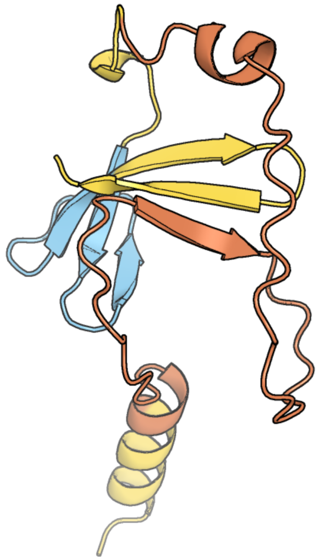

Figure 261: predicted structure of chlorv-1..261

chlorv-1..262

- Sequence-based annotation for chlorv-1..262 is hypothetical protein
- No significant structural hit found

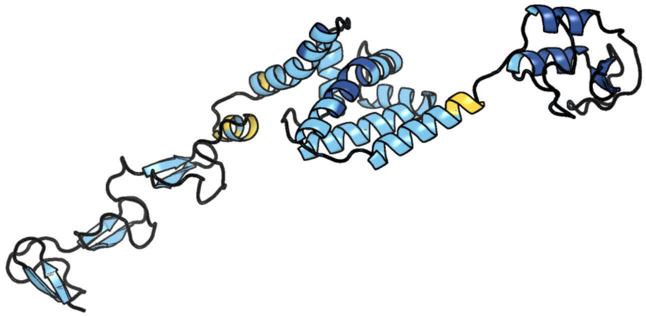

Figure 262: predicted structure of chlorv-1..262

chlorv-1..263

- Sequence-based annotation for chlorv-1..263 is hypothetical protein
- Best hit was 6er3 chain A: BNR/Asp-box repeat protein

target	prob	fident	alnlen	evalue	theader
6er3-assembly1.cif.gz_A	1	0.132	188	1.434e-08	Ruminococcus gnavus IT-sialidase CBM40 bound to alpha2,3 sialyllactose
6er4-assembly1.cif.gz_B	1	0.13	191	1.984e-08	Ruminococcus gnavus IT-sialidase CBM40 bound to alpha2,6 sialyllactose
6er2-assembly1.cif.gz_A	1	0.132	188	2.094e-08	Ruminococcus gnavus IT-sialidase CBM40

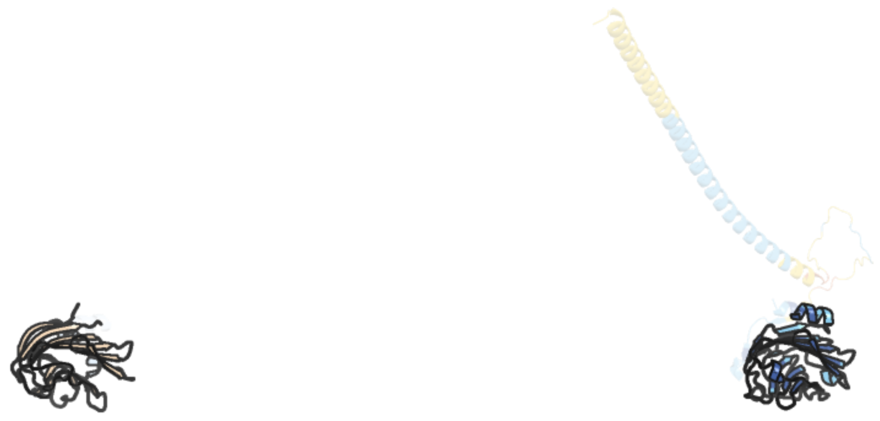

Figure 263: left: reference structure of 6er3 chain A. right: predicted structure of chlorv-1..263, unaligned sequences are shown as transparent

chlorv-1..264

- Sequence-based annotation for chlorv-1..264 is hypothetical protein
- No significant structural hit found

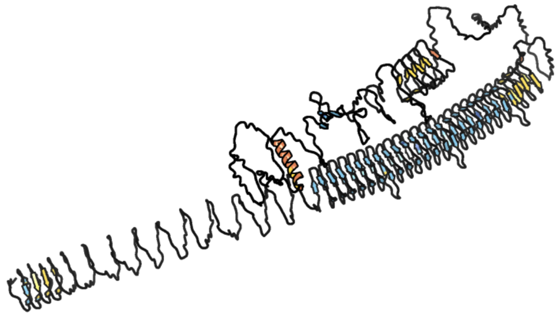

Figure 264: predicted structure of chlorv-1..264

chlorv-1..265

- Sequence-based annotation for chlorv-1..265 is hypothetical protein
- Best hit was 6g41 chain B: Minor capsid protein

target	prob	fident	alnlen	evalue	theader
6g41-assembly1.cif.gz_B	1	0.106	310	2.75e-09	Crystal structure of SeMet-labeled mavirus penton protein
6g42-assembly1.cif.gz_E	1	0.103	318	6.018e-09	Crystal structure of mavirus penton protein
6g41-assembly2.cif.gz_G	1	0.121	314	9.415e-09	Crystal structure of SeMet-labeled mavirus penton protein

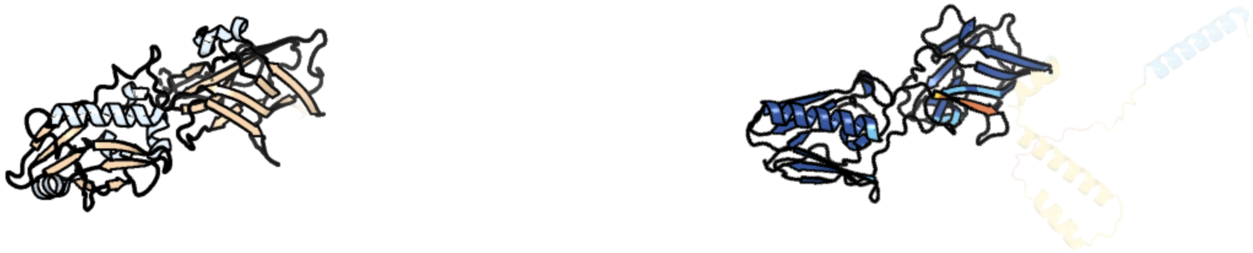

Figure 265: left: reference structure of 6g41 chain B. right: predicted structure of chlorv-1..265, unaligned sequences are shown as transparent

chlorv-1..266

- Sequence-based annotation for chlorv-1..266 is hypothetical protein
- Best hit was 6g41 chain G: Minor capsid protein

target	prob	fident	alnlen	evalue	theader
6g41-assembly2.cif.gz_G	1	0.091	305	2.458e-08	Crystal structure of SeMet-labeled mavirus penton protein
6g41-assembly1.cif.gz_B	1	0.1	310	2.596e-08	Crystal structure of SeMet-labeled mavirus penton protein
6g42-assembly1.cif.gz_E	1	0.09	320	9.085e-08	Crystal structure of mavirus penton protein

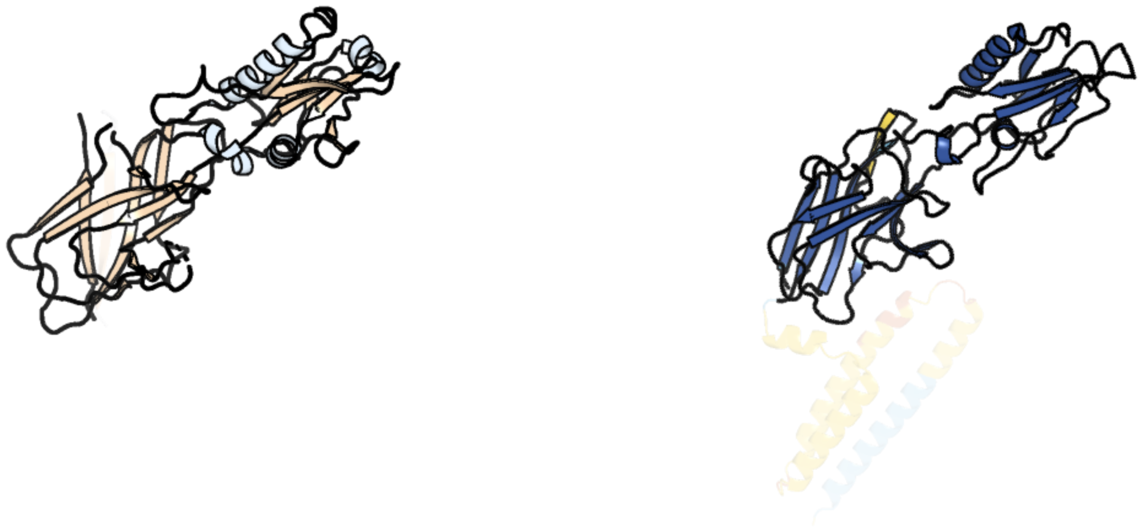

Figure 266: left: reference structure of 6g41 chain G. right: predicted structure of chlorv-1..266, unaligned sequences are shown as transparent

chlorv-1..267

- Sequence-based annotation for chlorv-1..267 is hypothetical protein
- No significant structural hit found

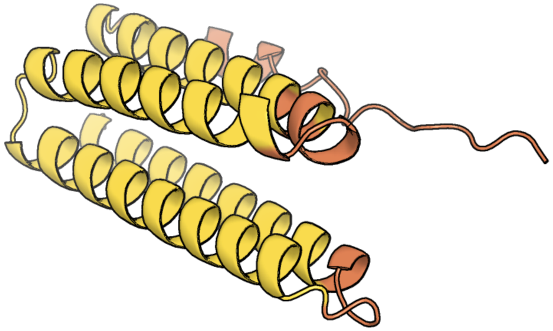

Figure 267: predicted structure of chlorv-1..267

chlorv-1..268

- Sequence-based annotation for chlorv-1..268 is hypothetical protein
- No significant structural hit found

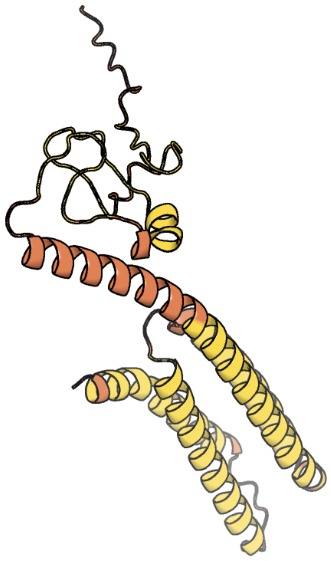

Figure 268: predicted structure of chlorv-1..268

chlorv-1..269

- Sequence-based annotation for chlorv-1..269 is hypothetical protein
- No significant structural hit found

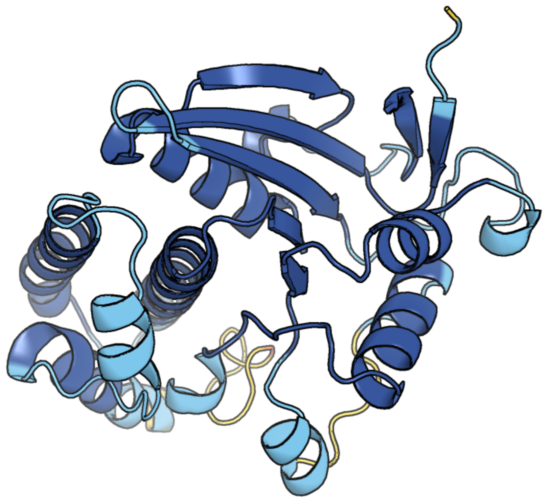

Figure 269: predicted structure of chlorv-1..269

chlorv-1..270

- Sequence-based annotation for chlorv-1..270 is putative Helcase
- Best hit was 8qca chain A: Antiviral helicase SKI2

target	prob	fident	alnlen	evalue	theader
8qca-assembly1.cif.gz_A	1	0.157	827	1.889e-16	CryoEM structure of a S. Cerevisiae Ski2387 complex in the closed state bound to RNA
4buj-assembly1.cif.gz_A	1	0.164	853	4.377e-16	Crystal structure of the S. cerevisiae Ski2-3-8 complex
4buj-assembly2.cif.gz_E	1	0.157	856	7.12e-16	Crystal structure of the S. cerevisiae Ski2-3-8 complex

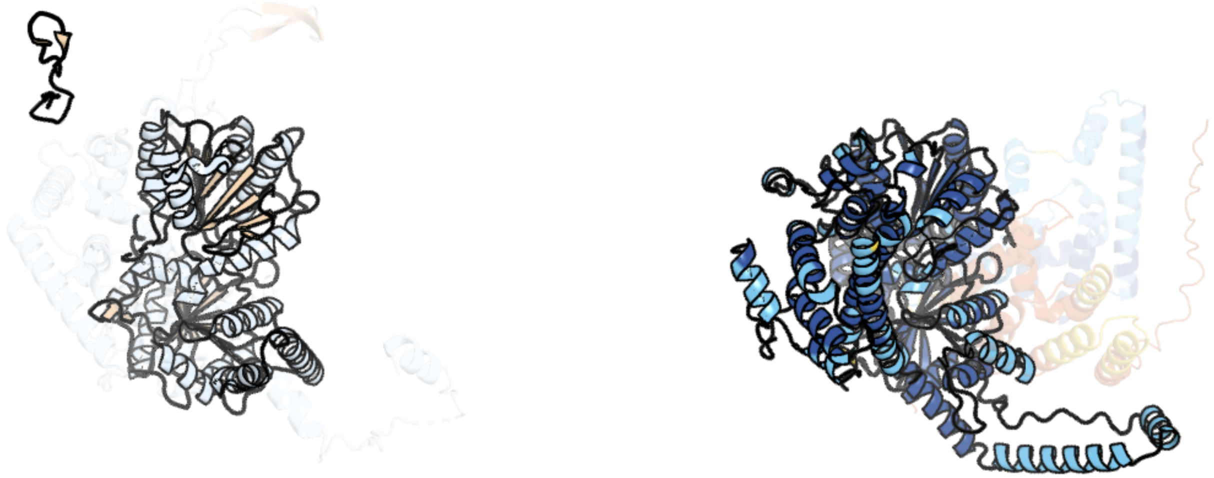

Figure 270: left: reference structure of 8qca chain A. right: predicted structure of chlorv-1..270, unaligned sequences are shown as transparent

chlorv-1..271

- Sequence-based annotation for chlorv-1..271 is hypothetical protein
- Best hit was 4ix3 chain A: MsStt7d protein

target	prob	fident	alnlen	evalue	theader
4ix3-assembly1.cif.gz_A	1	0.141	389	5.45e-08	Crystal structure of a Stt7 homolog from Micromonas algae
4mvf-assembly1.cif.gz_A	1	0.149	327	4.375e-07	Crystal Structure of Plasmodium falciparum CDPK2 complexed with inhibitor staurosporine
8u2o-assembly1.cif.gz_A	1	0.153	346	5.64e-07	Crystal Structure of Cdk-related protein kinase 6 (PK6) from Plasmodium falciparum in complex with inhibitor TCMDC-123995

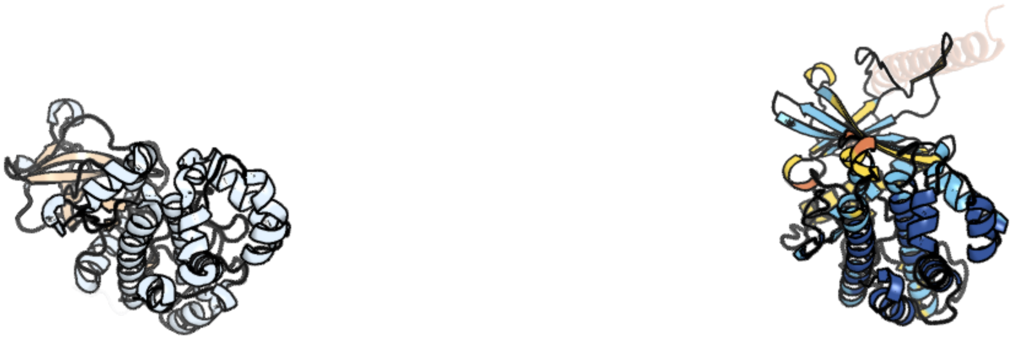

Figure 271: left: reference structure of 4ix3 chain A. right: predicted structure of chlorv-1..271, unaligned sequences are shown as transparent

chlorv-1..272

- Sequence-based annotation for chlorv-1..272 is hypothetical protein
- No significant structural hit found

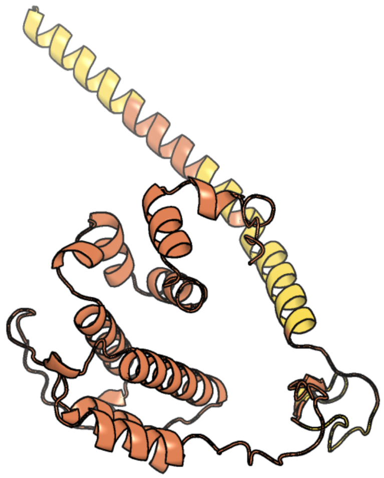

Figure 272: predicted structure of chlorv-1..272

chlorv-1..273

- Sequence-based annotation for chlorv-1..273 is hypothetical protein
- No significant structural hit found

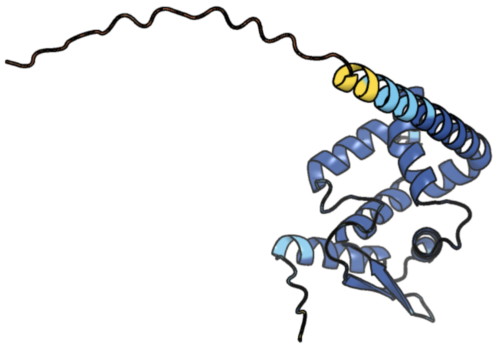

Figure 273: predicted structure of chlorv-1..273

chlorv-1..274

- Sequence-based annotation for chlorv-1..274 is hypothetical protein
- No significant structural hit found

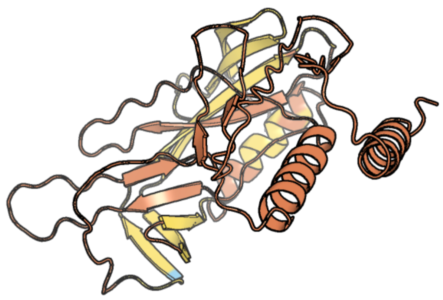

Figure 274: predicted structure of chlorv-1..274

chlorv-1..275

- Sequence-based annotation for chlorv-1..275 is hypothetical protein
- Best hit was 7yeq chain A: CP312R

target	prob	fident	alnlen	evalue	theader
7yeq-assembly1.cif.gz_A	1	0.082	254	4.201e-06	Structural insight into African Swine Fever Virus CP312R protein reveals it as a single-stranded DNA binding protein
5odk-assembly1.cif.gz_A	0.999	0.111	198	0.0003457	Single-stranded DNA-binding protein from bacteriophage Enc34, C-terminal truncation
5odj-assembly1.cif.gz_A	0.999	0.131	198	0.0004677	Single-stranded DNA-binding protein from bacteriophage Enc34

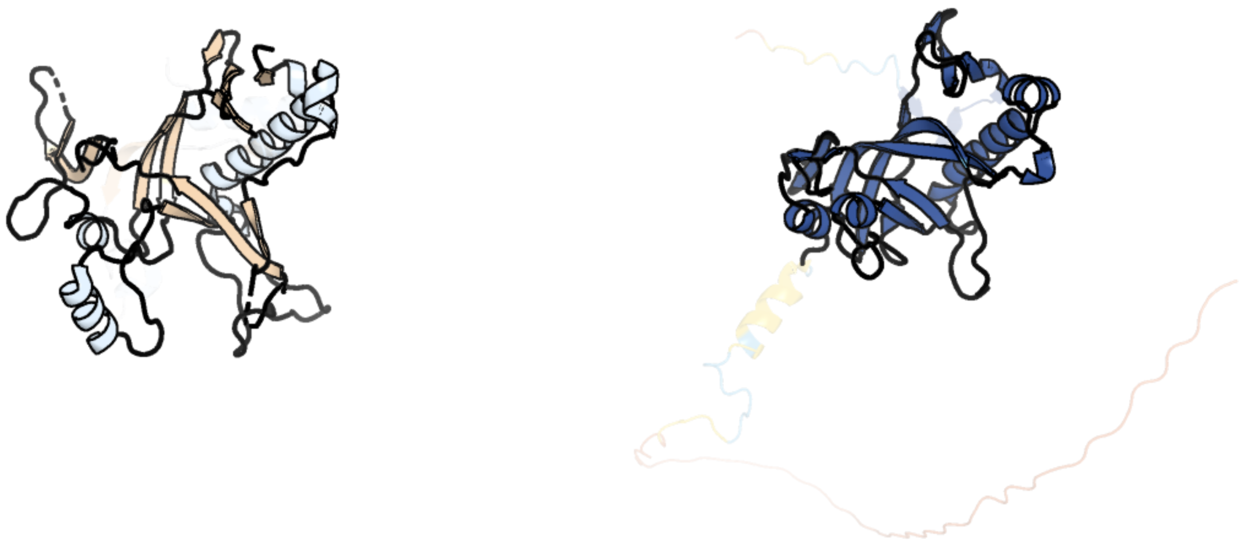

Figure 275: left: reference structure of 7yeq chain A. right: predicted structure of chlorv-1..275, unaligned sequences are shown as transparent

chlorv-1..276

- Sequence-based annotation for chlorv-1..276 is hypothetical protein
- No significant structural hit found

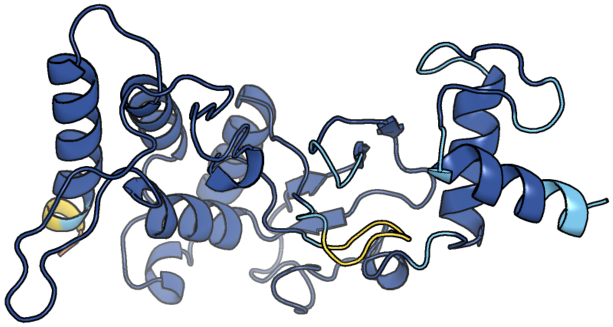

Figure 276: predicted structure of chlorv-1..276

chlorv-1..277

- Sequence-based annotation for chlorv-1..277 is putative Helicase
- Best hit was 2vsx chain A: ATP-DEPENDENT RNA HELICASE EIF4A

target	prob	fident	alnlen	evalue	theader
2vsx-assembly1.cif.gz_A	1	0.363	380	2.047e-35	Crystal Structure of a Translation Initiation Complex
8c6j-assembly1.cif.gz_7	1	0.384	377	5.216e-35	Human spliceosomal PM5 C* complex
1fuu-assembly1.cif.gz_B	1	0.375	376	6.204e-34	YEAST INITIATION FACTOR 4A

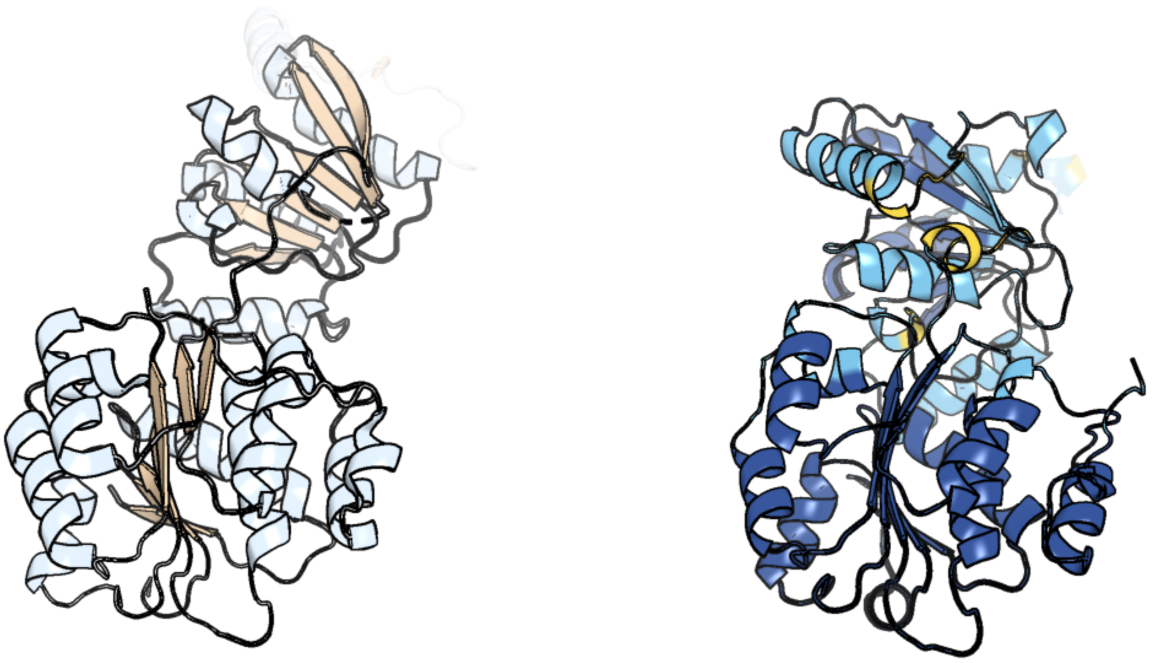

Figure 277: left: reference structure of 2vsx chain A. right: predicted structure of chlorv-1..277, unaligned sequences are shown as transparent

chlorv-1..278

- Sequence-based annotation for chlorv-1..278 is putative Erv1/Alr family protein/Oxidoreductase
- Best hit was 3gwn chain B: Probable FAD-linked sulfhydryl oxidase R596

target	prob	fident	alnlen	evalue	theader
3gwn-assembly1.cif.gz_B	1	0.415	113	6.397e-09	Crystal structure of the FAD binding domain from mimivirus sulfhydryl oxidase R596
3td7-assembly1.cif.gz_A-2	1	0.421	114	4.138e-08	Crysal structure of the mimivirus sulfhydryl oxidase R596
2hj3-assembly1.cif.gz_A	1	0.323	102	6.468e-06	Structure of the Arabidopsis Thaliana Erv1 Thiol Oxidase

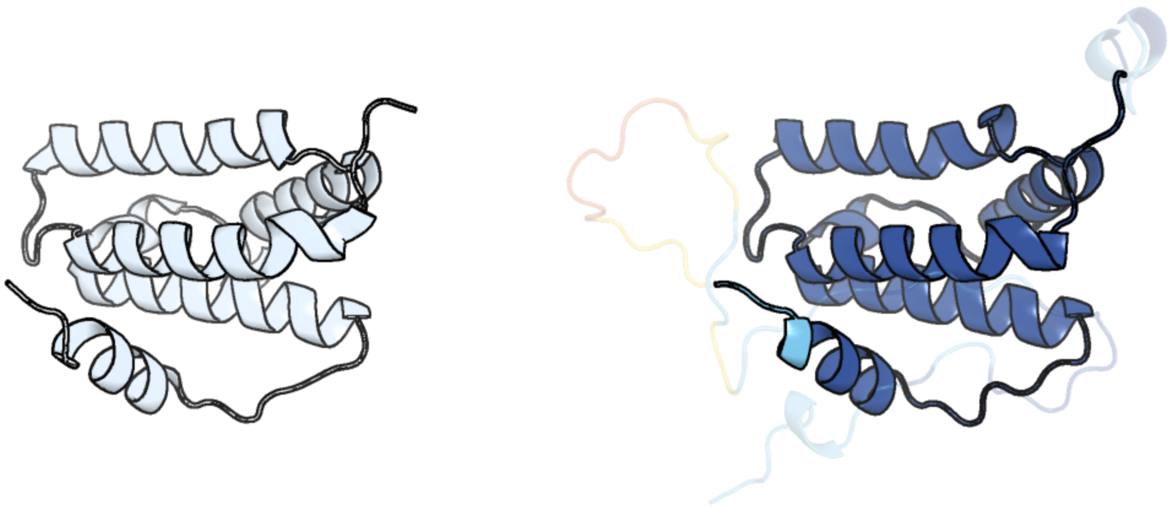

Figure 278: left: reference structure of 3gwn chain B. right: predicted structure of chlorv-1..278, unaligned sequences are shown as transparent

chlorv-1..279

- Sequence-based annotation for chlorv-1..279 is hypothetical protein
- Best hit was 8h2f chain A: DnaQ

target	prob	fident	alnlen	evalue	theader
8h2f-assembly1.cif.gz_A	1	0.161	204	4.036e-09	Crystal structure of DnaQ domain in complex witn TMP of Streptococcus thermophilus strain DGCC 7710
2f96-assembly1.cif.gz_B	1	0.085	223	2.529e-07	2.1 A crystal structure of Pseudomonas aeruginosa rnase T (Ribonuclease T)
3nh2-assembly1.cif.gz_A	1	0.119	218	2.861e-07	Crystal structure of RNase T in complex with a stem DNA with a 3' overhang

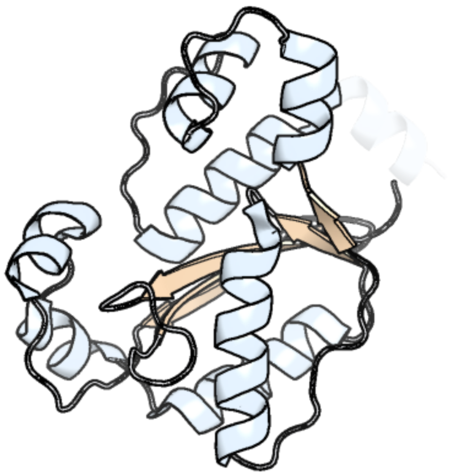

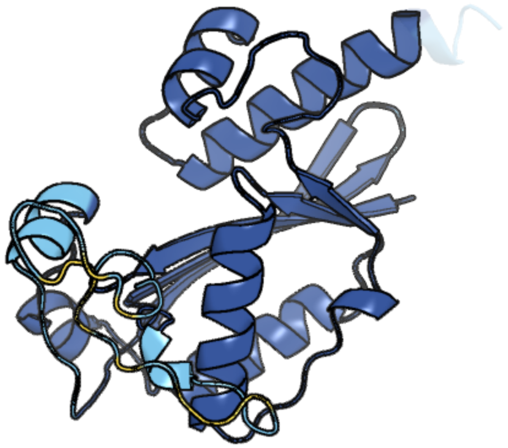

Figure 279: left: reference structure of 8h2f chain A. right: predicted structure of chlorv-1..279, unaligned sequences are shown as transparent

chlorv-1..280

- Sequence-based annotation for chlorv-1..280 is hypothetical protein
- Best hit was 3dkq chain B: PKHD-type hydroxylase Sbal_3634

target	prob	fident	alnlen	evalue	theader
3dkq-assembly1.cif.gz_B-2	1	0.167	221	1.075e-06	Crystal structure of Putative Oxygenase (YP_001051978.1) from SHEWANELLA BALTICA OS155 at 2.26 A resolution
3dkq-assembly1.cif.gz_C-2	1	0.148	235	2.239e-06	Crystal structure of Putative Oxygenase (YP_001051978.1) from SHEWANELLA BALTICA OS155 at 2.26 A resolution
4j25-assembly7.cif.gz_G	1	0.138	195	3.312e-06	Crystal structure of a Pseudomonas putida prolyl-4-hydroxylase (P4H)

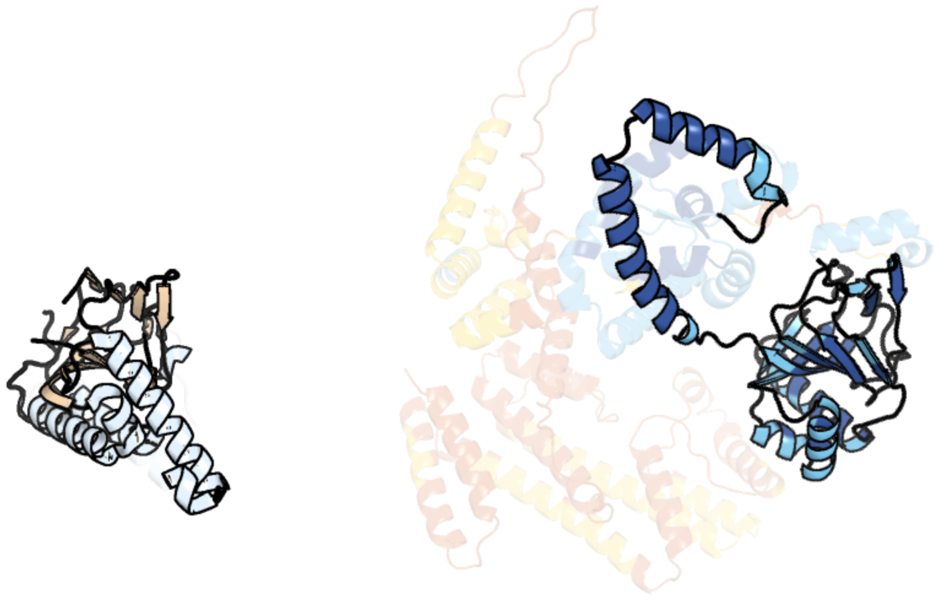

Figure 280: left: reference structure of 3dkq chain B. right: predicted structure of chlorv-1..280, unaligned sequences are shown as transparent

chlorv-1..281

- Sequence-based annotation for chlorv-1..281 is hypothetical protein
- No significant structural hit found

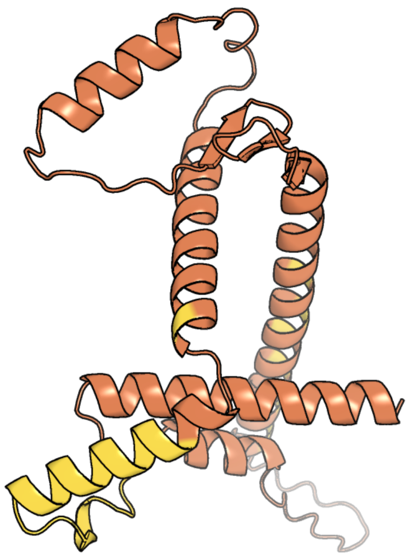

Figure 281: predicted structure of chlorv-1..281

chlorv-1..282

- Sequence-based annotation for chlorv-1..282 is putative HNH endonuclease
- No significant structural hit found

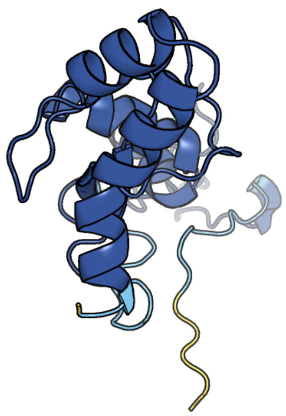

Figure 282: predicted structure of chlorv-1..282

chlorv-1..283

- Sequence-based annotation for chlorv-1..283 is hypothetical protein
- Best hit was 6hnq chain Q: Probable ss-1,3-N-acetylglucosaminyltransferase

target	prob	fident	alnlen	evalue	theader
6hnq-assembly4.cif.gz_Q	1	0.158	366	3.115e-11	TarP-6RboP-(CH2)6NH2
6h4m-assembly2.cif.gz_I	1	0.168	367	3.52e-11	TarP-UDP-GlcNAc-3RboP
6hnq-assembly2.cif.gz_H	1	0.164	370	4.23e-11	TarP-6RboP-(CH2)6NH2

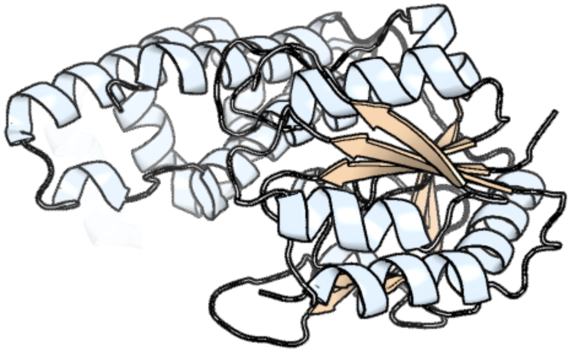

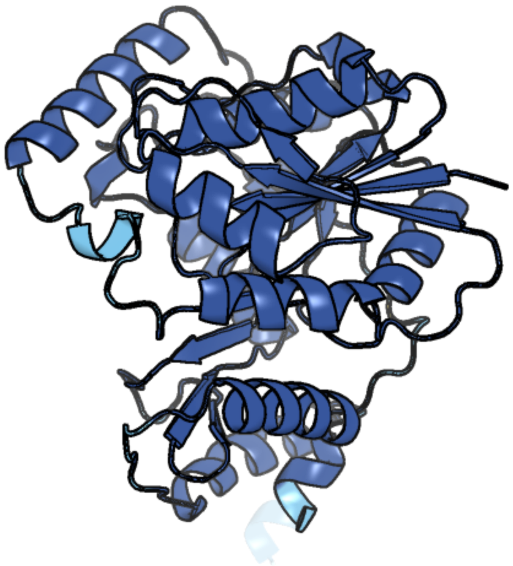

Figure 283: left: reference structure of 6hnq chain Q. right: predicted structure of chlorv-1..283, unaligned sequences are shown as transparent

chlorv-1..284

- Sequence-based annotation for chlorv-1..284 is hypothetical protein
- Best hit was 7opk chain A: 5'-3' exoribonuclease

target	prob	fident	alnlen	evalue	theader
7opk-assembly1.cif.gz_A	1	0.135	511	2.306e-08	Crystal structure of C. thermophilum Xrn2
6q8y-assembly1.cif.gz_z	1	0.132	722	2.64e-08	Cryo-EM structure of the mRNA translating and degrading yeast 80S ribosome-Xrn1 nuclease complex
3pif-assembly2.cif.gz_B	1	0.131	752	6.216e-08	Crystal structure of the 5'->3' exoribonuclease Xrn1, E178Q mutant in Complex with Manganese

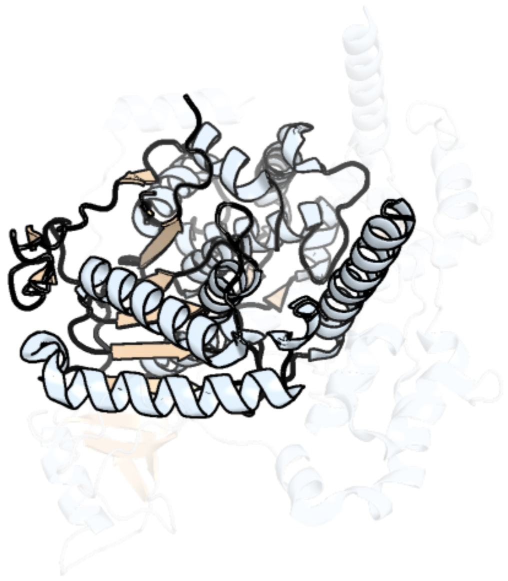

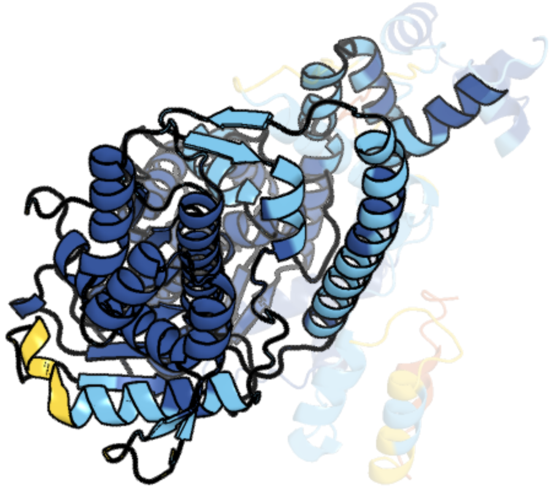

Figure 284: left: reference structure of 7opk chain A. right: predicted structure of chlorv-1..284, unaligned sequences are shown as transparent

chlorv-1..285

- Sequence-based annotation for chlorv-1..285 is hypothetical protein
- No significant structural hit found

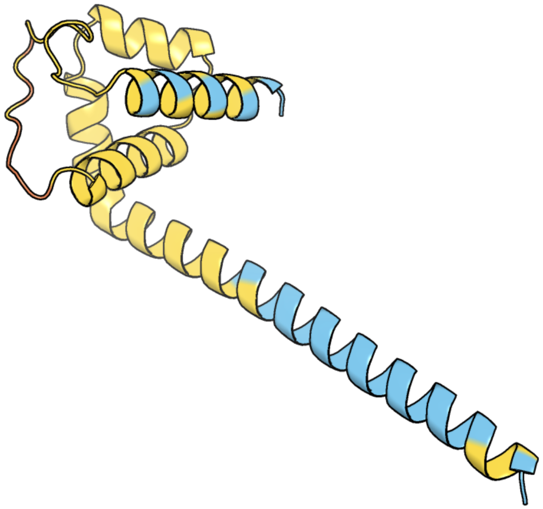

Figure 285: predicted structure of chlorv-1..285

chlorv-1..286

- Sequence-based annotation for chlorv-1..286 is hypothetical protein
- No significant structural hit found

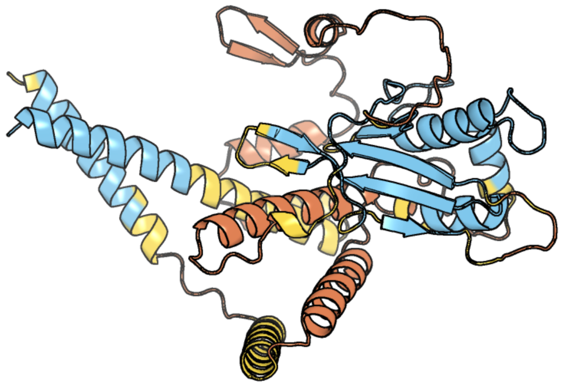

Figure 286: predicted structure of chlorv-1..286

chlorv-1..287

- Sequence-based annotation for chlorv-1..287 is hypothetical protein
- Best hit was 5mog chain C: Phytoene dehydrogenase, chloroplastic/chromoplastic

target	prob	fident	alnlen	evalue	theader
5mog-assembly1.cif.gz_C	1	0.124	491	2.704e-19	Oryza sativa phytoene desaturase inhibited by norflurazon
3i6d-assembly1.cif.gz_A	1	0.141	465	3.734e-16	Crystal structure of PPO from bacillus subtilis with AF
3i6d-assembly2.cif.gz_B	1	0.139	465	5.635e-16	Crystal structure of PPO from bacillus subtilis with AF

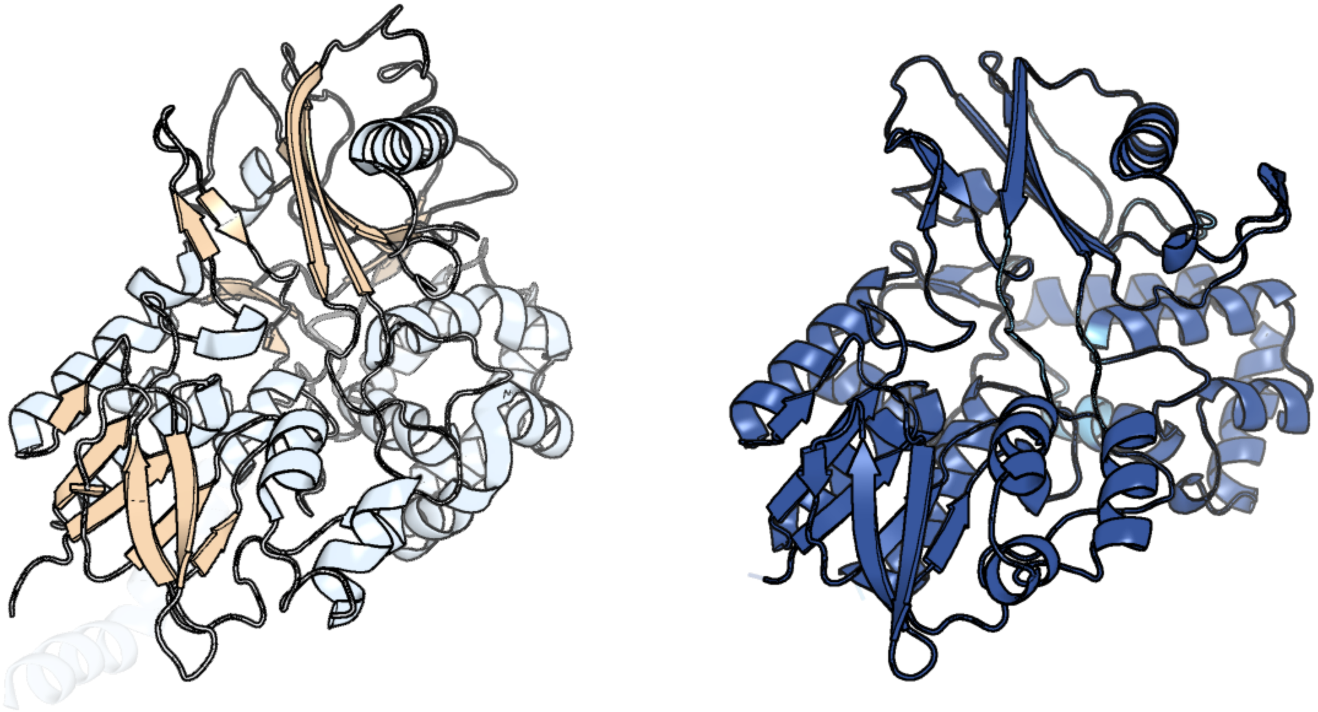

Figure 287: left: reference structure of 5mog chain C. right: predicted structure of chlorv-1..287, unaligned sequences are shown as transparent

chlorv-1..288

- Sequence-based annotation for chlorv-1..288 is putative Phosphatase
- No significant structural hit found

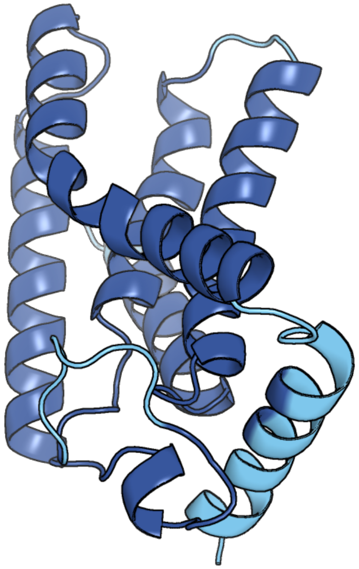

Figure 288: predicted structure of chlorv-1..288

chlorv-1..289

- Sequence-based annotation for chlorv-1..289 is putative Helicase
- Best hit was 7amv chain W: ATP-dependent helicase VETFS

target	prob	fident	alnlen	evalue	theader
7amv-assembly1.cif.gz_W	1	0.189	592	4.272e-22	Atomic structure of the poxvirus transcription pre-initiation complex in the initially melted state
7aoh-assembly1.cif.gz_Y	1	0.197	581	1.281e-21	Atomic structure of the poxvirus late initially transcribing complex
6rfl-assembly1.cif.gz_Y	1	0.202	578	9.758e-21	Structure of the complete Vaccinia DNA-dependent RNA polymerase complex

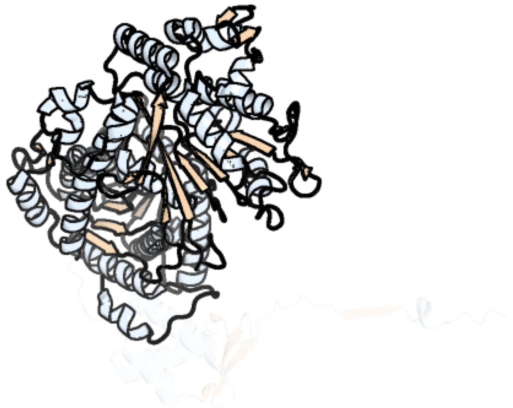

Figure 289: left: reference structure of 7amv chain W. right: predicted structure of chlorv-1..289, unaligned sequences are shown as transparent

chlorv-1..290

- Sequence-based annotation for chlorv-1..290 is hypothetical protein
- Best hit was 5mqp chain F: Glycoside hydrolase BT_1002

target	prob	fident	alnlen	evalue	theader
5mqp-assembly6.cif.gz_F	1	0.11	753	3.281e-17	Glycoside hydrolase BT_1002
3vsv-assembly1.cif.gz_B	1	0.095	772	3.631e-08	The complex structure of XylC with xylose
4ru5-assembly1.cif.gz_B	1	0.094	686	2.673e-07	Crystal Structure of the Pseudomonas phage phi297 tailspike gp61

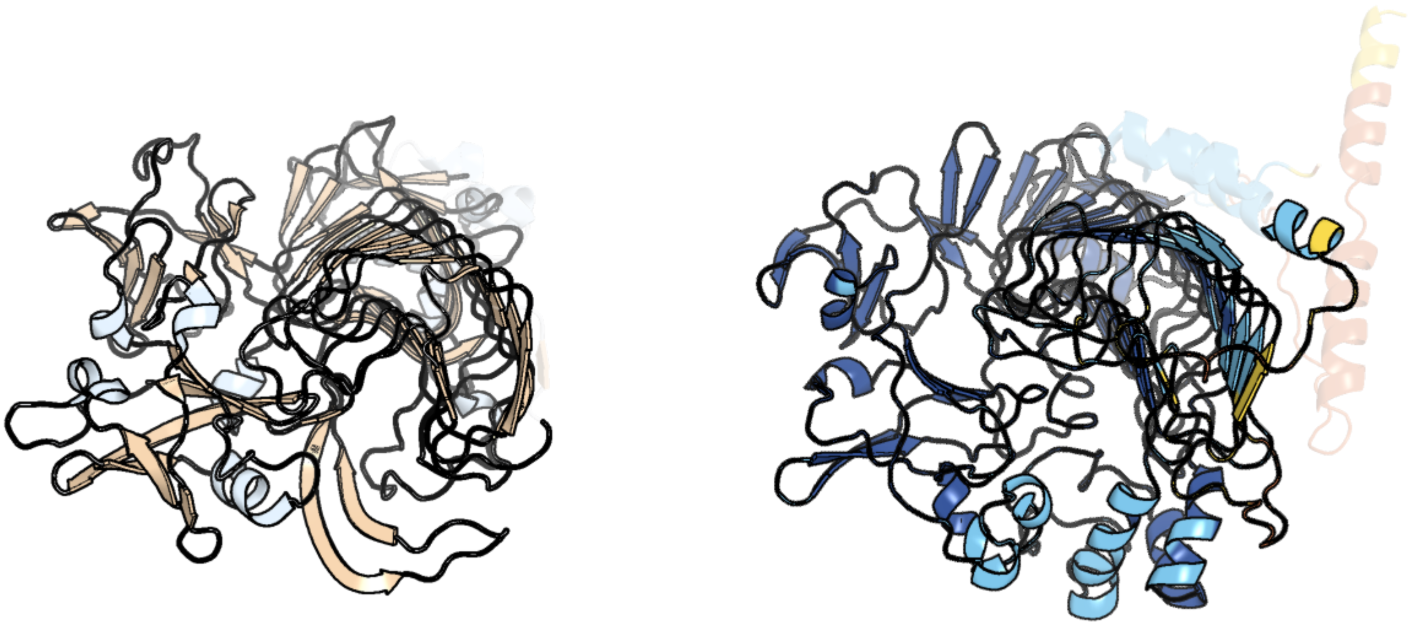

Figure 290: left: reference structure of 5mqp chain F. right: predicted structure of chlorv-1..290, unaligned sequences are shown as transparent

chlorv-1..291

- Sequence-based annotation for chlorv-1..291 is hypothetical protein
- No significant structural hit found

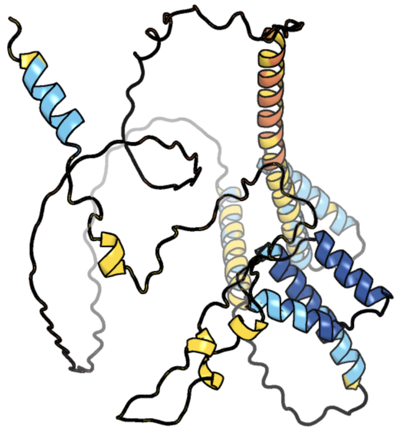

Figure 291: predicted structure of chlorv-1..291

chlorv-1..292

- Sequence-based annotation for chlorv-1..292 is putative DnaJ domain
- Best hit was 8j07 chain u: DnaJ homolog subfamily B member 13

target	prob	fident	alnlen	evalue	theader
8j07-assembly1.cif.gz_u	1	0.149	361	3.479e-12	96nm repeat of human respiratory doublet microtubule and associated axonemal complexes
8j07-assembly1.cif.gz_v	1	0.144	361	1.164e-11	96nm repeat of human respiratory doublet microtubule and associated axonemal complexes
4j80-assembly2.cif.gz_D	1	0.117	349	2.858e-10	Thermus thermophilus DnaJ

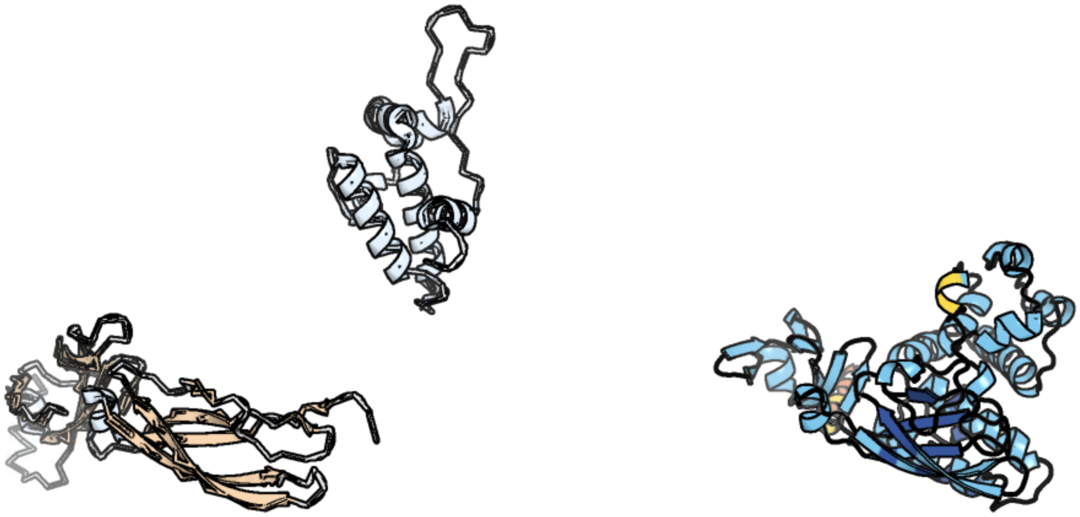

Figure 292: left: reference structure of 8j07 chain u. right: predicted structure of chlorv-1..292, unaligned sequences are shown as transparent

chlorv-1..293

- Sequence-based annotation for chlorv-1..293 is hypothetical protein
- No significant structural hit found

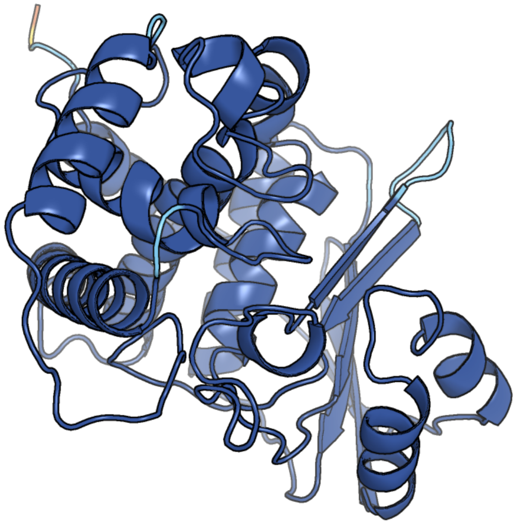

Figure 293: predicted structure of chlorv-1..293

chlorv-1..294

- Sequence-based annotation for chlorv-1..294 is hypothetical protein
- No significant structural hit found

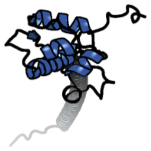

Figure 294: predicted structure of chlorv-1..294

chlorv-1..295

- Sequence-based annotation for chlorv-1..295 is hypothetical protein
- No significant structural hit found

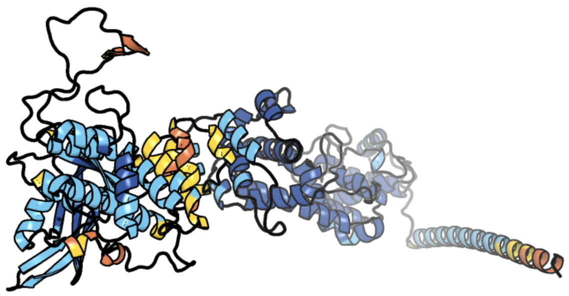

Figure 295: predicted structure of chlorv-1..295

chlorv-1..296

- Sequence-based annotation for chlorv-1..296 is hypothetical protein
- No significant structural hit found

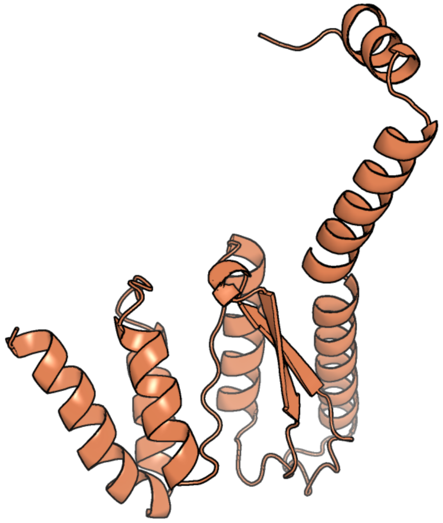

Figure 296: predicted structure of chlorv-1..296

chlorv-1..297

- Sequence-based annotation for chlorv-1..297 is hypothetical protein
- No significant structural hit found

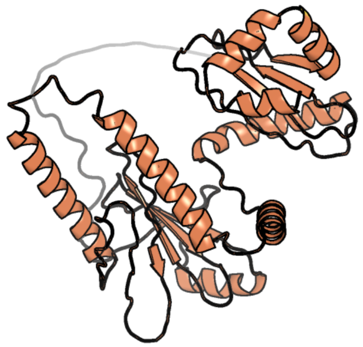

Figure 297: predicted structure of chlorv-1..297

chlorv-1..298

- Sequence-based annotation for chlorv-1..298 is hypothetical protein
- No significant structural hit found

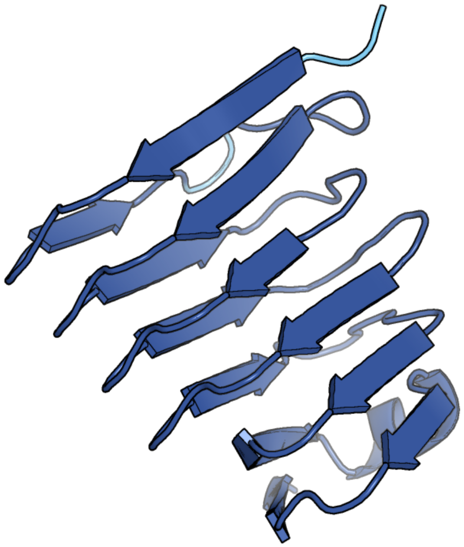

Figure 298: predicted structure of chlorv-1..298

chlorv-1..299

- Sequence-based annotation for chlorv-1..299 is hypothetical protein
- Best hit was 6irw chain A: Phosphorylated CTD-interacting factor 1

target	prob	fident	alnlen	evalue	theader
6irw-assembly1.cif.gz_A	1	0.141	495	2.928e-14	Crystal structure of the human cap-specific adenosine methyltransferase bound to SAH
6iry-assembly1.cif.gz_A	1	0.153	496	2.928e-14	Crystal structure of the zebrafish cap-specific adenosine methyltransferase bound to SAH
6irx-assembly1.cif.gz_A	1	0.145	496	3.094e-14	Crystal structure of the zebrafish cap-specific adenosine methyltransferase

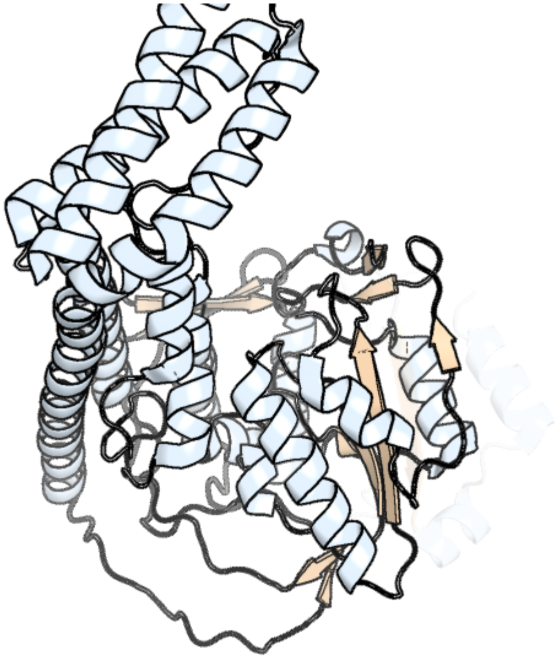

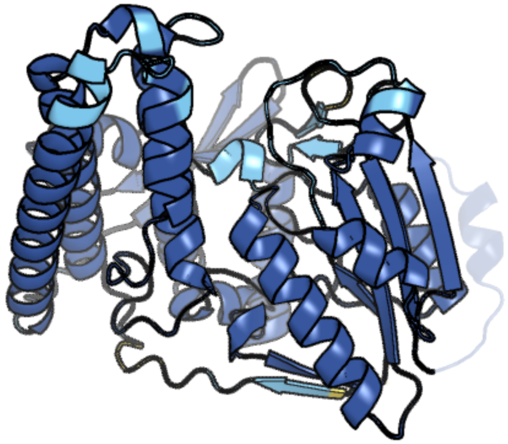

Figure 299: left: reference structure of 6irw chain A. right: predicted structure of chlorv-1..299, unaligned sequences are shown as transparent

chlorv-1..300

- Sequence-based annotation for chlorv-1..300 is putative Ubiquitin-conjugating enzyme
- Best hit was 5knl chain C: Ubiquitin-conjugating enzyme E2 15

target	prob	fident	alnlen	evalue	theader
5knl-assembly1.cif.gz_C	1	0.375	165	5.562e-18	Crystal structure of S. pombe ubiquitin E1 (Uba1) in complex with Ubc15 and ubiquitin
5knl-assembly2.cif.gz_F	1	0.353	164	1.306e-17	Crystal structure of S. pombe ubiquitin E1 (Uba1) in complex with Ubc15 and ubiquitin
3fsh-assembly2.cif.gz_B	1	0.408	169	1.568e-17	Crystal structure of the ubiquitin conjugating enzyme Ube2g2 bound to the G2BR domain of ubiquitin ligase gp78

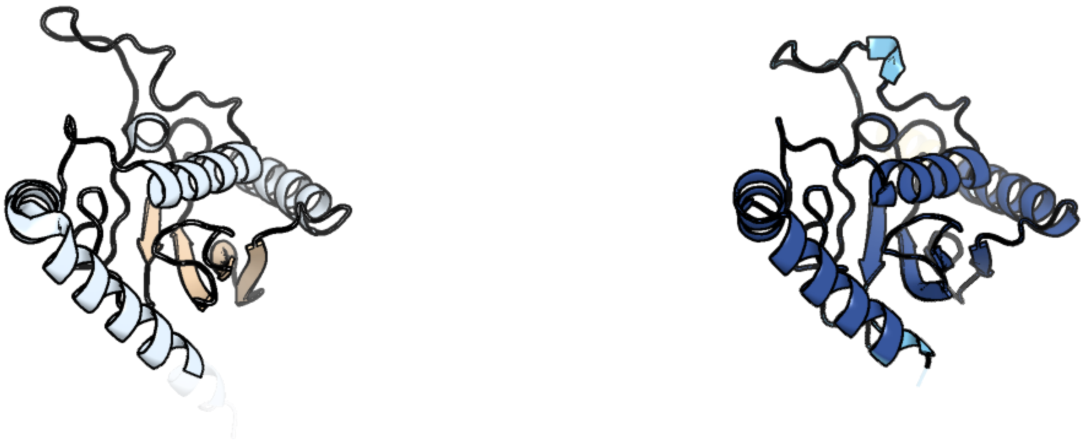

Figure 300: left: reference structure of 5knl chain C. right: predicted structure of chlorv-1..300, unaligned sequences are shown as transparent

chlorv-1..301

- Sequence-based annotation for chlorv-1..301 is hypothetical protein
- No significant structural hit found

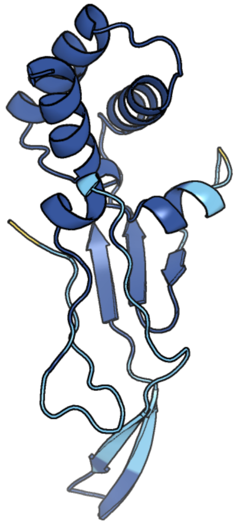

Figure 301: predicted structure of chlorv-1..301

chlorv-1..302

- Sequence-based annotation for chlorv-1..302 is putative Aidotransferase
- Best hit was 4zfl chain A: Amidohydrolase EgtC

target	prob	fident	alnlen	evalue	theader
4zfl-assembly1.cif.gz_A	1	0.156	255	1.277e-13	Ergothioneine-biosynthetic Ntn hydrolase variant EgtC_C2A with natural substrate
4zfk-assembly1.cif.gz_A	1	0.16	256	3.191e-13	Ergothioneine-biosynthetic Ntn hydrolase EgtC with glutamine
4zfj-assembly1.cif.gz_B	1	0.149	254	1.993e-12	Ergothioneine-biosynthetic Ntn hydrolase EgtC, apo form

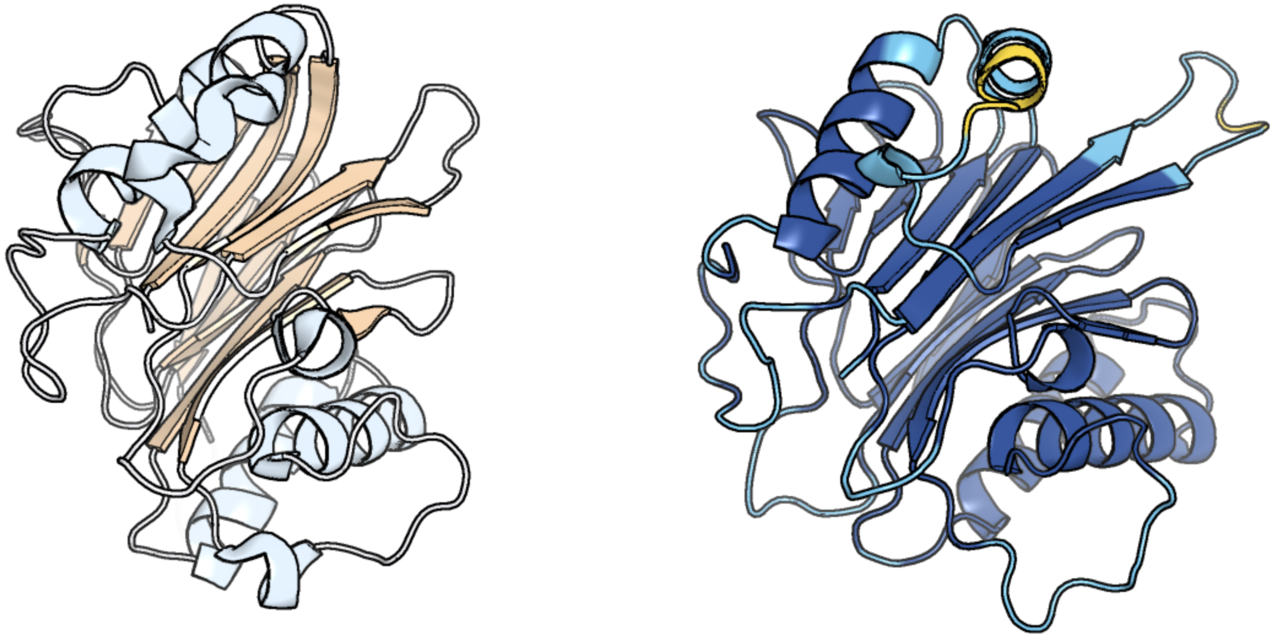

Figure 302: left: reference structure of 4zfl chain A. right: predicted structure of chlorv-1..302, unaligned sequences are shown as transparent

chlorv-1..303

- Sequence-based annotation for chlorv-1..303 is hypothetical protein
- No significant structural hit found

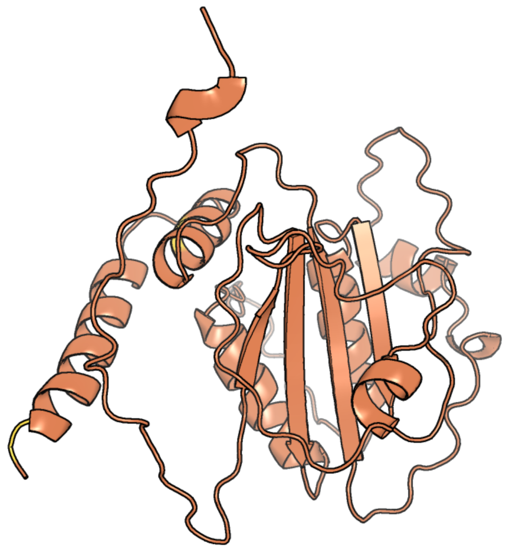

Figure 303: predicted structure of chlorv-1..303

chlorv-1..304

- Sequence-based annotation for chlorv-1..304 is hypothetical protein
- No significant structural hit found

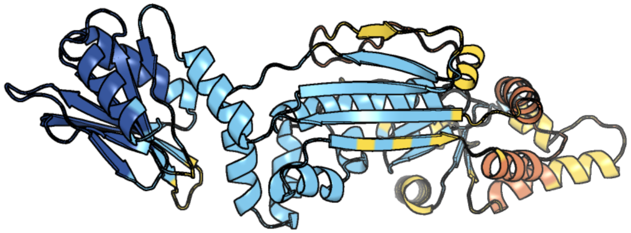

Figure 304: predicted structure of chlorv-1..304

chlorv-1..305

- Sequence-based annotation for chlorv-1..305 is hypothetical protein
- Best hit was 7x87 chain B: Beta-galactosidase

target	prob	fident	alnlen	evalue	theader
7x87-assembly1.cif.gz_B	0.994	0.112	293	3.388e-05	The complex structure of beta-1,2-glucosyltransferase from Ignavibacterium album with sophotetraose observed as sophorose
7vkx-assembly1.cif.gz_A	0.992	0.111	296	3.817e-05	The complex structure of beta-1,2-glucosyltransferase from Ignavibacterium album with glucose
7x87-assembly1.cif.gz_A	0.992	0.116	293	4.844e-05	The complex structure of beta-1,2-glucosyltransferase from Ignavibacterium album with sophotetraose observed as sophorose

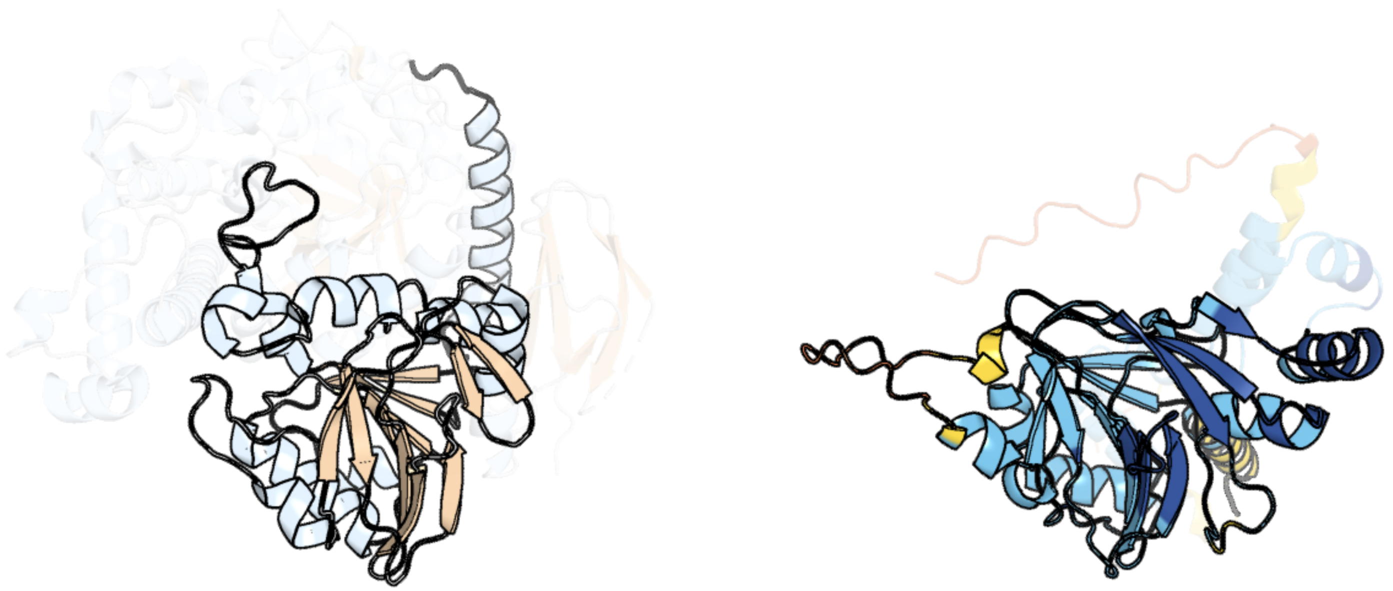

Figure 305: left: reference structure of 7x87 chain B. right: predicted structure of chlorv-1..305, unaligned sequences are shown as transparent

chlorv-1..306

- Sequence-based annotation for chlorv-1..306 is hypothetical protein
- No significant structural hit found

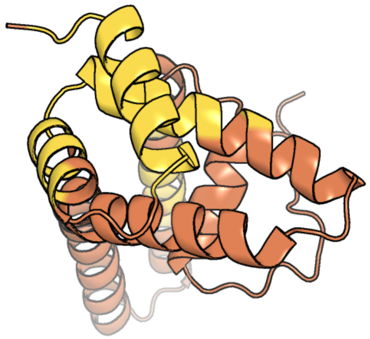

Figure 306: predicted structure of chlorv-1..306

chlorv-1..307

- Sequence-based annotation for chlorv-1..307 is hypothetical protein
- Best hit was 2iuw chain A: ALKYLATED REPAIR PROTEIN ALKB HOMOLOG 3

target	prob	fident	alnlen	evalue	theader
2iuw-assembly1.cif.gz_A-2	1	0.193	212	5.46e-09	Crystal structure of human ABH3 in complex with iron ion and 2-oxoglutarate
3rzg-assembly1.cif.gz_A	1	0.183	185	8.343e-09	Duplex Interrogation by a Direct DNA Repair Protein in the Search of Damage
3btz-assembly1.cif.gz_A	1	0.176	204	1.354e-08	Crystal structure of human ABH2 cross-linked to dsDNA

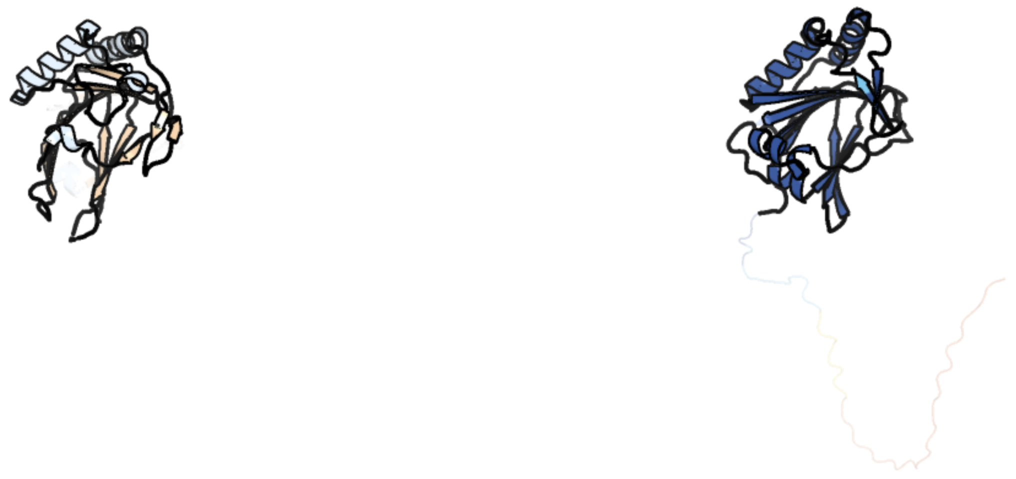

Figure 307: left: reference structure of 2iuw chain A. right: predicted structure of chlorv-1..307, unaligned sequences are shown as transparent

chlorv-1..308

- Sequence-based annotation for chlorv-1..308 is putative Peptidase
- Best hit was 4boz chain A: UBIQUITIN THIOESTERASE OTU1

target	prob	fident	alnlen	evalue	theader
4boz-assembly1.cif.gz_A	1	0.304	161	1.517e-15	Structure of OTUD2 OTU domain in complex with K11-linked di ubiquitin
4bos-assembly1.cif.gz_B	1	0.302	162	5.371e-15	Structure of OTUD2 OTU domain in complex with Ubiquitin K11-linked peptide
4boq-assembly1.cif.gz_A-2	1	0.325	163	7.259e-15	Structure of OTUD2 OTU domain

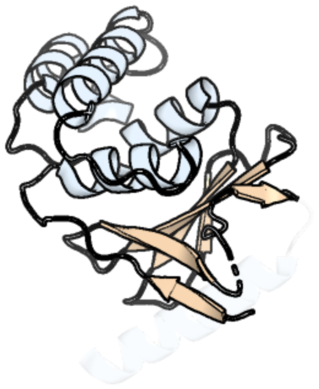

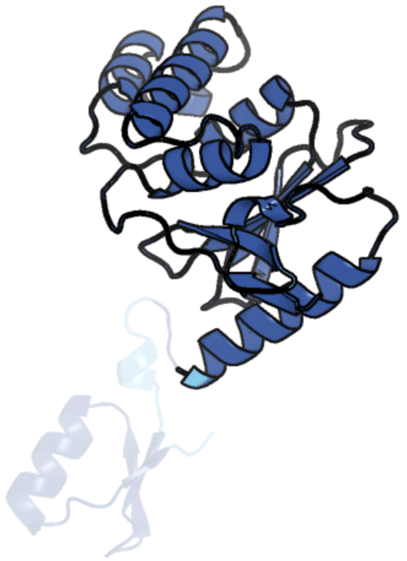

Figure 308: left: reference structure of 4boz chain A. right: predicted structure of chlorv-1..308, unaligned sequences are shown as transparent

chlorv-1..309

- Sequence-based annotation for chlorv-1..309 is hypothetical protein
- No significant structural hit found

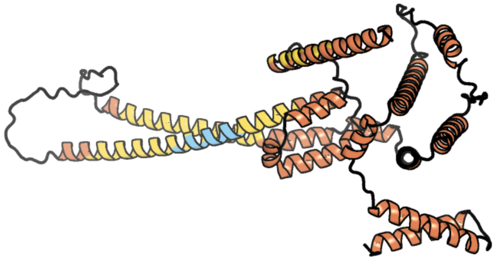

Figure 309: predicted structure of chlorv-1..309

chlorv-1..310

- Sequence-based annotation for chlorv-1..310 is hypothetical protein
- No significant structural hit found

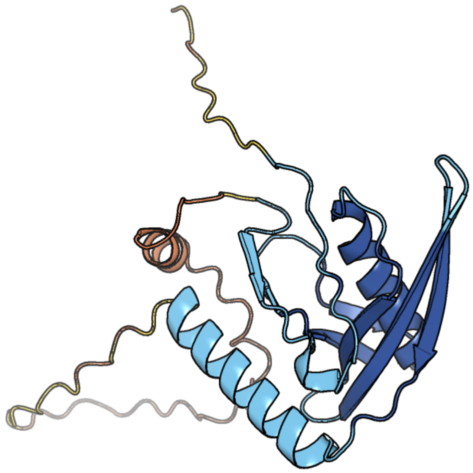

Figure 310: predicted structure of chlorv-1..310

chlorv-1..311

- Sequence-based annotation for chlorv-1..311 is putative mRNA capping enzyme
- Best hit was 4ckb chain D: MRNA-CAPPING ENZYME CATALYTIC SUBUNIT

target	prob	fident	alnlen	evalue	theader
4ckb-assembly1.cif.gz_D	1	0.163	1047	2.578e-40	Vaccinia virus capping enzyme complexed with GTP and SAH
6rfl-assembly1.cif.gz_O	1	0.17	1041	3.011e-40	Structure of the complete Vaccinia DNA-dependent RNA polymerase complex
4ckc-assembly2.cif.gz_D	1	0.17	1046	4.105e-40	Vaccinia virus capping enzyme complexed with SAH (monoclinic form)

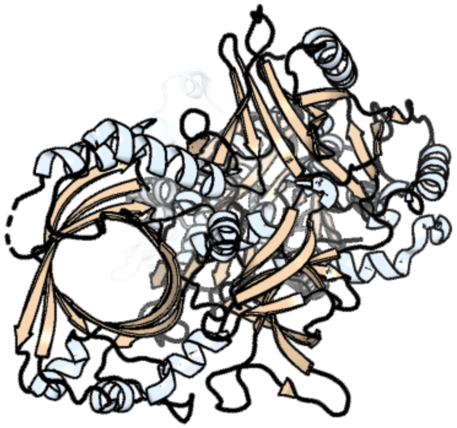

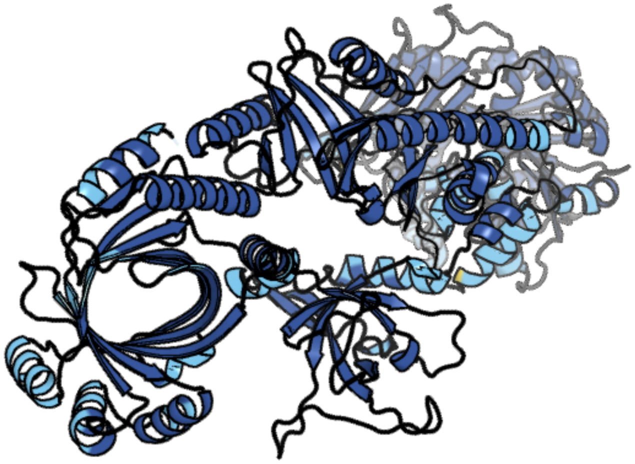

Figure 311: left: reference structure of 4ckb chain D. right: predicted structure of chlorv-1..311, unaligned sequences are shown as transparent

chlorv-1..312

- Sequence-based annotation for chlorv-1..312 is putative Poly(A) polymerase catalytic subunit
- Best hit was 4wse chain B: Putative poly(A) polymerase catalytic subunit

target	prob	fident	alnlen	evalue	theader
4wse-assembly1.cif.gz_B	1	0.267	464	2.245e-37	Crystal structure of the Mimivirus polyadenylate synthase
4wse-assembly1.cif.gz_A	1	0.263	467	3.645e-37	Crystal structure of the Mimivirus polyadenylate synthase
4p37-assembly1.cif.gz_A	1	0.256	467	1.014e-36	Crystal structure of the Megavirus polyadenylate synthase

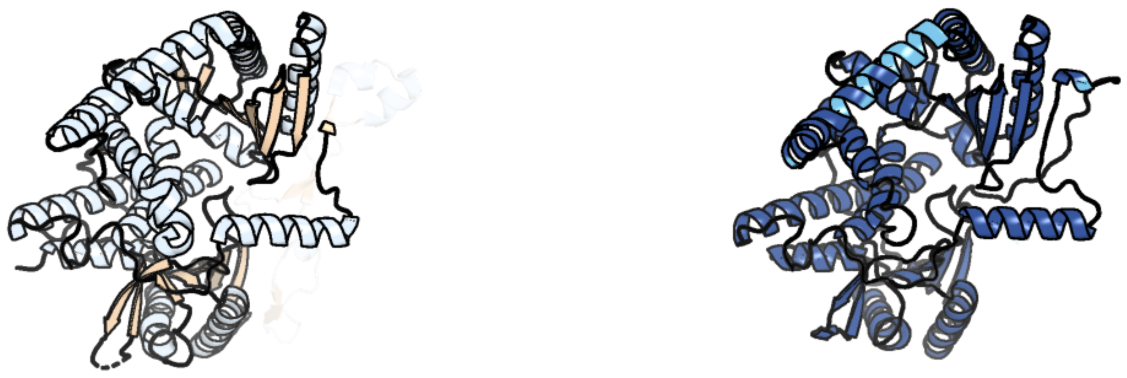

Figure 312: left: reference structure of 4wse chain B. right: predicted structure of chlorv-1..312, unaligned sequences are shown as transparent

chlorv-1..313

- Sequence-based annotation for chlorv-1..313 is hypothetical protein
- No significant structural hit found

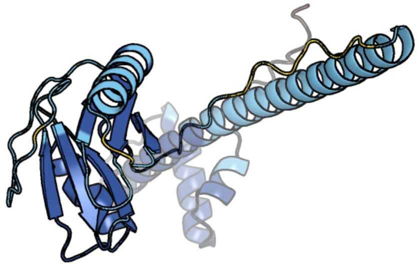

Figure 313: predicted structure of chlorv-1..313

chlorv-1..314

- Sequence-based annotation for chlorv-1..314 is putative Endonuclease/Exonuclease/phosphatase
- Best hit was 8igi chain B: Exodeoxyribonuclease (LexA)

target	prob	fident	alnlen	evalue	theader
8igi-assembly2.cif.gz_B	1	0.467	261	1.679e-34	Crystal structure of HP1526 (XthA)- a base excision DNA repair protein in Helicobacter pylori
6bov-assembly1.cif.gz_A	1	0.401	259	2.607e-34	Human APE1 substrate complex with an A/G mismatch adjacent the THF
2o3c-assembly1.cif.gz_C	1	0.406	263	4.047e-34	Crystal structure of zebrafish Ape

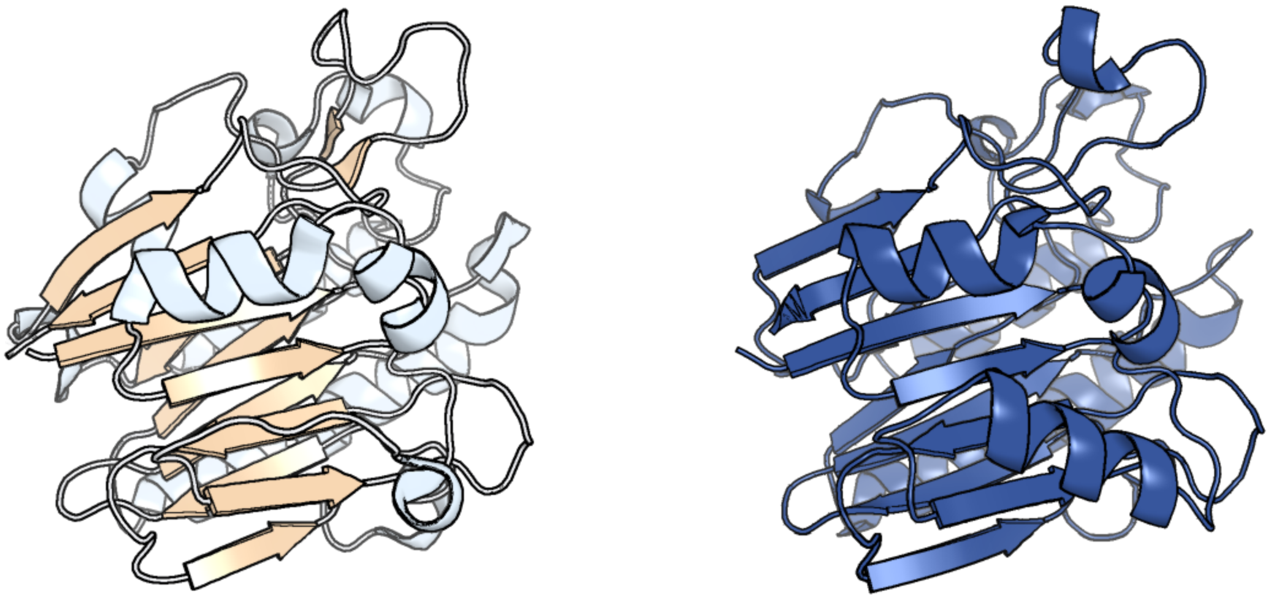

Figure 314: left: reference structure of 8igi chain B. right: predicted structure of chlorv-1..314, unaligned sequences are shown as transparent

chlorv-1..315

- Sequence-based annotation for chlorv-1..315 is hypothetical protein
- No significant structural hit found

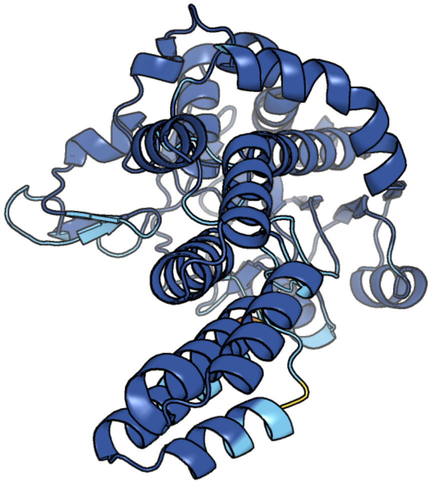

Figure 315: predicted structure of chlorv-1..315

chlorv-1..316

- Sequence-based annotation for chlorv-1..316 is putative Threonine synthase
- Best hit was 1vb3 chain A: Threonine synthase

target	prob	fident	alnlen	evalue	theader
1vb3-assembly1.cif.gz_A	1	0.337	429	1.819e-37	Crystal Structure of Threonine Synthase from Escherichia coli
4f4f-assembly2.cif.gz_B	1	0.297	461	5.304e-34	X-Ray crystal structure of PLP bound Threonine synthase from Brucella melitensis
1kl7-assembly2.cif.gz_B	1	0.293	501	3.346e-30	Crystal Structure of Threonine Synthase from Yeast

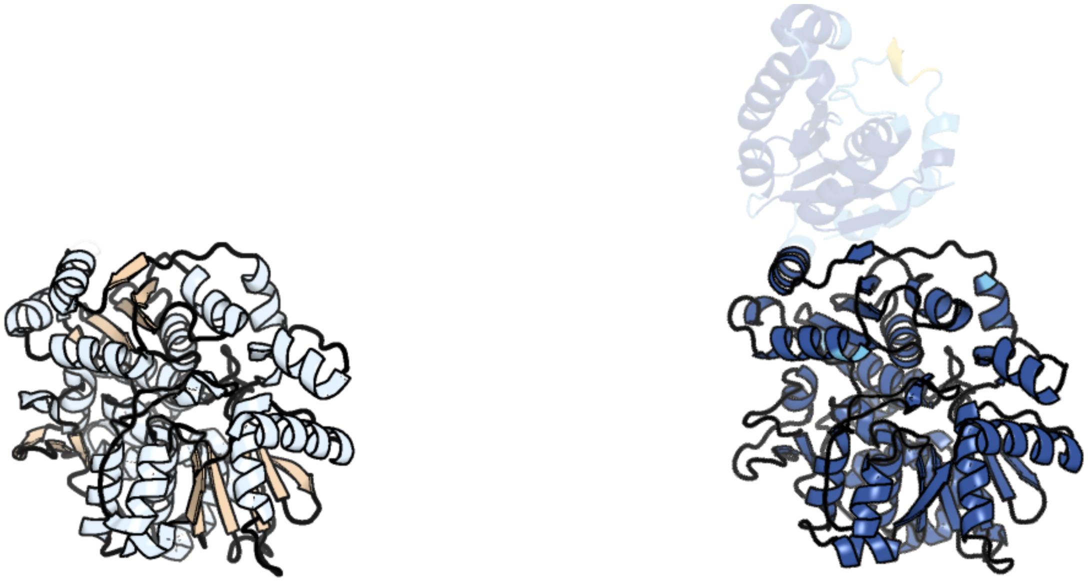

Figure 316: left: reference structure of 1vb3 chain A. right: predicted structure of chlorv-1..316, unaligned sequences are shown as transparent

chlorv-1..317

- Sequence-based annotation for chlorv-1..317 is putative Helicase
- Best hit was 6rfl chain Y: Nucleoside triphosphate phosphohydrolase-I

target	prob	fident	alnlen	evalue	theader
6rfl-assembly1.cif.gz_Y	1	0.183	773	8.165e-31	Structure of the complete Vaccinia DNA-dependent RNA polymerase complex
7aoh-assembly1.cif.gz_Y	1	0.188	778	9.101e-31	Atomic structure of the poxvirus late initially transcribing complex
7tn2-assembly1.cif.gz_W	1	0.145	680	4.811e-16	Composite model of a Chd1-nucleosome complex in the nucleotide-free state derived from 2.3A and 2.7A Cryo-EM maps

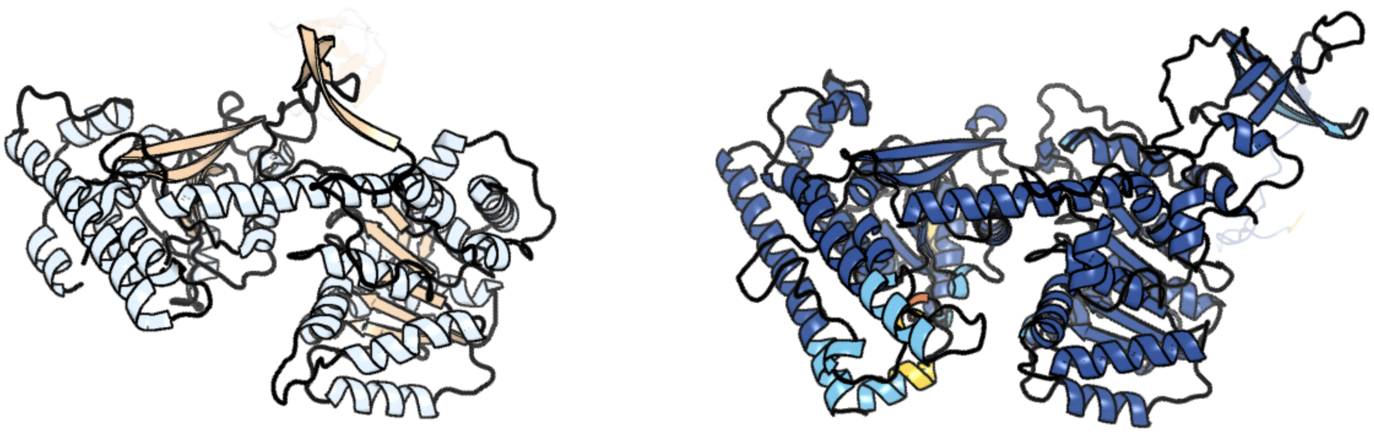

Figure 317: left: reference structure of 6rfl chain Y. right: predicted structure of chlorv-1..317, unaligned sequences are shown as transparent

chlorv-1..318

- Sequence-based annotation for chlorv-1..318 is putative Exonuclease/DNA polymerase III subunit epsilon
- Best hit was 8h2f chain A: DnaQ

target	prob	fident	alnlen	evalue	theader
8h2f-assembly1.cif.gz_A	1	0.184	206	1.804e-09	Crystal structure of DnaQ domain in complex witn TMP of Streptococcus thermophilus strain DGCC 7710
4js5-assembly2.cif.gz_B	1	0.196	204	4.581e-08	Crystal structure of E. coli Exonuclease I in complex with a dT13 oligonucleotide
6a4b-assembly1.cif.gz_B	1	0.149	228	9.4e-08	Structure of TREX2 in complex with a duplex DNA with 2 nucleotide 3'-overhang

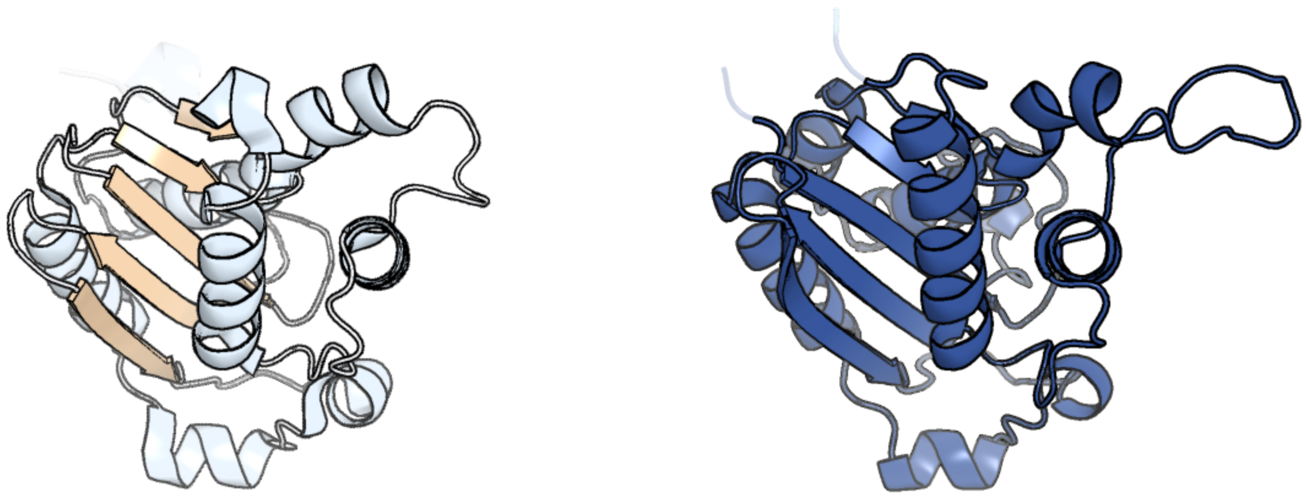

Figure 318: left: reference structure of 8h2f chain A. right: predicted structure of chlorv-1..318, unaligned sequences are shown as transparent

chlorv-1..319

- Sequence-based annotation for chlorv-1..319 is hypothetical protein
- No significant structural hit found

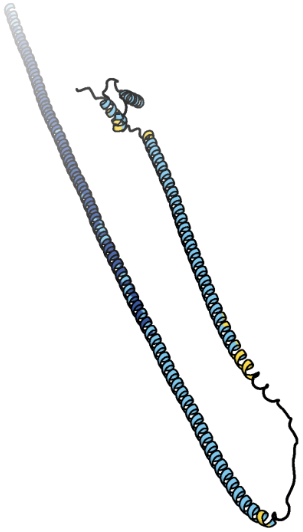

Figure 319: predicted structure of chlorv-1..319

chlorv-1..320

- Sequence-based annotation for chlorv-1..320 is putative Helicase
- Best hit was 2is6 chain B: DNA helicase II

target	prob	fident	alnlen	evalue	theader
2is6-assembly1.cif.gz_B	1	0.197	639	1.594e-30	Crystal structure of UvrD-DNA-ADPMgF3 ternary complex
2is4-assembly1.cif.gz_B	1	0.196	625	3.321e-30	Crystal structure of UvrD-DNA-ADPNP ternary complex
2is4-assembly1.cif.gz_A	1	0.184	638	1.232e-29	Crystal structure of UvrD-DNA-ADPNP ternary complex

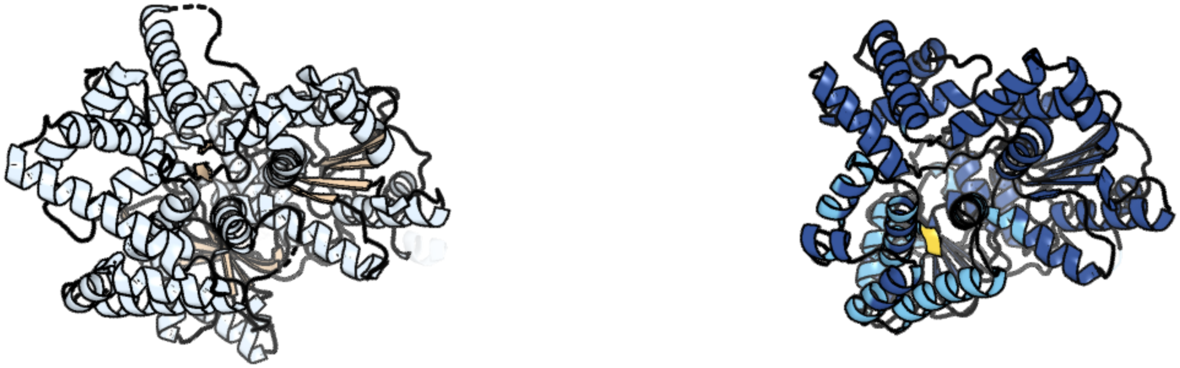

Figure 320: left: reference structure of 2is6 chain B. right: predicted structure of chlorv-1..320, unaligned sequences are shown as transparent

chlorv-1..321

- Sequence-based annotation for chlorv-1..321 is hypothetical protein
- No significant structural hit found

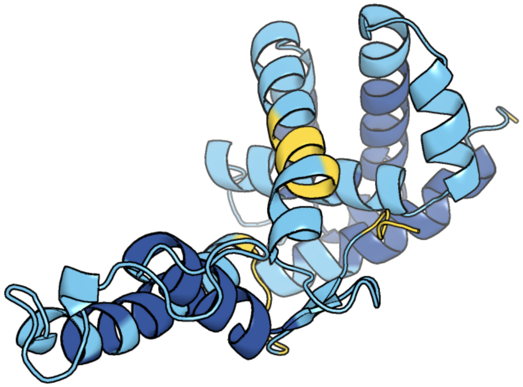

Figure 321: predicted structure of chlorv-1..321

chlorv-1..322

- Sequence-based annotation for chlorv-1..322 is hypothetical protein
- No significant structural hit found

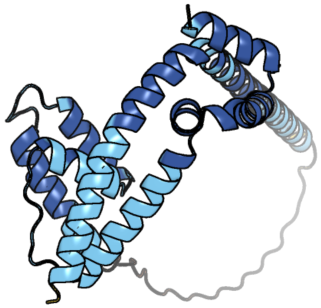

Figure 322: predicted structure of chlorv-1..322

chlorv-1..323

- Sequence-based annotation for chlorv-1..323 is putative DAHP synthetase I/aldolase
- Best hit was 5dcd chain B: Phospho-2-dehydro-3-deoxyheptonate aldolase

target	prob	fident	alnlen	evalue	theader
5dcd-assembly1.cif.gz_B	1	0.468	344	1.708e-43	Neisseria meningitidis 3-deoxy-D-arabino-heptulosonate 7-phosphate synthase regulated (Tyrosine)
8e0y-assembly1.cif.gz_A	1	0.497	340	3.3e-43	DAHP (3-deoxy-D-arabinoheptulosonate-7-phosphate) Synthase complexed with DAHP oxime, Pr(III), and Pi in unbound:(bound)2:other Conformations
1n8f-assembly1.cif.gz_A	1	0.495	343	3.683e-43	Crystal structure of E24Q mutant of phenylalanine-regulated 3-deoxy-D-arabino-heptulosonate-7-phosphate synthase (DAHP synthase) from Escherichia Coli in complex with Mn2+ and PEP

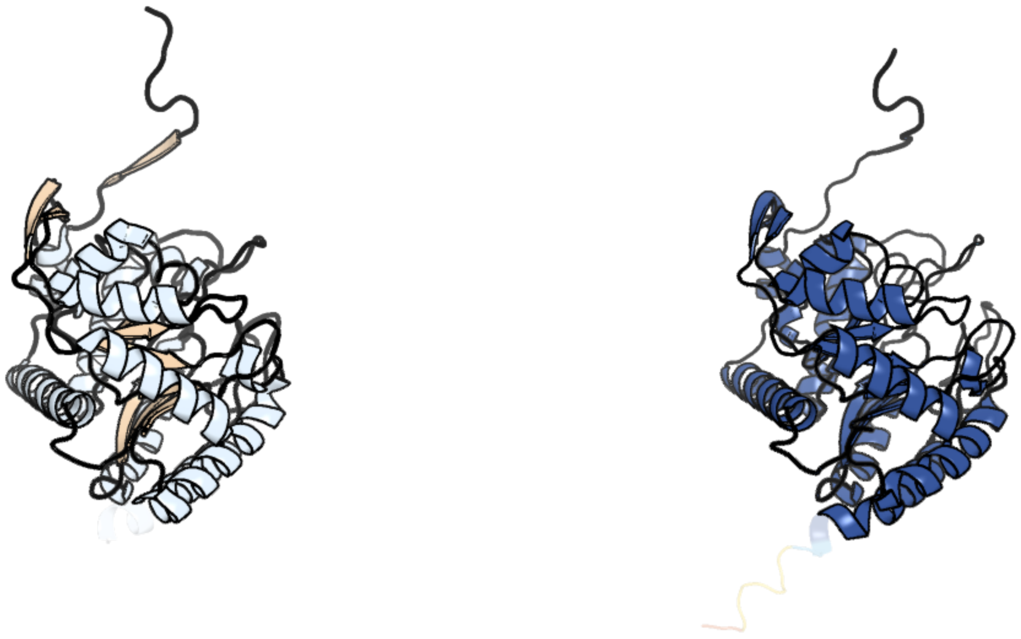

Figure 323: left: reference structure of 5dcd chain B. right: predicted structure of chlorv-1..323, unaligned sequences are shown as transparent

chlorv-1..324

- Sequence-based annotation for chlorv-1..324 is putative Dioxygenase
- Best hit was 6d0o chain C: (R)-phenoxypropionate/alpha-ketoglutarate-dioxygenase

target	prob	fident	alnlen	evalue	theader
6d0o-assembly1.cif.gz_C	1	0.218	266	8.626e-22	rdpA dioxygenase holoenzyme
6d3i-assembly1.cif.gz_J-2	1	0.222	270	1.578e-21	ftv7 dioxygenase with 2,4-D bound
1gy9-assembly1.cif.gz_B	1	0.257	264	2.409e-21	Taurine/alpha-ketoglutarate Dioxygenase from Escherichia coli

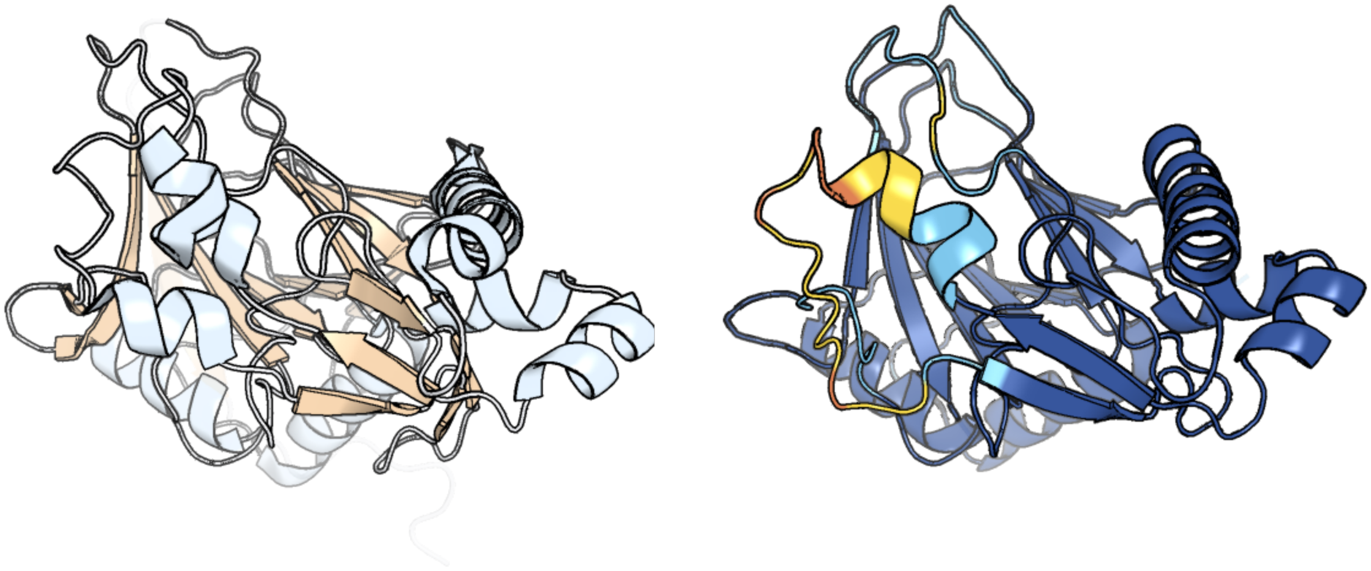

Figure 324: left: reference structure of 6d0o chain C. right: predicted structure of chlorv-1..324, unaligned sequences are shown as transparent

chlorv-1..325

- Sequence-based annotation for chlorv-1..325 is putative RNA polymerase Rpb3/Rpb11
- Best hit was 7ok0 chain D: DNA-directed RNA polymerase subunit D

target	prob	fident	alnlen	evalue	theader
7ok0-assembly1.cif.gz_D	1	0.181	286	6.624e-13	Cryo-EM structure of the Sulfolobus acidocaldarius RNA polymerase at 2.88 A
4v8s-assembly2.cif.gz_BD	1	0.188	286	1.227e-12	Archaeal RNAP-DNA binary complex at 4.32Ang
7oqy-assembly1.cif.gz_D	1	0.169	289	1.451e-12	Cryo-EM structure of the cellular negative regulator TFS4 bound to the archaeal RNA polymerase

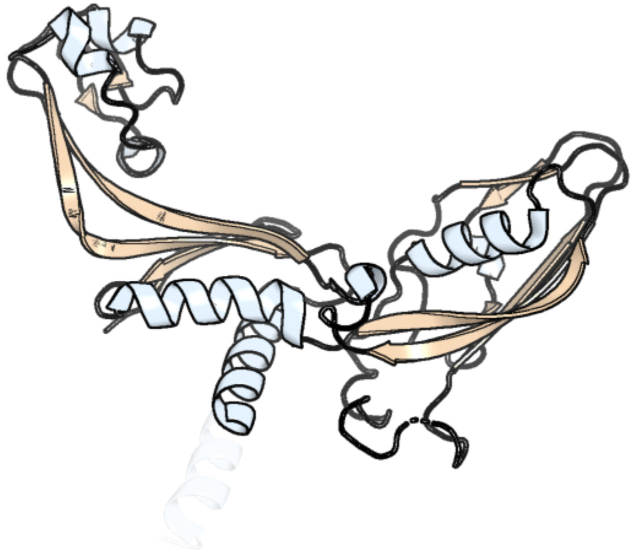

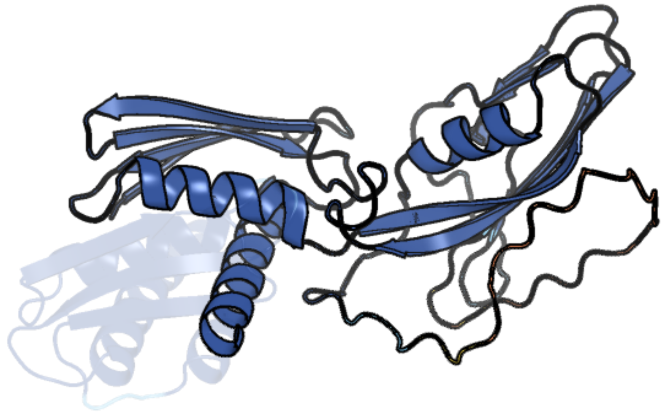

Figure 325: left: reference structure of 7ok0 chain D. right: predicted structure of chlorv-1..325, unaligned sequences are shown as transparent

chlorv-1..326

- Sequence-based annotation for chlorv-1..326 is hypothetical protein
- No significant structural hit found

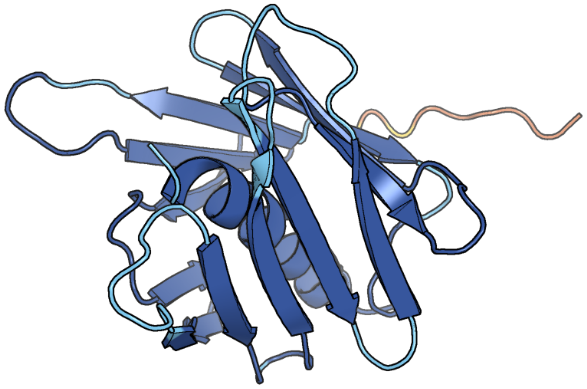

Figure 326: predicted structure of chlorv-1..326

chlorv-1..327

- Sequence-based annotation for chlorv-1..327 is hypothetical protein
- No significant structural hit found

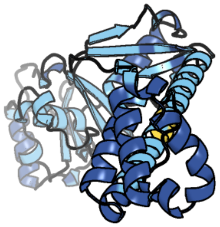

Figure 327: predicted structure of chlorv-1..327

chlorv-1..328

- Sequence-based annotation for chlorv-1..328 is putative GTP binding protein
- Best hit was 1eft chain A: ELONGATION FACTOR TU

target	prob	fident	alnlen	evalue	theader
1eft-assembly1.cif.gz_A	1	0.18	487	2.543e-28	THE CRYSTAL STRUCTURE OF ELONGATION FACTOR EF-TU FROM THERMUS AQUATICUS IN THE GTP CONFORMATION
1exm-assembly1.cif.gz_A	1	0.181	479	7.59e-28	CRYSTAL STRUCTURE OF THERMUS THERMOPHILUS ELONGATION FACTOR TU (EF-TU) IN COMPLEX WITH THE GTP ANALOGUE GPPNHP.
4lbv-assembly1.cif.gz_A	1	0.178	481	2.399e-27	Identifying ligand binding hot spots in proteins using brominated fragments

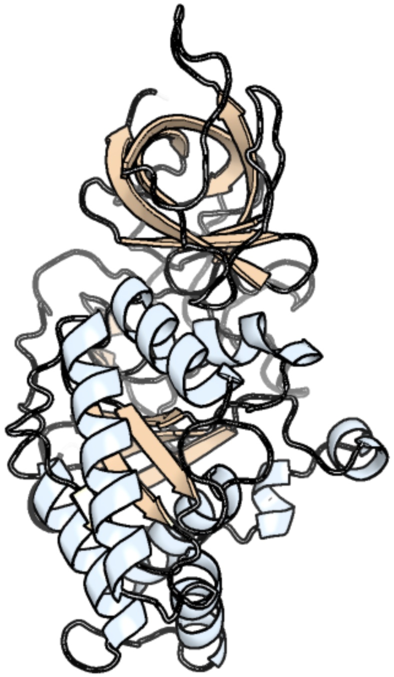

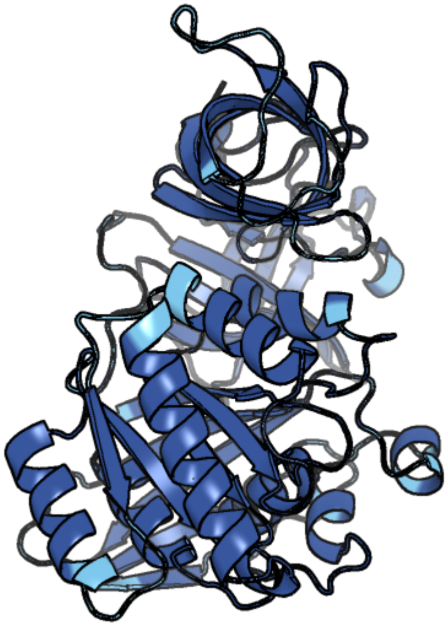

Figure 328: left: reference structure of 1eft chain A. right: predicted structure of chlorv-1..328, unaligned sequences are shown as transparent

chlorv-1..329

- Sequence-based annotation for chlorv-1..329 is putative Ribonuclease H
- Best hit was 2qkk chain M: Ribonuclease H1

target	prob	fident	alnlen	evalue	theader
2qkk-assembly4.cif.gz_M	1	0.331	151	2.446e-11	Human RNase H catalytic domain mutant D210N in complex with 14-mer RNA/DNA hybrid
2qkk-assembly4.cif.gz_N	1	0.302	152	6.185e-11	Human RNase H catalytic domain mutant D210N in complex with 14-mer RNA/DNA hybrid
2qkk-assembly3.cif.gz_J	1	0.324	154	8.426e-11	Human RNase H catalytic domain mutant D210N in complex with 14-mer RNA/DNA hybrid

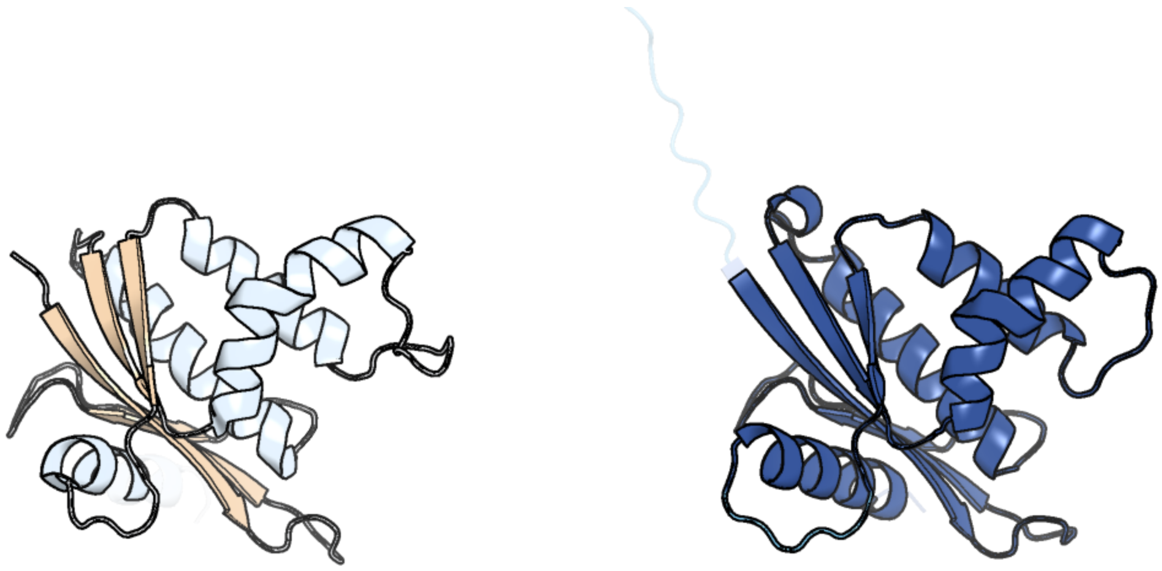

Figure 329: left: reference structure of 2qkk chain M. right: predicted structure of chlorv-1..329, unaligned sequences are shown as transparent

chlorv-1..330

- Sequence-based annotation for chlorv-1..330 is hypothetical protein
- No significant structural hit found

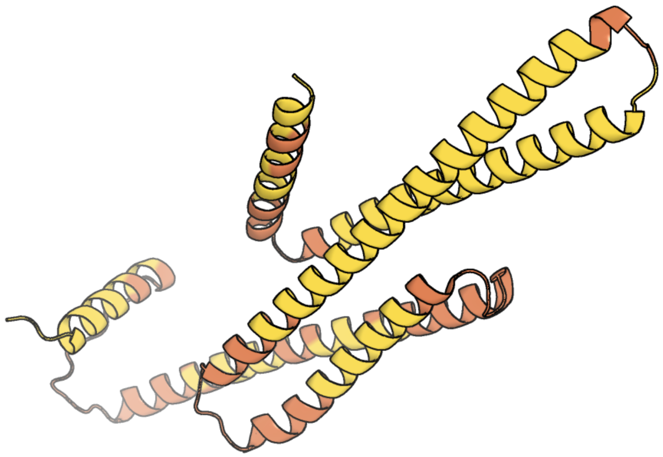

Figure 330: predicted structure of chlorv-1..330

chlorv-1..331

- Sequence-based annotation for chlorv-1..331 is putative replication factor C small subunit 2
- Best hit was 7tfh chain B: Replication factor C subunit 4

target	prob	fident	alnlen	evalue	theader
7tfh-assembly1.cif.gz_B	1	0.273	318	1.618e-20	Atomic model of the S. cerevisiae clamp-clamp loader complex PCNA-RFC bound to two DNA molecules, one at the 5'-recessed end and the other at the 3'-recessed end
8fs4-assembly1.cif.gz_B	1	0.273	318	6.574e-20	Structure of S. cerevisiae Rad24-RFC loading the 9-1-1 clamp onto a 10-nt gapped DNA in step 2 (open 9-1-1 ring and flexibly bound chamber DNA)
7z6h-assembly1.cif.gz_D	1	0.263	323	3.402e-19	Structure of DNA-bound human RAD17-RFC clamp loader and 9-1-1 checkpoint clamp

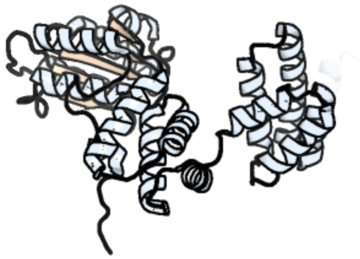

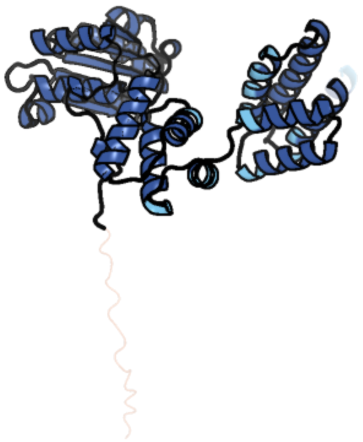

Figure 331: left: reference structure of 7tfh chain B. right: predicted structure of chlorv-1..331, unaligned sequences are shown as transparent

chlorv-1..332

- Sequence-based annotation for chlorv-1..332 is hypothetical protein
- No significant structural hit found

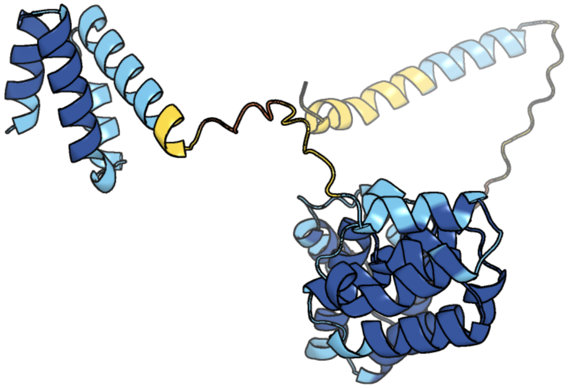

Figure 332: predicted structure of chlorv-1..332

chlorv-1..333

- Sequence-based annotation for chlorv-1..333 is putative Hsp70 protein
- Best hit was 5e84 chain F: 78 kDa glucose-regulated protein

target	prob	fident	alnlen	evalue	theader
5e84-assembly6.cif.gz_F	1	0.541	615	1.933e-70	ATP-bound state of BiP
7n1r-assembly1.cif.gz_A	1	0.544	613	3.322e-70	A novel and unique ATP hydrolysis to AMP by a human Hsp70 BiP
5tky-assembly1.cif.gz_A	1	0.499	609	5.228e-69	Crystal structure of the co-translational Hsp70 chaperone Ssb in the ATP-bound, open conformation

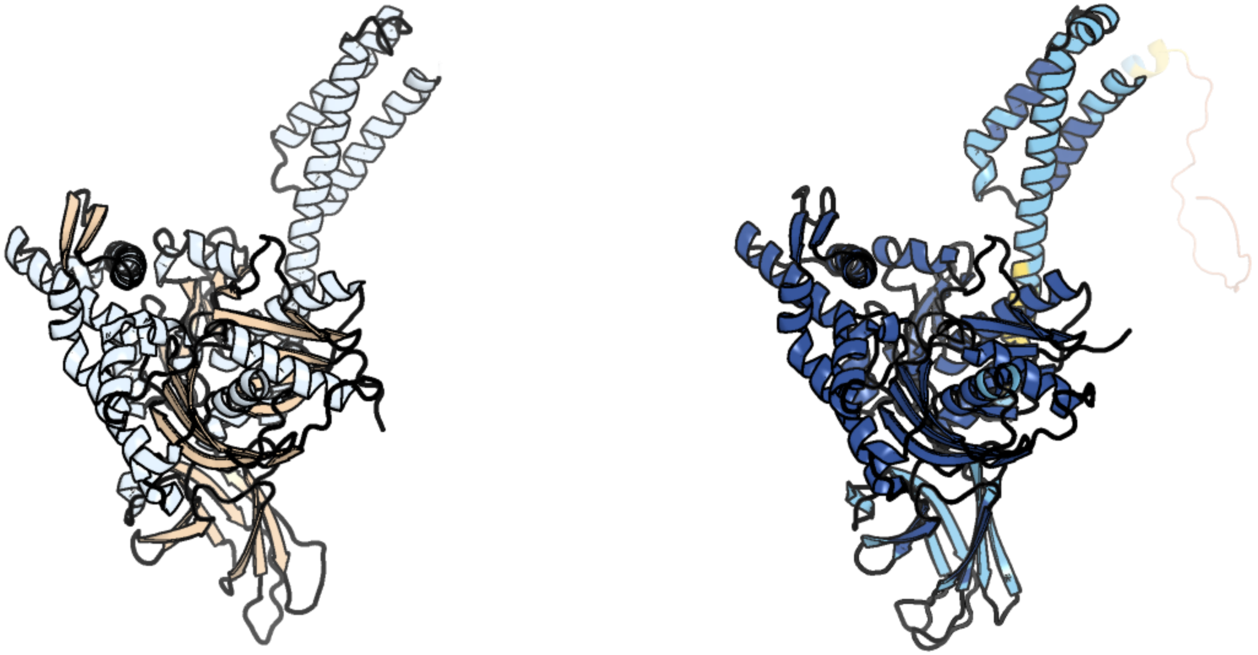

Figure 333: left: reference structure of 5e84 chain F. right: predicted structure of chlorv-1..333, unaligned sequences are shown as transparent

chlorv-1..334

- Sequence-based annotation for chlorv-1..334 is hypothetical protein
- No significant structural hit found

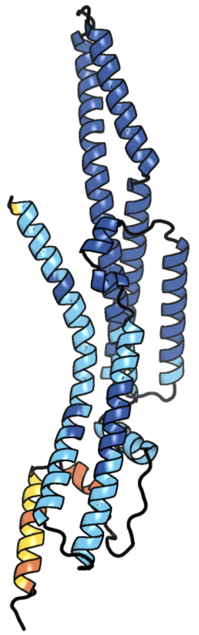

Figure 334: predicted structure of chlorv-1..334

chlorv-1..335

- Sequence-based annotation for chlorv-1..335 is hypothetical protein
- No significant structural hit found

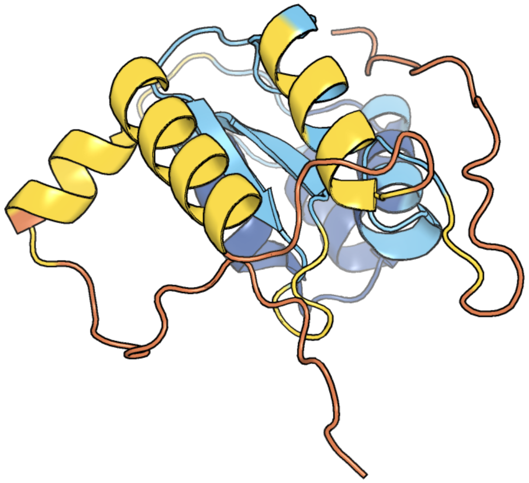

Figure 335: predicted structure of chlorv-1..335

chlorv-1..336

- Sequence-based annotation for chlorv-1..336 is putative DNA photolyase
- Best hit was 2e0i chain B: 432aa long hypothetical deoxyribodipyrimidine photolyase

target	prob	fident	alnlen	evalue	theader
2e0i-assembly2.cif.gz_B	1	0.372	465	7.309e-34	Crystal structure of archaeal photolyase from Sulfolobus tokodaii with two FAD molecules: Implication of a novel light-harvesting cofactor
1owo-assembly1.cif.gz_A	1	0.283	484	3.505e-33	DATA4:photoreduced DNA photolyase / received X-rays dose 1.2 exp15 photons/mm2
4u63-assembly1.cif.gz_A	1	0.289	487	3.866e-31	Crystal structure of a bacterial class III photolyase from Agrobacterium tumefaciens at 1.67A resolution

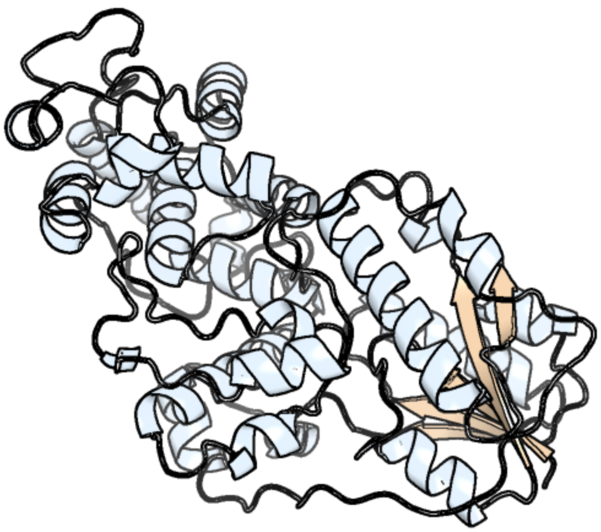

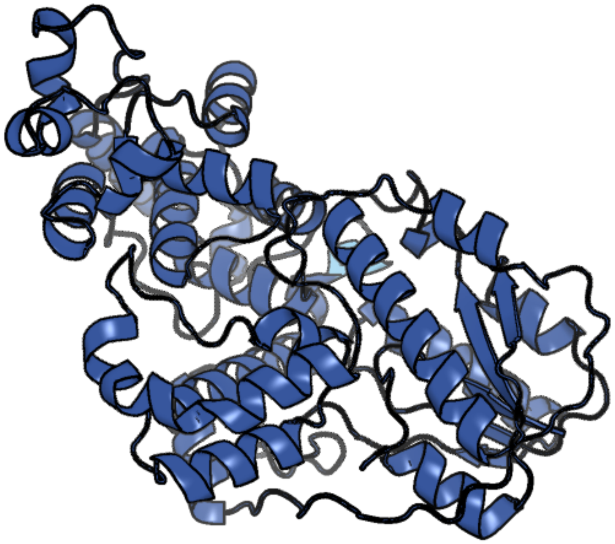

Figure 336: left: reference structure of 2e0i chain B. right: predicted structure of chlorv-1..336, unaligned sequences are shown as transparent

chlorv-1..337

- Sequence-based annotation for chlorv-1..337 is hypothetical protein
- No significant structural hit found

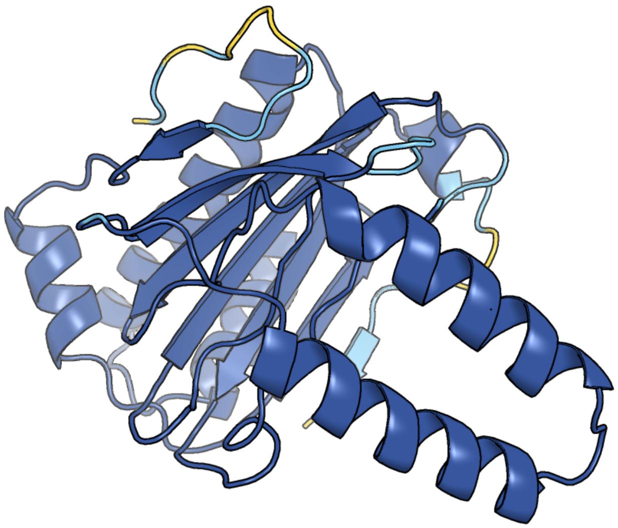

Figure 337: predicted structure of chlorv-1..337

chlorv-1..338

- Sequence-based annotation for chlorv-1..338 is hypothetical protein
- No significant structural hit found

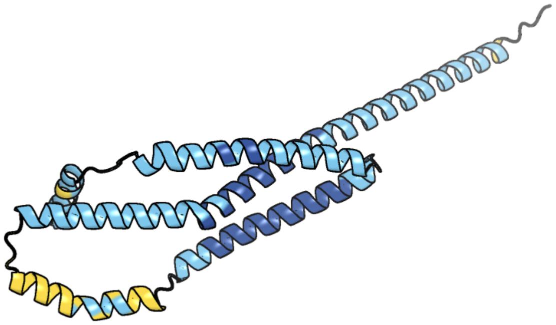

Figure 338: predicted structure of chlorv-1..338

chlorv-1..339

- Sequence-based annotation for chlorv-1..339 is putative Helicase/Zinc finger RING-type
- Best hit was 6l8n chain A: DNA repair protein RAD5

target	prob	fident	alnlen	evalue	theader
6l8n-assembly1.cif.gz_A	1	0.171	934	3.466e-29	Crystal structure of the K. lactis Rad5
7r78-assembly1.cif.gz_A	1	0.153	962	3.875e-29	cryo-EM structure of DNMT5 quaternary complex with hemimethylated DNA, AMP-PNP and SAH
7r77-assembly1.cif.gz_A	1	0.149	978	9.992e-29	Cryo-EM structure of DNMT5 binary complex with hemimethylated DNA

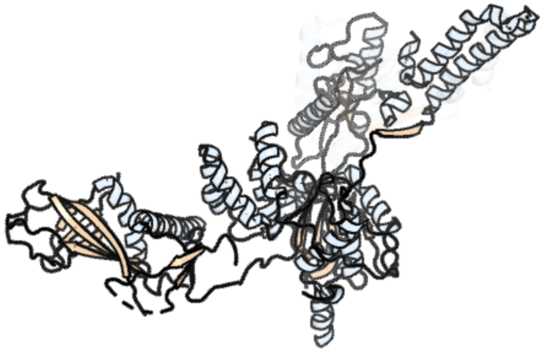

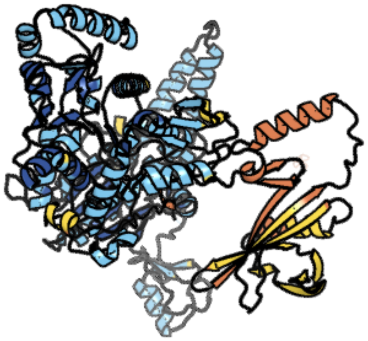

Figure 339: left: reference structure of 6l8n chain A. right: predicted structure of chlorv-1..339, unaligned sequences are shown as transparent

chlorv-1..340

- Sequence-based annotation for chlorv-1..340 is hypothetical protein
- Best hit was 2z0t chain D: Putative uncharacterized protein PH0355

target	prob	fident	alnlen	evalue	theader
2z0t-assembly4.cif.gz_D	1	0.186	102	1.459e-05	Crystal structure of hypothetical protein PH0355
1s04-assembly1.cif.gz_A	1	0.186	102	0.0002177	Solution NMR Structure of Protein PF0455 from Pyrococcus furiosus. Northeast Structural Genomics Consortium Target PfR13
1xne-assembly1.cif.gz_A	1	0.137	102	0.0009626	Solution Structure of Pyrococcus furiosus Protein PF0470: The Northeast Structural Genomics Consortium Target PfR14

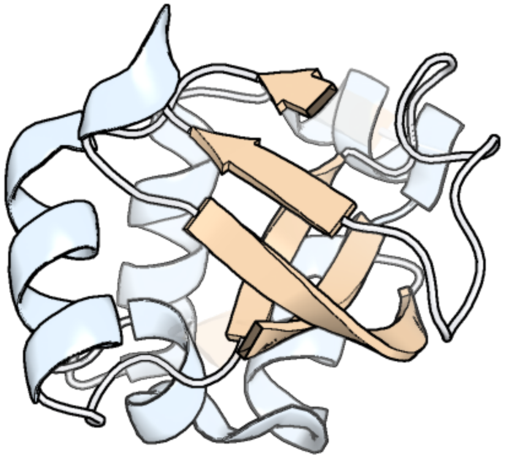

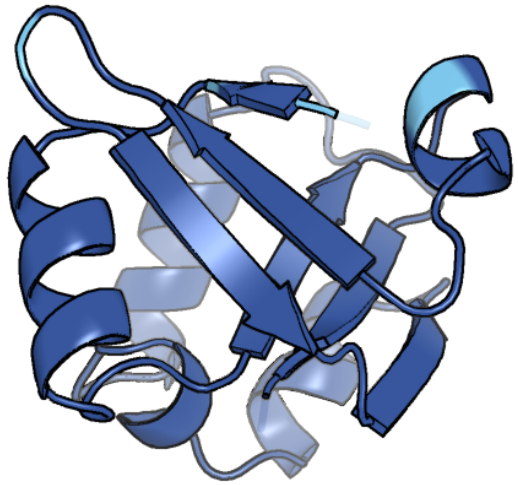

Figure 340: left: reference structure of 2z0t chain D. right: predicted structure of chlorv-1..340, unaligned sequences are shown as transparent

chlorv-1..341

- Sequence-based annotation for chlorv-1..341 is hypothetical protein
- Best hit was 2q3l chain A: Uncharacterized protein

target	prob	fident	alnlen	evalue	theader
2q3l-assembly2.cif.gz_A-2	1	0.097	123	1.407e-05	CRYSTAL STRUCTURE OF AN UNCHARACTERIZED PROTEIN FROM DUF3478 FAMILY WITH A SPOIIAA-LIKE FOLD (SHEW_3102) FROM SHEWANELLA LOIHICA PV-4 AT 2.25 A RESOLUTION
1vc1-assembly1.cif.gz_B	1	0.177	118	4.868e-05	Crystal structure of the TM1442 protein from Thermotoga maritima, a homolog of the Bacillus subtilis general stress response anti-anti-sigma factor RsbV
6m36-assembly1.cif.gz_H	1	0.155	109	8.211e-05	The crystal structure of B. subtilis RsbV/RsbW complex in the monoclinic crystal form

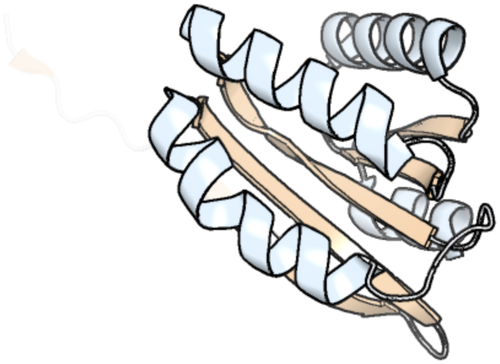

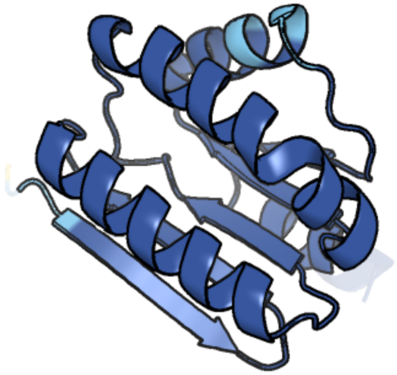

Figure 341: left: reference structure of 2q3l chain A. right: predicted structure of chlorv-1..341, unaligned sequences are shown as transparent

chlorv-1..342

- Sequence-based annotation for chlorv-1..342 is hypothetical protein
- No significant structural hit found

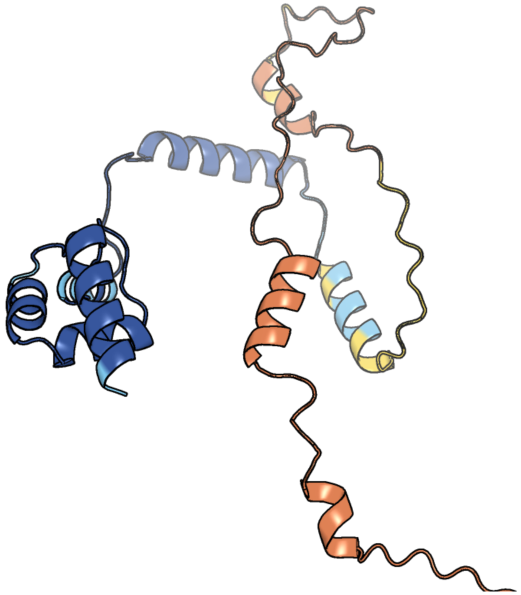

Figure 342: predicted structure of chlorv-1..342

chlorv-1..343

- Sequence-based annotation for chlorv-1..343 is putative Asparagine synthase
- Best hit was 6gq3 chain B: Asparagine synthetase [glutamine-hydrolyzing]

target	prob	fident	alnlen	evalue	theader
6gq3-assembly2.cif.gz_B	1	0.333	548	6.214e-39	Human asparagine synthetase (ASNS) in complex with 6-diazo-5-oxo-L-norleucine (DON) at 1.85 A resolution
1ct9-assembly1.cif.gz_A	1	0.314	535	2.494e-38	CRYSTAL STRUCTURE OF ASPARAGINE SYNTHETASE B FROM ESCHERICHIA COLI
1ct9-assembly1.cif.gz_D	1	0.305	533	4.641e-37	CRYSTAL STRUCTURE OF ASPARAGINE SYNTHETASE B FROM ESCHERICHIA COLI

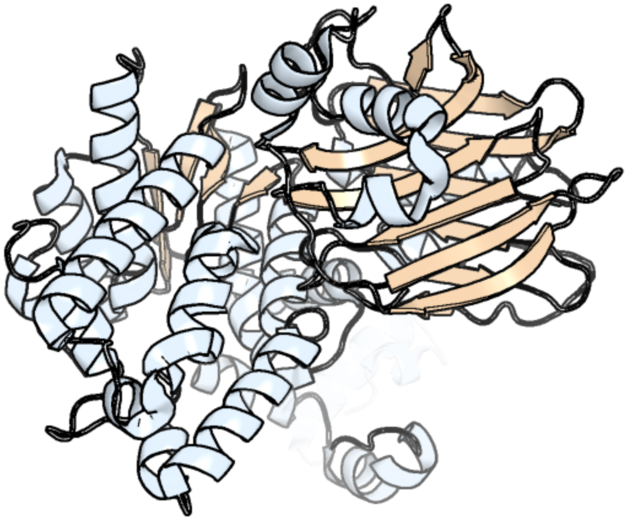

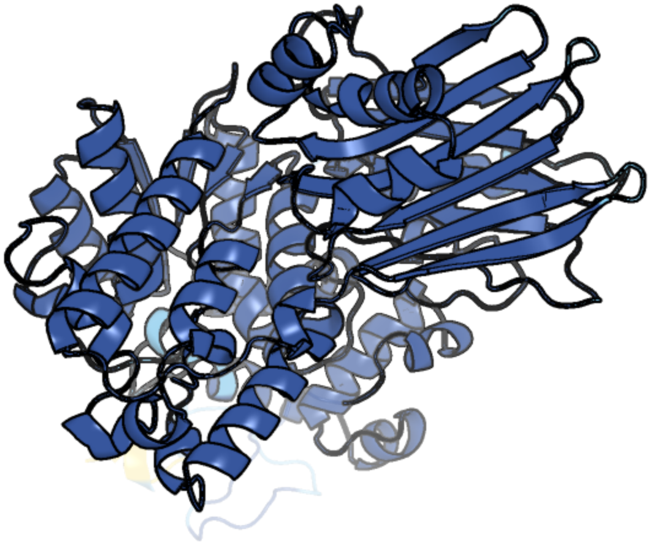

Figure 343: left: reference structure of 6gq3 chain B. right: predicted structure of chlorv-1..343, unaligned sequences are shown as transparent

chlorv-1..344

- Sequence-based annotation for chlorv-1..344 is hypothetical protein
- No significant structural hit found

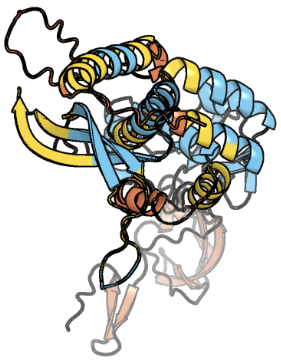

Figure 344: predicted structure of chlorv-1..344

chlorv-1..345

- Sequence-based annotation for chlorv-1..345 is hypothetical protein
- No significant structural hit found

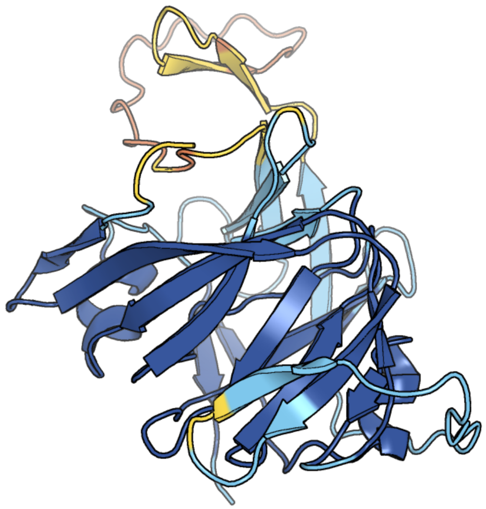

Figure 345: predicted structure of chlorv-1..345

chlorv-1..346

- Sequence-based annotation for chlorv-1..346 is hypothetical protein
- No significant structural hit found

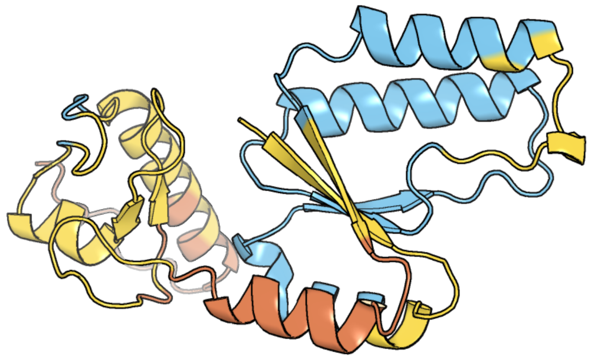

Figure 346: predicted structure of chlorv-1..346

chlorv-1..347

- Sequence-based annotation for chlorv-1..347 is hypothetical protein
- No significant structural hit found

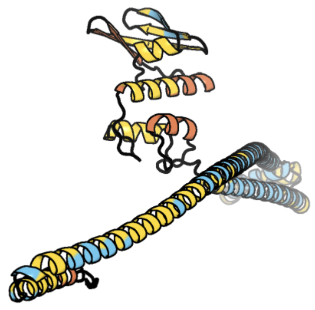

Figure 347: predicted structure of chlorv-1..347

chlorv-1..348

- Sequence-based annotation for chlorv-1..348 is putative YqaJ-like viral recombinase
- Best hit was 5yet chain B: Uncharacterized protein R354

target	prob	fident	alnlen	evalue	theader
5yet-assembly1.cif.gz_B	1	0.318	402	1.69e-29	Structure of R354_WT
5yeu-assembly1.cif.gz_A	1	0.309	401	7.06e-28	Structural and mechanistic analyses reveal a unique Cas4-like protein in the mimivirus virophage resistance element system
5yeu-assembly1.cif.gz_B	1	0.312	403	9.518e-27	Structural and mechanistic analyses reveal a unique Cas4-like protein in the mimivirus virophage resistance element system

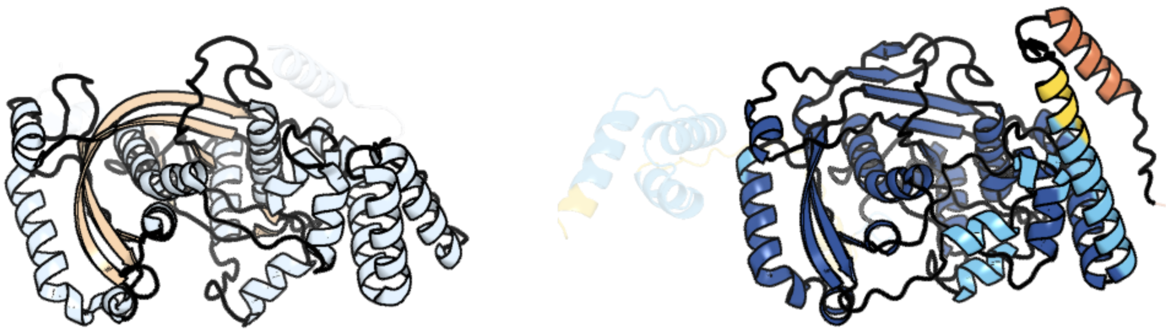

Figure 348: left: reference structure of 5yet chain B. right: predicted structure of chlorv-1..348, unaligned sequences are shown as transparent

chlorv-1..349

- Sequence-based annotation for chlorv-1..349 is hypothetical protein
- No significant structural hit found

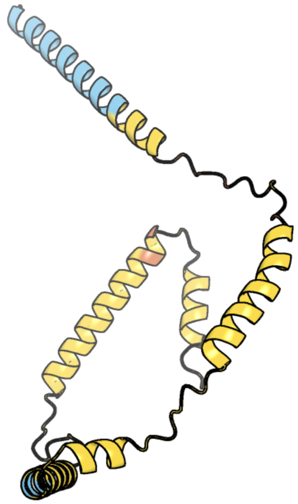

Figure 349: predicted structure of chlorv-1..349

chlorv-1..350

- Sequence-based annotation for chlorv-1..350 is putative DNA ligase/mRNA capping enzyme
- Best hit was 1ckm chain A: MRNA CAPPING ENZYME

target	prob	fident	alnlen	evalue	theader
1ckm-assembly1.cif.gz_A	1	0.128	350	1.34e-12	STRUCTURE OF TWO DIFFERENT CONFORMATIONS OF MRNA CAPPING ENZYME IN COMPLEX WITH GTP
4pz8-assembly1.cif.gz_A	1	0.144	361	1.092e-11	PCE1 guanylyltransferase bound to SPT5 CTD
3kyh-assembly1.cif.gz_D	1	0.117	393	9.41e-11	Saccharomyces cerevisiae Cet1-Ceg1 capping apparatus

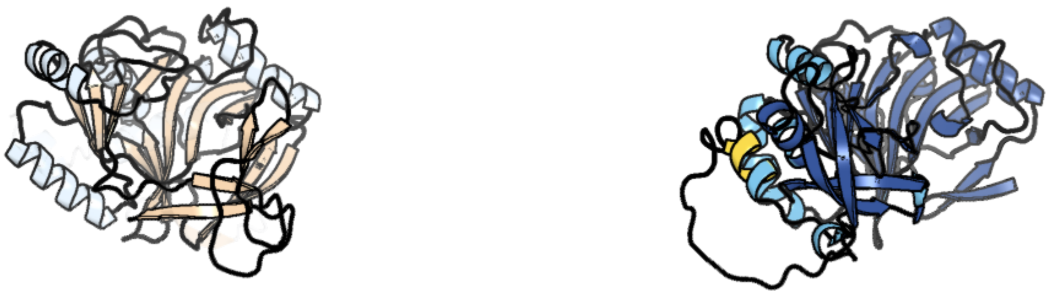

Figure 350: left: reference structure of 1ckm chain A. right: predicted structure of chlorv-1..350, unaligned sequences are shown as transparent

chlorv-1..351

- Sequence-based annotation for chlorv-1..351 is putative Aspartyl/Asparaginyl beta-hydroxylase
- Best hit was 5apa chain A: ASPARTYL/ASPARAGINYL BETA-HYDROXYLASE

target	prob	fident	alnlen	evalue	theader
5apa-assembly1.cif.gz_A	1	0.211	175	5.779e-12	Crystal structure of human aspartate beta-hydroxylase isoform a
6q9i-assembly1.cif.gz_A	1	0.194	180	1.734e-11	Aspartyl/Asparaginyl beta-hydroxylase (AspH) H679A in complex with Factor X peptide fragment (39mer-4Ser)
7bmj-assembly1.cif.gz_A	1	0.202	178	4.605e-11	Aspartyl/Asparaginyl beta-hydroxylase (AspH) oxygenase and TPR domains in complex with manganese, 5-fluoropyridine-2,4-dicarboxylic acid, and factor X substrate peptide fragment (39mer-4Ser)

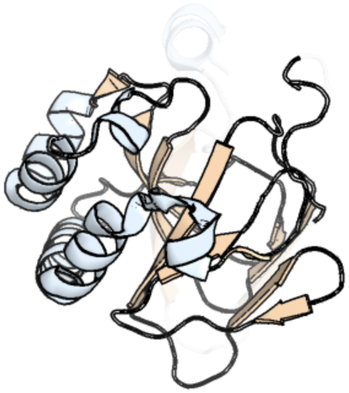

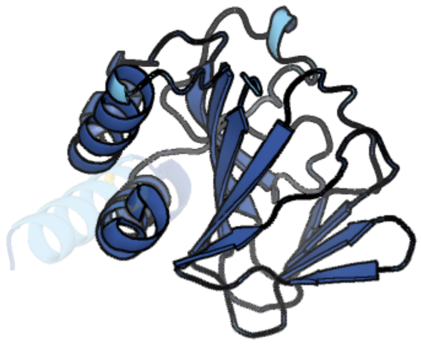

Figure 351: left: reference structure of 5apa chain A. right: predicted structure of chlorv-1..351, unaligned sequences are shown as transparent

chlorv-1..352

- Sequence-based annotation for chlorv-1..352 is putative Aspartyl/Asparaginyl beta-hydroxylase
- Best hit was 5apa chain A: ASPARTYL/ASPARAGINYL BETA-HYDROXYLASE

target	prob	fident	alnlen	evalue	theader
5apa-assembly1.cif.gz_A	1	0.225	213	1.386e-13	Crystal structure of human aspartate beta-hydroxylase isoform a
6q9i-assembly1.cif.gz_A	1	0.202	212	1.092e-12	Aspartyl/Asparaginyl beta-hydroxylase (AspH) H679A in complex with Factor X peptide fragment (39mer-4Ser)
7bmj-assembly1.cif.gz_A	1	0.216	212	1.092e-12	Aspartyl/Asparaginyl beta-hydroxylase (AspH) oxygenase and TPR domains in complex with manganese, 5-fluoropyridine-2,4-dicarboxylic acid, and factor X substrate peptide fragment (39mer-4Ser)

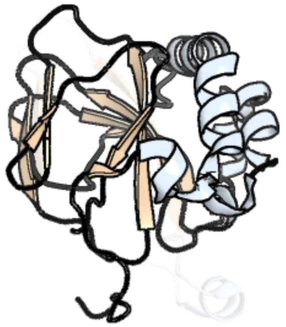

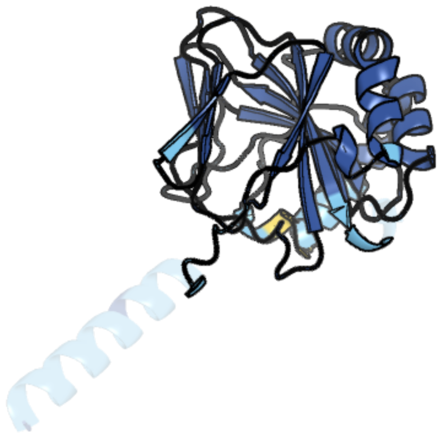

Figure 352: left: reference structure of 5apa chain A. right: predicted structure of chlorv-1..352, unaligned sequences are shown as transparent

chlorv-1..353

- Sequence-based annotation for chlorv-1..353 is putative Protein phosphatase 2C
- Best hit was 4n0g chain B: Protein phosphatase 2C 37

target	prob	fident	alnlen	evalue	theader
4n0g-assembly2.cif.gz_B	1	0.288	274	2.895e-24	Crystal Structure of PYL13-PP2CA complex
3nmv-assembly1.cif.gz_B	1	0.258	282	6.575e-24	Crystal structure of pyrabactin-bound abscisic acid receptor PYL2 mutant A93F in complex with type 2C protein phosphatase ABI2
5gwp-assembly1.cif.gz_A	1	0.232	310	1.493e-23	Crystal structure of RCAR3:PP2C wild-type with (+)-ABA

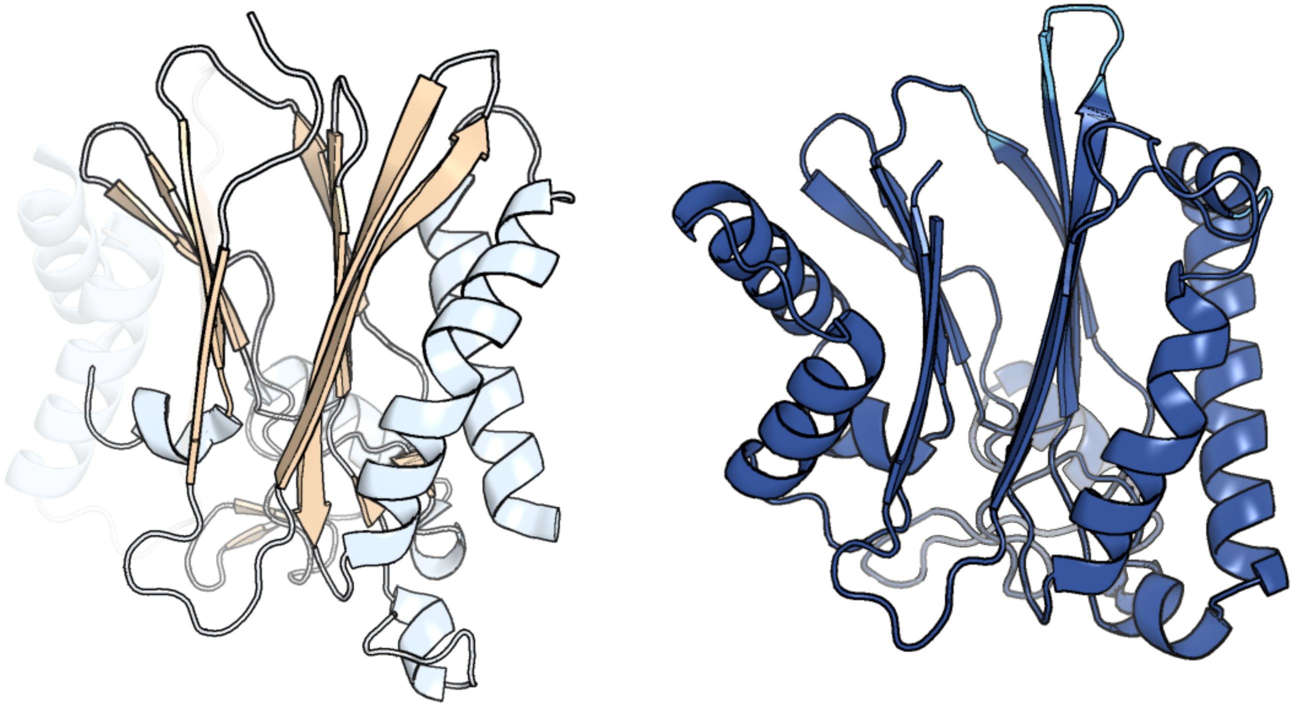

Figure 353: left: reference structure of 4n0g chain B. right: predicted structure of chlorv-1..353, unaligned sequences are shown as transparent

chlorv-1..354

- Sequence-based annotation for chlorv-1..354 is putative DNA mismatch repair protein MutS
- Best hit was 7ai6 chain B: DNA mismatch repair protein MutS

target	prob	fident	alnlen	evalue	theader
7ai6-assembly1.cif.gz_B	1	0.191	877	1.021e-43	MutS in mismatch bound state
3k0s-assembly1.cif.gz_A	1	0.191	867	1.124e-43	Crystal structure of E.coli DNA mismatch repair protein MutS, D693N mutant, in complex with GT mismatched DNA
1ng9-assembly1.cif.gz_A	1	0.193	865	1.902e-43	E.coli MutS R697A: an ATPase-asymmetry mutant

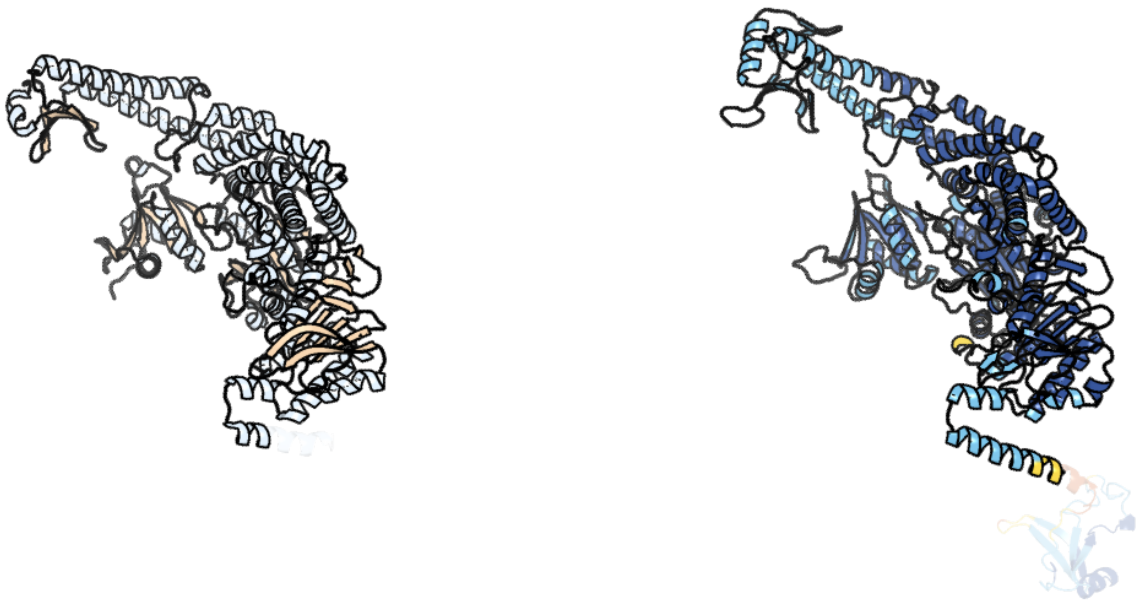

Figure 354: left: reference structure of 7ai6 chain B. right: predicted structure of chlorv-1..354, unaligned sequences are shown as transparent

chlorv-1..355

- Sequence-based annotation for chlorv-1..355 is hypothetical protein
- No significant structural hit found

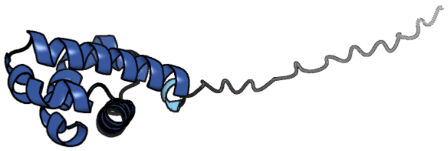

Figure 355: predicted structure of chlorv-1..355

chlorv-1..356

- Sequence-based annotation for chlorv-1..356 is hypothetical protein
- No significant structural hit found

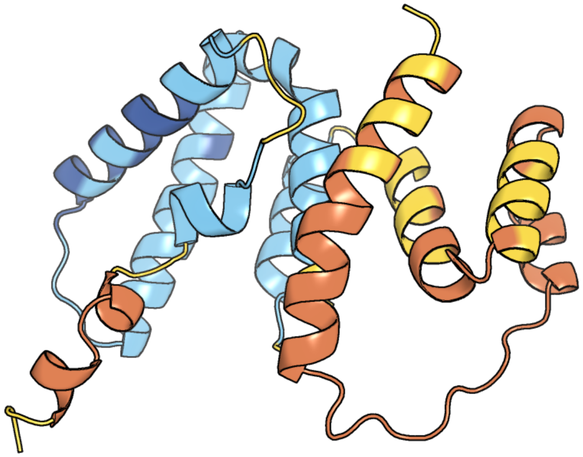

Figure 356: predicted structure of chlorv-1..356

chlorv-1..357

- Sequence-based annotation for chlorv-1..357 is putative Protein kinase
- Best hit was 5mxx chain A: SRPK1

target	prob	fident	alnlen	evalue	theader
5mxx-assembly1.cif.gz_A	1	0.288	367	1.209e-23	Crystal structure of human SR protein kinase 1 (SRPK1) in complex with compound 1
7zks-assembly1.cif.gz_A	1	0.279	368	2.937e-23	SRPK1 IN COMPLEX WITH INHIBITOR
7pqs-assembly2.cif.gz_B	1	0.287	369	3.435e-23	SRPK1 in complex with MSC2711186

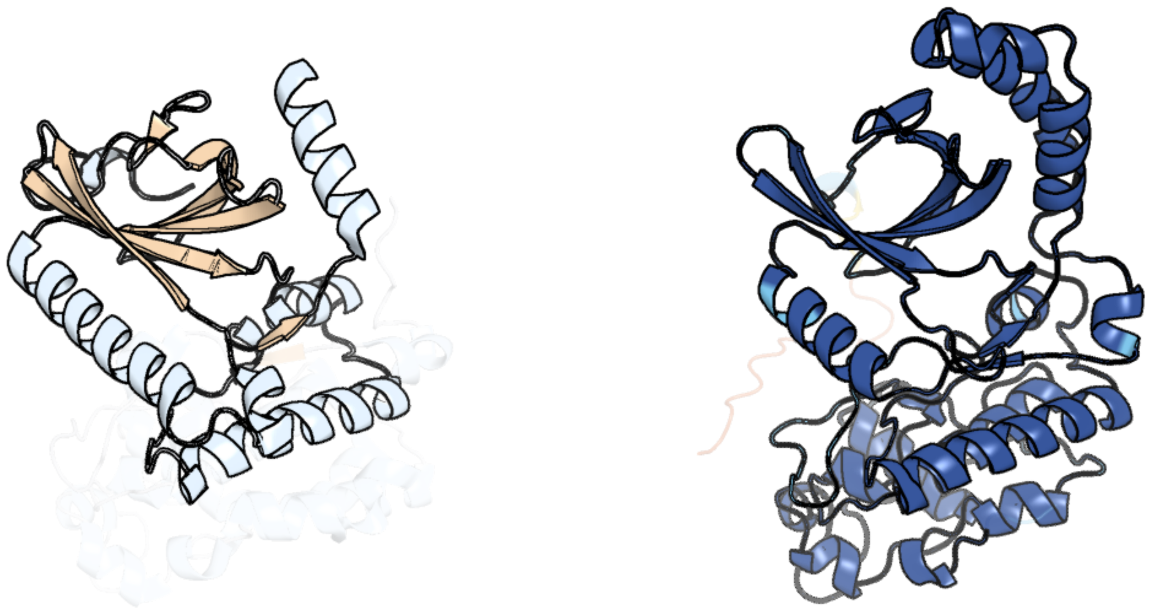

Figure 357: left: reference structure of 5mxx chain A. right: predicted structure of chlorv-1..357, unaligned sequences are shown as transparent

chlorv-1..358

- Sequence-based annotation for chlorv-1..358 is hypothetical protein
- No significant structural hit found

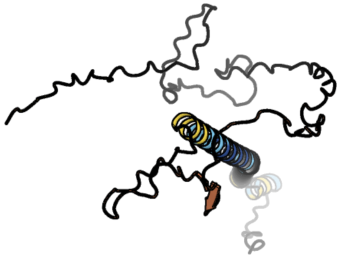

Figure 358: predicted structure of chlorv-1..358

chlorv-1..359

- Sequence-based annotation for chlorv-1..359 is putative RNA polymerase
- Best hit was 4a3c chain F: DNA-DIRECTED RNA POLYMERASES I, II, AND III SUBUNIT RPABC 2

target	prob	fident	alnlen	evalue	theader
4a3c-assembly1.cif.gz_F	1	0.392	84	5.185e-08	RNA Polymerase II initial transcribing complex with a 5nt DNA-RNA hybrid
7zsb-assembly1.cif.gz_F	1	0.308	120	1.806e-07	Yeast RNA polymerase II transcription pre-initiation complex with the +1 nucleosome and NTP, complex C
2nvx-assembly1.cif.gz_F	1	0.373	83	2.059e-07	RNA polymerase II elongation complex in 5 mM Mg+2 with 2'-dUTP

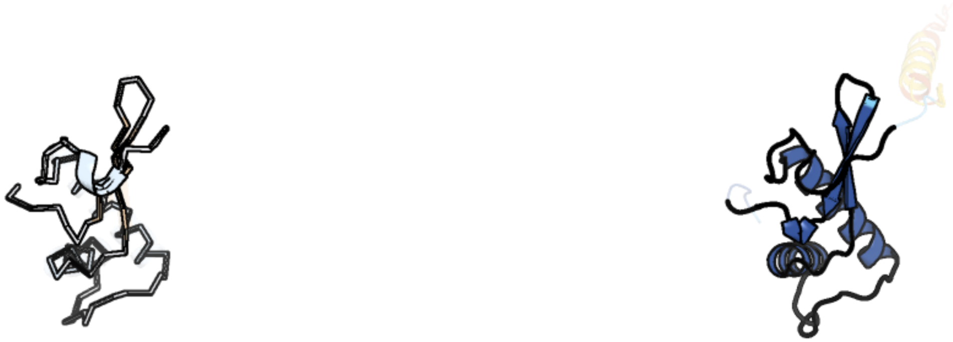

Figure 359: left: reference structure of 4a3c chain F. right: predicted structure of chlorv-1..359, unaligned sequences are shown as transparent

chlorv-1..360

- Sequence-based annotation for chlorv-1..360 is hypothetical protein
- No significant structural hit found

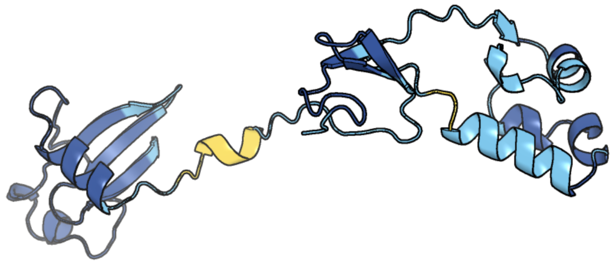

Figure 360: predicted structure of chlorv-1..360

chlorv-1..361

- Sequence-based annotation for chlorv-1..361 is putative D5-like helicase-primase
- Best hit was 8iqi chain B: Putative primase C962R

target	prob	fident	alnlen	evalue	theader
8iqi-assembly1.cif.gz_B	1	0.191	951	1.364e-39	Structure of Full-Length AsfvPrimPol in Complex-Form
8iqi-assembly1.cif.gz_A	1	0.193	948	2.121e-39	Structure of Full-Length AsfvPrimPol in Complex-Form
8iqi-assembly1.cif.gz_E	1	0.193	954	5.811e-38	Structure of Full-Length AsfvPrimPol in Complex-Form

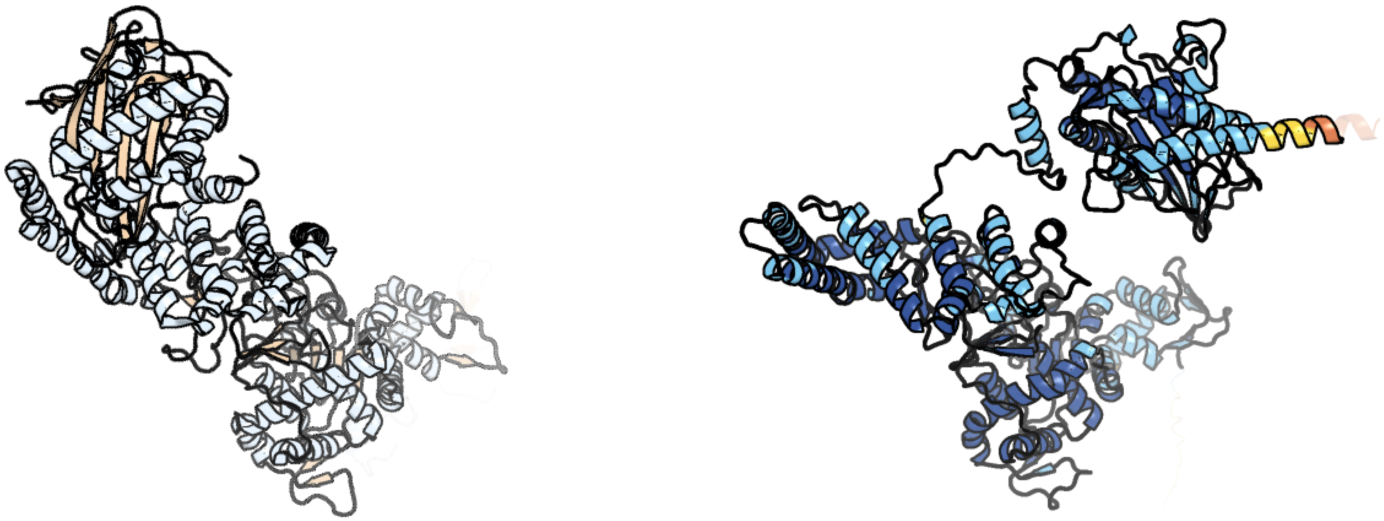

Figure 361: left: reference structure of 8iqi chain B. right: predicted structure of chlorv-1..361, unaligned sequences are shown as transparent

chlorv-1..362

- Sequence-based annotation for chlorv-1..362 is putative Zinc finger
- No significant structural hit found

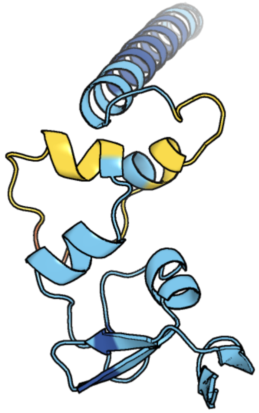

Figure 362: predicted structure of chlorv-1..362

chlorv-1..363

- Sequence-based annotation for chlorv-1..363 is hypothetical protein
- No significant structural hit found

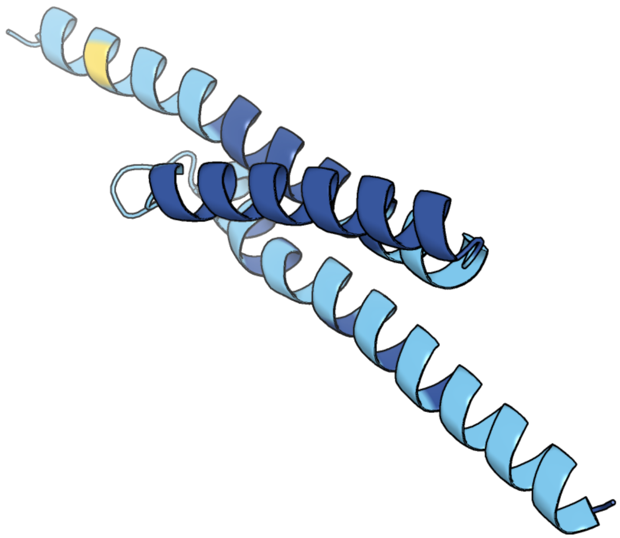

Figure 363: predicted structure of chlorv-1..363

chlorv-1..364

- Sequence-based annotation for chlorv-1..364 is putative HD phosphohydrolase
- Best hit was 5yhw chain C: Deoxynucleoside triphosphate triphosphohydrolase SAMHD1

target	prob	fident	alnlen	evalue	theader
5yhw-assembly2.cif.gz_C	1	0.24	433	1.724e-21	Crystal structure of Pig SAMHD1
7ltt-assembly1.cif.gz_D	1	0.226	442	6.897e-21	SAMHD1(113-626) H206R D207N R366C
7a5y-assembly2.cif.gz_H	1	0.236	436	1.06e-20	Crystal structure of tetrameric human H215A-SAMHD1 (residues 109-626) with Rp-dGTP-alphaS (T8T) and Mg

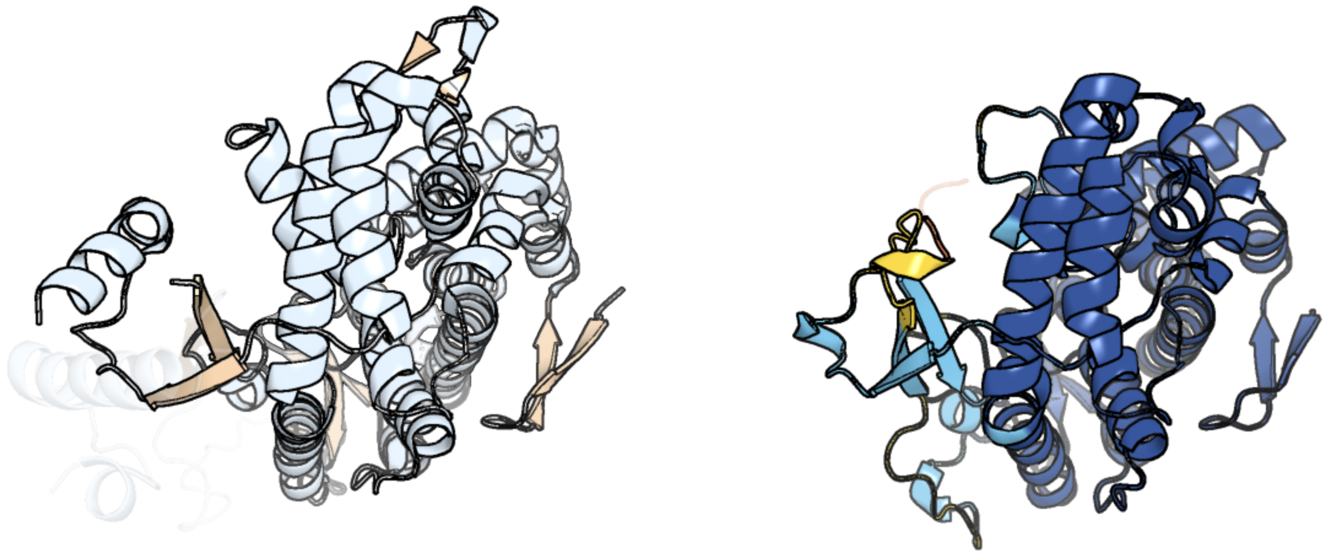

Figure 364: left: reference structure of 5yhw chain C. right: predicted structure of chlorv-1..364, unaligned sequences are shown as transparent

chlorv-1..365

- Sequence-based annotation for chlorv-1..365 is putative Nuclease
- Best hit was 2ihn chain A: Ribonuclease H

target	prob	fident	alnlen	evalue	theader
2ihn-assembly1.cif.gz_A	1	0.212	287	1.612e-11	Co-crystal of Bacteriophage T4 RNase H with a fork DNA substrate
3h8s-assembly1.cif.gz_A	1	0.217	289	8.087e-11	Structure of D19N T4 RNase H in the presence of divalent magnesium
3h7i-assembly1.cif.gz_A	1	0.225	297	1.541e-10	Structure of the metal-free D132N T4 RNase H

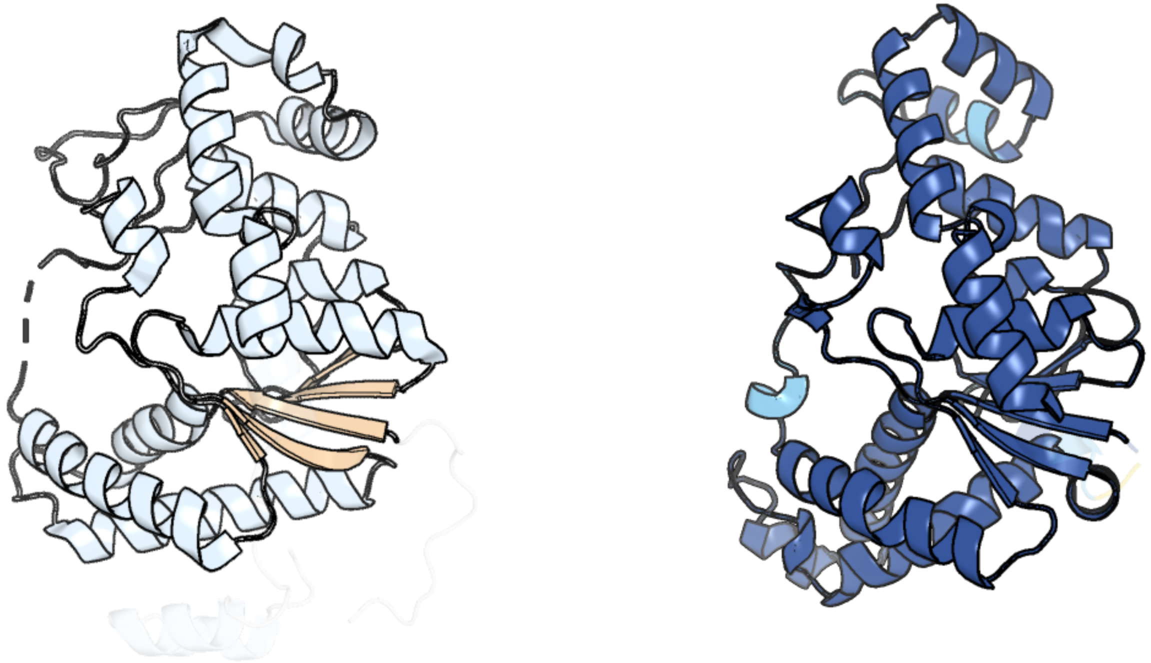

Figure 365: left: reference structure of 2ihn chain A. right: predicted structure of chlorv-1..365, unaligned sequences are shown as transparent

chlorv-1..366

- Sequence-based annotation for chlorv-1..366 is putative Peptidyl-tRNA hydrolase
- Best hit was 1xty chain D: Peptidyl-tRNA hydrolase

target	prob	fident	alnlen	evalue	theader
1xty-assembly2.cif.gz_D	1	0.403	119	4.227e-12	Crystal structure of Sulfolobus solfataricus peptidyl-tRNA hydrolase
2zv3-assembly4.cif.gz_G	1	0.377	114	6.339e-12	Crystal structure of project MJ0051 from Methanocaldococcus jannaschii DSM 2661
2zv3-assembly2.cif.gz_C	1	0.37	116	1.088e-11	Crystal structure of project MJ0051 from Methanocaldococcus jannaschii DSM 2661

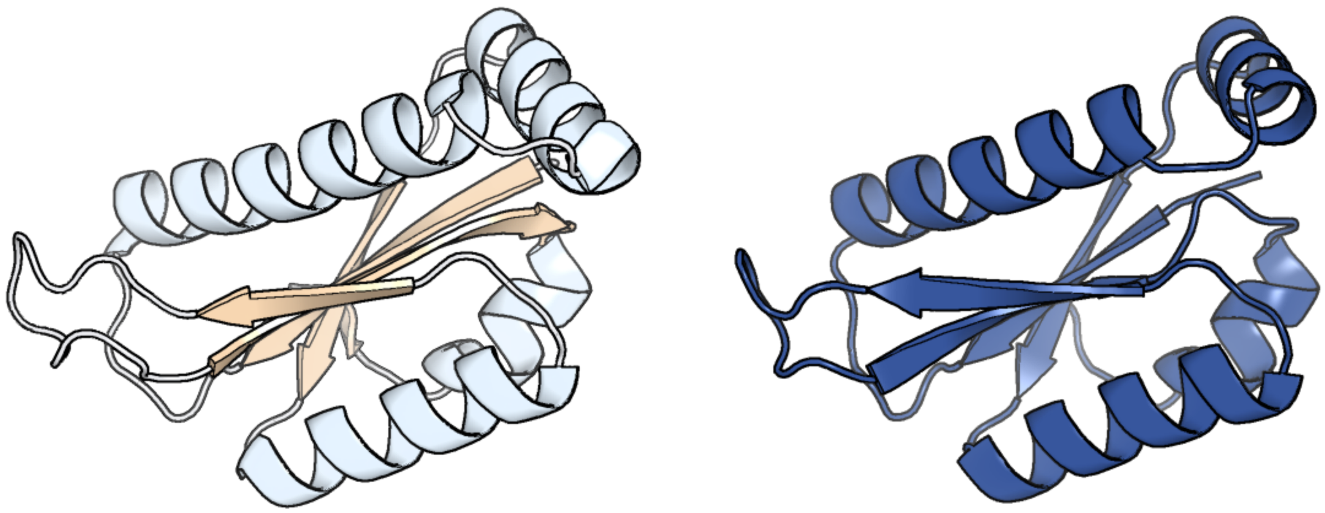

Figure 366: left: reference structure of 1xty chain D. right: predicted structure of chlorv-1..366, unaligned sequences are shown as transparent

chlorv-1..367

- Sequence-based annotation for chlorv-1..367 is hypothetical protein
- Best hit was 5uy6 chain A: Calcium/calmodulin-dependent protein kinase kinase 2

target	prob	fident	alnlen	evalue	theader
5uy6-assembly1.cif.gz_A	1	0.122	302	1.988e-06	Crystal Structure of the Human CAMKK2B
8sam-assembly1.cif.gz_A	1	0.114	384	7.441e-06	Crystal structure of class III lanthipeptide synthetase LP-GS-ThurKC in complex with ATP
8tuc-assembly1.cif.gz_A	1	0.122	301	3.109e-05	Unphosphorylated CaMKK2 in complex with CC-8977

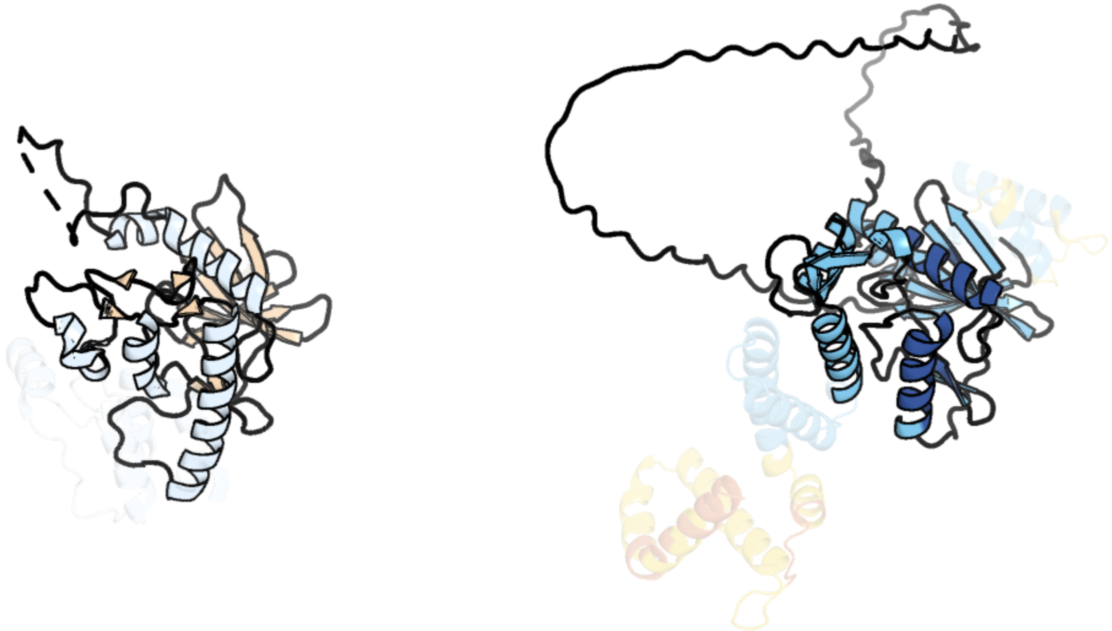

Figure 367: left: reference structure of 5uy6 chain A. right: predicted structure of chlorv-1..367, unaligned sequences are shown as transparent

chlorv-1..368

- Sequence-based annotation for chlorv-1..368 is putative MutM DNA repair protein formamidopyrimidine-DNA glycosylase H2TH domain
- Best hit was 3a46 chain B: Formamidopyrimidine-DNA glycosylase

target	prob	fident	alnlen	evalue	theader
3a46-assembly2.cif.gz_B	1	0.332	304	2.284e-23	Crystal structure of MvNei1/THF complex
3a42-assembly1.cif.gz_A	1	0.321	299	3.664e-23	Crystal structure of MvNei1
3vk7-assembly2.cif.gz_B	1	0.314	305	8.885e-23	Crystal structure of DNA-glycosylase bound to DNA containing 5-Hydroxyuracil

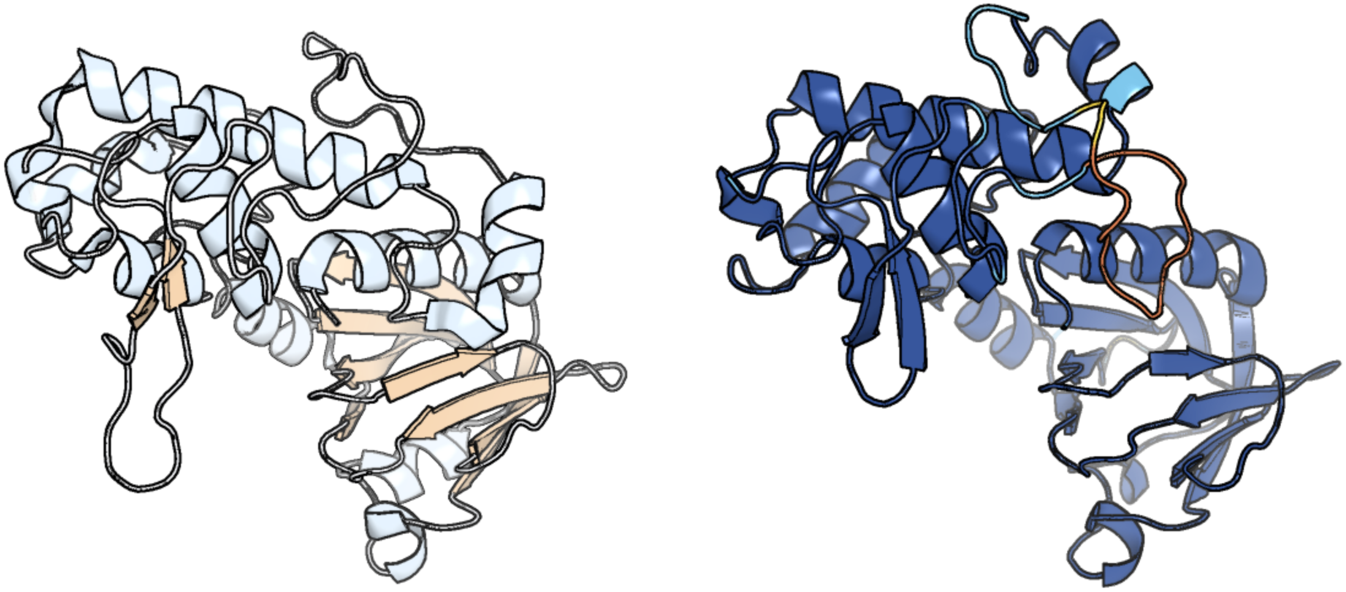

Figure 368: left: reference structure of 3a46 chain B. right: predicted structure of chlorv-1..368, unaligned sequences are shown as transparent

chlorv-1..369

- Sequence-based annotation for chlorv-1..369 is hypothetical protein
- No significant structural hit found

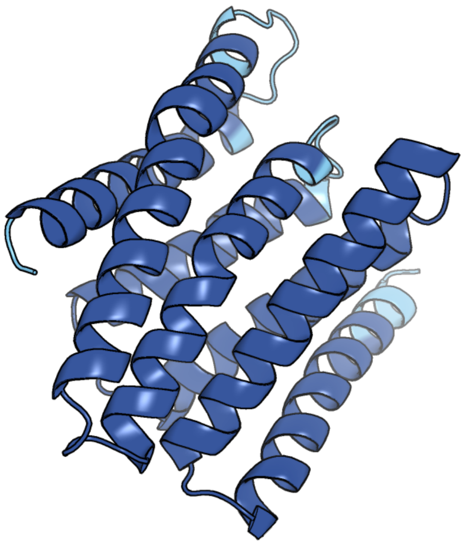

Figure 369: predicted structure of chlorv-1..369

chlorv-1..370

- Sequence-based annotation for chlorv-1..370 is putative Ribonucleotide reductase large chain
- Best hit was 3hnc chain B: Ribonucleoside-diphosphate reductase large subunit

target	prob	fident	alnlen	evalue	theader
3hnc-assembly1.cif.gz_B	1	0.455	860	8.587e-81	Crystal structure of human ribonucleotide reductase 1 bound to the effector TTP
3hnf-assembly1.cif.gz_B	1	0.453	860	1.151e-80	Crystal structure of human ribonucleotide reductase 1 bound to the effectors TTP and dATP
5tus-assembly3.cif.gz_B	1	0.452	860	2.164e-79	Potent competitive inhibition of human ribonucleotide reductase by a novel non-nucleoside small molecule

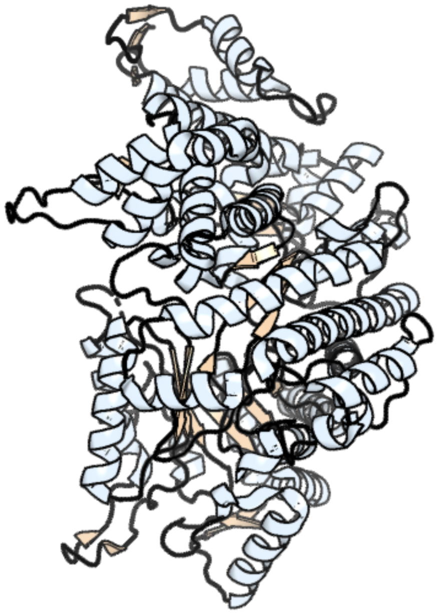

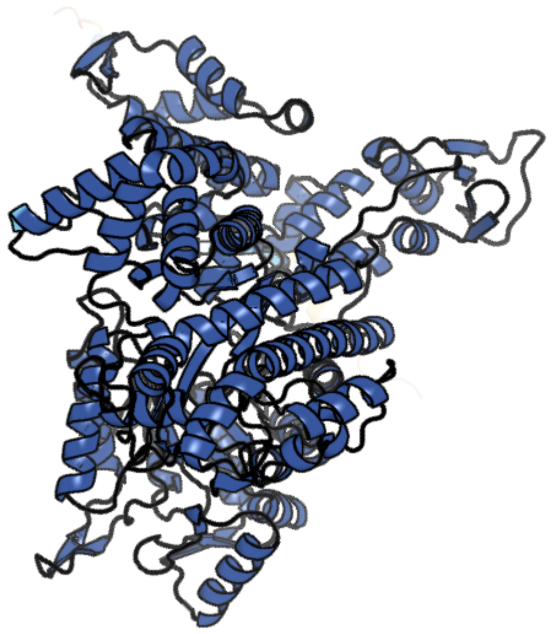

Figure 370: left: reference structure of 3hnc chain B. right: predicted structure of chlorv-1..370, unaligned sequences are shown as transparent

chlorv-1..371

- Sequence-based annotation for chlorv-1..371 is hypothetical protein
- No significant structural hit found

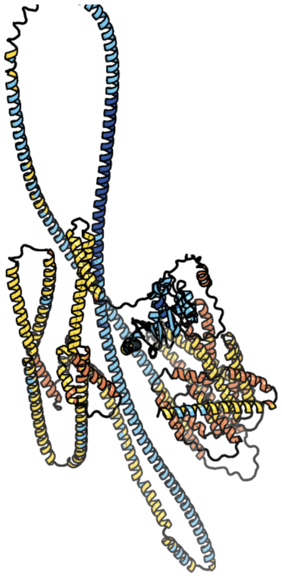

Figure 371: predicted structure of chlorv-1..371

chlorv-1..372

- Sequence-based annotation for chlorv-1..372 is putative DNA topoisomerase I
- Best hit was 4rul chain A: DNA topoisomerase 1

target	prob	fident	alnlen	evalue	theader
4rul-assembly1.cif.gz_A	1	0.312	793	1.098e-55	Crystal structure of full-length E.Coli topoisomerase I in complex with ssDNA
6pcm-assembly1.cif.gz_A	1	0.304	846	2.252e-53	Crystal Structure of Mycobacterium smegmatis Topoisomerase I with ssDNA bound to both N- and C-terminal domains
6ozw-assembly1.cif.gz_A	1	0.368	605	4.138e-53	Crystal structure of the 65-kilodalton amino-terminal fragment of DNA topoisomerase I from Streptococcus mutans

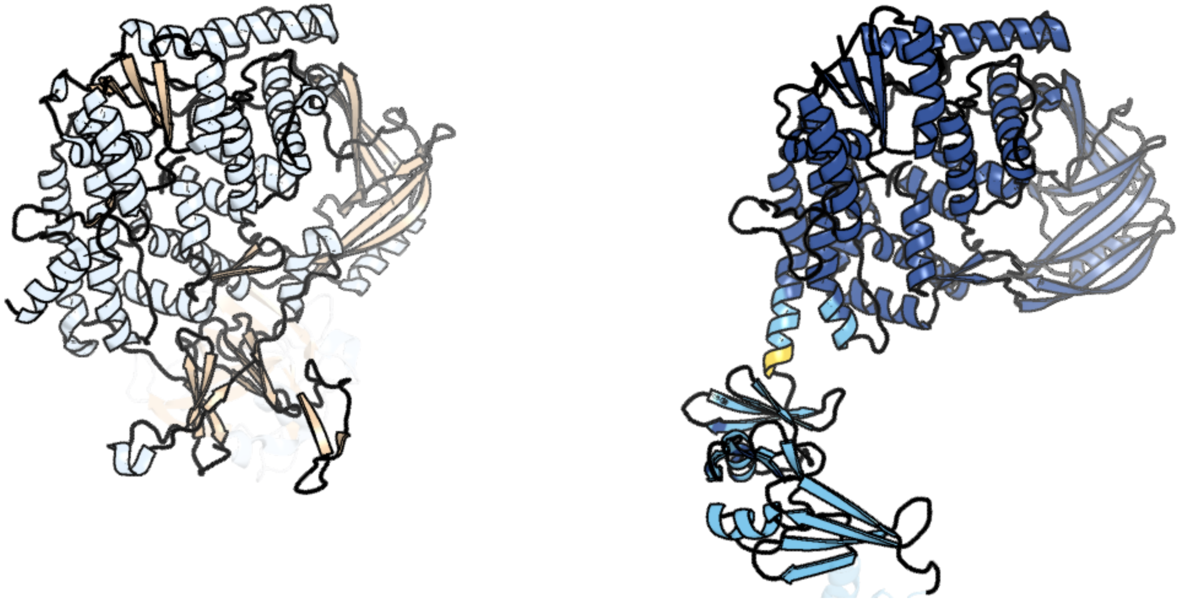

Figure 372: left: reference structure of 4rul chain A. right: predicted structure of chlorv-1..372, unaligned sequences are shown as transparent

chlorv-1..373

- Sequence-based annotation for chlorv-1..373 is hypothetical protein
- No significant structural hit found

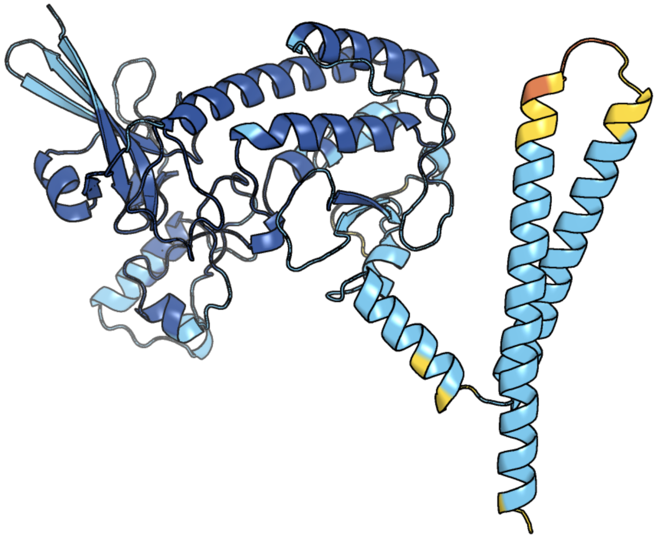

Figure 373: predicted structure of chlorv-1..373

chlorv-1..374

- Sequence-based annotation for chlorv-1..374 is putative Methyltransferase
- Best hit was 7eew chain A: Type I restriction-modification system methyltransferase subunit

target	prob	fident	alnlen	evalue	theader
7eew-assembly1.cif.gz_A-2	1	0.165	574	1.767e-18	Crystal structure of the intact MTase from Vibrio vulnificus YJ016 in complex with the DNA-mimicking Ocr protein and the S-adenosyl-L-homocysteine (SAH)
8cy2-assembly1.cif.gz_A	1	0.167	579	2.574e-15	CamA Adenine Methyltransferase Complexed to Cognate Substrate DNA and Inhibitor APNEA (Compound 9)
8cxt-assembly1.cif.gz_A	1	0.162	577	3.406e-15	CamA Adenine Methyltransferase Complexed to Cognate Substrate DNA and Inhibitor N6-benzyladenosine (Compound 1)

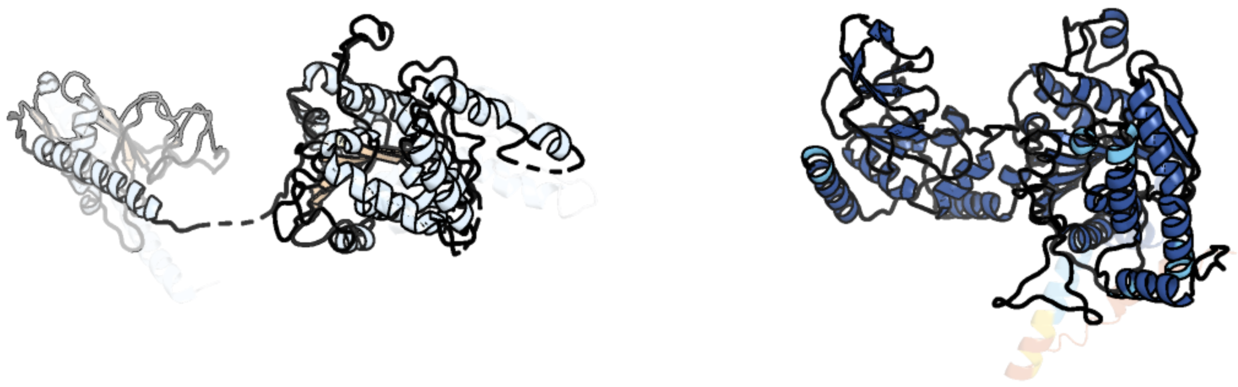

Figure 374: left: reference structure of 7eew chain A. right: predicted structure of chlorv-1..374, unaligned sequences are shown as transparent

chlorv-1..375

- Sequence-based annotation for chlorv-1..375 is hypothetical protein
- No significant structural hit found

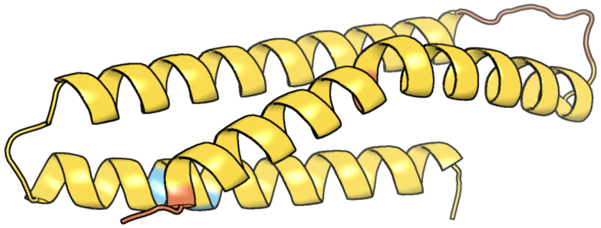

Figure 375: predicted structure of chlorv-1..375

chlorv-1..376

- Sequence-based annotation for chlorv-1..376 is hypothetical protein
- No significant structural hit found

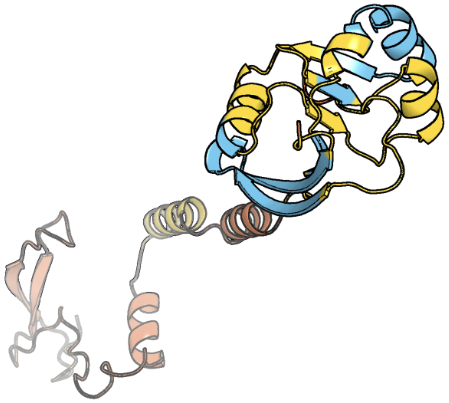

Figure 376: predicted structure of chlorv-1..376

chlorv-1..377

- Sequence-based annotation for chlorv-1..377 is hypothetical protein
- No significant structural hit found

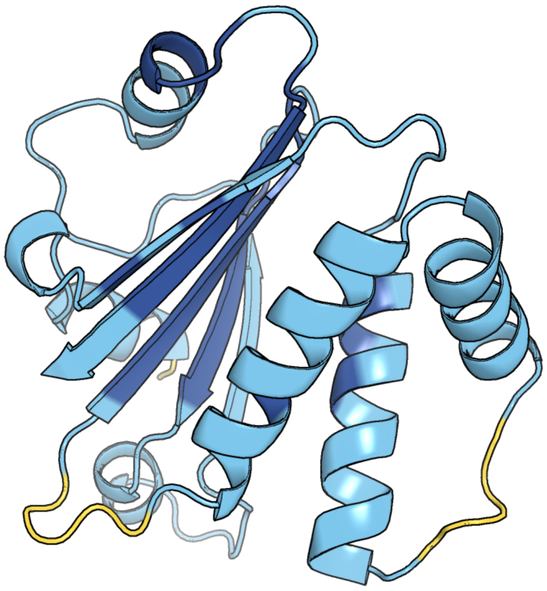

Figure 377: predicted structure of chlorv-1..377

chlorv-1..378

- Sequence-based annotation for chlorv-1..378 is putative Peptidase
- Best hit was 3mt6 chain T: ATP-dependent Clp protease proteolytic subunit

target	prob	fident	alnlen	evalue	theader
3mt6-assembly2.cif.gz_K	1	0.206	165	2.459e-15	Structure of ClpP from Escherichia coli in complex with ADEP1
3mt6-assembly1.cif.gz_T	1	0.206	165	2.961e-15	Structure of ClpP from Escherichia coli in complex with ADEP1
4emp-assembly1.cif.gz_F	1	0.242	165	3.795e-15	Crystal structure of the mutant of ClpP E137A from Staphylococcus aureus

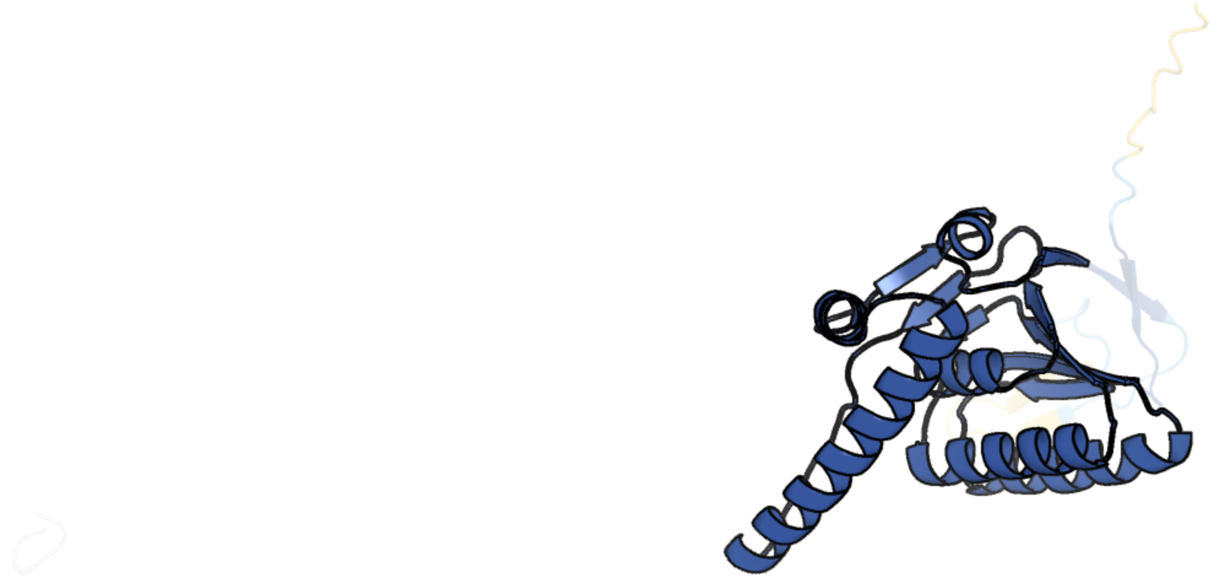

Figure 378: left: reference structure of 3mt6 chain T. right: predicted structure of chlorv-1..378, unaligned sequences are shown as transparent

chlorv-1..379

- Sequence-based annotation for chlorv-1..379 is putative Transcription factor/TATA-binding protein
- Best hit was 8cen chain O: TATA-binding protein

target	prob	fident	alnlen	evalue	theader
4b0a-assembly1.cif.gz_A	1	0.17	258	2.95e-09	The high-resolution structure of yTBP-yTAF1 identifies conserved and competing interaction surfaces in transcriptional activation
1mp9-assembly1.cif.gz_B	1	0.181	248	4.135e-09	TBP from a mesothermophilic archaeon, Sulfolobus acidocaldarius
8cen-assembly1.cif.gz_O	1	0.158	246	5.179e-09	Yeast RNA polymerase II transcription pre-initiation complex with core Mediator

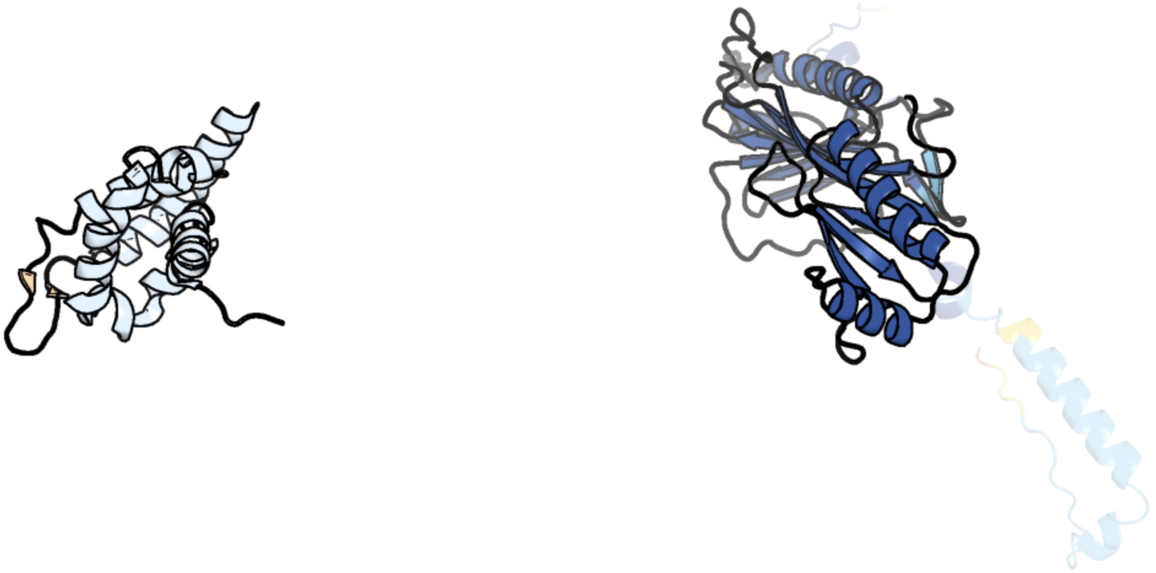

Figure 379: left: reference structure of 8cen chain O. right: predicted structure of chlorv-1..379, unaligned sequences are shown as transparent

chlorv-1..380

- Sequence-based annotation for chlorv-1..380 is putative Thymidine kinase
- Best hit was 4uxj chain E: THYMIDINE KINASE

target	prob	fident	alnlen	evalue	theader
4uxj-assembly2.cif.gz_E-3	1	0.3	180	1.51e-17	Leishmania major Thymidine Kinase in complex with dTTP
4uxi-assembly1.cif.gz_B-2	1	0.282	177	2.067e-17	Leishmania major Thymidine Kinase in complex with thymidine
2wvj-assembly1.cif.gz_D	1	0.308	178	2.83e-17	Mutation of Thr163 to Ser in Human Thymidine Kinase Shifts the Specificity from Thymidine towards the Nucleoside Analogue Azidothymidine

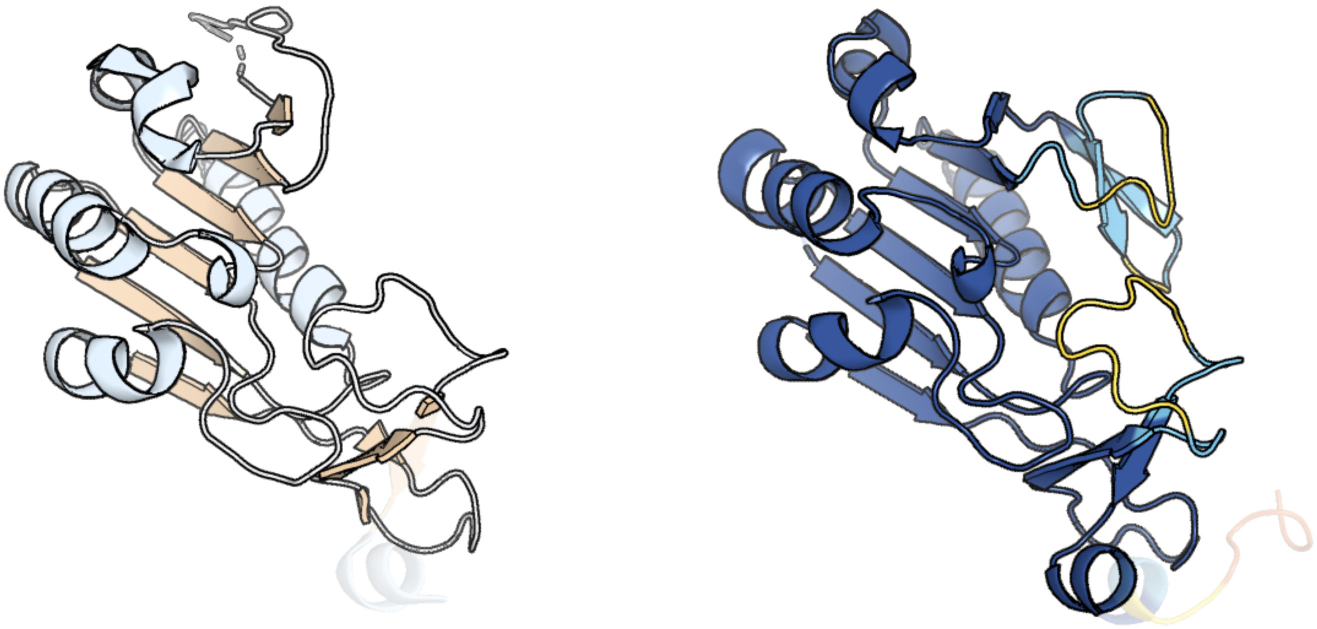

Figure 380: left: reference structure of 4uxj chain E. right: predicted structure of chlorv-1..380, unaligned sequences are shown as transparent

chlorv-1..381

- Sequence-based annotation for chlorv-1..381 is hypothetical protein
- Best hit was 1kcf chain B: HYPOTHETICAL 30.2 KD PROTEIN C25G10.02 IN CHROMOSOME I

target	prob	fident	alnlen	evalue	theader
1kcf-assembly1.cif.gz_B	1	0.172	284	6.772e-06	Crystal Structure of the Yeast Mitochondrial Holliday Junction Resolvase, Ydc2
1kcf-assembly1.cif.gz_A	1	0.164	291	0.0001282	Crystal Structure of the Yeast Mitochondrial Holliday Junction Resolvase, Ydc2
6p7a-assembly2.cif.gz_B-2	1	0.147	285	0.0002596	CRYSTAL STRUCTURE OF THE FOWLPOX VIRUS HOLLIDAY JUNCTION RESOLVASE

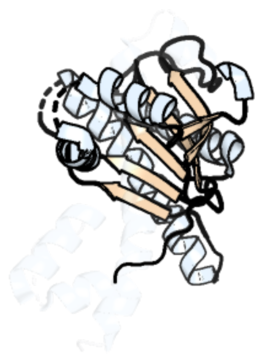

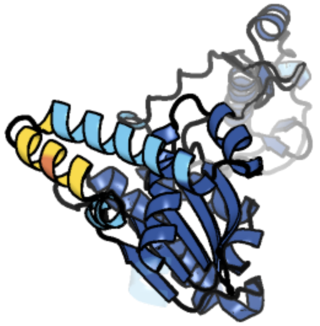

Figure 381: left: reference structure of 1kcf chain B. right: predicted structure of chlorv-1..381, unaligned sequences are shown as transparent

chlorv-1..382

- Sequence-based annotation for chlorv-1..382 is hypothetical protein
- No significant structural hit found

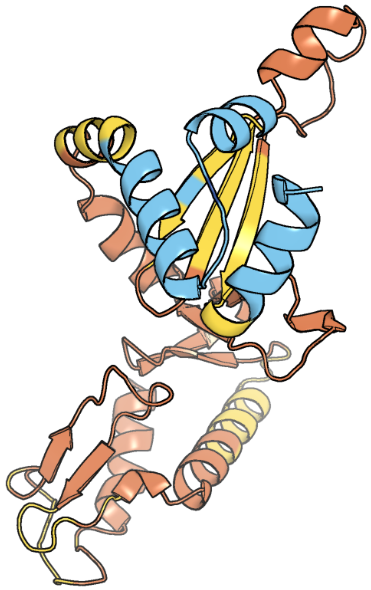

Figure 382: predicted structure of chlorv-1..382

chlorv-1..383

- Sequence-based annotation for chlorv-1..383 is hypothetical protein
- Best hit was 8ppl chain Ir: Eukaryotic translation initiation factor 2 subunit 1

target	prob	fident	alnlen	evalue	theader
8ppl-assembly1.cif.gz_Ir	1	0.179	273	4.699e-13	MERS-CoV Nsp1 bound to the human 43S pre-initiation complex
6ybv-assembly1.cif.gz_r	1	0.181	276	6.343e-13	Structure of a human 48S translational initiation complex - eIF2-TC
1q8k-assembly1.cif.gz_A	1	0.198	272	2.614e-11	Solution structure of alpha subunit of human eIF2

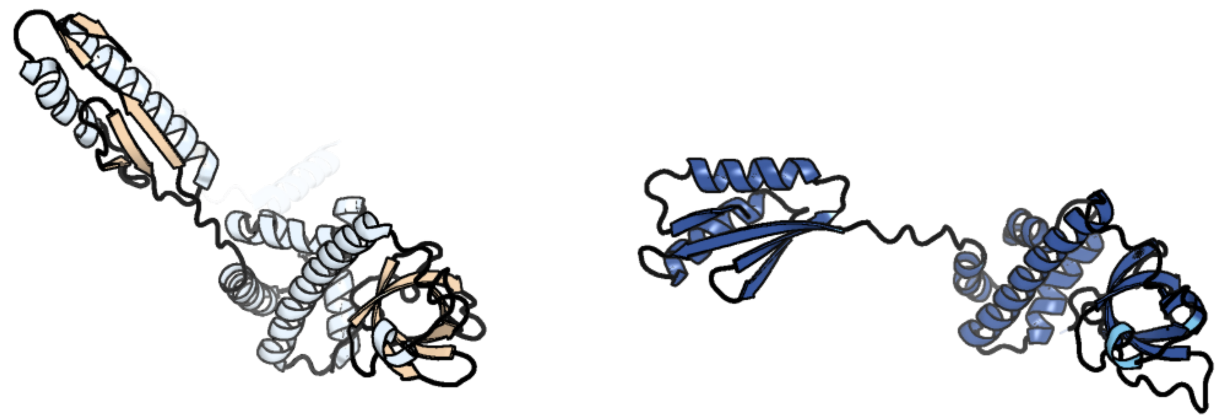

Figure 383: left: reference structure of 8ppl chain Ir. right: predicted structure of chlorv-1..383, unaligned sequences are shown as transparent

chlorv-1..384

- Sequence-based annotation for chlorv-1..384 is putative Poxvirus Late Transcription Factor VLTF2
- No significant structural hit found

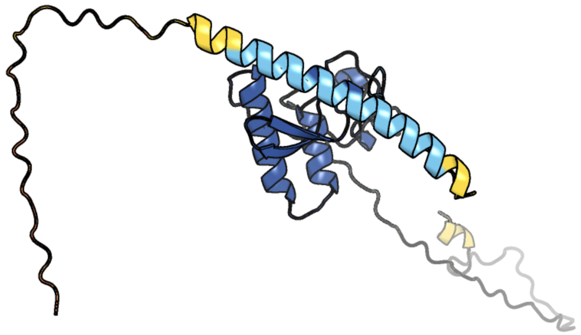

Figure 384: predicted structure of chlorv-1..384

chlorv-1..385

- Sequence-based annotation for chlorv-1..385 is hypothetical protein
- No significant structural hit found

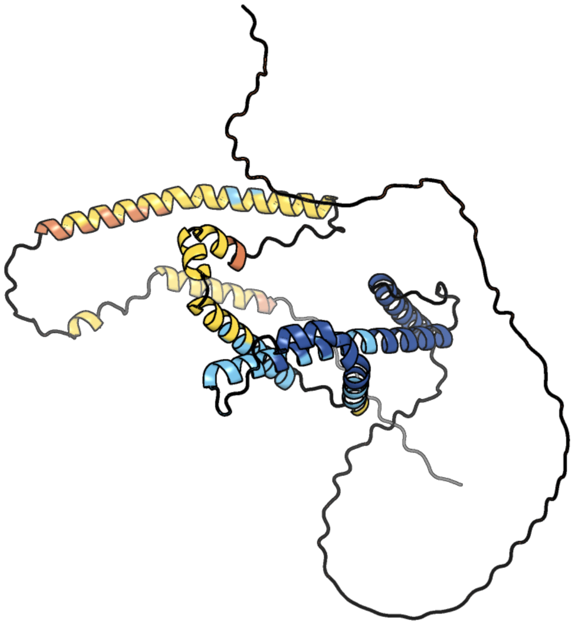

Figure 385: predicted structure of chlorv-1..385

chlorv-1..386

- Sequence-based annotation for chlorv-1..386 is hypothetical protein
- No significant structural hit found

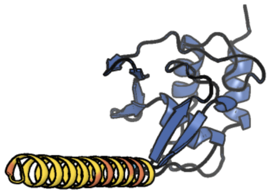

Figure 386: predicted structure of chlorv-1..386

chlorv-1..387

- Sequence-based annotation for chlorv-1..387 is hypothetical protein
- No significant structural hit found

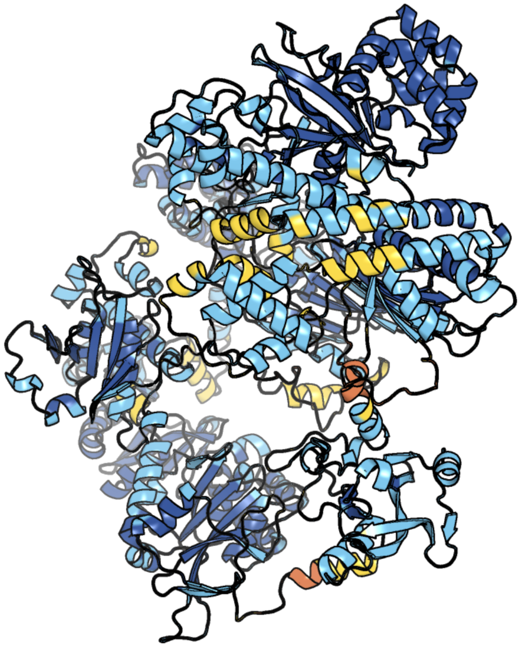

Figure 387: predicted structure of chlorv-1..387

chlorv-1..388

- Sequence-based annotation for chlorv-1..388 is hypothetical protein
- No significant structural hit found

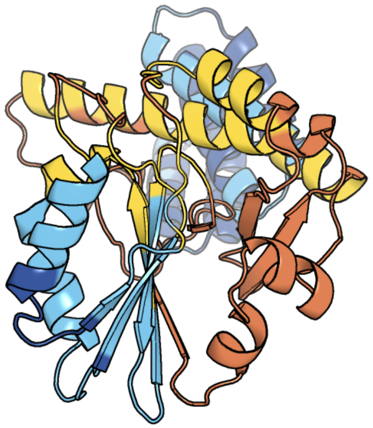

Figure 388: predicted structure of chlorv-1..388

chlorv-1..389

- Sequence-based annotation for chlorv-1..389 is hypothetical protein
- No significant structural hit found

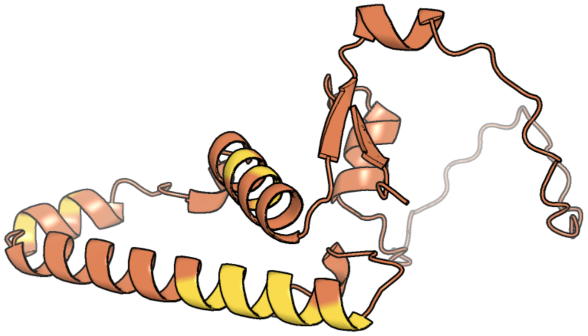

Figure 389: predicted structure of chlorv-1..389

chlorv-1..390

- Sequence-based annotation for chlorv-1..390 is hypothetical protein
- No significant structural hit found

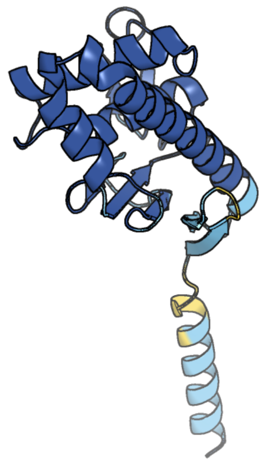

Figure 390: predicted structure of chlorv-1..390

chlorv-1..391

- Sequence-based annotation for chlorv-1..391 is hypothetical protein
- No significant structural hit found

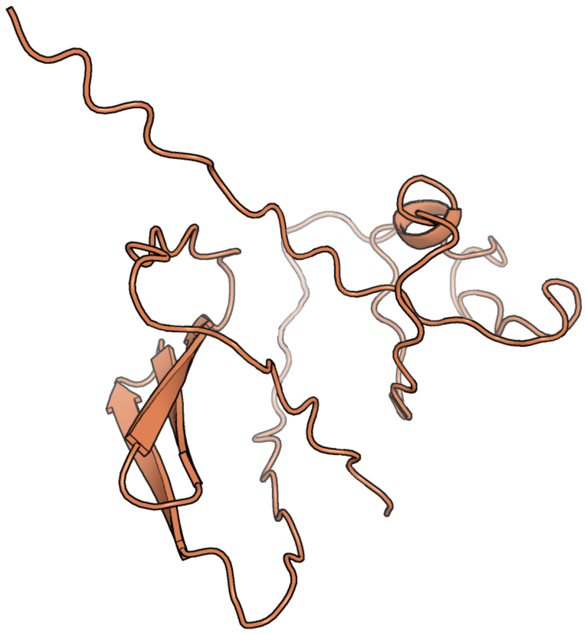

Figure 391: predicted structure of chlorv-1..391

chlorv-1..392

- Sequence-based annotation for chlorv-1..392 is putative Methyltransferase
- Best hit was 4n48 chain B: Cap-specific mRNA (nucleoside-2'-O-)-methyltransferase 1

target	prob	fident	alnlen	evalue	theader
4n48-assembly1.cif.gz_B	1	0.133	412	1.485e-13	Cap-specific mRNA (nucleoside-2'-O-)-methyltransferase 1 Protein in complex with capped RNA fragment
4n48-assembly2.cif.gz_A	1	0.142	406	1.839e-13	Cap-specific mRNA (nucleoside-2'-O-)-methyltransferase 1 Protein in complex with capped RNA fragment
4n49-assembly1.cif.gz_A	1	0.141	397	2.047e-13	Cap-specific mRNA (nucleoside-2'-O-)-methyltransferase 1 Protein in complex with m7GpppG and SAM

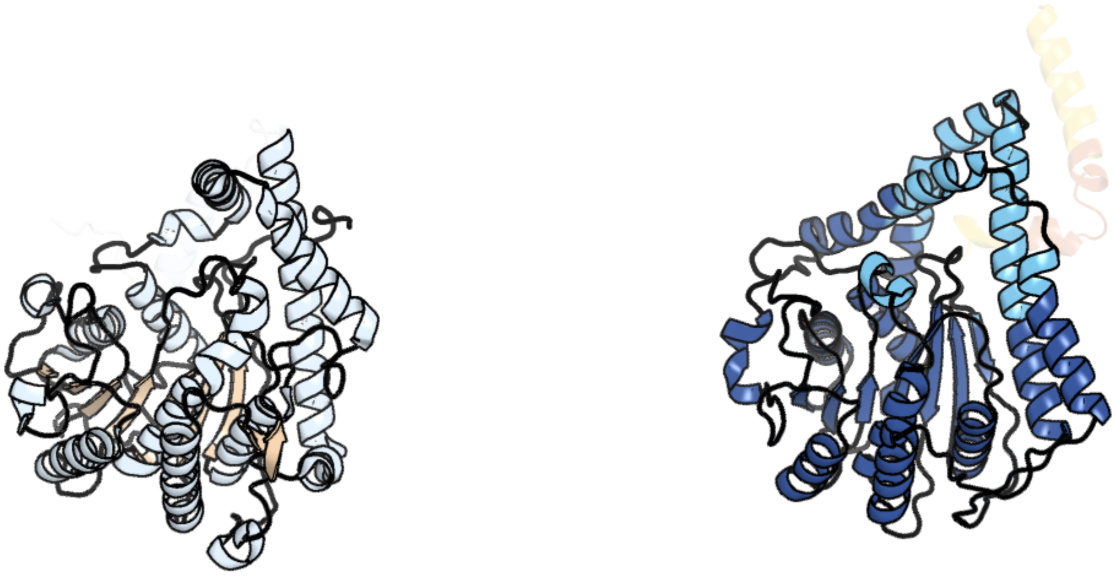

Figure 392: left: reference structure of 4n48 chain B. right: predicted structure of chlorv-1..392, unaligned sequences are shown as transparent

chlorv-1..393

- Sequence-based annotation for chlorv-1..393 is hypothetical protein
- No significant structural hit found

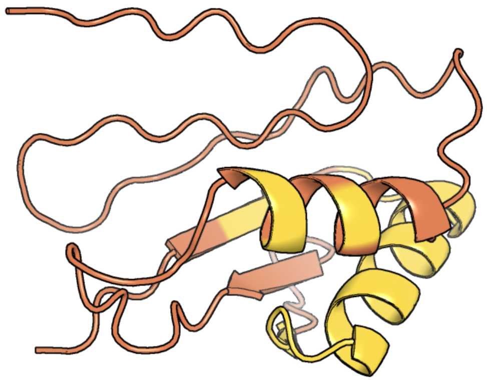

Figure 393: predicted structure of chlorv-1..393

chlorv-1..394

- Sequence-based annotation for chlorv-1..394 is putative Oxidoreductase/proline dehydrogenase
- Best hit was 2g37 chain A: proline dehydrogenase/delta-1-pyrroline-5-carboxylate dehydrogenase

target	prob	fident	alnlen	evalue	theader
2g37-assembly1.cif.gz_A	1	0.167	257	8.573e-10	Structure of Thermus thermophilus L-proline dehydrogenase
5ur2-assembly1.cif.gz_A	1	0.157	285	2.883e-09	Crystal structure of proline utilization A (PutA) from Bdellovibrio bacteriovorus inactivated by N-propargylglycine
5ur2-assembly2.cif.gz_D	1	0.164	285	3.255e-09	Crystal structure of proline utilization A (PutA) from Bdellovibrio bacteriovorus inactivated by N-propargylglycine

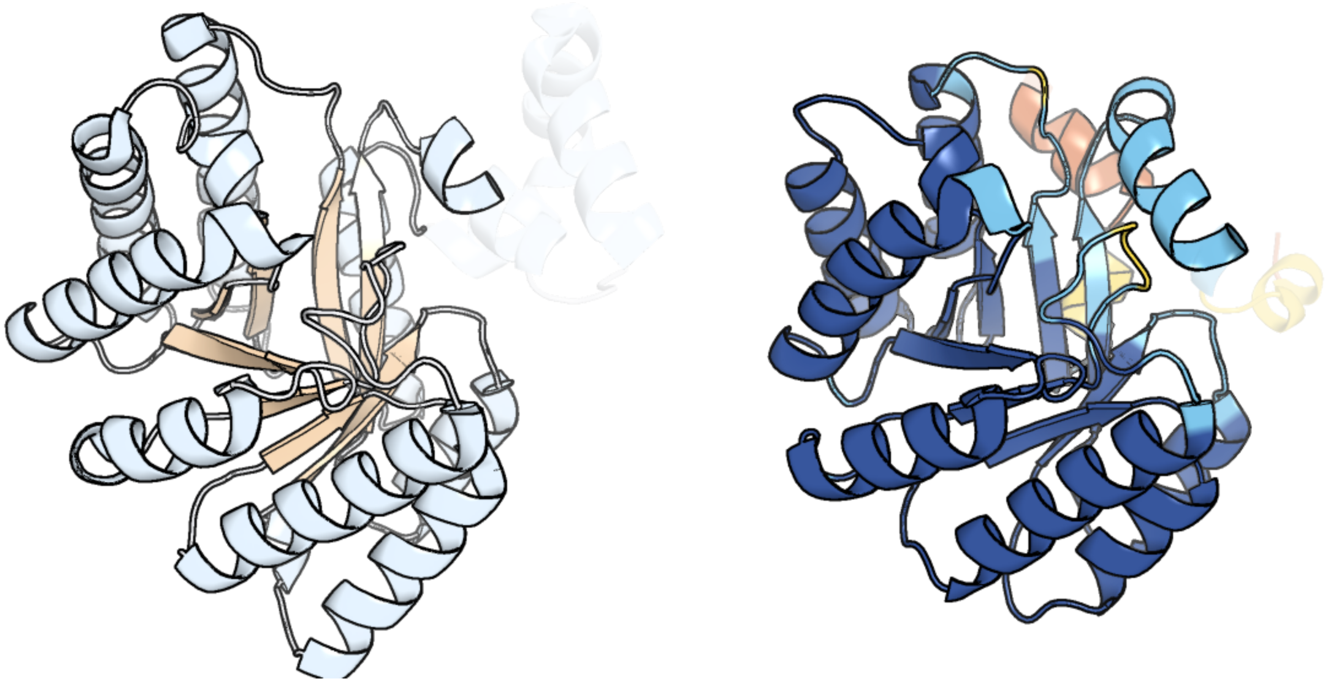

Figure 394: left: reference structure of 2g37 chain A. right: predicted structure of chlorv-1..394, unaligned sequences are shown as transparent

chlorv-1..395

- Sequence-based annotation for chlorv-1..395 is hypothetical protein
- No significant structural hit found

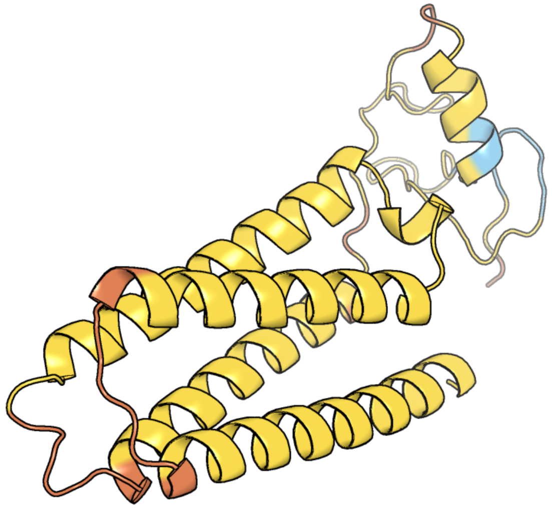

Figure 395: predicted structure of chlorv-1..395

chlorv-1..396

- Sequence-based annotation for chlorv-1..396 is hypothetical protein
- No significant structural hit found

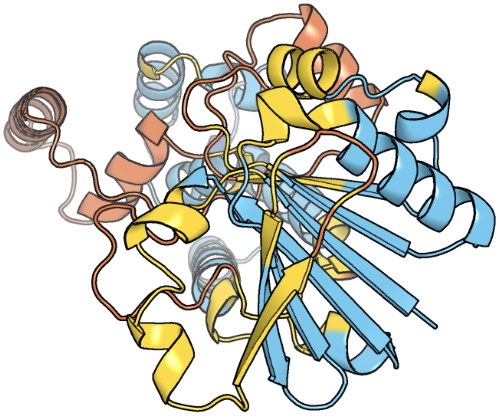

Figure 396: predicted structure of chlorv-1..396

chlorv-1..397

- Sequence-based annotation for chlorv-1..397 is putative Glycosidase
- Best hit was 4ac1 chain X: ENDO-N-ACETYL-BETA-D-GLUCOSAMINIDASE

target	prob	fident	alnlen	evalue	theader
4ac1-assembly1.cif.gz_X	1	0.273	285	8.874e-21	The structure of a fungal endo-beta-N-acetylglucosaminidase from glycosyl hydrolase family 18, at 1.3A resolution
2y8v-assembly2.cif.gz_D	1	0.27	281	4.763e-18	Structure of chitinase, ChiC, from Aspergillus fumigatus.
6k7z-assembly2.cif.gz_B	1	0.18	293	6.603e-12	Crystal structure of a GH18 chitinase from Pseudoalteromonas aurantia

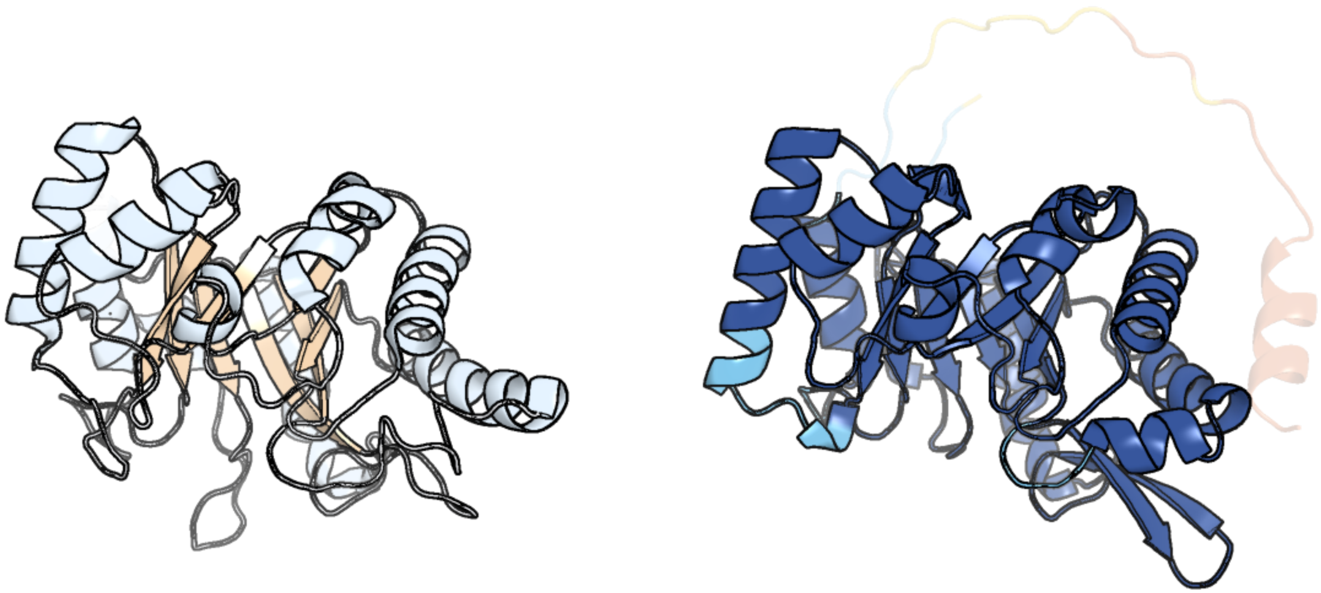

Figure 397: left: reference structure of 4ac1 chain X. right: predicted structure of chlorv-1..397, unaligned sequences are shown as transparent

chlorv-1..398

- Sequence-based annotation for chlorv-1..398 is putative NUDIX hydrolase
- Best hit was 2qjt chain A: Nicotinamide-nucleotide adenylyltransferase

target	prob	fident	alnlen	evalue	theader
2qjt-assembly1.cif.gz_A	1	0.133	187	3.246e-07	Crystal structure of a bifunctional NMN adenylyltransferase/ADP ribose pyrophosphatase complexed with AMP and MN ion from Francisella tularensis
2r5w-assembly1.cif.gz_A	1	0.162	160	8.197e-07	Crystal structure of a bifunctional NMN adenylyltransferase/ADP ribose pyrophosphatase from Francisella tularensis
3gz8-assembly2.cif.gz_D	1	0.145	165	3.189e-06	Cocrystal structure of NUDIX domain of Shewanella oneidensis NrtR complexed with ADP ribose

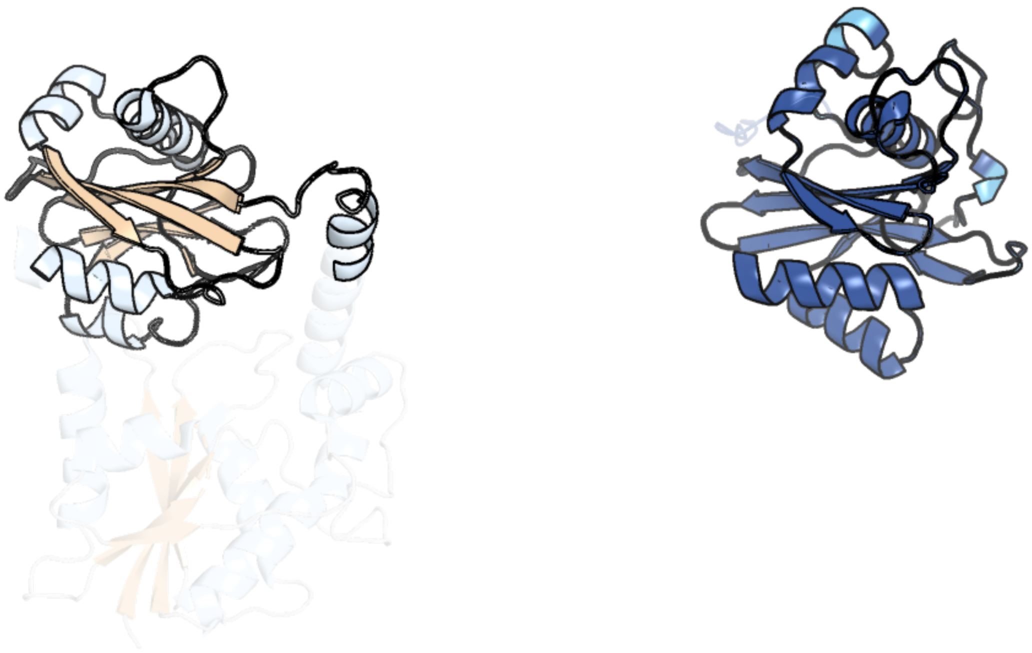

Figure 398: left: reference structure of 2qjt chain A. right: predicted structure of chlorv-1..398, unaligned sequences are shown as transparent

chlorv-1..399

- Sequence-based annotation for chlorv-1..399 is hypothetical protein
- No significant structural hit found

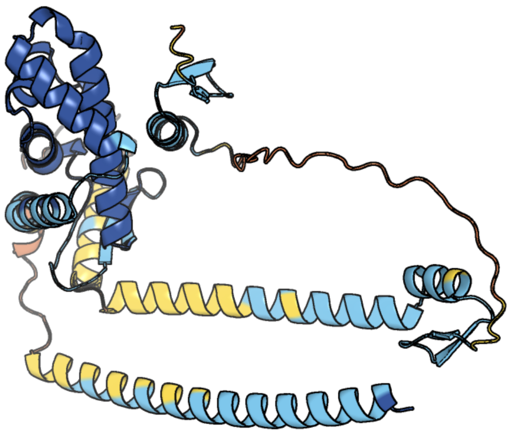

Figure 399: predicted structure of chlorv-1..399

chlorv-1..400

- Sequence-based annotation for chlorv-1..400 is putative Hydrolase
- Best hit was 6nkf chain B: Lip_vut4, C3L

target	prob	fident	alnlen	evalue	theader
6nkf-assembly2.cif.gz_B	1	0.156	351	4.464e-11	Crystal Structure of the Lipase Lip_vut4 from Goat Rumen metagenome.
5aoa-assembly1.cif.gz_A	1	0.16	343	1.354e-10	The structure of a novel thermophilic esterase from the Planctomycetes species, Thermogutta terrifontis, Est2-Propionate bound
5aob-assembly1.cif.gz_A	1	0.153	345	1.599e-10	The structure of a novel thermophilic esterase from the Planctomycetes species, Thermogutta terrifontis, Est2-butyrate bound

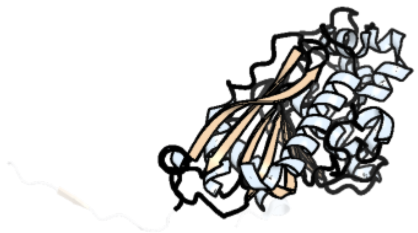

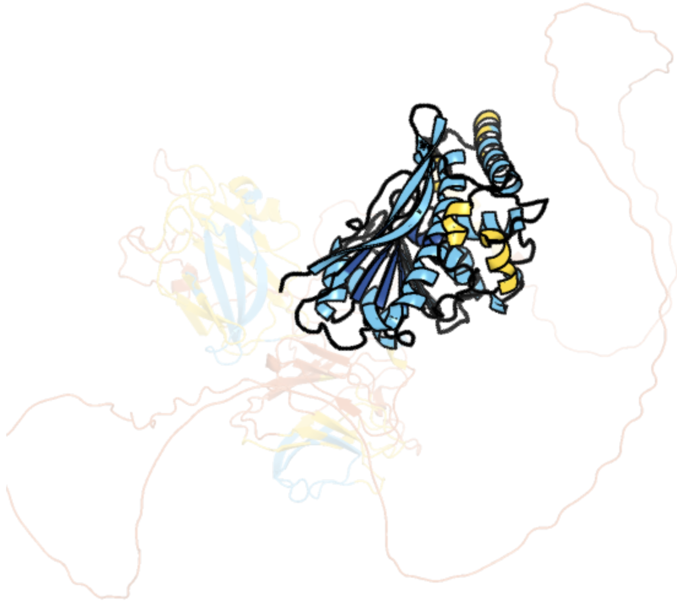

Figure 400: left: reference structure of 6nkf chain B. right: predicted structure of chlorv-1..400, unaligned sequences are shown as transparent

chlorv-1..401

- Sequence-based annotation for chlorv-1..401 is putative Cytidine deaminase
- Best hit was 1tiy chain A: Guanine deaminase

target	prob	fident	alnlen	evalue	theader
1tiy-assembly1.cif.gz_A	1	0.461	156	9.332e-22	X-RAY STRUCTURE OF GUANINE DEAMINASE FROM BACILLUS SUBTILIS NORTHEAST STRUCTURAL GENOMICS CONSORTIUM TARGET SR160
7dbf-assembly1.cif.gz_D	1	0.401	137	1.761e-15	The structure of the Arabidopsis thaliana guanosine deaminase
7w1q-assembly1.cif.gz_D	1	0.367	155	3.407e-15	The structure of the Arabidopsis thaliana guanosine deaminase mutant E82Q complexed with 2'-O-methylguanosine

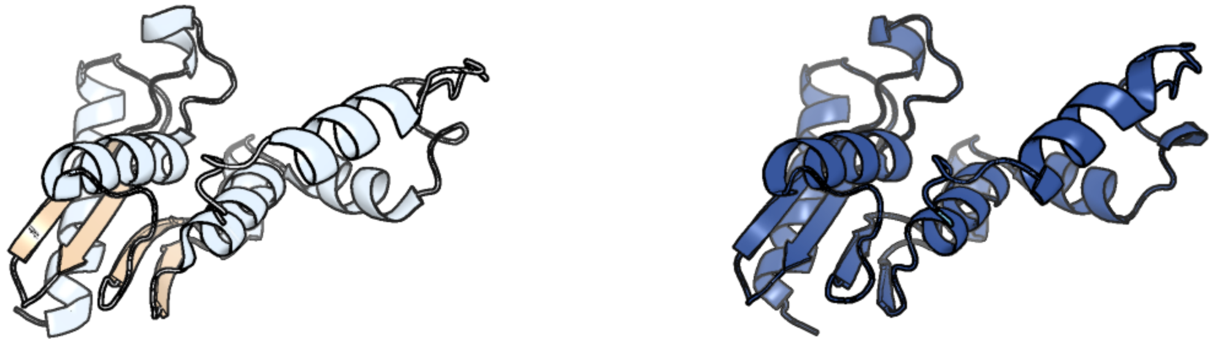

Figure 401: left: reference structure of 1tiy chain A. right: predicted structure of chlorv-1..401, unaligned sequences are shown as transparent

chlorv-1..402

- Sequence-based annotation for chlorv-1..402 is hypothetical protein
- No significant structural hit found

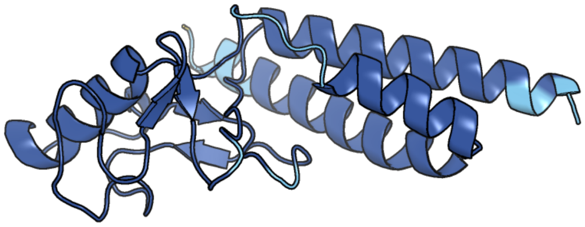

Figure 402: predicted structure of chlorv-1..402

chlorv-2..092

- Sequence-based annotation for chlorv-2..092 is
- No significant structural hit found

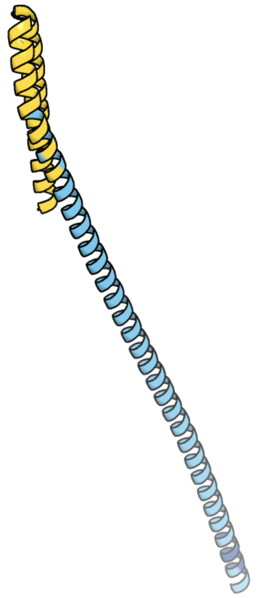

Figure 403: predicted structure of chlorv-2..092

chlorv-2..093

- Sequence-based annotation for chlorv-2..093 is
- No significant structural hit found

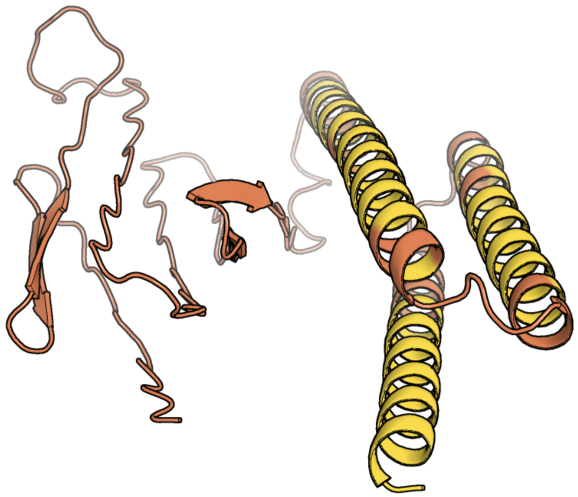

Figure 404: predicted structure of chlorv-2..093

chlorv-2..179

- Sequence-based annotation for chlorv-2..179 is
- No significant structural hit found

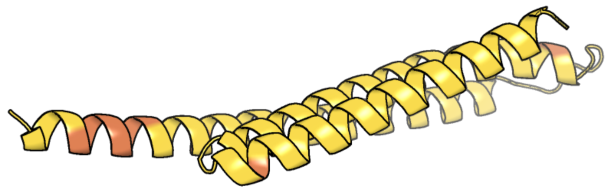

Figure 405: predicted structure of chlorv-2..179

chlorv-2..225

- Sequence-based annotation for chlorv-2..225 is
- No significant structural hit found

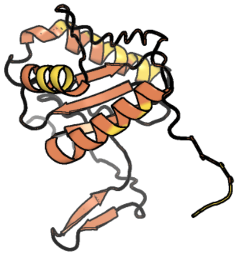

Figure 406: predicted structure of chlorv-2..225

chlorv-2..241

- Sequence-based annotation for chlorv-2..241 is
- No significant structural hit found

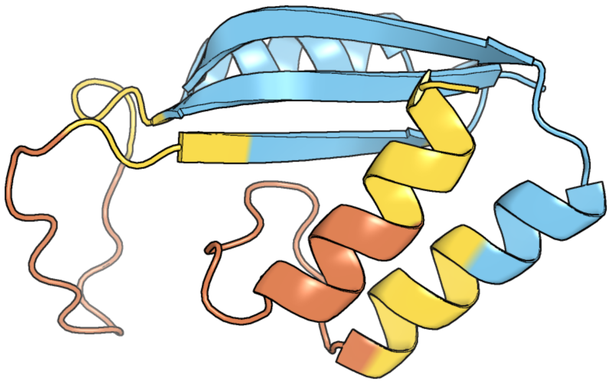

Figure 407: predicted structure of chlorv-2..241

chlorv-3..004

- Sequence-based annotation for chlorv-3..004 is
- Best hit was 8ftm chain B: 5'-3' RNA helicase-like protein

target	prob	fident	alnlen	evalue	theader
8ftm-assembly2.cif.gz_B	1	0.167	691	6.478e-20	Setx-ssRNA-ADP-SO4 complex
8ftm-assembly1.cif.gz_A	1	0.167	670	8.287e-20	Setx-ssRNA-ADP-SO4 complex
5ean-assembly1.cif.gz_A-2	1	0.174	584	2.516e-17	Crystal structure of Dna2 in complex with a 5' overhang DNA

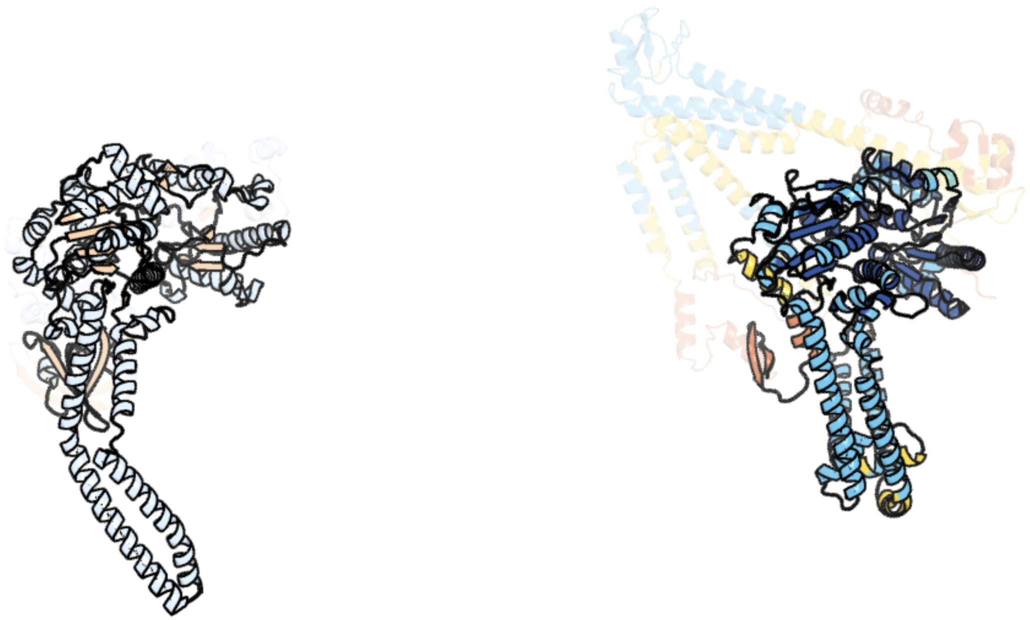

Figure 408: left: reference structure of 8ftm chain B. right: predicted structure of chlorv-3..004, unaligned sequences are shown as transparent

chlorv-3..006

- Sequence-based annotation for chlorv-3..006 is
- Best hit was 2ip2 chain B: Probable phenazine-specific methyltransferase

target	prob	fident	alnlen	evalue	theader
2ip2-assembly1.cif.gz_B	1	0.084	237	1.325e-05	Structure of the Pyocyanin Biosynthetic Protein PhzM
3e05-assembly1.cif.gz_G	1	0.118	227	1.325e-05	CRYSTAL STRUCTURE OF Precorrin-6y C5,15-methyltransferase FROM Geobacter metallireducens GS-15
3dtn-assembly1.cif.gz_A	1	0.094	244	1.41e-05	Crystal structure of putative Methyltransferase-MM_2633 from Methanosarcina mazei .

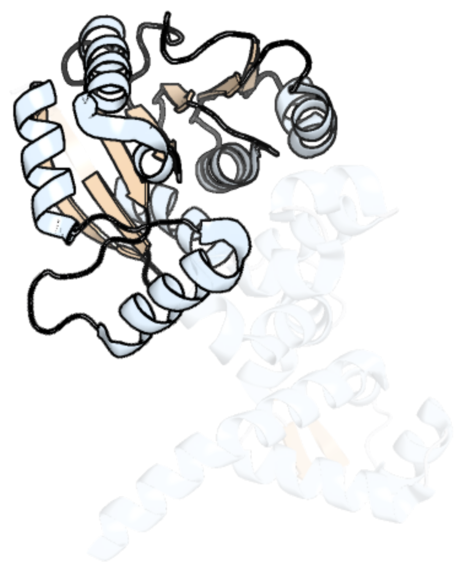

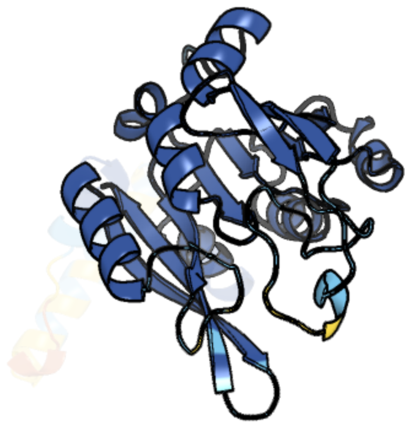

Figure 409: left: reference structure of 2ip2 chain B. right: predicted structure of chlorv-3..006, unaligned sequences are shown as transparent

chlorv-3..007

- Sequence-based annotation for chlorv-3..007 is
- No significant structural hit found

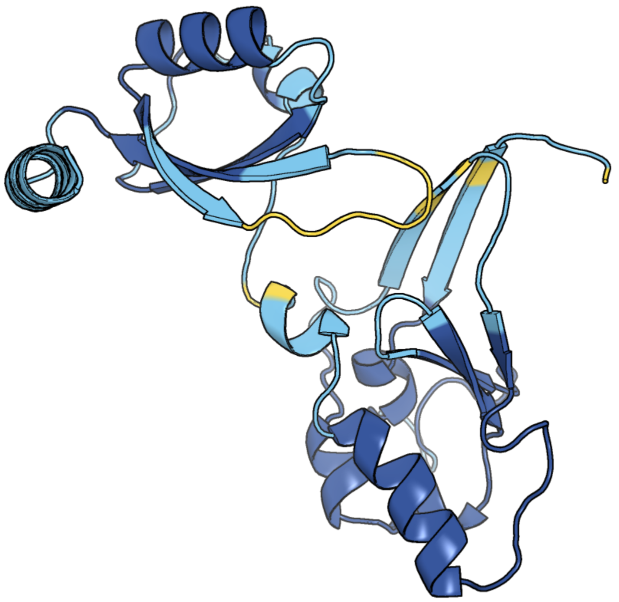

Figure 410: predicted structure of chlorv-3..007

chlorv-3..031

- Sequence-based annotation for chlorv-3..031 is
- No significant structural hit found

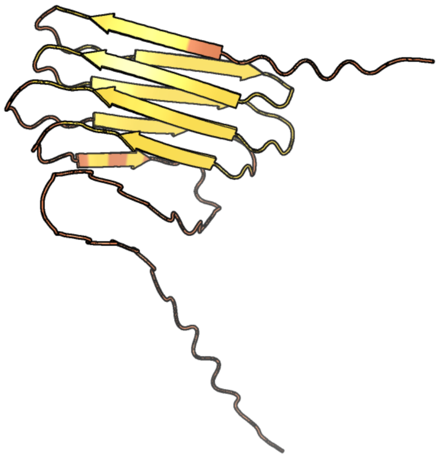

Figure 411: predicted structure of chlorv-3..031

chlorv-3..032

- Sequence-based annotation for chlorv-3..032 is
- No significant structural hit found

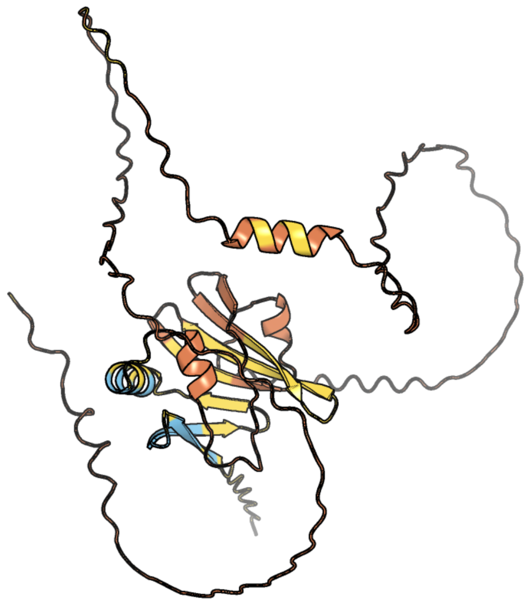

Figure 412: predicted structure of chlorv-3..032

chlorv-3..079

- Sequence-based annotation for chlorv-3..079 is
- No significant structural hit found

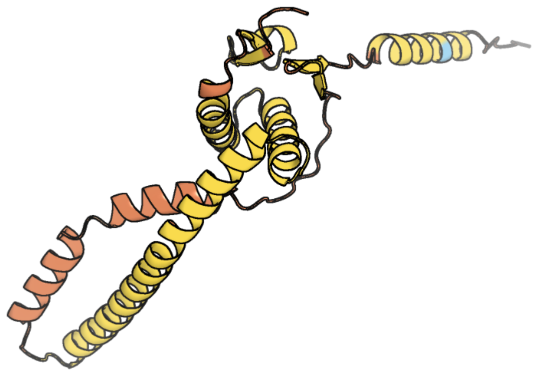

Figure 413: predicted structure of chlorv-3..079

chlorv-3..080

- Sequence-based annotation for chlorv-3..080 is
- No significant structural hit found

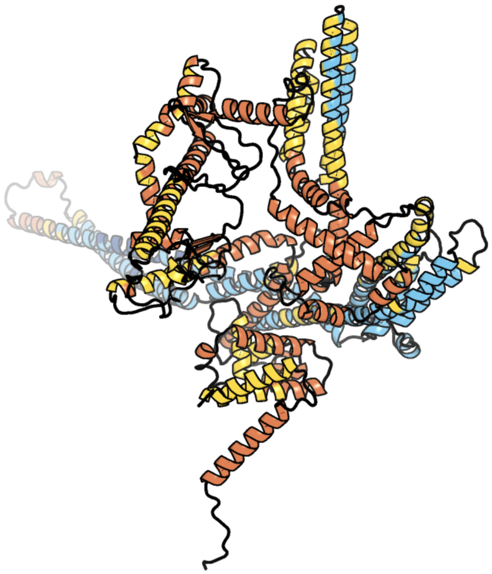

Figure 414: predicted structure of chlorv-3..080

chlorv-3..095

- Sequence-based annotation for chlorv-3..095 is
- No significant structural hit found

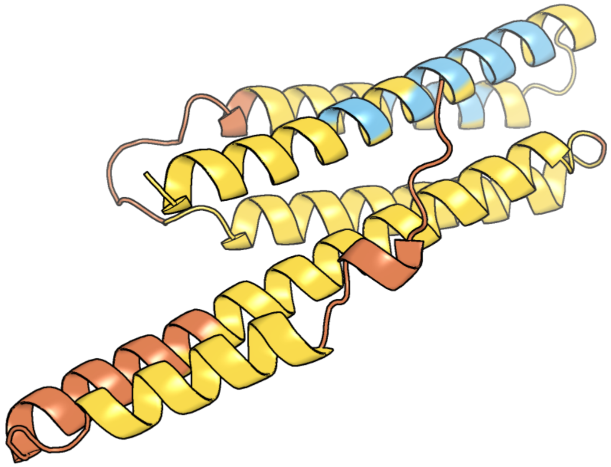

Figure 415: predicted structure of chlorv-3..095

chlorv-3..096

- Sequence-based annotation for chlorv-3..096 is
- No significant structural hit found

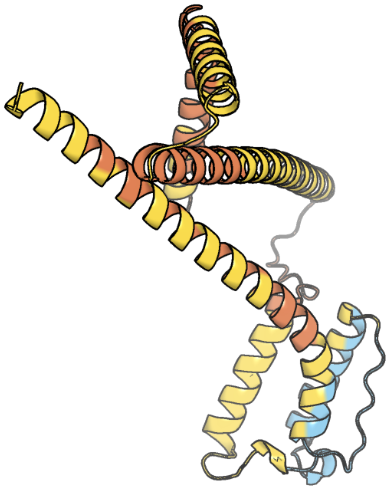

Figure 416: predicted structure of chlorv-3..096

chlorv-3..102

- Sequence-based annotation for chlorv-3..102 is
- Best hit was 7eew chain A: Type I restriction-modification system methyltransferase subunit

target	prob	fident	alnlen	evalue	theader
7eew-assembly1.cif.gz__A-2	1	0.171	826	4.718e-25	Crystal structure of the intact MTase from Vibrio vulnificus YJ016 in complex with the DNA-mimicking Ocr protein and the S-adenosyl-L-homocysteine (SAH)
1ydx-assembly1.cif.gz__A	1	0.217	377	3.143e-19	Crystal structure of Type-I restriction-modification system S subunit from M. genitalium
2okc-assembly1.cif.gz__A	1	0.235	416	3.341e-17	Crystal structure of Type I restriction enzyme StySJI M protein (NP__813429.1) from Bacteroides thetaiotaomicron VPI-5482 at 2.20 A resolution

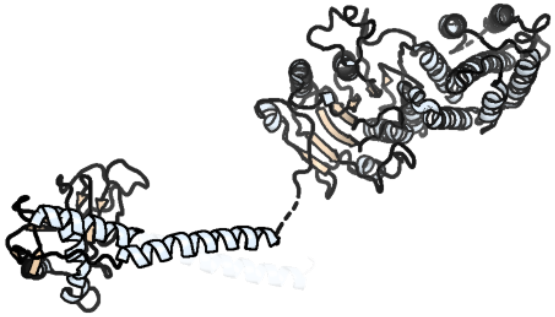

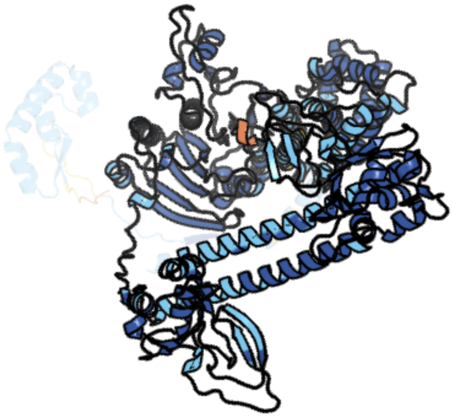

Figure 417: left: reference structure of 7eew chain A. right: predicted structure of chlorv-3..102, unaligned sequences are shown as transparent

chlorv-3..103

- Sequence-based annotation for chlorv-3..103 is
- Best hit was 4xqk chain B: LlaBIII

target	prob	fident	alnlen	evalue	theader
4xqk-assembly2.cif.gz_B	1	0.174	424	3.565e-10	ATP-dependent Type ISP restriction-modification enzyme LlaBIII bound to DNA
4xqk-assembly1.cif.gz_A	1	0.15	513	1.235e-09	ATP-dependent Type ISP restriction-modification enzyme LlaBIII bound to DNA
3h1t-assembly1.cif.gz_A	1	0.146	246	1.751e-05	The fragment structure of a putative HsdR subunit of a type I restriction enzyme from Vibrio vulnificus YJ016

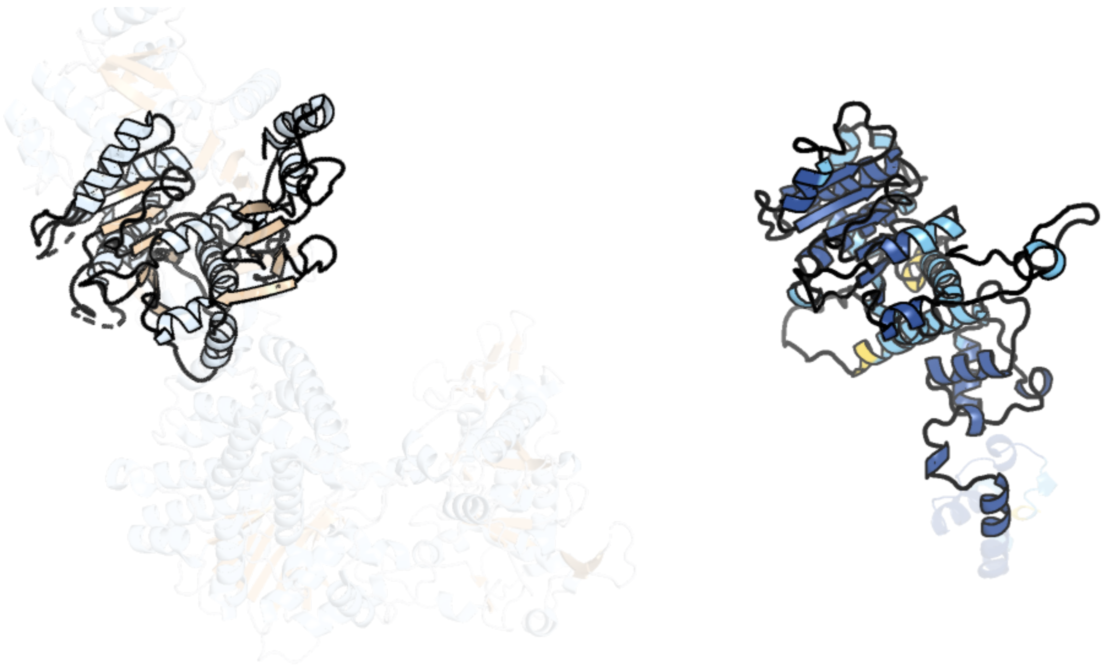

Figure 418: left: reference structure of 4xqk chain B. right: predicted structure of chlorv-3..103, unaligned sequences are shown as transparent

chlorv-3..104

- Sequence-based annotation for chlorv-3..104 is
- No significant structural hit found

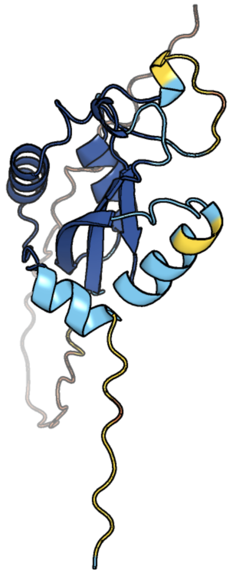

Figure 419: predicted structure of chlorv-3..104

chlorv-3..114

- Sequence-based annotation for chlorv-3..114 is
- No significant structural hit found

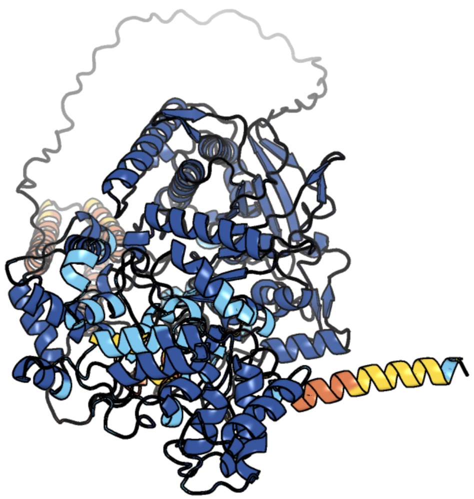

Figure 420: predicted structure of chlorv-3..114

chlorv-3..120

- Sequence-based annotation for chlorv-3..120 is
- No significant structural hit found

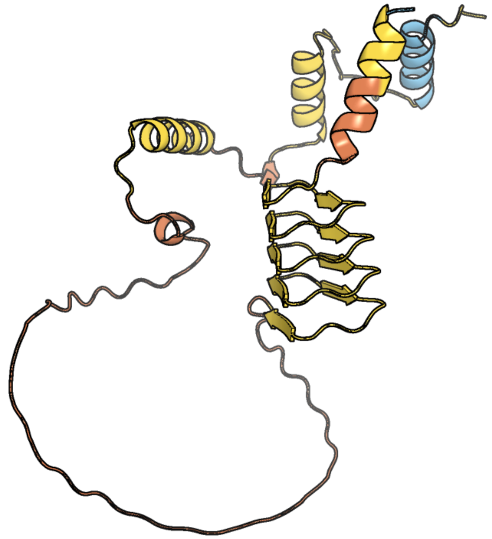

Figure 421: predicted structure of chlorv-3..120

chlorv-3..133

- Sequence-based annotation for chlorv-3..133 is
- Best hit was 7k6p chain K: Histone-lysine N-methyltransferase, H3 lysine-79 specific

target	prob	fident	alnlen	evalue	theader
7k6p-assembly1.cif.gz_K	1	0.186	193	8.781e-09	Active state Dot1 bound to the unacetylated H4 nucleosome
5fa8-assembly1.cif.gz_A	1	0.215	153	2.793e-08	SAM complex with aKMT from the hyperthermophilic archaeon Sulfolobus islandicu
1u2z-assembly2.cif.gz_B	1	0.194	190	4.107e-08	Crystal structure of histone K79 methyltransferase Dot1p from yeast

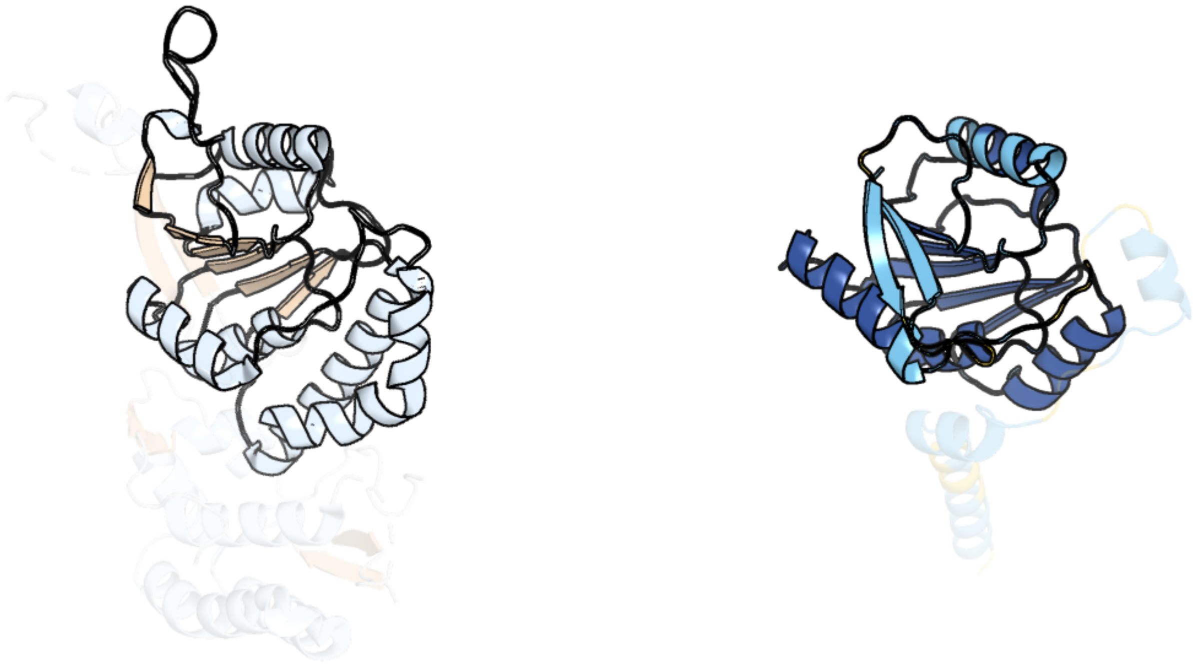

Figure 422: left: reference structure of 7k6p chain K. right: predicted structure of chlorv-3..133, unaligned sequences are shown as transparent

chlorv-3..150

- Sequence-based annotation for chlorv-3..150 is
- No significant structural hit found

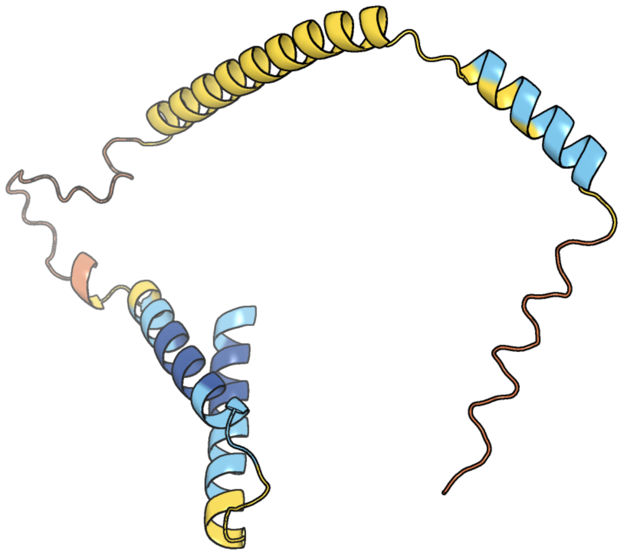

Figure 423: predicted structure of chlorv-3..150

chlorv-3..151

- Sequence-based annotation for chlorv-3..151 is
- No significant structural hit found

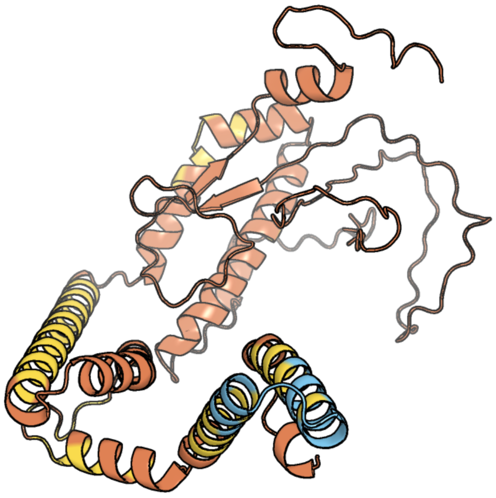

Figure 424: predicted structure of chlorv-3..151

chlorv-3..152

- Sequence-based annotation for chlorv-3..152 is
- No significant structural hit found

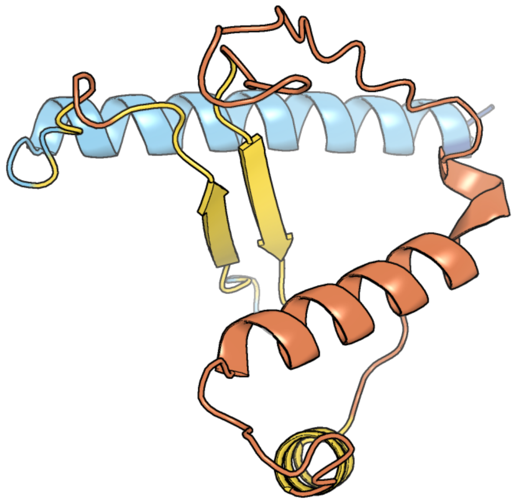

Figure 425: predicted structure of chlorv-3..152

chlorv-3..153

- Sequence-based annotation for chlorv-3..153 is
- No significant structural hit found

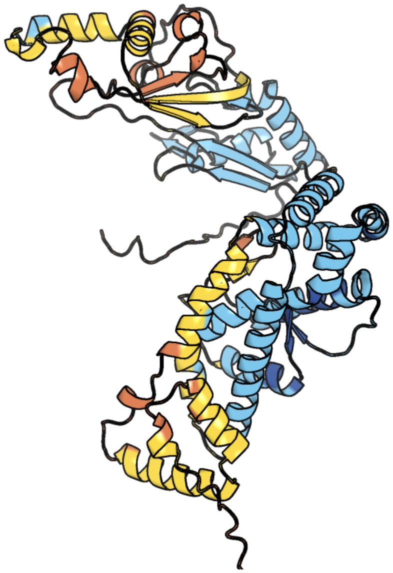

Figure 426: predicted structure of chlorv-3..153

chlorv-3..196

- Sequence-based annotation for chlorv-3..196 is
- No significant structural hit found

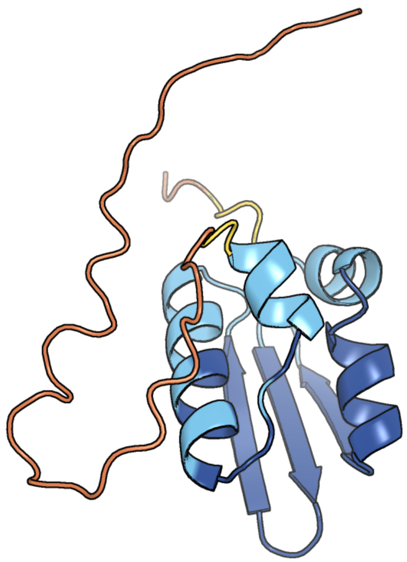

Figure 427: predicted structure of chlorv-3..196

chlorv-3..205

- Sequence-based annotation for chlorv-3..205 is
- No significant structural hit found

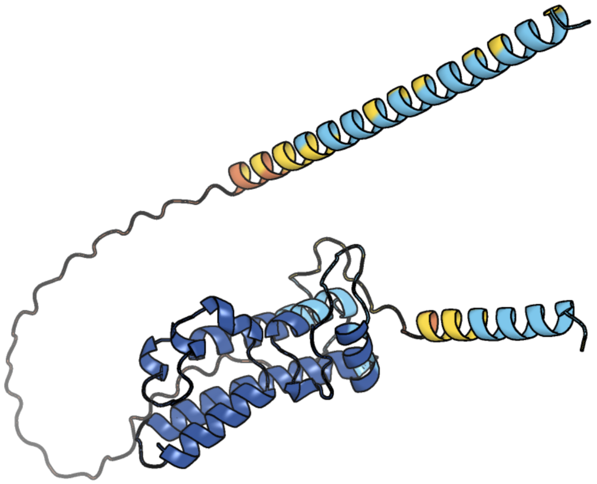

Figure 428: predicted structure of chlorv-3..205

chlorv-3..219

- Sequence-based annotation for chlorv-3..219 is
- No significant structural hit found

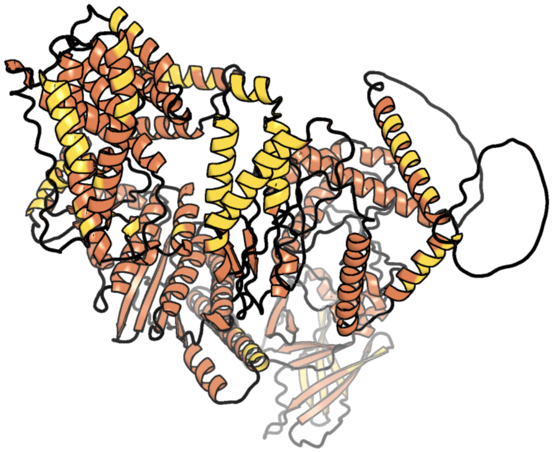

Figure 429: predicted structure of chlorv-3..219

chlorv-3..225

- Sequence-based annotation for chlorv-3..225 is
- No significant structural hit found

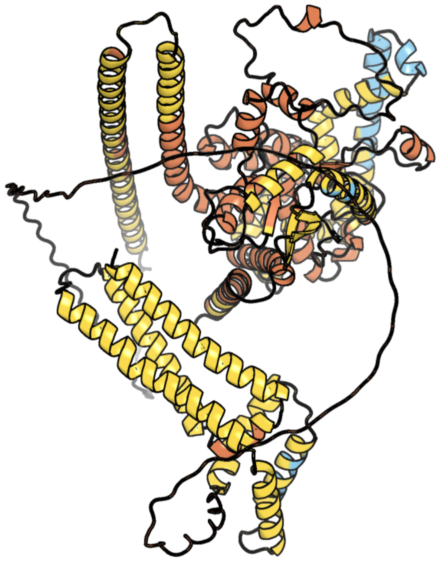

Figure 430: predicted structure of chlorv-3..225

chlorv-3..329

- Sequence-based annotation for chlorv-3..329 is
- No significant structural hit found

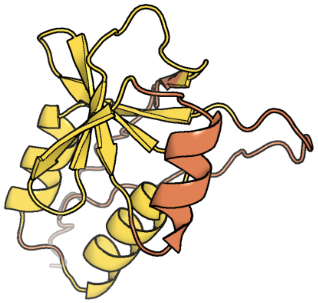

Figure 431: predicted structure of chlorv-3..329

chlorv-3..350

- Sequence-based annotation for chlorv-3..350 is
- No significant structural hit found

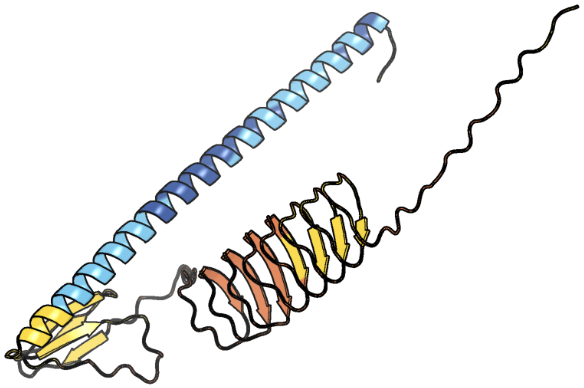

Figure 432: predicted structure of chlorv-3..350

chlorv-3..384

- Sequence-based annotation for chlorv-3..384 is
- No significant structural hit found

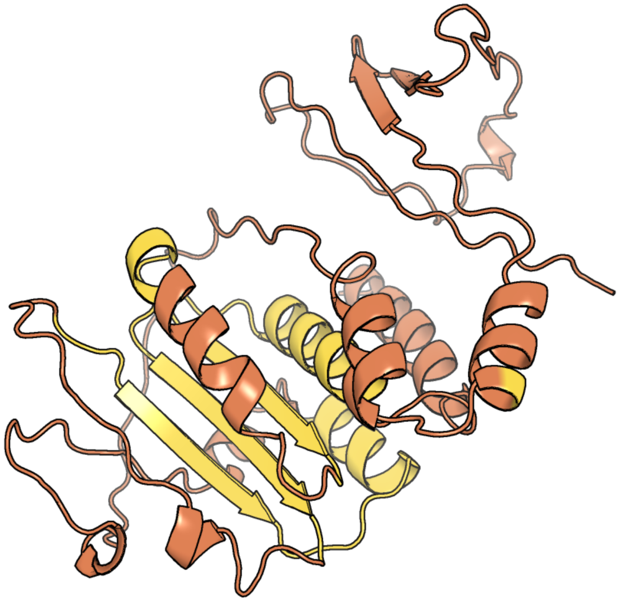

Figure 433: predicted structure of chlorv-3..384

chlorv-3..391

- Sequence-based annotation for chlorv-3..391 is
- Best hit was 6a0w chain A: Lipase

target	prob	fident	alnlen	evalue	theader
6a0w-assembly1.cif.gz_A	1	0.204	171	3.713e-08	Crystal structure of lipase from Rhizopus microsporus var. chinensis
6qpr-assembly1.cif.gz_A	1	0.208	187	4.175e-08	Rhizomucor miehei lipase propeptide complex, Ser95/Ile96 deletion mutant
5ap9-assembly1.cif.gz_A	1	0.201	258	6.672e-08	Controlled lid-opening in Thermomyces lanuginosus lipase - a switch for activity and binding

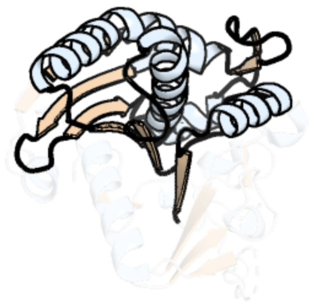

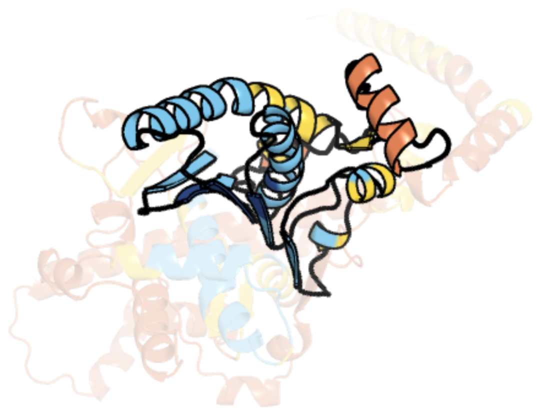

Figure 434: left: reference structure of 6a0w chain A. right: predicted structure of chlorv-3..391, unaligned sequences are shown as transparent

chlorv-4..001

- Sequence-based annotation for chlorv-4..001 is
- Best hit was 5l9b chain A: Egl nine homolog 1

target	prob	fident	alnlen	evalue	theader
5l9b-assembly1.cif.gz_A	1	0.128	210	6.825e-07	HIF PROLYL HYDROXYLASE 2 (PHD2/ EGLN1) IN COMPLEX WITH 2-OXOGLUTARATE (2OG) AND HIF-1ALPHA CODD (556-574)
4j25-assembly8.cif.gz_H	1	0.094	211	8.055e-07	Crystal structure of a Pseudomonas putida prolyl-4-hydroxylase (P4H)
4j25-assembly7.cif.gz_G	1	0.095	209	1.062e-06	Crystal structure of a Pseudomonas putida prolyl-4-hydroxylase (P4H)

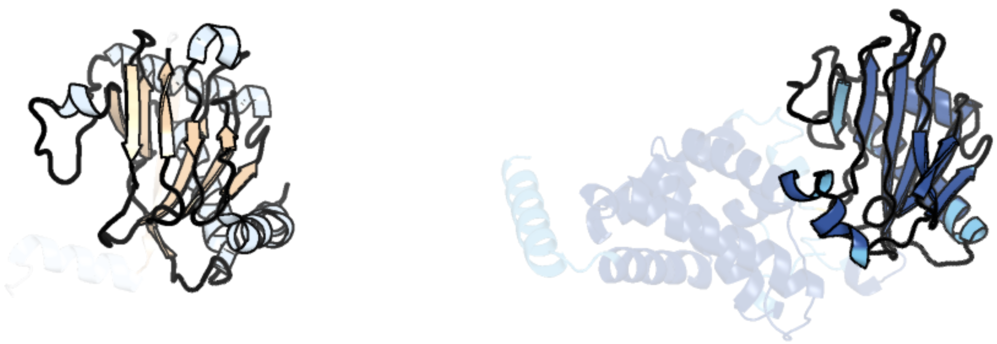

Figure 435: left: reference structure of 5l9b chain A. right: predicted structure of chlorv-4..001, unaligned sequences are shown as transparent

chlorv-4..002

- Sequence-based annotation for chlorv-4..002 is
- Best hit was 2rdq chain A: 1-deoxypentalenic acid 11-beta hydroxylase; Fe(II)/alpha-ketoglutarate dependent hydroxylase

target	prob	fident	alnlen	evalue	theader
2rdq-assembly1.cif.gz_A	1	0.128	272	9.521e-10	Crystal Structure of PtlH with Fe/alpha ketoglutarate bound
7eys-assembly2.cif.gz_A	1	0.147	264	4.767e-09	Complex structure of SptF with Fe, alpha-ketoglutarate, and andiconin D
7eyr-assembly2.cif.gz_A	1	0.159	264	5.74e-09	Fe(II)/(alpha)ketoglutarate-dependent dioxygenase SptF apo

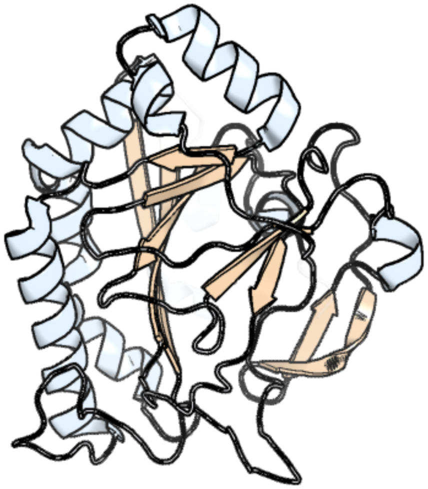

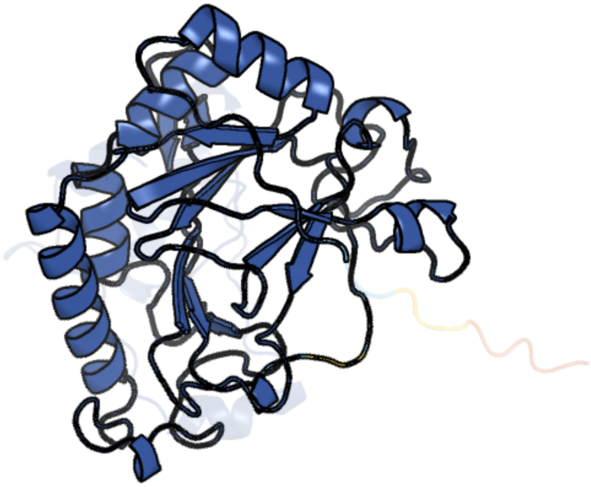

Figure 436: left: reference structure of 2rdq chain A. right: predicted structure of chlorv-4..002, unaligned sequences are shown as transparent

chlorv-4..004

- Sequence-based annotation for chlorv-4..004 is
- Best hit was 3ngm chain B: Extracellular lipase

target	prob	fident	alnlen	evalue	theader
3ngm-assembly2.cif.gz_B	1	0.2	170	1.182e-07	Crystal structure of lipase from Gibberella zeae
6a0w-assembly1.cif.gz_A	1	0.177	175	1.329e-07	Crystal structure of lipase from Rhizopus microsporus var. chinensis
2yij-assembly1.cif.gz_A	1	0.143	306	1.68e-07	Crystal Structure of phospholipase A1

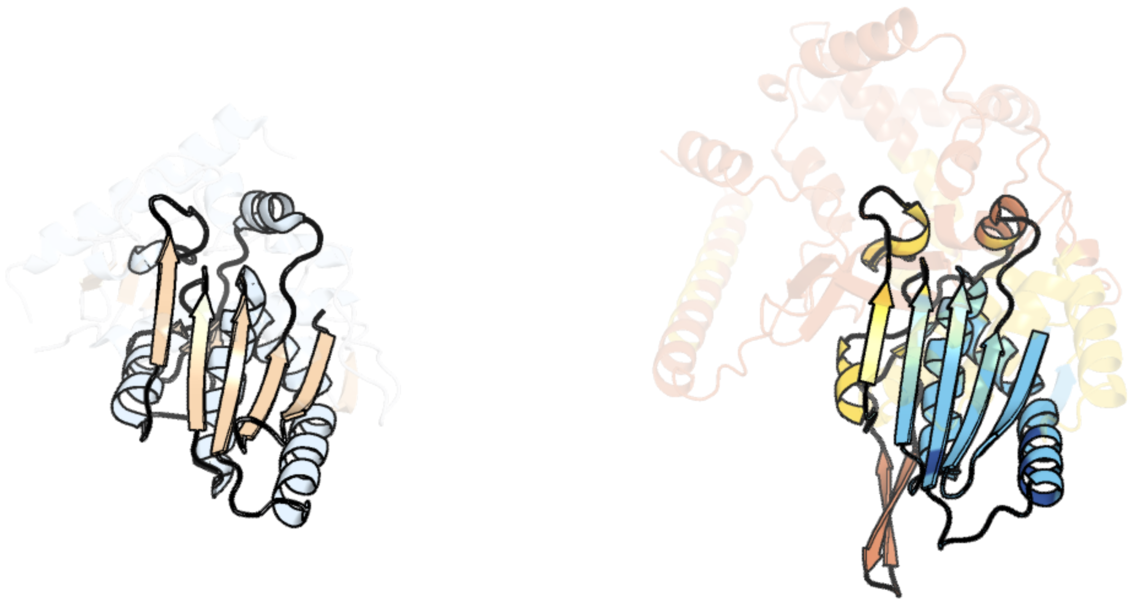

Figure 437: left: reference structure of 3ngm chain B. right: predicted structure of chlorv-4..004, unaligned sequences are shown as transparent

chlorv-4..011

- Sequence-based annotation for chlorv-4..011 is
- Best hit was 5h70 chain A: Probable thymidylate kinase

target	prob	fident	alnlen	evalue	theader
5h70-assembly1.cif.gz_A	1	0.143	209	3.871e-06	Crystal structure of ADP bound dTMP kinase (st1543) from Sulfolobus Tokodaii Strain7
7e9v-assembly1.cif.gz_A	1	0.131	197	1.292e-05	The Crystal Structure of human UMP-CMP kinase from Biortus.
4ukd-assembly1.cif.gz_A	1	0.154	188	1.377e-05	UMP/CMP KINASE FROM SLIME MOLD COMPLEXED WITH ADP, UDP, BERYLLIUM FLUORIDE

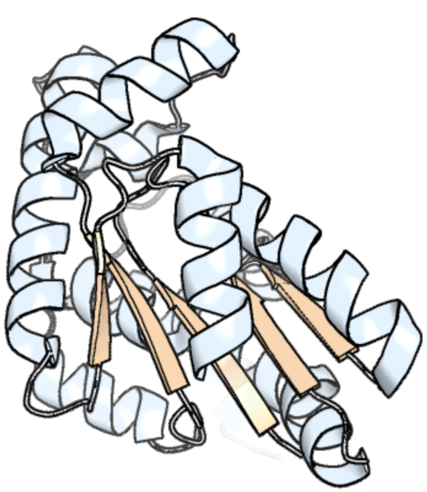

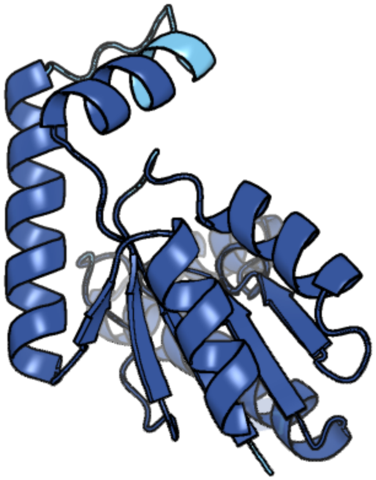

Figure 438: left: reference structure of 5h70 chain A. right: predicted structure of chlorv-4..011, unaligned sequences are shown as transparent

chlorv-4..012

- Sequence-based annotation for chlorv-4..012 is
- Best hit was 8gxn chain B: caffeyl-CoA-O-methyltransferase

target	prob	fident	alnlen	evalue	theader
8gxn-assembly1.cif.gz_B	1	0.168	214	6.969e-08	The crystal structure of CsFAOMT2 in complex with SAH
5zw3-assembly1.cif.gz_B	1	0.12	250	1.869e-07	Crystal Structure of TrmR from B. subtilis
5zw3-assembly1.cif.gz_A	1	0.111	260	2.098e-07	Crystal Structure of TrmR from B. subtilis

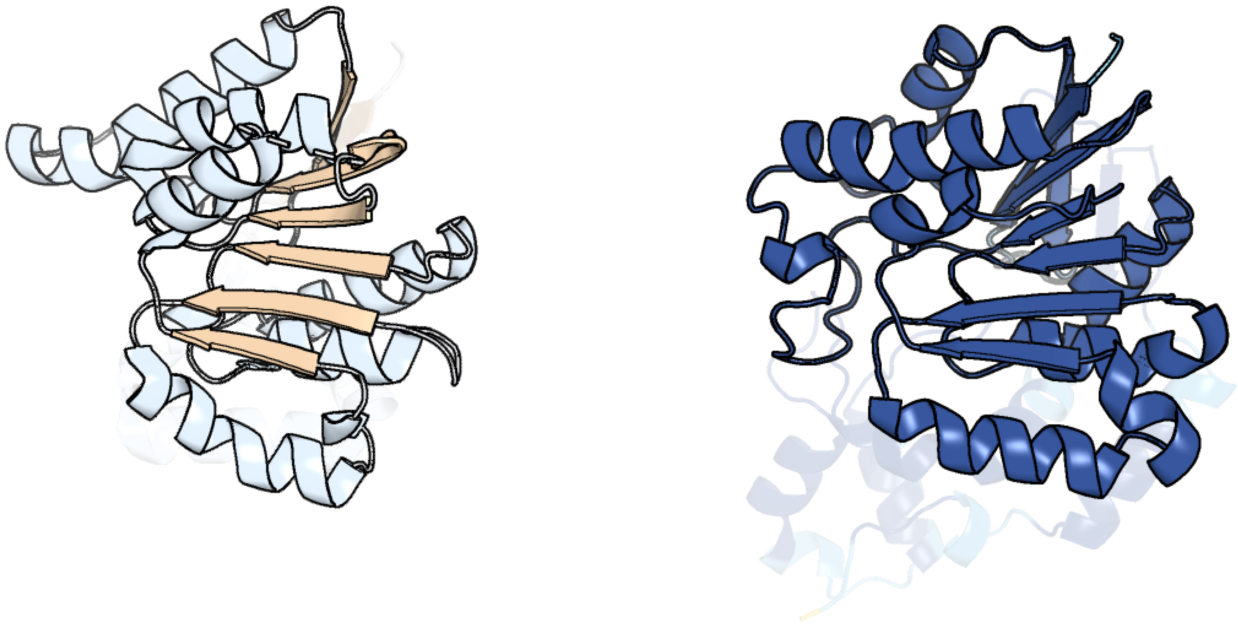

Figure 439: left: reference structure of 8gxn chain B. right: predicted structure of chlorv-4..012, unaligned sequences are shown as transparent

chlorv-4..013

- Sequence-based annotation for chlorv-4..013 is
- No significant structural hit found

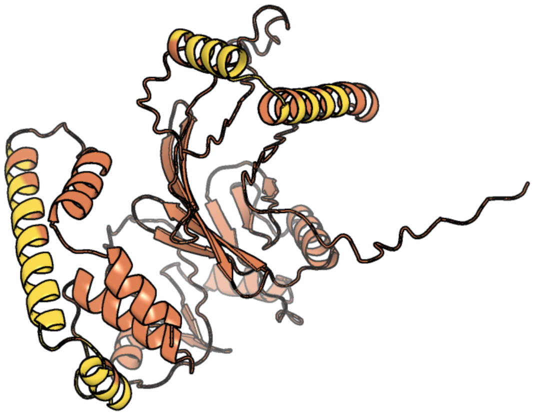

Figure 440: predicted structure of chlorv-4..013

chlorv-4..014

- Sequence-based annotation for chlorv-4..014 is
- Best hit was 1tf5 chain A: Preprotein translocase secA subunit

target	prob	fident	alnlen	evalue	theader
1tf5-assembly1.cif.gz_A	1	0.1	744	1.421e-08	Crystal structure of SecA in an open conformation from Bacillus Subtilis
1tf2-assembly1.cif.gz_A	1	0.103	761	3.061e-08	Crystal structure of SecA:ADP in an open conformation from Bacillus Subtilis
3jv2-assembly1.cif.gz_A	1	0.094	787	5.262e-08	Crystal Structure of B. subtilis SecA with bound peptide

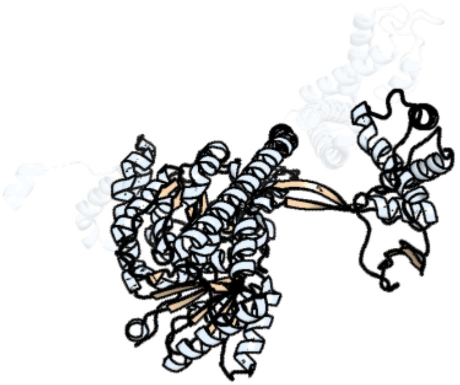

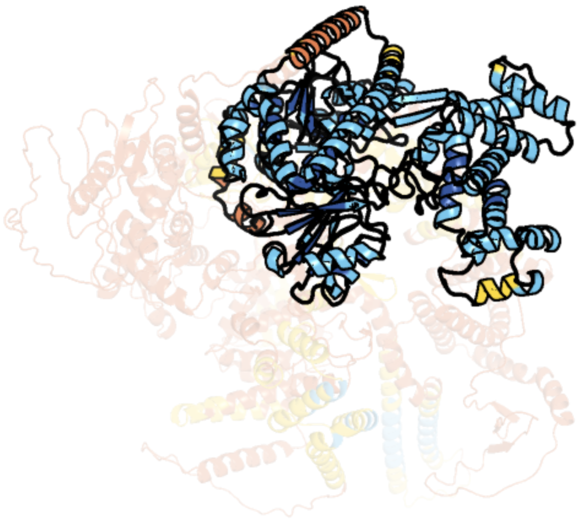

Figure 441: left: reference structure of 1tf5 chain A. right: predicted structure of chlorv-4..014, unaligned sequences are shown as transparent

chlorv-4..016

- Sequence-based annotation for chlorv-4..016 is
- No significant structural hit found

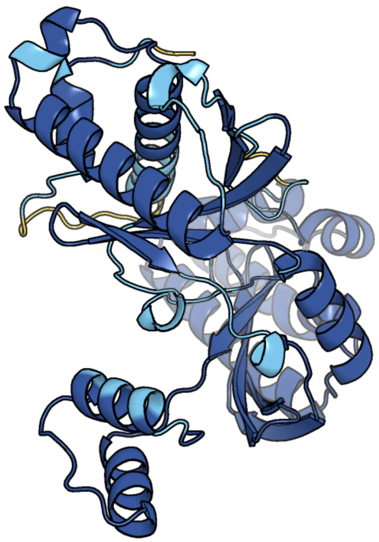

Figure 442: predicted structure of chlorv-4..016

chlorv-4..017

- Sequence-based annotation for chlorv-4..017 is
- Best hit was 7lt5 chain C: Site-specific DNA-methyltransferase (adenine-specific)

target	prob	fident	alnlen	evalue	theader
7lt5-assembly3.cif.gz_C	1	0.169	542	1.807e-18	CamA Adenine Methyltransferase Complexed to Cognate Substrate DNA and Cofactor SAH
7rfk-assembly3.cif.gz_C	1	0.164	541	5.356e-18	CamA Adenine Methyltransferase Complexed to Cognate Substrate DNA and Inhibitor Sinefungin
7qw7-assembly1.cif.gz_A	1	0.166	523	1.325e-17	Adenine-specific DNA methyltransferase M.BseCI complexed with AdoHcy and cognate fully methylated DNA duplex

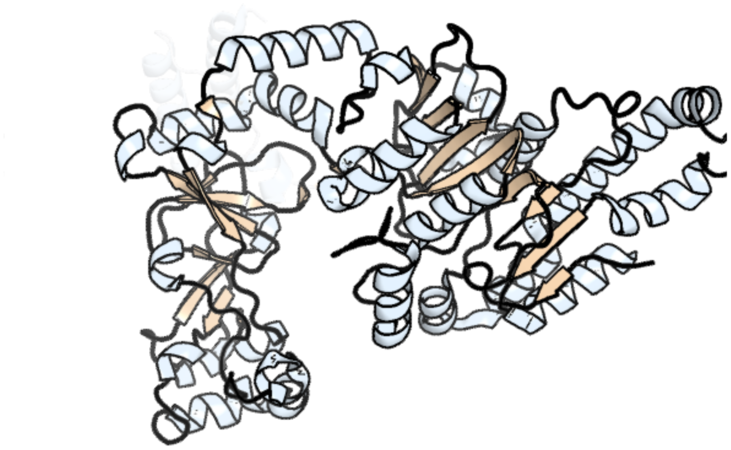

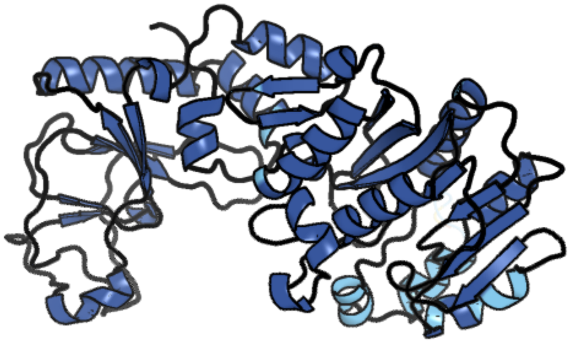

Figure 443: left: reference structure of 7lt5 chain C. right: predicted structure of chlorv-4..017, unaligned sequences are shown as transparent

chlorv-4..028

- Sequence-based annotation for chlorv-4..028 is
- No significant structural hit found

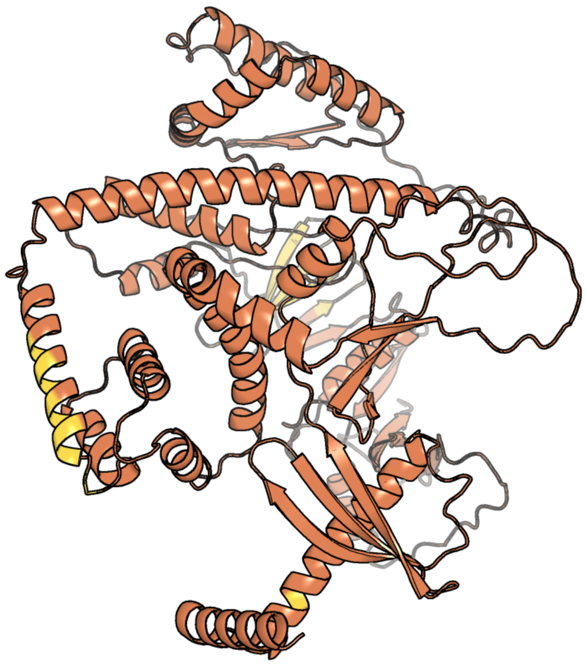

Figure 444: predicted structure of chlorv-4..028

chlorv-4..032

- Sequence-based annotation for chlorv-4..032 is
- No significant structural hit found

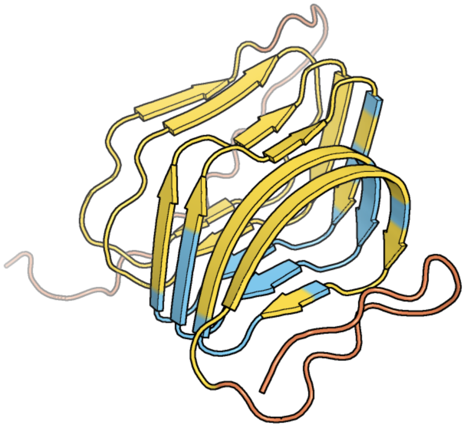

Figure 445: predicted structure of chlorv-4..032

chlorv-4..037

- Sequence-based annotation for chlorv-4..037 is
- No significant structural hit found

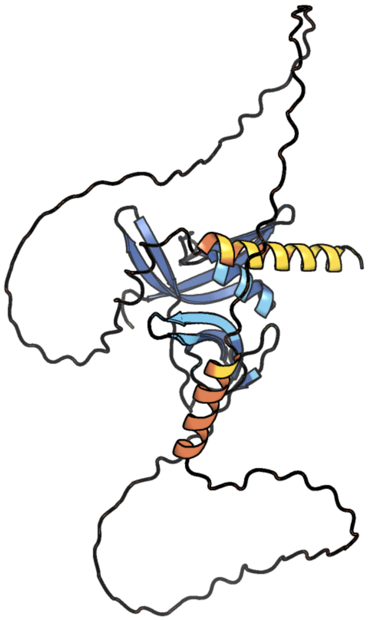

Figure 446: predicted structure of chlorv-4..037

chlorv-4..048

- Sequence-based annotation for chlorv-4..048 is
- No significant structural hit found

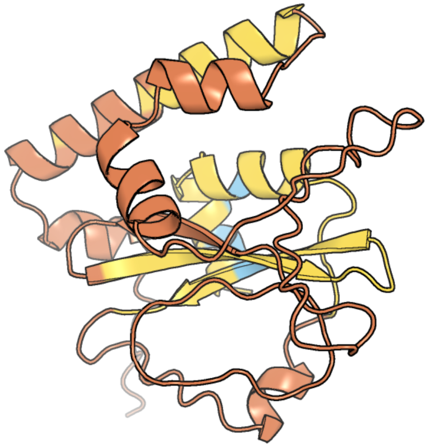

Figure 447: predicted structure of chlorv-4..048

chlorv-4..059

- Sequence-based annotation for chlorv-4..059 is
- Best hit was 8ssr chain A: Transcriptional repressor CTCF

target	prob	fident	alnlen	evalue	theader
8ssr-assembly1.cif.gz_A	1	0.194	268	2.036e-08	ZnFs 3-11 of CCCTC-binding factor (CTCF) Complexed with 35mer DNA 35-20
8ssu-assembly1.cif.gz_A	1	0.212	250	3.951e-08	ZnFs 3-11 of CCCTC-binding factor (CTCF) Complexed with 19mer DNA
8ffz-assembly1.cif.gz_A	1	0.222	193	4.458e-08	TFIIIA-TFIIIC-Brf1-TBP complex bound to 5S rRNA gene

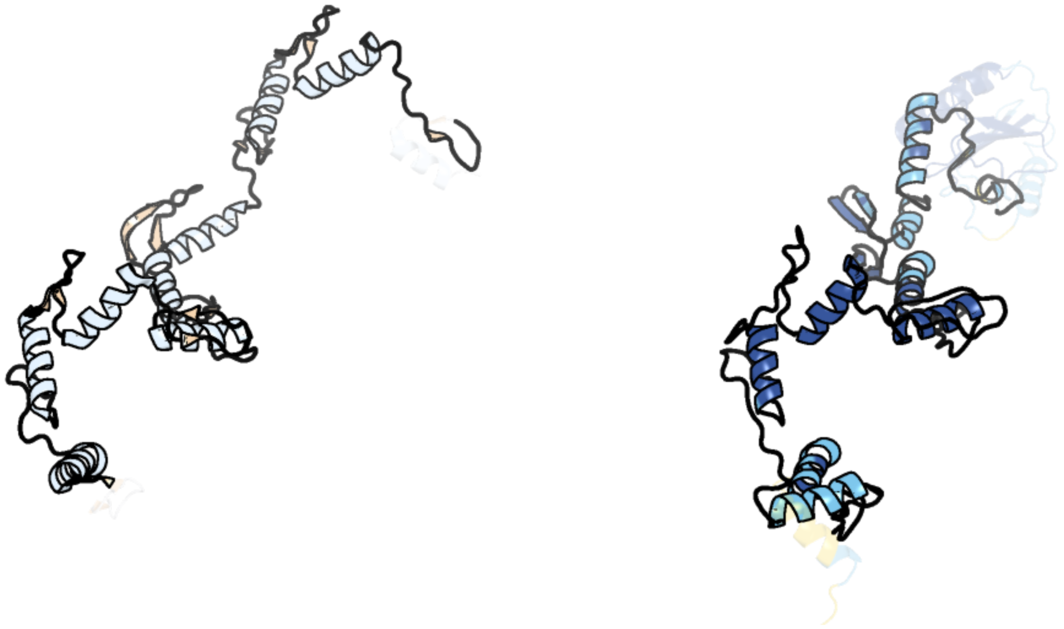

Figure 448: left: reference structure of 8ssr chain A. right: predicted structure of chlorv-4..059, unaligned sequences are shown as transparent

chlorv-4..061

- Sequence-based annotation for chlorv-4..061 is
- No significant structural hit found

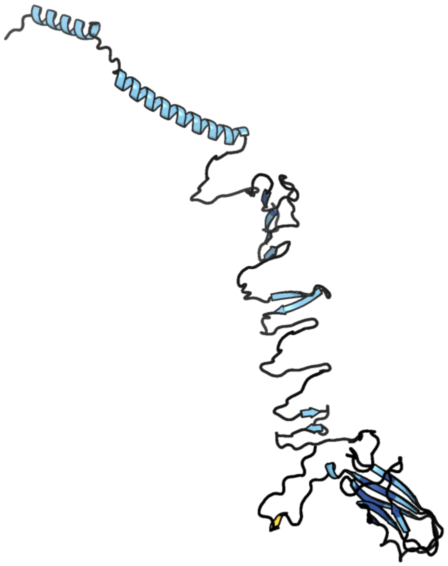

Figure 449: predicted structure of chlorv-4..061

chlorv-4..064

- Sequence-based annotation for chlorv-4..064 is
- No significant structural hit found

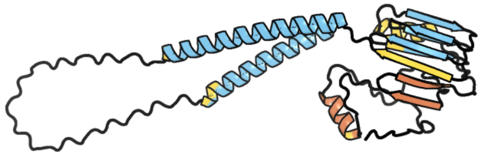

Figure 450: predicted structure of chlorv-4..064

chlorv-4..071

- Sequence-based annotation for chlorv-4..071 is
- Best hit was 2qip chain A: Protein of unknown function VPA0982

target	prob	fident	alnlen	evalue	theader
2qip-assembly1.cif.gz_A-2	1	0.201	159	4.608e-09	Crystal structure of a protein of unknown function VPA0982 from Vibrio parahaemolyticus RIMD 2210633
5yaa-assembly4.cif.gz_D	1	0.175	148	1.056e-06	Crystal structure of Marf1 NYN domain from Mus musculus
6fdl-assembly2.cif.gz_B	1	0.164	152	1.612e-06	Crystal structure of the NYN domain of human MARF1

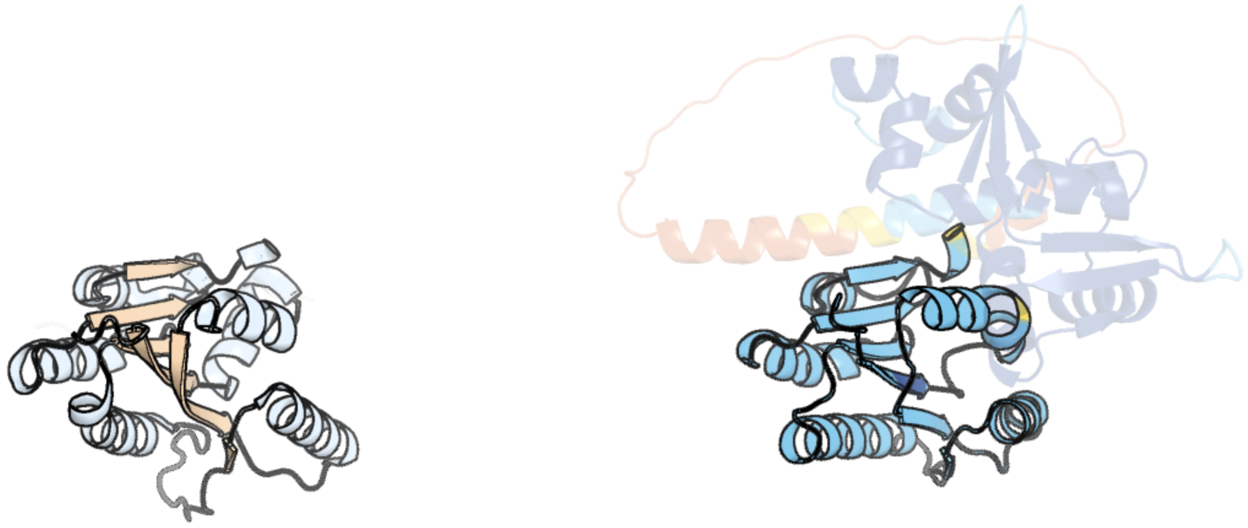

Figure 451: left: reference structure of 2qip chain A. right: predicted structure of chlorv-4..071, unaligned sequences are shown as transparent

chlorv-4..073

- Sequence-based annotation for chlorv-4..073 is
- No significant structural hit found

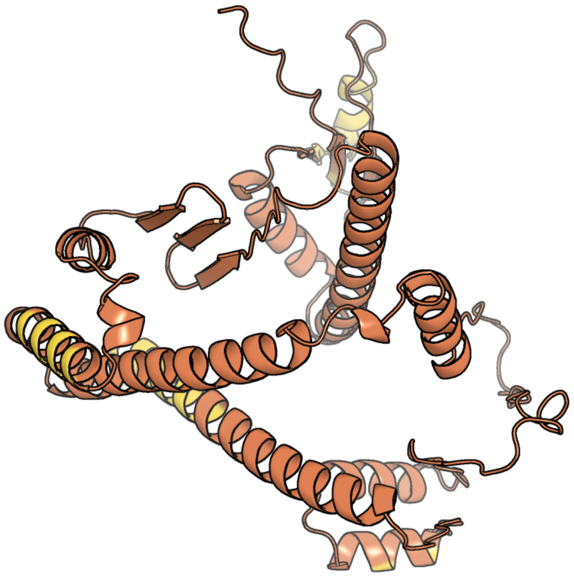

Figure 452: predicted structure of chlorv-4..073

chlorv-4..074

- Sequence-based annotation for chlorv-4..074 is
- No significant structural hit found

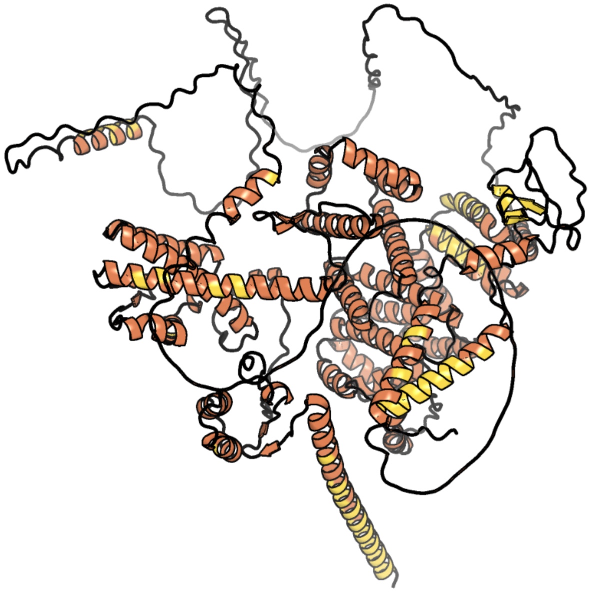

Figure 453: predicted structure of chlorv-4..074

chlorv-4..076

- Sequence-based annotation for chlorv-4..076 is
- Best hit was 3c48 chain B: Predicted glycosyltransferases

target	prob	fident	alnlen	evalue	theader
3c48-assembly1.cif.gz_B	1	0.11	418	7.713e-10	Structure of the retaining glycosyltransferase MshA: The first step in mycothiol biosynthesis. Organism: Corynebacterium glutamicum- APO (OPEN) structure.
3c4q-assembly2.cif.gz_B	1	0.115	426	8.149e-10	Structure of the retaining glycosyltransferase MshA : The first step in mycothiol biosynthesis. Organism : Corynebacterium glutamicum- Complex with UDP
5d00-assembly1.cif.gz_A	1	0.107	401	8.61e-10	Crystal structure of BshA from B. subtilis complexed with N-acetylglucosaminyl-malate and UMP

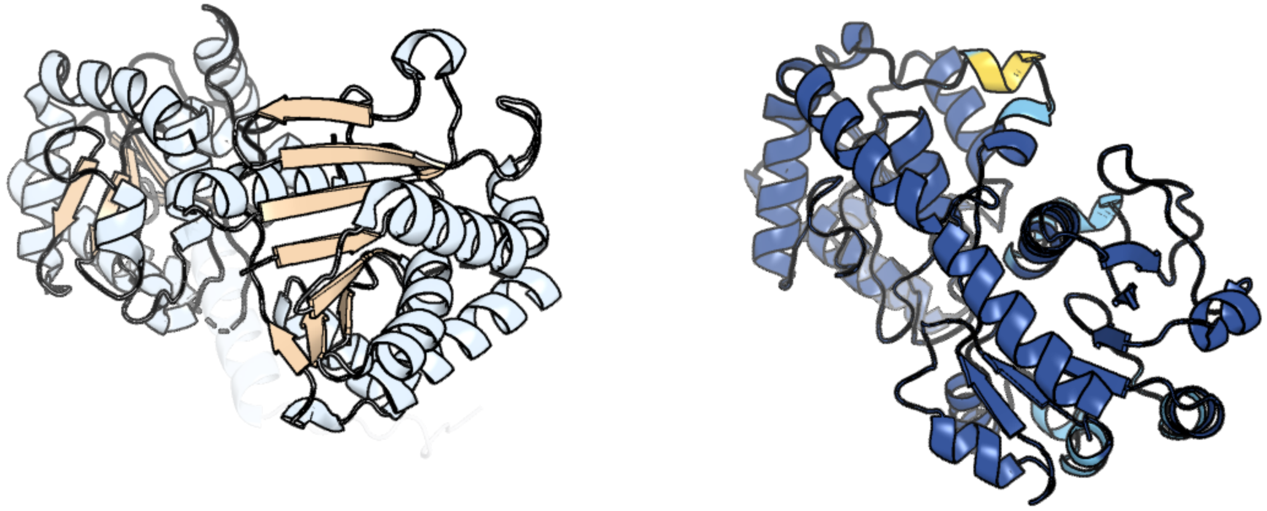

Figure 454: left: reference structure of 3c48 chain B. right: predicted structure of chlorv-4..076, unaligned sequences are shown as transparent

chlorv-4..080

- Sequence-based annotation for chlorv-4..080 is
- No significant structural hit found

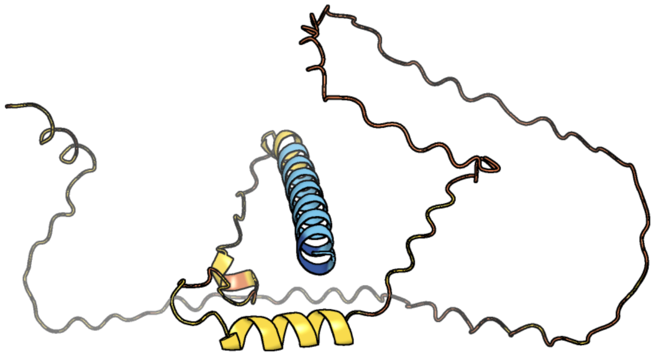

Figure 455: predicted structure of chlorv-4..080

chlorv-4..085

- Sequence-based annotation for chlorv-4..085 is
- No significant structural hit found

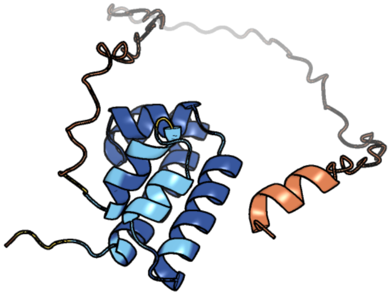

Figure 456: predicted structure of chlorv-4..085

chlorv-4..101

- Sequence-based annotation for chlorv-4..101 is
- No significant structural hit found

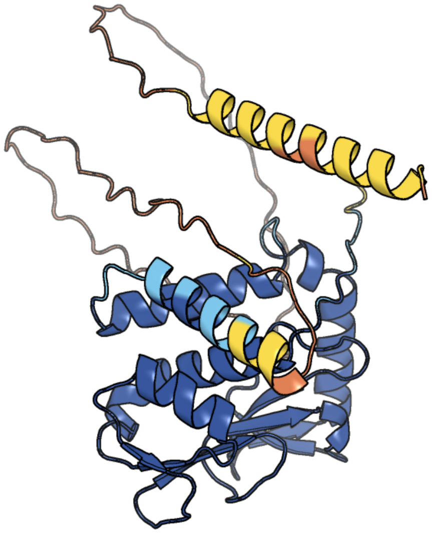

Figure 457: predicted structure of chlorv-4..101

chlorv-4..102

- Sequence-based annotation for chlorv-4..102 is
- No significant structural hit found

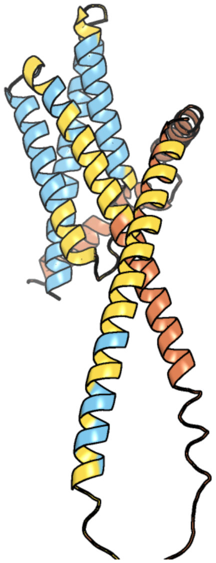

Figure 458: predicted structure of chlorv-4..102

chlorv-4..103

- Sequence-based annotation for chlorv-4..103 is
- No significant structural hit found

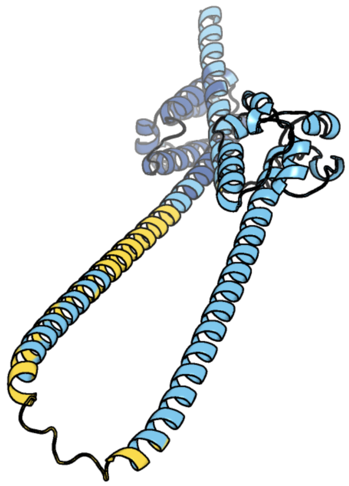

Figure 459: predicted structure of chlorv-4..103

chlorv-4..106

- Sequence-based annotation for chlorv-4..106 is
- No significant structural hit found

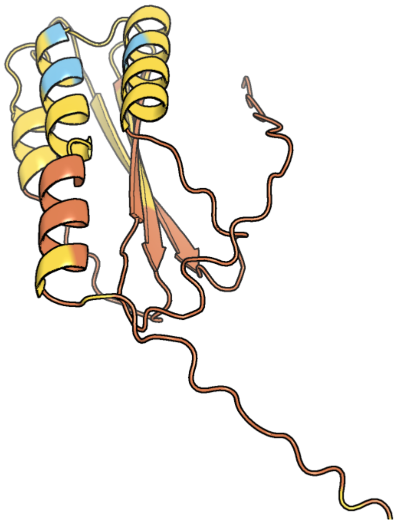

Figure 460: predicted structure of chlorv-4..106

chlorv-4..121

- Sequence-based annotation for chlorv-4..121 is
- No significant structural hit found

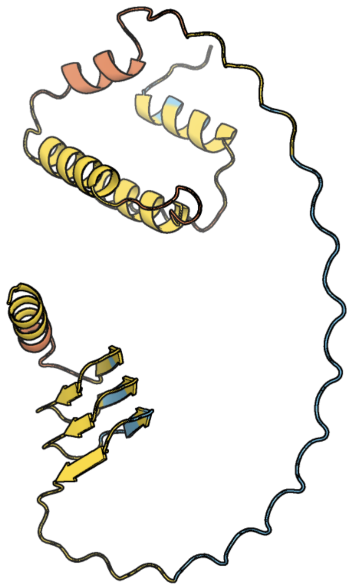

Figure 461: predicted structure of chlorv-4..121

chlorv-4..122

- Sequence-based annotation for chlorv-4..122 is
- No significant structural hit found

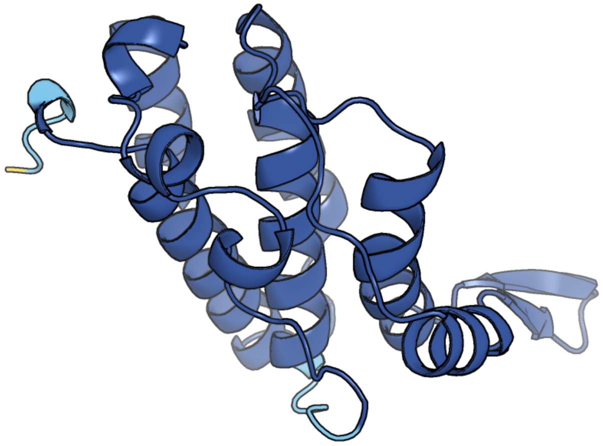

Figure 462: predicted structure of chlorv-4..122

chlorv-4..124

- Sequence-based annotation for chlorv-4..124 is
- No significant structural hit found

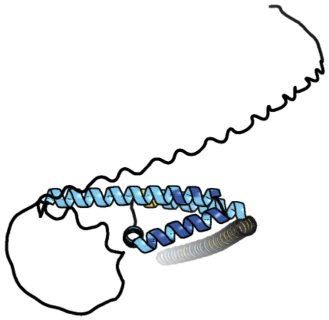

Figure 463: predicted structure of chlorv-4..124

chlorv-4..135

- Sequence-based annotation for chlorv-4..135 is
- No significant structural hit found

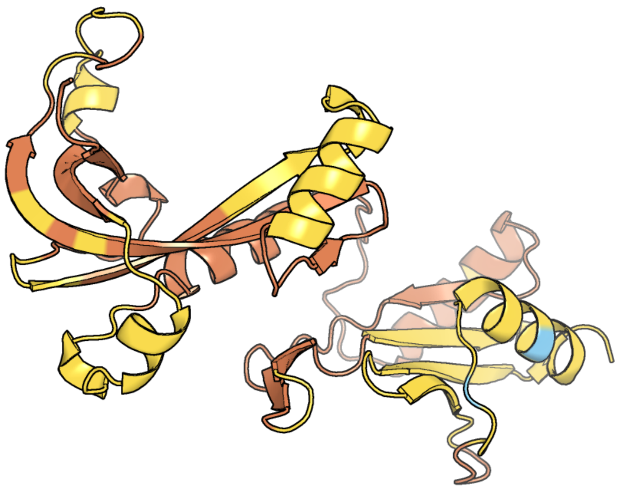

Figure 464: predicted structure of chlorv-4..135

chlorv-4..148

- Sequence-based annotation for chlorv-4..148 is
- Best hit was 7z2c chain K: Kinesin-like protein

target	prob	fident	alnlen	evalue	theader
7z2c-assembly1.cif.gz_K	1	0.157	363	1.324e-06	P. falciparum kinesin-8B motor domain in no nucleotide bound to tubulin dimer
7z2a-assembly1.cif.gz_K	1	0.142	365	1.39e-06	P. berghei kinesin-8B motor domain in no nucleotide state bound to tubulin dimer
7tr0-assembly1.cif.gz_K	1	0.144	332	3.171e-06	CaKip3[2-436] - AMP-PNP in complex with a microtubule

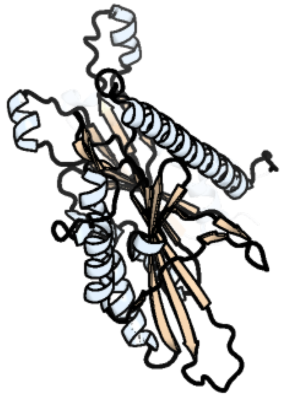

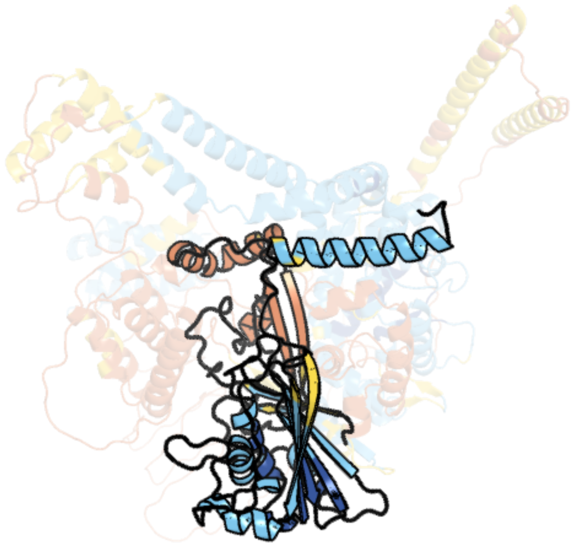

Figure 465: left: reference structure of 7z2c chain K. right: predicted structure of chlorv-4..148, unaligned sequences are shown as transparent

chlorv-4..151

- Sequence-based annotation for chlorv-4..151 is
- No significant structural hit found

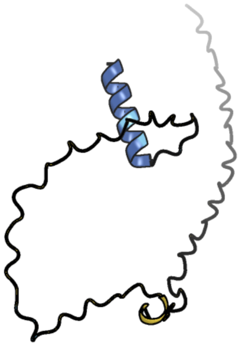

Figure 466: predicted structure of chlorv-4..151

chlorv-4..153

- Sequence-based annotation for chlorv-4..153 is
- No significant structural hit found

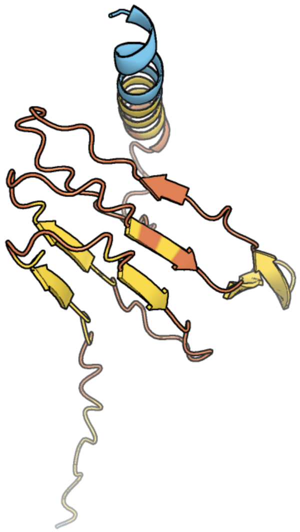

Figure 467: predicted structure of chlorv-4..153

chlorv-4..161

- Sequence-based annotation for chlorv-4..161 is
- No significant structural hit found

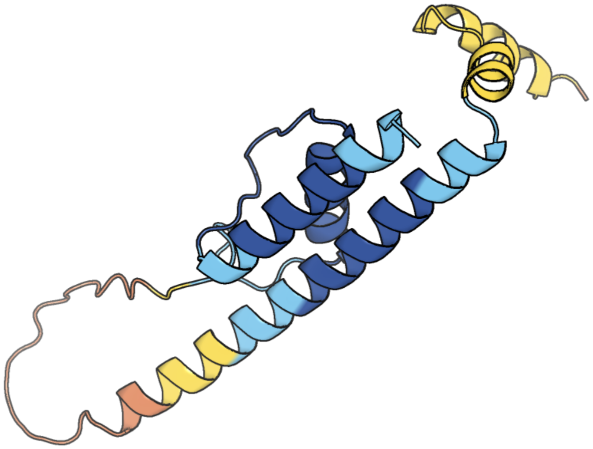

Figure 468: predicted structure of chlorv-4..161

chlorv-4..167

- Sequence-based annotation for chlorv-4..167 is
- No significant structural hit found

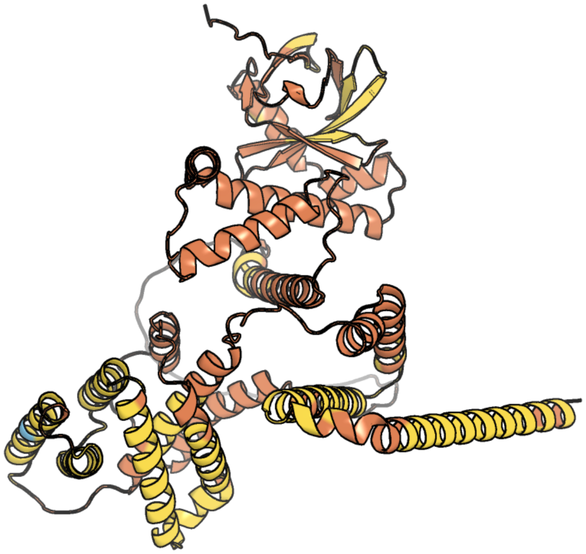

Figure 469: predicted structure of chlorv-4..167

chlorv-4..168

- Sequence-based annotation for chlorv-4..168 is
- No significant structural hit found

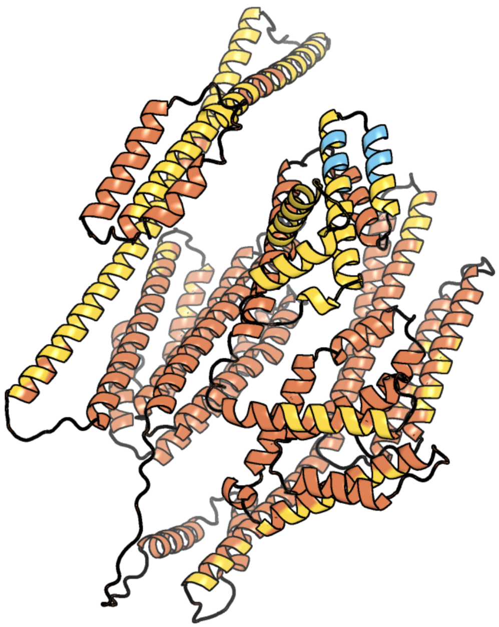

Figure 470: predicted structure of chlorv-4..168

chlorv-4..169

- Sequence-based annotation for chlorv-4..169 is
- No significant structural hit found

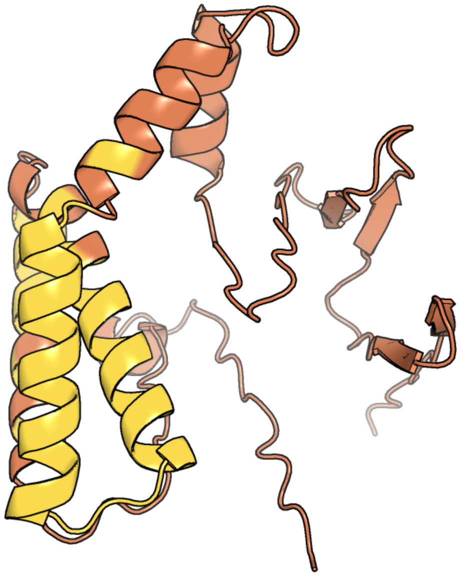

Figure 471: predicted structure of chlorv-4..169

chlorv-4..178

- Sequence-based annotation for chlorv-4..178 is
- No significant structural hit found

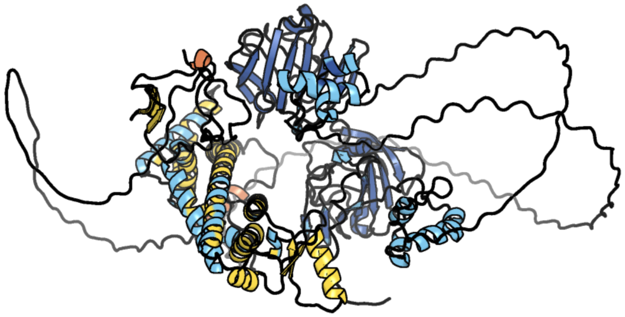

Figure 472: predicted structure of chlorv-4..178

chlorv-4..180

- Sequence-based annotation for chlorv-4..180 is
- No significant structural hit found

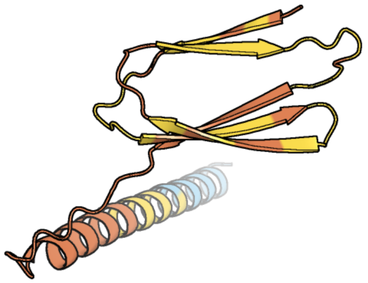

Figure 473: predicted structure of chlorv-4..180

chlorv-4..191

- Sequence-based annotation for chlorv-4..191 is
- No significant structural hit found

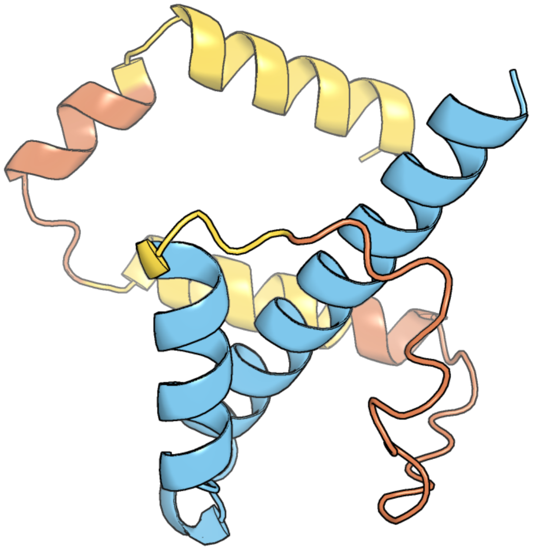

Figure 474: predicted structure of chlorv-4..191

chlorv-4..193

- Sequence-based annotation for chlorv-4..193 is
- No significant structural hit found

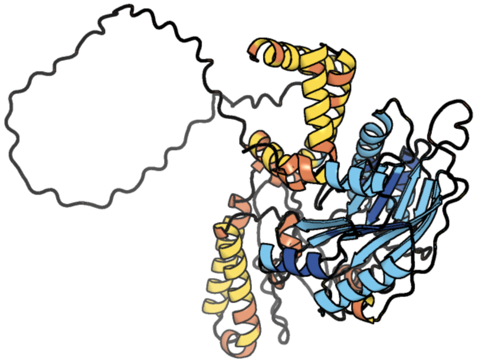

Figure 475: predicted structure of chlorv-4..193

chlorv-4..201

- Sequence-based annotation for chlorv-4..201 is
- Best hit was 1u7p chain D: magnesium-dependent phosphatase-1

target	prob	fident	alnlen	evalue	theader
1u7p-assembly4.cif.gz_D	1	0.188	170	5.713e-05	X-ray Crystal Structure of the Hypothetical Phosphotyrosine Phosphatase MDP-1 of the Haloacid Dehalogenase Superfamily
2ah5-assembly1.cif.gz_A	1	0.137	189	0.0002145	Hydrolase, haloacid dehalogenase-like family protein SP0104 from Streptococcus pneumoniae
3nrj-assembly3.cif.gz_I	1	0.124	185	0.0002728	Crystal structure of probable yrbi family phosphatase from pseudomonas syringae pv.phaseolica 1448a complexed with magnesium

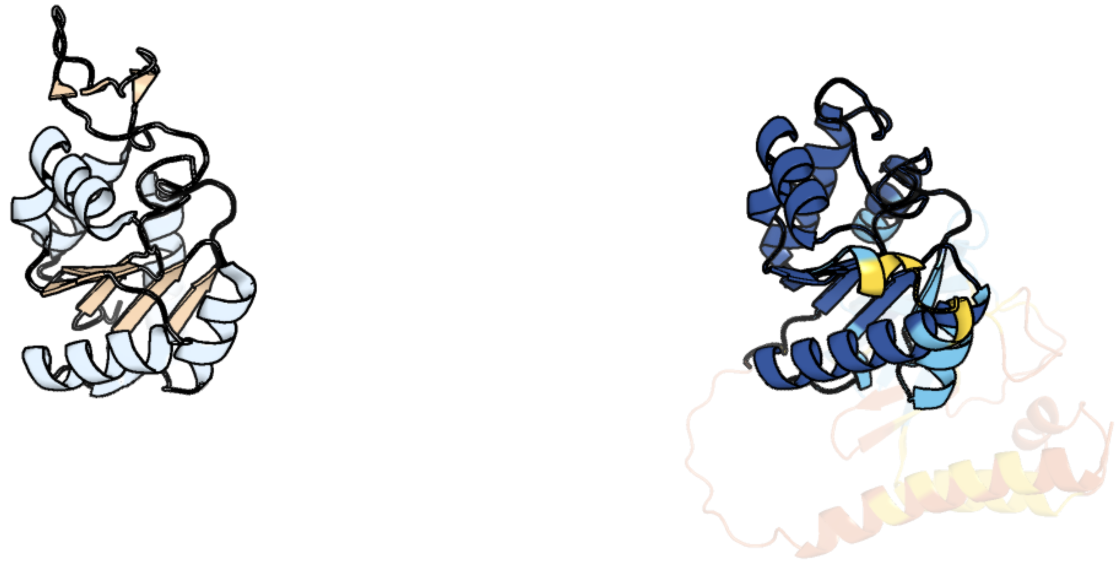

Figure 476: left: reference structure of 1u7p chain D. right: predicted structure of chlorv-4..201, unaligned sequences are shown as transparent

chlorv-4..203

- Sequence-based annotation for chlorv-4..203 is
- Best hit was 1k9e chain A: alpha-D-glucuronidase

target	prob	fident	alnlen	evalue	theader
1k9e-assembly1.cif.gz_A	1	0.105	841	3.023e-14	Crystal structure of a mutated family-67 alpha-D-glucuronidase (E285N) from Bacillus stearothermophilus T-6, complexed with 4-O-methyl-glucuronic acid
6hze-assembly2.cif.gz_B	1	0.095	1008	3.53e-14	BP0997, GH138 enzyme targeting pectin rhamnogalacturonan II
1mqr-assembly1.cif.gz_A-2	1	0.11	838	1.669e-13	THE CRYSTAL STRUCTURE OF ALPHA-D-GLUCURONIDASE (E386Q) FROM BACILLUS STEAROTHERMOPHILUS T-6

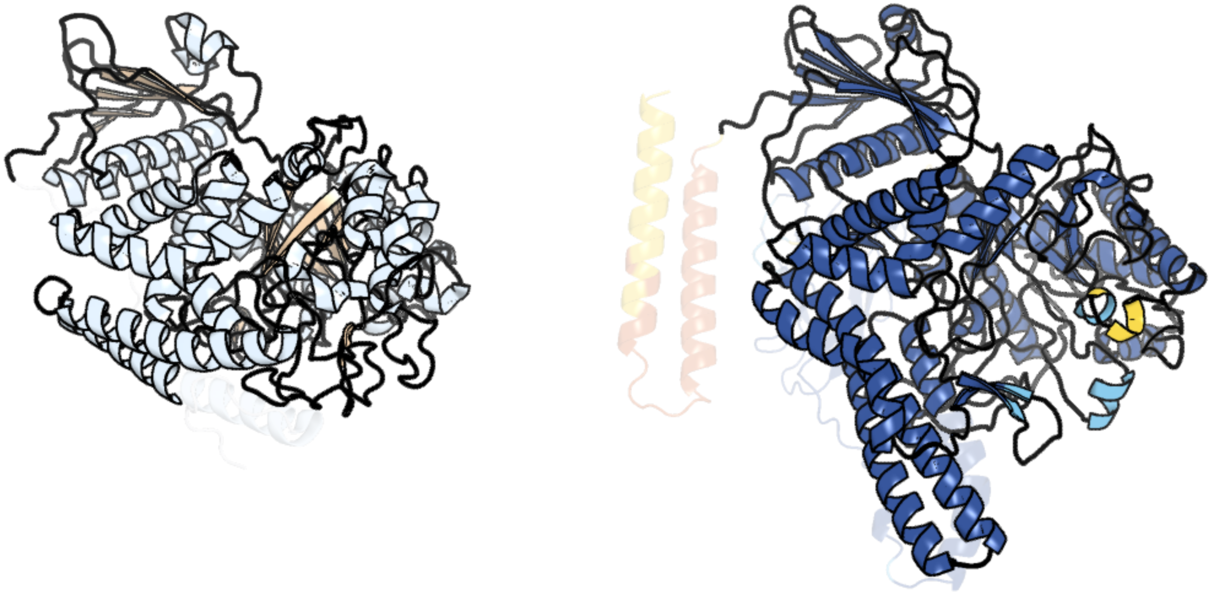

Figure 477: left: reference structure of 1k9e chain A. right: predicted structure of chlorv-4..203, unaligned sequences are shown as transparent

chlorv-4..204

- Sequence-based annotation for chlorv-4..204 is
- Best hit was 3kmj chain A: A612L protein

target	prob	fident	alnlen	evalue	theader
3kmj-assembly1.cif.gz_A	1	0.31	116	3.202e-10	Crystal structure of vSET under condition B
4rz0-assembly1.cif.gz_A	1	0.353	113	3.387e-10	Crystal Structure of Plasmodium falciparum putative histone methyltransferase PFL0690c
3kma-assembly1.cif.gz_B	1	0.283	106	3.18e-09	Crystal Structure of vSET under Condition A

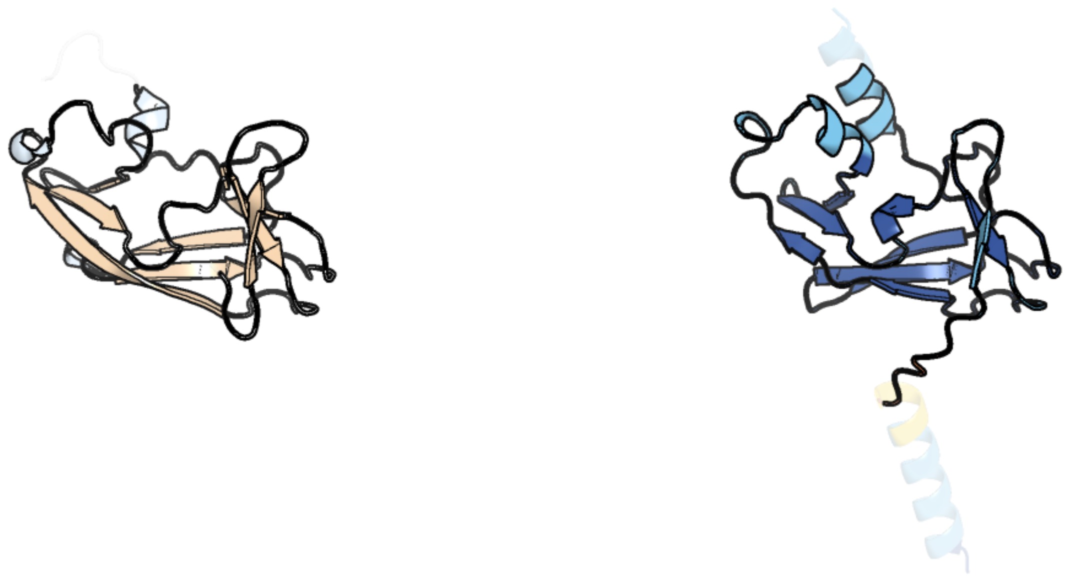

Figure 478: left: reference structure of 3kmj chain A. right: predicted structure of chlorv-4..204, unaligned sequences are shown as transparent

chlorv-4..212

- Sequence-based annotation for chlorv-4..212 is
- No significant structural hit found

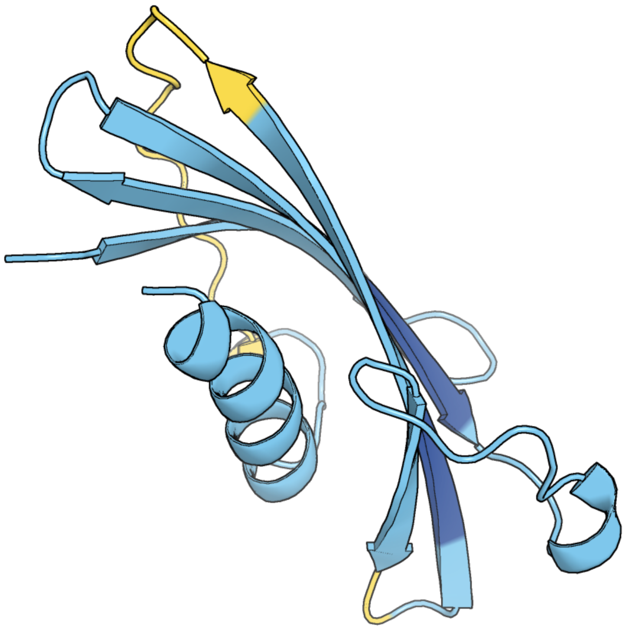

Figure 479: predicted structure of chlorv-4..212

chlorv-4..218

- Sequence-based annotation for chlorv-4..218 is
- No significant structural hit found

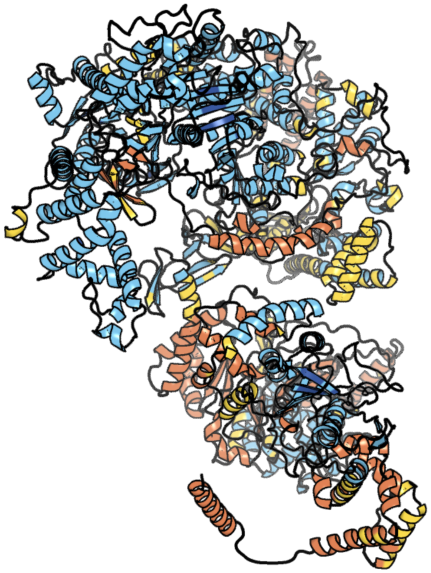

Figure 480: predicted structure of chlorv-4..218

chlorv-4..221

- Sequence-based annotation for chlorv-4..221 is
- Best hit was 7sqc chain 6B: FAP15

target	prob	fident	alnlen	evalue	theader
7sqc-assembly1.cif.gz_6B	1	0.172	307	8.09e-13	Ciliary C1 central pair apparatus isolated from Chlamydomonas reinhardtii
1s95-assembly2.cif.gz_B	1	0.179	317	1.009e-12	Structure of serine/threonine protein phosphatase 5
7tvf-assembly2.cif.gz_C	1	0.169	348	1.066e-12	Crystal structure of the SHOC2-MRAS-PP1CA (SMP) complex to a resolution of 2.17 Angstrom

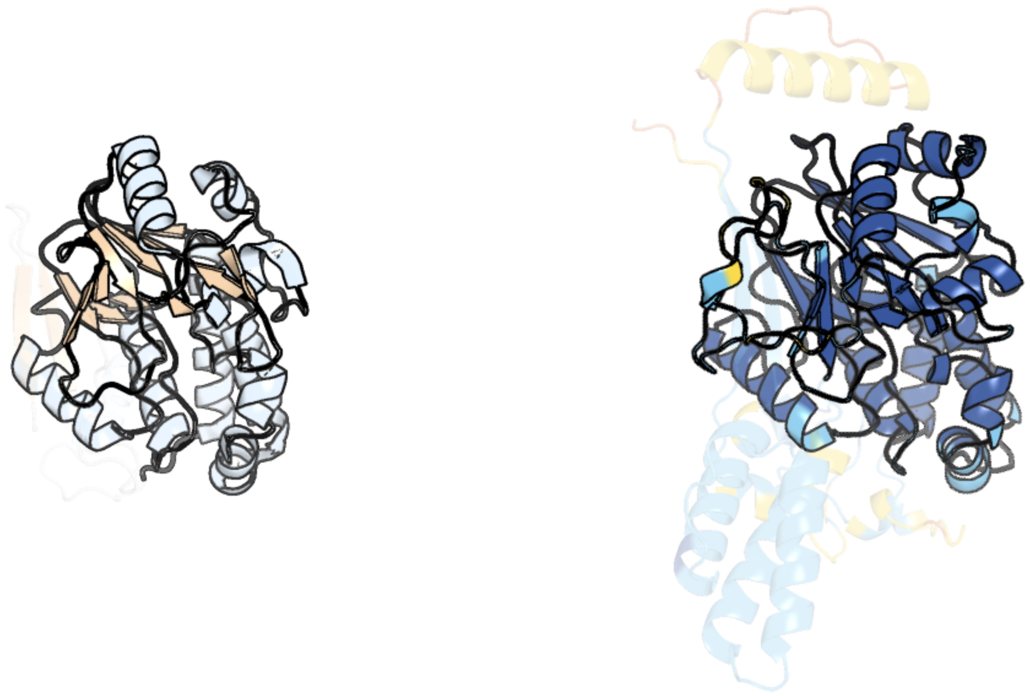

Figure 481: left: reference structure of 7sqc chain 6B. right: predicted structure of chlorv-4..221, unaligned sequences are shown as transparent

chlorv-4..244

- Sequence-based annotation for chlorv-4..244 is
- No significant structural hit found

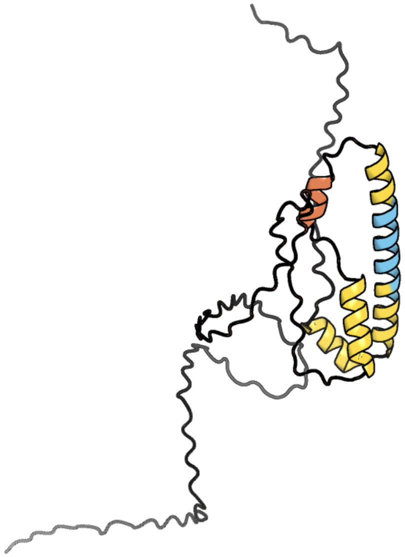

Figure 482: predicted structure of chlorv-4..244

chlorv-4..248

- Sequence-based annotation for chlorv-4..248 is
- No significant structural hit found

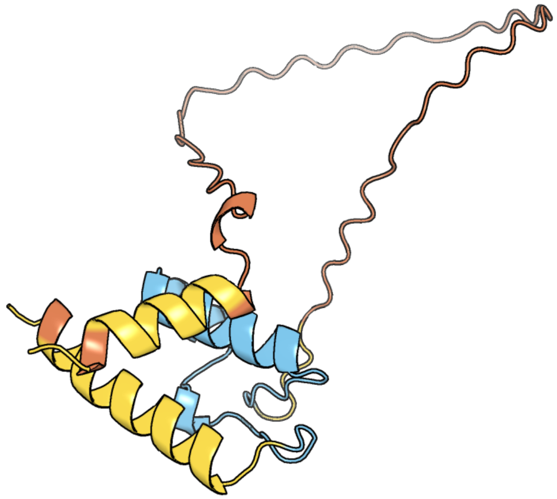

Figure 483: predicted structure of chlorv-4..248

chlorv-4..249

- Sequence-based annotation for chlorv-4..249 is
- No significant structural hit found

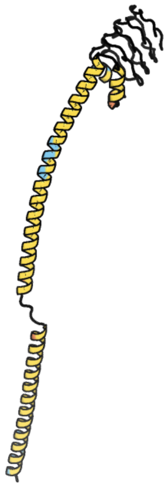

Figure 484: predicted structure of chlorv-4..249

chlorv-4..250

- Sequence-based annotation for chlorv-4..250 is
- No significant structural hit found

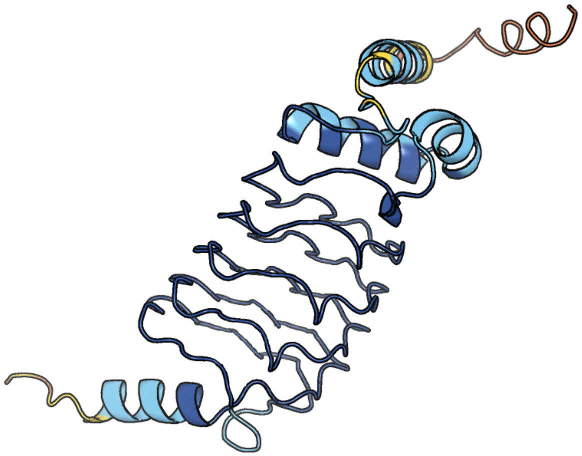

Figure 485: predicted structure of chlorv-4..250

chlorv-4..255

- Sequence-based annotation for chlorv-4..255 is
- No significant structural hit found

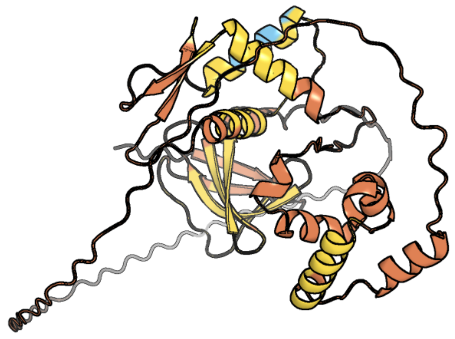

Figure 486: predicted structure of chlorv-4..255

chlorv-4..256

- Sequence-based annotation for chlorv-4..256 is
- Best hit was 5oxf chain A: GTP-binding protein

target	prob	fident	alnlen	evalue	theader
5oxf-assembly1.cif.gz__A	1	0.179	567	7.922e-08	An oligomerised bacterial dynamin pair provides a mechanism for the long range sensing and tethering of membranes
5oxf-assembly1.cif.gz__B	1	0.175	502	2.47e-06	An oligomerised bacterial dynamin pair provides a mechanism for the long range sensing and tethering of membranes
6jfm-assembly2.cif.gz__B	1	0.095	440	3.089e-06	Mitofusin2 (MFN2)__T111D

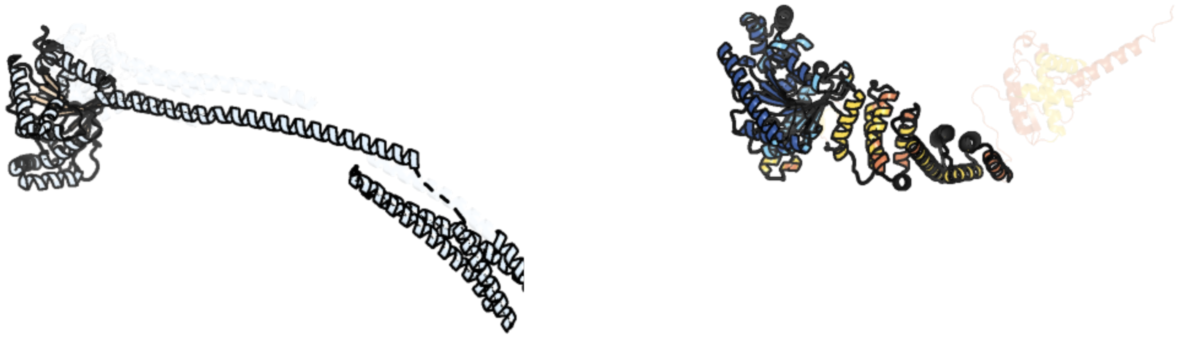

Figure 487: left: reference structure of 5oxf chain A. right: predicted structure of chlorv-4..256, unaligned sequences are shown as transparent

chlorv-4..263

- Sequence-based annotation for chlorv-4..263 is
- No significant structural hit found

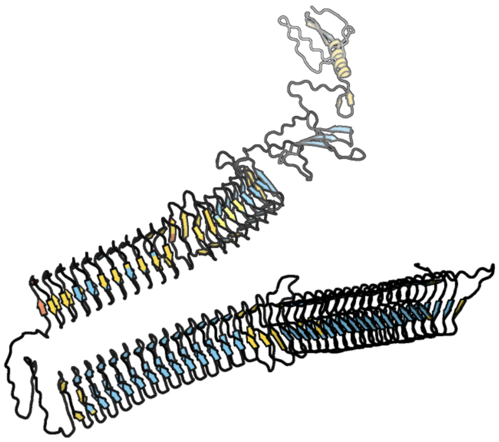

Figure 488: predicted structure of chlorv-4..263

chlorv-4..272

- Sequence-based annotation for chlorv-4..272 is
- No significant structural hit found

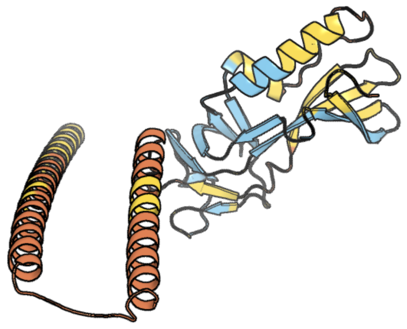

Figure 489: predicted structure of chlorv-4..272

chlorv-4..277

- Sequence-based annotation for chlorv-4..277 is
- Best hit was 2cdq chain B: ASPARTOKINASE

target	prob	fident	alnlen	evalue	theader
2cdq-assembly1.cif.gz_B	1	0.202	475	2.093e-28	Crystal structure of Arabidopsis thaliana aspartate kinase complexed with lysine and S-adenosylmethionine
3c1n-assembly3.cif.gz_D	1	0.255	470	2.093e-28	Crystal Structure of Allosteric Inhibition Threonine-sensitive Aspartokinase from Methanococcus jannaschii with L-threonine
3c1n-assembly2.cif.gz_B	1	0.236	477	3.895e-28	Crystal Structure of Allosteric Inhibition Threonine-sensitive Aspartokinase from Methanococcus jannaschii with L-threonine

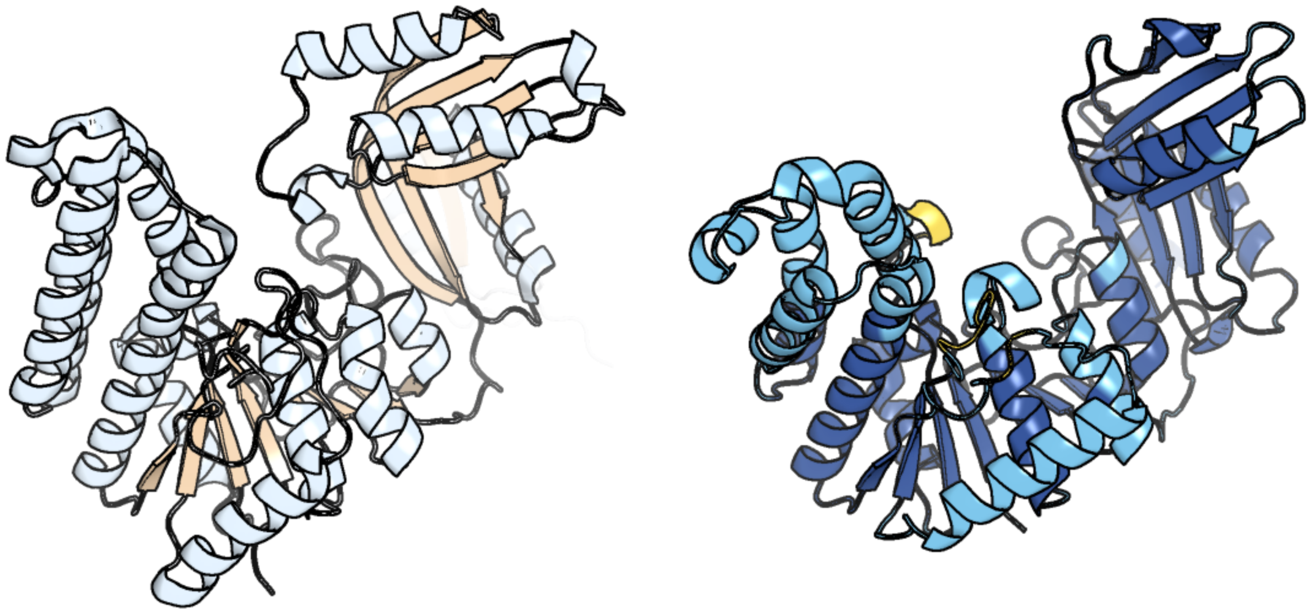

Figure 490: left: reference structure of 2cdq chain B. right: predicted structure of chlorv-4..277, unaligned sequences are shown as transparent

chlorv-4..282

- Sequence-based annotation for chlorv-4..282 is
- No significant structural hit found

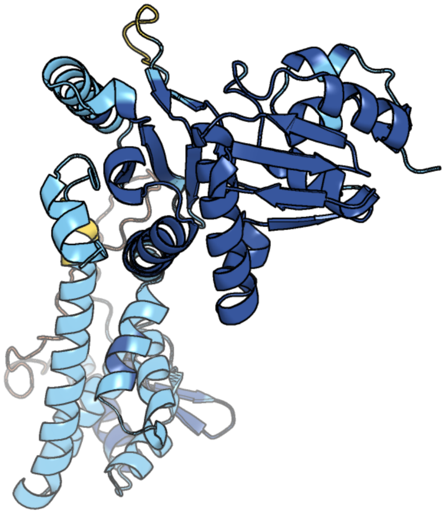

Figure 491: predicted structure of chlorv-4..282

chlorv-4..283

- Sequence-based annotation for chlorv-4..283 is
- No significant structural hit found

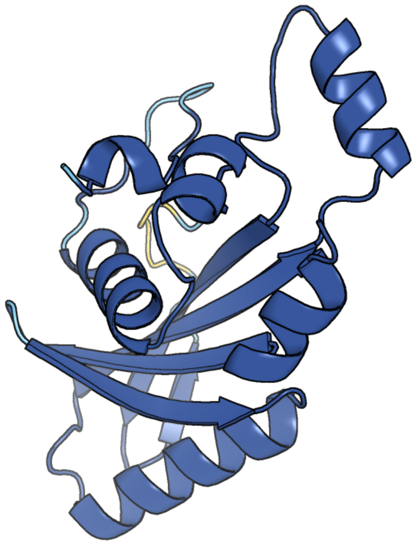

Figure 492: predicted structure of chlorv-4..283

chlorv-4..292

- Sequence-based annotation for chlorv-4..292 is
- No significant structural hit found

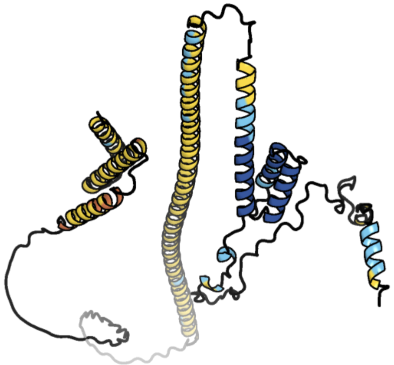

Figure 493: predicted structure of chlorv-4..292

chlorv-4..310

- Sequence-based annotation for chlorv-4..310 is
- Best hit was 5djs chain A: Tetratricopeptide TPR_2 repeat protein

target	prob	fident	alnlen	evalue	theader
5djs-assembly1.cif.gz_A	1	0.132	477	1.908e-17	Thermobaculum terrenum O-GlcNAc transferase mutant - K341M
8dth-assembly1.cif.gz_B	1	0.164	469	2.19e-17	Cryo-EM structure of Arabidopsis SPY alternative conformation 2
4gyw-assembly1.cif.gz_C	1	0.126	600	7.921e-17	Crystal structure of human O-GlcNAc Transferase in complex with UDP and a glycopeptide

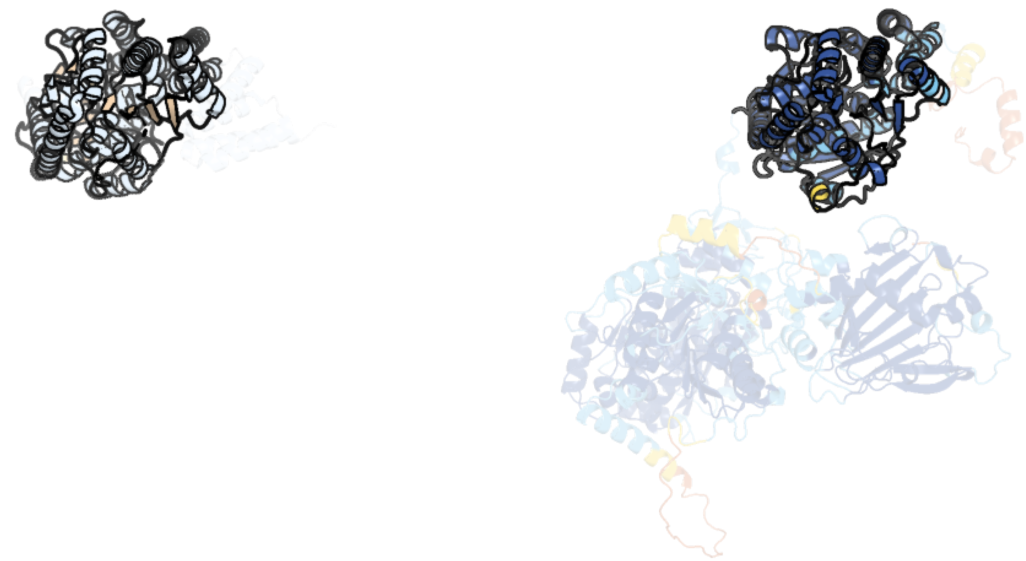

Figure 494: left: reference structure of 5djs chain A. right: predicted structure of chlorv-4..310, unaligned sequences are shown as transparent

chlorv-4..316

- Sequence-based annotation for chlorv-4..316 is
- No significant structural hit found

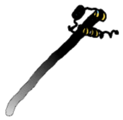

Figure 495: predicted structure of chlorv-4..316

chlorv-4..319

- Sequence-based annotation for chlorv-4..319 is
- No significant structural hit found

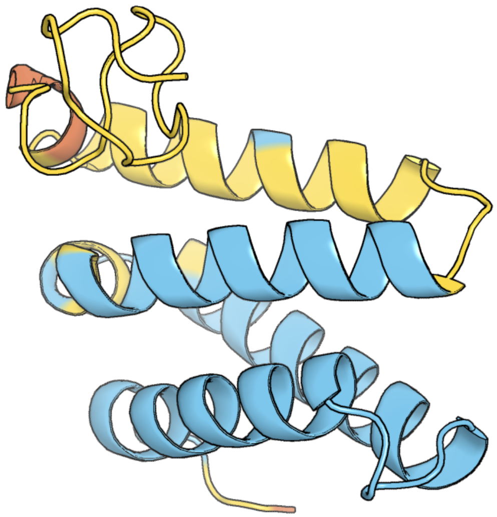

Figure 496: predicted structure of chlorv-4..319

chlorv-4..328

- Sequence-based annotation for chlorv-4..328 is
- No significant structural hit found

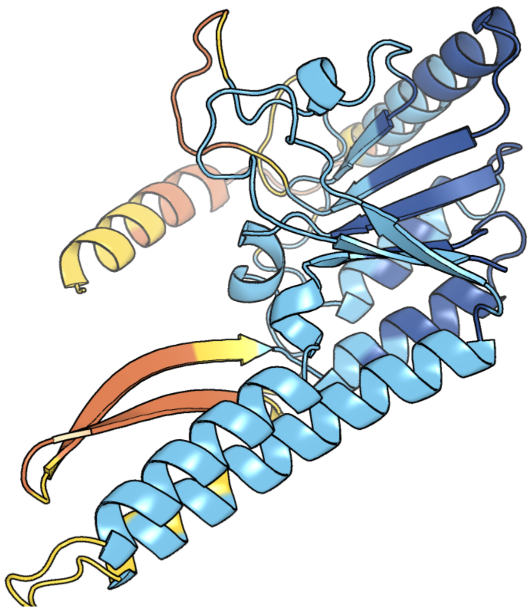

Figure 497: predicted structure of chlorv-4..328

chlorv-4..331

- Sequence-based annotation for chlorv-4..331 is
- No significant structural hit found

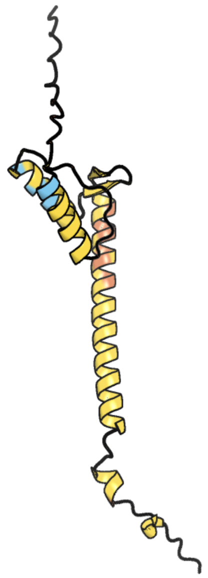

Figure 498: predicted structure of chlorv-4..331

chlorv-4..341

- Sequence-based annotation for chlorv-4..341 is
- No significant structural hit found

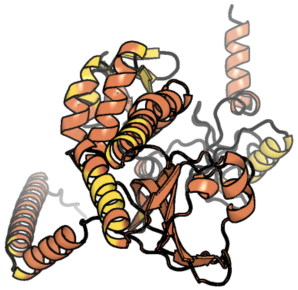

Figure 499: predicted structure of chlorv-4..341

chlorv-4..354

- Sequence-based annotation for chlorv-4..354 is
- No significant structural hit found

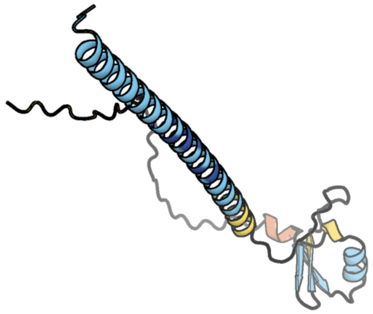

Figure 500: predicted structure of chlorv-4..354

chlorv-4..381

- Sequence-based annotation for chlorv-4..381 is
- No significant structural hit found

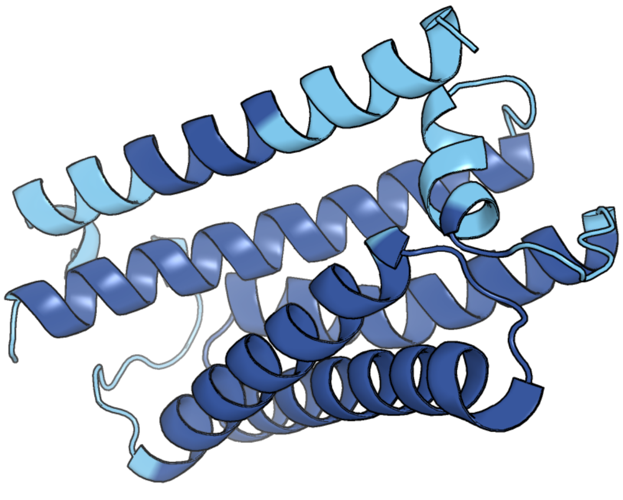

Figure 501: predicted structure of chlorv-4..381

chlorv-4..390

- Sequence-based annotation for chlorv-4..390 is
- No significant structural hit found

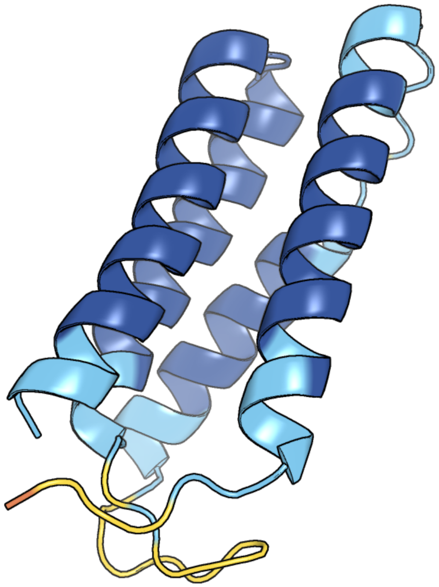

Figure 502: predicted structure of chlorv-4..390

chlorv-4..391

- Sequence-based annotation for chlorv-4..391 is
- Best hit was 3oy7 chain B: Glycosyltransferase B736L

target	prob	fident	alnlen	evalue	theader
3oy7-assembly1.cif.gz_B	1	0.129	531	5.98e-19	Crystal structure of a virus encoded glycosyltransferase in complex with GDP-mannose
7mi0-assembly1.cif.gz_A	1	0.138	355	1.336e-12	Crystal Structure of Glycosyltransferase from Rickettsia africae ESF-5
3l01-assembly2.cif.gz_B	1	0.103	404	6.223e-12	Crystal structure of monomeric glycogen synthase from Pyrococcus abyssi

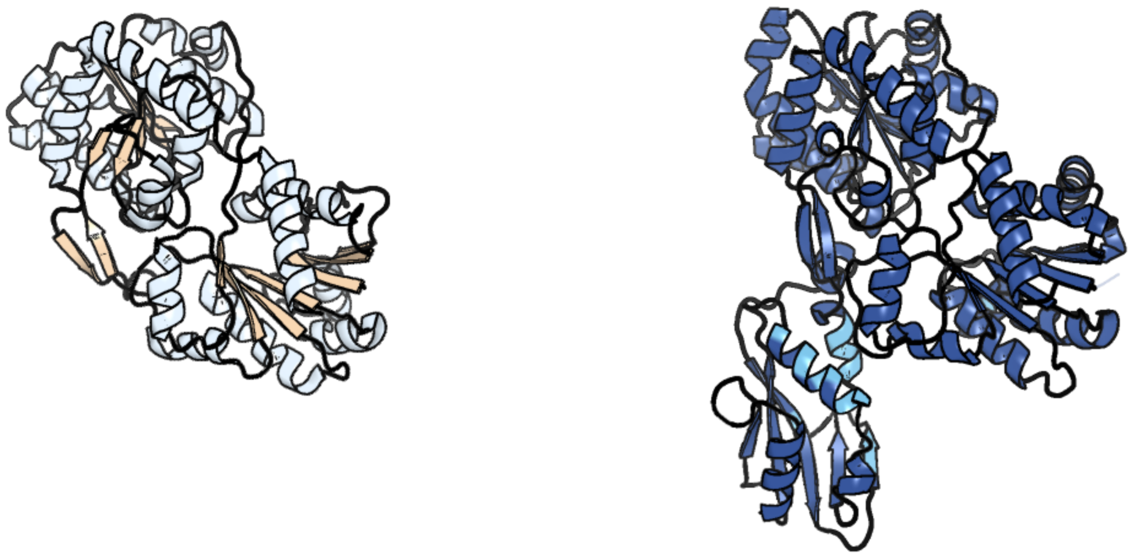

Figure 503: left: reference structure of 3oy7 chain B. right: predicted structure of chlorv-4..391, unaligned sequences are shown as transparent

chlorv-4..393

- Sequence-based annotation for chlorv-4..393 is
- No significant structural hit found

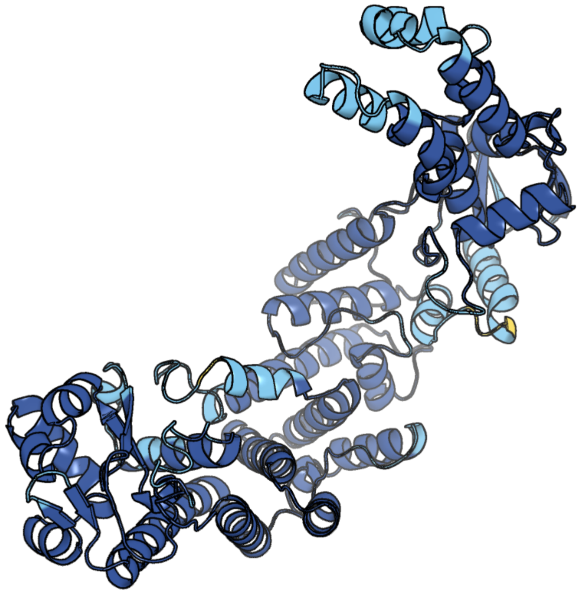

Figure 504: predicted structure of chlorv-4..393

chlorv-4..394

- Sequence-based annotation for chlorv-4..394 is
- Best hit was 5l9b chain A: Egl nine homolog 1

target	prob	fident	alnlen	evalue	theader
5l9b-assembly1.cif.gz_A	1	0.128	210	1.335e-06	HIF PROLYL HYDROXYLASE 2 (PHD2/ EGLN1) IN COMPLEX WITH 2-OXOGLUTARATE (2OG) AND HIF-1ALPHA CODD (556-574)
6f0w-assembly1.cif.gz_A	1	0.12	216	1.577e-06	prolyl hydroxylase in complex with hypoxia inducible factor oxygen degradation domain peptide fragment from Trichoplax adhaerens
4j25-assembly7.cif.gz_G	1	0.095	209	1.667e-06	Crystal structure of a Pseudomonas putida prolyl-4-hydroxylase (P4H)

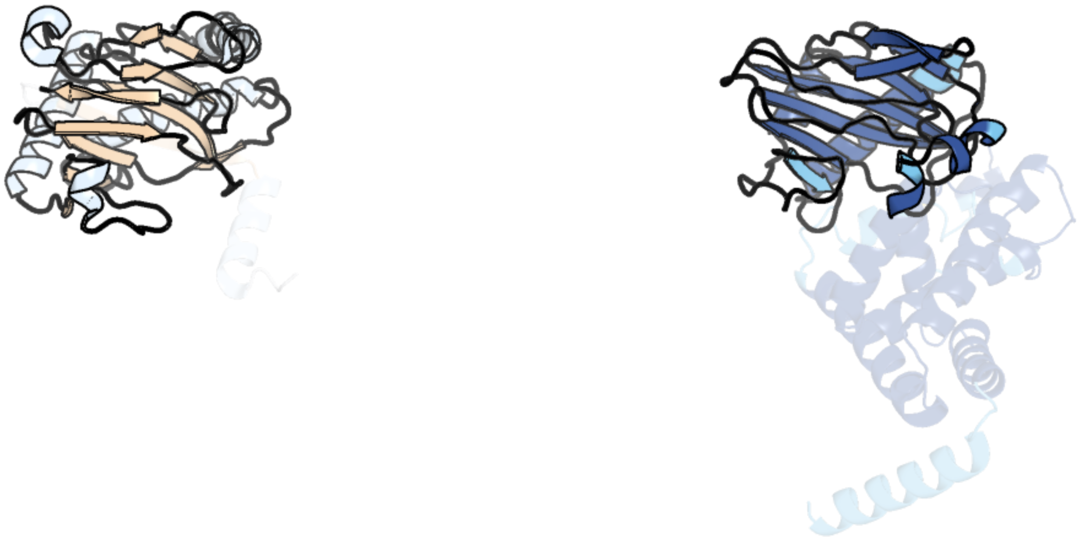

Figure 505: left: reference structure of 5l9b chain A. right: predicted structure of chlorv-4..394, unaligned sequences are shown as transparent
